# Rapid and Scalable Halosulfonylation of Strain‐Release Reagents**

**DOI:** 10.1002/anie.202213508

**Published:** 2022-11-10

**Authors:** Helena D. Pickford, Vasyl Ripenko, Ryan E. McNamee, Serhii Holovchuk, Amber L. Thompson, Russell C. Smith, Pavel K. Mykhailiuk, Edward A. Anderson

**Affiliations:** ^1^ Chemistry Research Laboratory Department of Chemistry University of Oxford 12 Mansfield Road Oxford OX1 3TA UK; ^2^ Enamine Ltd Chervonotkatska 78 02094 Kyiv Ukraine; ^3^ Chemistry Department Taras Shevchenko National University of Kyiv Volodymyrska 64 01601 Kyiv Ukraine; ^4^ AbbVie Drug Discovery Science & Technology (DDST) 1 North Waukegan Road North Chicago IL 60064 USA

**Keywords:** Bicyclic Compounds, Bioisosteres, Small-Ring Systems, Strained Molecules, Sulfonylation

## Abstract

Sulfonylated aromatics are commonplace motifs in drugs and agrochemicals. However, methods for the direct synthesis of sulfonylated non‐classical arene bioisosteres, which could improve the physicochemical properties of drug and agrochemical candidates, are limited. Here we report a solution to this challenge: a one‐pot halosulfonylation of [1.1.1]propellane, [3.1.1]propellane and bicyclo[1.1.0]butanes that proceeds under practical, scalable and mild conditions. The sulfonyl halides used in this chemistry feature aryl, heteroaryl and alkyl substituents, and are conveniently generated in situ from readily available sulfinate salts and halogen atom sources. This methodology enables the synthesis of an array of pharmaceutically and agrochemically relevant halogen/sulfonyl‐substituted bioisosteres and cyclobutanes, on up to multidecagram scale.

## Graphical abstract

A one‐pot halosulfonylation of strained hydrocarbons is described that proceeds under practical, scalable and mild conditions. Sulfonyl halides featuring aryl, heteroaryl and alkyl substituents are generated in situ from sulfinate salts and convenient halogen atom sources. This chemistry enables the synthesis of an array of halogen/sulfonyl‐substituted bioisosteres and cyclobutanes, on up to multidecagram scale. Hal=Halogen.

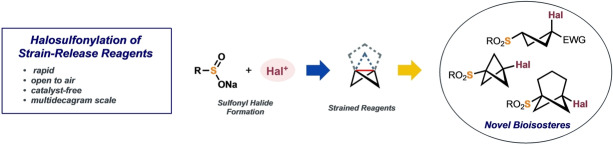

## Introduction

**Figure 1:**
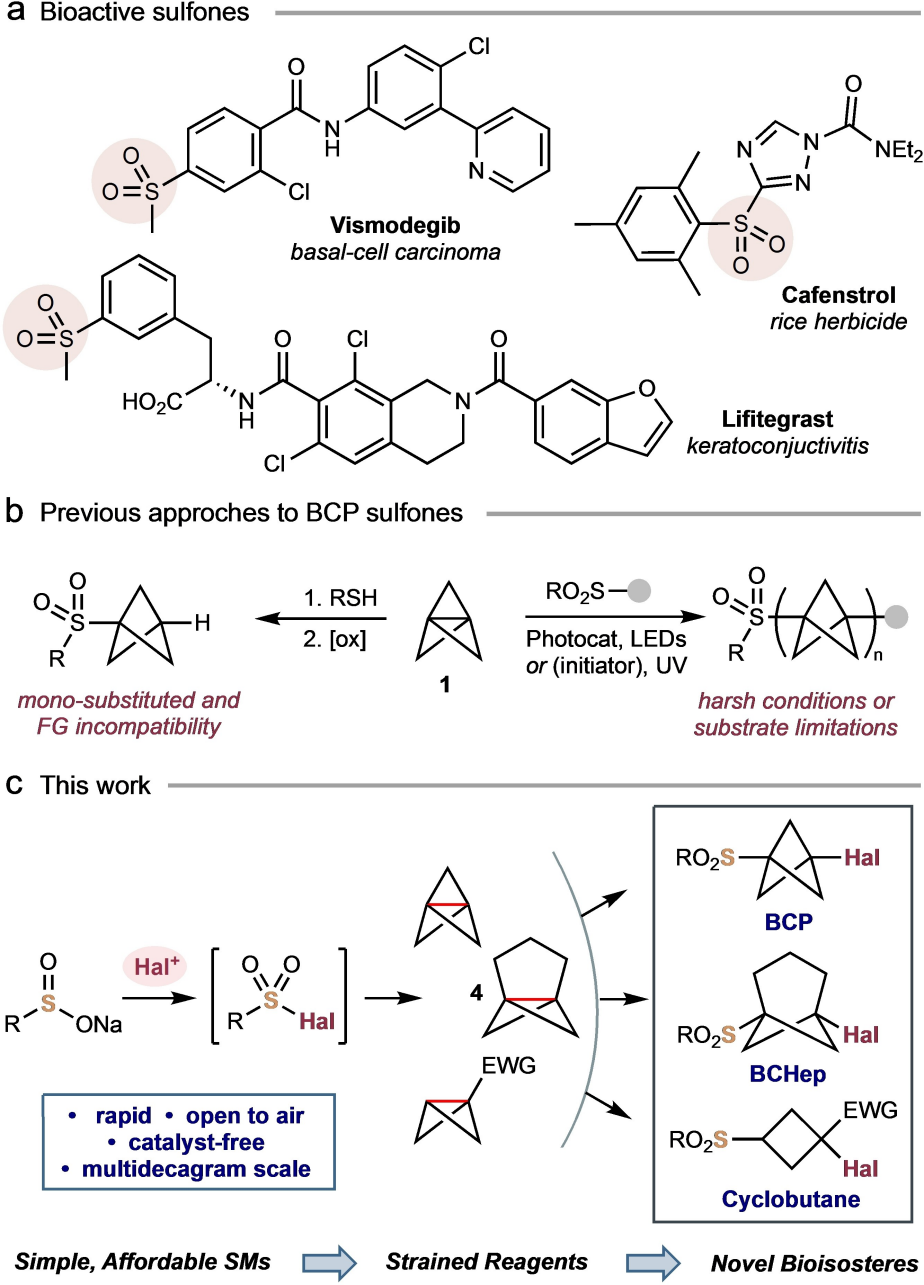
a) Bioactive aryl sulfones. b) Existing syntheses of sulfonyl BCPs. c) This work: A scalable in situ formation of sulfonyl halides for addition reactions across strained hydrocarbons.

Aryl sulfones are prevalent motifs in pharmaceutical and agrochemical compounds, such as the basal carcinoma treatment vismodegib, the rice herbicide cafenstrol, and the conjunctivitis treatment lifitegrast (Figure 1a).^[1]^ As such, methods for their synthesis are in high demand.^[2]^ The development of sulfonylated bioisosteres of aromatic rings, which could improve physicochemical and pharmacokinetic properties, is therefore an important goal in drug and agrochemical discovery.^[3]^ Methodologies to access such compounds are hence of significant interest, as demonstrated by the emergence of sulfonyl bicyclo[1.1.1]pentanes (BCPs) and cyclobutanes in medicinal chemistry patents.^[4]^ While monosubstituted BCP sulfones can be efficiently prepared through oxidation of BCP thioethers (Figure 1b, left),[^4a^, ^5^] preparation of the disubstituted sulfonyl BCPs reported to date has required lengthy syntheses involving manipulation of BCP 1,4‐dicarboxylic acid.[^4a^, ^4b^, ^4c^, ^4d^] In short, concise and convenient methods to synthesize disubstituted BCP sulfones (and similar structures) remain elusive.

The direct addition of sulfonyl groups to [1.1.1]propellane **1** offers an attractive method to achieve these sought‐after compounds (Figure 1b, right). This approach has been explored in the addition of certain sulfonyl chlorides (R=Me, Ph) to **1**, but required harsh UV irradiation, and suffered from poor to average yields and/or uncontrolled reactivity that generated ′staffane′ oligomers.^[6]^ Thioether‐ and selenoether‐substituted BCP sulfones have been prepared in a similar manner through mild heating or irradiation of dichalcogenides,^[7]^ as well as penta‐ and tetra‐fluoro(aryl)sulfanyl chlorides.^[8]^ *S,C‐*disubstituted BCP sulfones bearing β‐carbonyl, allyl or alkynyl substituents are accessible using photocatalysis; however, this method utilizes non‐commercial, tailored substrates such as enol sulfonate esters or alkynyl sulfones.^[9]^

We recently demonstrated the suitability of [1.1.1]propellane and [3.1.1]propellane to engage in atom transfer radical addition (ATRA) reactions with carbon and nitrogen‐centred radicals to afford halide‐functionalized *para‐* ^[10]^ and *meta‐*arene bioisosteres.^[11]^ However, equivalent methodologies to access sulfonyl BCP and bicyclo[3.1.1]heptane (BCHep) iodides and bromides are so far unknown. Here, we exploit sulfonyl halides, which can be easily generated in situ from readily available sulfinate salts, to rapidly construct difunctionalized sulfonyl arene bioisosteres via direct radical addition to strained hydrocarbon reagents (Figure 1c). This chemistry proceeds in high yields in as little as a few minutes and is scalable to multidecagram quantities. We further disclose that the resultant halide and sulfone provide convenient handles for product diversification through radical and anionic C−C bond formation.[^10b^, ^12^]

## Results and Discussion

Our initial goal was to achieve an efficient radical addition of sulfonyl halides with [1.1.1]propellane **1**. Several challenges were anticipated, such as desulfonylation or elimination of the sulfonyl halide, background reaction of **1** with halogenating reagents to form di‐halo BCPs, and oligomerization to form staffane byproducts. With these issues in mind, we designed a protocol to generate sulfonyl halides in situ from readily available sulfinate salts with electrophilic halogen sources, prior to the addition of **1** (Table 1). Using sodium toluenesulfinate **2** **a**, reaction with *N*‐iodosuccinimide (NIS) or ICl and **1** gave encouraging yields of sulfone BCP iodide **3** **a‐I** (41 % and 49 % respectively, entries 1 and 2), which increased to 78 % using 1,3‐diiodo‐5,5‐dimethylhydantoin (DIH, entry 3). Other iodinating reagents such as 2,2‐diiododimedone (DID) or I_2_ offered no improvement (entries 4–5), also affording rearranged *exo*‐cyclobutene products or, in the latter case, di‐iodinated BCP.

**Table 1:** Optimization of halosulfonylation of **1**.^[a]^

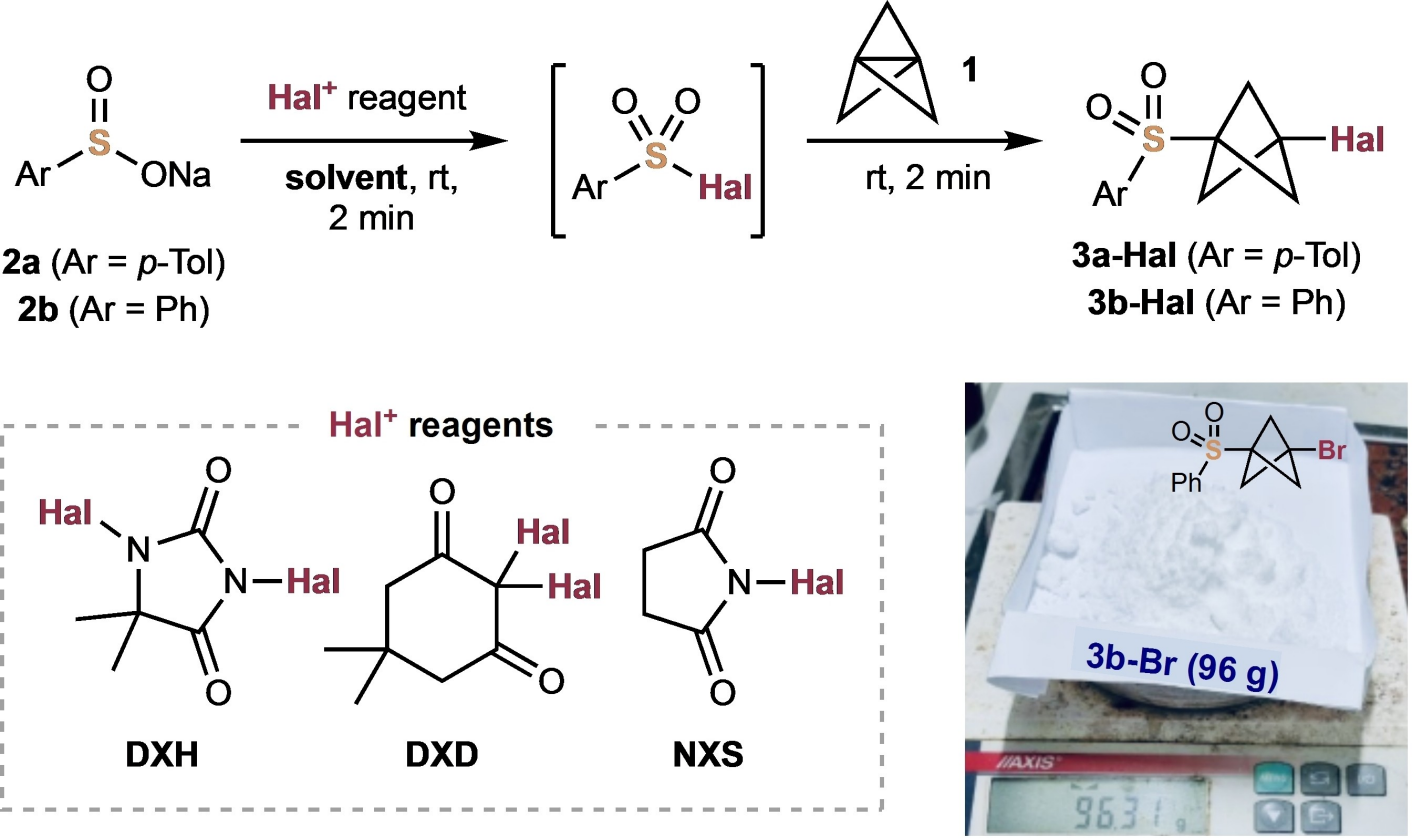				
				
Entry	Substrate	Hal^+^	Solvent	Yield **3** **a** or **3** **b** [%]^[b]^
1	**2** **a**	NIS	THF	41
2	**2** **a**	ICl	THF	49
3	**2** **a**	DIH	THF	78
4	**2** **a**	DID	THF	31
5	**2** **a**	I_2_	THF	7^[c]^
6	**2** **a**	DIH	Et_2_O	quant.
7	**2** **a**	DIH	H_2_O	99
8	**2** **a**	DIH	CH_2_Cl_2_	quant.^[d]^
9^[e]^	**2** **a**	DIH	Et_2_O	nr
10	**2** **a**	DBH	Et_2_O	9
11	**2** **a**	DBH	Et_2_O	quant.^[d,f]^
12	**2** **a**	NBS	Et_2_O	50^[f]^
13	**2** **b**	I_2_	Et_2_O	91^[d,g]^
14	**2** **b**	Br_2_	Et_2_O	97^[d,g]^

[a] Optimization carried out on 0.15 mmol scale, with 2.5 equiv sulfinate (1.0 M in H_2_O), 1.0 equiv of halogenating agent and 1.0 equiv of **1** (0.75 M in Et_2_O). [b] ^1^H NMR yields calculated with mesitylene as internal standard. [c] 18 % di‐iodo BCP observed. [d] Isolated yield. [e] Reaction run in the dark. [f] 18 h reaction time after addition of **1**. [g] 1.0 equiv of pre‐isolated sulfonyl halide and 1.3 equiv of **1** were reacted in Et_2_O for 15 h at rt. X/Hal=Halogen. DXH=dihalohydantoin. DXD=dihalodimedone. NXS=*N*‐halosuccinimide *p*‐Tol=*para*‐tolyl. nr = no reaction.

Proceeding with DIH as the iodine source, we found that the reaction solvent significantly influenced the yield of **3** **a‐I**: THF was surpassed by solvents such as Et_2_O, CH_2_Cl_2_ and H_2_O, all of which gave the desired **3** **a‐I** in near quantitative yield with only trace staffane formation (entries 6–8). No reaction was observed in the absence of light, supporting a radical‐based mechanism (entry 9). Lower reaction temperatures (−5 °C) were equally efficient, and proved important in the subsequent substrate evaluation.^[13]^ We were pleased to find that on switching the halogenating reagent to 1,3‐dibromo‐5,5‐dimethylhydantoin (DBH), these conditions could also be applied to the formation of sulfonyl BCP bromide **3** **a‐Br**. Interestingly, the intermediate sulfonyl bromide was formed within 2 min, but an extended reaction time of 18 h was required for complete reaction with **1** (entries 10 and 11), the latter affording **3** **a‐Br** in quantitative yield. NBS also proved a suitable brominating agent for the sulfinate salt (entry 12). The chemistry could be applied on multidecagram scale, highlighting the robust nature of the addition; for example, phenylsulfonyl BCP iodide **3** **b‐I** could be obtained in 91 % yield on 34 g scale (entry 13), while the BCP bromide **3** **b‐Br** was isolated in 97 % yield on 96 g scale (entry 14). In these cases, the sulfonyl halide intermediate was pre‐isolated using I_2_ and Br_2_ as halogen sources before reaction with **1**.

**Figure 2:**
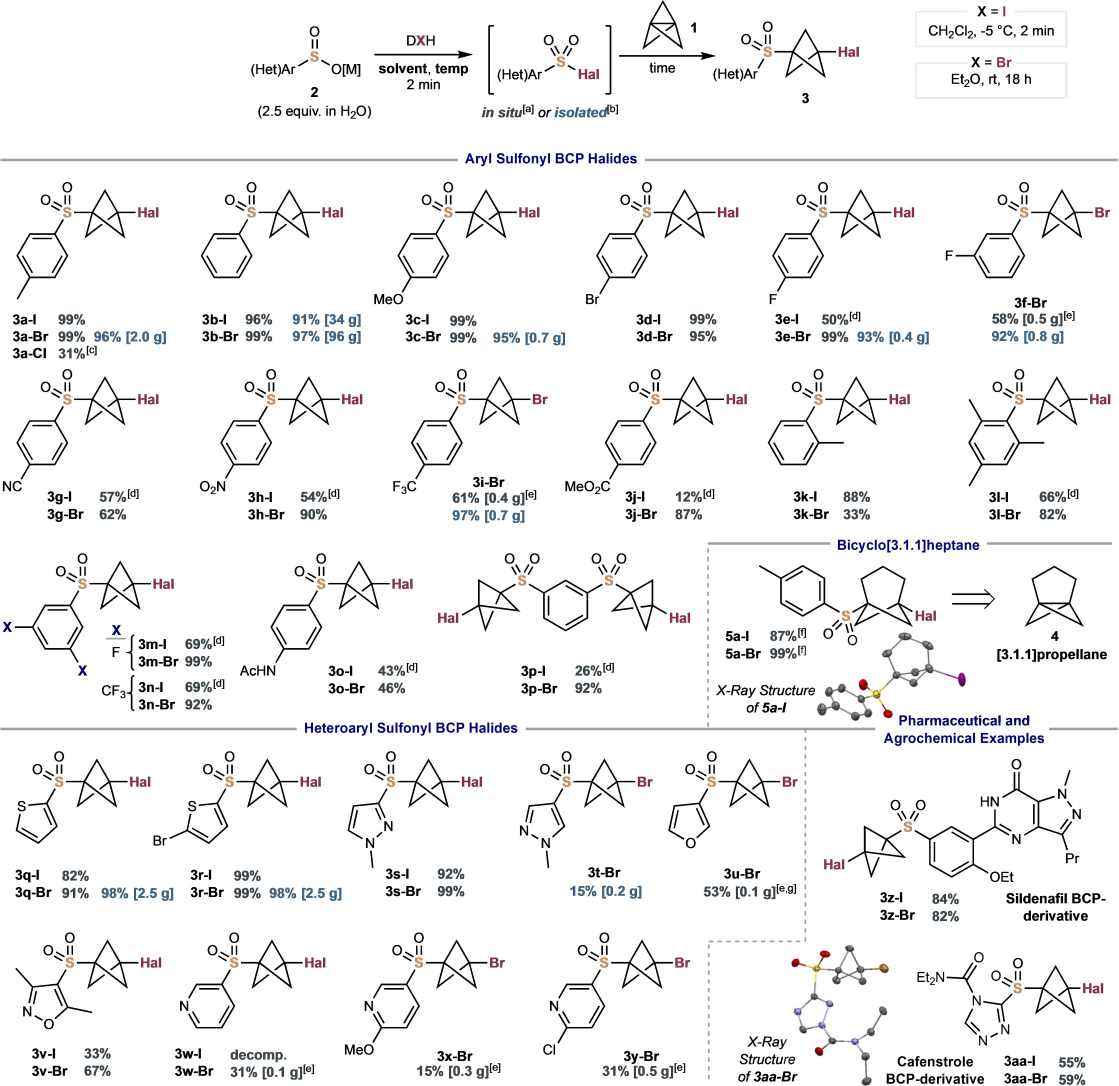
Scope of halosulfonylation of [1.1.1] and [3.1.1]propellane with aryl and heteroaryl sulfinates, [M]=Na or Li. [a] Yields in grey represent sulfonyl halides prepared in situ from 2.5 equiv of **2** (1.0 M in H_2_O) and 1.0 equiv of DIH/DBH/DCH, then 1.0 equiv of **1** (0.70–0.75 M in Et_2_O) according to the title scheme on a 0.2 mmol scale. [b] Yields in blue represent use of 1.0 equiv of isolated sulfonyl halide and 1.3 equiv of **1** in Et_2_O at rt for 15 h. [c] DCH instead of DBH, 10 mol % Et_3_B, 2 h, rt. [d] **2** (1.0 M in DMF) was iodinated at −40 °C; after addition of **1**, stirred at −40 °C for 20 min, then rt, 10 min. [e] 1.0 equiv of **2** was brominated using 1.0 equiv of NBS in MeCN at rt for 30 min; then 1.5 equiv of **1**, 15 h. [f] 1.0 equiv of [3.1.1]propellane **4** (0.23 M in Et_2_O) instead of **1**. [g] 0.95 equiv of PPh_3_⋅Br_2_, 1.0 equiv of **2** and 1.5 equiv of **1** in MeCN for 15 h at rt. Structure of **5** **a‐I** and **3** **aa‐Br** from X‐ray diffraction studies, displacement ellipsoids are drawn at 50 % probability and hydrogen atoms are omitted for clarity.^[14]^

With conditions in hand for iodo‐ and bromosulfonylation of **1**, the scope of the reaction was studied (Figure 2). Aryl sulfinates bearing neutral or electron‐donating substituents (**2** **a**–**2** **c**) gave the corresponding sulfone BCP iodides and bromides **3** **a**–**3** **c** in near quantitative yields (91–99 %, up to ≈2 g scale), although the equivalent chlorosulfonylation proved lower yielding (**3** **a‐Cl**, 31 %).

Halogenated arenes were well‐tolerated, particularly for BCP bromide synthesis (**3** **d**–**3** **f**, 50–99 %). Good to excellent yields were also observed for electron‐deficient aryl sulfonyl bromides **3** **g‐Br**–**3** **j‐Br** (for example, the *p‐*CF_3_ aryl sulfone **3** **i‐Br** was obtained in 97 % yield from the isolated sulfonyl bromide intermediate on 0.7 g scale, or in 61 % yield using a one‐pot procedure); however the corresponding iodides performed less well, and typically benefited from cooling to −40 °C to limit competing desulfonylation. Sterically‐encumbered *o‐*tolyl and mesityl sulfinates proceeded smoothly, giving BCP iodide **3** **k‐I** in 88 % yield, and mesityl adducts **3** **l‐I** and **3** **l‐Br** in 66 % and 82 % yield respectively. A lower yield of **3** **k‐Br** was observed (33 %) due to competing benzylic bromination of the methyl group. Additional *m‐*electron‐withdrawing substituents were also accommodated, with 3,5‐di‐F and 3,5‐di‐CF_3_ arenes affording sulfonyl BCP halides **3** **m** and **3** **n** in 69–99 % yield. The inferior reactivity observed with acetamide **2** **o** was surprising, even on cooling to −40 °C, possibly due to competing reaction at the amide. A bis‐arylsulfinate could even be used successfully to deliver bis‐sulfonyl BCP iodide **3** **p‐I** and bromide **3** **p‐Br** in 26 % and 92 % yield respectively. [3.1.1]propellane **4** is emerging as a convenient reagent to access novel *m*‐substituted arene bioisosteres;^[11]^ pleasingly, the addition of sulfonyl halides translated smoothly to this propellane, giving sulfonyl BCHep halide products in excellent yields (**5** **a‐I**, 87 % and **5** **a‐Br**, 99 %).

Many heteroaryl sulfonyl halides excelled under these protocols. Electron‐rich 5‐membered heterocyclic sulfinates performed best, such as thiophene **2** **q** and 2‐bromothiophene **2** **r**, with yields ranging from 82–99 % for both the iodide and bromide products. The thiophene sulfonyl BCP bromides could be prepared on 2.5 g scale from the isolated sulfonyl bromides with equally impressive 98 % yields for both substrates. 3‐Pyrazole sulfinate **2** **s** was similarly successful, giving the BCP iodide and bromide **3** **s‐I**/**Br** in 92 % and 99 % yields, respectively, whereas 2‐pyrazole sulfinate gave a poorer yield of the bromide **3** **t‐Br** (15 %). 3‐Furyl sulfonyl BCP bromide **3** **u‐Br** was achieved in 53 % yield using PPh_3_⋅Br_2_ complex to prepare the sulfonyl bromide in situ. Substrates containing *N*‐nucleophilic sites proved challenging, presumably due to competing reactivity with the sulfonyl halide:^[15]^ oxazole **2** **v** gave a low yield of the BCP iodide **3** **v‐I** (33 %) but a 67 % yield of the bromide **3** **v‐Br**. Iodosulfonylation of pyridylsulfinate **2** **w** was unsuccessful, while the pyridyl sulfonyl bromide adducts **3** **w‐Br**–**3** **z‐Br** were isolated in reduced yields of 15–31 %, on up to 0.5 g scale. Pleasingly, we found that the methodology could be readily applied in pharmaceutical and agrochemical settings, successfully achieving BCP derivatives of the vasodilator sildenafil (**3** **z‐I**, 84 % and **3** **y‐Br**, 82 %) and the rice herbicide cafenstrole (**3** **aa‐I**, 55 % and **3** **aa‐Br**, 59 %).

**Figure 3:**
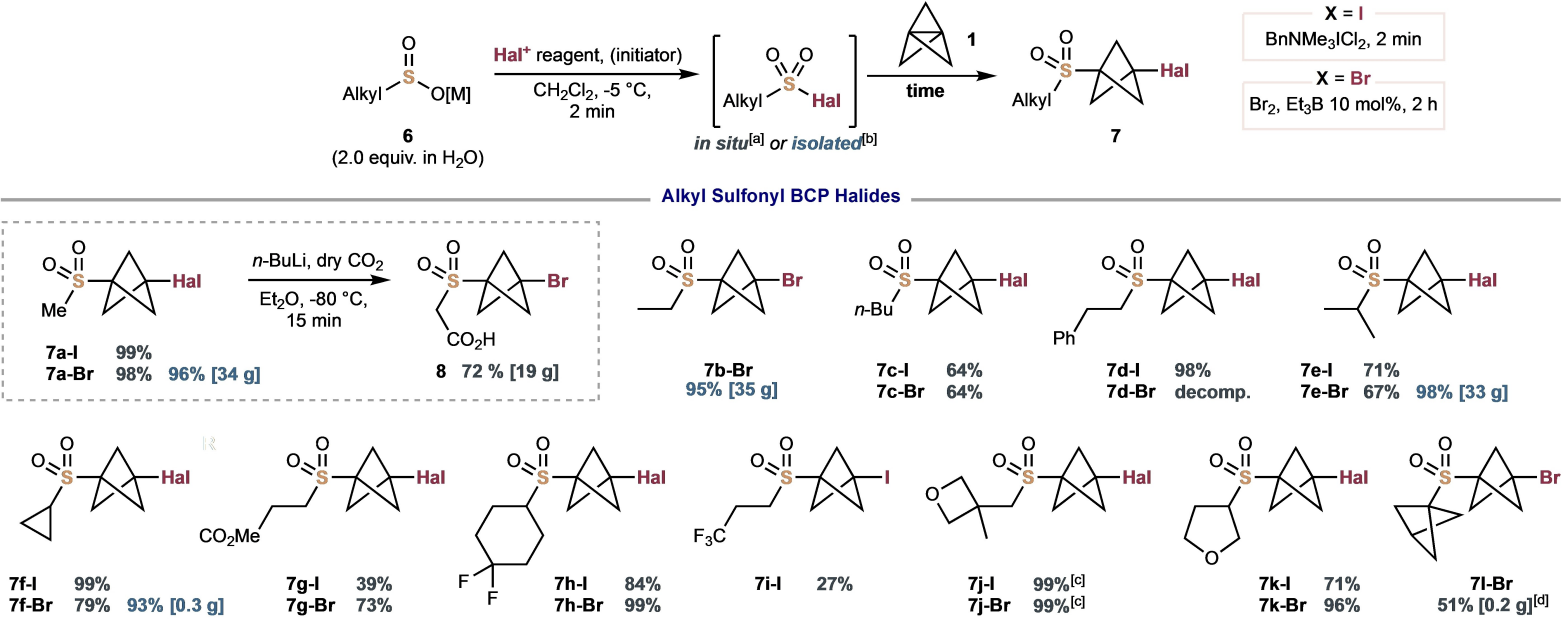
Scope of alkyl sulfonyl BCP halides. [a] Yields in grey represent sulfonyl halides prepared in situ from 2.0 equiv of **6** (1.0 M in H_2_O, [M]=Na or Li) and 1.0 equiv of **1** (0.70–0.75 M in Et_2_O) according to the title scheme on a 0.1 mmol scale. BCP iodides: 1.4 equiv of BnNMe_3_ICl_2_ was used. BCP bromides: 1.8 equiv of Br_2_ with 10 mol % of Et_3_B was used. [b] Yields in blue represent use of 1.0 equiv of isolated sulfonyl halide and 1.3 equiv of **1**, in Et_2_O for 15 h at rt, without initiator. [c] 0.5 μmol scale. [d] 0.95 equiv of PPh_3_⋅Br_2_, 1.0 equiv of **6** and 1.5 equiv of **1** in MeCN at rt for 15 h.

In contrast to (hetero)arylsulfonyl halides, the generation of alkylsulfonyl iodides and bromides is challenging due to their tendency to undergo rapid elimination to form HI/HBr and SO_2_.^[16]^ We were encouraged to find that our standard conditions using dihalohydantions as halogen sources enabled the formation of alkyl sulfonyl halides in situ; however, rather than the desired addition reaction, only *N*‐sulfonylation of the hydantoin byproduct was observed on addition of [1.1.1]propellane **1**. Further evaluation of suitable halogenating agents identified BnMe_3_N^+^ICl_2_^−^ and Br_2_ as convenient alternatives that enabled the smooth halo‐sulfonylation of **1** (Figure 3). Typically, alkylsulfonyl iodides added rapidly to **1** (within 2 min), whereas alkylsulfonyl bromides benefited from addition of Et_3_B (10 mol %) to obtain optimal yields in just two hours on small scales; the latter adducts could also be synthesised on decagram scale over a 15 h period without an initiator. Primary alkyl sulfinates gave good to excellent yields of sulfonyl BCP bromides (**7** **a‐Br**–**7** **c‐Br**, 64–98 %), where the methyl and ethyl sulfonyl products were also synthesized in near quantitative yields on >30 g scale from the isolated sulfonyl bromides. Equivalent sulfonyl BCP iodides **7** **a‐I** and **7** **c‐I** were also generated in high yields (99 % and 64 % respectively). These compounds provide valuable opportunities for further sulfone‐based reactivity; for example, deprotonation/CO_2_ quench of BCP bromide **7** **a‐Br** delivered carboxylic acid **8** in 72 % yield on 25 g scale, which could be a useful building block in drug discovery by analogy to its bioisosteric phenyl counterpart.^[17]^ The reaction of phenylethyl sulfinate gave the BCP iodide **7** **d‐I** in an excellent 98 % yield; unfortunately, attempted bromosulfonylation resulted in decomposition. Secondary alkyl sulfinates performed admirably, such as isopropyl and cyclopropyl sulfones (**7** **e** and **7** **f**, 67–99 %); notably, the isopropyl sulfone BCP bromide **7** **e‐Br** was afforded in an excellent 96 % yield on 33 g scale. Methyl propanoate BCP sulfone **7** **g** was synthesised as both the iodide (**7** **g‐I**, 39 %) and the bromide (**7** **g‐Br**, 73 %). Fluoroalkyl sulfinates were also tolerated: difluorocyclohexyl sulfone **7** **h** was isolated in excellent yields as both the BCP iodide and bromide, (84 % and 99 % respectively). Interestingly, an acyclic fluoroalkyl sulfinate proved less efficient (**7** **i‐I**, 27 %). Oxacycle‐containing sulfinates were also accommodated: oxetane BCP iodide **7** **j‐I** and bromide **7** **j‐Br** were afforded in near‐quantitative yields, while tetrahydrofuran‐substituted sulfone **7** **k** was prepared as the BCP iodide and bromide in 71 % and 96 % yield, respectively. Finally, the bis‐BCP sulfone bromide **7** **l‐Br** was achieved in 51 % yield with PPh_3_⋅Br_2_ as the brominating agent.

**Scheme 1:**
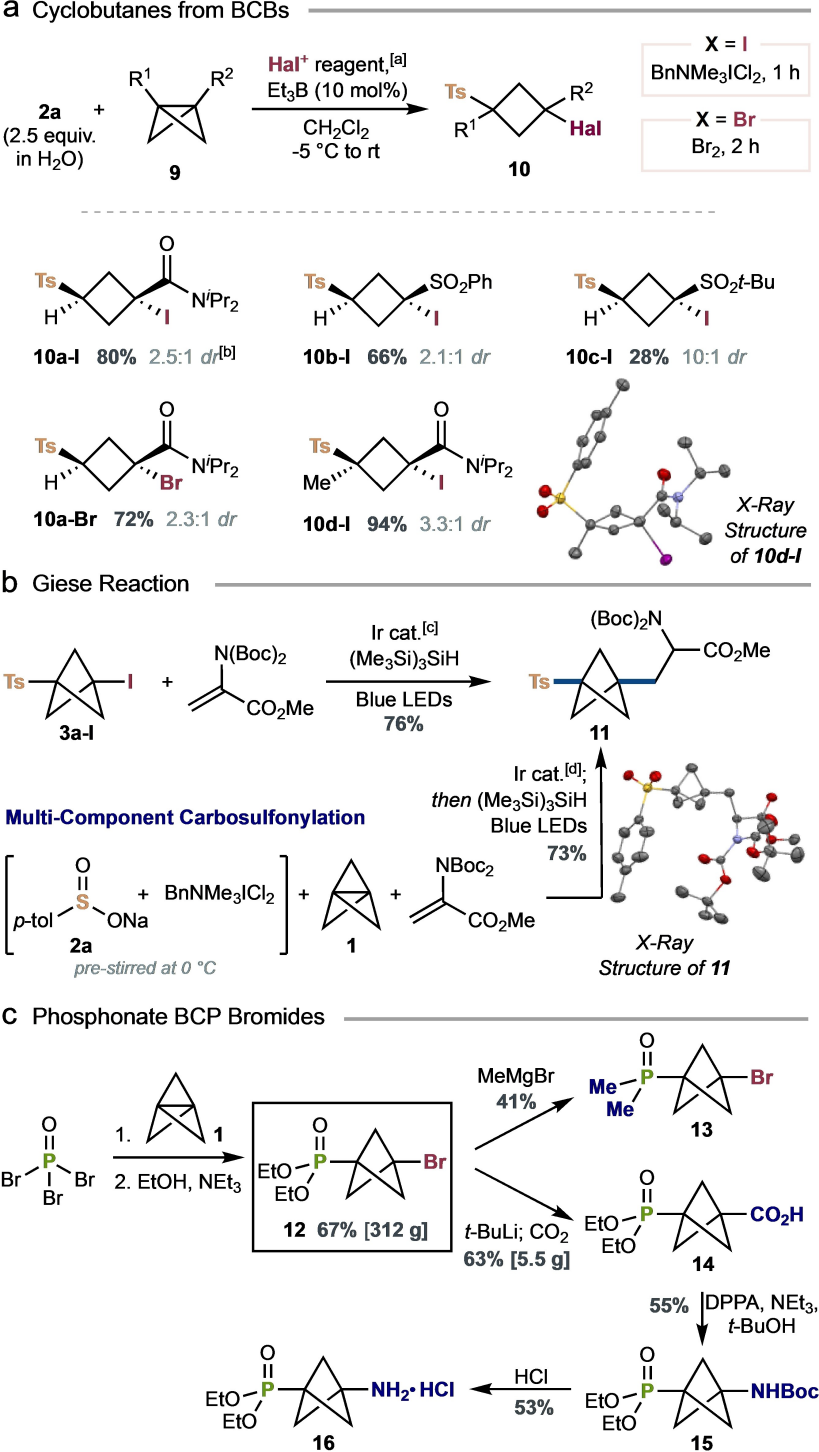
a) Addition of sulfonyl halides to BCBs. b) Giese reaction of a sulfonyl BCP iodide. c) Synthesis and functionalization of phosphonate BCP bromides (see the Supporting Information for detailed reaction conditions). [a] Reactions carried out on a 0.1 mmol scale according to the title scheme, using 2.5 equiv of **2** **a** (1.0 M in H_2_O) and either 1.4 equiv of BnNMe_3_ICl_2_ or 1.8 equiv of Br_2_, stirred for 2 min before addition of 1.0 equiv of BCB **9** (0.1 M in CH_2_Cl_2_) and 10 mol % Et_3_B. [b] The major diastereoisomer of **10** **b‐I** was assigned by NOESY correlations; other adducts were assigned by comparison of ^1^H NMR spectra. [c] 2.5 mol % Ir[(dF(CF_3_)ppy)_2_(dtbbpy)]PF_6_, 2.0 equiv of Na_2_CO_3_, 2.0 equiv of (Me_3_Si)_3_SiH, 6.0 equiv, of dehydroalanine, MeOH/H_2_O (1 : 1), blue LEDs, rt, 18 h. [d] 2.5 equiv of **2** **a** (1.0 M in H_2_O) and 1.4 equiv of BnNMe_3_ICl_2_ in CH_2_Cl_2_ were pre‐stirred at 0 °C for 2 min, then added to a vial containing reagents as in [c] and 1.0 equiv of **1**; (Me_3_Si)_3_SiH was added last. Structure of **10** **d‐I** and **11** from X‐ray diffraction studies, displacement ellipsoids are drawn at 50 % probability, hydrogen atoms and disordered solvent (**11** only) are omitted for clarity.^[14]^ Boc=*tert*‐butoxycarbonyl. DPPA=diphenylphosphoryl azide.

With successful additions to [1.1.1] and [3.1.1]propellane established, we questioned whether other strained hydrocarbons might undergo successful halosulfonylation. To our delight, bicyclo[1.1.0]butanes (BCBs) **9** proved highly suitable reagents for this chemistry, in the presence of 10 mol % Et_3_B initiator (Scheme 1a). Although BCBs have been subject to the addition of alkyl radicals, α‐amino radicals, and thiols, previous work has exclusively involved monosubstituted BCBs;^[18]^ to our knowledge, the addition of sulfonyl radicals has not been reported. Tosyl iodide, generated in situ from sulfinate salt **2** **a**, underwent smooth addition to monosubstituted BCBs **9** **a**–**c** to afford iodocyclobutyl sulfones substituted with amide (**10** **a‐I**, 80 %, 2.5 : 1 *dr*), aryl sulfone (**10** **b‐I**, 66 %, 2.1 : 1 *dr*), and alkyl sulfone (**10** **c‐I**, 28 %, 10 : 1 *dr*) groups. Tosyl bromide engaged in an analogous addition to BCB **9** **a** to give the sulfonylated bromocyclobutane **10** **a‐Br** in 72 % yield (2.3 : 1 *dr*). Most pleasingly, sulfonyl addition to disubstituted BCB **9** **d** returned an exceptional yield of 94 % of the iodo‐sulfonylated cyclobutane **10** **d‐I** (3.3 : 1 *dr*), in what represents the first addition of any radical to a disubstituted BCB.

The halide resident in the BCP, BCHep and cyclobutyl products offer many opportunities for further chemistry. As such, we were excited to observe the successful photocatalyzed Giese addition of tosyl BCP iodide **3** **a‐I** to a dehydroalanine derivative, giving the α‐amino acid analogue **11** in an excellent 76 % yield (Scheme 1b).^[10b]^ A multi‐component carbosulfonylation of **1** directly from sulfinate **2** **a** proved equally productive, providing **11** in 73 % yield. Finally, we studied the potential of this chemistry to extend beyond sulfur‐centered radicals, and identified phosphonate functionalities, which are prevalent within drug discovery—for example, as antivirals, pro‐nucleotides, and farnesyl pyrophosphate synthase inhibitors (Scheme 1c).^[19]^ In the event, the direct addition of phosphoryl tribromide to **1**, followed by reaction of the intermediate dibromide with ethanol, enabled the synthesis of 312 g of phosphonate BCP bromide **12** (67 % yield). This versatile BCP building block could be converted to the phosphine oxide **13** on treatment with MeMgBr (41 %), thereby offering a useful entry to bioisosteres of phosphine oxide containing pharmaceuticals;^[20]^ or to the phosphoryl BCP carboxylic acid **14** via lithium‐halogen exchange and trapping of the resultant bridgehead carbanion with CO_2_ (63 % on 5.5 g scale). Accessed in just three steps from **1**, it is worth noting that the methyl ester of **14** has previously required seven steps for its preparation and use as a glutamate receptor ligand.^[21]^ Curtius rearrangement of **14** enabled the synthesis of phosphoryl carbamate BCP **15**, and then amino phosphoryl BCP salt **16** on treatment with HCl.

## Conclusion

In conclusion, we have developed a practical, efficient, and flexible methodology to directly construct sulfonyl and phosphonate BCP, BCHep and cyclobutyl halides from simple sulfinate salts and convenient electrophilic halogen sources. A wide range of substituents were tolerated, enabling the preparation of pharmaceutical and agrochemical analogues. The collection of heteroaryl and alkyl sulfone products represents a particularly significant advance in the functionality of sulfonyl halide reagents accessible to date. The reaction protocols are rapid and straightforward to execute, forgoing the need for anhydrous solvents, inert reaction conditions, or metal catalysts, and translate exceptionally to decagram and even hundred‐gram scales. Selected further manipulations of the products highlight their potential utility in the synthesis of novel medicinally‐relevant bioisosteres, which cannot be easily accessed by other means.

## Conflict of interest

The authors declare no conflict of interest.

1

## Supporting information

As a service to our authors and readers, this journal provides supporting information supplied by the authors. Such materials are peer reviewed and may be re‐organized for online delivery, but are not copy‐edited or typeset. Technical support issues arising from supporting information (other than missing files) should be addressed to the authors.

Supporting Information

Supporting Information

## Data Availability

The data that support the findings of this study are available in the supplementary material of this article.
