# Supplementary material for: Rapid and Scalable Halosulfonylation of Strain‐Release Reagents
Source: Angew Chem Int Ed Engl. 2022 Nov 10;62(3):e202213508. doi: 10.1002/anie.202213508 (PMC10100009; doi:10.1002/anie.202213508)
Supplement: Supplementary file 1 — Supporting Information [file ANIE-62-0-s002.pdf]

Angewandte Chemie
Eine Zeitschrift der Gesellschaft Deutscher Chemiker

Supporting Information

Rapid and Scalable Halosulfonylation of Strain-Release Reagents

H. D. Pickford, V. Ripenko, R. E. McNamee, S. Holovchuk, A. L. Thompson, R. C. Smith, P. K. Mykhailiuk, E. A. Anderson**

Supporting Information

1. General Experimental Details 3
   1.1 Experimental Considerations 3
   1.2 General Procedures for Halo-Sulfonylation (A – I) 5
   1.3 General Procedures for Starting Materials (J – N) 11
2. Experimental Data 13
   2.1 Aryl Sulfonyl BCP Halides 13
   2.2 Heteroaryl Sulfonyl BCP Halides 35
   2.3 Pharmaceutical and Agrochemical BCP Halides 43
   2.4 Alkyl Sulfonyl BCP Halides 46
   2.5 Sulfonyl Cyclobutyl Halides 58
   2.6 Functionalisation Products 64
   2.7 Miscellaneous Compounds 66
3. Phosphonate BCP Halides 70
   3.1 Phosphonate BCP Halides 70

4. Preparation of Starting Materials 74
4.1 Strained Reagents 74
4.2 (Het)Aryl Sulfonyl Halides 76
4.3 Alkyl Sulfonyl Bromides 81
4.4 Aryl Sulfinate Salts 83
4.5 Pharmaceutical and Agrochemical Sulfinate Salts 89
4.6 Heteroaryl Sulfinate Salts 94
4.7 Alkyl Sulfinate Salts 98
4.8 Unsuccessful Sulfinates 101
5. Additional Optimisation 106
5.1 Sulfonyl BCP Iodide Optimisation 106
5.2 Sulfonyl BCP Bromides and Chlorides 109
5.3 Instability of Sulfonyl Iodides 110
5.4 Alkyl Sulfonyl Bromides Optimisation 112
5.5 Alkyl Sulfonyl Iodides Optimisation 115
5.6 Control Reactions and Reaction Monitoring 117
6. NMR Spectra 119
6.1 Aryl Sulfonyl BCP Iodides 119
6.2 Heteroaryl BCP Iodides 162
6.3 Pharmaceutical and Agrochemical BCP Halides 175
6.4 Alkyl Sulfonyl BCP Halides 179
6.5 Sulfonyl Cyclobutyl Halides 202
6.6 Sulfonyl Halides 212
6.7 Sulfinate Salts 228
6.8 Unsuccessful Sulfinates 263
6.9 Functionalisation Products 270
6.10 Miscellaneous Compounds 272
6.11 Phosphonate BCP Halides 275
7. X-Ray Crystallography 285
7.1 X-Ray Crystal Structures 285
8. References 294

1. General Experimental Details

1.1 Experimental Considerations

NMR Spectroscopy

^{1}H, ^{13}C, ^{19}F, and ^{31}P NMR spectra were recorded on Bruker AVIIIHD, NEO or Varian spectrometers using TOPSPIN software, with the deuterated solvent acting as the internal deuterium lock. Chemical shifts are calibrated using standard residual undeuterated solvents. ^{1}H NMR spectra were recorded at 400, 500 or 600 MHz. ^{13}C NMR spectra were recorded at 101, 126 or 151 MHz with ^{1}H decoupling. ^{19}F NMR spectra were recorded at 376 or 470 MHz. ^{31}P NMR spectra were recorded at 126 or 202 MHz. Assignments were determined either on the basis of unambiguous chemical shift/coupling patterns, or from 2D COSY, HMBC, HSQC and/or NOESY experiments. Peak multiplicities are defined as: s = singlet, d = doublet, t = triplet, q = quartet, quin. = quintet, sxt. = sextet, hept. = septet, m = multiplet, br. = broad, app. = apparent, obsc. = obscured. Coupling constants (*J*) are reported to the nearest 0.1 Hz. ^{1}H NMR yields were calculated using mesitylene as an internal standard.

Infrared Spectroscopy

Infrared spectra were recorded on a Bruker Tensor 27 FT-IR spectrometer with the sample being prepared as a thin film on a diamond ATR module. Absorption maxima (ν_{max}) are quoted in wavenumbers (cm^{-1}).

Mass Spectrometry

High resolution mass spectra (HRMS) were recorded on a Bruker MicroTOF or Agilent LC/MSD TOF mass spectrometer by ESI, EI or CI experiments. High resolution values are calculated to 4 decimal places from the molecular formula, and all values are within a tolerance of 5.

Melting Points

Melting points were obtained using a Griffin melting point apparatus and are uncorrected.

Reagents, Solvents and Techniques

All reagents were used directly as supplied from commercial sources or Enamine Ltd. (www.enamine.net). Solvents were either used as commercially supplied, or as purified by standard techniques. Anhydrous Et_2O, MeCN, CH_2Cl_2, THF, DMF and toluene were obtained from solvent dispenser units having been passed through an activated alumina column under Ar. Unless otherwise stated, non-aqueous reactions were performed using flame-dried glassware under Nitrogen atmosphere. Reactions were monitored by thin layer chromatography (TLC) on pre-coated aluminium-backed plates (Merck Kieselgel 60 UV 254 or GF254, 0.2 mm). Compounds were visualized by quenching of UV fluorescence or by staining with potassium permanganate, vanillin, ninhydrin, goof or PMA. Column chromatography was performed on silica gel obtained from Merck (Silica gel Si 60, 0.040 – 0.063 mm or 100 – 200 mesh) under a positive pressure of Nitrogen, using the stated solvent system.

1.2 General Procedures for Halo-Sulfonylation (A – I)

Summary of Procedures

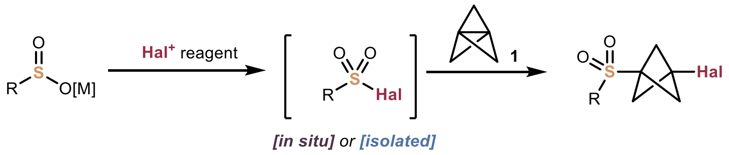

Procedure	*Notes*	*RSO$_2$-Hal*	*Hal*	*Reagents*	*Temp*
A	R = (Het)Aryl	In situ	I	DIH	-5 °C
B	R = (Het)Aryl – ED	In situ	I	DIH	-40 °C
C	R = (Het)Aryl	In situ	Br	DBH	rt
D	Multigram	Isolated	Br	-	rt
E	Multigram	In situ	Br	NBS	rt
F	R = Alkyl	In situ	I	$BnNMe_3ICl_2$	-5 °C
G	R = Alkyl	In situ	Br	Br_2	rt
H	BCB **9**	In situ	I	$BnNMe_3ICl_2$ / Et_3B	-5 °C
I	BCB **9**	In situ	Br	Br_2 / Et_3B	-5 °C

All procedures were carried out under air (unless stated otherwise), neither flame drying of glassware or use of anhydrous solvents were necessary. Purification of sulfonyl BCP halides with a short pad of silica was usually sufficient unless otherwise stated. Sulfonyl BCP halides could be re-crystallised from CH_2Cl_2/pentane at -18 °C to afford crystals of suitable quality for X-ray diffraction. *ED = electron-defficient.*

General Procedure A – *(Het)Aryl Sulfonyl BCP Iodides, R = (Het)Aryl and Hal = I*

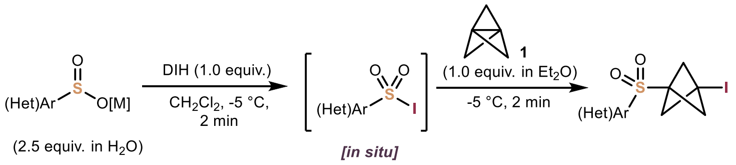

A solution of sulfinate salt (0.50 mL, 0.50 mmol, 1.0 M in H_2O, 2.5 equiv.) was added dropwise to a suspension of DIH (78.4 mg, 0.20 mmol, 1.0 equiv.) in CH_2Cl_2 (0.80 mL) at -5 °C (ice/salt). The mixture was stirred vigorously for 2 min and the slurry turned pale-yellow. A solution of [1.1.1]propellane **1** (0.27 mL, 0.20 mmol, 0.75 M in Et_2O, 1.0 equiv.) was added, and the slurry

typically changed from pale-yellow to white. The reaction mixture was stirred at -5 °C for 2 min and then sonicated (5 s, rt) to ensure complete mixing. The reaction mixture was quenched at room temperature with $Na_2S_2O_3$ (sat. aq., 0.50 mL). The biphasic mixture was poured onto H_2O (2 mL), extracted with CH_2Cl_2 (3 × 2 mL), then the combined organic phases were concentrated *in vacuo*. The residue was purified by silica plug (wash with pentane, followed by elution with EtOAc/pentane, 2:3), unless stated otherwise.

Note: Lower reaction temperatures were required if the sulfonyl iodide turned dark orange/brown before addition of ***1****. High yielding reactions typically remained pale yellow at this stage.*

General Procedure B - *Modification to Procedure A at -40 ° C, R = electron deficient and Hal = I*

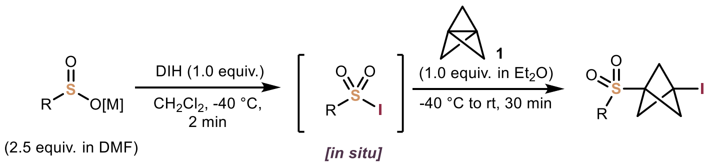

A solution of sulfinate salt (0.50 mL, 0.50 mmol, 1.0 M in DMF, 2.5 equiv.) was added dropwise to a suspension of DIH (78.4 mg, 0.20 mmol, 1.0 equiv.) in CH_2Cl_2 (0.80 mL) at -40 °C (MeCN/dry ice). The mixture was stirred vigorously for 2 min, then [1.1.1]propellane **1** (0.27 mL, 0.20 mmol, 0.75 M in Et_2O, 1.0 equiv.) was added and the mixture was stirred at this temperature for 20 min. The reaction was sonicated (5 s) then stirred at 0 °C for 10 min. The reaction was quenched at room temperature with $Na_2S_2O_3$ (sat. aq., 0.50 mL) and the biphasic mixture was poured onto H_2O (2 mL) and extracted with CH_2Cl_2 (2 × 2 mL). The combined organic phases were washed with LiCl (5% aq., 5 × 2 mL) and concentrated *in vacuo*. The residue was purified by silica plug (wash with pentane, followed by collection with EtOAc/pentane, 2:3), unless stated otherwise.

General Procedure C – *(Het)Aryl Sulfonyl BCP Bromides, R = (Het)Aryl and Hal = Br*

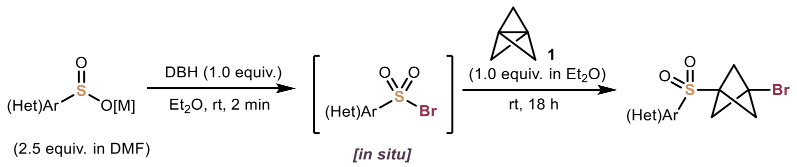

A solution of sulfinate salt (0.50 mL, 0.50 mmol, 1.0 M in H_2O, 2.5 equiv.) was added dropwise to a suspension of dibromo-5,5-dimethylhydantoin (DBH) (57.1 mg, 0.20 mmol, 1.0 equiv.) in Et_2O (0.20 mL) at room temperature (20 °C). The mixture was stirred vigorously for 2 min, then a solution of [1.1.1]propellane **1** (0.29 mL, 0.20 mmol, 0.69 M in Et_2O, 1.0 equiv.) was added. The reaction vial was capped, wrapped in parafilm and stirred at room temperature for 18 h. The sulfonyl BCP bromide typically precipitated as a white solid overnight. The reaction mixture was quenched with $Na_2S_2O_3$ (sat. aq., 0.3 mL), extracted with CH_2Cl_2 (3 × 2 mL), then the combined organic phases were concentrated *in vacuo*. The residue was purified by silica plug (wash with pentane, followed by collection with EtOAc/pentane, 2:3), unless stated otherwise.

Note: Typically, the reaction slurry remained off-white, additional white precipitates may form before addition of ***1*** *and after stirring overnight.*

General Procedure D – *Multigram, Sulfonyl BCP Bromides (RSO_2Br formed in situ with NBS)*

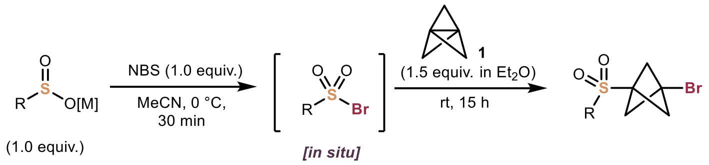

To a round-bottom flask (100 mL) was added sodium sulfinate (2.2 mmol, 1.00 equiv.), suspended in MeCN (50 mL) under Ar at 0 °C. *N*-Bromosuccinimide (NBS) (0.39 g, 2.2 mmol, 1.00 equiv.) was added in one portion 0 °C. The mixture was stirred for 30 min at 0 °C, then a solution of [1.1.1]propellane **1** (5 mL, 3.3 mmol, 0.7 M in Et_2O, 1.50 equiv.) was added. The mixture was stirred at room temperature for 15 h. The solvent was removed under reduced pressure, and the residue was purified by column chromatography.

General Procedure E – *Multigram, Sulfonyl BCP Bromides (isolated RSO_2Br)*

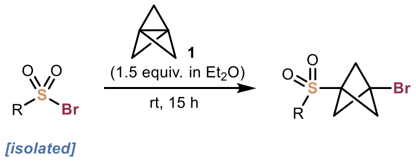

[1.1.1]Propellane **1** (700 mL, 0.47 mol, 0.7 M in Et_2O, 1.30 equiv.) was added to a Ar degassed solution of sulfonyl bromide (80.00 g, 0.36 mol, 1.00 equiv.) in Et_2O (200 mL) in one portion, at room temperature. The mixture was stirred at room temperature for 15 h. The solvent was then removed *in vacuo* (30 – 15 mmHg, 35 °C), and the residue was triturated (pentane, ca. 1000 mL), filtered and dried under reduced pressure (10 mmHg, 35 °C).

General Procedure F - *Alkyl Sulfonyl BCP Iodides, R = Alkyl and Hal = I*

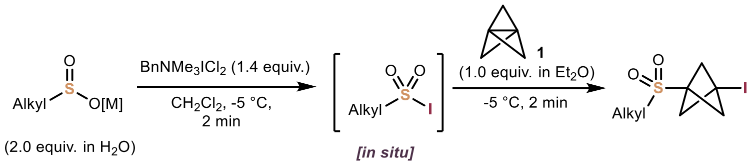

A solution of sulfinate salt (0.20 mL, 0.20 mmol, 1.0 M in H_2O, 2.0 equiv.) was added dropwise to a suspension of benzyltrimethylammonium dichloroiodate (50.5 mg, 0.14 mmol, 1.4 equiv.) in CH_2Cl_2 (0.10 mL) at -5 °C (ice/salt). The mixture was stirred vigorously for 2 min, then a solution of [1.1.1]propellane **1** (0.15 mL, 0.10 mmol, 0.70 M in Et_2O, 1.0 equiv.) was added. The reaction mixture was stirred at -5 °C for 2 min, then vial was sonicated (5 s) to ensure complete mixing. The reaction mixture was quenched at room temperature with $Na_2S_2O_3$ (sat. aq., 0.50 mL). The biphasic mixture was poured onto H_2O (1 mL), extracted with CH_2Cl_2 (3 × 2 mL), then the combined organic phases were concentrated *in vacuo*. The residue was purified by silica plug (wash with pentane, followed by collection with EtOAc/pentane, 2:3), unless stated otherwise.

General Procedure G - *Alkyl Sulfonyl BCP Bromides, R = Alkyl and Hal = Br*

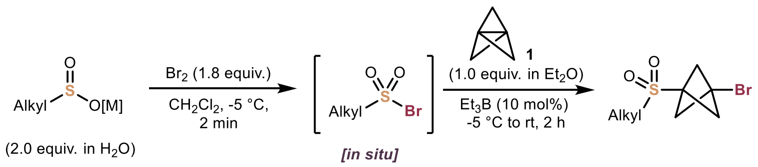

A solution of sodium sulfinate salt (0.10 mL, 0.10 mmol, 1.0 M in H_2O) was added to a 3 mL vial equipped with a stir bar and septum cooled to -5 °C. CH_2Cl_2 (0.10 mL) was added. A solution of Br_2 (0.18 mL, 0.18 mmol, 1.0 M in CH_2Cl_2) was added and the mixture was stirred for 2 min, until disappearance of the bromine colour to a colourless solution. A solution of [1.1.1]propellane **1** (0.15 mL, 0.10 mmol, 0.70 M in Et_2O) and then Et_3B (10 µL, 0.01 mmol, 1.0 M in hexanes, 10 mol%) was added. The vial stirred at -5 °C for 2 min, then vial was sonicated (5 s) to ensure complete mixing. The vial was quenched at room temperature with $Na_2S_2O_3$ (sat. aq., 0.50 mL). The biphasic mixture was poured onto H_2O (1 mL), extracted with CH_2Cl_2 (3 × 2 mL), then the combined organic phases were concentrated *in vacuo*. The residue was purified by silica plug (wash with pentane, followed by collection with EtOAc/pentane, 2:3), unless stated otherwise.

General Procedure H - *Sulfonyl Cyclobutyl Iodides, R = Aryl and Hal = I*

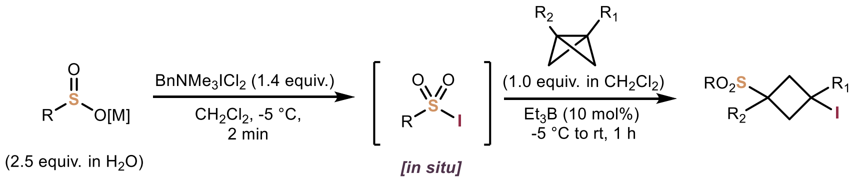

A solution of sulfinate salt (0.25 mL, 0.25 mmol, 1.0 M in H_2O, 2.5 equiv.) was added dropwise to a suspension of benzyltrimethylammonium dichloroiodate (50.5 mg, 0.14 mmol, 1.4 equiv.) in CH_2Cl_2 (0.10 mL) at -5 °C (ice/salt). The mixture was stirred vigorously for 2 min, then a solution of BCB (0.10 mL, 0.10 mmol, 1.0 M in Et_2O) and Et_3B (10 µL, 0.01 mmol, 1.0 M in hexanes, 10 mol%) were added sequentially. The reaction mixture was stirred at -5 °C for 2 min, then the vial was capped, warpped in parafilm, sonicated (5 s) and stirred at room temperature for 1 h. The reaction mixture was quenched at room temperature with $Na_2S_2O_3$ (sat. aq., 0.50 mL). The biphasic mixture was poured onto H_2O (1 mL), extracted with CH_2Cl_2 (3 × 2 mL), then the combined organic phases were concentrated *in vacuo*. The residue was purified by column chromatography.

General Procedure I - *Sulfonyl Cyclobutyl Iodides, R = Aryl and Hal = Br*

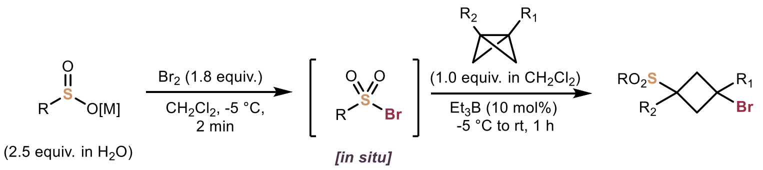

A solution of sulfinate salt (0.25 mL, 0.25 mmol, 1.0 M in H_2O, 2.5 equiv.) was added dropwise to a solution of Br_2 (0.18 mL, 0.18 mmol, 1.0 M in CH_2Cl_2) in CH_2Cl_2 (0.10 mL) at -5 °C (ice/salt). The mixture was stirred vigorously for 2 min, until disappearance of the brown bromine colour. A solution of BCB (0.10 mL, 0.10 mmol, 1.0 M in Et_2O) and Et_3B (10 µL, 0.01 mmol, 1.0 M in hexanes, 10 mol%) were added sequentially. The reaction mixture was stirred at -5 °C for 2 min, then the vial was capped, warpped in parafilm, sonicated (5 s) and stirred at room temperature for 2 h. The reaction mixture was quenched with $Na_2S_2O_3$ (sat. aq., 0.50 mL). The biphasic mixture was poured onto H_2O (1 mL), extracted with CH_2Cl_2 (3 × 2 mL), then the combined organic phases were concentrated *in vacuo*. The residue was purified by column chromatography.

1.3 General Procedures for Starting Materials (J – N)

General procedure J – *Sodium sulfinates from reduction of sulfonyl chlorides*

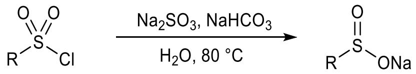

According to a modified literature procedure.[1] Sulfonyl chloride (5.00 mmol) was added to a solution of sodium sulfite (1.26 g, 10.0 mmol) and sodium bicarbonate (840 mg, 10.0 mmol) in water (5.0 mL). The suspension was then heated at 80 °C for 4 h. After cooling to room temperature, water was removed *in vacuo*. The resultant solid was stirred in EtOH (5 mL) for 30 min at 40 °C, then filtered and washed with warm EtOH (3 x 10 mL). The combined ethanol washes were concentrated *in vacuo* to yield the sulfinate salt.

General procedure K – *Sodium sulfinates from reduction of sulfonyl chlorides*

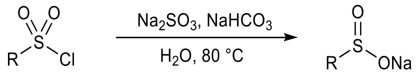

To a solution of sodium sulfite (62.00 g, 0.49 mol, 2.00 equiv.) in water (500 mL) were added sulfonyl chloride (0.245 mol, 1.00 equiv.) in one portion and sodium bicarbonate (41.00 g, 0.49 mol, 2.00 equiv.) in three portions at room temperature. The resulting mixture was heated at 80 °C for 24 h under stirring. After cooling to room temperature, the mixture was concentrated under reduced pressure (30 – 15 mmHg, 50 – 80 °C). The product was dried *in vacuo* (2.0 – 0.1 mmHg, at 80 °C) under P_2O_5 until the amount of water was less than 0.1 – 0.2 mol%. At this stage, the crude product was regularly crushed and shaken. The obtained solid was washed with dry hot ethanol (60 – 70 °C, 10 × 100 mL). The filtrate was concentrated *in vacuo* (30 – 15 mmHg, 40 – 50 °C) and dried under reduced pressure (2.0 – 0.1 mmHg, 80 °C) to give the sodium sulfinate salt.

General procedure L - *Lithium sulfinates from aryl bromides*

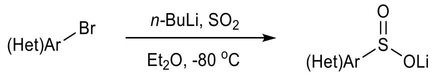

Aryl bromides (50 mmol, 1.00 equiv.) was dissolved in dry Et_2O (200 mL) and cooled to -(80 – 85) °C under Ar atmosphere. *n*-BuLi (2.5M in hexane, 0.05 mol, 20 mL, 1.00 equiv.) was added dropwise, and the solution was stirred at -80 °C for 30 min. After that, SO_2 was bubbled through the solution at -(85 – 75) °C until the release of heat stopped. The reaction mixture was allowed

to warm to room temperature and filtered. The residue was washed with Et_2O (100 mL) and dried under reduced pressure (2.0 – 0.1 mmHg, at 40 °C) to give the lithium sulfinate salt.

General procedure M – *Alkyl sulfonyl bromides from sulfonyl chlorides*

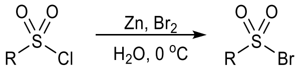

Adapted from a literature procedure.[2] To a mixture of zinc dust (85.00 g, 1.30 mol, 1.50 equiv.) in 200 mL of ice-water (100 g of ice), sulfonyl chloride (0.87 mol, 1.00 equiv.) was added within 1.5 h at 0 – 5 °C with vigorous stirring. After addition, the mixture was stirred for 30 min. After that, the reaction mixture was filtered and washed with a small amount of water (50 mL). The filtrate was transferred to the flask and cooled to 0 °C. Bromine (132.00 g, 42 mL, 0.82 mol, 0.95 equiv.) was added during 15 – 20 min at -5 – 0 °C with vigorous stirring. The reaction mixture was extracted with CH_2Cl_2 (3 × 100 mL). The combined organic layers were washed with 2M aq. $NaHSO_3$ (3 × 100 mL), ice-water (3 × 200 mL), dried over Na_2SO_4 and concentrated under reduced pressure (30 – 15 mmHg, 15 – 20 °C) to afford crude product which was used for the next step without purification.

General procedure N – *Aryl sulfonyl bromides from sulfinate salts*

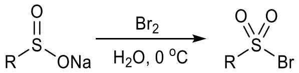

To a solution of sodium sulfinate (0.20 mol, 1.00 equiv.) in water (300 mL) was added bromine (31.00 g, 10 mL, 0.19 mol, 0.95 equiv.) within 10 min with vigorous stirring at the 0 °C. The mixture was stirred for 30 min. After that, the reaction mixture was extracted with Et_2O (3 × 300 mL). The combined organic layers were dried over Na_2SO_4 and concentrated under reduced pressure (30 – 15 mmHg, 35 °C). The final product was dried under reduced pressure (2.0 – 0.1 mmHg, 35 °C) to afford the sulfonyl bromide.

2. Experimental Data

2.1 Aryl Sulfonyl BCP Halides

1-Iodo-3-tosylbicyclo[1.1.1]pentane, 3a-I

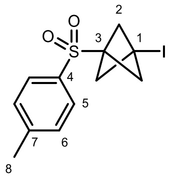

A stock solution of sulfinate salt **2a** (213 mg, 1.20 mmol) in H_2O (1.20 mL) was prepared. DIH (78.4 mg, 0.20 mmol), sulfinate salt **2a** (0.50 mL, 0.50 mmol, 1.0 M in H_2O), [1.1.1]propellane **1** (0.29 mL, 0.20 mmol, 0.69 M in Et_2O) in CH_2Cl_2 (0.80 mL) were subjected to the conditions of **general procedure A**. BCP iodide **3a-I** (68.8 mg, 0.20 mmol, 99%) was obtained as a white solid.

R_f 0.74 (EtOAc/pentane, 1:9) [UV, Vanillin].
m.p. 188° C.
IR ν_{max}/cm^{-1} (film) 1594, 1314, 1302, 1290, 1166, 1136, 859, 811, 665.
^{1}H NMR (400 MHz, $CDCl_3$) δ 7.75 – 7.68 (2H, m, H5), 7.38 – 7.34 (2H, m, H6), 2.50 (6H, s, H2), 2.46 (3H, s, H8).
^{13}C NMR (101 MHz, $CDCl_3$) δ 145.4, 133.7, 130.2, 128.7, 59.3, 57.9, 21.8, 2.6.
HRMS (ESI^+) $[M + Na]^+$ $C_{12}H_{13}O_2INaS^+$ requires 370.9573; found 370.9574.

1-Bromo-3-tosylbicyclo[1.1.1]pentane, 3a-Br

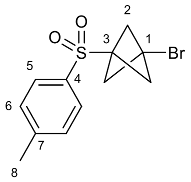

Miligram Scale: A stock solution of sulfinate salt **2a** (213 mg, 1.20 mmol) in H_2O (1.20 mL) was prepared. DBH (57.1 mg, 0.20 mmol), sulfinate salt **2a** (0.50 mL, 0.50 mmol, 1.0 M in H_2O), [1.1.1]propellane **1** (0.29 mL, 0.20 mmol, 0.69 M in Et_2O) in Et_2O (0.20 mL) were subjected to the conditions of **general procedure C**. Purification by silica plug (EtOAc/pentane, 1:4) gave the BCP bromide **3a-Br** (60.4 mg, 0.199 mmol, 99%) as a white crystalline solid.

Multigram Scale: The conditions of **general procedure E** *(isolated RSO_2Br)* gave BCP bromide **3a-Br** (1.96 g, 6.51 mmol, 96%) as a white solid.

R_f 0.38 (EtOAc/pentane, 1:9) [UV, Vanillin].

m.p. 164 – 165 °C.

IR ν_{max}/cm^{-1} (film) 2970, 1379, 1160, 1128, 951, 817.

^{1}H NMR (400 MHz, $CDCl_3$) δ 7.75 – 7.68 (2H, m, H6), 7.40 – 7.32 (2H, m, H5), 2.46 (3H, s, H8), 2.44 (6H, s, 2H).

^{13}C NMR (101 MHz, $CDCl_3$) δ 145.4, 133.9, 130.1, 128.6, 58.0, 52.6, 35.4, 21.8.

HRMS (ESI^+) $[M + Na]^+$ $C_{12}H_{13}O_2{}^{81}BrNaS^+$ requires 324.9691; found 324.9691.

1-Chloro-3-tosylbicyclo[1.1.1]pentane, 3a-Cl

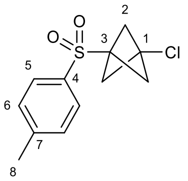

Sulfinate salt **2a** (0.50 mL, 0.50 mmol, 1.0 M in H_2O) was added to a solution of 1,3-dichloro-5,5-dimethylhydantoin (DCH) (39.4 mg, 0.20 mmol) in Et_2O (0.20 mL) at 0 °C, then stirred for 2 min. [1.1.1]propellane **1** (0.21 mL, 0.20 mmol, 0.93 M in Et_2O) and Et_3B (20 µL, 0.02 mmol, 10 mol%, 1.0 M in hexanes) were added sequentially, then the reaction was stirred at room temperature for 2 h. The reaction mixture was quenched with $Na_2S_2O_3$ (sat. aq., 0.3 mL), extracted with CH_2Cl_2 (3 × 2 mL), then the combined organic phases were concentrated *in vacuo*. Purification by column chromatography (SiO_2, EtOAc/pentane, 0:1 → 1:9) gave BCP chloride **3a-Cl** (15.9 mg, 0.31 mmol, 31%) as a white crystalline solid.

From Sulfonyl Chloride: [1.1.1]propellane **1** (0.27 mL, 0.20 mmol, 0.75 M in Et_2O) was added to a solution of 4-methylbenzenesulfonyl chloride (38.0 mg, 0.20 mmol) in MeCN (0.2 mL) in a 3 mL vial under air. The vial was capped and stirred at room temperature for 16 h. Concentration *in vacuo* and purification by column chromatography (SiO_2, EtOAc/pentane, 0:1 → 1:9) gave BCP chloride **3a-Cl** (14.6 mg, 0.22 mmol, 29%) as a white crystalline solid.

R_f 0.67 (EtOAc/pentane, 1:9).

IR ν_{max}/cm^{-1} (film) 2981, 1596, 1311, 1293, 1188, 1143, 811, 672.

^{1}**H NMR** (400 MHz, $CDCl_3$) δ 7.76 – 7.69 (2H, m, H5), 7.41 – 7.33 (2H, m, H6), 2.46 (3H, s, H8), 2.38 (6H, s, H2).

^{13}**C NMR** (101 MHz, $CDCl_3$) δ 145.4, 134.0, 130.1, 128.7, 57.2, 49.9, 48.6, 21.8.

HRMS (ESI$^+$) [M + Na]$^+$ $C_{12}H_{13}O_2{}^{35}ClNaS^+$ requires 279.0217; found 279.0218.

Characteristic ^{1}H NMR data for Staffane of 3a-Cl (separable)

^{1}**H NMR** (400 MHz, $CDCl_3$) δ 1.99 (6H, s), 1.91 (6H, s).

1-Iodo-3-(phenylsulfonyl)bicyclo[1.1.1]pentane, 3b-I

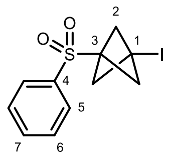

A stock solution of sulfinate salt **2b** (98.4 mg, 0.599 mmol) in H_2O (0.60 mL) was prepared. DIH (78.4 mg, 0.20 mmol), sulfinate salt **2b** (0.50 mL, 0.50 mmol, 1.0 M in H_2O), [1.1.1]propellane **1** (0.29 mL, 0.20 mmol, 0.69 M in Et_2O) in CH_2Cl_2 (0.80 mL) were subjected to the conditions of **general procedure A**. BCP iodide **3b-I** (64.4 mg, 0.193 mmol, 96%) was obtained as an off-white solid.

Note: The reaction *at 20 ˚C in Et_2O (0.20 mL) afforded BCP iodide **3b-I** in lower yield (56.6 mg, 0.169 mmol, 85%).*

Decagram Scale: Modified conditions of **general procedure E** *(isolated RSO_2I)*. [1.1.1]Propellane **1** (250 mL, 0.17 mol, 0.7 M in Et_2O, 1.30 equiv.) was added to a Ar degassed solution of benzenesulfonyl iodide (35.00 g, 0.13 mol, 1.00 equiv.) in 200 mL of Et_2O in one portion at room temperature. The mixture was stirred at room temperature for 15 h. The solvent was removed *in vacuo* (30 – 15 mmHg, 35 °C), and the residue was triturated (pentane, ca. 500 mL), filtered and dried under reduced pressure (10 mmHg, 35 °C) to provide the BCP iodide **3b-I** (39.00 g, 0.11 mol, 91%) as a yellow solid.

Alternative Procedure: Modified conditions of **general procedure D** *(in situ RSO_2I with NIS)* To a round-bottom flask (100 mL) was added sodium benzenesulfinate (0.20 g, 1.20 mmol, 1.00

equiv.), suspended in 50 mL of MeCN under Ar at 0 °C. NIS (0.28 g, 1.2 mmol, 1.00 equiv.) was added in one portion 0 °C. The mixture was stirred for further 30 min at 0 °C, then a solution of [1.1.1]propellane **1** (2.5 mL, 1.8 mmol, 0.7 M in Et_2O, 1.50 equiv.) was added. The mixture was stirred at room temperature for 15 h. The solvent was removed under reduced pressure, and the residue was purified by column chromatography (SiO_2, hexane/MTBE, 7:3) to provide BCP iodide **3b-I** (0.11 g, 0.32 mmol, 22%) as a yellow solid.

R$_f$ 0.32 (EtOAc/pentane, 1:9).
m.p. 170 – 171 °C.
IR ν_{max}/cm^{-1} (film) 2972, 1450, 1305, 1291, 1201, 1168, 1140, 864, 721, 691, 610.
^{1}H NMR (600 MHz, $CDCl_3$) δ 7.88 – 7.81 (2H, m, H5), 7.73 – 7.64 (1H, m, H7), 7.63 – 7.54 (2H, m, H6), 2.51 (6H, s, H2).
^{13}C NMR (151 MHz, $CDCl_3$) δ 136.8, 134.3, 129.5, 128.7, 59.3, 57.9, 2.4.
HRMS (ESI$^+$) [M + NH_4]$^+$ $C_{11}H_{15}INO_2S^+$ requires 351.9868; found 351.9860.

1-Bromo-3-(phenylsulfonyl)bicyclo[1.1.1]pentane, 3b-Br

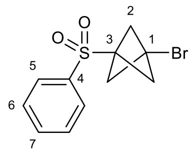

Miligram Scale: A stock solution of sulfinate salt **2b** (196.8 mg, 1.20 mmol) in H_2O (1.20 mL) was prepared. DBH (57.1 mg, 0.20 mmol), sulfinate salt **2b** (0.50 mL, 0.50 mmol, 1.0 M in H_2O), [1.1.1]propellane **1** (0.34 mL, 0.20 mmol, 0.59 M in Et_2O) in Et_2O (0.20 mL) were subjected to the conditions of **general procedure C**. BCP bromide **3b-Br** (57.0 mg, 0.199 mmol, 99%) was obtained as a white solid.

Decagram Scale: According to **general procedure E**. [1.1.1]Propellane **1** (700 mL, 0.47 mol, 0.7 M in Et_2O) was added to a Ar degassed solution of benzenesulfonyl bromide **2b1** (80.00 g, 0.36 mol) in Et_2O (200 mL) in one portion at room temperature. The mixture was stirred at room temperature for 15 h. The solvent was removed *in vacuo* (30 – 15 mmHg, 35 °C), and the residue was triturated in pentane (ca. 1000 mL), filtered and dried under reduced pressure (10 mmHg, 35 °C) to provide the sulfonyl BCP bromide **3b-Br** (96.30 g, 0.35 mol, 97%) as a white solid.

R_f 0.31 (EtOAc/pentane, 1:9).

m.p. 159 – 160 °C.

IR ν_{max}/cm^{-1} (film) 1449, 1307, 1291, 1205, 1180, 1147, 723, 616.

^{1}H NMR (600 MHz, $CDCl_3$) δ 7.88 – 7.83 (2H, m, H5), 7.72 – 7.66 (1H, m, H7), 7.61 – 7.57 (2H, m, H6), 2.46 (6H, s, H2).

^{13}C NMR (151 MHz, $CDCl_3$) δ 136.9, 134.3, 129.5, 128.7, 58.1, 52.6, 35.4.

HRMS (ESI$^+$) [M + NH_4]$^+$ $C_{11}H_{15}BrNO_2S^+$ requires 305.9986; found 305.9978.

1-Iodo-3-((4-methoxyphenyl)sulfonyl)bicyclo[1.1.1]pentane, 3c-I

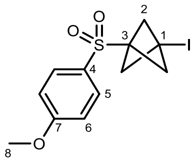

A stock solution of sulfinate salt **2c** (126 mg, 0.60 mmol, contained 8% impurity) in H_2O (0.60 mL) was prepared. DIH (78.4 mg, 0.20 mmol), sulfinate salt **2c** (0.50 mL, 0.50 mmol, 1.0 M in H_2O), [1.1.1]propellane **1** (0.29 mL, 0.20 mmol, 0.69 M in Et_2O) in CH_2Cl_2 (0.80 mL) were subjected to the conditions of **general procedure A**. BCP iodide **3c-I** (72.7 mg, 0.199 mmol, 99%) was obtained as a white solid.

R_f 0.20 (EtOAc/pentane, 1:9).

m.p. 164 – 169 °C.

IR ν_{max}/cm^{-1} (film) 2981, 1593, 1492 1258, 1163, 1132, 803, 769, 669.

^{1}H NMR (400 MHz, $CDCl_3$) δ 7.80 – 7.72 (2H, m, H5), 7.06 – 6.98 (2H, d, J = 8.9 Hz, H6), 3.89 (3H, s, H8), 2.50 (6H, s, H2).

^{13}C NMR (101 MHz, $CDCl_3$) δ 164.2, 130.8, 128.1, 114.7, 59.3, 58.1, 55.9, 2.7.

HRMS (ESI$^+$) [M + Na]$^+$ $C_{12}H_{13}O_3INaS^+$ requires 386.9522; found 386.9521.

1-Bromo-3-((4-methoxyphenyl)sulfonyl)bicyclo[1.1.1]pentane, 3c-Br

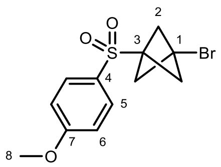

Miligram Scale: A stock solution of sulfinate salt **2c** (126 mg, 0.60 mmol, contained 8% impurity) in H_2O (0.60 mL) was prepared. DBH (57.1 mg, 0.20 mmol), sulfinate salt **2c** (0.50 mL, 0.50 mmol, 1.0 M in H_2O), [1.1.1]propellane **1** (0.29 mL, 0.20 mmol, 0.69 M in Et_2O) in Et_2O (0.20 mL) were subjected to the conditions of **general procedure C**. BCP bromide **3c-Br** (63.0 mg, 0.199 mmol, 99%) was obtained as a white solid.

Multigram Scale: The conditions of **general procedure E** *(isolated RSO_2Br)* gave BCP bromide **3c-Br** (1.19 g, 3.75 mmol, 97%) as a beige solid.

Alternative Procedure: The conditions of **general procedure D** *(in situ RSO_2Br with NBS)* and purification by column chromatography (SiO_2, hexane/MTBE, 7:3) gave BCP bromide **3c-Br** (0.70 g, 2.21 mmol, 43%) as a beige solid.

R_f 0.20 (EtOAc/pentane, 1:9).

m.p. 171 – 172 °C.

IR ν_{max}/cm^{-1} (film) 1595, 1495, 1307, 1259, 1177, 1138, 672.

^{1}H NMR (600 MHz, $CDCl_3$) δ 7.83 – 7.75 (2H, m, H5), 7.09 – 7.01 (2H, m, H6), 3.90 (3H, s, H8), 2.45 (6H, s, H2).

^{1}H NMR (500 MHz, DMSO-d_6) δ 7.74 (2H, d, *J* = 8.8 Hz, H5), 7.19 (2H, d, *J* = 8.8 Hz, H6), 3.87 (3H, s, H8), 2.47 (6H, s, H2).

^{13}C NMR (151 MHz, $CDCl_3$) δ 164.3, 130.8, 128.3, 114.7, 58.0, 55.9, 52.8, 35.5.

^{13}C NMR (151 MHz, DMSO-d_6) δ 163.7, 130.4, 127.7, 114.9, 57.5, 55.8, 52.1, 36.2.

HRMS (ESI$^+$) [M + H]$^+$ $C_{12}H_{14}O_3{}^{79}BrS^+$ requires 316.9842; found 316.9841.

1-((4-Bromophenyl)sulfonyl)–3-iodobicyclo[1.1.1]pentane, 3d-I

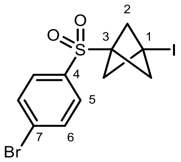

A stock solution of sulfinate salt **2d** (290 mg, 1.20 mmol) in H_2O (1.20 mL) was prepared, a drop of DMF was added to aid solubility of the sulfinate salt. DIH (78.4 mg, 0.20 mmol), sulfinate salt **2d** (0.50 mL, 0.50 mmol, 1.0 M in H_2O), [1.1.1]propellane **1** (0.29 mL, 0.20 mmol, 0.69 M in Et_2O) in CH_2Cl_2 (0.80 mL) were subjected to the conditions of **general procedure A**. BCP iodide **3d-I** (81.4 mg, 1.97 mmol, 99%) was obtained as a white solid.

R$_f$ 0.32 (EtOAc/pentane, 1:17).
m.p. 230 ° C.
IR ν_{max}/cm^{-1} (film) 1574, 1311, 1278, 1202, 1168, 1136, 777.
^{1}H NMR (600 MHz, $CDCl_3$) δ 7.74 – 7.71 (2H, m, H5), 7.71 – 7.68 (2H, m, H6), 2.51 (6H, s, H2).
^{13}C NMR (151 MHz, $CDCl_3$) δ 135.8, 132.9, 130.2, 129.9, 59.3, 57.9, 2.0.
HRMS (ESI$^{+/-}$) Not Found.

1-Bromo-3-((4-bromophenyl)sulfonyl)bicyclo[1.1.1]pentane, 3d-Br

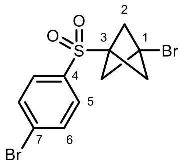

A stock solution of sulfinate salt **2d** (145 mg, 0.597 mmol) in H_2O (0.60 mL) was prepared, a drop of DMF was added to aid solubility of the sulfinate salt. DBH (57.1 mg, 0.20 mmol), sulfinate salt **2d** (0.50 mL, 0.50 mmol, 1.0 M in H_2O), [1.1.1]propellane **1** (0.29 mL, 0.20 mmol, 0.69 M in Et_2O) in Et_2O (0.20 mL) were subjected to the conditions of **general procedure C**. BCP iodide **3d-Br** (69.4 mg, 0.190 mmol, 95%) was obtained as a pale-yellow solid.

R$_f$ 0.40 (EtOAc/pentane, 1:9).
m.p. 222 ° C.
IR ν_{max}/cm^{-1} (film) 1574, 1387, 1312, 1180, 1141, 755, 644.
^{1}H NMR (400 MHz, $CDCl_3$) δ 7.77 – 7.67 (4H, m, H5, H6), 2.47 (6H, s, H2).

^{13}**C NMR** (101 MHz, $CDCl_3$) δ 135.9, 132.9, 130.1, 129.9, 58.0, 52.6, 35.2.
HRMS (ESI$^{+/-}$) Not Found.

1-((4-Fluorophenyl)sulfonyl)-3-iodobicyclo[1.1.1]pentane, 3e-I

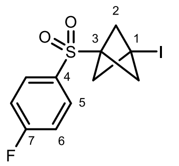

A stock solution of sulfinate salt **2e** (109 mg, 0.598 mmol) in DMF (0.60 mL) was prepared. DIH (78.4 mg, 0.20 mmol), sulfinate salt **2e** (0.50 mL, 0.50 mmol, 1.0 M in DMF), [1.1.1]propellane **1** (0.29 mL, 0.20 mmol, 0.69 M in Et_2O) in CH_2Cl_2 (0.80 mL) were subjected to the conditions of **general procedure B** (-40 °C). BCP iodide **3e-I** (35.4 mg, 0.101 mmol, 50%) was obtained as a white solid.

R$_f$ 0.25 (EtOAc/pentane, 1:19).
m.p. 168 – 170 °C.
IR ν_{max}/cm^{-1} (film) 1593, 1314, 1293, 1170, 1137, 820.
^{1}**H NMR** (600 MHz, $CDCl_3$) δ 7.89 – 7.83 (2H, m, H5), 7.29 – 7.23 (2H, m, H6), 2.51 (6H, s, H2).
^{13}**C NMR** (151 MHz, $CDCl_3$) δ 166.3 (d, $^1J_{CF}$ = 257.3 Hz), 132.8 (d, $^4J_{CF}$ = 3.3 Hz), 131.6 (d, $^3J_{CF}$ = 9.7 Hz), 117.0 (d, $^2J_{CF}$ = 22.5 Hz), 59.3, 58.0, 2.1.
^{19}**F NMR** (377 MHz, $CDCl_3$) δ -102.4
HRMS (ESI$^{+/-}$) Not found.

1-Bromo-3-((4-fluorophenyl)sulfonyl)bicyclo[1.1.1]pentane, 3e-Br

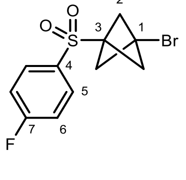

Miligram Scale: A stock solution of sulfinate salt **2e** (109 mg, 0.60 mmol) in H_2O (0.60 mL) was prepared. DBH (57.1 mg, 0.20 mmol), sulfinate salt **2e** (0.50 mL, 0.50 mmol, 1.0 M in H_2O), [1.1.1]propellane **1** (0.29 mL, 0.20 mmol, 0.69 M in Et_2O) in Et_2O (0.20 mL) were subjected to the conditions of **general procedure C**. BCP bromide **3e-Br** (61.0 mg, 0.20 mmol, 99%) was obtained as a white solid.

Gram Scale: The conditions of **general procedure E** *(isolated RSO_2Br)* gave BCP bromide **3e-Br** (33.00 g, 0.13 mol, 98%) as a white solid.

Alternative Procedure: The conditions of **general procedure D** *(in situ RSO_2Br with NBS)* and purification by column chromatography (SiO_2, hexane/MTBE, 7:3) gave BCP bromide **3e-Br** (0.43 g, 1.4 mmol, 62%) as a white solid.

R_f 0.45 (EtOAc/pentane, 1:9).
m.p. 163 – 164 °C.
IR ν_{max}/cm^{-1} (film) 2981, 1590, 1493, 1382, 1310, 1292, 1236, 1178, 1144, 1138, 870, 672.
^{1}H NMR (600 MHz, $CDCl_3$) δ 7.90 – 7.84 (2H, m, H5), 7.31 – 7.23 (2H, m, H6), 2.46 (6H, s, H2).
^{13}C NMR (151 MHz, $CDCl_3$) δ 166.3 (d, $^1J_{CF}$ = 257.7 Hz), 133.0 (d, $^3J_{CF}$ = 3.2 Hz), 131.5 (d, $^2J_{CF}$ = 9.6 Hz), 117.0 (d, $^2J_{CF}$ = 22.8 Hz), 58.0, 52.7, 35.2.
^{19}F NMR (377 MHz, $CDCl_3$) δ -102.3.
HRMS (ESI^+) $[M + H]^+$ $C_{11}H_{11}{}^{81}BrFO_2S^+$ requires 306.9627; found 306.9624.

1-Bromo-3-((3-fluorophenyl)sulfonyl)bicyclo[1.1.1]pentane, 3f-Br

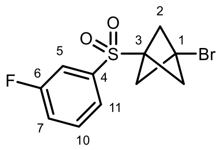

Miligram Scale: The conditions of **general procedure D** *(in situ RSO_2Br with NBS)* and purification by column chromatography (SiO_2, hexane/MTBE, 7:3) gave BCP bromide **3f-Br** (0.54 g, 1.77 mmol, 58%) as a white solid.

Gram Scale: The conditions of **general procedure E** *(isolated RSO_2Br)* gave BCP bromide **3f-Br** (1.22 g, 4.00 mmol, 96%) as a white solid.

R_f 0.67 (hexane/EtOAc, 7:3).
m.p. 146 – 147 °C.
^{1}H NMR (500 MHz, $CDCl_3$) δ 7.65 (1H, d, J = 7.7 Hz, H5), 7.62 – 7.53 (2H, m, H7, H11), 7.40 (1H, td, J = 8.2, 2.4 Hz, H10), 2.48 (6H, s, H2).

^{13}C NMR (126 MHz, $CDCl_3$) δ 162.7 (d, $^1J_{CF}$ = 253.4 Hz), 138.9 (d, $^3J_{CF}$ = 6.5 Hz), 131.5 (d, $^3J_{CF}$ = 7.6 Hz), 124.5 (d, $^4J_{CF}$ = 3.3 Hz), 121.7 (d, $^2J_{CF}$ = 21.2 Hz), 116.0 (d, $^2J_{CF}$ = 24.2 Hz), 58.1, 52.5, 35.1.

^{19}F {^{1}H} NMR (376 MHz, $CDCl_3$) δ -109.0 (s).

HRMS (ESI$^+$) [M + NH_4]$^+$ $C_{11}H_{14}{}^{81}BrFNO_2S^+$ requires 323.9892; found 323.9885.

4-((3-Bromobicyclo[1.1.1]pentan-1-yl)sulfonyl)benzonitrile, 3g-I

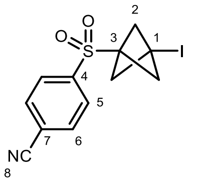

A stock solution of sulfinate salt **2g** (113mg, 0.597 mmol) in H_2O (0.60 mL) was prepared. DIH (78.4 mg, 0.20 mmol), sulfinate salt **2g** (0.50 mL, 0.50 mmol, 1.0 M in H_2O), [1.1.1]propellane **1** (0.34 mL, 0.20 mmol, 0.59 M in Et_2O) in CH_2Cl_2 (0.80 mL) were subjected to the conditions of **general procedure A**. BCP iodide **3g-I** (40.6 mg, 0.113 mmol, 57%) was obtained as a white solid.

R_f 0.17 (EtOAc/pentane, 1:17).

m.p. 234 ° C.

IR ν_{max}/cm^{-1} (film) 1311, 1293, 1201, 1168, 1135, 844.

^{1}H NMR (600 MHz, $CDCl_3$) δ 8.02 – 7.94 (2H, m, H5), 7.93 – 7.85 (2H, m, H6), 2.53 (6H, s, H2).

^{13}C NMR (151 MHz, $CDCl_3$) δ 141.1, 133.3, 129.4, 118.2, 117.0, 59.3, 57.7, 1.4.

HRMS (ESI$^{+/-}$) Not found.

4-((3-Bromobicyclo[1.1.1]pentan-1-yl)sulfonyl)benzonitrile, 3g-Br

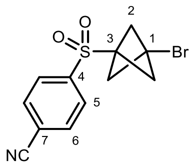

A stock solution of sulfinate salt **2g** (113 mg, 0.60 mmol) in H_2O (0.60 mL) was prepared. DBH (57.1 mg, 0.20 mmol), sulfinate salt **2g** (0.50 mL, 0.50 mmol, 1.0 M in H_2O), [1.1.1]propellane **1** (0.29 mL, 0.20 mmol, 0.69 M in Et_2O) in Et_2O (0.20 mL) were subjected to the conditions of **general procedure C**. BCP bromide **3g-Br** (38.5 mg, 0.123 mmol, 62%) was obtained as a white solid.

R_f 0.10 (EtOAc/pentane, 1:17).

IR ν_{max}/cm^{-1} (film) 2981, 1583, 1465, 1311, 1275, 1204, 1184, 1141, 881.

^{1}H NMR (600 MHz, $CDCl_3$) δ 8.01 – 7.97 (2H, m, H5), 7.92 – 7.87 (2H, m, H6), 2.48 (6H, s, H2).

^{13}C NMR (151 MHz, $CDCl_3$) δ 141.2, 133.3, 129.4, 118.3, 117.0, 58.1, 52.5, 34.9.

HRMS (ESI$^+$) Not found.

1-Iodo-3-((4-nitrophenyl)sulfonyl)bicyclo[1.1.1]pentane, 3h-I

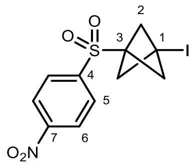

A stock solution of sulfinate salt **2h** (140 mg, 0.60 mmol) in DMF (0.60 mL) was prepared. DIH (78.4 mg, 0.20 mmol), sulfinate salt **2h** (0.50 mL, 0.50 mmol, 1.0 M in DMF), [1.1.1]propellane **1** (0.29 mL, 0.20 mmol, 0.69 M in Et_2O) in CH_2Cl_2 (0.80 mL) were subjected to the conditions of **general procedure B** (-40 °C). Purification by column chromatography (SiO_2, EtOAc/pentane, 0:1 → 1:9) gave BCP iodide **3h-I** (40.8 mg, 0.108 mmol, 54%) as an off-white solid.

R_f 0.57 (EtOAc/pentane, 1:9).

m.p. 240 °C.

IR ν_{max}/cm^{-1} (film) 1385, 1166, 1151, 669.

^{1}H NMR (600 MHz, $CDCl_3$) δ 8.44 – 8.41 (2H, m, H6), 8.07 – 8.04 (2H, m, H5), 2.54 (6H, s, H2).

^{13}C NMR (151 MHz, $CDCl_3$) δ 151.3, 142.6, 130.2, 124.7, 59.3, 57.7, 1.3.

HRMS (ESI$^{+/-}$, EI) Not found.

1-Bromo-3-((4-nitrophenyl)sulfonyl)bicyclo[1.1.1]pentane, 3h-Br

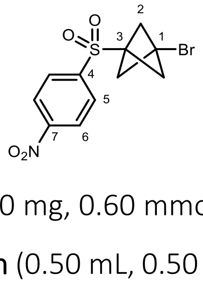

A stock solution of sulfinate salt **2h** (140 mg, 0.60 mmol) in H_2O (0.60 mL) was prepared. DBH (57.1 mg, 0.20 mmol), sulfinate salt **2h** (0.50 mL, 0.50 mmol, 1.0 M in H_2O), [1.1.1]propellane **1** (0.29 mL, 0.20 mmol, 0.69 M in Et_2O) in Et_2O (0.20 mL) were subjected to the conditions of

general procedure C. BCP bromide **3h-Br** (59.3 mg, 0.179 mmol, 90%) was obtained as a white solid.

R$_f$ 0.57 (EtOAc/pentane, 1:9).
m.p. 221 ° C.
IR ν_{max}/cm^{-1} (film) 1759, 1538, 1352, 1308, 1205, 1181, 1143, 736, 669.
^{1}**H NMR** (600 MHz, $CDCl_3$) δ 8.48 – 8.40 (2H, m, H6), 8.11 – 8.03 (2H, m, H5), 2.50 (6H, s, H2).
^{13}C NMR (151 MHz, $CDCl_3$) δ 151.3, 142.6, 130.2, 124.7, 58.1, 52.6, 34.9.
HRMS (ESI$^{+/-}$, APCI, EI) Not Found.

1-Bromo-3-((4-(trifluoromethyl)phenyl)sulfonyl)bicyclo[1.1.1]pentane, 3i-Br

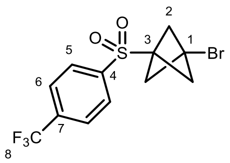

Miligram Scale: The conditions of **general procedure D** *(in situ RSO$_2$Br with NBS)* and purification by column chromatography (SiO_2, hexane/EtOAc, 9:1) gave BCP bromide **3i-Br** (0.41 g, 1.15 mmol, 61%) as a pink solid.

Gram Scale: The conditions of **general procedure E** *(isolated RSO$_2$Br)* gave BCP bromide **3i-Br** (1.16 g, 3.27 mmol, 95%) as a pink solid.

R$_f$ 0.76 (hexane/EtOAc, 7:3).
m.p. 139 – 140 °C.
^{1}**H NMR** (500 MHz, $CDCl_3$) δ 8.00 (2H, d, J = 8.2 Hz, H5), 7.86 (2H, d, J = 8.2 Hz, H6), 2.48 (6H, s, H2).
^{13}**C NMR** (151 MHz, $CDCl_3$) δ 140.5, 136.1 (q, $^2J_{CF3}$ = 33.2 Hz), 129.3, 126.7 (q, $^3J_{CF3}$ = 3.5 Hz), 123.1 (q, $^1J_{CF3}$ = 273.2 Hz), 58.1, 52.6, 35.0.
^{19}**F {^{1}H} NMR** (376 MHz, $CDCl_3$) δ -63.7 (s).
HRMS (ESI$^+$) [M + H]$^+$ $C_{12}H_{11}{}^{81}BrF_3O_2S^+$ requires 356.9595; found 356.9593.

Methyl 4-((3-iodobicyclo[1.1.1]pentan-1-yl)sulfonyl)benzoate, 3j-I

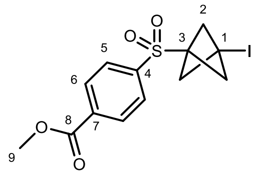

A stock solution of sulfinate salt **2j** (0.60 mmol) in DMF (0.60 mL) was prepared. DIH (78.4 mg, 0.20 mmol), sulfinate salt **2j** (0.50 mL, 0.50 mmol, 1.0 M in DMF), [1.1.1]propellane **1** (0.29 mL, 0.20 mmol, 0.69 M in Et_2O) in CH_2Cl_2 (0.80 mL) were subjected to the conditions of **general procedure B** (-40 °C). Purification by column chromatography (SiO_2, EtOAc/pentane, 1:9 → 3:7), then trituration (Et_2O) gave BCP iodide **3j-I** (9.0 mg, 0.024 mmol, 12%) as a white solid.

R_f 0.30 (EtOAc/pentane, 1:4).
IR ν_{max}/cm^{-1} (film) 1723, 1170, 1150.
^{1}H NMR (600 MHz, $CDCl_3$) δ 8.23 (2H, d, *J* = 8.1 Hz, H5), 7.92 (2H, d, *J* = 8.5 Hz, H6), 3.98 (3H, s, H9), 2.52 (6H, s, H2).
^{13}C NMR (151 MHz, $CDCl_3$) δ 165.5, 140.7, 135.5, 130.6, 128.8, 59.3, 57.8, 53.0, 1.9.
HRMS (ESI$^{+/-}$) $[M + Na]^+$ $C_{13}H_{13}IO_4SNa^+$ calculated 414.9472, found 414.9471.

Methyl 4-((3-bromobicyclo[1.1.1]pentan-1-yl)sulfonyl)benzoate, 3j-Br

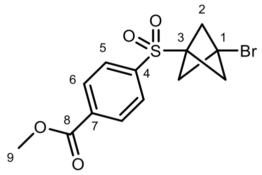

A stock solution of sulfinate salt **2j** (0.60 mmol) in H_2O (0.60 mL) was prepared. DBH (57.1 mg, 0.20 mmol), sulfinate salt **2j** (0.50 mL, 0.50 mmol, 1.0 M in H_2O), [1.1.1]propellane **1** (0.29 mL, 0.20 mmol, 0.69 M in Et_2O) in Et_2O (0.20 mL) were subjected to the conditions of **general procedure C**. Purification by column chromatography (SiO_2, EtOAc/pentane, 1:9 → 3:7) gave BCP bromide **3j-Br** (60 mg, 0.17 mmol, 87%) as a white solid.

R_f 0.28 (EtOAc / pentane, 1:4)
IR ν_{max}/cm^{-1} (film) 1730, 1277, 1181.
^{1}H NMR (600 MHz, $CDCl_3$) δ 8.23 (2H, d, *J* = 8.7 Hz, H5), 7.93 (2H, d, *J* = 8.7 Hz, H6), 3.98 (3H, s, H9), 2.47 (6H, s, H2).

^{13}C NMR (151 MHz, $CDCl_3$) δ 165.48, 140.7, 135.5, 130.6, 128.8, 58.1, 53.0, 52.6, 35.1.
HRMS (ESI$^{+/-}$) $[M + Na]^+$ $C_{13}H_{13}IO_4SNa^+$ calculated 414.9472, found 414.9471.

1-Iodo-3-(o-tolylsulfonyl)bicyclo[1.1.1]pentane, 3k-I

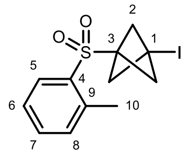

A stock solution of sulfinate salt **2k** (107 mg, 0.601 mmol) in H_2O (0.60 mL) was prepared. DIH (78.4 mg, 0.20 mmol), sulfinate salt **2k** (0.50 mL, 0.50 mmol, 1.0 M in H_2O), [1.1.1]propellane **1** (0.29 mL, 0.20 mmol, 0.69 M in Et_2O) in CH_2Cl_2 (0.80 mL) were subjected to the conditions of **general procedure A**. BCP iodide **3k-I** (61.0 mg, 0.175 mmol, 88%) was obtained as a yellow solid.

R$_f$ 0.53 (EtOAc/pentane, 1:9) [UV].
m.p. 130 °C.
IR ν_{max}/cm^{-1} (film) 1303, 1292, 1203, 1162, 1139, 1122, 856, 689, 615.
^{1}H NMR (400 MHz, $CDCl_3$) δ 7.91 (1H, dd, *J* = 7.9, 1.4 Hz, H5), 7.53 (1H, app. td, *J* = 7.5, 1.5 Hz, H7), 7.38 (1H, app. ddt, *J* = 7.9, 1.2, 0.6 Hz, H6), 7.36 – 7.32 (1H, m, H8), 2.64 (3H, s, H10), 2.54 (6H, s, H2).
^{13}C NMR (101 MHz, $CDCl_3$) δ 139.2, 135.0, 134.3, 133.0, 131.1, 127.0, 59.5, 58.3, 21.0, 2.3.
HRMS (ESI$^+$) $[M + H]^+$ $C_{12}H_{14}O_2IS^+$ requires 348.9754; found 348.9754.

1-Bromo-3-(o-tolylsulfonyl)bicyclo[1.1.1]pentane, 3k-Br

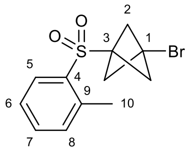

A stock solution of sulfinate salt **2k** (126 mg, 0.707 mmol) in H_2O (0.60 mL) was prepared. DBH (57.1 mg, 0.20 mmol), sulfinate salt **2k** (0.50 mL, 0.50 mmol, 1.0 M in H_2O), [1.1.1]propellane **1** (0.29 mL, 0.20 mmol, 0.69 M in Et_2O) in Et_2O (0.20 mL) were subjected to the conditions of **general procedure C**. Purification by column chromatography (SiO_2, EtOAc/pentane, 1:9 → 1:4) gave BCP bromide **3k-Br** (19.6 mg, 0.0651 mmol, 33%) as a white solid.

R_f 0.29 (EtOAc/pentane, 1:4) [UV].

m.p. 105 ° C.

IR ν_{max}/cm^{-1} (film) 1473, 1305, 1181, 1168, 1147, 652.

^{1}H NMR (600 MHz, $CDCl_3$) δ 7.92 (1H, dd, *J* = 7.9, 1.4 Hz, H5), 7.54 (1H, td, *J* = 7.5, 1.4 Hz, H7), 7.38 (1H, ddq, *J* = 8.0, 1.2, 0.6 Hz, H8), 7.34 (1H, ddt, *J* = 7.6, 1.4, 0.6 Hz, H6), 2.65 (3H, s, H10), 2.49 (6H, s, H2)

^{13}C NMR (151 MHz, $CDCl_3$) δ 139.2, 135.1, 134.3, 133.0, 131.1, 127.0, 77.2, 58.2, 53.0, 35.1, 21.0.

HRMS (ESI$^{+/-}$, EI) Not found.

1-Iodo-3-(mesitylsulfonyl)bicyclo[1.1.1]pentane, 3l-I

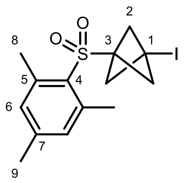

A stock solution of sulfinate salt **2l** (124 mg, 0.601 mmol) in DMF (0.60 mL) was prepared. DIH (78.4 mg, 0.20 mmol), sulfinate salt **2l** (0.50 mL, 0.50 mmol, 1.0 M in DMF), [1.1.1]propellane **1** (0.29 mL, 0.20 mmol, 0.69 M in Et_2O) in CH_2Cl_2 (0.80 mL) were subjected to the conditions of **general procedure B** (-40 °C). BCP iodide **3l-I** (49.7 mg, 0.132 mmol, 66%) was obtained as an off-white solid.

R_f 0.35 (EtOAc/pentane, 1:19).

m.p. 180 – 182 °C.

IR ν_{max}/cm^{-1} (film) 2976, 1602, 1310, 1193, 1165, 1132, 861, 658.

^{1}H NMR (400 MHz, $CDCl_3$) δ 6.96 (2H, hept., *J* = 0.7 Hz, H6), 2.59 – 2.58 (6H, m, H8), 2.57 (6H, s, H2), 2.31 – 2.30 (3H, m, H9).

^{13}C NMR (101 MHz, $CDCl_3$) δ 144.0, 141.0, 132.4, 130.7, 59.4, 58.6, 23.4, 21.2, 2.5.

HRMS (ESI$^+$) $[M + H]^+$ $C_{14}H_{18}O_2IS^+$ requires 377.0067; found 377.0067.

1-Bromo-3-(mesitylsulfonyl)bicyclo[1.1.1]pentane, 3l-Br

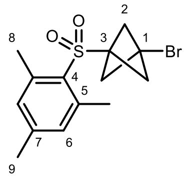

A stock solution of sulfinate salt **2l** (124 mg, 0.601 mmol) in H_2O (0.60 mL) was prepared. DBH (57.1 mg, 0.20 mmol), sulfinate salt **2l** (0.50 mL, 0.50 mmol, 1.0 M in H_2O), [1.1.1]propellane **1** (0.34 mL, 0.20 mmol, 0.59 M in Et_2O) in Et_2O (0.20 mL) were subjected to the conditions of **general procedure C**. BCP bromide **3l-Br** (53.6 mg, 0.163 mmol, 82%) was obtained as a white solid.

R$_f$ 0.35 (EtOAc/pentane, 1:19).
m.p. 82 °C.
IR ν_{max}/cm^{-1} (film) 1357, 1311, 1167, 1139, 660.
^{1}**H NMR** (400 MHz, $CDCl_3$) δ 6.99 – 6.96 (2H, m, H6), 2.59 (6H, s, H8), 2.51 (6H, s, H2), 2.31 (3H, s, H9).
^{13}**C NMR** (101 MHz, $CDCl_3$) δ 144.0, 141.0, 132.5, 130.8, 58.0, 53.2, 35.1, 23.4, 21.2.
HRMS (ESI$^+$) $[M + Na]^+$ $C_{14}H_{17}O_2{}^{81}BrNaS^+$ requires 353.0004; found 353.0005.

1-((3,5-Difluorophenyl)sulfonyl)-3-iodobicyclo[1.1.1]pentane, 3m-I

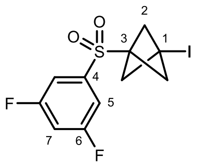

A stock solution of sulfinate salt **2m** (120 mg, 0.600 mmol) in DMF (0.60 mL) was prepared. DIH (78.4 mg, 0.20 mmol), sulfinate salt **2m** (0.50 mL, 0.50 mmol, 1.0 M in DMF), [1.1.1]propellane **1** (0.29 mL, 0.20 mmol, 0.69 M in Et_2O) in CH_2Cl_2 (0.80 mL) were subjected to the conditions of **general procedure B** (-40 °C). Purification by column chromatography (SiO_2, Et_2O/pentane, 2:98) gave BCP iodide **3m-I** (51.2 mg, 0.138 mmol, 69%) as a white solid.

R$_f$ 0.45 (Et_2O/pentane, 2:98).
m.p. 180 ° C.
IR ν_{max}/cm^{-1} (film) 1592, 1534, 1403, 1371, 1362, 1191, 1171, 989, 781.

^{1}**H NMR** (600 MHz, $CDCl_3$) δ 7.42 – 7.37 (2H, m, H5), 7.14 (1H, tt, J = 8.4, 2.4 Hz, H7), 2.55 (6H, s, H2).

^{13}**C NMR** (151 MHz, $CDCl_3$) δ 163.1 (dd, $^{1,3}J_{CF}$ = 256.7, 11.4 Hz), 140.2 (t, $^{3}J_{CF}$ = 7.9 Hz), 112.3 (dd, $^{2,4}J_{CF}$ = 21.5, 6.5 Hz), 110.1 (t, $^{2}J_{CF}$ = 25.0 Hz), 59.3, 57.6, 1.4.

^{19}**F NMR** (377 MHz, $CDCl_3$) δ -104.3.

HRMS (ESI$^{+/-}$ / APCI) Not found.

1-Bromo-3-((3,5-difluorophenyl)sulfonyl)bicyclo[1.1.1]pentane, 3m-Br

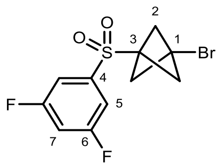

A stock solution of sulfinate salt **2m** (120 mg, 0.600 mmol) in H_2O (0.60 mL) was prepared. DBH (57.1 mg, 0.20 mmol), sulfinate salt **2m** (0.50 mL, 0.50 mmol, 1.0 M in H_2O), [1.1.1]propellane **1** (0.29 mL, 0.20 mmol, 0.69 M in Et_2O) in Et_2O (0.20 mL) were subjected to the conditions of **general procedure C**. BCP bromide **3m-Br** (64.0 mg, 0.199 mmol, 99%) was obtained as a white solid.

R_f 0.73 (EtOAc/pentane, 1:9).

m.p. 163 °C.

IR ν_{max}/cm^{-1} (film) 1604, 1441, 1318, 1305, 1201, 1182, 1128, 986, 870, 676.

^{1}**H NMR** (600 MHz, $CDCl_3$) δ 7.45 – 7.35 (2H, m, H5), 7.15 (1H, tt, J = 8.4, 2.3 Hz, H7), 2.50 (6H, s, H2).

^{13}**C NMR** (151 MHz, $CDCl_3$) δ 163.1 (dd, $^{1,3}J_F$ = 256.7, 11.4 Hz), 140.3 (t, $^{3}J_F$ = 8.0 Hz), 112.3 (dd, $^{2,4}J_F$ = 21.8, 6.5 Hz), 110.1 (t, $^{2}J_F$ = 24.9 Hz), 58.1, 52.4, 34.9.

^{19}**F NMR** (376 MHz, $CDCl_3$) δ -104.3.

HRMS (ESI^{+} / APCI) Not found.

1-((3,5-Bis(trifluoromethyl)phenyl)sulfonyl)-3-iodobicyclo[1.1.1]pentane, 3n-I

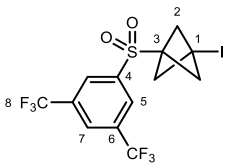

A stock solution of sulfinate salt **2n** (205 mg, 0.60 mmol, contained 14% impurity) in DMF (0.60 mL) was prepared. DIH (78.4 mg, 0.20 mmol), sulfinate salt **2n** (0.50 mL, 0.50 mmol, 1.0 M in DMF), [1.1.1]propellane **1** (0.29 mL, 0.20 mmol, 0.69 M in Et_2O) in CH_2Cl_2 (0.80 mL) were subjected to the conditions of **general procedure B** (-41 °C). Purification by column chromatography (SiO_2, pentane) gave BCP iodide **3n-I** (64.5 mg, 0.137 mmol, 69%) as an off-white solid.

R_f 0.75 (pentane).

m.p. 125 – 130° C.

IR ν_{max}/cm^{-1} (film) 2981, 1362, 1334, 1280, 1174, 1136, 1105, 647.

^{1}H NMR (600 MHz, $CDCl_3$) δ 8.30 (2H, app. dp, *J* = 1.7, 0.6 Hz, H5), 8.19 (1H, app. tp, *J* = 1.4, 0.7 Hz, H7), 2.56 (6H, s, H2).

^{13}C NMR (151 MHz, $CDCl_3$) δ 139.9, 133.7 (q, $^2J_{CF3}$ = 34.9 Hz), 129.0 (q, $^3J_{CF3}$ = 3.7 Hz), 128.0 (p, $^3J_{CF3}$ = 3.7 Hz), 122.4 (q, $^1J_{CF3}$ = 273.6 Hz), 59.2, 57.7, 1.0.

^{19}F NMR (377 MHz, $CDCl_3$) δ -62.9.

HRMS (ESI$^{+/-}$) Not found.

1-((3,5-Difluorophenyl)sulfonyl)-3-iodobicyclo[1.1.1]pentane, 3n-Br

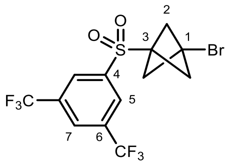

A stock solution of sulfinate salt **2n** (205 mg, 0.60 mmol, contained 14% impurity) in H_2O (0.60 mL) was prepared. DBH (57.1 mg, 0.20 mmol), sulfinate salt **2n** (0.50 mL, 0.50 mmol, 1.0 M in H_2O), [1.1.1]propellane **1** (0.29 mL, 0.20 mmol, 0.69 M in Et_2O) in Et_2O (0.20 mL) were subjected to the conditions of **general procedure C**. BCP bromide **3n-Br** (77.8 mg, 0.184 mmol, 92%) was obtained as a white solid.

R_f 0.93 (EtOAc/pentane, 1:9).

m.p. 119 – 210 °C.

IR v_{max}/cm^{-1} (film) 2981, 1279, 1267, 1282, 1138, 1107.

^{1}H NMR (400 MHz, $CDCl_3$) δ 8.31–8.30 (2H, m, H5), 8.20 (1H, tt, *J* = 1.5, 0.8 Hz, H7), 2.52 (6H, s, H2).

^{13}C NMR (151 MHz, $CDCl_3$) δ 140.0, 133.7 (q, $^2J_{CF3}$ = 34.9 Hz), 129.6–128.7 (m), 128.0 (p, $^3J_{CF3}$ = 3.6 Hz), 122.4 (q, $^1J_{CF3}$ = 273.6 Hz), 58.1, 52.6, 34.7, 25.5.

^{19}F NMR (377 MHz, $CDCl_3$) δ -62.9.

HRMS (ESI$^{+/-}$, APCI) Not found.

N-(4-((3-Iodobicyclo[1.1.1]pentan-1-yl)sulfonyl)phenyl)acetamide, 3o-I

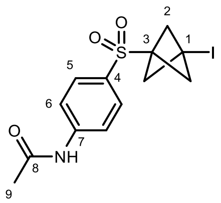

A stock solution of sulfinate salt **2o** (133 mg, 0.60 mmol) in DMF (0.60 mL) was prepared. DIH (78.4 mg, 0.20 mmol), sulfinate salt **2o** (0.50 mL, 0.50 mmol, 1.0 M in DMF), [1.1.1]propellane **1** (0.29 mL, 0.20 mmol, 0.69 M in Et_2O) in CH_2Cl_2 (0.80 mL) were subjected to the conditions of **general procedure B** (-40 °C). BCP iodide **3o-I** (33.4 mg, 0.0854 mmol, 43%) was obtained as an off-white solid.

m.p. 136 °C.

IR v_{max}/cm^{-1} (film) 1675, 1591, 1532, 1402, 1322, 1309, 1169, 1134, 730.

^{1}H NMR (600 MHz, $CDCl_3$) δ 7.80 – 7.77 (2H, m, H5), 7.76 – 7.68 (2H, m, H6), 7.41 (1H, s, NH), 2.50 (6H, s, H2), 2.24 (3H, s, H9).

^{13}C NMR (151 MHz, $CDCl_3$) δ 168.6, 143.3, 131.3, 130.1, 119.5, 59.3, 58.0, 25.0, 2.4.

HRMS (ESI$^+$) [M + H]$^+$ $C_{13}H_{15}O_3INS$ requires 391.9808; found 391.9811.

N-(4-((3-Bromobicyclo[1.1.1]pentan-1-yl)sulfonyl)phenyl)acetamide, 3o-Br

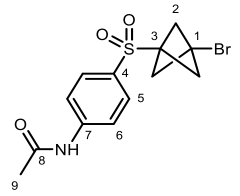

A stock solution of sulfinate salt **2o** (133 mg, 0.60 mmol) in H_2O (0.60 mL) was prepared. DBH (57.1 mg, 0.20 mmol), sulfinate salt **2o** (0.50 mL, 0.50 mmol, 1.0 M in H_2O), [1.1.1]propellane **1** (0.29 mL, 0.20 mmol, 0.69 M in Et_2O) in Et_2O (0.20 mL) were subjected to the conditions of **general procedure C**. BCP bromide **3o-Br** (31.5 mg, 0.0915 mmol, 46%) was obtained as a white solid.

m.p. 217 °C.
IR ν_{max}/cm^{-1} (film) 1680, 1591, 1531, 1322, 1184, 1140, 732.
^{1}H NMR (600 MHz, $CDCl_3$) δ 7.81 – 7.78 (2H, m, H5), 7.76 – 7.70 (2H, m, H6), 7.38 (1H, s, NH), 2.45 (6H, s, H2), 2.25 (3H, s, H9).
^{13}C NMR (151 MHz, $CDCl_3$) δ 168.6, 143.3, 131.5, 130.1, 119.5, 58.1, 52.7, 35.4, 25.0.
HRMS (APCI$^+$) $[M + H]^+$ $C_{13}H_{15}O_3{}^{79}BrNS^+$ requires 343.9951; found 343.9953.

1,3-Bis((3-bromobicyclo[1.1.1]pentan-1-yl)sulfonyl)benzene, 3p-I

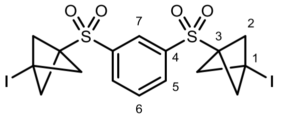

A stock solution of sulfinate salt **2p** (75.0 mg, 0.300 mmol) in DMF/H_2O (0.60 mL, 1:1) was prepared. DIH (78.4 mg, 0.20 mmol), sulfinate salt **2p** (0.50 mL, 0.25 mmol, 0.50 M in DMF/H_2O), [1.1.1]propellane **1** (0.29 mL, 0.20 mmol, 0.69 M in Et_2O) in CH_2Cl_2 (0.80 mL) were subjected to the conditions of **general procedure B** (-40 °C). Purification by column chromatography (SiO_2, EtOAc/pentane, 1:19) gave BCP iodide **3p-I** (15.3 mg, 0.0259 mmol, 26%) as a yellow solid.

R_f 0.60 (EtOAc/pentane, 2:3).
m.p. 246 °C.
IR ν_{max}/cm^{-1} (film) 1537, 1384, 1353, 1308, 1169, 1149, 1137, 649.

^{1}H NMR (600 MHz, $CDCl_3$) δ 8.31 (1H, dt, *J* = 1.8, 0.9 Hz, H7), 8.15 (2H, dd, *J* = 7.8, 1.8 Hz, H5), 7.83 (1H, td, *J* = 7.8, 0.5 Hz, H6), 2.53 (12H, s, H2).

^{13}C NMR (151 MHz, $CDCl_3$) δ 139.1, 133.9, 130.9, 128.9, 59.3, 57.8, 1.3.

HRMS ($ESI^{+/-}$, EI) Not found.

1,3-Bis((3-bromobicyclo[1.1.1]pentan-1-yl)sulfonyl)benzene, 3p-Br

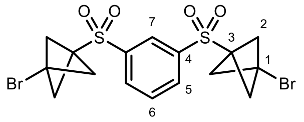

A stock solution of sulfinate salt **2p** (150 mg, 0.60 mmol) in H_2O (0.60 mL) was prepared. DBH (57.1 mg, 0.20 mmol), sulfinate salt **2p** (0.25 mL, 0.25 mmol, 1.0 M in H_2O), [1.1.1]propellane **1** (0.29 mL, 0.20 mmol, 0.69 M in Et_2O) in Et_2O (0.20 mL) were subjected to the conditions of **general procedure C**. Purification by silica plug (EtOAc/pentane, 1:4) gave BCP bromide **3p-Br** (45.6 mg, 0.0919 mmol, 92%) as a white solid.

R_f 0.63 (EtOAc/pentane, 2:3).

m.p. 250 – 260 ° C.

IR v_{max}/cm^{-1} (film) 1321, 1203, 1181, 1132, 800, 688, 637.

^{1}H NMR (400 MHz, $CDCl_3$) δ 8.33 (1H, td, *J* = 1.8, 0.5 Hz, H7), 8.17 (2H, dd, *J* = 8.0, 1.8 Hz, H5), 7.85 (1H, td, *J* = 8.0 0.5 Hz, H6), 2.49 (12H, s, H2).

^{13}C NMR (101 MHz, $CDCl_3$) δ 139.2, 133.9, 131.0, 128.8, 58.1, 52.6, 34.9.

HRMS (ESI^+) Not found.

1-Iodo-5-tosylbicyclo[3.1.1]heptane, 5a-I

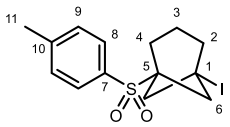

A stock solution of sulfinate salt **2a** (534 mg, 3.00 mmol) in H_2O (3.0 mL) was prepared. DIH (78.4 mg, 0.20 mmol), sulfinate salt **2a** (0.50 mL, 0.50 mmol, 1.0 M in H_2O), [3.1.1]propellane **4** (0.87 mL, 0.20 mmol, 0.23 M in Et_2O) in CH_2Cl_2 (0.80 mL) were subjected to the conditions of **general procedure A**. Purification by silica plug (EtOAc/pentane, 1:4) gave sulfonyl BCHep iodide **5a-I** (65.4 mg, 0.174 mmol, 87%) as a white solid, which could be recrystalised from CH_2Cl_2/pentane.

R$_f$ 0.30 (EtOAc/pentane, 1:9).

m.p. 134 – 140 °C.

IR ν_{max}/cm^{-1} (film) 2954, 1596, 1310, 1300, 1290, 1150, 1108, 1079, 857, 816, 671.

^{1}H NMR (400 MHz, $CDCl_3$) δ 7.69 – 7.62 (2H, m, H8), 7.38 – 7.31 (2H, m, H9), 3.23 (2H, dt, *J* = 7.6, 3.8 Hz, H6), 2.52 – 2.44 (2H, m, H), 2.45 (3H, s, H11), 2.35 – 2.25 (2H, m, H6), 1.91 – 1.82 (4H, m, H).

^{13}C NMR (101 MHz, $CDCl_3$) δ 145.1, 132.6, 130.0, 129.3, 65.4, 46.2, 42.5, 26.7, 25.9, 21.8, 19.3.

HRMS (ESI$^+$) [M + Na]$^+$ $C_{14}H_{17}O_2INaS^+$ requires 398.9886; found 398.9886.

1-Bromo-5-tosylbicyclo[3.1.1]heptane, 5a-Br

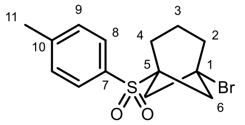

A stock solution of sulfinate salt **2a** (534 mg, 3.00 mmol) in H_2O (3.0 mL) was prepared. DBH (57.1 mg, 0.20 mmol), sulfinate salt **2a** (0.50 mL, 0.50 mmol, 1.0 M in H_2O), [3.1.1]propellane **4** (0.87 mL, 0.20 mmol, 0.23 M in Et_2O) in Et_2O (0.20 mL) were subjected to the conditions of **general procedure C**. BCP bromide **5a-Br** (65.7 mg, 0.20 mmol, 99%) was obtained as a white solid.

R$_f$ 0.32 (EtOAc/pentane, 1:19).

m.p. 93 ° C.

IR ν_{max}/cm^{-1} (film) 1311, 1301, 1291, 1151, 1113, 1080, 671, 613.

^{1}H NMR (400 MHz, $CDCl_3$) δ 7.71 – 7.64 (2H, m, H8), 7.39 – 7.31 (2H, m, H9), 3.14 – 3.04 (2H, m, H6), 2.45 (3H, s, H11), 2.34 – 2.29 (2H, m, H4), 2.26 – 2.17 (2H, m, H6), 1.94 – 1.86 (2H, m, H3), 1.86 – 1.81 (2H, m, H2).

^{13}C NMR (101 MHz, $CDCl_3$) δ 145.1, 132.8, 130.0, 129.4, 62.4, 52.3, 44.5, 39.3, 26.0, 21.8, 18.7.

HRMS (ESI$^+$) [M + Na]$^+$ $C_{14}H_{17}O_2{}^{79}BrNaS^+$ requires 351.0025; found 351.0027.

2.2 Heteroaryl Sulfonyl BCP Halides

2-((3-Iodobicyclo[1.1.1]pentan-1-yl)sulfonyl)thiophene, 3q-I

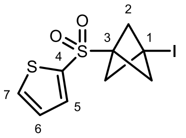

A stock solution of sulfinate salt **2q** (102 mg, 0.599 mmol) in H_2O (0.60 mL) was prepared. DIH (39.2 mg, 0.10 mmol), sulfinate salt **2q** (0.25 mL, 0.25 mmol, 1.0 M in H_2O), [1.1.1]propellane **1** (0.15 mL, 0.10 mmol, 0.59 M in Et_2O) in CH_2Cl_2 (0.40 mL) were subjected to the conditions of **general procedure A**. BCP iodide **3a-I** (27.9 mg, 0.0820 mmol, 82%) was obtained as a white solid.

R$_f$ 0.20 (EtOAc/pentane, 1:17).

m.p. 182 ° C.

IR ν_{max}/cm^{-1} (film) 1400, 1305, 1199, 1167, 1128, 1017, 868, 856, 741, 616.

^{1}H NMR (400 MHz, $CDCl_3$) δ 7.77 (1H, dd, *J* = 5.0, 1.3 Hz, H7), 7.65 (1H, dd, *J* = 3.8, 1.3 Hz, H5), 7.19 (1H, dd, *J* = 5.0, 3.8 Hz, H6), 2.56 (6H, s, H2).

^{13}C NMR (101 MHz, $CDCl_3$) δ 137.5, 135.1, 135.0, 128.4, 59.4, 58.5, 1.8.

HRMS (ESI$^+$) $[M + H]^+$ $C_9H_{10}O_2IS_2^+$ requires 340.9161; found 340.9161.

2-((3-Bromobicyclo[1.1.1]pentan-1-yl)sulfonyl)thiophene, 3q-Br

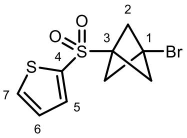

A stock solution of sulfinate salt **2q** (204 mg, 1.20 mmol) in H_2O (1.20 mL) was prepared. DBH (57.1 mg, 0.20 mmol), sulfinate salt **2q** (0.50 mL, 0.50 mmol, 1.0 M in H_2O), [1.1.1]propellane **1** (0.34 mL, 0.20 mmol, 0.59 M in Et_2O) in Et_2O (0.20 mL) were subjected to the conditions of **general procedure C**. BCP Bromide **3q-Br** (53.4 mg, 0.182 mmol, 91%) was obtained as an off-white solid.

Multigram Scale: The conditions of **general procedure E** *(isolated RSO_2Br)* gave BCP bromide **3q-Br** (2.52 g, 8.60 mmol, 98%) as a beige solid.

R_f 0.20 (EtOAc/pentane, 1:17).

m.p. 132 – 133 °C.

IR ν_{max}/cm^{-1} (film) 1401, 1344, 1202, 1181, 1133, 880, 742, 671, 618.

^{1}H NMR (400 MHz, $CDCl_3$) δ 7.77 (1H, dd, *J* = 5.0, 1.3 Hz, H7), 7.67 (1H, dd, *J* = 3.8, 1.3 Hz, H5), 7.20 (1H, dd, *J* = 5.0, 3.8 Hz, H6), 2.51 (6H, s, H2).

^{13}C NMR (151 MHz, $CDCl_3$) δ 137.7, 135.1, 135.0, 128.4, 58.2, 53.3, 35.0.

HRMS (ESI^+) $[M + NH_4]^+$ $C_9H_{13}{}^{79}BrNO_2S_2{}^+$ requires 311.9551; found 311.9543.

2-Bromo-5-((3-iodobicyclo[1.1.1]pentan-1-yl)sulfonyl)thiophene, 3r-I

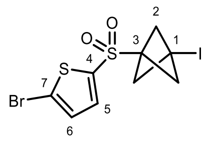

A stock solution of sulfinate salt **2r** (376 mg, 1.20 mmol, contained 21% impurity) in H_2O (1.20 mL) was prepared. DIH (78.4 mg, 0.20 mmol), sulfinate salt **2r** (0.50 mL, 0.50 mmol, 1.0 M in H_2O), [1.1.1]propellane **1** (0.29 mL, 0.20 mmol, 0.69 M in Et_2O) in CH_2Cl_2 (0.80 mL) were subjected to the conditions of **general procedure A.** BCP iodide **3r-I** (82.8 mg, 0.198 mmol, 99%) was obtained as a pale-yellow solid.

R_f 0.29 (EtOAc/pentane, 1:17).

m.p. 169 °C.

IR ν_{max}/cm^{-1} (film) 1399, 1316, 1201, 1164, 1129, 858, 678, 625.

^{1}H NMR (600 MHz, $CDCl_3$) δ 7.41 (1H, d, *J* = 4.0 Hz, H5), 7.16 (1H, d, *J* = 4.0 Hz, H6), 2.57 (6H, s, H2).

^{13}C NMR (151 MHz, $CDCl_3$) δ 138.3, 135.3, 131.5, 123.4, 59.4, 58.4, 1.4.

HRMS (ESI^+) Not Found.

2-Bromo-5-((3-bromobicyclo[1.1.1]pentan-1-yl)sulfonyl)thiophene, 3r-Br

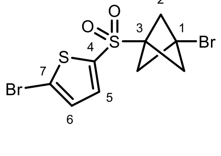

A stock solution of sulfinate salt **2r** (376 mg, 1.20 mmol, contained 21% impurity) in H_2O (1.20 mL) was prepared. DBH (57.1 mg, 0.20 mmol), sulfinate salt **2r** (0.50 mL, 0.50 mmol, 1.0 M in

H_2O), [1.1.1]propellane **1** (0.29 mL, 0.20 mmol, 0.69 M in Et_2O) in Et_2O (0.20 mL) were subjected to the conditions of **general procedure C**. BCP iodide **3r-Br** (74.0 mg, 0.20 mmol, 99%) was obtained as a pale-yellow solid.

R$_f$ 0.28 (EtOAc/pentane, 1:17).
m.p. 174 ° C.
IR ν_{max}/cm^{-1} (film) 1399, 1316, 1204, 1177, 1136, 870, 679, 628.
^{1}H NMR (600 MHz, $CDCl_3$) δ 7.42 (1H, d, *J* = 4.0 Hz, H5), 7.17 (1H, d, *J* = 4.0 Hz, H6), 2.53 (6H, s, H2).
^{13}C NMR (151 MHz, $CDCl_3$) δ 138.4, 135.3, 131.5, 123.4, 58.2, 53.3, 34.8.
HRMS (ESI$^+$) Not Found.

3-((3-Iodobicyclo[1.1.1]pentan-1-yl)sulfonyl)-1-methyl-1H-pyrazole, 3s-I

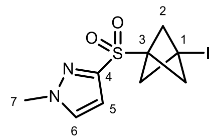

A stock solution of sulfinate salt **2s** (131 mg, 0.545 mmol, contained 30% impurity) in H_2O (0.60 mL) was prepared. DIH (39.2 mg, 0.103 mmol), sulfinate salt **2s** (0.25 mL, 0.25 mmol, 1.0 M in H_2O), [1.1.1]propellane **1** (0.15 mL, 0.10 mmol, 0.70 M in Et_2O) in CH_2Cl_2 (0.40 mL) were subjected to the conditions of **general procedure A**. BCP iodide **3s-I** (32.8 mg, 0.970 mmol, 92%) was obtained as a pale-yellow solid.

R$_f$ 0.31(EtOAc/pentane, 2:3) [UV, PMA].
m.p. 182° C.
IR ν_{max}/cm^{-1} (film) 1521, 1308, 1201, 1169, 1141, 1111, 861, 706, 662.
^{1}H NMR (400 MHz, $CDCl_3$) δ 7.80 (1H, m, H6), 7.75 (1H, d, *J* = 0.7 Hz, H5), 3.98 (3H, d, *J* = 0.4 Hz, H7), 2.55 (6H, s, H2).
^{13}C NMR (151 MHz, $CDCl_3$) δ 139.9, 133.2, 119.3, 59.1, 58.5, 40.0, 2.3.
HRMS (ESI$^+$) $[M + H]^+$ $C_9H_{12}O_2N_2IS_2^+$ requires 338.9659; found 338.9659.

3-((3-Bromobicyclo[1.1.1]pentan-1-yl)sulfonyl)-1-methyl-1H-pyrazole, 3s-Br

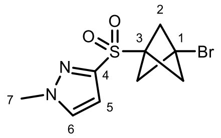

A stock solution of sulfinate salt **2s** (131 mg, 0.545 mmol, contained 30% impurity) in H_2O (0.60 mL) was prepared. DBH (28.6 mg, 0.100 mmol), sulfinate salt **2s** (0.25 mL, 0.25 mmol, 1.0 M in H_2O), [1.1.1]propellane **1** (0.15 mL, 0.10 mmol, 0.70 M in Et_2O) in Et_2O (0.10 mL) were subjected to the conditions of **general procedure C**. Purification by silica plug (EtOAc/pentane 1:9 → 1:1) gave BCP Bromide **3s-Br** (28.7 mg, 0.99 mmol, 99%) was obtained as a white solid.

R$_f$ 0.31(EtOAc/pentane, 2:3) [UV, PMA].

m.p. 145° C.

IR ν_{max}/cm^{-1} (film) 1522, 1388, 1310, 1175, 1113, 875, 667.

^{1}**H NMR** (400 MHz, $CDCl_3$) δ 7.81 (1H, m, H6), 7.76 (1H, d, *J* = 0.7 Hz, H5), 3.98 (3H, d, *J* = 0.4 Hz, H3), 2.50 (6H, s, H2).

^{13}**C NMR** (151 MHz, $CDCl_3$) δ 139.9, 133.2, 119.5, 57.9, 53.2, 40.0, 35.3.

HRMS (ESI$^+$) $[M + H]^+$ $C_9H_{12}O_2N_2{}^{79}BrS_2{}^+$ requires 290.9797; found 290.9797.

4-((3-Bromobicyclo[1.1.1]pentan-1-yl)sulfonyl)-1-methyl-1H-pyrazole, 3t-Br

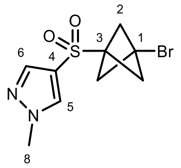

The conditions of **general procedure D** *(in situ RSO_2Br with NBS)* gave BCP bromide **3t-Br** (0.249 g, 0.86 mmol, 15%) as a beige solid. An analytically pure sample was purified by HPLC (AGILENT 1260 INFINITY, 2 – 15 min, H_2O/MeOH, 10 – 60%, flow 30 mLmin^{-1} (loading pump 4 mLmin^{-1}), column Cromatorex C18, 5 µm, 100*19 mm).

R$_f$ 0.14 (hexane/EtOAc, 7:3).

m.p. 156 – 157 °C.

^{1}**H NMR** (500 MHz, $CDCl_3$) δ 7.81 (1H, s, H6), 7.75 (1H, s, H5), 3.97 (3H, s, H7), 2.49 (6H, s, H2).

^{13}**C NMR** (151 MHz, $CDCl_3$) δ 139.9, 133.2, 119.4, 57.8, 53.2, 39.9, 35.3.

HRMS (ESI$^+$) $[M + H]^+$ $C_9H_{11}{}^{79}BrN_2O_2S^+$ requires 289.9782; found 289.9785.

3-((3-Bromobicyclo[1.1.1]pentan-1-yl)sulfonyl)furan, 3u-Br

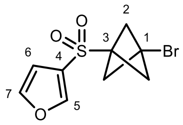

To a solution of PPh_3 (0.30 g, 1.10 mmol, 0.95 equiv.) in dry CH_3CN (20 mL) was slowly added bromine (0.17 g, 6.10 mmol, 0.95 equiv.) under Ar atmosphere at 0 °C. The mixture was stirred at 0 °C for 30 min, and lithium furan-3-sulfinate (0.16 g, 1.17 mmol, 1.00 equiv.) was added in one portion. The resulting mixture was stirred at 0 °C for 15 min, and a solution of [1.1.1]propellane **1** (3 mL, 1.80 mmol, 0.7 M in Et_2O, 1.50 equiv.) was added. The mixture was stirred at room temperature for 15 h. The solvent was removed under reduced pressure. The final product was purified by column chromatography (SiO_2, hexane/EtOAc, 7:3) to give BCP bromide **3u-Br** (1.00 g, 3.65 mmol, 57%) as a white powder.

R*f* 0.60 (hexane/EtOAc, 7:3).
m.p. 138 – 139 °C.
^{1}H NMR (500 MHz, $CDCl_3$) δ 7.99 (s, 1H), 7.56 (t, *J* = 1.7 Hz, 1H), 6.64 (d, *J* = 1.2 Hz, 1H), 2.53 (s, 6H).
^{13}C NMR (126 MHz, $CDCl_3$) δ 147.8, 145.4, 124.9, 109.1, 57.9, 52.8, 35.0.
HRMS ($ESI^{+/-}$) Not found.

4-((3-Iodobicyclo[1.1.1]pentan-1-yl)sulfonyl)–3,5-dimethylisoxazole, 3v-I

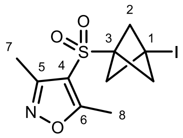

A stock solution of sulfinate salt **2v** (220 mg, 1.201 mmol) in H_2O (1.20 mL) was prepared. DIH (78.4 mg, 0.20 mmol), sulfinate salt **2v** (0.50 mL, 0.50 mmol, 1.0 M in H_2O), [1.1.1]propellane **1** (0.29 mL, 0.20 mmol, 0.69 M in Et_2O) in CH_2Cl_2 (0.80 mL) were subjected to the conditions of **general procedure A.** BCP iodide **3v-I** (23.6 mg, 0.0668 mmol, 33%) was obtained as a white solid.

R$_f$ 0.65 (EtOAc/pentane, 1:9).
m.p. 144 ° C.
IR ν_{max}/cm^{-1} (film) 2342, 1586, 1408, 1315, 1271, 1183, 1101, 860, 669, 645.

^{1}H NMR (600 MHz, $CDCl_3$) δ 2.63 (3H, s, H8), 2.58 (6H, s, H2), 2.39 (3H, s, H7).
^{13}C NMR (151 MHz, $CDCl_3$) δ 175.8, 158.4, 113.3, 58.9, 12.8, 11.0, 1.3.
HRMS (ESI$^{+/-}$) Not Found.

4-((3-Bromobicyclo[1.1.1]pentan-1-yl)sulfonyl)-3,5-dimethylisoxazole, 3v-Br

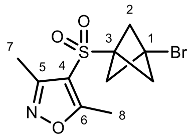

A stock solution of sulfinate salt **2v** (220 mg, 1.20 mmol) in H_2O (1.20 mL) was prepared. DBH (57.1 mg, 0.20 mmol), sulfinate salt **2v** (0.50 mL, 0.50 mmol, 1.0 M in H_2O), [1.1.1]propellane **1** (0.29 mL, 0.20 mmol, 0.69 M in Et_2O) in Et_2O (0.20 mL) were subjected to the conditions of **general procedure C**. Purification by column chromatography (SiO_2, EtOAc/pentane, 1:9) gave BCP bromide **3v-Br** (40.8 mg, 0.133 mmol, 67%) as a white solid.

R_f 0.63 (EtOAc/pentane, 1:9).
m.p. 138 – 140 ° C.
IR ν_{max}/cm^{-1} (film) 2981, 1576, 1407, 1379, 1317, 1271, 1191, 1183, 1166, 1103, 873, 689, 645.
^{1}H NMR (600 MHz, $CDCl_3$) δ 2.63 (3H, s, H7/8), 2.53 (6H, s, H2), 2.39 (3H, s, H7/8).
^{13}C NMR (151 MHz, $CDCl_3$) δ 175.8, 158.4, 113.5, 57.7, 53.5, 34.7, 12.8, 11.0.
HRMS (ESI$^{+/-}$) Not found.

3-((3-Bromobicyclo[1.1.1]pentan-1-yl)sulfonyl)pyridine, 3w-Br

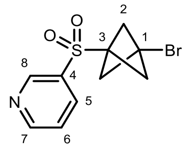

The conditions of **general procedure D** *(in situ RSO_2Br with NBS)* gave BCP bromide **3w-Br** (0.108 g, 0.375 mmol, 31%) as a beige solid. An analytically pure sample was purified by HPLC (AGILENT 1260 INFINITY, 1 – 5 min, H_2O/MeCN, 15 – 65%, flow 30 mLmin^{-1} (loading pump 4 mLmin^{-1}), column Cromatorex C18, 5 µm, 100*19 mm).

Alternative Preparation: A stock solution of sulfinate salt **2w** (99.0 mg, 0.599 mmol) in H_2O (0.60 mL) was prepared. DBH (57.1 mg, 0.20 mmol), sulfinate salt **2w** (0.50 mL, 0.50 mmol, 1.0 M in H_2O), [1.1.1]propellane **1** [1.1.1]propellane **1** (0.29 mL, 0.20 mmol, 0.70 M in Et_2O) in Et_2O (0.20 mL) were subjected to the conditions of **general procedure C**. Purification by column chromatography (SiO_2, EtOAc/pentane, 1:4) gave BCP bromide **3w-I** (13.8 mg, 0.0479 mmol, 24%) was obtained as a white solid.

R$_f$ 0.26 (EtOAc/pentane, 1:4).
m.p. 165 – 166 °C.
IR ν_{max}/cm^{-1} (film) 1576, 1568, 1416, 1312, 1184, 1150, 703.
^{1}H NMR (600 MHz, $CDCl_3$) δ 9.07 (1H, d, *J* = 2.4 Hz, H8), 8.92 (1H, dd, *J* = 4.9, 1.7 Hz, H7), 8.15 (1H, ddd, *J* = 8.1, 2.4, 1.7 Hz, H5), 7.55 (1H, app. ddd, *J* = 8.1, 4.9, 0.9 Hz, H6), 2.50 (6H, s, H2).
^{13}C NMR (151 MHz, $CDCl_3$) δ 154.9, 149.7, 136.4, 133.6, 124.1, 58.0, 52.9, 35.1.
HRMS (ESI$^+$) $[M + H]^+$ $C_{10}H_{11}O_2N^{81}BrS^+$ requires 289.9667; found 289.9668.

*Note: Attempts to prepare corresponding iodide **3w-I** under **general procedure C** were unsuccessful.*

5-((3-Bromobicyclo[1.1.1]pentan-1-yl)sulfonyl)-2-methoxypyridine, 3x-Br

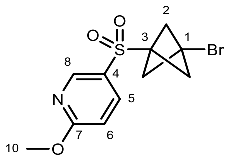

The conditions of **general procedure D** *(in situ RSO_2Br with NBS)* and purification by column chromatography (SiO_2, hexane/MTBE, 7:3) gave BCP bromide **3x-Br** (0.25 g, 0.786 mmol, 15%) as a beige solid.

R$_f$ 0.56 (hexane/EtOAc, 7:3).
m.p. 169 – 170 °C.
^{1}H NMR (500 MHz, DMSO-d_6) δ 8.59 (1H, s, H8), 8.04 (1H, d, *J* = 7.4 Hz, H5), 7.07 (1H, d, *J* = 7.7 Hz, H6), 3.97 (3H, s, H9), 2.53 (6H, s, H2).
^{13}C NMR (126 MHz, DMSO-d_6) δ 166.9, 148.5, 138.8, 126.1, 111.8, 57.5, 54.4, 52.3, 36.1.
HRMS (ESI$^+$) $[M + H]^+$ $C_{11}H_{13}{}^{81}BrNO_3S^+$ requires 319.9779; found 319.9774.

5-((3-Bromobicyclo[1.1.1]pentan-1-yl)sulfonyl)-2-chloropyridine, 3y-Br

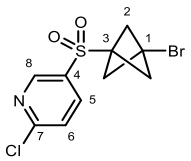

The conditions of **general procedure D** *(in situ* RSO_2Br *with NBS)* gave BCP bromide **3y-Br** (0.479 g, 1.485 mmol, 31%) as a beige solid. An analytically pure sample was purified by HPLC (AGILENT 1260 INFINITY, 2 – 10 min, H_2O/MeCN, 30 – 55%, flow 30 $mLmin^{-1}$ (loading pump 4 $mLmin^{-1}$), column Cromatorex C18, 5 µm, 100*19 mm).

R_f 0.43 (hexane/EtOAc, 7:3).

m.p. 193 – 194 °C.

^{1}H NMR (500 MHz, $CDCl_3$) δ 8.82 (1H, d, *J* = 2.3 Hz, H8), 8.07 (1H, dd, *J* = 8.3, 2.5 Hz, H5), 7.55 (1H, d, *J* = 8.3 Hz, H6), 2.50 (6H, s, H2).

^{13}C NMR (151 MHz, $CDCl_3$) δ 157.6, 150.0, 138.7, 132.5, 125.2, 58.0, 52.9, 34.9.

HRMS (ESI^+) $[M + H]^+$ $C_{10}H_{10}{}^{81}BrClNO_2S^+$ requires 323.9284; found 323.9284.

2.3 Pharmaceutical and Agrochemical BCP Halides

5-(2-Ethoxy-5-((3-iodobicyclo[1.1.1]pentan-1-yl)sulfonyl)phenyl)-1-methyl-3-propyl-1,6-dihydro–7H-pyrazolo[4,3-d]pyrimidin–7-one, 3z-I

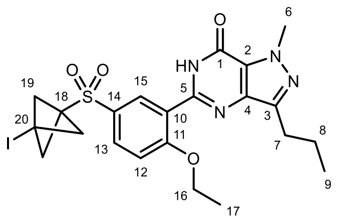

A stock solution of sulfinate salt **2z** (548 mg, 1.10 mmol) in H_2O (1.10 mL) was prepared. DIH (78.4 mg, 0.20 mmol), sulfinate salt **2z** (0.50 mL, 0.50 mmol, 1.0 M in DMF), [1.1.1]propellane **1** (0.29 mL, 0.20 mmol, 0.69 M in Et_2O) in CH_2Cl_2 (0.80 mL) were subjected to the conditions of **general procedure B** (-40 °C). Purification by column chromatography (SiO_2, EtOAc/pentane 3:7 → 4:1) gave BCP iodide **3z-I** (95.5 mg, 0.168 mmol, 84%) as a white solid.

R_f 0.43 (EtOAc/pentane, 1:1).

m.p. 220 °C.

IR ν_{max}/cm^{-1} (film) 3322, 2960, 2361, 1698, 1320, 1170, 1140, 1030, 732, 619.

^{1}H NMR (600 MHz, $CDCl_3$) δ 10.75 (1H, s, NH), 8.90 (1H, d, *J* = 2.4 Hz, H15), 7.92 (1H, dd, *J* = 8.8, 2.4 Hz, H13), 7.19 (1H, d, *J* = 8.8 Hz, H12), 4.40 (2H, q, *J* = 7.0 Hz, H16), 4.28 (3H, s, H6), 2.94 (2H, t, *J* = 7.6 Hz, H7), 2.57 (6H, s, H19), 1.87 (2H, app. sxt., *J* = 7.4 Hz, H8), 1.66 (3H, t, *J* = 7.0 Hz, H17), 1.04 (3H, t, *J* = 7.4 Hz, H9).

^{13}C NMR (151 MHz, $CDCl_3$) δ 160.4, 153.7, 147.3, 146.2, 138.5, 132.4, 132.3, 130.0, 124.7, 121.7, 113.5, 66.5, 59.4, 58.0, 38.4, 27.9, 22.4, 14.7, 14.2, 2.3.

HRMS (ESI^+) $[M + H]^+$ $C_{22}H_{26}O_4IN_4S^+$ requires 569.0713; found 569.0711.

5-(5-((3-Bromobicyclo[1.1.1]pentan-1-yl)sulfonyl)-2-ethoxyphenyl)-1-methyl-3-propyl- 1,6-dihydro–7H-pyrazolo[4,3-d]pyrimidin–7-one, 3z-Br

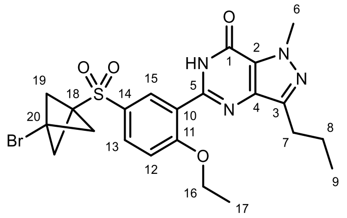

A stock solution of sulfinate salt **2z** (548 mg, 1.10 mmol) in H_2O (1.10 mL) was prepared. DBH (57.1 mg, 0.20 mmol), sulfinate salt **2z** (0.50 mL, 0.50 mmol, 1.0 M in H_2O), [1.1.1]propellane **1** (0.34 mL, 0.20 mmol, 0.59 M in Et_2O) in Et_2O (0.20 mL) were subjected to the conditions of **general procedure C**. Purification by column chromatography (SiO_2, EtOAc/pentane 1:9 → 1:4) gave BCP bromide **3z-Br** (78.9 mg, 0.152 mmol, 76%) as a white solid.

R_f 0.40 (EtOAc/pentane, 1:1).

m.p. 166 °C.

IR ν_{max}/cm^{-1} (film) 1774, 1724, 1701, 1180, 1145, 773.

^{1}H NMR (600 MHz, $CDCl_3$) δ 10.78 (1H, s, NH), 8.89 (1H, d, *J* = 2.4 Hz, H15), 7.93 (1H, dd, *J* = 8.7, 2.5 Hz, H13), 7.19 (1H, d, *J* = 8.8 Hz, H12), 4.40 (2H, q, *J* = 7.0 Hz, H16), 4.28 (3H, s, H6), 2.94 (2H, t, *J* = 7.6 Hz, H7), 2.52 (6H, s, H19), 1.87 (2H, app. sxt., *J* = 7.4 Hz, H8), 1.66 (3H, t, *J* = 7.0 Hz, H17), 1.04 (3H, t, *J* = 7.4 Hz, H9).

^{13}C NMR (151 MHz, $CDCl_3$) δ 160.4, 153.7, 147.3, 146.2, 138.5, 132.4, 132.3, 130.1, 124.7, 121.8, 113.5, 66.4, 58.1, 52.8, 38.4, 35.4, 27.9, 22.4, 14.7, 14.2.

HRMS (APCI$^+$) [M + H]$^+$ $C_{22}H_{26}O_4BrN_4S^+$ requires 521.0853; found 521.0852.

N,N-Diethyl-3-((3-iodobicyclo[1.1.1]pentan-1-yl)sulfonyl)-4H-1,2,4-triazole-4-carboxamide, 3aa-I

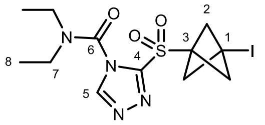

A stock solution of sulfinate salt **2aa** (124 mg, 0.60 mmol) in DMF (0.60 mL) was prepared. DIH (78.4 mg, 0.20 mmol), sulfinate salt **2aa** (0.50 mL, 0.50 mmol, 1.0 M in DMF), [1.1.1]propellane **1** (0.29 mL, 0.20 mmol, 0.69 M in Et_2O) in CH_2Cl_2 (0.80 mL) were subjected to the conditions of **general procedure B** (-40 °C). Purification by column chromatography (SiO_2,

EtOAc/pentane 1:9 → 1:4) gave BCP iodide **3aa-I** (46.7 mg, 0.110 mmol, 55%) as a colourless oil.

R$_f$ 0.67 (EtOAc/pentane, 3:7).
IR ν_{max}/cm^{-1} (film) 1718, 1387, 1337, 1201.
^{1}H NMR (600 MHz, $CDCl_3$) δ 8.90 (1H, s, H5), 3.68 – 3.50 (4H, m, H7), 2.70 (6H, s, H2), 1.31 (6H, t, J = 7.1 Hz, H8).
^{13}C NMR (151 MHz, $CDCl_3$) δ 160.6, 148.3, 147.6, 59.8, 57.1, 44.3, 14.2, 12.5, 1.6.
HRMS (ESI$^+$) [M + Na]$^+$ $C_{12}H_{17}O_3N_4INaS^+$ requires 446.9958; found 446.9959.

3-((3-Bromobicyclo[1.1.1]pentan-1-yl)sulfonyl)-N,N-diethyl-4H-1,2,4-triazole-4-carboxamide, 3aa-Br

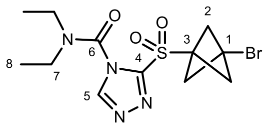

A stock solution of sulfinate salt **2aa** (139 mg, 0.55 mmol) in H_2O (0.55 mL) was prepared. DBH (57.1 mg, 0.20 mmol), sulfinate salt **2aa** (0.50 mL, 0.50 mmol, 1.0 M in H_2O), [1.1.1]propellane **1** (0.34 mL, 0.20 mmol, 0.59 M in Et_2O) in Et_2O (0.20 mL) were subjected to the conditions of **general procedure C**. Purification by column chromatography (SiO_2, EtOAc/pentane 1:9 → 1:4) gave BCP bromide **3aa-Br** (44.7 mg, 0.119 mmol, 59%) as a white solid.

R$_f$ 0.28 (EtOAc/pentane, 1:4).
m.p. 111° C.
IR ν_{max}/cm^{-1} (film) 2981, 1713, 1338, 1203, 1183, 1138.
^{1}H NMR (600 MHz, $CDCl_3$) δ 8.91 (1H, s, H5), 3.74 – 3.48 (4H, br. m, H7), 2.66 (6H, s, H2), 1.32 (6H, t, J = 7.1 Hz, H8).
^{13}C NMR (151 MHz, $CDCl_3$) δ 160.7, 148.3, 147.7, 58.6, 51.9, 44.3, 34.9, 14.2, 12.5.
HRMS (ESI$^+$) [M + Na]$^+$ $C_{12}H_{17}O_3N_4BrNaS^+$ requires 399.0097; found 399.0099.

2.4 Alkyl Sulfonyl BCP Halides

1-Iodo-3-(methylsulfonyl)bicyclo[1.1.1]pentane, 7a-I

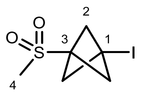

A stock solution of sulfinate salt **6a** (245 mg, 2.40 mmol) in H_2O (2.40 mL) was prepared. Benzyltrimethylammonium dichloroiodate (101 mg, 0.290 mmol), sulfinate salt **6a** (0.40 mL, 0.40 mmol, 1.0 M in H_2O), [1.1.1]propellane **1** (0.29 mL, 0.20 mmol, 0.70 M in Et_2O) in CH_2Cl_2 (0.20 mL) were subjected to the conditions of **general procedure F**. BCP iodide **7a-I** (54.0 mg, 0.199 mmol, 99%) was obtained as a white solid.

R$_f$ 0.30 (EtOAc/pentane, 3:7).
m.p. 182° C.
IR ν_{max}/cm^{-1} (film) 1296, 1188, 1167, 860, 838.
^{1}H NMR (400 MHz, $CDCl_3$) δ 2.84 (3H, s, H4), 2.69 (6H, s, H2).
^{13}C NMR (101 MHz, $CDCl_3$) δ 59.4, 57.2, 38.2, 1.2.
HRMS (ESI^{+}-) Not Found.

1-Bromo-3-(methylsulfonyl)bicyclo[1.1.1]pentane, 7a-Br

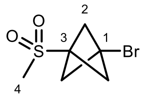

Miligramscale: A stock solution of sulfinate salt **6a** (245 mg, 2.40 mmol) in H_2O (2.40 mL) was prepared. Sulfinate salt **6a** (0.40 mL, 0.40 mmol, 1.0 M in H_2O), Br_2 (0.36 mL, 0.36 mmol, 1.0 M in CH_2Cl_2), [1.1.1]propellane **1** (0.29 mL, 0.20 mmol, 0.70 M in Et_2O) and Et_3B (20 µL, 0.02 mmol, 1.0 M in hexanes) in CH_2Cl_2 (0.20 mL) were subjected to the conditions of **general procedure G**. BCP iodide **7a-Br** (44.6 mg, 0.198 mmol, 98%) was obtained as a white solid.

Decagram Scale: The conditions of **general procedure E** *(isolated RSO_2Br)* gave BCP bromide **7a-Br** (34.10 g, 0.152 mol, 96%) as a white solid.

R$_f$ 0.22 (hexane/EtOAc, 7:3).
m.p. 114 – 115 °C.

IR ν_{max}/cm^{-1} (film) 1359, 1308, 1178, 1162, 1133, 910, 876, 733.

^{1}H NMR (400 MHz, $CDCl_3$) δ 2.87 (3H, s, H4), 2.64 (6H, s, H2).

^{13}C NMR (101 MHz, $CDCl_3$) δ 58.2, 51.9, 38.6, 34.3.

HRMS (ESI$^+$) [M + H]$^+$ $C_6H_{10}{}^{79}BrO_2S^+$ requires 226.9564; found 226.9557.

1-Bromo-3-(ethylsulfonyl)bicyclo[1.1.1]pentane, 7b-Br

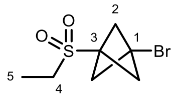

Decagram Scale: The conditions of **general procedure E** *(isolated RSO_2Br)* gave BCP bromide **7b-Br** (34.80 g, 0.146 mol, 95%) as a white solid.

R$_f$ 0.38 (hexane/EtOAc, 7:3).

m.p. 131 – 132 °C

^{1}H NMR (500 MHz, $CDCl_3$) δ 2.99 (q, *J* = 7.5 Hz, 2H), 2.64 (s, 6H), 1.39 (t, *J* = 7.5 Hz, 3H).

^{13}C NMR (126 MHz, $CDCl_3$) δ 58.5, 51.1, 45.8, 34.5, 6.3.

HRMS (ESI$^+$) [M + H]$^+$ $C_7H_{12}{}^{79}BrO_2S^+$ requires 238.9721; found 240.9718.

1-(Butylsulfonyl)-3-iodobicyclo[1.1.1]pentane, 7c-I

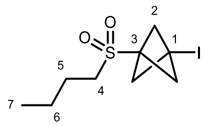

A stock solution of lithium sulfinate **6c** (25.6 mg, 0.209 mmol) in H_2O (0.20 mL) was prepared. Benzyltrimethylammonium dichloroiodate (50.5 mg, 0.145 mmol), lithium sulfinate **6c** (0.20 mL, 0.20 mmol, 1.0 M in H_2O), [1.1.1]propellane **1** (0.15 mL, 0.10 mmol, 0.70 M in Et_2O) in CH_2Cl_2 (0.10 mL) were subjected to the conditions of **general procedure F**. Purification by silica plug (pentane wash, followed by elution with EtOAc/pentane, 1:4) gave BCP iodide **7c-I** (20.0 mg, 0.0637 mmol, 64%) as a white solid.

R$_f$ 0.55 (EtOAc/pentane, 1:4) [goofy].

m.p. 110 ° C.

IR ν_{max}/cm^{-1} (film) 2980, 1318, 1290, 1269, 1203, 1165, 1119, 1100, 865, 623.

^{1}H NMR (600 MHz, $CDCl_3$) δ 2.96 – 2.88 (2H, m, H4), 2.64 (6H, s, H2), 1.88 – 1.77 (2H, m, H5), 1.47 (2H, sxt., J = 7.4 Hz, H6), 0.96 (3H, t, J = 7.4 Hz, H7).

^{13}C NMR (151 MHz, $CDCl_3$) δ 59.8, 56.8, 50.8, 23.5, 22.0, 13.7, 1.7.

HRMS ($ESI^{+/-}$) Not found.

1-Bromo-3-(butylsulfonyl)bicyclo[1.1.1]pentane, 7c-Br

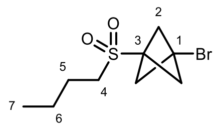

A stock solution of lithium sulfinate **6c** (25.6 mg, 0.20 mmol) in H_2O (0.20 mL) was prepared. Lithium sulfinate **6c** (0.20 mL, 0.20 mmol, 1.0 M in H_2O), Br_2 (0.18 mL, 0.18 mmol, 1.0 M in CH_2Cl_2), [1.1.1]propellane **1** (0.15 mL, 0.10 mmol, 0.70 M in Et_2O) and Et_3B (10 µL, 0.01 mmol, 1.0 M in hexanes) in CH_2Cl_2 (0.10 mL) were subjected to the conditions of **general procedure G**. BCP iodide **7c-Br** (17.0 mg, 0.0636 mmol, 64%) was obtained as a white solid.

R_f 0.60 (EtOAc/pentane, 1:4) [goofy].

m.p. 97 ° C.

IR ν_{max}/cm^{-1} (film) 1319, 1173, 1100, 668.

^{1}H NMR (600 MHz, $CDCl_3$) δ 2.99 – 2.90 (2H, m, H4), 2.64 (6H, s, H2), 1.85 – 1.79 (2H, m, H5), 1.48 (2H, sxt., J = 7.4 Hz, H6), 0.97 (3H, t, J = 7.4 Hz, H7).

^{13}C NMR (151 MHz, $CDCl_3$) δ 58.5, 51.4, 51.1, 34.6, 23.6, 22.0, 13.7.

HRMS ($ESI^{+/-}$) Not found.

1-Iodo-3-(phenethylsulfonyl)bicyclo[1.1.1]pentane, 7d-I

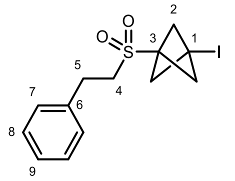

A stock solution of sulfinate salt **6d** (38.4 mg, 0.200 mmol) in H_2O (0.20 mL) was prepared. Benzyltrimethylammonium dichloroiodate (50.5 mg, 0.145 mmol), sulfinate salt **6d** (0.20 mL, 0.20 mmol, 1.0 M in H_2O), [1.1.1]propellane **1** (0.15 mL, 0.10 mmol, 0.70 M in Et_2O) in CH_2Cl_2 (0.10 mL) were subjected to the conditions of **general procedure F**. Purification by silica plug

(pentane wash, followed by elution with EtOAc/pentane, 1:4) gave BCP iodide **7d-I** (35.6 mg, 0.983 mmol, 98%) as a white solid.

R$_f$ 0.70 (EtOAc/pentane, 1:4).
m.p. 151 ° C.
IR ν_{max}/cm^{-1} (film) 2981, 1303, 1291, 1262, 1164, 1151, 1115.
^{1}H NMR (600 MHz, $CDCl_3$) δ 7.36 – 7.32 (2H, m, H8), 7.29 – 7.27 (1H, m, H9), 7.23 – 7.20 (2H, m, H7), 3.20–3.16 (2H, m, H4), 3.15–3.11 (2H, m, H5), 2.66 (6H, s, H2).
^{13}C NMR (151 MHz, $CDCl_3$) δ 137.6, 129.2, 128.6, 127.4, 59.7, 56.9, 52.5, 27.5, 1.4.
HRMS (ESI$^+$) [M + Na]$^+$ $C_{13}H_{15}O_2INaS^+$ requires 384.9730; found 384.9730.

*Note: Attempts to prepare corresponding bromide **7d-Br** under **general procedure G** were unsuccessful.*

1-Iodo-3-(isopropylsulfonyl)bicyclo[1.1.1]pentane, 7e-I

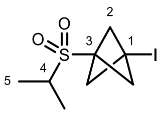

A stock solution of sulfinate salt **6e** (32.5 mg, 0.250 mmol) in H_2O (0.25 mL) was prepared. Benzyltrimethylammonium dichloroiodate (50.5 mg, 0.145 mmol), sulfinate salt **6e** (0.20 mL, 0.20 mmol, 1.0 M in H_2O), [1.1.1]propellane **1** (0.15 mL, 0.10 mmol, 0.70 M in Et_2O) in CH_2Cl_2 (0.10 mL) were subjected to the conditions of **general procedure F**. Purification by silica plug (pentane wash, followed by elution with EtOAc/pentane, 1:4) gave BCP iodide **7e-I** (21.3 mg, 0.710 mmol, 71%) as a white solid.

R$_f$ 0.43 (EtOAc/pentane, 1:4).
m.p. 110 ° C.
IR ν_{max}/cm^{-1} (film) 2979, 1300, 1288, 1204, 1161, 1116, 857, 686.
^{1}H NMR (600 MHz, $CDCl_3$) δ 3.14 (1H, hept., J = 6.9 Hz, H4), 2.72 (6H, s, H2), 1.39 (6H, d, J = 6.9 Hz, H5).
^{13}C NMR (151 MHz, $CDCl_3$) δ 60.5, 56.0, 52.8, 15.8, 1.9.
HRMS (ESI$^{+/-}$) Not found.

1-Bromo-3-(isopropylsulfonyl)bicyclo[1.1.1]pentane, 7e-Br

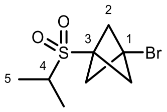

Miligram Scale: A stock solution of sulfinate salt **6e** (32.5 mg, 0.250 mmol) in H_2O (0.25 mL) was prepared. Sulfinate salt **6e** (0.20 mL, 0.20 mmol, 1.0 M in H_2O), Br_2 (0.18 mL, 0.18 mmol, 1.0 M in CH_2Cl_2), [1.1.1]propellane **1** (0.15 mL, 0.10 mmol, 0.70 M in Et_2O) and Et_3B (10 µL, 0.01 mmol, 1.0 M in hexanes) in CH_2Cl_2 (0.10 mL) were subjected to the conditions of **General Procedure G**. BCP iodide **7e-Br** (17.0 mg, 0.0672 mmol, 67%) was obtained as a white solid.

Decagram Scale: The conditions of **general procedure E** *(isolated RSO_2Br)* gave BCP bromide **7e-Br** (33.00 g, 0.13 mol, 98%) white solid.

R_f 0.57 (EtOAc/pentane, 1:4) [goofy].
m.p. 137 – 138 °C.
IR ν_{max}/cm^{-1} (film) 1283, 1210, 1173, 1160, 1108, 867.
^{1}H NMR (600 MHz, $CDCl_3$) δ 3.16 (1H, hept., *J* = 6.9 Hz, H4), 2.67 (6H, s, H2), 1.40 (6H, d, *J* = 6.9 Hz, H5).
^{13}C NMR (151 MHz, $CDCl_3$) δ 59.2, 53.0, 50.6, 34.7, 15.8.
HRMS (ESI$^+$) $[M + H]^+$ $C_8H_{17}{}^{79}BrNO_2S^+$ requires 272.0143; found 272.0136.

1-(Cyclopropylsulfonyl)-3-iodobicyclo[1.1.1]pentane, 7f-I

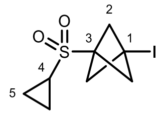

A stock solution of sulfinate salt **6f** (25.6 mg, 0.200 mmol) in H_2O (0.20 mL) was prepared. Benzyltrimethylammonium dichloroiodate (50.5 mg, 0.145 mmol), sulfinate salt **6f** (0.20 mL, 0.20 mmol, 1.0 M in H_2O), [1.1.1]propellane **1** (0.15 mL, 0.10 mmol, 0.70 M in Et_2O) in CH_2Cl_2 (0.10 mL) were subjected to the conditions of **general procedure F**. BCP iodide **7f-I** (29.4 mg, 0.099 mmol, 99%) was obtained as a white solid.

R_f 0.33 (EtOAc/pentane, 1:4).
m.p. 145 ° C.

IR ν_{max}/cm^{-1} (film) 2980, 1315, 1292, 1167, 1143, 1123, 701, 610.

^{1}H NMR (600 MHz, $CDCl_3$) δ 2.70 (6H, s, H2), 2.30 (1H, tt, J = 7.9, 4.8 Hz, H4), 1.26 – 1.17 (2H, m, H5), 1.10 – 1.00 (2H, m, H5).

^{13}C NMR (151 MHz, $CDCl_3$) δ 59.8, 56.9, 27.2, 4.5, 2.2.

HRMS (ESI$^{+/-}$) Not found.

1-Bromo-3-(cyclopropylsulfonyl)bicyclo[1.1.1]pentane, 7f-Br

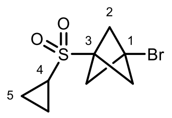

Miligram Scale: A stock solution of sulfinate salt **6f** (25.6 mg, 0.20 mmol) in H_2O (0.20 mL) was prepared. Sulfinate salt **6f** (0.20 mL, 0.20 mmol, 1.0 M in H_2O), Br_2 (0.18 mL, 0.18 mmol, 1.0 M in CH_2Cl_2), [1.1.1]propellane **1** (0.15 mL, 0.10 mmol, 0.70 M in Et_2O) and Et_3B (10 µL, 0.01 mmol, 1.0 M in hexanes) in CH_2Cl_2 (0.10 mL) were subjected to the conditions of **general procedure G**. Purification by column chromatography (SiO_2, EtOAc/Pentane, 1:9 → 2:3) gave BCP Bromide **7f-Br** (19.8 mg, 0.788 mmol, 79%) as a white solid.

Multigram Scale: The conditions of **general procedure E** *(isolated RSO_2Br)* gave BCP bromide **7f-Br** (1.28 g, 5.11 mmol, 95%) as a yellow solid.

Alternative Procedure: The conditions of **general procedure D** *(in situ RSO_2Br with NBS)* gave BCP bromide **7f-Br** (0.35 g, 1.4 mmol, 22%) as a yellow solid.

R_f 0.39 (EtOAc/pentane, 1:4) [goofy].

m.p. 155 – 156 °C.

IR ν_{max}/cm^{-1} (film) 1315, 1289, 1206, 1178, 1126, 891.

^{1}H NMR (600 MHz, $CDCl_3$) δ 2.65 (6H, s, H2), 2.32 (1H, tt, J = 8.0, 4.8 Hz, H4), 1.25–1.20 (2H, m, H5), 1.08–1.04 (2H, m, H5).

^{13}C NMR (151 MHz, $CDCl_3$) δ 58.5, 51.4, 34.9, 27.5, 4.6.

HRMS (ESI$^+$) $[M + H]^+$ $C_8H_{12}{}^{79}BrO_2S^+$ requires 252.9721; found 252.9713.

Methyl 3-((3-iodobicyclo[1.1.1]pentan-1-yl)sulfonyl)propanoate, 7g-I

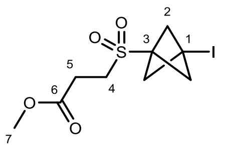

A stock solution of sulfinate salt **6g** (34.8 mg, 0.200 mmol) in H_2O (0.20 mL) was prepared. Benzyltrimethylammonium dichloroiodate (50.5 mg, 0.145 mmol), sulfinate salt **6g** (0.20 mL, 0.20 mmol, 1.0 M in H_2O), [1.1.1]propellane **1** (0.15 mL, 0.10 mmol, 0.70 M in Et_2O) in CH_2Cl_2 (0.10 mL) were subjected to the conditions of **general procedure F**. BCP iodide **7g-I** (13.3 mg, 0.0386 mmol, 39%) was obtained as a white solid.

R$_f$ 0.30 (EtOAc/pentane, 1:4)[vanilin].

m.p. 115 °C.

IR ν_{max}/cm^{-1} (film) 1738, 1311, 1203, 1167, 1120, 669.

^{1}**H NMR** (600 MHz, $CDCl_3$) δ 3.74 (3H, s, H7), 3.26 (2H, t, *J* = 7.7 Hz, H4), 2.85 (2H, t, *J* = 7.7 Hz, H5), 2.70 (6H, s, H2).

^{13}**C NMR** (151 MHz, $CDCl_3$) δ 170.8, 59.6, 56.8, 52.7, 31.1, 26.2, 1.2.

HRMS (ESI$^+$) [M + Na]$^+$ $C_9H_{13}O_4INaS^+$ requires 366.9471; found 366.9472.

Methyl 3-((3-bromobicyclo[1.1.1]pentan-1-yl)sulfonyl)propanoate, 7g-Br

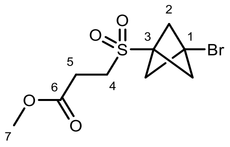

A stock solution of sulfinate salt **6g** (34.8 mg, 0.20 mmol) in H_2O (0.20 mL) was prepared. Sulfinate salt **6g** (0.20 mL, 0.20 mmol, 1.0 M in H_2O), Br_2 (0.18 mL, 0.18 mmol, 1.0 M in CH_2Cl_2), [1.1.1]propellane **1** (0.15 mL, 0.10 mmol, 0.70 M in Et_2O) and Et_3B (10 µL, 0.01 mmol, 1.0 M in hexanes) in CH_2Cl_2 (0.10 mL) were subjected to the conditions of **general procedure G**. BCP iodide **7g-Br** (21.5 mg, 0.0724 mmol, 73%) was obtained as a white solid.

R$_f$ 0.36 (EtOAc/pentane, 3:7) [Goofy].

IR ν_{max}/cm^{-1} (film) 2981, 1727, 1373, 1310, 1257, 1179, 1122.

^{1}**H NMR** (600 MHz, $CDCl_3$) δ 3.75 (3H, s, H7), 3.30 (2H, t, *J* = 7.7 Hz, H4), 2.86 (2H, t, *J* = 7.7 Hz, H5), 2.65 (6H, s, H2).

^{13}C NMR (151 MHz, $CDCl_3$) δ 170.8, 58.4, 52.7, 51.5, 46.5, 34.4, 26.2.

HRMS (ESI$^+$) $[M + H]^+$ $C_9H_{14}O_4{}^{79}BrS^+$ requires 296.9791; found 296.9788.

1-((4,4-Difluorocyclohexyl)sulfonyl)-3-iodobicyclo[1.1.1]pentane, 7h-I

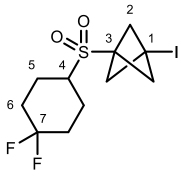

A stock solution of sulfinate salt **6h** (41.2 mg, 0.200 mmol) in H_2O (0.20 mL) was prepared. Benzyltrimethylammonium dichloroiodate (50.5 mg, 0.145 mmol), sulfinate salt **6h** (0.20 mL, 0.20 mmol, 1.0 M in H_2O), [1.1.1]propellane **1** (0.15 mL, 0.10 mmol, 0.70 M in Et_2O) in CH_2Cl_2 (0.10 mL) were subjected to the conditions of **general procedure F**. Purification by silica plug (pentane wash, followed by elution with EtOAc/pentane, 1:4) gave BCP iodide **7h-I** (31.4 mg, 0.0835 mmol, 84%) as a white solid.

R_f 0.46 (EtOAc/pentane, 1:4) [UV].

m.p. 165° C.

IR ν_{max}/cm^{-1} (film) 1301, 1205, 1162, 1100, 969, 741, 645.

^{1}H NMR (600 MHz, $CDCl_3$) δ 2.98–2.90 (1H, m, H4), 2.73 (6H, s, H2), 2.34–2.25 (2H, m, H6), 2.24–2.18 (2H, m, H5), 2.03–1.93 (2H, m, H5), 1.86–1.72 (2H, m, H6).

^{13}C NMR (151 MHz, $CDCl_3$) δ 121.6 (dd, $^1J_{CF}$ = 242.8, 240.6 Hz), 60.4, 57.9 (d, $^4J_{CF}$ = 1.1 Hz), 56.3, 32.3 (dd, $^2J_{CF}$ = 25.9, 24.7 Hz), 22.4 (dd, $^3J_{CF}$ = 9.0, 1.4 Hz), 1.4.

^{19}F NMR (377 MHz, $CDCl_3$) δ -101.6 (d, J = 242.4 Hz), – 94.7 (d, J = 242.5 Hz).

HRMS (ESI^{+-}, APCI and EI) Not Found.

1-Bromo-3-((4,4-difluorocyclohexyl)sulfonyl)bicyclo[1.1.1]pentane, 7h-Br

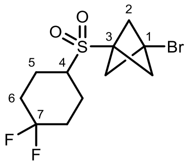

A stock solution of sulfinate salt **6h** (41.2 mg, 0.200 mmol) in H_2O (0.20 mL) was prepared. Sulfinate salt **6h** (0.20 mL, 0.20 mmol, 1.0 M in H_2O), Br_2 (0.18 mL, 0.18 mmol, 1.0 M in CH_2Cl_2), [1.1.1]propellane **1** (0.15 mL, 0.10 mmol, 0.70 M in Et_2O) and Et_3B (10 µL, 0.01 mmol,

1.0 M in hexanes) in CH_2Cl_2 (0.10 mL) were subjected to the conditions of **general procedure G**. BCP iodide **7h-Br** (32.5 mg, 0.099 mmol, 99%) was obtained as a white solid.

R_f 0.50 (EtOAc/pentane, 1:4).
m.p. 163 – 164° C.
IR ν_{max}/cm^{-1} (film) 2981, 1379, 1301, 1171, 1102, 969, 879, 742.
^{1}H NMR (600 MHz, $CDCl_3$) δ 3.00 – 2.92 (1H, m, H4), 2.68 (6H, s, H2), 2.35 – 2.25 (2H, m, H6), 2.25 – 2.18 (2H, m, H5), 2.04 – 1.94 (2H, m, H5), 1.86 – 1.73 (2H, m, H6).
^{13}C NMR (151 MHz, $CDCl_3$) δ 121.6 (dd, $^1J_{CF2}$ = 242.8, 240.6 Hz), 59.1, 58.2, 50.9, 34.4, 32.5–32.1 (m), 22.4 (dd, $^3J_{CF2}$ = 9.1, 1.5 Hz).
^{19}F NMR (377 MHz, $CDCl_3$) δ -101.60 (d, J = 242.8 Hz), -94.70 (d, J = 242.1 Hz).
HRMS ($ESI^{+/-}$, APCI) Not Found.

(1-(Bicyclo[1.1.1]pentan-1-ylsulfonyl)-3-bromobicyclo[1.1.1]pentane, 3i-I

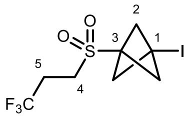

A stock solution of sulfinate salt **2i** (40.5 mg, 0.200 mmol) in DMF (0.22 mL) was prepared. Benzyltrimethylammonium dichloroiodate (50.5 mg, 0.145 mmol), sulfinate salt **2i** (0.20 mL, 0.20 mmol, 1.0 M in H_2O), [1.1.1]propellane **1** (0.11 mL, 0.10 mmol, 0.93 M in Et_2O) in CH_2Cl_2 (0.40 mL) were subjected to the conditions of **general procedure B**. Purification by column chromatography (SiO_2, EtOAc/pentane, 0:1 → 1:4) gave BCP iodide **3i-I** (10.0 mg, 0.027 mmol, 27%) as a white solid.

R_f 0.10 (hexane/EtOAc, 9:1) [faint UV, goofy].
m.p. 156 – 158 °C.
IR ν_{max}/cm^{-1} (film) 1454, 1393, 1285, 1169, 1146, 1088, 961, 870.
^{1}H NMR (400 MHz, $CDCl_3$) δ 3.16 – 3.08 (m, 0H), 2.72 (s, 1H), 2.71 – 2.58 (m, 0H).
^{19}F NMR (377 MHz, $CDCl_3$) δ -66.02 (t, J = 10.0 Hz).
^{13}C NMR (101 MHz, $CDCl_3$) δ 59.56, 56.78, 43.82 (q, J = 2.7 Hz), 29.85, 26.76 (q, J = 31.8 Hz), 0.57.
HRMS ($ESI^{+/-}$) Not found.

3-(((3-Iodobicyclo[1.1.1]pentan-1-yl)sulfonyl)methyl)-3-methyloxetane, 7j-I

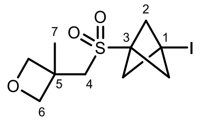

A stock solution of sulfinate salt **6j** (34.4 mg, 0.20 mmol) in DMF:H_2O (1:2, 0.20 mL) was prepared. Benzyltrimethylammonium dichloroiodate (25.3 mg, 0.0727 mmol), sulfinate salt **6j** (0.10 mL, 0.10 mmol, 1.0 M in H_2O), [1.1.1]propellane **1** (0.08 mL, 50 µmol, 0.70 M in Et_2O) in CH_2Cl_2 (0.10 mL) were subjected to the conditions of **general procedure F**. BCP iodide **7j-I** (17 mg, 0.0500 mmol, 99%) was obtained as a white solid.

R_f 0.21 (EtOAc/pentane, 2:3).
m.p. 158 ° C.
IR ν_{max}/cm^{-1} (film) 1292, 1272, 1206, 1165, 1117, 1083, 920, 870.
^{1}H NMR (600 MHz, $CDCl_3$) δ 4.64 (2H, d, *J* = 6.4 Hz, H6), 4.46 (2H, d, *J* = 6.4 Hz, H6), 3.31 (2H, s, H4), 2.69 (6H, s, H2), 1.65 (3H, s, H7).
^{13}C NMR (151 MHz, $CDCl_3$) δ 82.4, 59.4, 57.9, 57.1, 37.8, 23.5, 1.2.
HRMS (EI^+) $[M]^+$ $C_{10}H_{15}O_3IS^+$ requires 341.9787; found 341.0202.

3-(((3-Bromobicyclo[1.1.1]pentan-1-yl)sulfonyl)methyl)-3-methyloxetane, 7j-Br

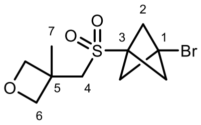

A stock solution of sulfinate salt **6j** (34.4 mg, 0.20 mmol) in DMF:H_2O (1:2, 0.20 mL) was prepared. Sulfinate salt **6j** (0.10 mL, 0.10 mmol, 1.0 M in H_2O), Br_2 (0.09 mL, 0.09 mmol, 1.0 M in CH_2Cl_2), [1.1.1]propellane **1** (0.08 mL, 50 µmol, 0.70 M in Et_2O) and Et_3B (5 µL, 0.01 mmol, 1.0 M in hexanes) in CH_2Cl_2 (0.05 mL) were subjected to the conditions of **general procedure G**. BCP iodide **7j-Br** (14.7 mg, 0.0498 mmol, 99%) was obtained as a white solid.

R_f 0.27 (EtOAc/pentane, 1:4).
m.p. 125 – 128 °C.
IR ν_{max}/cm^{-1} (film) 2957, 1303, 1284, 1177, 1122, 978.
^{1}H NMR (600 MHz, $CDCl_3$) δ 4.64 (2H, d, *J* = 6.4 Hz, H6), 4.47 (2H, d, *J* = 6.4 Hz, H6), 3.33 (2H, s, H4), 2.64 (6H, s, H2), 1.66 (3H, s, H7).

^{13}C NMR (151 MHz, $CDCl_3$) δ 82.4, 58.1, 57.4, 52.6, 37.8, 34.5, 23.5.

HRMS (ESI^+) $[M + H]^+$ $C_{10}H_{16}O_3{}^{79}BrS^+$ requires 294.9998; found 294.9997.

3-((3-Iodobicyclo[1.1.1]pentan-1-yl)sulfonyl)tetrahydrofuran, 7k-I

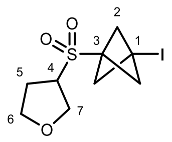

A stock solution of sulfinate salt **6k** (31.6 mg, 0.200 mmol) in H_2O (0.20 mL) was prepared. Benzyltrimethylammonium dichloroiodate (50.5 mg, 0.145 mmol), sulfinate salt **6k** (0.20 mL, 0.20 mmol, 1.0 M in H_2O), [1.1.1]propellane **1** (0.15 mL, 0.10 mmol, 0.70 M in Et_2O) in CH_2Cl_2 (0.10 mL) were subjected to the conditions of **general procedure F**. Purification by silica plug (pentane wash, followed by elution with EtOAc/pentane, 1:4) gave BCP iodide **7k-I** (23.4 mg, 0.0713 mmol, 71%) as a white solid.

R_f 0.13 (EtOAc/pentane, 1:4)[UV, goofy].

m.p. 139 – 142 ° C.

IR ν_{max}/cm^{-1} (film) 1292, 1272, 1206, 1165, 1117, 1083, 920, 870.

^{1}H NMR (600 MHz, $CDCl_3$) δ 4.16 (1H, dd, *J* = 10.1, 5.4 Hz, H7), 4.03 (1H, dd, *J* = 10.1, 8.1 Hz, H7), 3.97 (1H, td, *J* = 8.2, 5.9 Hz, H6), 3.82 (1H, dt, *J* = 8.7, 6.8 Hz, H6), 3.65 (1H, ddt, *J* = 9.8, 8.1, 5.4 Hz, H4), 2.74–2.67 (6H, m, H2), 2.36 (1H, ddt, *J* = 13.4, 7.6, 6.0 Hz, H5), 2.26 (1H, dddd, *J* = 13.4, 9.8, 7.3, 5.9 Hz, H5).

^{13}C NMR (151 MHz, $CDCl_3$) δ 68.5, 67.2, 60.0, 59.9, 56.1, 27.4, 1.6.

HRMS ($ESI^{+/-}$, APCI and EI) Not Found.

3-((3-Bromobicyclo[1.1.1]pentan-1-yl)sulfonyl)tetrahydrofuran, 7k-Br

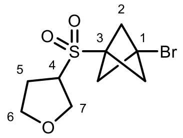

A stock solution of sulfinate salt **6k** (31.6 mg, 1.20 mmol) in H_2O (0.20 mL) was prepared. Sulfinate salt **6k** (0.20 mL, 0.20 mmol, 1.0 M in H_2O), Br_2 (0.18 mL, 0.18 mmol, 1.0 M in CH_2Cl_2), [1.1.1]propellane **1** (0.15 mL, 0.10 mmol, 0.70 M in Et_2O) and Et_3B (10 µL, 0.01 mmol,

1.0 M in hexanes) in CH_2Cl_2 (0.10 mL) were subjected to the conditions of **general procedure G**. BCP bromide **7k-Br** (26.9 mg, 0.0957 mmol, 96%) was obtained as a white solid.

R$_f$ 0.13 (EtOAc/pentane, 1:4) [goofy].
m.p. 115 – 118 °C.
IR ν_{max}/cm^{-1} (film) 2981, 1382, 1312, 1174, 1081, 876, 668.
^{1}**H NMR** (600 MHz, $CDCl_3$) δ 4.17 (1H, dd, *J* = 10.2, 5.5 Hz, H7), 4.04 (1H, dd, *J* = 10.2, 8.1 Hz, H7), 3.97 (1H, ddd, *J* = 8.8, 7.8, 6.0 Hz, H6), 3.83 (1H, dt, *J* = 8.8, 6.9 Hz, H6), 3.67 (1H, ddt, *J* = 9.8, 8.1, 5.5 Hz, H4), 2.68 – 2.63 (6H, m, H2), 2.37 (1H, ddt, *J* = 13.4, 7.6, 6.0 Hz, H5), 2.27 (1H, dddd, *J* = 13.4, 9.8, 7.3, 6.0 Hz, H5).
^{13}**C NMR** (151 MHz, $CDCl_3$) δ 68.5, 67.2, 60.3, 58.6, 50.7, 34.7, 27.5.
HRMS (ESI$^{+/-}$, APCI) Not found.

(1-(Bicyclo[1.1.1]pentan-1-ylsulfonyl)-3-bromobicyclo[1.1.1]pentane, 7l-Br

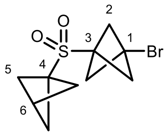

To a solution of PPh_3 (1.60 g, 6.16 mmol, 0.95 equiv.) in dry CH_3CN (50 mL) was slowly added bromine (0.98 g, 0.3 mL, 6.10 mmol, 0.95 equiv.) under Ar atmosphere at 0 °C. The mixture was stirred at 0 °C for 30 min, and sodium bicyclo[1.1.1]pentane-1-sulfinate **6l** (1.00 g, 6.4 mmol, 1.00 equiv.) was added in one portion. The resulting mixture was stirred at 0 °C for 15 min, and a solution of [1.1.1]propellane **1** (14 mL, 9.7 mmol, 0.7 M in Et_2O, 1.50 equiv.) was added. The mixture was stirred at room temperature for 15 h. The solvent was removed under reduced pressure. The final product was purified by column chromatography (SiO_2, hexane/EtOAc, 7:3) to give BCP bromide **7l-Br** (1.00 g, 3.65 mmol, 57%) as a white powder.

R$_f$ 0.45 (hexane/EtOAc, 7:3).
m.p. 181 – 182 °C.
^{1}**H NMR** (500 MHz, $CDCl_3$) δ 2.80 (1H, s, H6), 2.64 (6H, s, H2), 2.28 (6H, s, H5).
^{13}**C NMR** (126 MHz, $CDCl_3$) δ 77.2, 59.0, 53.6, 51.8, 50.1, 35.0, 28.1.
HRMS (ESI$^+$) $[M + H]^+$ $C_{10}H_{14}{}^{81}BrO_2S^+$ requires 278.9877; found 278.9860.

2.5 Sulfonyl Cyclobutyl Halides

1-Iodo-*N*,*N*-diisopropyl-3-tosylcyclobutane-1-carboxamide, 10a-I

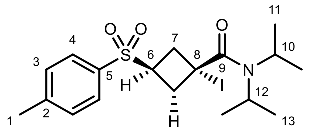

A stock solution of sulfinate salt **2a** (890 mg, 1.20 mmol) in H_2O (3.00 mL) was prepared. Benzyltrimethylammonium dichloroiodate (50.5 mg, 0.12 mmol), sulfinate salt **2a** (0.20 mL, 0.20 mmol, 1.0 M in H_2O), BCB **9a** (0.15 mL, 0.10 mmol, 0.10 M in Et_2O) in CH_2Cl_2 (0.10 mL) were subjected to the conditions of **general procedure H**. BCP iodide **10a-I** (36.8 mg, 0.159 mmol, 80%, 2.5:1 dr) was obtained as a white solid.

R$_f$ 0.40 (EtOAc/pentane, 1:4) [UV].

IR v_{max}/cm^{-1} (film) 2971, 1633, 1441, 1371, 1329, 1148, 1087, 732, 680.

^{1}H NMR (600 MHz, $CDCl_3$) δ 7.78 – 7.69 (2.4 H, m, H4, H4′), 7.39 – 7.32 (2.4H, m, H3, H3′), 4.11 (1H, tt, *J* = 9.5, 7.6 Hz, H6), 3.88 (0.4H, hept., *J* = 6.5 Hz, H12′), 3.77 (1H, hept., *J* = 6.6 Hz, H12), 3.70 (0.4H, pent., *J* = 8.6 Hz, H6′), 3.38 – 3.24 (1.4H, m, H10, H10′), 3.28 – 3.14 (2.8H, m, H7b, H7a′, 7b′), 2.56 (2H, br. s, H7a), 2.44 (4.2H, s, H1, H1′), 1.38 (6H, d, *J* = 6.8 Hz, H11), 1.35 (2.4H, d, *J* = 6.7 Hz, H11′), 1.26 – 1.20 (8.4, m, H13, H13′).

^{13}C NMR (151 MHz, $CDCl_3$) δ 169.1, 168.4, 145.3, 134.6, 134.4, 130.2, 128.5, 128.5, 55.1, 51.6, 51.0, 50.0, 46.9, 46.7, 40.8 (br.), 29.1, 21.9, 21.8, 19.9 (br.).

HRMS (ESI$^+$) [M + H]$^+$ $C_{18}H_{27}O_3NIS^+$ requires 464.0751; found 464.0748.

Numbers denoted with a prime in blue correspond to the minor diastereisomer. Diastereisomer ratio calculated from the analysis of the ^{1}H NMR of the crude sample. Recrystallisation from CH_2Cl_2/pentane at -20 °C gave a pure sample of the major diastereoisomer:

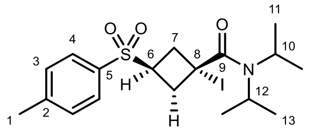

^{1}H NMR (600 MHz, $CDCl_3$) δ 7.75 (2H, br. d, *J* = 8.2 Hz, H4), 7.36 (2H, br. d, *J* = 8.5 Hz, H3), 4.12 (1H, tt, *J* = 9.3, 7.6 Hz, H6), 3.78 (1H, hept., *J* = 6.7 Hz, H12), 3.32 (1H, hept., *J* = 6.9 Hz, H10),

3.21 (2H, br. s, H7b), 2.56 (2H, br. s, H7a), 2.45 (3H, s, H1), 1.39 (6H, d, *J* = 6.9 Hz, H11), 1.24 (6H, d, *J* = 6.7 Hz, H13).

^{13}C NMR (151 MHz, $CDCl_3$) δ 169.1, 145.3, 134.7, 130.2, 128.5, 51.7, 50.0, 46.7, 40.9, 29.1, 21.8, 19.9.

Assignment of major diasteromer was based on analogy of the ^{1}H NMR to **10b-l.**

1-Bromo-*N,N*-diisopropyl-3-tosylcyclobutane-1-carboxamide, 10a-Br

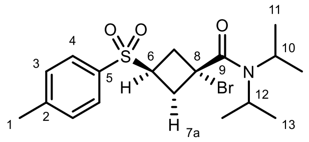

A stock solution of sulfinate salt **2a** (890 mg, 1.20 mmol) in H_2O (3.00 mL) was prepared. A solution of sodium sulfinate salt **2a** (0.25 mL, 0.25 mmol, 1.0 M in H_2O) was added to a 3 mL vial equipped with a stir bar and septum at -5 °C. CH_2Cl_2 (0.10 mL) was added. A solution of Br_2 (0.18 mL, 0.18 mmol, 1.0 M in CH_2Cl_2) was added and the mixture was stirred vigerously for 2 min, until disappearance of the brown bromine colour to a colourless solution. A solution of BCB **9a** (18.1 mg, 0.20 mL, 0.10 mmol, 0.5 M in CH_2Cl_2) and then Et_3B (10 µL, 0.01 mmol, 1.0 M in hexanes) were added sequentially. The vial was capped, sonicated (5 s), and stirred vigerously at room temperature for 2 h. The reaction was quenched with $Na_2S_2O_3$ (sat. aq., 0.50 mL). The biphasic mixture was poured onto H_2O (1 mL), extracted with CH_2Cl_2 (3 × 2 mL), then the combined organic phases were dried (Mg_2SO_4), filtered and concentrated *in vacuo*. Purification by column chromatography (SiO_2, EtOAc/ pentane 1:19 → 1:4) gave bromide **10a-Br** (29.8 mg, 0.0718 mmol, 72%, 2.3:1 dr) as a white solid.

R_f 0.39 (EtOAc/pentane, 1:4)[UV, ninhydrin].

m.p. 145 ° C.

IR ν_{max}/cm^{-1} (film) 1632, 1593, 1339, 1149, 1076, 651.

^{1}H NMR (600 MHz, $CDCl_3$) δ 7.78 – 7.72 (2.5H, m, H4, H4'), 7.39 – 7.33 (2.5H, m, H3, H3'), 4.07 (1H, pent., *J* = 8.5 Hz, H6), 3.95 (0.25H, hept., *J* = 6.6 Hz, H10'), 3.84 (1H, hept., *J* = 6.6 Hz, H12), 3.57 (0.25H, pent., *J* = 8.6 Hz, H6'), 3.39 – 3.30 (3.5H, m, H10, H12', H7b), 3.29 – 3.22 (0.50H, m, H7b'), 3.17 (0.50H, dd, *J* = 13.3, 9.0 Hz, H7a'), 2.63 (2H, br. s, H7a), 2.45 (3.8H, s, H1), 1.39

(6H, d, J = 6.8 Hz, H11), 1.36 (1.5H, obsc. d, J = 6.6 Hz, H11′), 1.22 (7.5H, d, J = 6.6 Hz, H13, H13′).

^{13}C NMR (151 MHz, $CDCl_3$) δ 167.3, 166.7, 145.3, 134.6, 130.2, 128.5, 77.2, 53.1, 52.7, 51.5, 50.3, 49.5, 48.0, 46.9, 46.7, 38.6, 38.5, 21.8, 20.2, 20.1, 20.1, 20.0.

HRMS ($ESI^{+/-}$) Not found.

Numbers denoted with a prime in blue correspond to the minor diastereisomer. Diastereisomer ratio calculated from the analysis of the ^{1}H NMR of the crude sample. Assignment of the major diastereisomer was assigned based on analogy of ^{1}H NMR to **10a-I**.

1-((3-Iodo-3-(phenylsulfonyl)cyclobutyl)sulfonyl)-4-methylbenzene, 10b-I

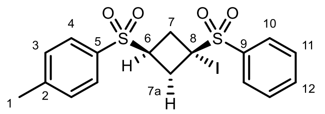

A stock solution of sulfinate salt **2a** (890 mg, 1.20 mmol) in H_2O (3.00 mL) was prepared. Benzyltrimethylammonium dichloroiodate (50.5 mg, 0.14 mmol), sulfinate salt **2a** (0.25 mL, 0.25 mmol, 1.0 M in H_2O), BCB **9b** (19.4 mg, 0.20 mL, 0.10 mmol, 0.50 M in CH_2Cl_2), Et_3B (10 µL, 0.10 mmol, 1.0 M in hexane) in CH_2Cl_2 (0.10 mL) were subjected to the conditions of **general procedure H**. Purification by column chromatography (SiO_2, EtOAc/ pentane 1:9 → 1:4) gave iodide **10b-I** (31.3 mg, 0.66 mmol, 66%, 2.1:1 dr) as a white solid.

R_f 0.19 (EtOAc/pentane, 1:4)[UV].

m.p. 137 ° C.

IR ν_{max}/cm^{-1} (film) 1384, 1317, 1148, 1085, 914, 687, 647.

^{1}H NMR (600 MHz, $CDCl_3$) δ 8.05 – 8.01 (0.6H, m, H10′), 8.00 – 7.96 (2H, m, H10), 7.77 – 7.70 (4.8H, m, H4, H4′, H12, H12′), 7.65 – 7.57 (2.6H, m, H11, H11′), 7.41 – 7.36 (2.6H, m, H3, H3′), 4.26 (0.6H, pent., J = 8.7 Hz, H6′), 4.09 (1H, tt, J = 9.6, 7.7 Hz, H6), 3.61 – 3.53 (2H, m, H7b), 3.49 – 3.42 (1.2H, m, H7a′), 3.41 – 3.34 (1.2H, m, H7b′), 2.57 – 2.50 (2H, m, H7a), 2.47 (3.6H, s, H1, H1′).

^{13}C NMR (151 MHz, $CDCl_3$) δ 145.7, 145.7, 135.1, 135.0, 134.2, 133.5, 131.6, 131.0, 130.4, 130.4, 129.4, 129.1, 128.6, 128.6, 55.7, 50.9, 42.4, 39.0, 39.0, 37.9, 21.9, 21.9.

HRMS (ESI^{+}) $[M + H]^+$ $C_{17}H_{18}O_4IS_2$ requires 476.9686; found 476.9686.

Numbers denoted with a prime in blue correspond to the minor diastereisomer. Diastereisomer ratio calculated from the analysis of the ^{1}H NMR of the crude sample. Assignment of the major diastereisomer was based on NOESY correllations.

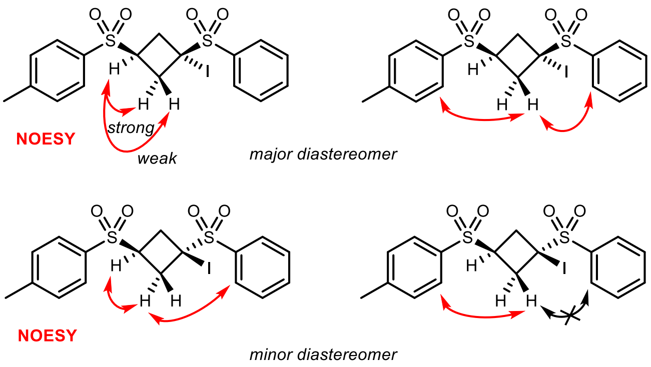

1-((3-(*t*-Butylsulfonyl)-3-iodocyclobutyl)sulfonyl)-4-methylbenzene, 10c-I

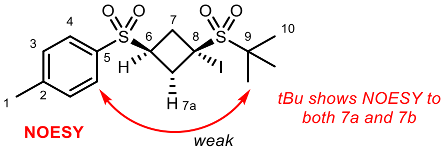

A stock solution of sulfinate salt **2a** (890 mg, 1.20 mmol) in H_2O (3.00 mL) was prepared. Benzyltrimethylammonium dichloroiodate (50.5 mg, 0.14 mmol), sulfinate salt **2a** (0.25 mL, 0.25 mmol, 1.0 M in H_2O), BCB **9c** (17.4 mg, 0.20 mL, 0.10 mmol, 0.50 M in CH_2Cl_2), Et_3B (10 µL, 0.10 mmol, 1.0 M in hexane) in CH_2Cl_2 (0.10 mL) were subjected to the conditions of **general procedure H**. The dr of the crude reaction mixture was 10:1. Purification by column chromatography (SiO_2, EtOAc/pentane, 1:9 → 1:4) gave iodide **10c-I** (12.7 mg, 0.0279 mmol, 28%, 20:1 dr) as a white solid.

R$_f$ 0.13 (EtOAc/pentane, 1:4).

m.p. 171 – 180 °C.

IR ν_{max}/cm^{-1} (film) 1596, 1320, 1395, 1148, 1114, 1086, 712, 695.

^{1}H NMR (600 MHz, $CDCl_3$) δ 7.76 (2H, d, *J* = 8.3 Hz, H4), 7.38 (2H, d, *J* = 8.0 Hz, H5), 4.21 (1H, tt, *J* = 9.7, 7.6 Hz, H6), 3.83 – 3.75 (2H, m, H7b), 2.61 (2H, ddt, *J* = 10.9, 7.6, 2.6 Hz, H7a), 2.46 (3H, s, H1), 1.62 (9H, s, H10).

^{13}C NMR (151 MHz, $CDCl_3$) δ 145.6, 134.3, 130.4, 128.6, 65.6, 52.1, 41.1, 36.1, 26.4, 21.9.

HRMS (ESI^{+}) [M + Na]$^{+}$ $C_{15}H_{21}O_4INaS_2^{+}$ requires 478.9818; found 478.9817.

Diastereisomer ratio calculated from the analysis of the ^{1}H NMR of the crude sample. Assignment of the major diastereisomer was based on analogy of ^{1}H NMR data with **10b-I** and weak NOESY correlations.

1-((3-Iodo-3-(phenylsulfonyl)cyclobutyl)sulfonyl)-4-methylbenzene, 10d-I

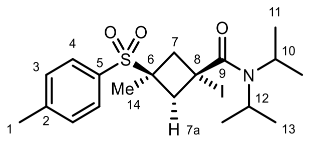

A stock solution of sulfinate salt **2a** (890 mg, 1.20 mmol) in H_2O (3.00 mL) was prepared. Benzyltrimethylammonium dichloroiodate (50.5 mg, 0.14 mmol), sulfinate salt **2a** (0.25 mL, 0.25 mmol, 1.0 M in H_2O), BCB **9d** (19.5 mg, 0.20 mL, 0.10 mmol, 0.50 M in CH_2Cl_2), Et_3B (10 µL, 0.10 mmol, 1.0 M in hexane) in CH_2Cl_2 (0.10 mL) were subjected to the conditions of **general procedure H**. Purification by column chromatography (SiO_2, EtOAc/pentane, 1:9 → 1:4) gave iodide **10d-I** (44.9 mg, 0.94 mmol, 94%, in 3.3:1 dr) as a white solid.

R_f 0.31 (EtOAc/pentane, 1:4).
m.p. 144 – 146° C.
IR ν_{max}/cm^{-1} (film) 1634, 1596, 1416, 1372, 1329, 1301, 1138, 1081, 732, 647.
^{1}H NMR (600 MHz, $CDCl_3$) δ 7.74 (3.2H, d, J = 8.6 Hz, H4, H4’), 7.43 (0.7H, d, J = 7.9 Hz, H3’), 7.34 (2H, d, J = 7.9 Hz, H3), 4.01 (1H, br. m, H7b), 3.87 (0.7H, hept., J = 6.5 Hz, H12’), 3.75 (1H, hept., J = 6.6 Hz, H12), 3.69 – 3.37 (2.4H, br. m, H7b, 7a’, 7b), 3.33 (0.7H, hept. J = 6.9 Hz, H10’), 3.29 (1H, hept., J = 6.8 Hz, H10), 2.56 (2H, br. s, H7a), 2.46 (2.1H, s, H1’), 2.43 (3H, s, H1), 1.90 (3H, s, H14), 1.37 (10.2H, d, J = 6.8 Hz, H11, H11’), 1.28 (2.1H, s, H14’), 1.25 (10.2H, d, J = 6.7 Hz, H13, H13’).
^{13}C NMR (151 MHz, $CDCl_3$) δ 169.6, 169.5, 145.2, 145.2, 131.9, 131.8, 130.2, 130.0, 129.9, 129.8, 59.8, 57.2, 50.9, 50.2, 46.8, 46.7, 46.6 (br.), 45.4 (br.), 23.4, 22.6, 21.8, 21.8, 21.1, 20.9, 20.2 (br.), 19.4 (br.).
HRMS (ESI$^+$) $[M + H]^+$ $C_{19}H_{29}O_3NIS^+$ requires 478.0907; found 478.0907.

Numbers denoted with a prime in blue correspond to the minor diastereisomer. Diastereisomer ratio calculated from the analysis of the ^{1}H NMR of the crude sample.

Diastereoisomer assignment based on comparison of ^{1}H NMR spectra of **10a-I** and X-Ray crystal structure. NOESY correlations were inconclusive.

2.6 Functionalisation Products

2-((3-Bromobicyclo[1.1.1]pentan-1-yl)sulfonyl)acetic acid, 8

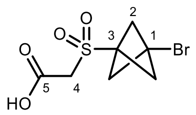

1-Bromo-3-(methylsulfonyl)bicyclo[1.1.1]pentane **7a-Br** (30.00 g, 0.133 mol, 1.00 equiv.) was dissolved in dry Et_2O (1000 mL) and cooled to -(85 – 90) °C. A solution of *n*-BuLi (2.5M in hexane, 0.16 mol, 65 mL, 1.20 equiv.) was added dropwise under Ar at -(85 – 90) °C. The resulting solution was stirred at -85 °C for 30 min and poured on dry CO_2 (500 g). The mixture was allowed to warm to room temperature and H_2O (1000 mL) was added. The solution was extracted with MTBE (3 × 200 mL). The aqueous layer was acidified with aq. 1M HCl (pH = ca. 3), and then extracted with EtOAc (3 × 300 mL). The combined organic layers were dried over Na_2SO_4 and concentrated under reduced pressure. The residue was triturated (hexane/MTBE, 7:3, ca. 250 mL), filtered and dried under reduced pressure (2.0 – 0.1 mmHg, 40 °C) to give BCP bromide **8** (25.80 g, 0.095 mol, 72%) as a beige solid.

m.p. 143 – 144 °C.
^{1}H NMR (500 MHz, $CDCl_3$) δ 4.03 (2H, s, 2H), 2.72 (6H, s, H2).
^{13}C NMR (126 MHz, $CDCl_3$) δ 164.7, 59.0, 56.4, 52.7, 34.2.
HRMS (ESI^+) $[M + Na]^+$ $C_7H_9BrNaO_4S^+$ requires 292.9282; found 292.9275.

Methyl-2-(bis(tert-butoxycarbonyl)amino)-3-(3-tosylbicyclo[1.1.1]pentan-1-yl)propanoate, 11

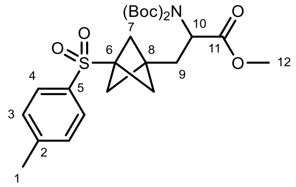

According to a literature procedure.[4] A flame dried vial was charged with BCP iodide **3a-I** (52.1 mg, 0.15 mmol), methyl 2-(bis(tert-butoxycarbonyl)amino)acrylate (271 mg, 0.90 mmol), $Ir[dF(CF)_3ppy]_2(dtbbpy)PF_6$ (4.2 mg, 2.5 mol%) and anhydrous Na_2CO_3 (32 mg, 0.30 mmol). The vial was then fitted with a PTFE septum, evacuated, and placed under an Ar atmosphere. The solids were dissolved in $MeOH/H_2O$ (9:1, 1.0 mL, 0.15 M), then $(Me_3Si)_3SiH$ (93 µL, 0.30 mmol) was added. The vial was then degassed by freeze-pump-thaw cycles (× 3), sonicated for 10

seconds, and then irradiated with Blue LEDs for 24 h with rapid stirring. The reaction mixture was poured onto H_2O (5 mL) and extracted with CH_2Cl_2 (3 × 5 mL). The combined organic phases were dried (Mg_2SO_4), filtered and concentrated *in vacuo*. Purification by column chromatography (SiO_2, EtOAc/pentane, 1:19 → 1:4) gave the compound **11** (79.5 mg, 0.152 mmol, 76%) as a white foam.

Multi-Component Procedure: Methyl 2-(bis(tert-butoxycarbonyl)amino)acrylate (271 mg, 0.90 mmol), Ir[dF(CF)$_3$ppy]$_2$(dtbbpy)PF_6 (4.2 mg, 2.5 mol%), anhydrous Na_2CO_3 (32 mg, 0.30 mmol) and anhydrous MeOH (1.0 mL) were added to a vial fitted with PTFE septum. The reaction mixture was sonicated (30 sec), then degassed with N_2 for 10 min. The vial was cooled to 0 °C and then [1.1.1]propellane **1** (0.21 mL, 0.20 mmol, 0.93 M in Et_2O) was then added. In a separate vial, $BnNMe_3ICl$ (101 mg, 0.290 mmol) and degassed CH_2Cl_2 (1.0 mL) were added at 0 °C; a solution of **2a** (0.5 mL, 0.25 mmol, 1.0 M in H_2O) was added, the vial was stirred for 2 min and then the mixture was transferred to the first vial by cannular. The reaction vial was stirred at 0 °C for 2 min, then $(Me_3Si)_3SiH$ (93 µL, 0.30 mmol) was added at room temperature. The vial was then irradiated with Blue LEDs for 18 h with rapid stirring. The reaction mixture was poured onto H_2O (5 mL) and extracted with CH_2Cl_2 (3 × 5 mL). The combined organic phases were dried (Mg_2SO_4), filtered and concentrated *in vacuo*. Purification by column chromatography (SiO_2, EtOAc/pentane, 1:19 → 1:4) gave the compound **11** (76.7 mg, 0.147 mmol, 73%) as a white solid.

R$_f$ 0.25 (EtOAc/pentane, 1:9).
IR ν_{max}/cm^{-1} (film) 2981, 1747, 1700, 1368, 1312, 1149, 1128, 732, 667.
^{1}H NMR (600 MHz, $CDCl_3$) δ 7.70 (2H, d, *J* = 8.1 Hz, H4), 7.32 (2H, d, *J* = 8.1 Hz, H3), 4.82 (1H, dd, *J* = 9.7, 4.7 Hz, H10), 3.67 (3H, s, H12), 2.43 (3H, s, H1), 2.35 (1H, dd, *J* = 15.3, 4.7 Hz, H9), 2.12 (1H, dd, *J* = 15.3, 9.7 Hz, H9), 1.95 – 1.88 (6H, m, H7), 1.43 (18H, s, Boc).
^{13}C NMR (151 MHz, $CDCl_3$) δ 170.9, 152.0, 144.6, 134.1, 129.8, 128.8, 83.6, 56.0, 52.5, 51.6, 51.1, 37.5, 31.1, 28.1, 21.7.
HRMS (ESI$^+$) [M + Na]$^+$ $C_{26}H_{37}O_8NNaS^+$ requires 546.2132; found 546.2127.

2.7 Miscellaneous Compounds

1,3-Diiodobicyclo[1.1.1]pentane, S1

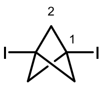

BCP di-iodide **S1** was observed during reaction optimisation and data is included here for reference.

^{1}H NMR (400 MHz, $CDCl_3$) δ 2.67 (6H, s, H2).
^{13}C NMR (101 MHz, $CDCl_3$) δ 68.1. 1.0.
Data in agreement with literature.[5]

1-Methyl-4-((3-methylenecyclobutyl)sulfonyl)benzene, S2

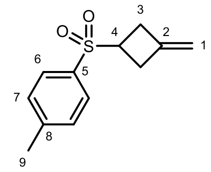

exo-Methylene cyclobutane **S2** was observed during reaction optimisation and NMR data is included here for reference.

^{1}H NMR (400 MHz, $CDCl_3$) δ 7.71 (2H, d, *J* = 8.3 Hz, H6), 7.40 – 7.32 (2H, m, H7), 4.85 (2H, tt, *J* = 2.8, 2.0 Hz, H1), 4.37 (1H, p, *J* = 6.8 Hz, H4), 2.96 (2H, dddd, *J* = 15.4, 6.8, 4.1, 2.0 Hz, H3), 2.73 – 2.61 (2H, m, H3), 2.46 (3H, s, H9).
^{13}C NMR (101 MHz, $CDCl_3$) δ 145.3, 129.9, 128.6, 125.1, 107.0, 63.5, 43.2, 43.2, 21.6.

Methyl 3-((5,5-dimethyl-2,4-dioxoimidazolidin-1-yl)sulfonyl)propanoate, S3

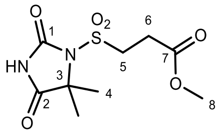

During the optimisation studies for the formation of alkyl sulfone BCP halides, compound **S3** was isolated as a by-product. DIH (78.4 mg, 0.20 mmol), sulfinate salt **6i** (0.50 mL, 0.50 mmol, 1.0 M in H_2O), [1.1.1]propellane **1** (0.29 mL, 0.20 mmol, 0.69 M in Et_2O) in CH_2Cl_2 (0.80 mL) were subjected to the conditions of **general procedure A.**

IR ν_{max}/cm^{-1} (film) 3422, 3307, 1741, 1655, 1382, 1366, 1194, 1167, 1129, 1052, 730.

^{1}H NMR (400 MHz, $CDCl_3$) δ 6.29 (1H, s, NH), 3.83 (2H, t, *J* = 6.8 Hz, H5), 3.71 (3H, s, H8), 2.98 (t, *J* = 6.8 Hz, H6), 1.52 (6H, s, H4).

^{13}C NMR (101 MHz, $CDCl_3$) δ 171.3, 170.5, 52.7, 49.9, 34.3, 27.9, 25.3.

HRMS (ESI$^+$) $[M + Na]^+$ $C_9H_{14}O_6N_2NaS^+$ requires 301.0465; found 301.0465.

1-Phenylsulfonyl)bicyclo[1.1.1]pentane, S4

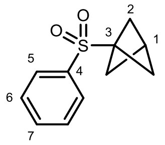

1-Iodo-3-(phenylsulfonyl)bicyclo[1.1.1]pentane **3b-I** (3.39 g, 10.00 mmol, 1.00 equiv.) was dissolved in dry toluene (100 mL), then Ar was bubbled through the reaction mixture. Tributyltin hydride (8.80 g, 8.2 mL, 30.00 mmol, 3.00 equiv.) was added in one portion, and then AIBN (0.16 g, 0.10 mmol, 0.10 equiv.) was added in one portion. The mixture was stirred at 115 °C for 15 h. After that, the mixture was cooled to room temperature, and CCl_4 (14 g, 9 mL, 90.00 mmol, 9.00 equiv.) and AIBN (0.16 g, 0.10 mmol, 0.10 equiv.) were added. The resulting mixture was stirred at 115 °C for 15 h. The mixture was cooled to room temperature, concentrated under reduced pressure (30 – 15 mmHg, 35 °C). The residue was purified by column chromatography (SiO_2, hexane/EtOAc, 7:3) to give the title compound (1 g, 0.48 mmol, 48%) as a light oil.

R*f* 0.43 (hexane/EtOAc, 7:3).

^{1}H NMR (500 MHz, $CDCl_3$) δ 7.85 (2H, d, *J* = 7.2 Hz, H5), 7.65 (1H, t, *J* = 7.4 Hz, H7), 7.56 (2H, t, *J* = 7.7 Hz, H6), 2.72 (1H, s, H1), 2.07 (6H, s, H2).

^{13}C NMR (126 MHz, $CDCl_3$) δ 137.0, 133.7, 129.2, 128.8, 55.2, 50.5, 26.9.

HRMS (ESI$^+$) $[M + H]^+$ $C_{11}H_{13}O_2S^+$ requires 209.0636; found 209.0626.

3-(*tert*-Butyl)bicyclo[1.1.1]pentane-1-carboxylic acid, S5

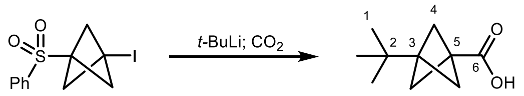

1-Iodo-3-(phenylsulfonyl)bicyclo[1.1.1]pentane **3b-I** (2.00 g, 6.00 mmol, 1.00 equiv.) was dissolved in anhydrous Et_2O (100 mL) and cooled to -85 – 90 °C under an Ar atmosphere. A solution of *t*-BuLi (1.9 M in pentane, 12.50 mmol, 6.6 mL, 2.10 equiv.) was added dropwise, and the mixture was stirred at -78 °C for 20 min. After 20 min, the solution was poured onto dry CO_2 (~ 100 g), and the reaction was allowed to warm to room temperature. H_2O (100 mL) was added, and the mixture was extracted with MTBE (3 × 50 mL). The organic phase was concentrated under reduced pressure (~30 – 15 mmHg, 35 – 50 °C) to afford the almost pure compound (0.57 g, 1.7 mmol) which was identified by ^1H NMR as the starting iodide **3b-I**. The aqueous layer was acidified with 1 M HCl to pH ~ 2, and then extracted with MTBE (3 × 50 mL). The combined organic layers were dried over Na_2SO_4 and concentrated under reduced pressure (~30 – 15 mmHg, 35 – 50 °C) to afford crude product (0.8 g). One of the main products obtained was identified as an 3-(*tert*-butyl)bicyclo[1.1.1]pentane-1-carboxylic acid **S5** (purity 33.7% by GCMS, yield by GCMS ~ 27%).

^{1}H NMR (500 MHz, $CDCl_3$) δ 10.28 (br s, 1H), 1.84 (s, 6H), 0.83 (s, 9H).
^{13}C NMR (126 MHz, $CDCl_3$) δ 176.8, 48.3, 48.1, 35.4, 29.4, 25.8.

*Direct synthesis of pure acid **S5**:*

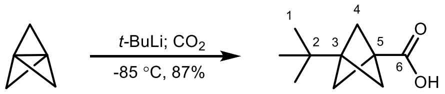

A solution of *t*-BuLi (250 mL, 0.45 mol, 1.10 equiv, 1.9 M in pentane) was added to a solution of [1.1.1]propellane **1** (400 mL, 0.43 mol, 1.00 equiv, ~1.1 M in Et_2O; prepared with PhLi (the reaction does not work if MeLi is used instead)) dropwise at -85 °C under Ar. The resulting mixture was stirred at -85 °C for 30 min. After 30 min, the solution was poured on dry CO_2 (~ 1000 g), and the reaction was allowed to warm to room temperature. H_2O (1000 mL) was added, and the mixture was extracted with MTBE (3 × 500 mL). The aqueous layer was acidified with 1 M HCl to pH ~ 2, and then extracted with MTBE (5 × 300 mL). The combined organic layers were dried (Na_2SO_4), filtered and concentrated under reduced pressure (30 – 15 mmHg, 35 – 50 °C). The residue was dried from pivalic acid under reduced pressure (2.0 – 0.1 mmHg,

80 °C for 10 h) to obtain the product which was triturated in hexane. The BCP acid **S5** (63.00 g, 0.37 mol, 87%) was isolated as a beige solid.

m.p. 185 – 186 °C.

^{1}H NMR (500 MHz, $CDCl_3$) δ 10.88 (1H, br. s, OH), 1.86 (6H, s, H4), 0.84 (9H, s, H1).

^{13}C NMR (126 MHz, $CDCl_3$) δ 177.3, 48.3, 48.2, 35.5, 29.5, 25.9.

HRMS (ESI^-) $[M - H]^-$ $C_{10}H_{15}O_2^-$ requires 167.1072, found 167.1071.

3. Phosphonate BCP Halides

3.1 Phosphonate BCP Halides

Diethyl (3-bromobicyclo[1.1.1]pentan-1-yl)phosphonate, 12

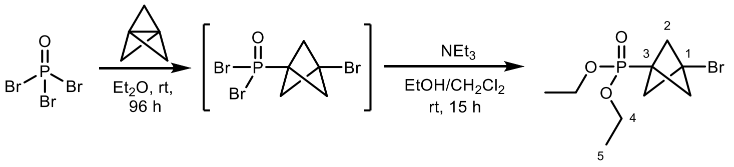

Freshly prepared [1.1.1]propellane **1** (3000 mL, 2.14 mol, 0.7 M in Et_2O, 1.50 equiv.) was added to an Ar degassed solution of phosphoryl tribromide (400.00 g, 1.40 mol, 1.00 equiv.) in Et_2O (1000 mL) in one portion. The mixture was stirred at room temperature for 24 h. The reaction progress was followed by NMR: the aliquot (5 mL) was taken from the reaction mixture and was added to an Ar degassed solution of thiophenol (0.20 g) in Et_2O (10 mL). The mixture was stirred at room temperature for 15 min and concentrated under reduced pressure (30 – 15 mmHg, 20 °C). The residue was analyzed *via* ^1H and ^{31}P NMR. The reaction conversion was ~60%.

After that, an additional portion of [1.1.1]propellane **1** (500 mL, 0.35 mol, 0.7 M in Et_2O, 0.25 equiv.) was added, and the mixture was stirred at room temperature for 3 days. The aliquot was taken for NMR again and showed the conversion of the reaction was ~70%. The resulting mixture was added to a mixture of CH_2Cl_2 (3000 mL), EtOH (1500 mL) and NEt_3 (865.00 g, 1200 mL, 8.57 mol, 6.00 equiv.) slowly over 1 h using intensive mechanical stirring at -10 °C – 5 °C. The mixture was stirred at 5 °C for 3 h and at room temperature for 15 h. The solution was concentrated under reduced pressure (30 – 15 mmHg, 35 °C). The residue was pre-purified by column chromatography (SiO_2, hexane/EtOAc, 2:8). After that, the material was concentrated under reduced pressure (30 – 15 mmHg, 35 – 50 °C) and dried under reduced pressure (1 mmHg, 50 °C) to provide the crude product which was purified again by column chromatography (SiO_2, hexane/EtOAc, 2:8). The resulting product contained triethyl phosphate which was formed from unreacted $POBr_3$. This material can be used without further purification (312.0 g, 0.94 mol, 80% purity by NMR, 67% yield) as a light liquid. The analytical pure sample of **12** was purified by column chromatography (SiO_2, MTBE/$CHCl_3$, 9:1).

R*f* 0.72 (MTBE/$CHCl_3$ 9:1).

^{1}H NMR (500 MHz, $CDCl_3$) δ 4.14 – 4.03 (4H, m, H4), 2.48 (6H, s, H2), 1.31 (6H, t, *J* = 7.1 Hz, H5).

^{13}C NMR (126 MHz, $CDCl_3$) δ 62.4 (d, *J* = 6.4 Hz), 59.0 (d, *J* = 1.5 Hz), 37.9 (d, *J* = 69.0 Hz), 34.3 (d, *J* = 170.1 Hz), 16.7 (d, *J* = 5.9 Hz).

^{31}P {^{1}H} NMR (202 MHz, $CDCl_3$) δ 16.2 (s).

HRMS (ESI$^+$) [M + H]$^+$ $C_9H_{17}BrO_3P^+$ requires 285.0078; found 285.0078.

(3-Bromobicyclo[1.1.1]pentan-1-yl)dimethylphosphine oxide, 13

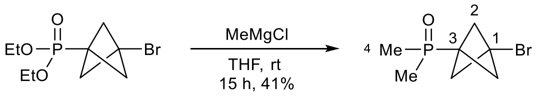

Diethyl (3-bromobicyclo[1.1.1]pentan-1-yl)phosphonate **12** (2.00 g, 7.00 mmol, 1.00 equiv.) was dissolved in dry THF (50 mL) and cooled to -20 °C under Ar atmosphere. A solution of methyl magnesium chloride (3 M in THF, 35.00 mmol, 12 mL, 5.00 equiv.) was added dropwise at -20 °C. The resulting mixture was stirred at room temperature for 15 h. After that, the solution was cooled to -20 °C, and a solution of HCl in dioxane (4 M, 50 mL) was added. The reaction was allowed to warm to room temperature and concentrated under reduced pressure (30 – 15 mmHg, 35 – 40 °C). The residue was purified by column chromatography (SiO_2, CH_3CN/CH_2Cl_2, 0:1 → 1:4) to give the title compound **13** (0.64 g, 2.83 mmol, 41%) as a beige solid.

R*f* 0.60 (CH_3CN/CH_2Cl_2, 1:3).

m.p. 54 – 55 °C.

^{1}H NMR (500 MHz, DMSO-d_6) δ 2.42 (6H, s, H2), 1.39 (3H, d, *J* = 1.6 Hz, H4), 1.36 (3H, d, *J* = 1.5 Hz, H4).

^{13}C NMR (126 MHz, DMSO-d_6) δ 57.8, 38.4, 37.8, 14.6 (d, *J* = 69.4 Hz).

^{31}P {^{1}H} NMR (202 MHz, DMSO-d_6) δ 32.4 (s).

HRMS (ESI$^+$) [M + H]$^+$ $C_7H_{13}BrOP^+$ requires 222.9887; found 222.9880.

3-(Diethoxyphosphoryl)bicyclo[1.1.1]pentane-1-carboxylic acid, 14

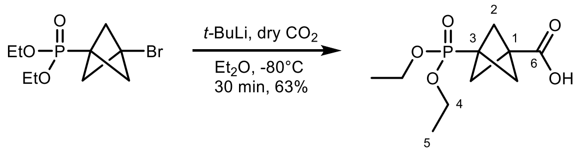

Diethyl (3-bromobicyclo[1.1.1]pentan-1-yl)phosphonate **12** (10.00 g, 35.3 mmol, 1.00 equiv.) was dissolved in dry THF (300 mL) and cooled to -85 °C under Ar atmosphere. *t*-BuLi (1.9 M in pentane, 77.00 mmol, 41 mL, 2.20 equiv.) was added dropwise. The solution was stirred at -80 °C for 30 min and poured on dry CO_2 (300 g). The reaction was allowed to warm to room temperature and sat. aq. NH_4Cl (20 g in 100 mL) was added. The mixture was extracted with MTBE (3 × 100 mL). The aqueous layer was acidified with 1 M HCl, and then extracted with EtOAc (3 × 100 mL). The combined organic layers were dried over Na_2SO_4 and concentrated under reduced pressure (30 – 15 mmHg, 35 – 40 °C). The residue was dried from pivalic acid under reduced pressure (2.0 – 0.1 mmHg, at 80 °C) to obtain crude product which was purified by column chromatography (SiO_2, MTBE/CH_3CN, gradient, 1:0 → 0:1) to give **14** (5.54 g, 0.022 mol, 63%) as a beige solid.

R*f* 0.36 (MeO*t*Bu/CH_3CN, 9:1).

m.p. 138 – 139 °C.

^{1}H NMR (500 MHz, $CDCl_3$) δ 10.25 (1H, br. s, OH), 4.18 – 4.01 (4H, m, H4), 2.31 (6H, s, H2), 1.31 (6H, t, *J* = 7.0 Hz, H5).

^{13}C NMR (126 MHz, $CDCl_3$) δ 171.9 (d, *J* = 34.7 Hz), 62.3 (d, *J* = 6.5 Hz), 52.1 (d, *J* = 2.0 Hz), 41.1 (d, *J* = 37.3 Hz), 32.1 (d, *J* = 165.1 Hz), 16.5 (d, *J* = 5.8 Hz).

^{31}P {^{1}H} NMR (202 MHz, $CDCl_3$) δ 18.9 (s).

HRMS (ESI$^+$) $[M + H]^+$ $C_{10}H_{18}O_5P^+$ requires 249.0892; found 249.0883.

tert-Butyl (3-(diethoxyphosphoryl)bicyclo[1.1.1]pentan-1-yl)carbamate, 15

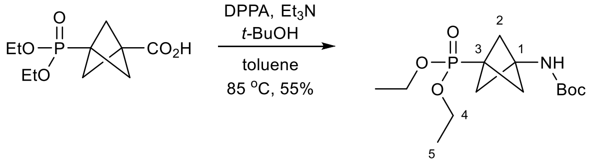

To a solution of 3-(diethoxyphosphoryl)bicyclo[1.1.1]pentane-1-carboxylic acid **14** (1.00 g, 4.00 mmol, 1.00 equiv.) in dry toluene (50 mL) were added Et_3N (0.80 g, 1.1 mL, 8.00 mmol, 2.00 equiv) and dry *t*-BuOH (3.00 g, 4 mL, 40.00 mmol, 10.00 equiv.) under Ar atmosphere. The

solution was stirred at room temperature for 30 min, and DPPA (1.15 g, 4.20 mmol, 1.05 equiv) was added. The resulting mixture was heated at 85 °C for 15 h. The mixture was cooled and concentrated under reduced pressure (30 – 15 mmHg, 35 – 40 °C). The residue was purified by column chromatography (SiO_2, hexane/EtOAc, 1:0 → 0:1) gave the title compound **15** (0.7 g, 2.2 mmol, 55%) as a yellow oil.

R*f* 0.40 (hexane/EtOAc, 1:4).

^{1}H NMR (500 MHz, $CDCl_3$) δ 5.04 (1H, br. s, NH), 4.12 – 4.02 (4H, m, H4), 2.26 (6H, s, H2), 1.42 (9H, s, Boc), 1.30 (6H, t, *J* = 7.0 Hz, H5).

^{13}C NMR (151 MHz, $CDCl_3$) δ 154.8, 80.0, 62.0 (d, *J* = 6.2 Hz), 53.9, 47.9 (d, *J* = 54.8 Hz), 29.7, 28.5, 16.7 (d, *J* = 5.9 Hz).

^{31}P {^{1}H} NMR (162 MHz, $CDCl_3$) δ 21.0 (s).

HRMS (ESI$^+$) $[M + H]^+$ $C_{14}H_{27}NO_5P^+$ requires 320.1627; found 320.1606.

Diethyl (3-aminobicyclo[1.1.1]pentan-1-yl)phosphonate hydrochloride, 16

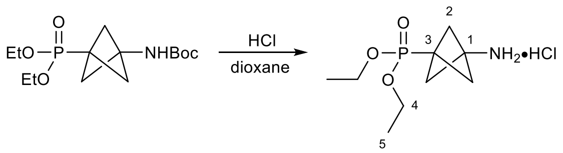

tert-Butyl (3-(diethoxyphosphoryl)bicyclo[1.1.1]pentan-1-yl)carbamate **15** (0.70 g, 2.20 mmol) was added to a solution of HCl (~4 M in dioxane, 50 mL). The solution was stirred at room temperature for 2 h. The mixture was concentrated under reduced pressure (30 – 15 mmHg, 35 – 40 °C). The residue was triturated in Et_2O and filtered. The final product was dried under reduced pressure (0.1 – 2.0 mmHg, at 50 °C for 2 h) to give **16** (0.30 g, 1.17 mol, 53%) as a white solid.

^{1}H NMR (400 MHz, DMSO-d_6) δ 4.06 – 3.91 (4H, m, H4), 3.37 (2H, br. s, NH), 2.11 (6H, s, H2), 1.22 (6H, t, *J* = 6.9 Hz, H5).

^{13}C NMR (126 MHz, DMSO-d_6) δ 61.6 (d, *J* = 6.1 Hz), 52.7, 40.4, 16.4 (d, *J* = 5.4 Hz).

^{31}P {^{1}H} NMR (162 MHz, DMSO-d_6) δ 18.9 (s).

HRMS (ESI$^+$) $[M + H]^+$ $C_9H_{19}NO_3P^+$ requires 220.1103, found 220.1096.

4. Preparation of Starting Materials

4.1 Strained Reagents

[1.1.1]Propellane **1** was prepared as previously reported by our group[4] or on 800g scale (see below). [3.1.1]propellane **4**[6] and BCBs **9a** – **9d**[7] were prepared according to literature procedures.

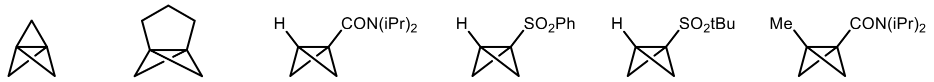

1 4 9a 9b 9c 9d

[1.1.1]Propellane, 1

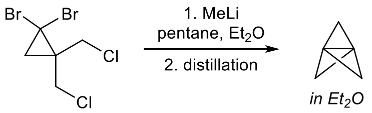

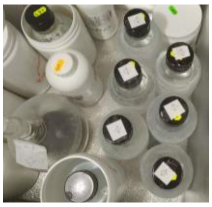

According to a modified literature procedure.[8] A flame dried three-neck round-bottom flask (6 L) equipped with an overhead stirrer was charged with 1,1-bis(chloromethyl)-2,2-dibromocyclopropane (800.00 g, 2.70 mol, 1.00 equiv.) in Et_2O (2 L) under Ar atmosphere. The mixture was cooled to -78 °C, and MeLi (3 M in diethoxymethane, 2 L, 6.00 mol, 2.20 equiv.) was added dropwise at the same temperature under Ar (*large heat release was observed when 1/2 volume of MeLi was added*). The 1st liter of the solution of MeLi was added during ca. 50 min, and the 2[d] liter – during additional 20 min. The reaction mixture was allowed to warm up to -20 °C (ca. 20 min). *Caution: at this temperature, and additional exotherm was observed.* The internal reaction temperature was controlled to not exceed 0 °C. After cease of the exotherm, the reaction mixture was additionally stirred at 0 °C for 1 h. After that, the overhead stirrer was changed to a magnetic stirrer. The mixture was distilled to a 5 L flask (the flask was cooled with liquid nitrogen to ~ -100 °C) under reduced pressure (200 – 10 mmHg, gradually increasing the temperature to 30 °C in the reaction mixture). Distillation was continued until the temperature of vapors reached 30 °C to obtain the propellane solution in Et_2O (10 – 20% diethoxymethane, MeBr and Et_2O). The solution was allowed to warm to -30 °C and was

titrated with benzenethiol. Then the solution was transferred into 1 L bottles for storage (stored under Ar at -30 ± 10 °C) and yielded [1.1.1]propellane **1** (3750 mL, 2.10 mol, 0.7 M in Et_2O, 78%).

Titration of propellane with thiophenol:

A solution of thiophenol (3.00 g) in Et_2O degassed with Ar was added to a solution of **1** (10 mL). The mixture was stirred for 15 min at room temperature, concentrated *in vacuo*, and subjected to ^1H NMR. The ratio of the obtained PhS-BCP-H and remaining PhSH was calculated based on the proton of the tertiary carbon in propellane **1** at 2.72 ppm (1H, s; $CDCl_3$) and the proton in PhSH at 3.44 ppm (1H, s; $CDCl_3$).

4.2 (Het)Aryl Sulfonyl Halides

4-Methylbenzenesulfonyl bromide

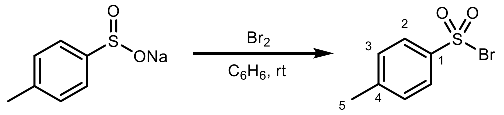

To a solution of sodium 4-methylbenzenesulfinate **2a** (10.00 g, 0.056 mol, 1.00 equiv.) in dry benzene (100 mL) was added bromine (8.50 g, 2.7 mL, 0.053 mol, 0.95 equiv.) in two portions at room temperature with vigorous stirring. The resulting mixture was stirred for 30 min. After that, the reaction mixture was filtered and washed with a small amount of benzene (10 mL). The filtrate was concentrated under reduced pressure (30 – 15 mmHg, 35 °C) to afford the desired product (13 g crude, 90% purity by NMR with residue of benzene, 95% yield) which was used in the next step without purification. An analytically pure sample was triturated (pentane), filtered and dried under reduced pressure (10 mmHg, 35 °C) to give a white solid.

m.p. 96 – 97 °C.

^{1}H NMR (500 MHz, $CDCl_3$) δ 7.88 (2H, d, *J* = 8.3 Hz, H2), 7.39 (2H, d, *J* = 8.3 Hz, H3), 2.49 (3H, s, H5).

^{13}C NMR (126 MHz, $CDCl_3$) δ 146.9, 144.8, 130.3, 126.7, 22.0.

HRMS (ESI$^{+/-}$) Not found.

Benzenesulfonyl bromide

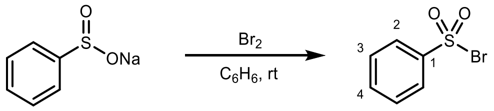

To a mixture of sodium benzenesulfinate **2b** (100.00 g, 0.60 mol, 1.00 equiv.) in dry benzene (1000 mL) was added bromine (92.00 g, 30 mL, 0.578 mol, 0.95 equiv.) at room temperature, over 10 min with vigorous stirring. The resulting mixture was stirred for 30 min at room temperature. After that, the reaction mixture was filtered and washed with a small amount of benzene (100 mL). The filtrate was concentrated under reduced pressure (30 – 15 mmHg, 35 °C) to afford the desired product (130 g crude, 90% purity by NMR with residue of benzene, 90% yield) which was used in the next step without purification. An analytically pure sample was obtained by vacuum distillation (1 mmHg, b.p. = 68 – 70 °C) as a colorless oil.

^{1}**H NMR** (500 MHz, $CDCl_3$) δ 8.01 (2H, d, *J* = 8.2 Hz, H2), 7.74 (1H, t, *J* = 7.5 Hz, H4), 7.62 (2H, t, *J* = 7.8 Hz, H3).

^{13}**C NMR** (126 MHz, $CDCl_3$) δ 147.3, 135.3, 129.8, 126.6.

HRMS ($ESI^{+/-}$) Not found.

Benzenesulfonyl iodide

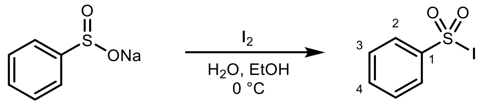

To a round-bottom flask (1000 mL) was added sodium benzenesulfinate **2b** (50.00 g, 0.30 mol), dissolved in 500 mL of distilled water at room temperature. A saturated solution of iodine (60.00 g, 0.24 mol) in EtOH (150 mL) was prepared and added gradually at 0 °C until a slight excess of iodine was present. During this period, the yellow precipitate was formed. The precipitate was filtered, washed with cold water (50 mL), and dried at room temperature to give benzenesulfonyl iodide (46.00 g, 0.17 mol, 58%) as a yellow solid.

m.p. 33 – 34 °C.

^{1}**H NMR** (500 MHz, $CDCl_3$) δ 7.87 (2H, d, *J* = 7.5 Hz, H2), 7.69 (1H, t, *J* = 7.5 Hz, H4), 7.56 (2H, t, *J* = 7.9 Hz, H3).

^{13}**C NMR** (126 MHz, $CDCl_3$) δ 134.9, 129.4, 128.8, 125.5.

HRMS ($ESI^{+/-}$) Not found.

4-Methoxybenzenesulfonyl bromide

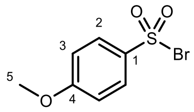

The conditions of **general procedure N** gave the sulfonyl bromide (41.00 g, 0.16 mol, 54%) as a yellow soild.

^{1}**H NMR** (500 MHz, $CDCl_3$) δ 7.94 (2H, d, *J* = 9.0 Hz, H2), 7.03 (2H, d, *J* = 8.9 Hz, H3), 3.93 (3H, s, H5).

^{13}**C NMR** (126 MHz, $CDCl_3$) δ 164.9, 139.3, 129.2, 114.7, 56.2.

HRMS ($ESI^{+/-}$) Not found.

4-Fluorobenzenesulfonyl bromide

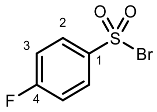

The conditions of **general procedure N** gave the sulfonyl bromide (33.40 g, 0.14 mol, 51%) as a yellow oil.

^{1}H NMR (500 MHz, $CDCl_3$) δ 8.08 – 7.99 (2H, m, H2), 7.29 (2H, t, J = 8.5 Hz, H3).
^{13}C NMR (126 MHz, $CDCl_3$) δ 166.4 (d, $^1J_{CF}$ = 260.0 Hz), 143.2, 129.8 (d, $^3J_{CF}$ = 10.1 Hz), 117.1 (d, $^2J_{CF}$ = 23.3 Hz).
^{19}F {^{1}H} NMR (376 MHz, $CDCl_3$) δ -100.1 (s).
HRMS ($ESI^{+/-}$) Not found.

3-Fluorobenzenesulfonyl bromide

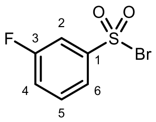

The conditions of **general procedure N** gave the sulfonyl bromide (31.07 g, 0.13 mol, 58%) as a yellow soild.

^{1}H NMR (500 MHz, $CDCl_3$) δ 7.82 (1H, d, J = 8.0 Hz, H6), 7.70 (1H, dt, J = 5.5, 2.1 Hz, H2), 7.62 (1H, td, J = 8.1, 5.2 Hz, H4), 7.45 (1H, td, J = 8.2, 2.3 Hz, H5).
^{13}C NMR (126 MHz, $CDCl_3$) δ 162.1 (d, $^1J_{CF}$ = 254.7 Hz), 148.3 (d, $^3J_{CF}$ = 7.5 Hz), 131.5 (d, $^3J_{CF}$ = 7.7 Hz), 122.7 (d, $^2J_{CF}$ = 21.2 Hz), 122.5 (d, $^4J_{CF}$ = 3.4 Hz), 114.2 (d, $^2J_{CF}$ = 25.5 Hz).
^{19}F {^{1}H} NMR (376 MHz, $CDCl_3$) δ -107.7 (s).
HRMS ($ESI^{+/-}$) Not found.

4-(Trifluoromethyl)benzenesulfonyl bromide

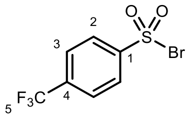

The conditions of **general procedure N** gave the sulfonyl bromide (39.40 g, 0.136 mol, 68%) as a beige solid.

^{1}H NMR (500 MHz, $CDCl_3$) δ 8.15 (2H, d, *J* = 8.3 Hz, H2), 7.90 (2H, d, *J* = 8.4 Hz, H3).
^{13}C NMR (151 MHz, $CDCl_3$) δ 149.8, 136.7 (q, $^{2}J_{CF3}$ = 33.7 Hz), 127.3, 127.1 (q, $^{3}J_{CF3}$ = 3.5 Hz), 122.9 (q, $^{1}J_{CF3}$ = 273.5 Hz).
^{19}F {^{1}H} NMR (376 MHz, $CDCl_3$) δ -63.9 (s).
HRMS (ESI$^{+/-}$) Not found.

Thiophene-2-sulfonyl bromide

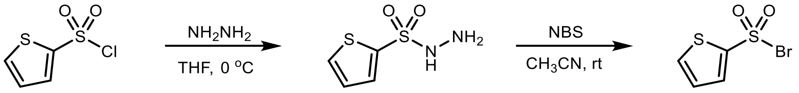

Step 1: Thiophene-2-sulfonohydrazide

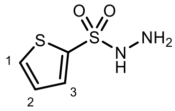

Thiophene-2-sulfonyl chloride (10.00 g, 54 mmol, 1.00 equiv.) was dissolved in dry THF (300 mL) and cooled to 0 °C under Ar atmosphere. Hydrazine monohydrate (7.80 g, 8 mL, 162 mmol, 3.00 equiv.) was added dropwise, and the solution was stirred at 0 °C for 30 min. After that the solvent was removed under reduced pressure (10 mmHg, 35 °C). The residue was extracted with CH_2Cl_2 (3 × 200 mL), and the combined organic layers were washed with water (3 × 200 mL), brine (3 × 200 mL), dried over Na_2SO_4 and concentrated under reduced pressure. The residue was triturated (hexane/MTBE, 9:1, ca. 100 mL), filtered and dried under reduced pressure (10 mmHg, 35 °C) to give the sulfonyl chloride (7 g, 38.8 mmol, 90% purity by NMR, 72% yield) as a yellow oil.

R*f* 0.41 (CH_2Cl_2/MeOH, 95:5).

^{1}H NMR (500 MHz, $CDCl_3$) δ 7.75 – 7.63 (2H, m, H1, H3), 7.17 (1H, t, *J* = 4.1 Hz, H2), 5.72 (1H, br. s, NH), 3.27 (2H, br. s, NH).

^{13}C NMR (126 MHz, $CDCl_3$) δ 136.7, 134.4, 133.7, 128.0.

HRMS (ESI$^-$) [M - H]$^-$ $C_4H_5N_2O_2S_2^-$ requires 176,9792; found 176.9787.

Step 2: Thiophene-2-sulfonyl bromide

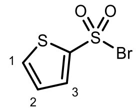

Thiophene-2-sulfonohydrazide (5.00 g, 28.00 mmol, 1.00 equiv.) was dissolved in dry CH_3CN (100 mL) and cooled to 0 °C under Ar atmosphere. NBS (10.00 g, 56.00 mmol, 2.00 equiv.) was added in one portion. The mixture was stirred at room temperature for 5 h. After that the solvent was removed under reduced pressure (10 mmHg, 25 °C). The residue was purified by column chromatography (SiO_2, hexane/EtOAc, 7:3) to give the sufonyl bromide (4.80 g, 0.021 mol, 90 % purity by NMR, 76%) as a yellow solid.

R*f* 0.6 (hexane/EtOAc, 7:3).

m.p. 51 – 52 °C.

^{1}H NMR (500 MHz, $CDCl_3$) δ 7.87 (1H, dd, *J* = 3.8, 1.1 Hz, H1), 7.81 (1H, dd, *J* = 5.0, 1.1 Hz, H3), 7.18 (1H, t, *J* = 4.2 Hz, H2).

^{13}C NMR (126 MHz, $CDCl_3$) δ 147.2, 135.5, 134.2, 127.7.

HRMS (ESI$^{+/-}$) Not found.

4.3 Alkyl Sulfonyl Bromides

Methanesulfonyl bromide

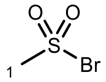

The conditions of **general procedure M** gave sulfonyl bromide (120 g crude, 90% purity by NMR with residue of CH_2Cl_2, 81%) which was used for the next step without purification. An analytically pure sample was obtained by vacuum distillation (1 mmHg, b.p. 40 – 50 °C) as a light brown liquid.

^{1}H NMR (500 MHz, $CDCl_3$) δ 3.83 (3H, s, H1).
^{13}C NMR (126 MHz, $CDCl_3$) δ 57.1.
GC (M) 159.
HRMS (ESI$^{+/-}$) Not found.

Ethanesulfonyl bromide

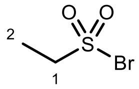

The conditions of **general procedure M** gave sulfonyl bromide (50 g crude, 90% purity by NMR with the residue of CH_2Cl_2, 70%), which was used for the next step without purification. An analytically pure sample was obtained by vacuum distillation (1 mmHg, b.p. = 25 °C) as a colorless oil.

^{1}H NMR (500 MHz, $CDCl_3$) δ 3.74 (2H, q, *J* = 7.3 Hz, H1), 1.57 (3H, t, *J* = 7.3 Hz, H2).
^{13}C NMR (151 MHz, $CDCl_3$) δ 64.4, 9.8.
GC (M) 174.
HRMS (ESI$^{+/-}$) Not found.

Propane-2-sulfonyl bromide

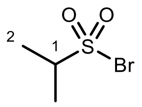

The conditions of **general procedure M** gave sulfonyl bromide (46 g crude, 90% purity by NMR with the residue of CH_2Cl_2, 63%), which was used for the next step without purification. An analytically pure sample was obtained by vacuum distillation (1 mmHg, b.p. = 26 °C) as a colorless oil.

^{1}H NMR (500 MHz, $CDCl_3$) δ 3.66 (1H, t, *J* = 6.6 Hz, H1), 1.54 (6H, d, *J* = 6.6 Hz, H2).
^{13}C NMR (126 MHz, $CDCl_3$) δ 71.2, 17.7.
HRMS (ESI$^{+/-}$) Not found.

Cyclopropanesulfonyl bromide

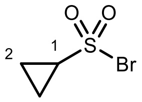

The conditions of **general procedure M** gave sulfonyl bromide (48.5 g crude, 95% purity by NMR, 75%), which was used for the next step without purification. An analytically pure sample was obtained by vacuum distillation (1 mmHg, b.p. = 30 °C) as a colorless oil.

^{1}H NMR (500 MHz, $CDCl_3$) δ 3.53 – 3.35 (1H, m, H1), 1.64 – 1.56 (2H, m, H2), 1.41 – 1.32 (2H, m, H2).
^{13}C NMR (126 MHz, $CDCl_3$) δ 47.7, 10.1.
HRMS (ESI$^{+/-}$) Not found.

4.4 Aryl Sulfinate Salts

Preparation of sulfinates not listed here were either commercially available or previously reported in literature.[1, 9]

Sodium 4-methoxybenzenesulfinate, 2c

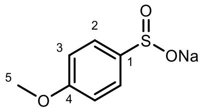

4-Methoxybenzenesulfonyl chloride (3.13 g, 10.0 mmol), sodium sulfite (2.52 g, 20.0 mmol) and sodium bicarbonate (1.68 g, 20.0 mmol) in water (10.0 mL) were subjected to the conditions of **general procedure J** to give the sulfinate salt **2c** (2.53 g, 8.45 mmol, 84%) as a white solid. The product contained an 8% impurity by ^{1}H NMR ($ArSO_3H$).

Alternative preparation: The conditions of **general procedure K** gave sulfinate salt **2c** (59.0 g, 0.30 mol, 90%) as a white solid.

IR ν_{max}/cm^{-1} (film) 2522, 1593, 1493, 1248, 1084, 1012, 981.
^{1}H NMR (400 MHz, MeOD) δ 7.62 – 7.53 (2H, m, H2), 7.00 – 6.92 (2H, m, H3), 3.81 (3H, s, H5).
^{1}H NMR (400 MHz, D_2O) δ 7.59 (d, *J* = 8.6 Hz, 2H), 7.09 (d, *J* = 8.6 Hz, 2H), 3.87 (s, 3H).
^{13}C NMR (101 MHz, MeOD) δ 162.2, 149.3, 126.7, 114.7, 55.8.
^{13}C NMR (126 MHz, D_2O) δ 160.4, 145.9, 125.2, 114.3, 55.4.
HRMS (ESI$^-$) [M - Na]$^-$ $C_7H_7O_3S$ requires 171.0121; found 171.0112.

Sodium 4-bromobenzenesulfinate, 2d

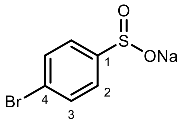

4-Bromobenzenesulfonyl chloride (1.28 g, 5.00 mmol), sodium sulfite (1.26 g, 10.0 mmol) and sodium bicarbonate (840 mg, 10.0 mmol) in water (5.0 mL) were subjected to the conditions of **general procedure J** to give the sulfinate salt **2d** (1.23 g, 5.08 mmol, quant.) as a white solid.

IR ν_{max}/cm^{-1} (film) 2437, 1569, 1469, 1048, 997, 975, 825, 725.

^{1}H NMR (400 MHz, MeOD) δ 7.56 (4H, app. d, J = 1.2 Hz, ArH).

^{13}C NMR (101 MHz, MeOD) δ 137.8, 132.5, 127.3, 122.9

HRMS (ESI$^-$) [M - Na]$^-$ $C_6H_4O_2{}^{79}BrS^-$ requires 218.9121; found 218.9115.

Sodium 4-fluorobenzenesulfinate, 2e

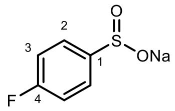

The conditions of **general procedure K** gave sulfinate salt **2e** (51.0 g, 0.28 mol, 77%) as a white solid.

m.p. 320 – 321 °C.

^{1}H NMR (400 MHz, DMSO-d$_6$) δ 7.51 (2H, dd, J = 8.4, 6.0 Hz, H2), 7.12 (2H, t, J = 8.9 Hz, H3).

^{13}C NMR (151 MHz, DMSO-d$_6$) δ 161.9 (d, $^1J_{CF}$ = 242.5 Hz), 156.2, 126.4 (d, $^3J_{CF}$ = 8.3 Hz), 114.3 (d, $^2J_{CF}$ = 21.1 Hz).

^{19}F {^{1}H} NMR (376 MHz, DMSO-d$_6$) δ -115.6 (s).

HRMS (ESI$^{+/-}$) Not found.

Sodium 3-fluorobenzenesulfinate, 2f

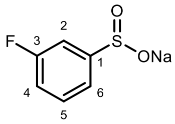

The conditions of **general procedure K** gave sulfinate salt **2f** (41.60 g, 0.22 mol, 89%) as a white solid.

^{1}H NMR (400 MHz, D_2O) δ 7.41 – 7.30 (1H, m, H2), 7.30 – 7.24 (1H, m, H6), 7.20 (1H, d, J = 8.4 Hz, H4), 7.07 – 6.96 (1H, m, H5).

^{13}C NMR (151 MHz, DMSO-d$_6$) δ 163.2, 162.1 (d, $^1J_{CF}$ = 246.7 Hz), 129.7 (d, $^3J_{CF}$ = 6.9 Hz), 120.5 (d, $^4J_{CF}$ = 2.3 Hz), 114.5 (d, $^2J_{CF}$ = 21.7 Hz), 110.4 (d, $^2J_{CF}$ = 20.7 Hz).

^{19}F {^{1}H} NMR (376 MHz, D_2O) δ -114.0 (s).

HRMS (ESI$^{+/-}$) Not found.

Sodium 4-nitrobenzenesulfinate, 2h

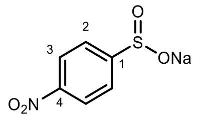

4-Nitrobenzenesulfonyl chloride (5.20 g, 23.5 mmol), sodium sulfite (6.00 g, 47.0 mmol) and sodium bicarbonate (4.00 mg, 47.0 mmol) in water (50 mL) were subjected to the conditions of **general procedure J** to give the sulfinate salt **2h** (4.70 g, 22.6 mmol, 96%) as an orange solid. The product contained a 12% impurity by ^{1}H NMR ($ArSO_3H$).

IR ν_{max}/cm^{-1} (film) 2361, 1517, 1354, 1040, 976, 853.
^{1}**H NMR** (400 MHz, MeOD) δ 8.34 – 8.25 (2H, m, H3), 7.89 – 7.83 (2H, m, H2).
^{13}**C NMR** (101 MHz, MeOD) δ 164.1, 128.4, 126.6, 124.7.
HRMS (ESI^{-}) [M - Na]$^{-}$ $C_6H_4O_4NS$ requires 185.9867; found 185.9858.

Sodium 4-(trifluoromethyl)benzenesulfinate, 2i

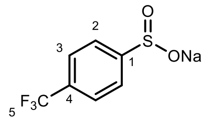

The conditions of **general procedure K** gave sulfinate salt **2i** (47.40 g, 0.20 mol, 84%) as a white solid.

m.p. 270 – 272 °C.
^{1}**H NMR** (500 MHz, D_2O) δ 7.85 (2H, d, J = 8.2 Hz, H2), 7.79 (2H, d, J = 8.1 Hz, H3).
^{13}**C NMR** (126 MHz, D_2O) δ 156.8, 131.0 (q, $^{2}J_{CF3}$ = 32.1 Hz), 125.6 (q, $^{3}J_{CF3}$ = 3.7 Hz), 123.7, 123.5 (q, $^{1}J_{CF3}$ = 271.7 Hz).
^{19}**F {^{1}H} NMR** (376 MHz, D_2O) δ -62.9 (s).
HRMS (ESI$^{+/-}$) Not found.

Sodium 4-(methoxycarbonyl)benzenesulfinate, 2j

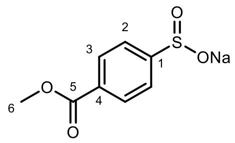

The conditions of **general procedure J** gave sulfinate salt **2j** (240 mg, 1.06 mmol, 25%) as a white solid.

^{1}H NMR (400 MHz, MeOD) δ 8.08 (2H, d, J = 8.4 Hz, H2), 7.75 (2H, d, J = 8.6 Hz, H3), 3.91 (3H, s, H5).
^{13}C NMR (101 MHz, MeOD) δ 168.2, 162.1, 132.1, 130.8, 125.5, 52.7

Sodium 3,5-difluorobenzenesulfinate, 2m

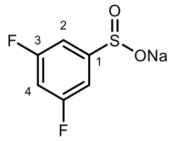

3,5-Difluorobenzenesulfonyl chloride (5.00 g, 23.5 mmol), sodium sulfite (6.00 g, 47.0 mmol) and sodium bicarbonate (4.00 mg, 47.0 mmol) in water (50 mL) were subjected to the conditions of **general procedure J** to give the sulfinate salt **2m** (4.70 g, 23.5 mmol, quant.) as a white solid.

IR ν_{max}/cm^{-1} (film) 1604, 1432, 1283, 1124, 1003, 977, 677.
^{1}H NMR (400 MHz, MeOD) δ 7.25 – 7.18 (2H, m, H2), 6.93 (1H, tt, $^{3}J_{HF}$ = 8.9, 2.4 Hz, H4).
^{13}C NMR (101 MHz, MeOD) δ 164.37 (dd, $^{1,3}J_{CF}$ = 251.7, 10.9 Hz), 162.59 (t, $^{3}J_{CF}$ = 4.1 Hz), 108.40 – 107.97 (m), 105.25 (t, $^{2}J_{CF}$ = 26.3 Hz).
^{19}F NMR (377 MHz, MeOD) δ -110.8.
HRMS (ESI^{-}) [M - Na]$^{-}$ $C_6H_3O_2F_2S^{-}$ requires 176.9827; found 176.9818.

Sodium 3,5-bis(trifluoromethyl)benzenesulfinate, 2n

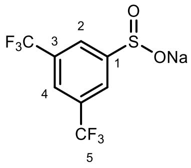

3,5-Bis(trifluoromethyl)benzenesulfonyl chloride (3.13 g, 10.0 mmol), sodium sulfite (2.52 g, 20.0 mmol) and sodium bicarbonate (1.68 g, 20.0 mmol) in water (10.0 mL) were subjected to the conditions of **general procedure J** to give the sulfinate salt **2n** (2.53 g, 8.45 mmol, 84%) as a white solid. The product contained a 14% impurity by ^{1}H NMR ($ArSO_3H$).

IR ν_{max}/cm^{-1} (film) 2361, 2339, 1278, 1106.

^{1}H NMR (600 MHz, MeOD) δ 8.19 (2H, qd, *J* = 1.1, 0.6 Hz, H2), 7.96 (1H, app. tp, *J* = 1.5, 0.7 Hz, H4).

^{13}C NMR (151 MHz, MeOD) δ 161.3, 132.8 (q, $^2J_{CF3}$ = 33.3 Hz), 126.3 (q, $^3J_{CF3}$ = 3.9 Hz), 124.8 (q, $^1J_{CF3}$ = 272.1 Hz), 123.8 (s, $^3J_{CF3}$ = 3.8 Hz).

^{19}F NMR (377 MHz, MeOD) δ -64.3.

HRMS (ESI$^-$) [M - Na]$^-$ $C_8H_3O_2F_6S^-$ requires 276.9763; found 276.9760.

Sodium 4-acetamidobenzenesulfinate, 2o

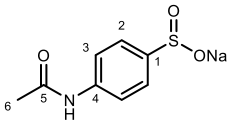

N-Acetylsulfinailyl chloride (1.17 g, 5.00 mmol), sodium sulfite (1.26 g, 10.0 mmol) and sodium bicarbonate (840 mg, 10.0 mmol) in water (5.0 mL) were subjected to the conditions of **general procedure J** to give the sulfinate salt **2o** (1.49 g, 6.74 mmol, quant.) as a white solid.

IR ν_{max}/cm^{-1} (film) 1654, 1600, 1394, 1190, 1134, 1051, 650.

^{1}H NMR (400 MHz, MeOD) δ 7.66 – 7.55 (4H, m, ArH), 2.13 (3H, s, H6).

^{13}C NMR (101 MHz, MeOD) δ 171.7, 152.6, 141.1, 125.9, 120.6, 23.9.

HRMS (ESI$^-$) [M - Na]$^-$ $C_8H_8O_3NS^-$ requires 198.0230; found 198.0220.

Sodium benzene-1,3-disulfinate, 2p

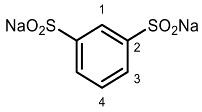

Benzene-1,3-disulfonyl dichloride (1.00 g, 3.65 mmol), sodium sulfite (1.84 g, 14.6 mmol) and sodium bicarbonate (1.23 g, 14.6 mmol) in H_2O (7.2 mL) were subjected to the conditions of **general procedure J** to give the sulfinate salt **2p** (1.00 g, 4.00 mmol, quant.) as a white solid.

^{1}H NMR (400 MHz, MeOD) δ 7.98 (1H, td, *J* = 1.7, 0.6 Hz, H1), 7.70 (2H, dd, *J* = 7.6, 1.7 Hz, H3), 7.51 (1H, app. ddd, *J* = 7.8, 7.2, 0.6 Hz, H4).
^{13}C NMR (101 MHz, MeOD) δ 157.7, 129.7, 126.1, 121.6.
HRMS (ESI$^{+/-}$) Not found.

4.5 Pharmaceutical and Agrochemical Sulfinate Salts

Sodium 4-ethoxy-3-(1-methyl–7-oxo-3-propyl-6,7-dihydro-1H-pyrazolo[4,3-d]pyrimidin-5-yl)benzenesulfinate, 2z

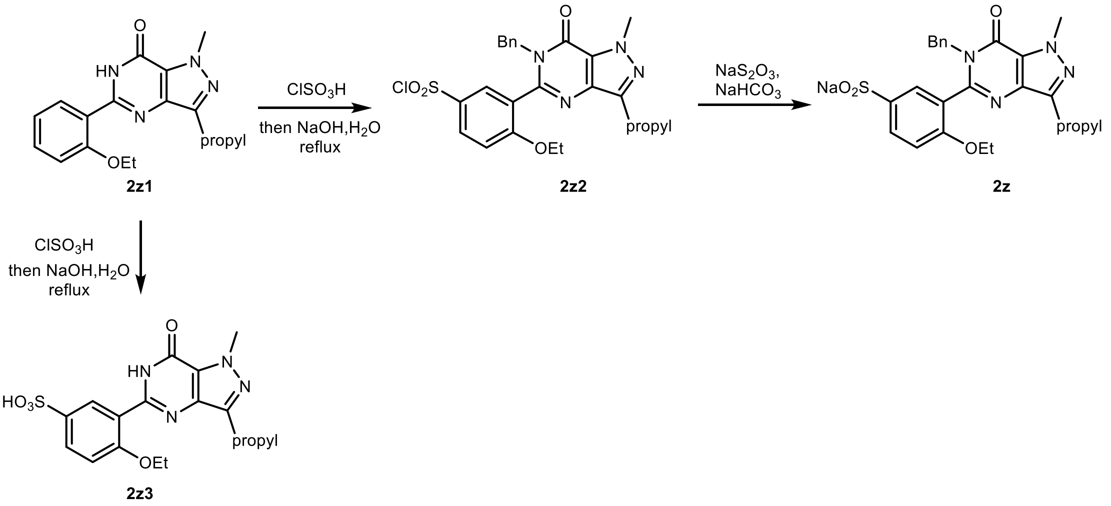

Step 1: 4-Ethoxy-3-(1-methyl-7-oxo-3-propyl-6,7-dihydro-1H-pyrazolo[4,3-d]pyrimidin-5-yl)benzenesulfonyl chloride, **2z2**

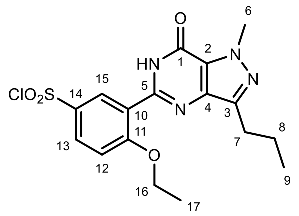

According to a modified literature procedure.[10, 11] 5-(2-ethoxyphenyl)-1-methyl-3-propyl-1H pyrazolo[4,3-d]pyrimidin-7(6H)-one **2z1** (1.00 g, 3.20 mmol) was added portionwise to neat chlorosulphonic acid (3.0 mL, 45.1 mmol) at 0 °C. The reaction mixture was stirred at room temperature for 4 h. The reaction mixture was then cooled to 0 °C and $CHCl_3$ (2 mL) was added slowly to the reaction mixture. The reaction mixture was then added slowly to a stirred conical flask containing ice-water (5 mL). The phases were separated and the aqueous phase was extracted with $CHCl_3$/MeOH (9:1, 3 × 10 mL). The combined organic phases were washed with brine (30 mL), dried ($MgSO_4$), filtered and concentrated *in vacuo* to give sulfonyl chloride **2z2** (1.12 g, 2.90 mmol, 91%) as a white solid.

R_f 0.55 (EtOAc).

m.p. 145 – 148 °C [lit. 179 – 181 °C].[11]

IR ν_{max}/cm^{-1} (film) 1683, 1596, 1376, 1177, 1155, 732.

^{1}H NMR (600 MHz, $CDCl_3$) δ 10.71 (1H, br. s, NH), 9.12 (1H, d, *J* = 2.6 Hz, H15), 8.11 (1H, dd, *J* = 9.0, 2.6 Hz, H13), 7.23 (1H, d, *J* = 9.0 Hz, H12), 4.44 (2H, q, *J* = 7.0 Hz, H16), 4.28 (3H, s, H6), 2.95 (2H, t, *J* = 7.6 Hz, H7), 1.87 (2H, app. sxt., *J* = 7.4 Hz, H8), 1.67 (3H, t, *J* = 7.0 Hz, H17), 1.04 (3H, t, *J* = 7.4 Hz, H9).

^{13}C NMR (151 MHz, $CDCl_3$) δ 161.0, 153.6, 147.4, 145.8, 138.3, 137.8, 131.2, 131.1, 124.7, 121.9, 113.7, 66.8, 38.4, 27.8, 22.5, 14.6, 14.2.

HRMS ($ESI^{+/-}$) not found.

Step 2: Sodium 4-ethoxy-3-(1-methyl-7-oxo-3-propyl-6,7-dihydro-1H-pyrazolo[4,3-d]pyrimidin – 5-yl)benzenesulfinate, **2z**

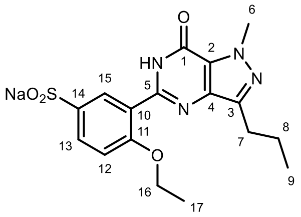

Sulfonyl chloride **2z2** (1.11 g, 3.20 mmol), sodium sulfite (808 mg, 6.41 mmol) and sodium bicarbonate (538 mg, 6.41 mmol) in water (6.4 mL) were subjected to the conditions of **general procedure J** to give the sulfinate salt **2z** (1.03 g, 2.59 mmol, 81%) as a white solid. The product contained a 25% impurity by ^{1}H NMR ($ArSO_3H$).

IR ν_{max}/cm^{-1} (film) 3414 (br.), 3285, 1705, 1581, 1537, 1206, 977, 776.

^{1}H NMR (600 MHz, MeOD) δ 8.37 (1H, d, *J* = 2.3 Hz, H15), 7.95 (1H, dd, *J* = 8.8, 2.3 Hz, H12/13), 7.21 (1H, d, *J* = 8.8 Hz, H12/13), 4.26 (2H, app. pentet, *J* = 7.0 Hz, H16), 4.23 (3H, s, H6), 2.88 (2H, t, *J* = 7.5 Hz, H7), 1.82 (2H, sxt., *J* = 7.4 Hz, H8), 1.47 (3H, app. td, *J* = 7.0, 2.8 Hz, H17), 1.00 (3H, t, *J* = 7.5 Hz, H9).

^{13}C NMR (151 MHz, MeOD) δ 159.3, 155.7, 150.6, 147.5, 139.7, 139.2, 131.2, 129.8, 125.8, 122.8, 113.4, 66.3, 38.4, 28.4, 23.5, 14.9, 14.2.

HRMS (ESI^+) $[M + Na]^+$ $C_{17}H_{20}O_4N_4NaS^+$ requires 399.1097; found 399.1098.

*4-Ethoxy-3-(1-methyl-7-oxo-3-propyl-6,7-dihydro-1H-pyrazolo[4,3-d]pyrimidin-5-yl)benzenesulfonic acid, **2z3***

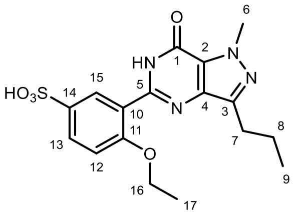

A solution sulfonyl chloride **2z2** (1.19 g, 2.90 mmol) in H_2O/EtOH (1:1, 20 mL) was heated to 100 °C for 18 h. The reaction mixture was then cooled to room temperature and concentrated *in vacuo* to give sulfonic acid **2z3** (1.23 g, 3.15 mmol, quant.) as a white solid.

*Note: Compound was not required for the synthesis of **2z** but included for reference.*

m.p. 235 °C [lit. 179 – 181 °C].[11]

IR ν_{max}/cm^{-1} (film) 3366 (br.), 1735, 1638, 1199, 1185, 1158, 1144, 1035

^{1}H NMR (400 MHz, DMSO-d$_6$) δ 7.86 (1H, d, *J* = 2.2 Hz, H15), 7.70 (1H, dd, *J* = 8.6, 2.2 Hz, H13), 7.11 (1H, d, *J* = 8.6 Hz, H12), 5.83 (1H, br. s, OH), 4.16 (3H, s, H6), 4.13 (2H, q, *J* = 7.0 Hz, H16), 2.79 (2H, t, *J* = 7.4 Hz, H7), 1.73 (2H, sxt., *J* = 7.4 Hz, H8), 1.32 (3H, t, *J* = 7.0 Hz, H17), 0.93 (3H, t, *J* = 7.4 Hz, H9).

^{13}C NMR (101 MHz, DMSO-d$_6$) δ 156.5, 153.5, 150.0, 144.3, 140.6, 136.4, 129.5, 128.1, 124.4, 120.6, 111.9, 64.3, 37.9, 27.2, 21.9, 14.5, 13.8.

HRMS (ESI$^+$) [M + H]$^+$ $C_{17}H_{21}O_5N_4S^+$ requires 393.1227; found 393.1227.

Sodium 4-(diethylcarbamoyl)-4H-1,2,4-triazole-3-Sulfinate, 2aa

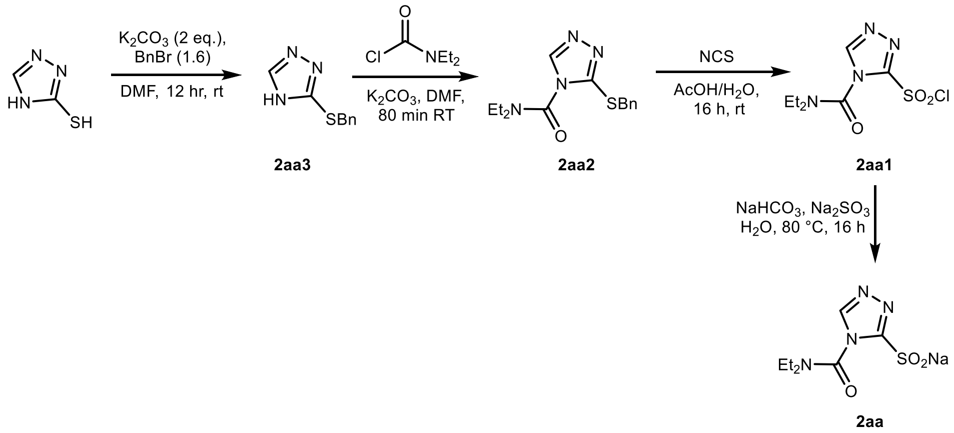

Step 1&2: 3-(Benzylthio)-N,N-diethyl-4H-1,2,4-triazole-4-carboxamide, 2aa2

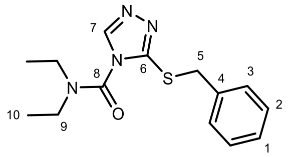

According to a modified literature procedure.[12] K_2CO_3 (2.76 g, 20.0 mmol) then benzyl bromide (1.43 mL, 12.0 mmol) was added to a solution of 2,4-dihydro-18H-,2,4-triazole-3-thione (1.01 g, 10.0 mmol) in anhydrous DMF (10 mL). The reaction mixture was stirred at room temperature for 48 h. A further portion of K_2CO_3 (1.38 g, 10.0 mmol) was added, followed by diethylcarbamic chloride (1.89 mL, 15.0 mmol). The reaction mixture was stirred for 1 h, and then poured onto H_2O (20 mL) and extracted with EtOAc (15 mL × 2). The combined organic phases were washed with 10% aq. LiCl (10 mL × 3), dried ($MgSO_4$), filtered and concentrated *in vacuo*. Purification by column chromatography (SiO_2, pentane → EtOAc/pentane 1:9) gave **2aa2** (2.27 g, 7.83 mmol, 78%) as a white solid.

Note: The product contained di-benzylated impurity, a stepwise approach is recommended for ease of purification. Data for 3-(benzylthio)-4H-1,2,4-triazole-methane, ***2aa3.***[13]

R_f 0.39 (EtOAc/pentane, 1:4).

IR ν_{max}/cm^{-1} (film) 2361, 1693, 1429, 1269, 1252, 1207, 913, 862, 745.

^{1}H NMR (400 MHz, $CDCl_3$) δ 8.75 (1H, s, H7), 7.42 – 7.38 (1H, m, ArH), 7.33 – 7.25 (4H, m, ArH), 4.38 (2H, s, H5), 3.56 (4H, br. s, H9), 1.25 (6H, t, *J* = 7.1 Hz, H10).

^{13}C NMR (101 MHz, $CDCl_3$) δ 162.5, 147.5, 137.0, 128.9, 128.7, 127.6, 43.7, 36.2, 13.6.

HRMS (ESI$^+$) [M + Na]$^+$ $C_{14}H_{18}ON_4NaS^+$ requires 313.1094; found 313.1093.

Step 3: 4-(Diethylcarbamoyl)-4H-1,2,4-triazole-3-sulfonyl chloride, ***2aa1***

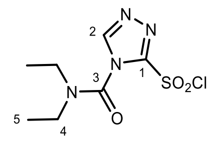

According to a modified literature procedure.[12] NCS (5.34 g, 40.0 mmol) was added to a solution of 3-(benzylthio)-1-isopropyl-1H-1,2,4-triazole (2.27 g, 7.83 mmol) in AcOH (40 mL) and water (20 mL). The mixture was stirred for 2 h, then partitioned between EtOAc (200 mL) and water (200 mL). The organic layer was washed with sat. aq. $NaHCO_3$ (100 mL), brine (50 mL), dried ($MgSO_4$), filtered and evaporated. TBME (20 mL) was added to the residue, the solid filtered off and the filtrate evaporated. Purification by column chromatography (SiO_2, EtOAc/pentane, 1:9) gave the sulfonyl chloride **2aa1** (738 mg, 2.77 mmol, 35%) as a colourless oil.

R_f 0.33 (EtOAc/pentane, 1:4).
IR ν_{max}/cm^{-1} (film) 1723, 1678, 1399, 1265, 1167, 743, 611.
^{1}H NMR (400 MHz, $CDCl_3$) δ 8.97 (1H, s, H2), 3.70 – 3.33 (4H, m, H4), 1.34 (6H, br. s, H5).
^{13}C NMR (101 MHz, $CDCl_3$) δ 148.5, 128.7, 128.4, 44.5, 12.4.
HRMS (ESI$^{+/-}$) Not found.

Step 4: Sodium 4-(diethylcarbamoyl)-4H- 1,2,4-triazole-3-sulfinate, ***2aa***

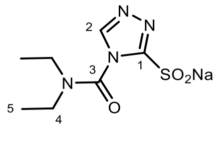

4-(Diethylcarbamoyl)-4H-1,2,4-triazole-3-sulfonyl chloride **2aa1** (738 mg, 2.77 mmol), sodium sulfite (700 mg, 5.54 mmol) and sodium bicarbonate (467 mg, 5.54 mmol) in water (5.0 mL) were subjected to the conditions of **general procedure J** at room temperature to give the sulfinate salt **2aa** (470 mg, 1.85 mmol, 67%) as white solid.

IR ν_{max}/cm^{-1} (film) 1703, 1435, 1273, 1056, 985.
^{1}H NMR (400 MHz, MeOD) δ 8.89 – 8.86 (1H, m, H2), 3.61 (4H, br. s, H4), 1.28 (6H, t, *J* = 7.1 Hz, H5).
^{13}C NMR (151 MHz, MeOD) δ 175.4, 150.7, 148.2, 44.8 (br.), 14.2 (br.).
HRMS (ESI$^{+/-}$) Not found.

4.6 Heteroaryl Sulfinate Salts

Sodium 5-bromothiophene-2-Sulfinate, 2r

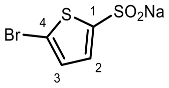

5-Bromothiophene-2-sulfonyl chloride (1.31 g, 5.00 mmol), sodium sulfite (1.26 g, 10.0 mmol) and sodium bicarbonate (840 mg, 10.0 mmol) in H_2O (5.0 mL) were subjected to the conditions of **general procedure J** to give the sulfinate salt **2r** (1.37 g, 5.53 mmol, quant.) as a pale-yellow solid. The product contained a 21% impurity by ^{1}H NMR ($ArSO_3H$) (87% calculated yield).

IR ν_{max}/cm^{-1} (film) 1404, 1199, 1062, 1049, 985, 956, 804, 656, 607.
^{1}H NMR (400 MHz, MeOD) δ 7.02 (1H, d, *J* = 3.8 Hz, H3), 7.00 (1H, d, *J* = 3.8 Hz, H2).
^{13}C NMR (151 MHz, MeOD) δ 165.0, 131.4, 126.6, 115.1.
HRMS (ESI$^-$) [M - Na]$^-$ $C_4H_2O_2{}^{79}BrS_2{}^-$ requires 224.8685; found 224.8681.

Sodium 1-methyl-1H-pyrazole-3-Sulfinate, 2s

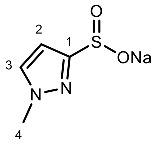

1-Methyl-1H-pyrazole-3-sulfonyl chloride (661 mg, 3.66 mmol), sodium sulfite (920 mg, 7.32 mmol) and sodium bicarbonate (613 mg, 7.32 mmol) in H_2O (5.0 mL) were subjected to the conditions of **general procedure J** to give the sulfinate salt **2s** (510 g, 3.04 mmol, 83%) as a white solid. The product contained a 25% impurity by ^{1}H NMR ($ArSO_3H$).

IR ν_{max}/cm^{-1} (film) 3391, 1510, 1230, 1113, 996, 968, 656, 638.
^{1}H NMR (400 MHz, MeOD) δ 7.64 (1H, br. s, H), 7.55 (1H, br. s, H), 3.88 (3H, s, H4).
^{13}C NMR (101 MHz, MeOD) δ 139.8, 137.4, 130.4, 38.9.
HRMS (ESI$^{-/+}$) Not found.

Sodium 1-methyl-1*H*-pyrazole-4-sulfinate, 2t

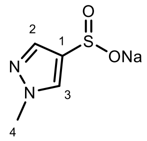

The conditions of **general procedure K** gave sulfinate salt **2t** (5.80 g, 0.0345 mol, 63%) as a white solid.

m.p. 253 – 255 °C.

^{1}H NMR (500 MHz, D_2O) δ 7.76 (1H, s, H2), 7.66 (1H, s, H3), 3.91 (3H, s, H4).

^{13}C NMR (151 MHz, D_2O) δ 135.9, 129.7, 38.3.

HRMS ($ESI^{+/-}$) Not found.

Lithium furan-3-sulfinate, 2u

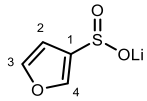

The conditions of **general procedure L** gave sulfinate salt **2u** (4.80 g, 0.035 mol, 70%) as a yellow solid.

^{1}H NMR (500 MHz, D_2O) δ δ 7.74 (1H, s, H3), 7.60 – 7.55 (1H, m, H4), 6.65 (1H, s, H2).

^{13}C NMR (126 MHz, D_2O) δ 144.3, 141.4, 139.3, 105.7.

HRMS (ESI^-) $[M]^-$ $C_4H_3O_3S^-$ requires 130.9808; found 130.9803.

Sodium 3,5-dimethylisoxazole-4-Sulfinate, 2v

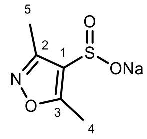

3,5-Dimethylisoxazole-4-sulfonyl chloride (978 mg, 5.00 mmol), sodium sulfite (1.26 g, 10.0 mmol) and sodium bicarbonate (840 mg, 10.0 mmol) in H_2O (5.0 mL) were subjected to the conditions of **general procedure J** to give the sulfinate salt **2v** (868 mg, 4.74 mmol, 94%) as a white solid.

IR ν_{max}/cm^{-1} (film) 3378, 1601, 1407, 1357, 1248, 1036, 1018, 973, 682.

^{1}**H NMR** (400 MHz, MeOD) δ 2.50 (3H, s, H4/5), 2.38 (3H, s, H4/5).

^{13}**C NMR** (101 MHz, MeOD) δ 168.6, 159.3, 128.3, 11.4, 10.7.

HRMS (ESI$^-$) [M - Na]$^-$ $C_5H_6O_3NS^-$ requires 160.0063; found 160.0065.

Sodium pyridine-3-Sulfinate, 2w

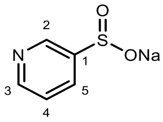

Pyridine-3-sulfonyl chloride (1.77 g, 10.0 mmol), sodium sulfite (2.52 g, 10.0 mmol) and sodium bicarbonate (1.68 g, 10.0 mmol) in water (10.0 mL) were subjected to the conditions of **general procedure J** to give the sulfinate salt **2w** (1.76 g, 10.7 mmol, quant.) as pale pink solid. The product contained a 5% impurity by ^{1}H NMR.

The lithium salt was also prepared under the conditions of **general procedure L** (2.46 g, 0.0165 mol, 61%) as a beige solid.

IR ν_{max}/cm^{-1} (film) 3281, 1696, 1412, 1049, 982, 706.

^{1}**H NMR** (400 MHz, MeOD) δ 8.76 (1H, dd, *J* = 2.1, 0.9 Hz, H2), 8.54 (1H, dd, *J* = 4.9, 1.6 Hz, H3), 8.10 – 8.04 (1H, m, H5), 7.50 (1H, ddd, *J* = 7.8, 4.9, 0.9 Hz, H4).

^{13}**C NMR** (101 MHz, MeOD) δ 153.4, 150.6, 147.1, 134.4, 125.3.

HRMS (ESI$^{+/-}$) Not found.

Lithium 6-methoxypyridine-3-sulfinate, 2x

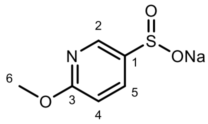

The conditions of **general procedure L** gave sulfinate salt **2x** (8 g, 0.045 mol, 90%) as a white powder.

^{1}**H NMR** (400 MHz, D_2O) δ 8.27 (1H, s, H2), 7.95 (1H, d, *J* = 7.0 Hz, H5), 6.97 (1H, d, *J* = 8.5 Hz, H4), 3.95 (3H, s, H6).

^{13}**C NMR** (126 MHz, D_2O) δ 164.5, 142.7, 142.5, 135.3, 110.6, 53.9.

HRMS (ESI$^{+/-}$) Not found.

Lithium 6-chloropyridine-3-sulfinate, 2y

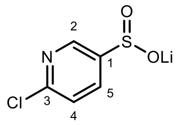

The conditions of **general procedure L** gave sulfinate salt **2y** (7.50 g, 0.041 mol, 82%) as a beige solid.

m.p. 261 – 263 °C.

^{1}H NMR (500 MHz, DMSO-d$_6$) δ 8.40 (1H, d, *J* = 1.6 Hz, H2), 7.86 (1H, dd, *J* = 8.0, 2.0 Hz, H5), 7.45 (1H, d, *J* = 8.0 Hz, H4).

^{13}C NMR (126 MHz, DMSO-d$_6$) δ 154.0, 149.3, 147.1, 136.4, 123.7.

HRMS (ESI$^-$) [M]$^-$ $C_5H_3ClNO_2S^-$ requires 175.9573; found 175.9577.

4.7 Alkyl Sulfinate Salts

Sodium cyclopropanesulfinate, 6f

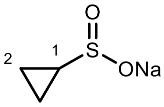

The conditions of **general procedure K** gave sulfinate **6f** (68.0 g, 0.53 mol, 75%) as a white solid.

m.p. 244 – 245 °C.

^{1}H NMR (400 MHz, DMSO-d_6) δ 1.62 – 1.45 (m, 1H), 0.48 – 0.43 (m, 2H), 0.29 – 0.24 (m, 2H).

^{13}C NMR (126 MHz, DMSO-d_6) δ 35.7, -0.4.

HRMS (ESI$^{+/-}$) Not found.

Sodium bicyclo[1.1.1]pentane-1-sulfinate, 6l

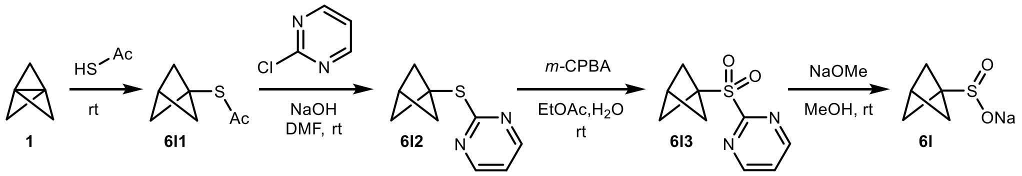

Step 1: S-(bicyclo[1.1.1]pentan-1-yl) ethanethioate, **6l1**

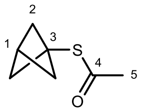

Thioacetic acid (10.50 g, 9.6 mL, 0.138 mol, 1.00 equiv.) was added to propellane (300 mL, 0.21 mol, 0.7 M in Et_2O, 1.50 equiv.) under Ar atmosphere in one portion. The mixture was stirred at room temperature for 15 h and concentrated under reduced pressure (30 – 15 mmHg, 35 °C). The residue was purified by vacuum distillation (b.p. = 43 °C, 10 mmHg) to give BCP **6l1** (16 g, 0.11 mmol, 83%) as a yellow oil.

^{1}H NMR (500 MHz, $CDCl_3$) δ 2.79 (1H, s, H1), 2.25 (3H, s, H5), 2.19 (6H, s, H2).

^{13}C NMR (126 MHz, $CDCl_3$) δ 196.7, 54.6, 42.2, 31.7, 31.4.

HRMS (ESI^{+}) $[M + H]^+$ $C_7H_{11}OS^+$ requires 143.0531; found 143.0522.

Step 2: 2-(Bicyclo[1.1.1]pentan-1-ylthio)pyrimidine, ***6l2***

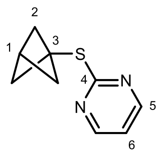

To a solution of *S*-bicyclo[1.1.1]pentan-1-yl ethanethioate **6l1** (10.00 g, 0.07 mol, 1.00 equiv.) in DMF (100 mL) was added 1 M aq. NaOH (70 mL, 0.07 mol, 1.00 equiv.) under Ar atmosphere at 0 °C. The resulting mixture was stirred for 2 h at room temperature. After that, a solution of 2-chloropyrimidine (8.00 g, 0.07 mol, 1.00 equiv.) in DMF (20 mL) was added, and the mixture was stirred at room temperature for 15 h. The mixture was poured in water (300 mL), extracted with MTBE (3 × 100 mL). The combined organic layers were washed with brine (5 × 200 mL), dried over Na_2SO_4 and concentrated under reduced pressure (30 – 15 mmHg, 35 °C). The residue was purified by column chromatography (SiO_2, hexane/MTBE, 7:3) to give **6l2** (8.60 g, 0.048 mol, 69%) as a yellow oil.

R_f 0.58 (hexane/EtOAc, 7:3).
^{1}H NMR (500 MHz, DMSO-d_6) δ 8.60 (2H, d, *J* = 4.8 Hz, H5), 7.18 (1H, t, *J* = 4.8 Hz, H6), 2.87 (1H, s, H1), 2.24 (6H, s, H2).
^{13}C NMR (151 MHz, DMSO-d_6) δ 171.7, 157.6, 117.4, 53.5, 42.0, 31.0.
HRMS ($ESI^{+/-}$) Not found.

Step 3: 2-(Bicyclo[1.1.1]pentan-1-ylsulfonyl)pyrimidine, **6l3**

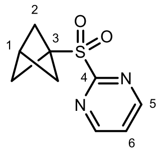

To a solution of 2-(bicyclo[1.1.1]pentan-1-ylthio)pyrimidine **6l2** (5.00 g, 0.023 mol, 1.00 equiv.) in a mixture of EtOAc and H_2O (1:1, 100 mL) was added *m*CPBA (11 g, 0.05 mol, 77% purity, 2.20 equiv.) in three portions at 0 – 5 °C. The mixture was stirred at room temperature for 15 h and diluted with water (100 mL). The aqueous layer was extracted with EtOAc (2 × 50 mL). The combined organic layers were washed with a saturated solution of $NaHCO_3$ (10 × 100 mL), dried over Na_2SO_4 and concentrated under reduced pressure (30 – 15 mmHg, 25 °C). The residue was purified by column chromatography (SiO_2, hexane/EtOAc, 7:3) to give **6l3** (3.70 g, 0.018 mol, 78%) as a white solid.

R$_f$ 0.60 (hexane/EtOAc, 7:3).

^{1}H NMR (500 MHz, DMSO-d$_6$) δ 9.10 (2H, d, *J* = 4.9 Hz, H5), 7.87 (1H, t, *J* = 4.9 Hz, H6), 2.81 (1H, s, H1), 2.21 (6H, s, H2).

^{13}C NMR (151 MHz, DMSO-d$_6$) δ 164.3, 159.1, 124.7, 53.1, 50.9, 27.2.

HRMS (ESI$^+$) [M + H]$^+$ $C_9H_{11}N_2O_2S$ requires 211.0541 found 211.0528.

Step 4: Sodium bicyclo[1.1.1]pentane-1-sulfinate, ***6l***

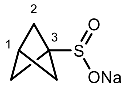

To a solution of 2-(bicyclo[1.1.1]pentan-1-ylsulfonyl)pyrimidine **6l3** (3.00 g, 0.014 mol, 1.00 equiv.) in dry MeOH (50 mL) was added NaOMe (13 mL, 1 M in MeOH, 0.013 mol, 0.95 equiv.) in one portion under Ar atmosphere at room temperature. The resulting mixture was stirred at room temperature for 15 h and concentrated under reduced pressure (30 – 15 mmHg, 25 °C). The residue was triturated (Et_2O), filtered and dried under reduced pressure (1 mmHg, 35 °C) to give **6l** (1.35 g, 8.80 mmol, 63%) as a white powder.

^{1}H NMR (500 MHz, DMSO-d$_6$) δ 1.61 (6H, s, H2).

^{13}C NMR (126 MHz, DMSO-d$_6$) δ 46.7, 25.3.

HRMS (ESI$^{+/-}$) Not found.

4.8 Unsuccessful Sulfinates

The following sulfinate salts were unsuccessful under our reaction conditions and reasonable modifications. The were either obtained from commercial sources,[14] prepared accoding to **general procedure J** or literature procedures.[15] [16] Typical limitations included de-sulfonylation of the sulfonyl halide intermediate (even at low temperature), significant formation of di-iodinated BCP **S1** or purification challenges.

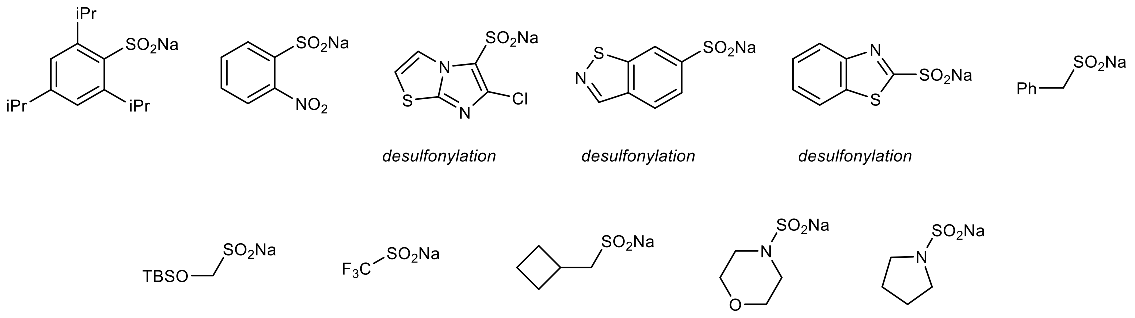

Note: Trifluoromethanesulfinate and cyclobutylmethanesulfinate appeared somewhat successful but suffered from purification challenges.

Sodium 2,4,6-triisopropylbenzenesulfinate

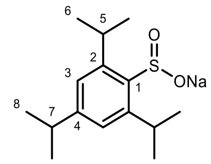

2,4,6-Triisopropylbenzenesulfonyl chloride (1.50 g, 5.00 mmol), sodium sulfite (1.26 g, 10.0 mmol) and sodium bicarbonate (840 mg, 10.0 mmol) in water (5.0 mL) were subjected to the conditions of **general procedure J** to give the sulfinate salt (890 mg, 2.96 mmol, 59%) as a white solid. The product contained a 45% impurity by ^{1}H NMR ($ArsO_3H$).

IR ν_{max}/cm^{-1} (film) 2960, 2159, 1210, 1188, 1089, 1051, 1019, 977, 684, 640.

^{1}H NMR (600 MHz, MeOD) δ 7.09 (2H, s, H3), 4.49 (2H, hept, *J* = 6.8 Hz, H5), 2.90 – 2.80 (1H, m, H7), 1.26 (6H, d, *J* = 6.9 Hz, H8), 1.23 (12H, obsc. d, *J* = 6.9 Hz, H6).

^{13}C NMR (151 MHz, MeOD) δ 150.9, 149.4, 139.9, 123.5, 35.3, 30.4, 25.2, 24.2.

HRMS (ESI$^-$) [M - Na]$^-$ $C_{15}H_{23}O_2S^-$ requires 267.1424; found 267.1422.

Sodium 2-nitrobenzenesulfinate

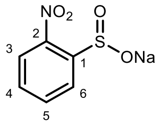

2-Nitrobenzenesulfonyl chloride (1.11 g, 5.00 mmol), sodium sulfite (1.26 g, 10.0 mmol) and sodium bicarbonate (840 mg, 10.0 mmol) in water (5.0 mL) were subjected to the conditions of **general procedure J** to give the sulfinate salt (447 mg, 2.14 mmol, 43%) as an orange solid.

IR ν_{max}/cm^{-1} (film) 2550, 2361, 1537, 1372, 1206 (br.), 1143, 1027, 615.
^{1}H NMR (400 MHz, MeOD) δ 8.07 – 8.04 (1H, m, H3), 7.67 – 7.57 (3H, m, H4 – 6).
^{13}C NMR (101 MHz, MeOD) δ 158.6, 132.4, 130.5, 128.5, 124.4.
HRMS (ESI+/-) Not found.

Sodium 6-chloroimidazo[2,1-b]thiazole-5-Sulfinate

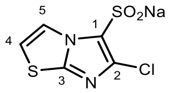

6-Chloroimidazo[2,1-b]thiazole-5-sulfonyl chloride (500 mg, 1.95 mmol), sodium sulfite (492 mg, 3.91 mmol) and sodium bicarbonate (328 mg, 3.91 mmol) in H_2O (5.0 mL) were subjected to the conditions of **general procedure J** to give the sulfinate salt (409 mg, 1.68 mmol, 86%) as a white solid.

IR ν_{max}/cm^{-1} (film) 1454, 1235, 1210, 1165, 1011, 959, 657.
^{1}H NMR (400 MHz, MeOD) δ 7.64 (1H, s, H5), 7.55 (1H, s, H4).
^{13}C NMR (101 MHz, MeOD) δ 139.9, 138.4, 137.4, 131.5, 130.4, 49.0.
HRMS (ESI$^{+/-}$) Not found.

Sodium benzo[*d*]isothiazole-6-Sulfinate

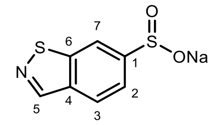

Benzo[d]isothiazole-6-sulfonyl chloride (1.00 g, 4.28 mmol), sodium sulfite (1.08 g, 8.56 mmol) and sodium bicarbonate (719 mg, 8.56 mmol) in H_2O (4.3 mL) were subjected to the conditions of **general procedure J** to give the sulfinate salt (475 mg, 2.15 mmol, 50%) as a white solid.

IR ν_{max}/cm^{-1} (film) 2160, 1220, 1195, 1144, 1128, 1056, 886, 814, 687, 663.
^{1}H NMR (600 MHz, MeOD) δ 9.35 (1H, s, H5), 8.54 (1H, dd, *J* = 1.7, 0.6 Hz, H7), 8.12 (1H, dd, *J* = 8.6, 0.6 Hz, H3), 8.01 (1H, dd, *J* = 8.6, 1.7 Hz, H2).
^{13}C NMR (151 MHz, MeOD) δ 159.2, 155.0, 144.2, 134.7, 125.6, 123.7, 121.3.
HRMS (ESI$^{+/-}$) Not Found.

Sodium Benzo[*d*]thiazole-2-Sulfinate

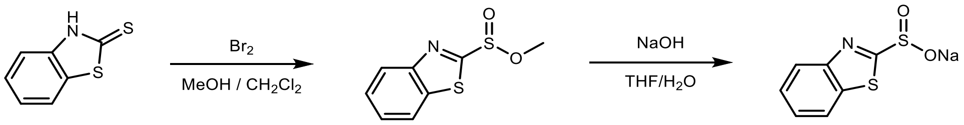

Step 1: Methyl benzo[d]thiazole-2-sulfinate

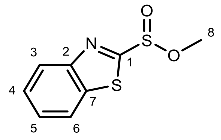

According to a modified literature procedure.[17] Sulfide (3.00 g, 18.0 mmol) was dissolved in dry CH_2Cl_2 (75 mL) and MeOH (75 mL) in a 500 ml conical flask. Bromine (2.31 mL, 45.0 mmol) was added dropwise and the reaction was stirred for 10 min. The reaction was quenched with sat. aq. $NaHCO_3$ (75 mL) and then extracted with CH_2Cl_2 (3 × 100 mL). The combined organic layers were washed with sat. aq. $Na_2S_2O_3$, dried over $MgSO_4$, filtered and concentrated *in vacuo*. The residue was purified by column chromatography (SiO_2, EtOAc/pentane, 1:9) to give the methyl sulfinate (3.26 g, 14.5 mmol, 81%) as an orange solid.

R_f 0.56 (EtOAc/pentane, 1:9).

^{1}H NMR (400 MHz, $CDCl_3$) δ 8.18 (1H, ddd, *J* = 8.1, 1.4, 0.7 Hz, H3/6), 8.01 (1H, ddd, *J* = 7.1, 1.4, 0.7 Hz, H3/6), 7.64 – 7.50 (2H, m, H4, H5), 3.74 (3H, s, H8).
^{13}C NMR (101 MHz, $CDCl_3$) δ 175.0, 153.8, 136.2, 127.3, 127.3, 125.1, 122.5, 51.5.

Step 2: Sodium benzo[d]thiazole-2-sulfinate

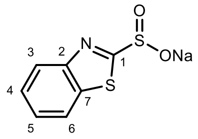

Methyl sulfinate was dissolved in THF (9.0 mL) and a solution of NaOH (2.0 M aq., 9.0 mL, 18.0 mmol) was added slowly at room temperature. The mixture was stirred for 10 min and checked for conversion of methyl ester by TLC. Addition of an additional portion of NaOH (2.0 M aq., 1.50 mL, 3 mmol) was added for complete conversion. The mixture was concentrated *in vacuo* and remaining H_2O was azeotroped with EtOH. The resulting pale-yellow solid was washed with Et_2O then dried under high vacuum overnight to give the sulfinate salt (3.42 g, 15.5 mmol, 86%, 2 steps) as an off-white solid.

^{1}H NMR (400 MHz, MeOD) δ 8.05 – 7.96 (2H, m, H3, H6), 7.52 (1H, ddd, *J* = 8.1, 7.2, 1.3 Hz, H4/5), 7.45 (1H, ddd, *J* = 7.9, 7.2, 1.3 Hz, H4/5).
^{13}C NMR (101 MHz, MeOD) δ 191.0, 154.7, 136.7, 127.4, 127.0, 124.2, 123.6.

Sodium morpholine-4-Sulfinate

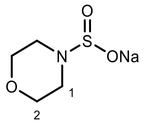

Morpholine-4-sulfonyl chloride (928 mg, 5.00 mmol), sodium sulfite (1.26 g, 10.0 mmol) and sodium bicarbonate (840 mg, 10.0 mmol) in H_2O (5.0 mL) were subjected to the conditions of **general procedure J** to give the sulfinate salt (411.4 mg, 2.38 mmol, 48%) as a white solid. The product contained a 13% impurity by ^{1}H NMR.

IR ν_{max}/cm^{-1} (film) 3403, 2962, 2724, 1102, 1080, 871.
^{1}H NMR (400 MHz, MeOD) δ 3.93 – 3.86 (4H, m, H), 3.26 – 3.18 (4H, m, H).
^{13}C NMR (101 MHz, MeOD) δ 67.4, 65.0, 47.8, 44.7.

HRMS ($ESI^{+/-}$) Not found.

Sodium pyrrolidine-1-Sulfinate

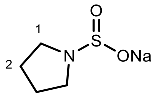

Pyrrolidine-1-sulfonyl chloride (848 mg, 5.00 mmol), sodium sulfite (1.26 g, 10.0 mmol) and sodium bicarbonate (840 mg, 10.0 mmol) in H_2O (5.0 mL) were subjected to the conditions of **general procedure J** to give the sulfinate salt (517 mg, 3.29 mmol, 66%) as a colourless solid. The product contained an 18% impurity by ^1H NMR.

IR ν_{max}/cm^{-1} (film) 3395, 1458, 1037, 669.

^{1}H NMR (400 MHz, MeOD) δ 3.30 – 3.22 (4H, m, H1), 2.04 – 1.97 (4H, m, H2).

^{13}C NMR (101 MHz, MeOD) δ 46.6, 25.1.

HRMS ($ESI^{+/-}$) Not found.

5. Additional Optimisation

5.1 Sulfonyl BCP Iodide Optimisation

Iodinating Reagent Optimisation

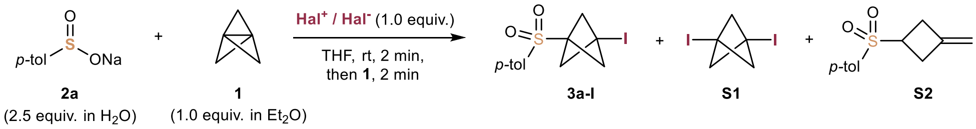

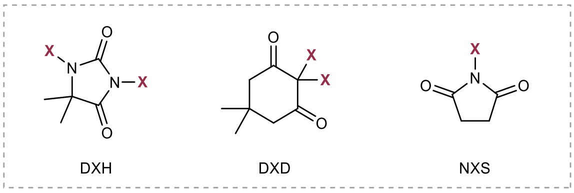

Entry	$I^{+/-}$ *source*	*Solvent*	***3a-I** NMR Yield /%*	***S1** NMR Yield /%*	***S2** NMR Yield /%*
1	I_2	THF	7	18	0
2	NIS	THF	41	9	0
3	ICl[a]	-	49	0	16
4	DIH	THF	78	13	0
5	IPy_2BF_4	THF	9	24	0
6	IPy_2BF_4	MeCN	2	2	52
7	DID	THF	31	6	27
8	NaI	THF	0	0	0
9	NaI	H_2O	0	0	0

***1** was prepared as a stock solution [0.70 M in Et_2O] throughout. [a] [1.0 M in CH_2Cl_2].*

Reaction Stoichiometry Optimisation

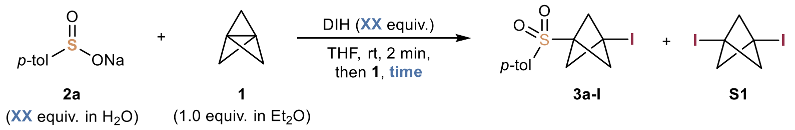

Entry.	*2a* equiv.	DIH equiv.	Time /min	*3a-I* NMR Yield /%	*S1* NMR Yield /%
1	0.00	1.0	2	0	18
2	1.00	1.0	2	35	29
3	1.50	1.0	2	58	34
4	2.00	1.0	2	73	22
5	2.50	1.0	2	78	13
6	3.00	1.0	2	87	12
7	2.50	0.5	2	38	10
8	2.00	1.0	15	71	29

Solvent Optimistion

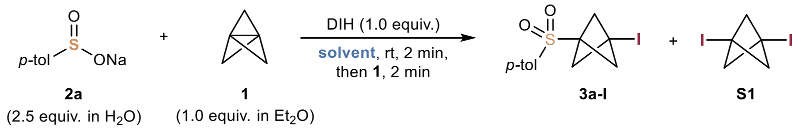

Entry	Solvent	Conc. of DIH /M	*3a-I* NMR Yield /%	*S1* NMR Yield /%
1	THF	1.0	78	13
2	THF	0.25	54	38
3	MeCN	1.0	74	19
4	Et_2O	1.0	quant.	1
5	H_2O	1.0	99	1
6	CH_2Cl_2	1.0	quant.	0
7	MeOH	1.0	83	12

Et_2O was carried forward as the solvent of choice to maintain consistency with the [1.1.1]propellane **1** stock solution (0.70 – 0.75 M in Et_2O). CH_2Cl_2 was retained as a solvent for further investigation.

Secondary Stoichiometry Optimisation

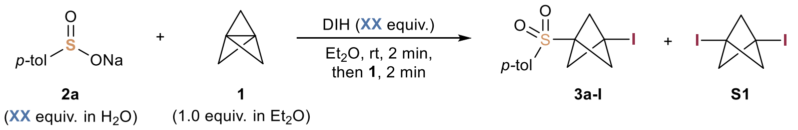

Entry	***2a** equiv.*	*DIH equiv.*	***3a-I** NMR Yield /%*	***S1** NMR Yield /%*
1	2.50	1.00	quant.	1
2	2.00	1.00	98	4
3	1.50	1.00	90	7
4	2.00	0.80	77	1
5	1.50	0.60	73	1
6	1.00	0.40	51	1

Reducing the equivalents of sulfinate salt **3a-S** from 2.5 to 1.5, maintained an excellent yield of sulfonyl BCP iodide **3a-I**, only dropping 10 % (entries 1-3). However, the BCP di-iodide **S1** (inseparable) impurity increased by a comparable amount, so to minimise purification requirements we continued with 2.5 equiv. of the sulfinate salt (entry 1).

5.2 Sulfonyl BCP Bromides and Chlorides

Reaction Time

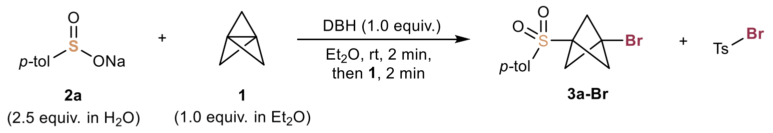

Entry	*Br+ Reagent*	*Time*	***3a-Br** NMR Yield /% (isolated)*	***TsBr** NMR Yield /%*
1	DBH	2 min	9	77
2	DBH	3 h	54	38
3	DBH	6 h	69	4
4	DBH	18 h	quant. (99)	0
5	NBS	18 h	58	-

In contrast to the reactivity of sulfonyl iodides, in a reaction time of 2 min, only a 9% yield of BCP bromide **3a-Br** was obtained (entry 1). Tosylbromide **3a-Br** was detected in the crude reaction mixture and increasing the reaction time to 18 h led to complete conversion (entries 1 – 4).

Sulfonyl BCP Chlorides

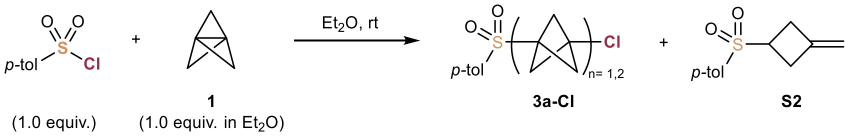

Entry	*Time*	***3a-Cl**, (n = 1) NMR Yield /%*	***3a-Cl**, (n = 2) NMR Yield /%*	***S2** NMR Yield /%*
1	2 min	0	0	7
2	3 h	14	9	59
3	6 h	14	9	-
4	18 h	29	-	39

Sulfonyl BCP chloride **3a-Cl** could be obtained, albeit in low yield. Higher proportions of staffane and rearranged by-product **S2** were formed (entries 1 – 4).

5.3 Instability of Sulfonyl Iodides

Solvent and Concentration Effects

Over the course of the reaction, tosyl iodide precipitated out of the reaction as a yellow solid. We anticipated that the stability of sulfonyl iodides would improve if it remained in solution (by dilution or use of more polar solvents). Therefore, we used a suboptimal set of reaction conditions (2.0 equiv. of **2a**) to investigate the effect of the concentration of the solvent on the reaction outcome (see table below). Dilution of the iodinating reagent from 1.00 M to 0.25 M improved the yield of **3a-I** from 74% to 89% (entries 1 and 2). MTBE is a more attractive solvent than Et_2O for industrial scale-up due to its higher flash point; however, its use was not productive (entry 3). Switching to CH_2Cl_2 maintained an excellent yield of 89% and further reduced the formation of BCP di-iodide **S1** (entry 4).

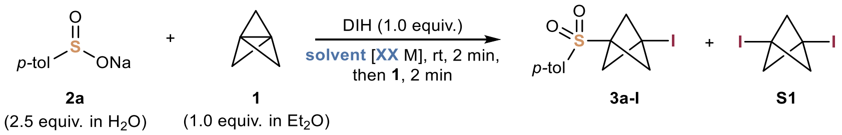

Entry	*Solvent*	*Conc. of DIH /M*	***3a-I** NMR Yield /%*	***S1** NMR Yield /%*
1	Et_2O	1.00	74	1
2	Et_2O	0.25	89	2
3	MTBE	0.25	7	14
4	CH_2Cl_2	0.25	89	<1

Electron Deficient Substrates

On application of the optimal conditions to an electron-deficient sulfinate salt **2n** (3,5-dCF_3PhSO_2Na), a significantly reduced yield of 23% of **3n-I** was observed (entry 1). Reduced temperature of -5 °C, CH_2Cl_2 as the solvent and dilution of the reaction improved the yield up to 47% (entries 2 – 4). Cooling to -40 °C (with DMF to prevent freezing) gave an optimal 69% yield of **3n-I** and the reaction was gradually warmed to room temperature over 30 mins to

ensure complete conversion (entry 5). Reducing the temperature to -78 °C required IPA or MeOH as the co-solvent, which led to significantly reduced yields (entries 6 and 7).

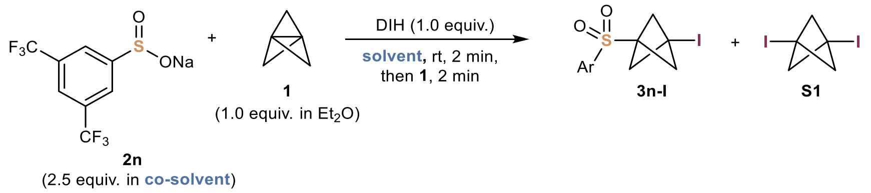

Entry	*Co-solvent*	*Solvent*	*Conc. of DIH/ M*	*Temp/ °C*	*Time/ min*	***3n-I*** *NMR Yield /%*	***S1*** *NMR Yield /%*
1	H_2O	Et_2O	1.00	25	2	23	21
2	H_2O	Et_2O	1.00	-5	2	24	25
3	H_2O	CH_2Cl_2	1.00	-5	2	31	9
4	H_2O	CH_2Cl_2	0.25	-5	2	47	5
5	DMF	CH_2Cl_2	0.25	-40	30	69	0
6	IPA	CH_2Cl_2	0.25	-78	30	40	6
7	MeOH	CH_2Cl_2	0.25	-78	30	16	2

5.4 Alkyl Sulfonyl Bromides Optimisation

The reaction of methoxypropanoate sulfinate **6g** under standard halo-bicyclopentylation conditions returned the sulfonylated hydantoin reagent **S3** as a significant product.

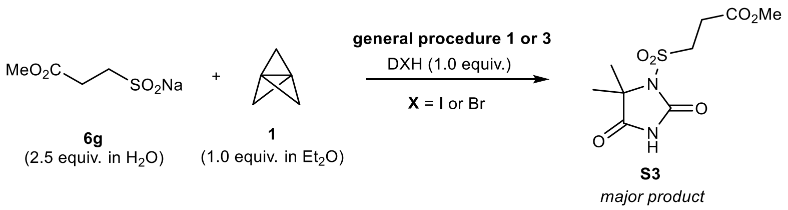

Optimisation of Alkyl Sulfonyl Bromide Formation

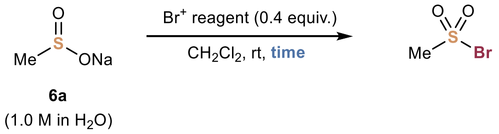

Entry	*Br⁺ Reagent*	*Time*	***MsBr** NMR yield /%*
1	Br_2	2 min	27
2	PTAB ($PhNMe_3^+$ Br_3^-)	2 min	26
3	TBAB ($Bu_4N^+Br^-$)	2 min	0
4	DBH	2 min	19
5	$PyrH^+Br_3^-$	2 min	9
6	$Bu_4N^+Br_3^-$	2 min	4
7	NBrSac[18]	2 min	0
8	Br_2	18 h	0

Bromine was found to be the superior brominating agent, giving mesyl bromide in 27% yield within a 2 min reaction time (entry 1). PTAB ($PhNMe_3^+Br_3^-$) gave a similarly promising result and was retained as a backup (entry 2). All other brominating reagents screened were less successful in the reaction (entries 3 – 7). Leaving the reaction for 18 h resulted in a 0% yield of mesyl bromide due to decomposition overnight (entry 8).

Stiochiometry Optimisation

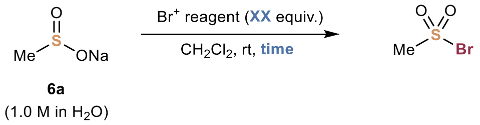

Entry	Br^+ Source	Equiv.	Time	**MsBr** NMR yield /%
1	Br_2	0.4	2 min	27
2	Br_2	0.8	2 min	63
3	PTAB	0.8	2 min	59
4	PTAB	0.8	15 min	59

Increasing the equivalents of bromine (from 0.4 to 0.8 equiv.) gave an improved 63% yield of mesyl bromide (entries 1 and 2). However, re-evaluation of PTAB as a brominating agent with this new stoichiometry did not offer further improvements (entry 3). No degradation of mesyl bromide was observed between a 2 min and 15 min reaction time (entries 3 and 4).

Alkyl Sulfonyl BCP Bromide Optimisation

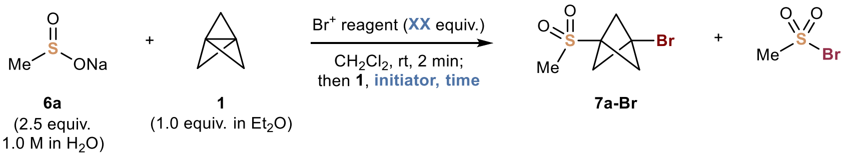

Entry	Br^+ reagent	Br^+ equiv.	Initiator[a]	Time	**7a-Br** NMR Yield /% (isolated)	**7a-Br** NMR Yield /%
1	Br_2	1.0	-	18 h	0	0
2	Br_2	1.0	Et_3B	18 h	30	-
3	Br_2	2.0	Et_3B	30 min	76	39
4	PTAB	2.0	Et_3B	30 min	17	40
5	Br_2	2.0	Et_3B	1 h	81	50
6	Br_2	2.0	Et_3B	2 h	94 (96)	51
7	Br_2	2.0	Et_3B	2.5 h	94	36

*[a] Et_3B in 10 mol%. [1.1.1]propellane **1** was prepared as a 0.70 M solution in Et_2O.*

Addition of triethylborane as a radical initiator was required to obtain BCP bromide **7a-Br** in 30% yield (entries 1 and 2). Use of 2.0 equiv. of Br_2 increased the yield to 76% in only 30 min reaction time (entry 3), and a 2 hour reaction time was later found to be optimal (96%, entries 5 – 7).

Stiochiometry Optimisation

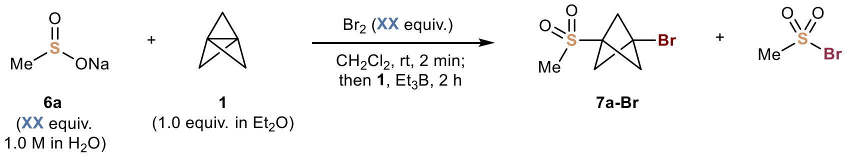

Entry	***6a** equiv.*	*Br_2 Equiv.*	***7a-Br** NMR Yield /% (isolated)*	***MsBr** NMR Yield /%*
1	2.5	2.0	94 (96)	51
2	2.0	1.6	86 (86)	6
3	2.0	1.8	99 (98)	-

The current best conditions isolated 96% of BCP bromide **7a-Br** along with 51% of unreacted mesyl bromide (entry 1). Gratifyingly, the use of 1.8 equiv. of Br_2 gave a near quantitative yield of **7a-Br** with just 2.0 equiv. of sulfinate (entries 2 and 3).

5.5 Alkyl Sulfonyl Iodides Optimisation

Iodinating Reagent Optimisation

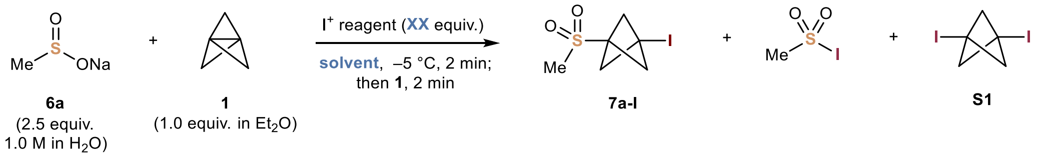

No.	*I^+ source*	*I^+ equiv.*	*Solvent*	***7a-I** NMR Yield /% (isolated)*	***MsI** NMR Yield /% [a]*	***S1** NMR Yield /%*
1	I_2	1.0	Et_2O	28	-	50
2	$BnMe_3N^+ICl_2^-$	2.5	CH_2Cl_2	83 (92)	109	2

*[a] NMR Yield of mesyl iodide is out of 250% based on reaction stoichiometry. [1.1.1]propellane **1** was prepared as a 0.75 M solution in Et_2O.*

$BnMe_3NICl_2$ was found to be an excellent iodinating agent for this transformation and sulfonyl BCP iodide **7a-I** was isolated in 92% yield in just 2 min (entries 1 and 2). However, a significant proportion of unreacted mesyl iodide was observed, indicating the amount of iodinating agent and sulfinate salt could be reduced.

Reaction Stoichiometry Optimisation

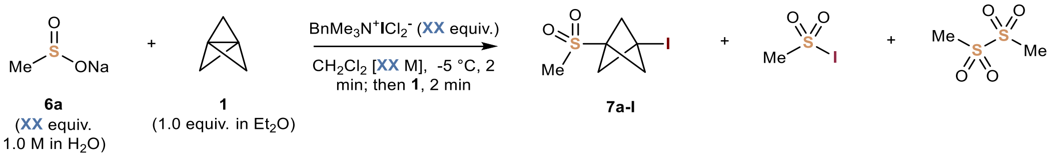

Entry	*$BnMe_3N^+ICl_2^-$ equiv.*	***6a*** *equiv.*	*Conc. /M*	***7a-I*** *NMR Yield /% (isolated)*	***MsI*** *NMR Yield /%* [a]	***Ms-Ms*** *NMR Yield /%*
1	2.5	2.5	1.0	83 (92)	109	7
2	1.8	2.5	1.0	71	57	0
3	1.4	2.5	1.0	102	24	0
4	1.4	2.0	1.0	102 (99)	27	2
5	1.4	1.5	1.0	77	35	7
6	1.4	2.0	0.25	39	24	23

The yield of di-iodinated BCP ***S1*** *was <2 % for all entries. [1.1.1]propellane* ***1*** *was prepared as a 0.75 M solution in Et_2O.*

The reaction efficiency was improved by using 1.4 equiv. of $BnMe_3N^+ICl_2^-$ and just 2.0 equiv. of sulfinate salt, obtaining BCP iodide **7a-I** in an excellent 99% yield (entries 1 –5).

5.6 Control Reactions and Reaction Monitoring

Control Reactions

To gain insight into the initiation process, we carried out several control experiments (see table below). Firstly, carrying out the iodo-sulfonylation of [1.1.1]propellane **1** in the dark led to no formation of tosyl iodide or BCP iodide **3a-I** (entry 1). Secondly, the addition of TEMPO as a radical inhibitor completely shut down the desired reactivity (entry 2). Analysis by LCMS and ^{1}H NMR observed no TEMPO-tosyl adducts. These results indicate that ambient light is necessary to initiate a radical process on this time scale. Thirdly, the omission of [1.1.1]propellane **1** led to the formation of sulfone dimer (entry 3). These observations are consistent with a radical mechanism following visible light homolysis of the S—Hal bond.

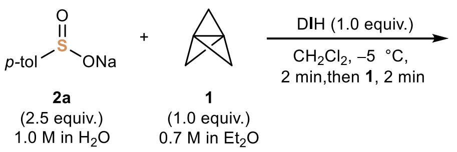

Entry	*Variation*	*Result*
1	Dark	No reaction
2	TEMPO (3.0 equiv.)	No reaction
3	Omission of **1**	Ts-Ts major product

Reaction Monitoring

^{1}H NMR monitoring the progress of the halo-sulfonylation of [1.1.1]propellane **1** over time showed that the addition of sulfonyl bromides required over 6 h and was complete after 18 h reaction time (see graph below) in comparison to the near-instant reaction of sulfonyl iodides. As expected, sulfonyl chlorides reacted significantly slower.

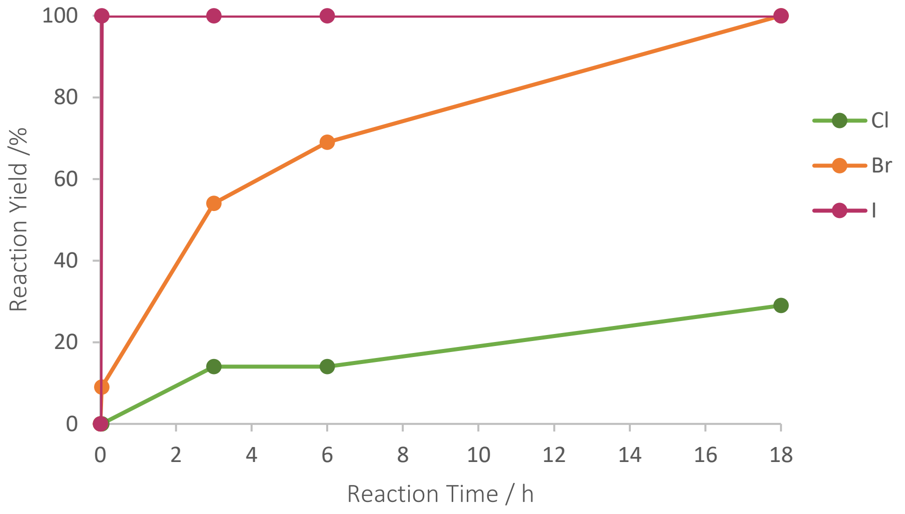

*Reaction progression for the formation of sulfonyl BCP halides **3a-I**, **3a-Br** and **3a-Cl** from the corresponding sulfinate salt/sulfonyl halide under optimised conditions.*

6. NMR Spectra

6.1 Aryl Sulfonyl BCP Iodides

1-Iodo-3-tosylbicyclo[1.1.1]pentane, 3a-I

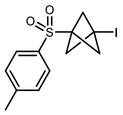

^{1}H NMR (400 MHz, $CDCl_3$)

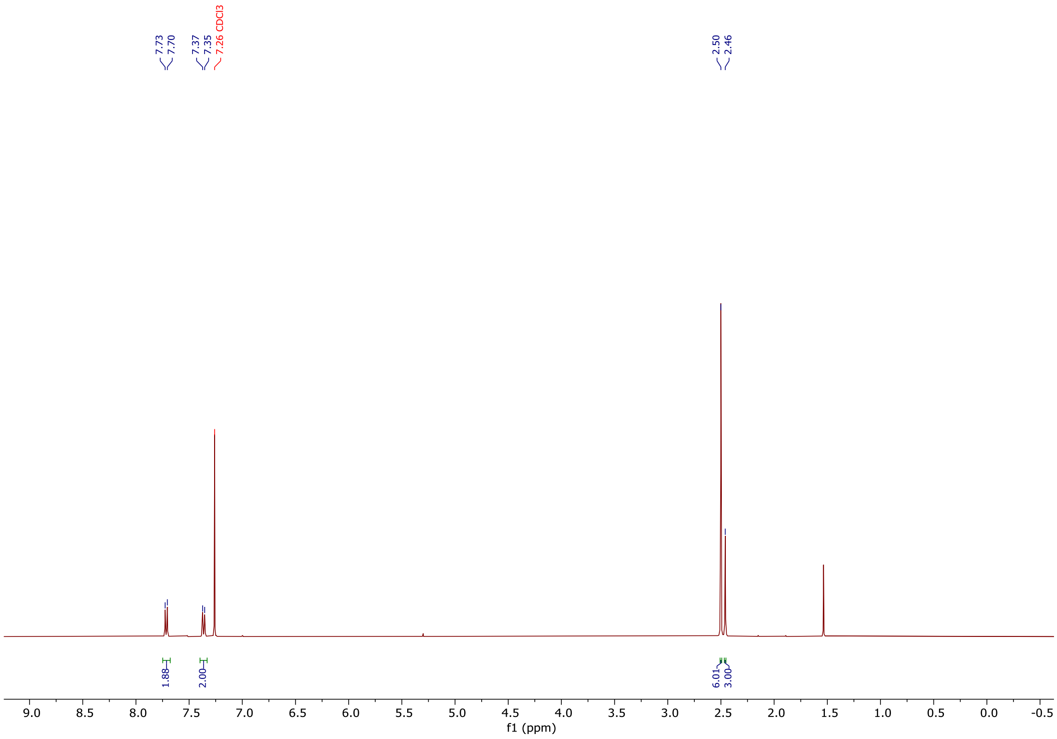

^{13}C NMR (101 MHz, $CDCl_3$)

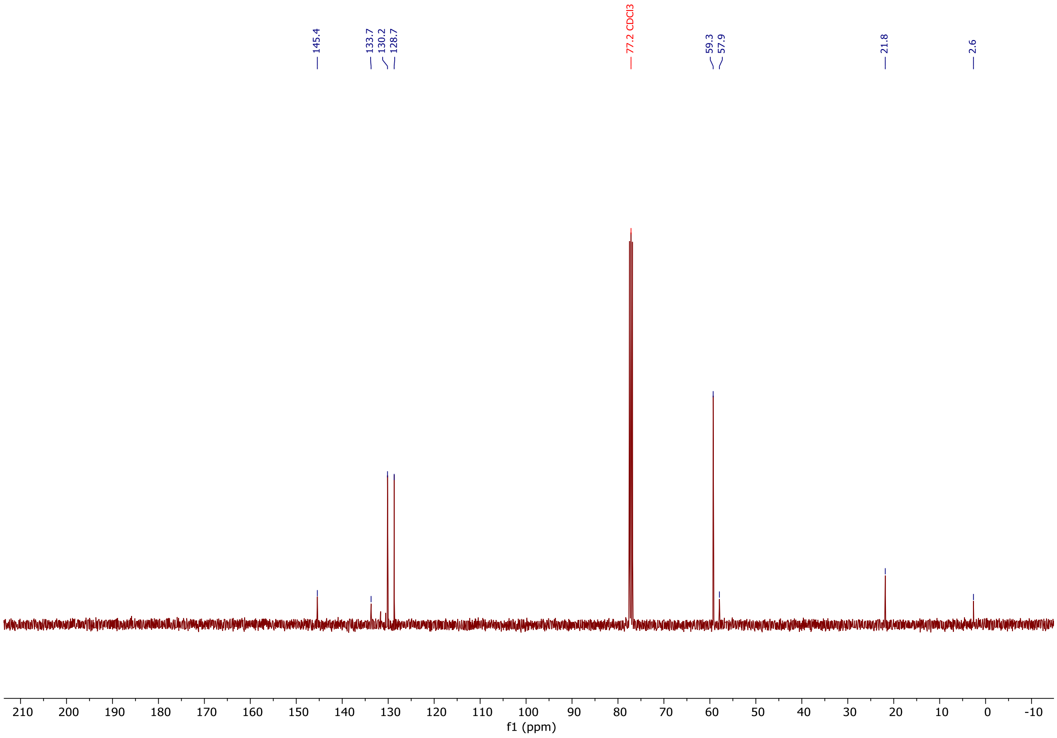

1-Bromo-3-tosylbicyclo[1.1.1]pentane, 3a-Br

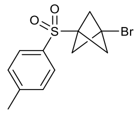

^{1}H NMR (400 MHz, $CDCl_3$)

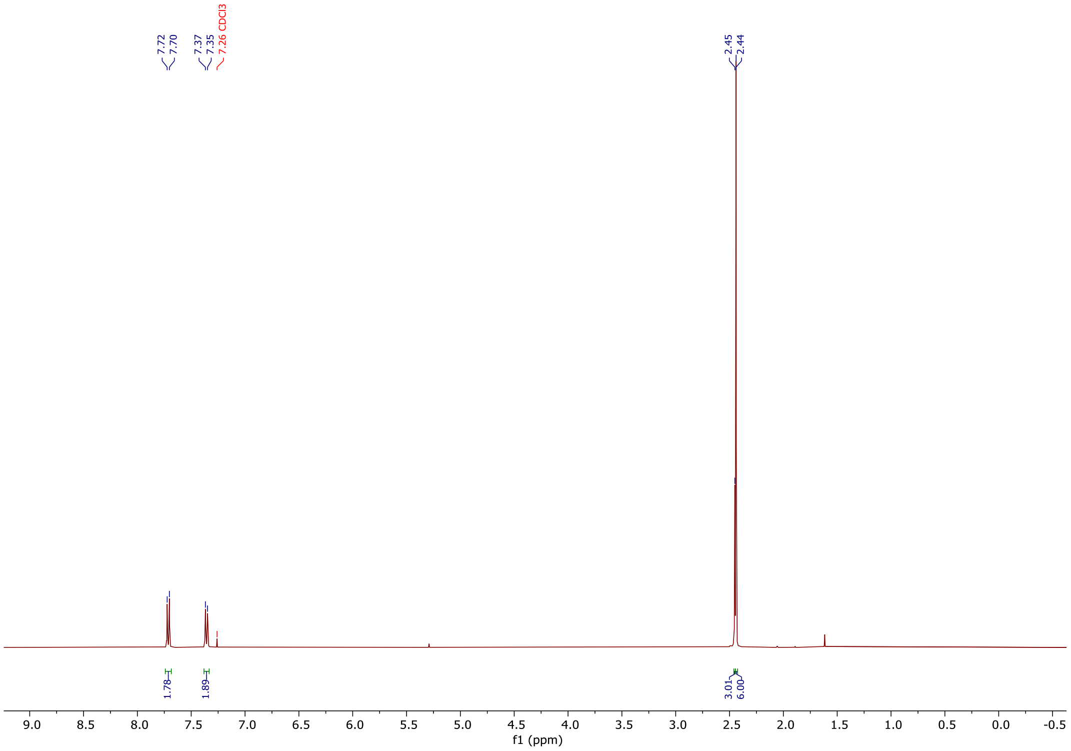

^{13}C NMR (101 MHz, $CDCl_3$)

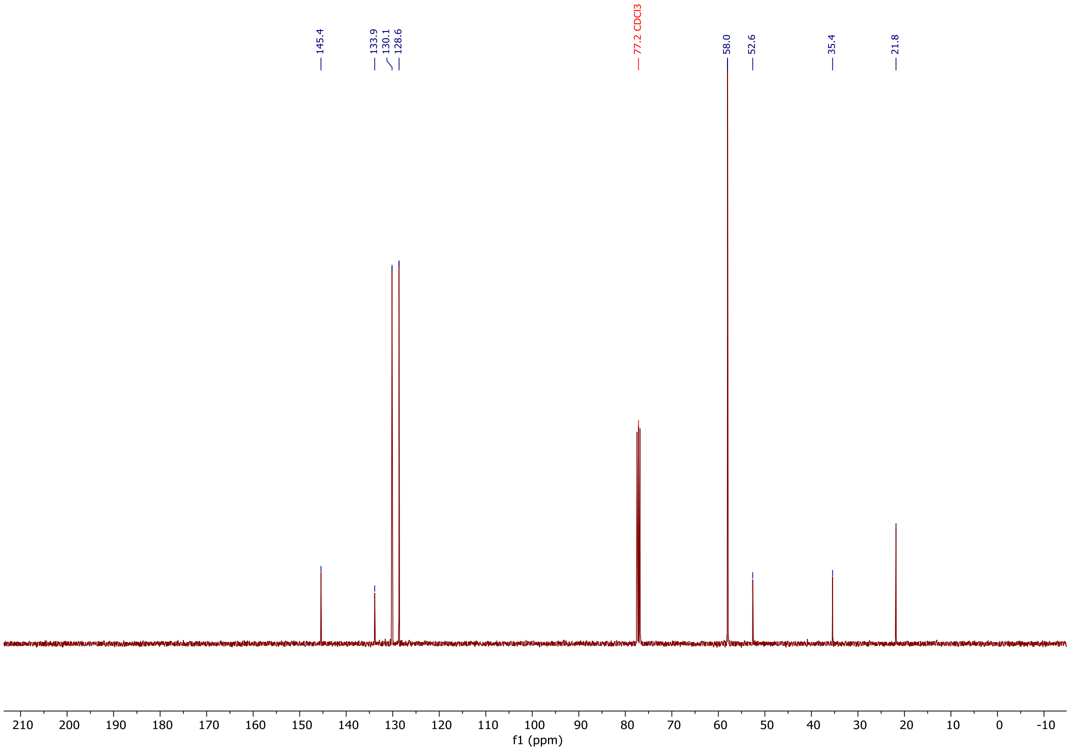

1-Chloro-3-tosylbicyclo[1.1.1]pentane, 3a-Cl

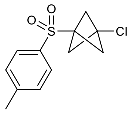

^{1}H NMR (400 MHz, $CDCl_3$)

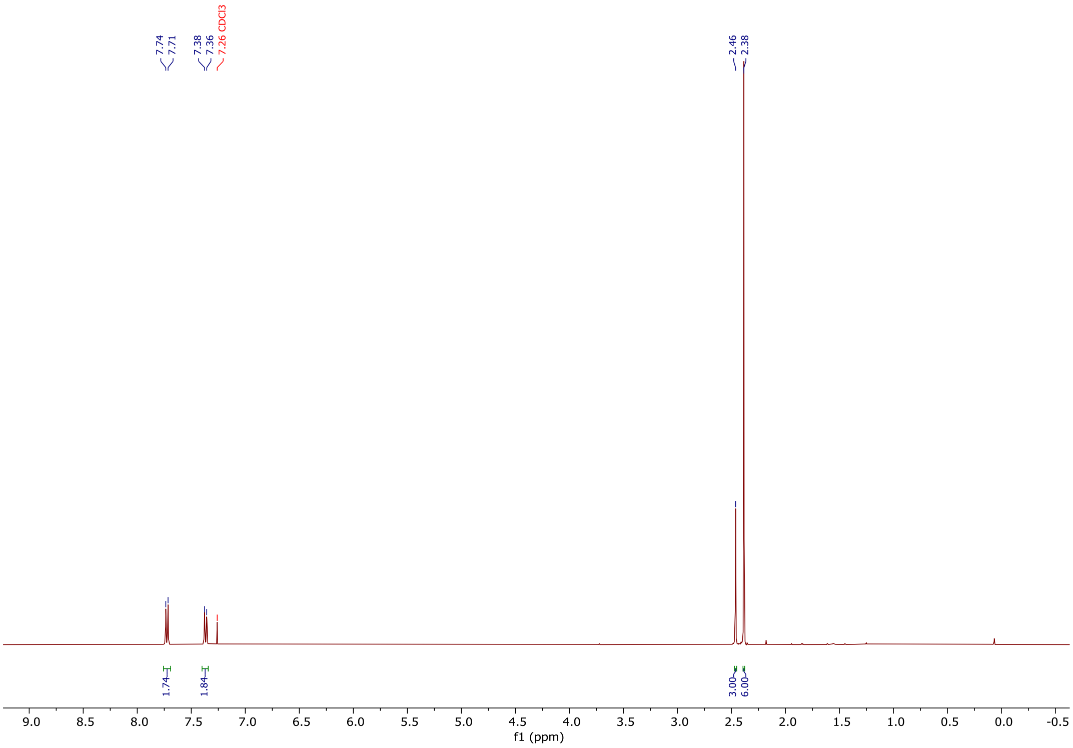

^{13}C NMR (101 MHz, $CDCl_3$)

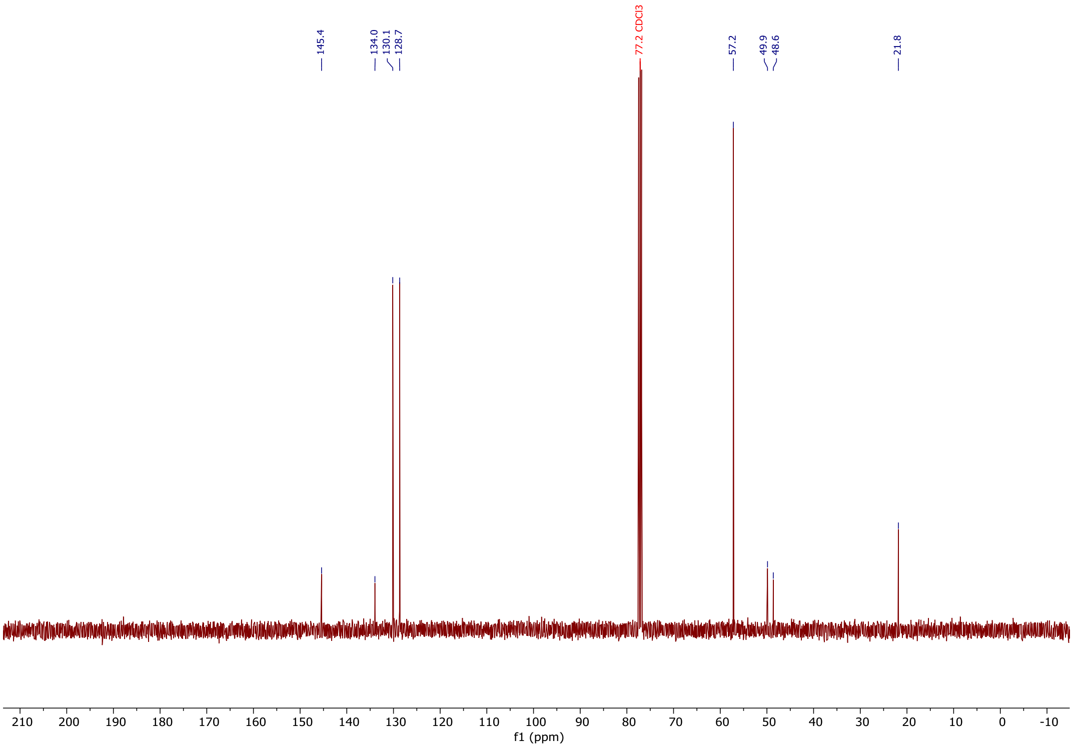

1-Iodo-3-(phenylsulfonyl)bicyclo[1.1.1]pentane, 3b-I

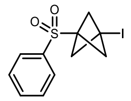

^{1}H NMR (600 MHz, CDCl$_3$)

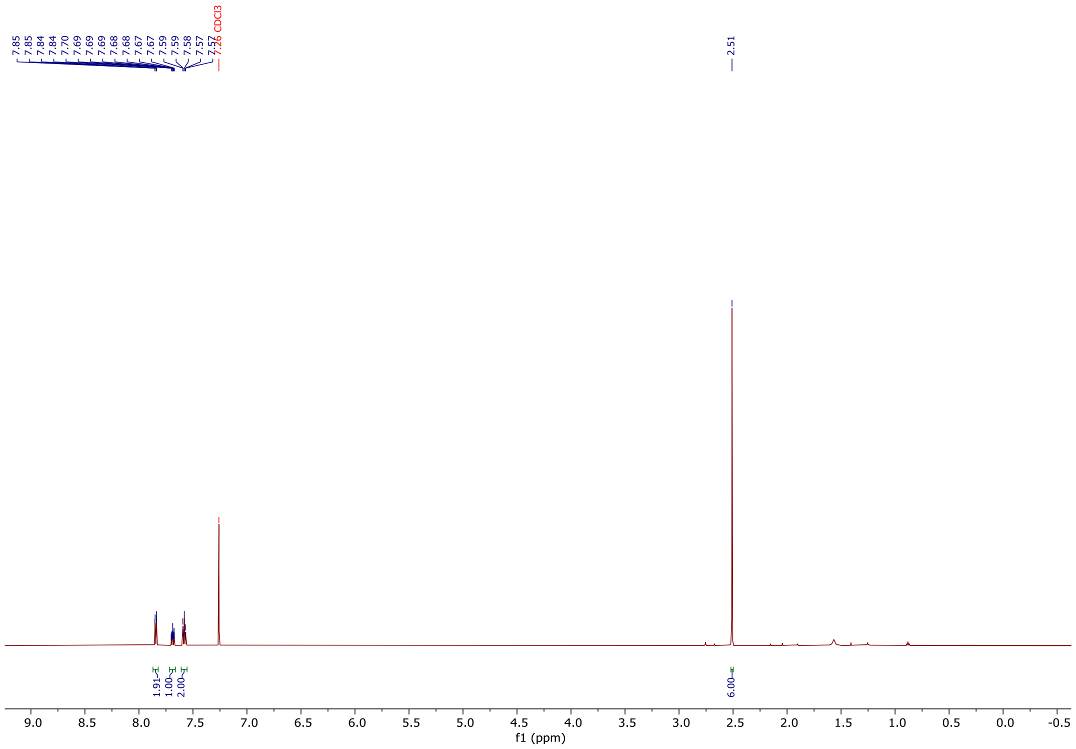

^{13}C NMR (151 MHz, CDCl$_3$)

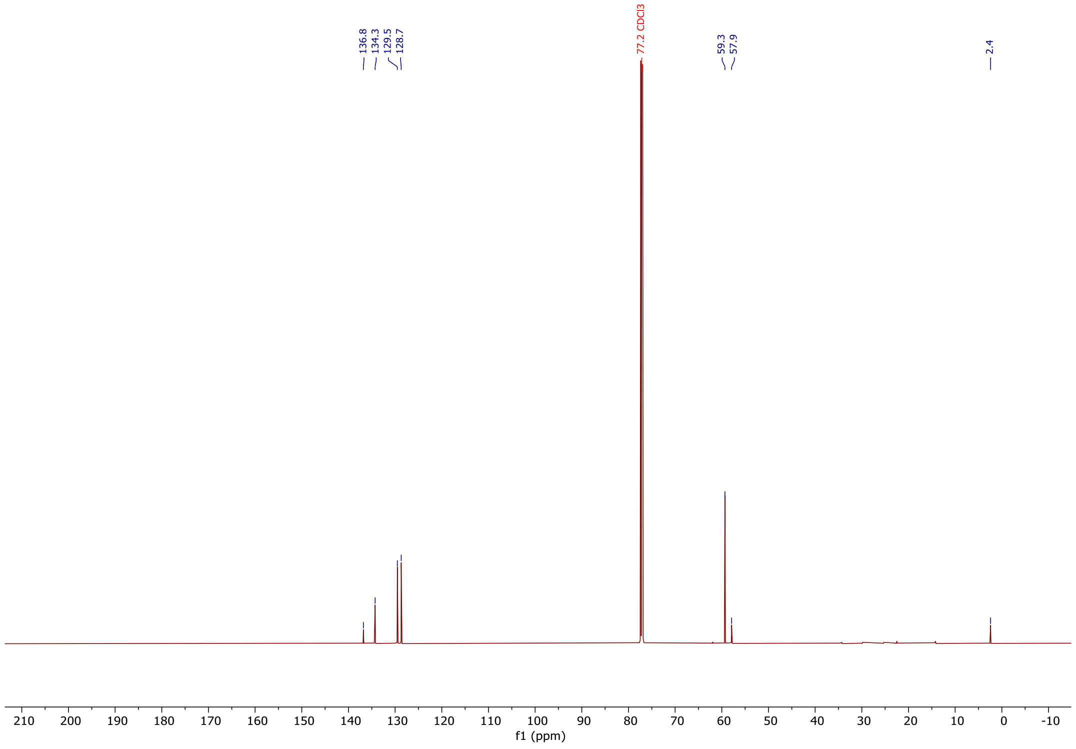

1-Bromo-3-(phenylsulfonyl)bicyclo[1.1.1]pentane, 3b-Br

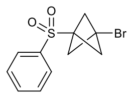

^{1}H NMR (600 MHz, $CDCl_3$)

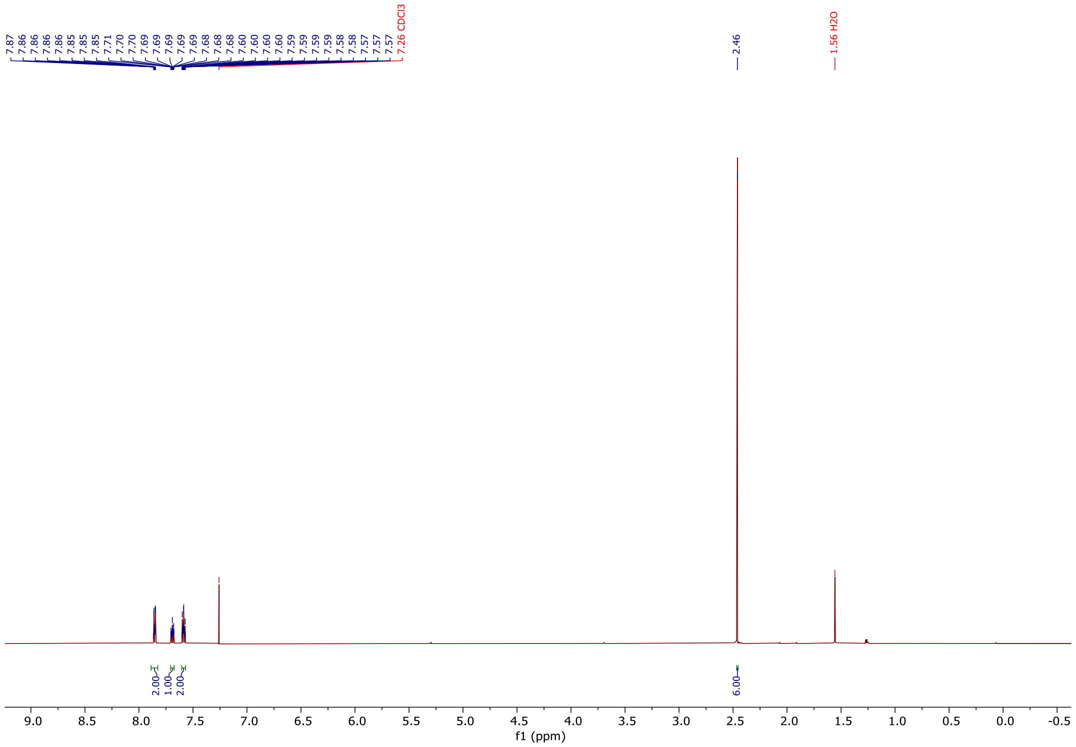

^{13}C NMR (126 MHz, $CDCl_3$)

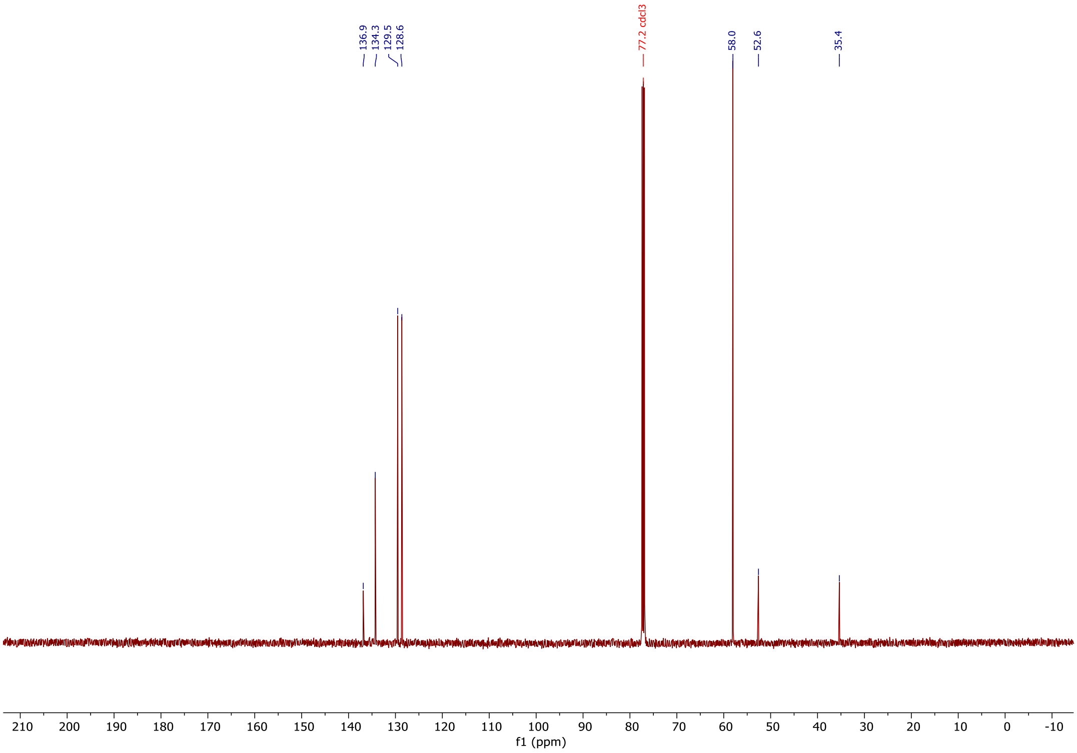

1-Iodo-3-((4-methoxyphenyl)sulfonyl)bicyclo[1.1.1]pentane, 3c-I

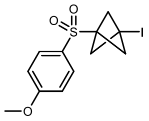

^{1}H NMR (400 MHz, $CDCl_3$)

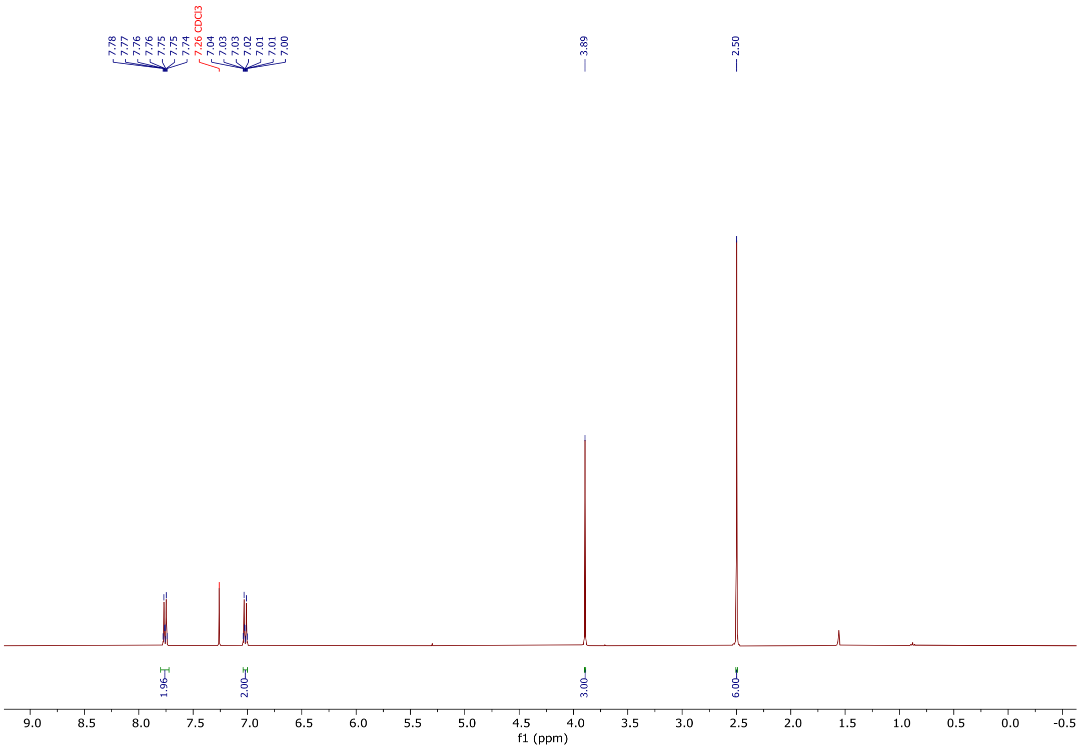

^{13}C NMR (101 MHz, $CDCl_3$)

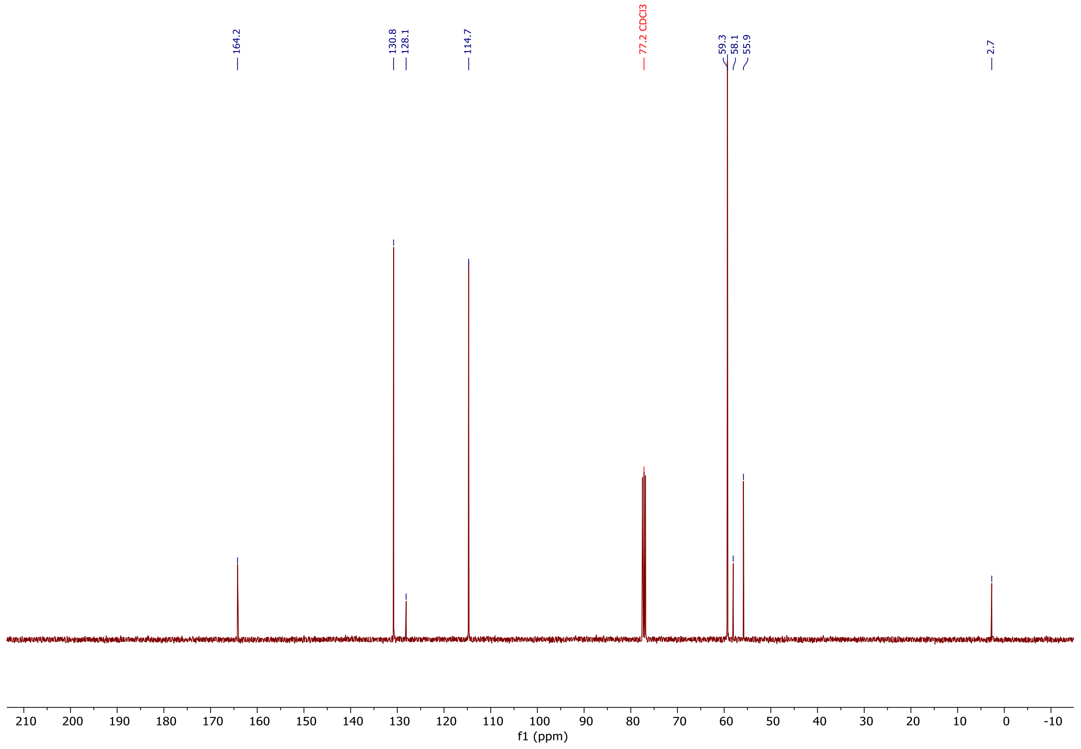

1-Bromo-3-((4-methoxyphenyl)sulfonyl)bicyclo[1.1.1]pentane, 3c-Br

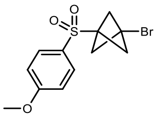

^{1}H NMR (400 MHz, $CDCl_3$)

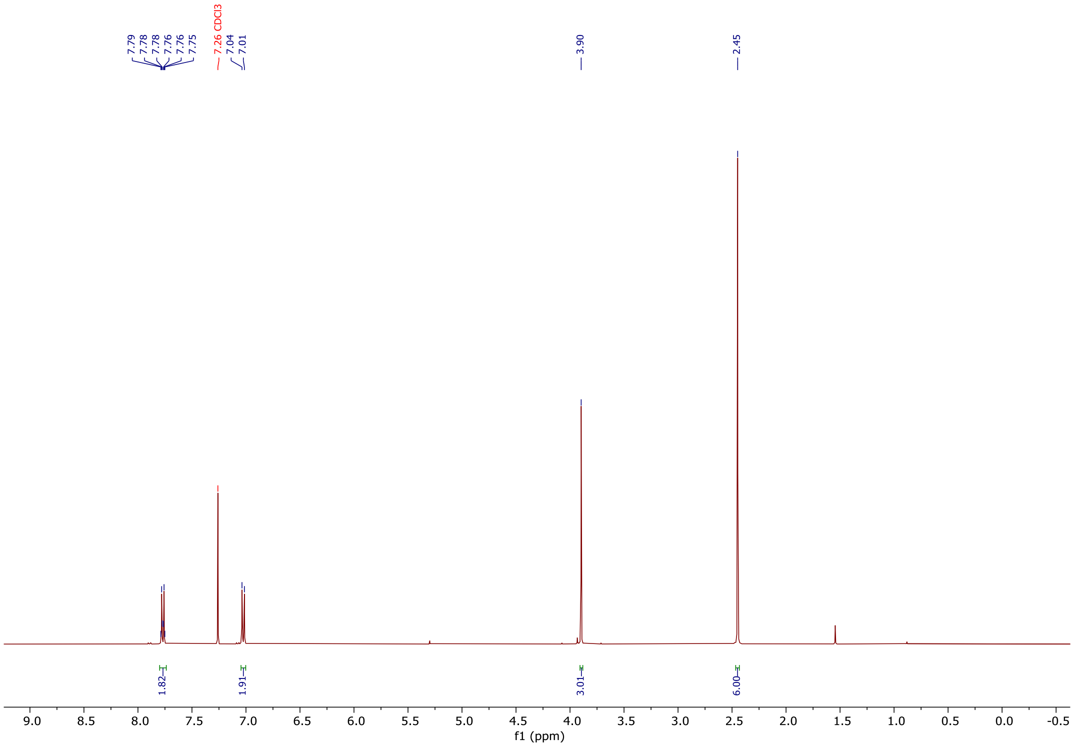

^{1}H NMR (500 MHz, DMSO-d_6)

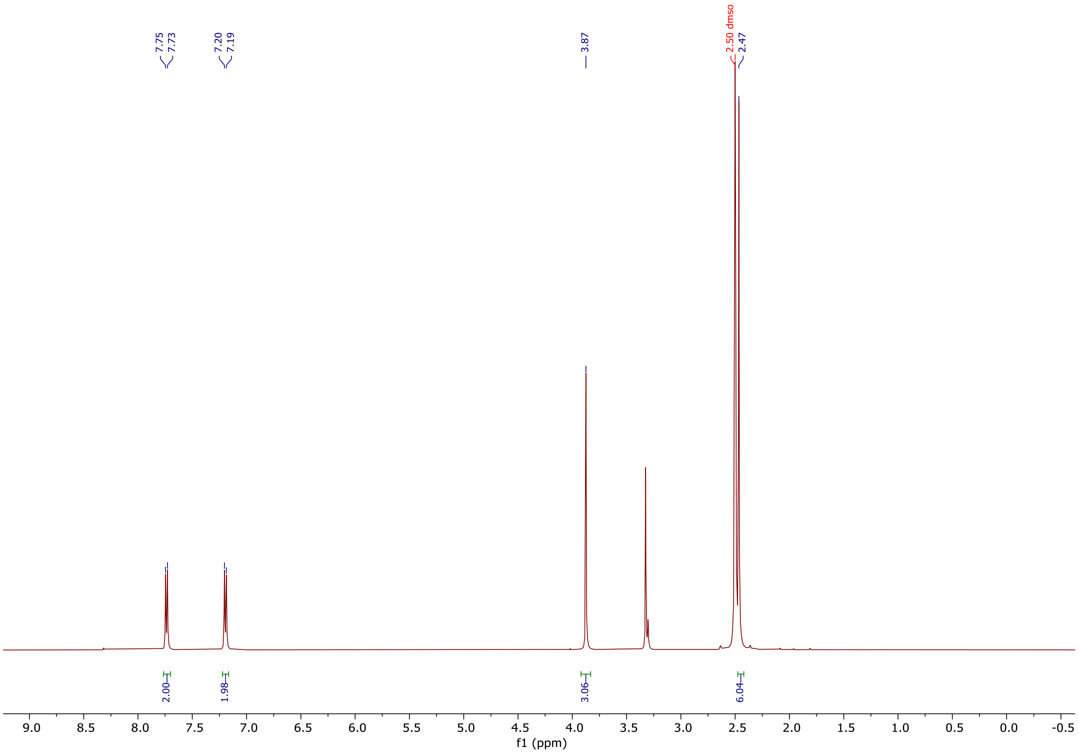

^{13}C NMR (101 MHz, $CDCl_3$)

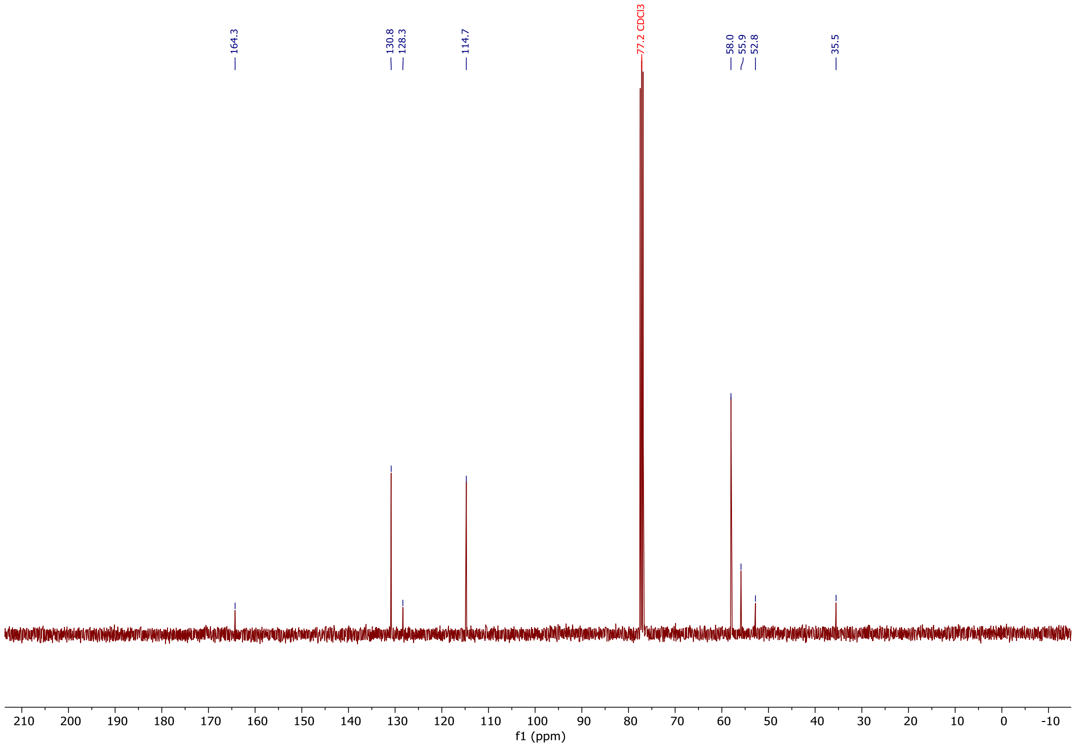

^{13}C NMR (151 MHz, DMSO-d_6)

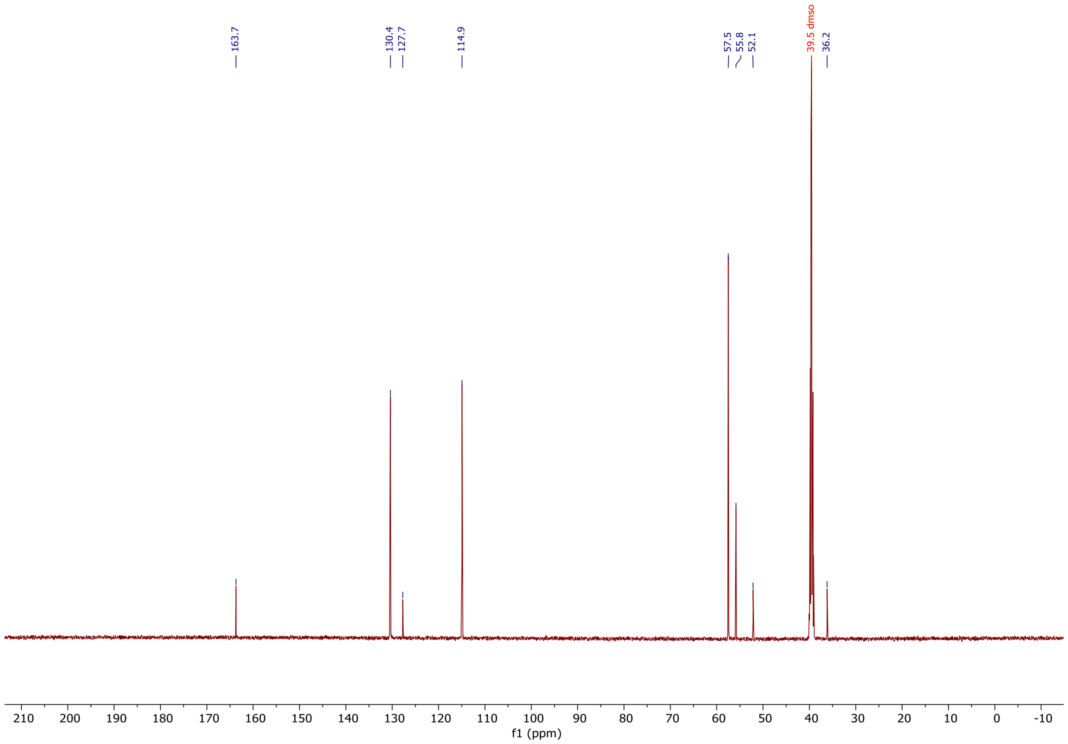

1-((4-Bromophenyl)sulfonyl)-3-iodobicyclo[1.1.1]pentane, 3d-I

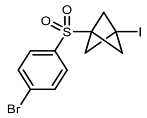

^{1}H NMR (600 MHz, $CDCl_3$)

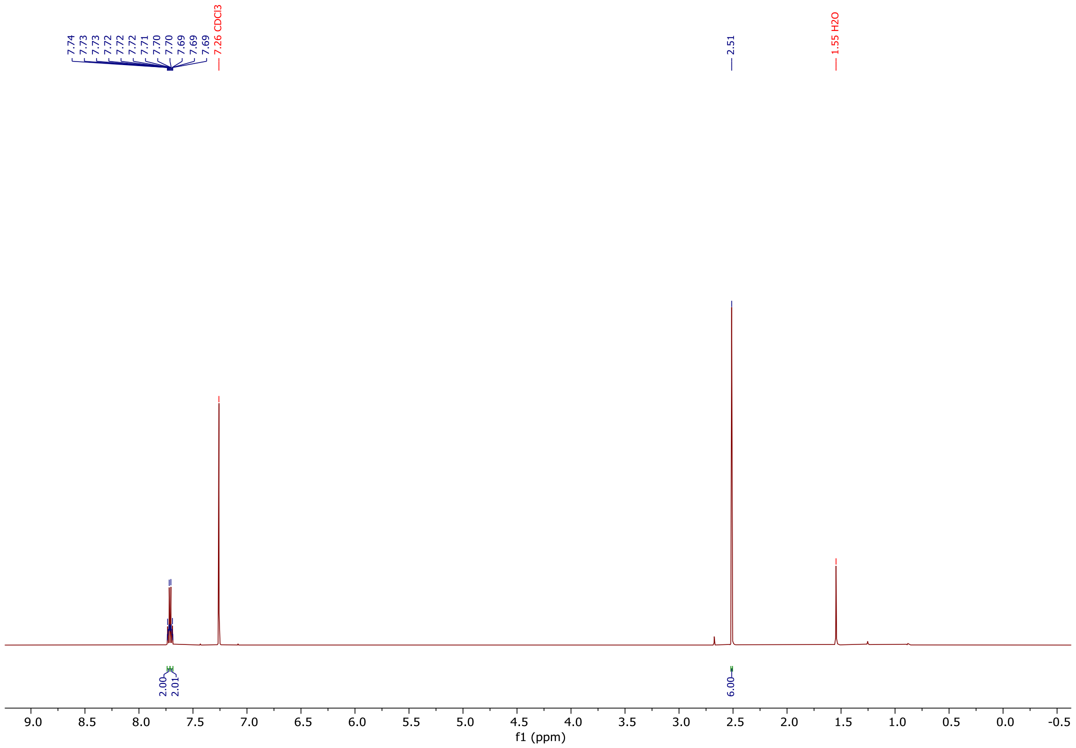

^{13}C NMR (151 MHz, $CDCl_3$)

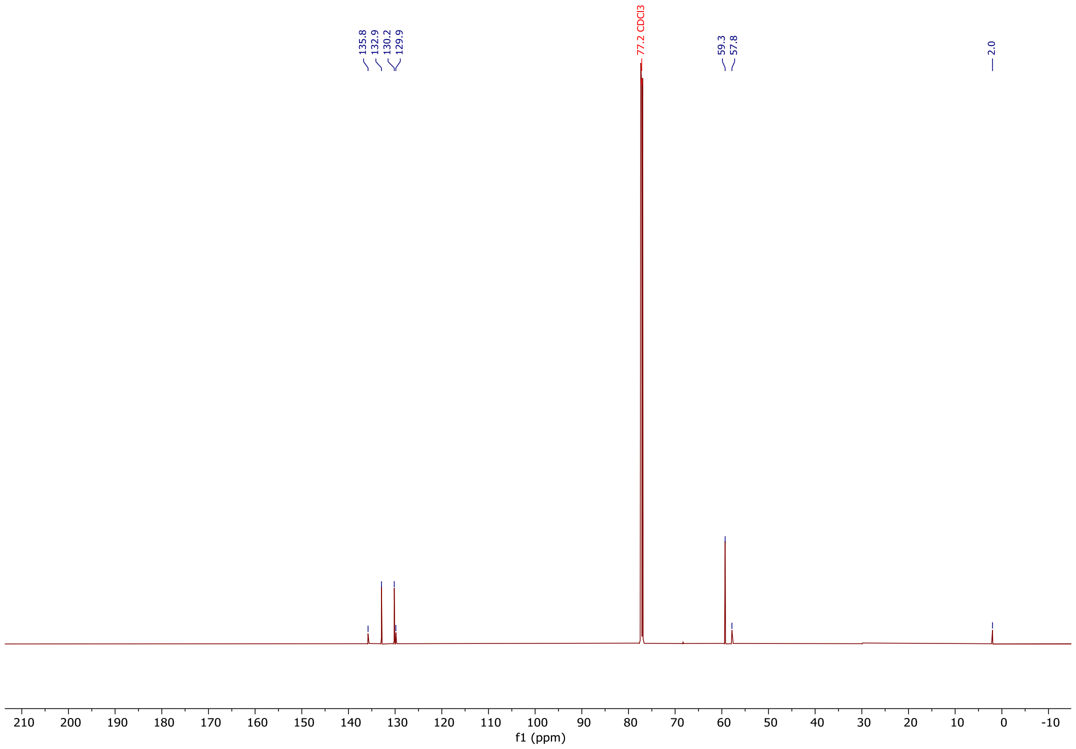

1-((4-Bromophenyl)sulfonyl)-3-bromobicyclo[1.1.1]pentane, 3d-Br

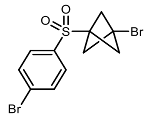

^{1}H NMR (400 MHz, $CDCl_3$)

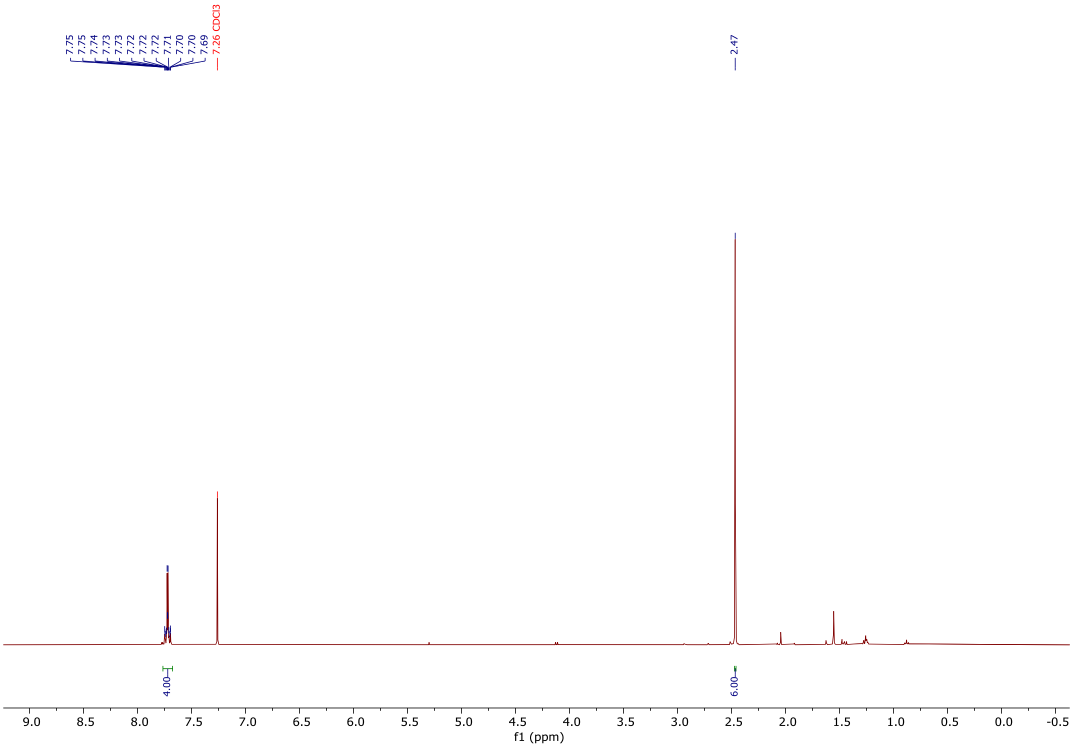

^{13}C NMR (151 MHz, $CDCl_3$)

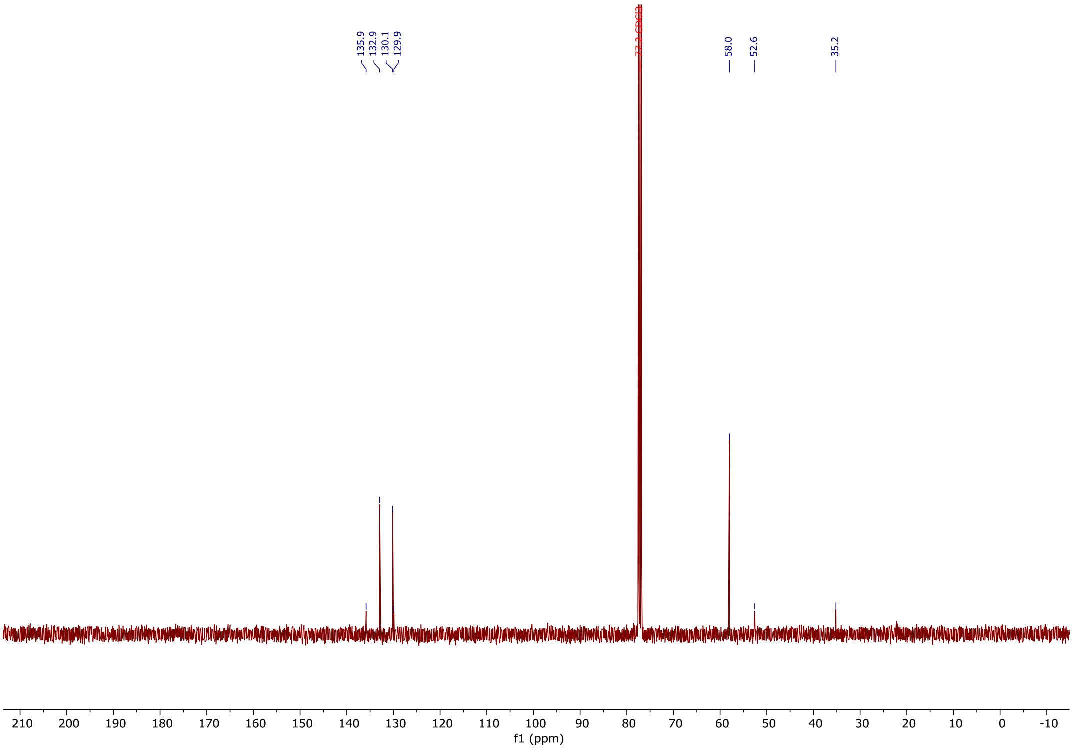

1-((4-Fluorophenyl)sulfonyl)-3-iodobicyclo[1.1.1]pentane, 3e-I

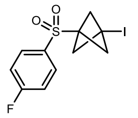

^{1}H NMR (600 MHz, $CDCl_3$)

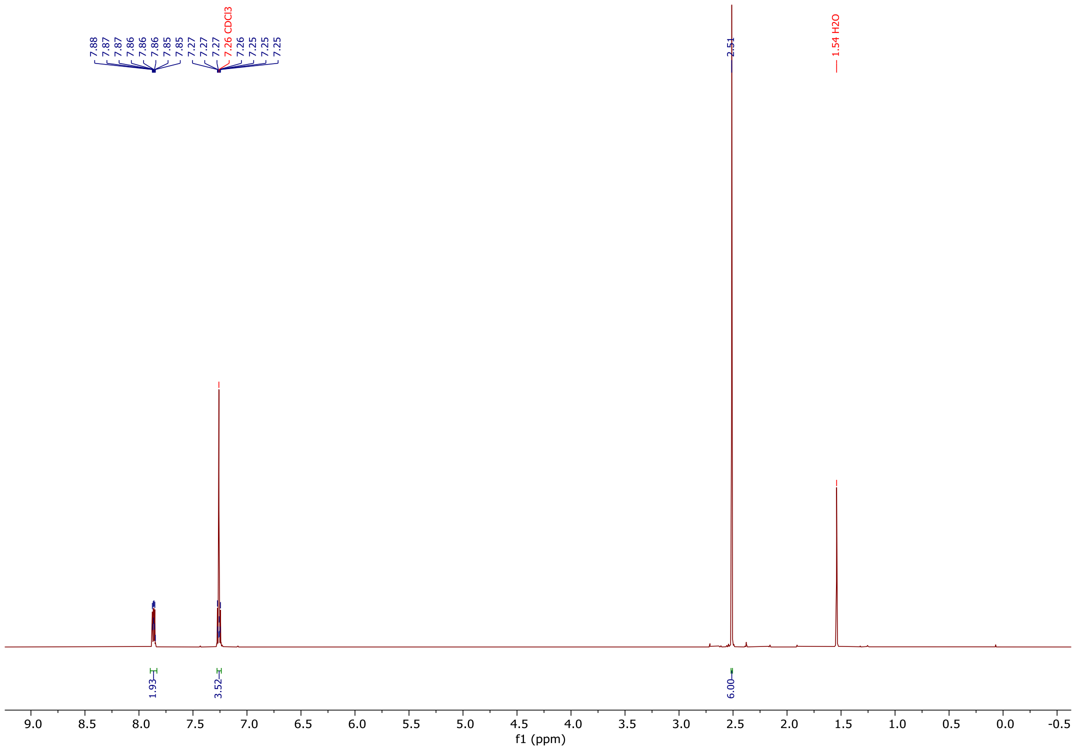

^{13}C NMR (151 MHz, $CDCl_3$)

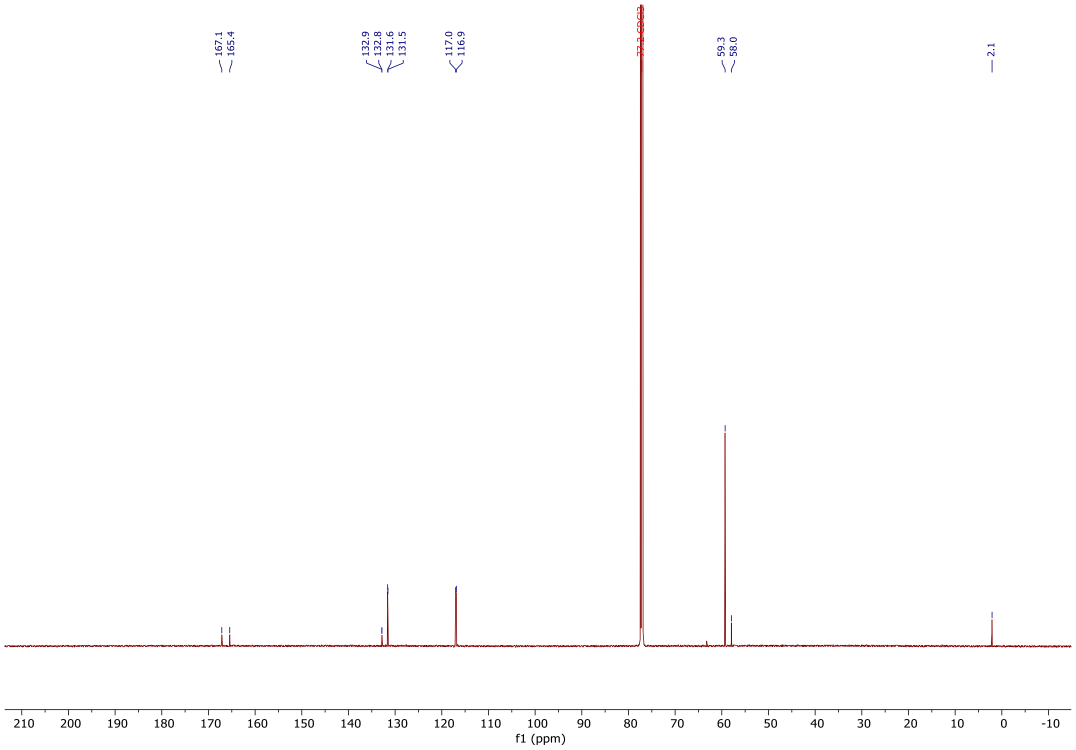

^{19}F NMR (377 MHz, $CDCl_3$)

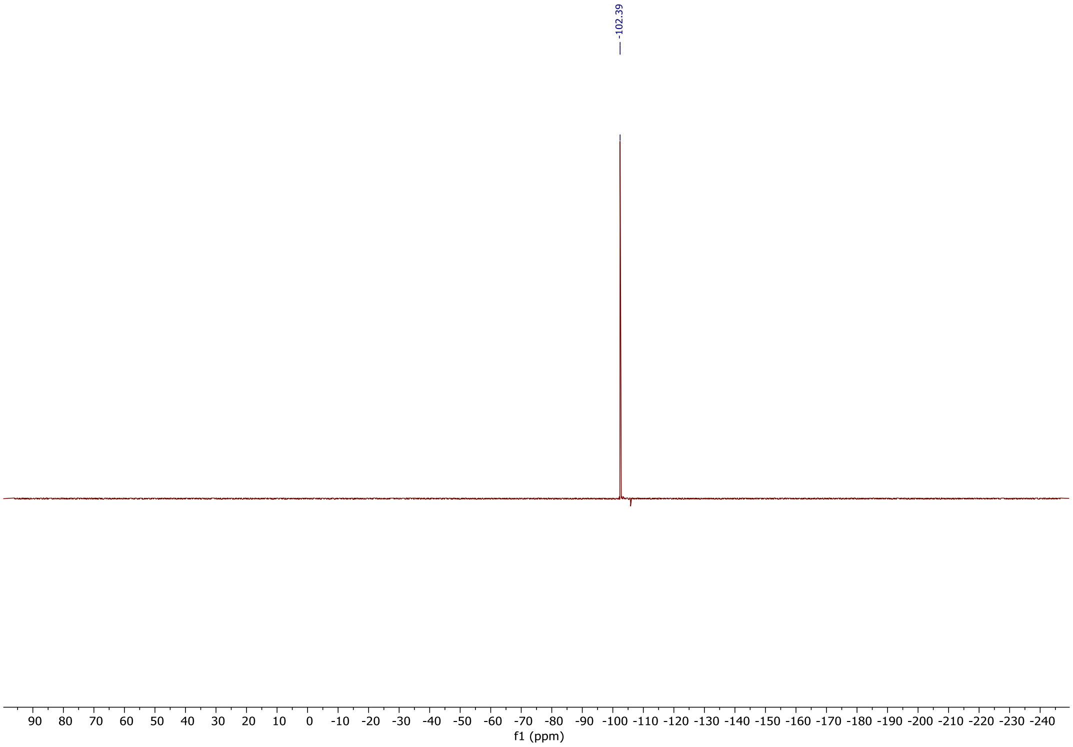

1-Bromo-3-((4-fluorophenyl)sulfonyl)bicyclo[1.1.1]pentane, 3e-Br

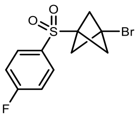

^{1}H NMR (600 MHz, $CDCl_3$)

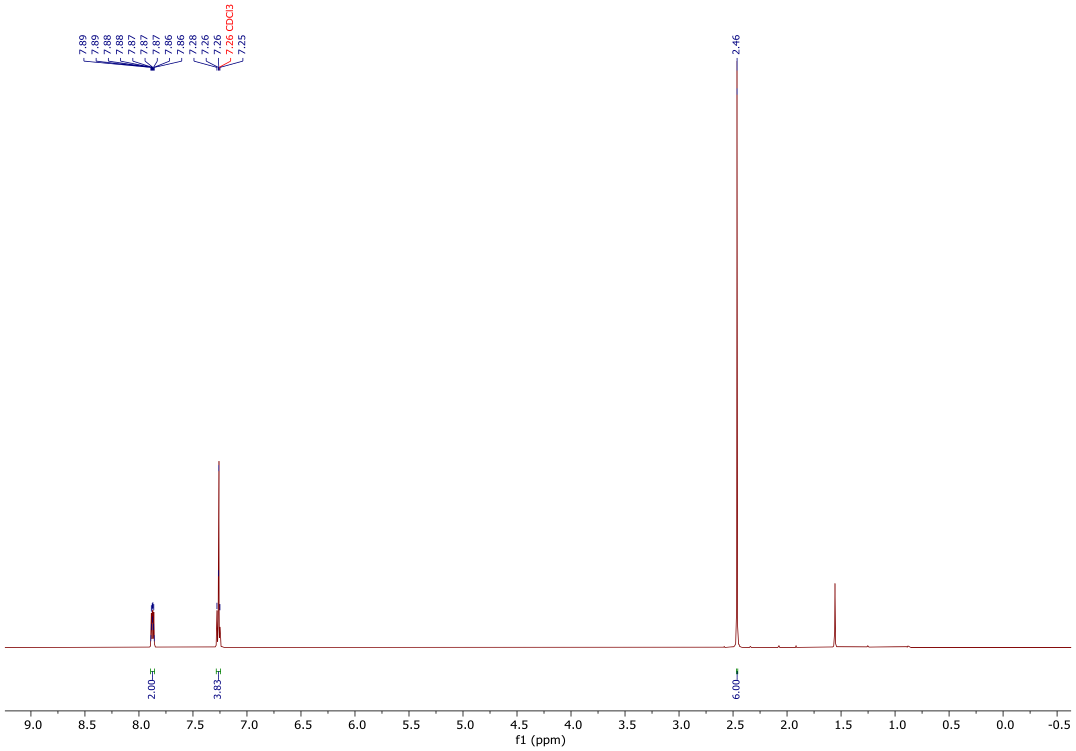

^{13}C NMR (151 MHz, $CDCl_3$)

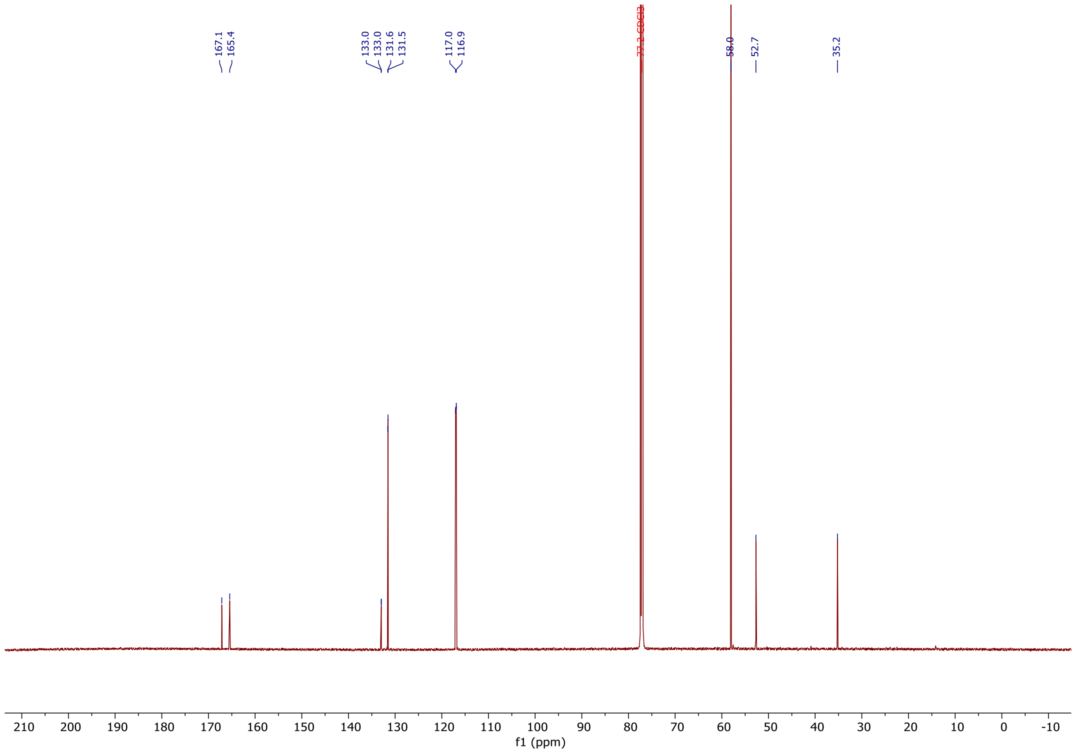

^{19}F NMR (377 MHz, $CDCl_3$)

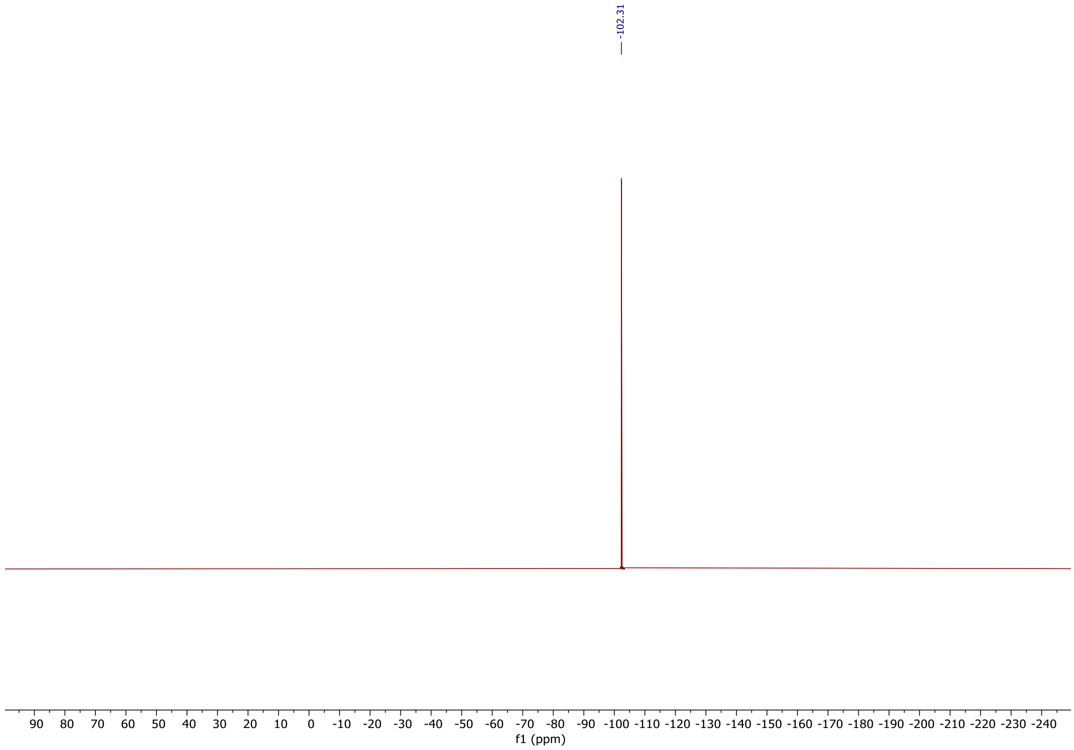

1-Bromo-3-((3-fluorophenyl)sulfonyl)bicyclo[1.1.1]pentane, 3f-Br

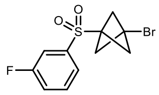

^{1}H NMR (500 MHz, $CDCl_3$)

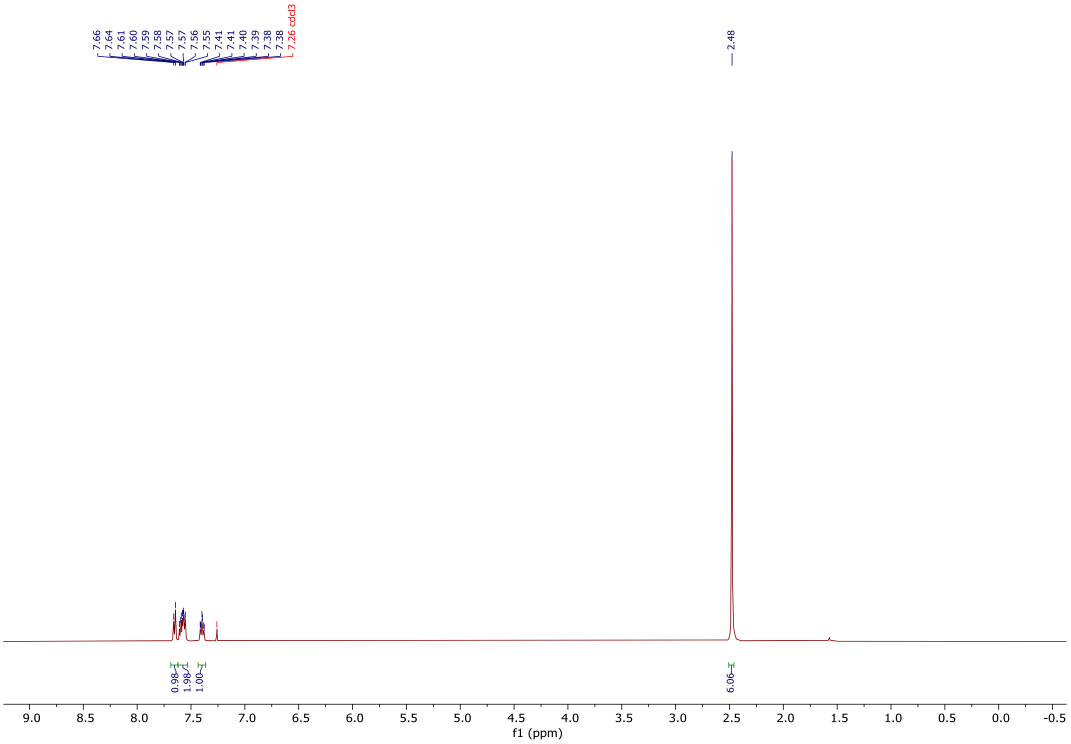

^{13}C NMR (126 MHz, $CDCl_3$)

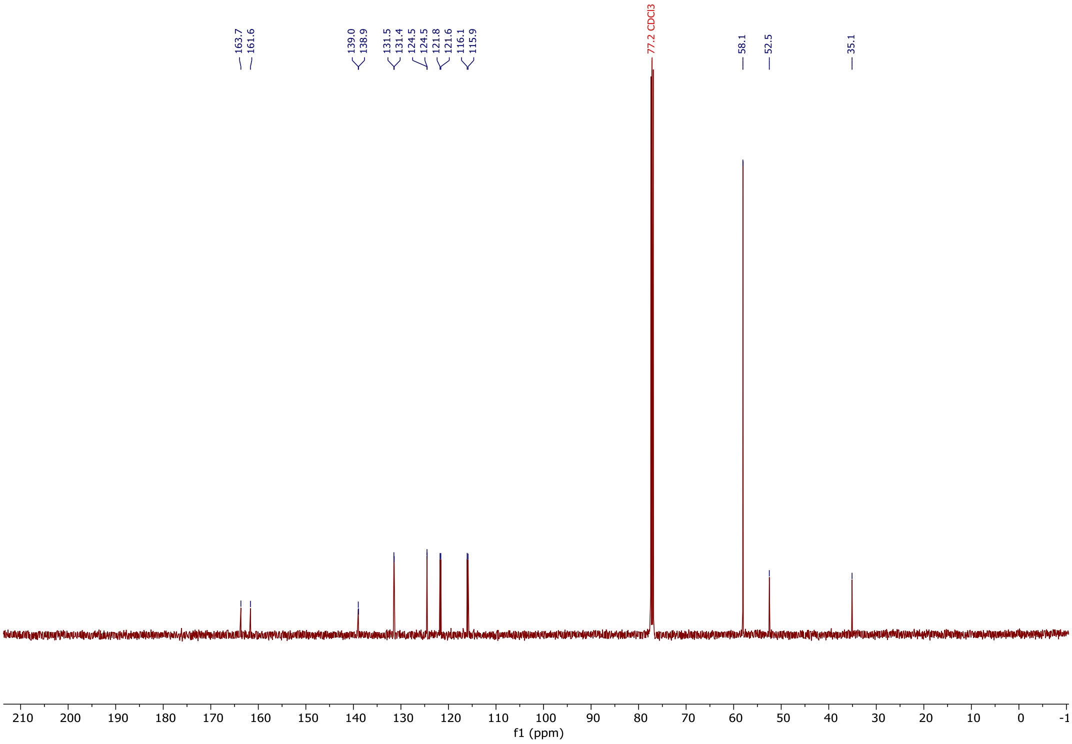

$^{19}F\ \{^{1}H\}$ **NMR** (376 MHz, $CDCl_3$)

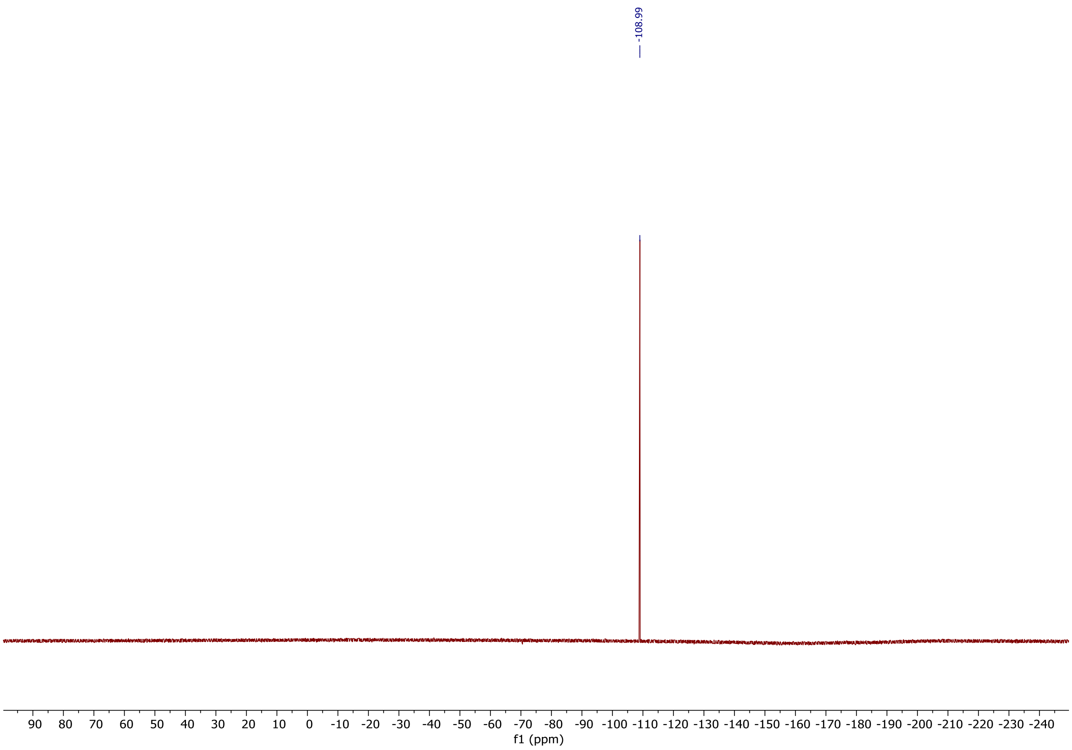

4-((3-Iodobicyclo[1.1.1]pentan-1-yl)sulfonyl)benzonitrile, 3g-I

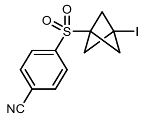

^{1}H NMR (600 MHz, $CDCl_3$)

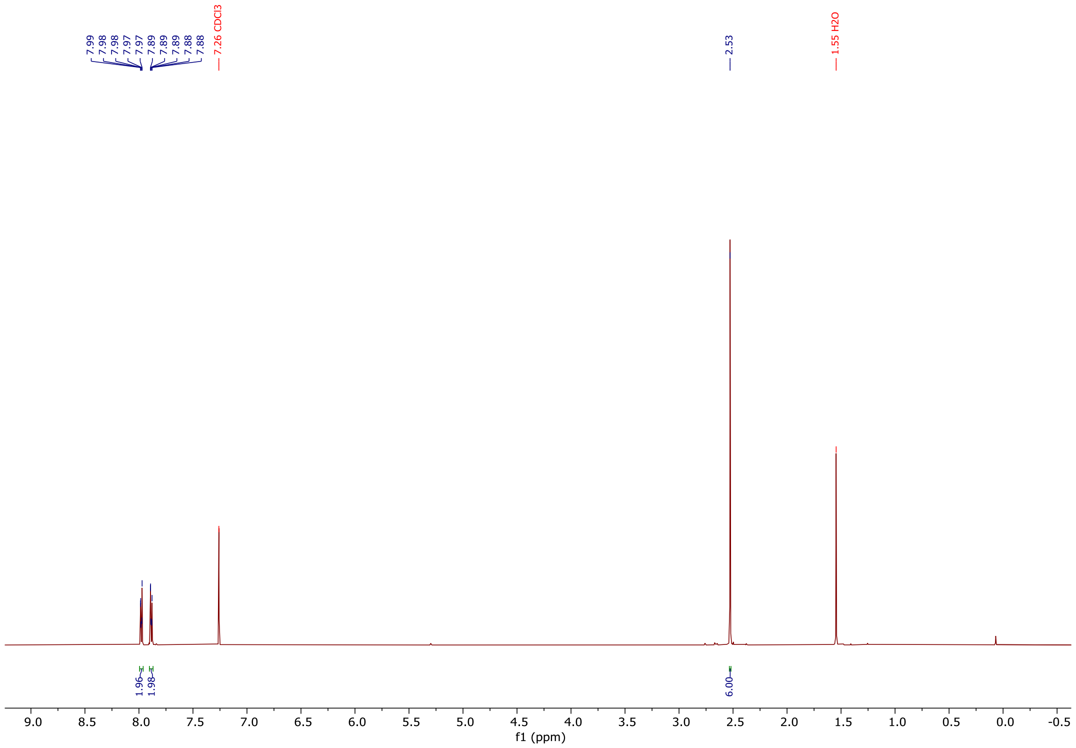

^{13}C NMR (151 MHz, $CDCl_3$)

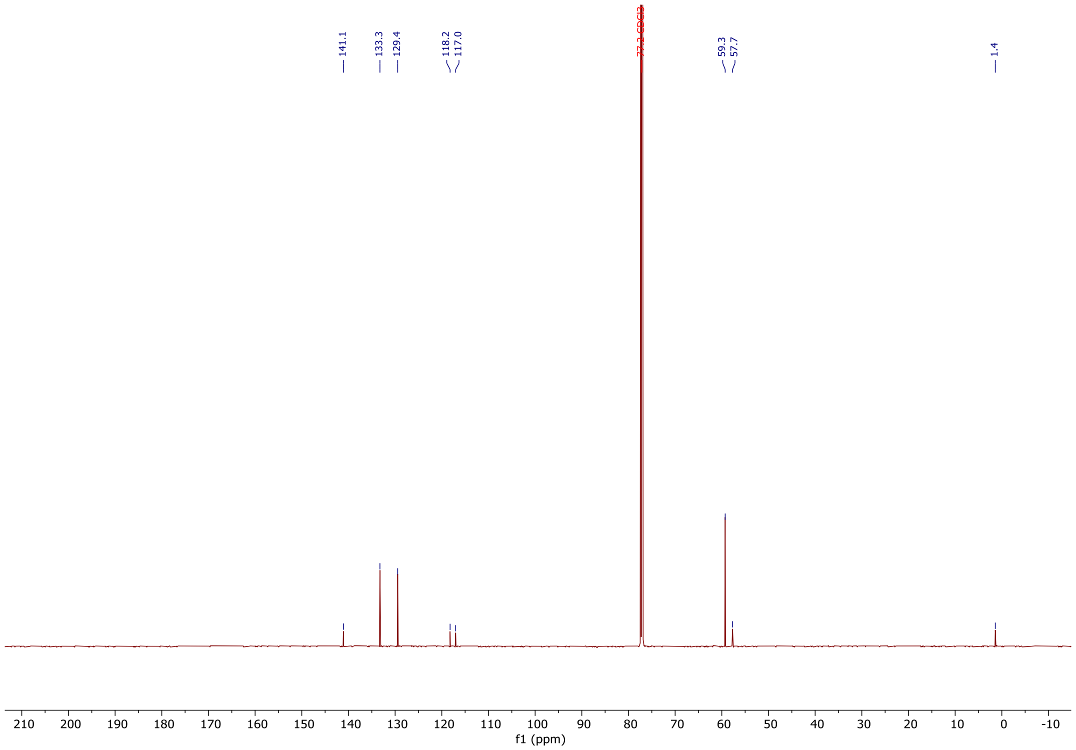

4-((3-Bromobicyclo[1.1.1]pentan-1-yl)sulfonyl)benzonitrile, 3g-Br

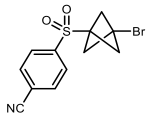

^{1}H NMR (600 MHz, $CDCl_3$)

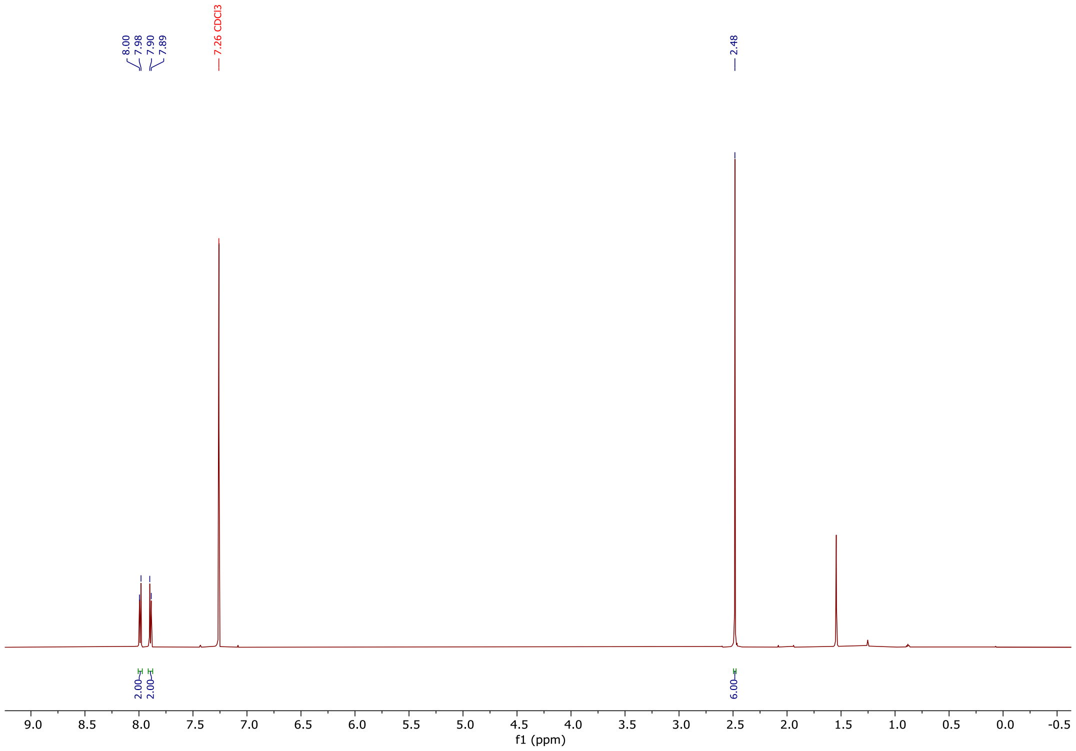

^{13}C NMR (151 MHz, $CDCl_3$)

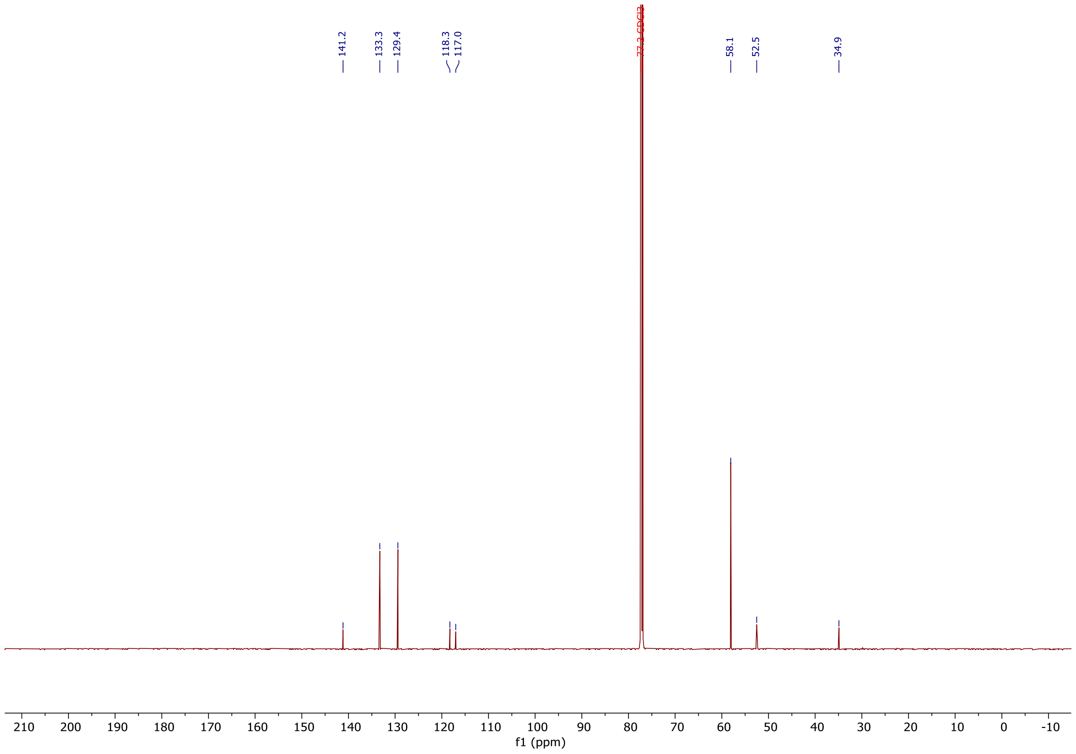

1-Iodo-3-((4-nitrophenyl)sulfonyl)bicyclo[1.1.1]pentane, 3h-I

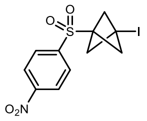

^{1}H NMR (600 MHz, $CDCl_3$)

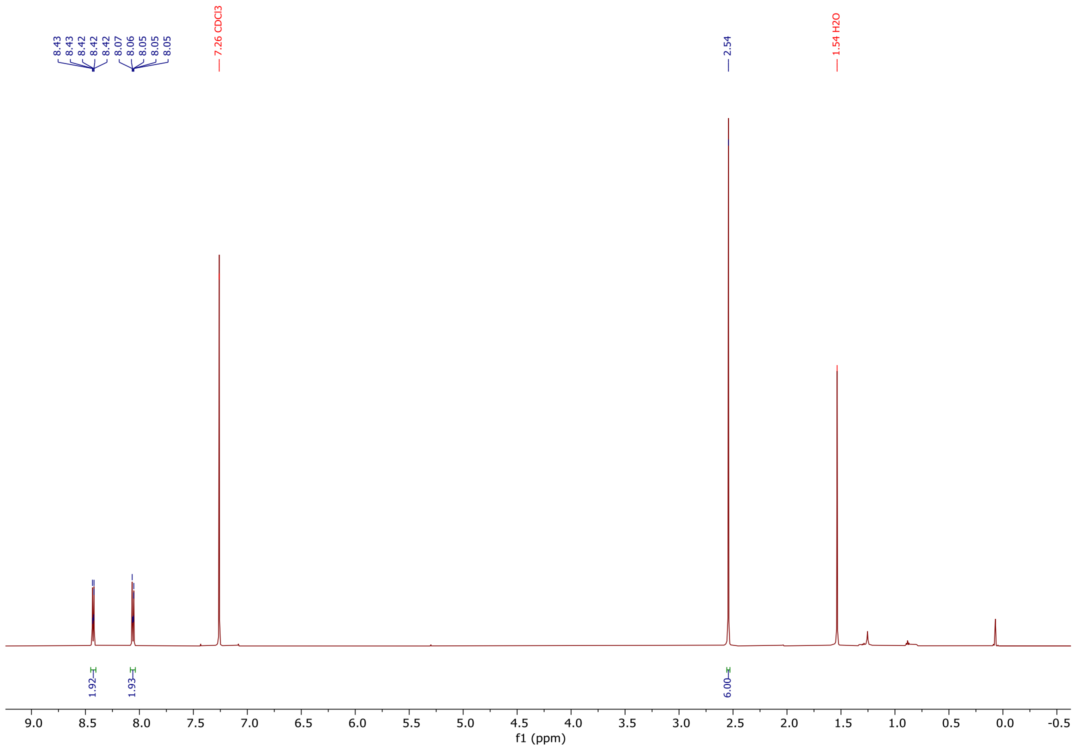

^{13}C NMR (151 MHz, $CDCl_3$)

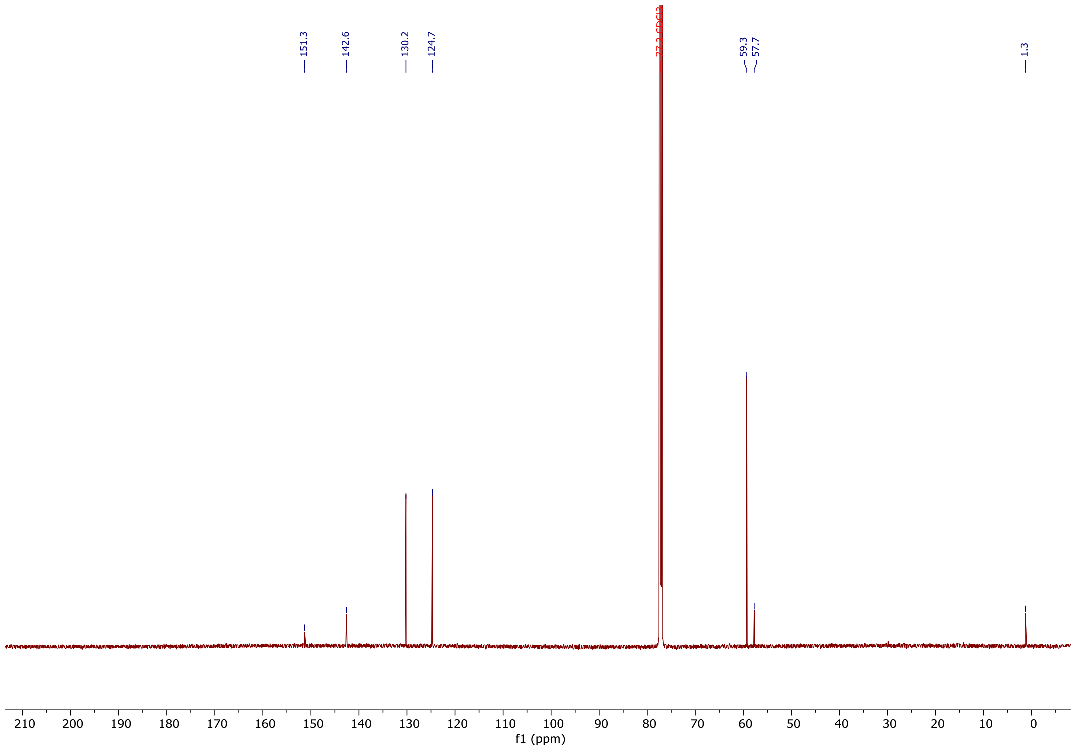

1-Bromo-3-((4-nitrophenyl)sulfonyl)bicyclo[1.1.1]pentane, 3h-Br

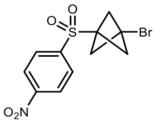

^{1}H NMR (600 MHz, $CDCl_3$)

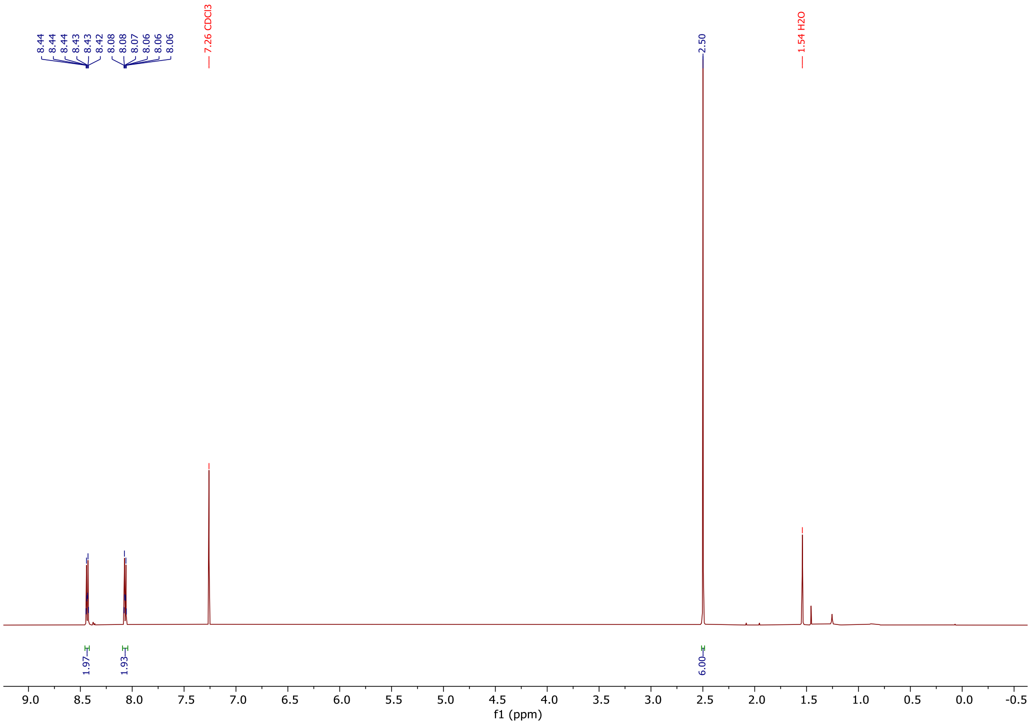

^{13}C NMR (151 MHz, $CDCl_3$)

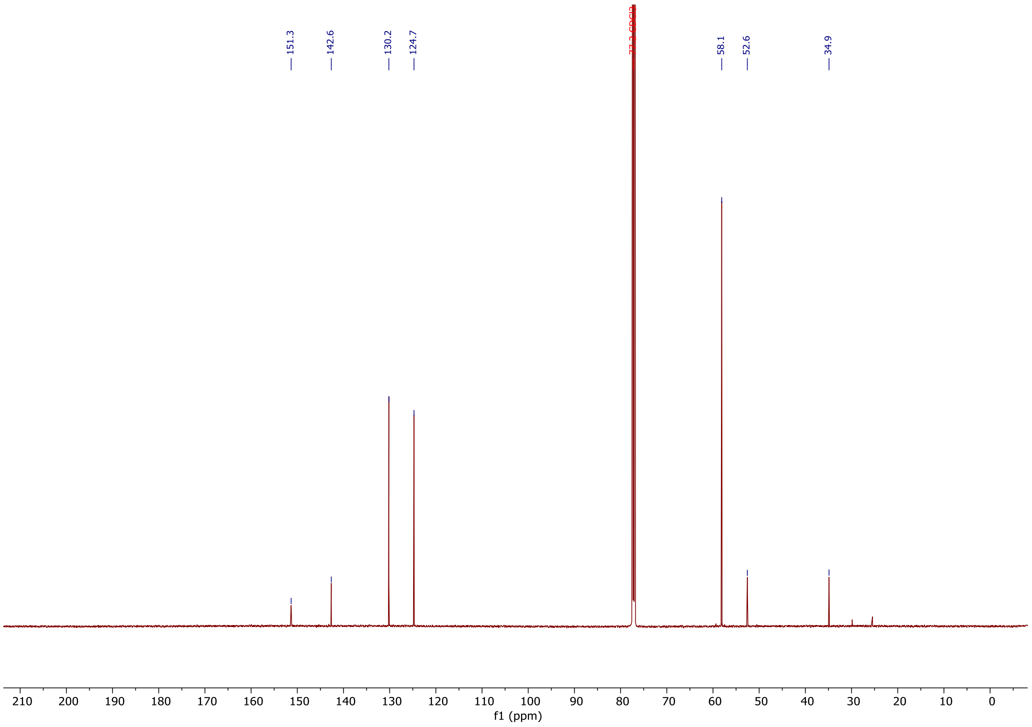

1-Bromo-3-((4-(trifluoromethyl)phenyl)sulfonyl)bicyclo[1.1.1]pentane, 3i-I

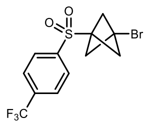

^{1}H NMR (500 MHz, $CDCl_3$)

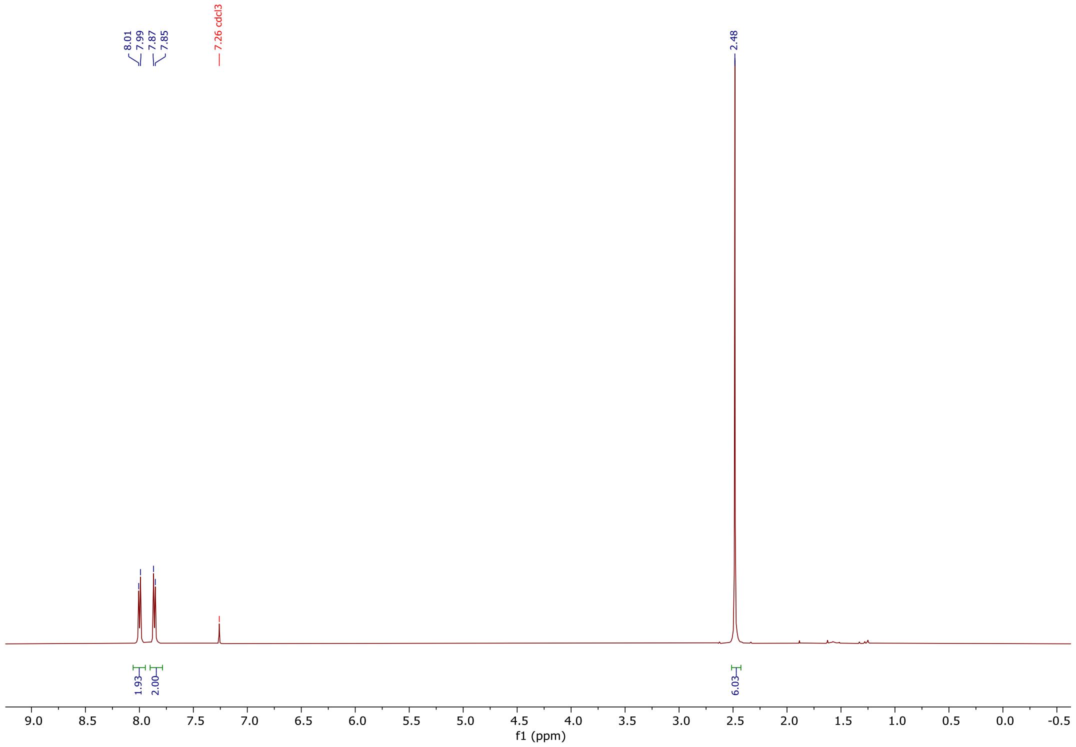

^{13}C NMR (151 MHz, $CDCl_3$)

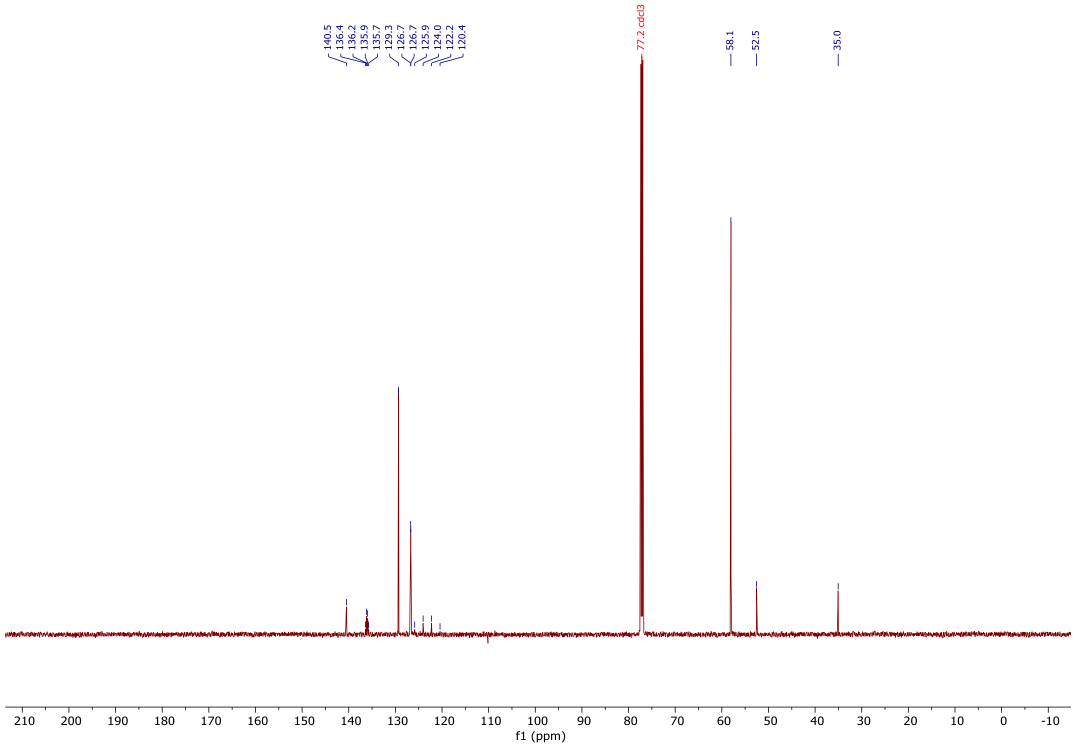

$^{19}F\ \{^{1}H\}$ **NMR** (376 MHz, $CDCl_3$)

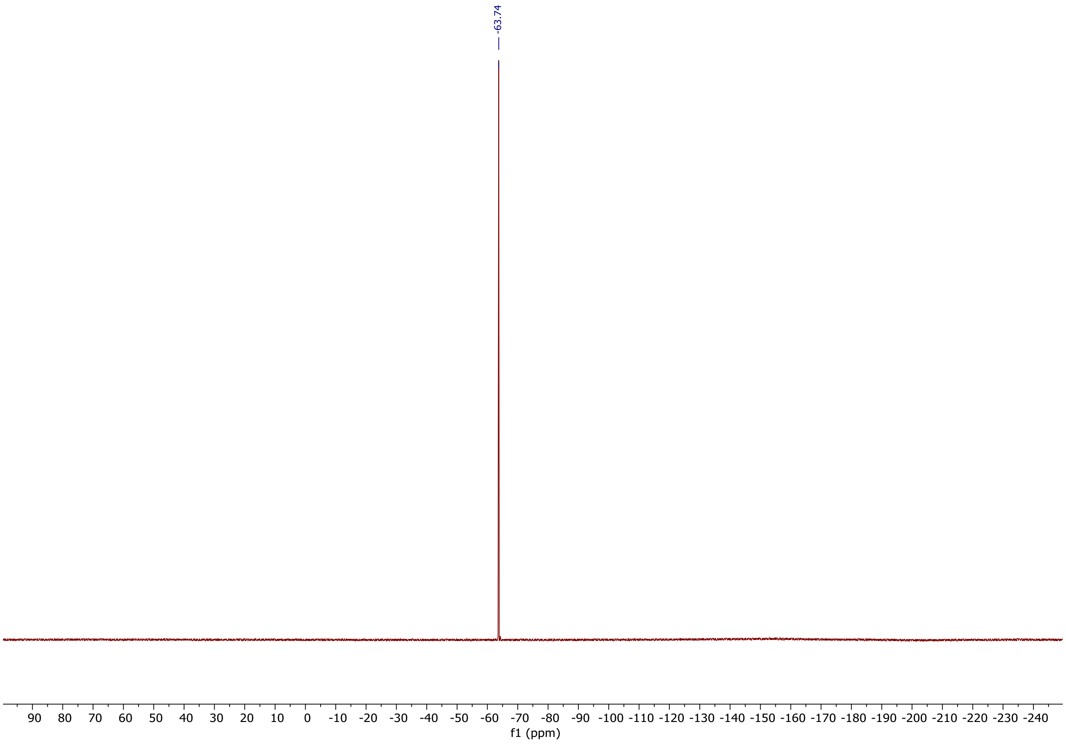

Methyl 4-((3-iodobicyclo[1.1.1]pentan-1-yl)sulfonyl)benzoate, 3j-I

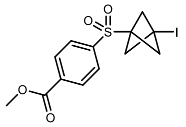

^{1}H NMR (600 MHz, $CDCl_3$)

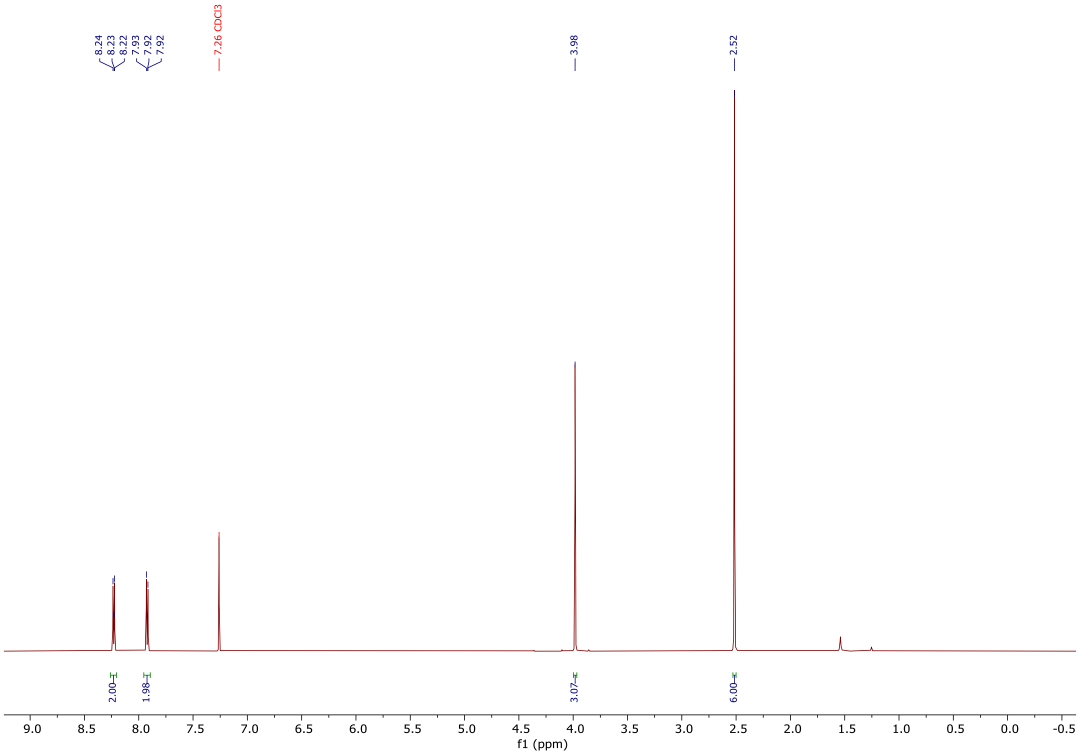

^{13}C NMR (151 MHz, $CDCl_3$)

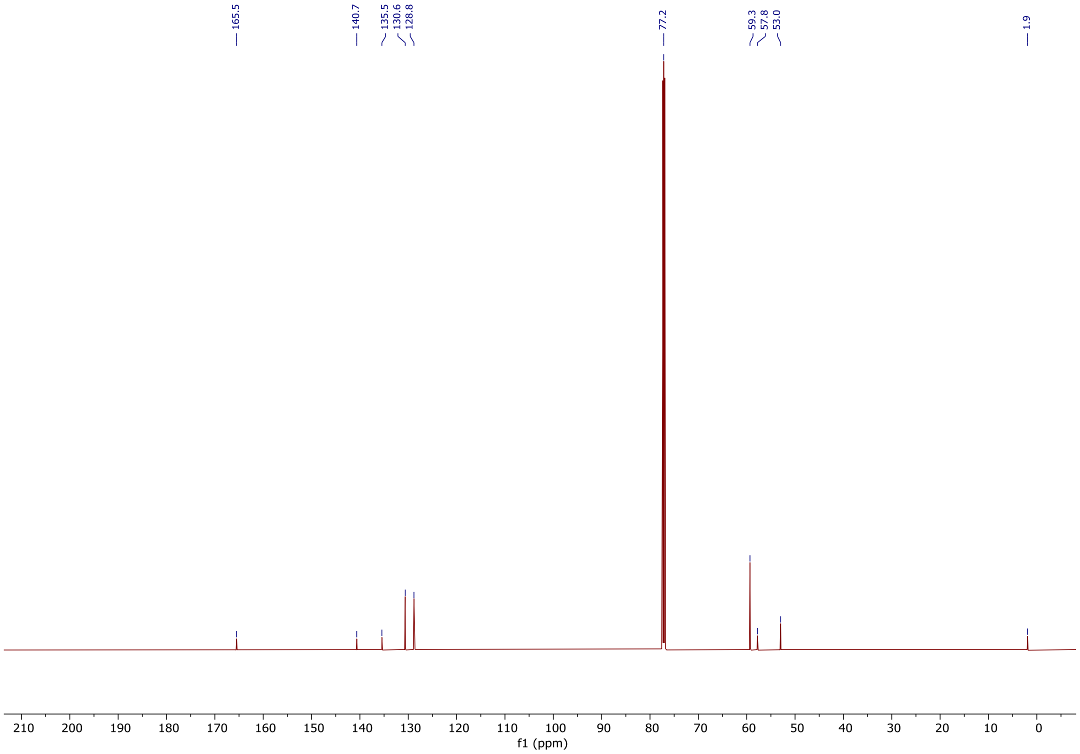

Methyl 4-((3-bromobicyclo[1.1.1]pentan-1-yl)sulfonyl)benzoate, 3j-Br

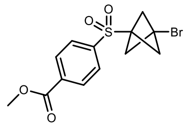

^{1}H NMR (600 MHz, $CDCl_3$)

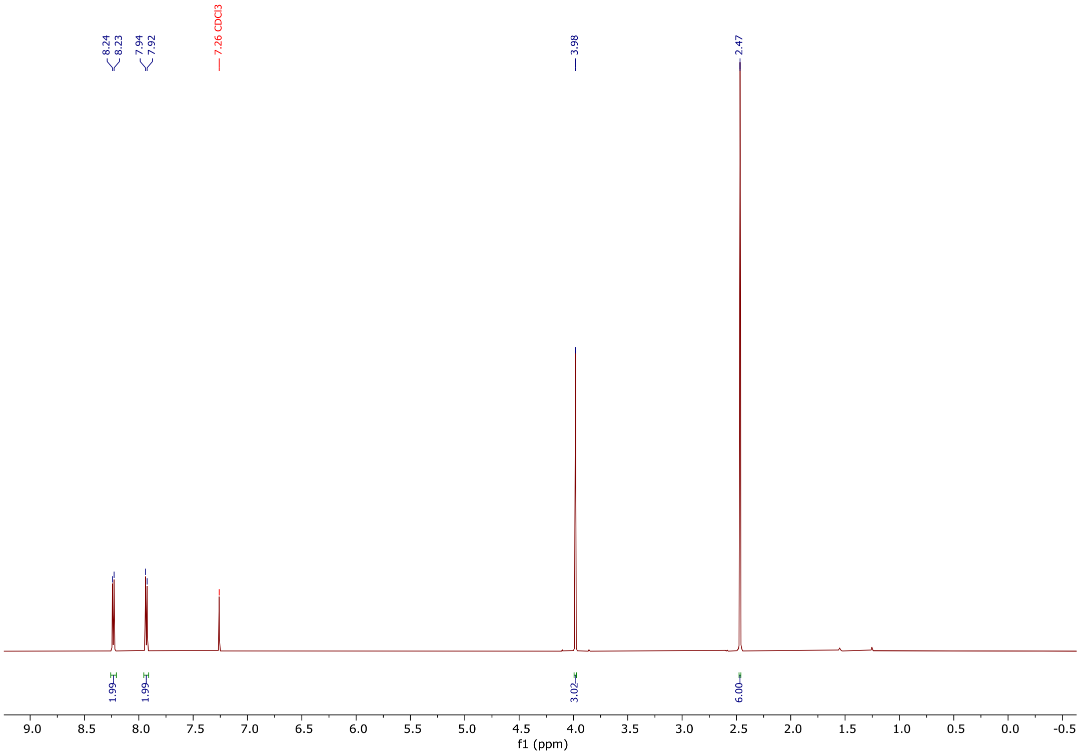

^{13}C NMR (151 MHz, $CDCl_3$)

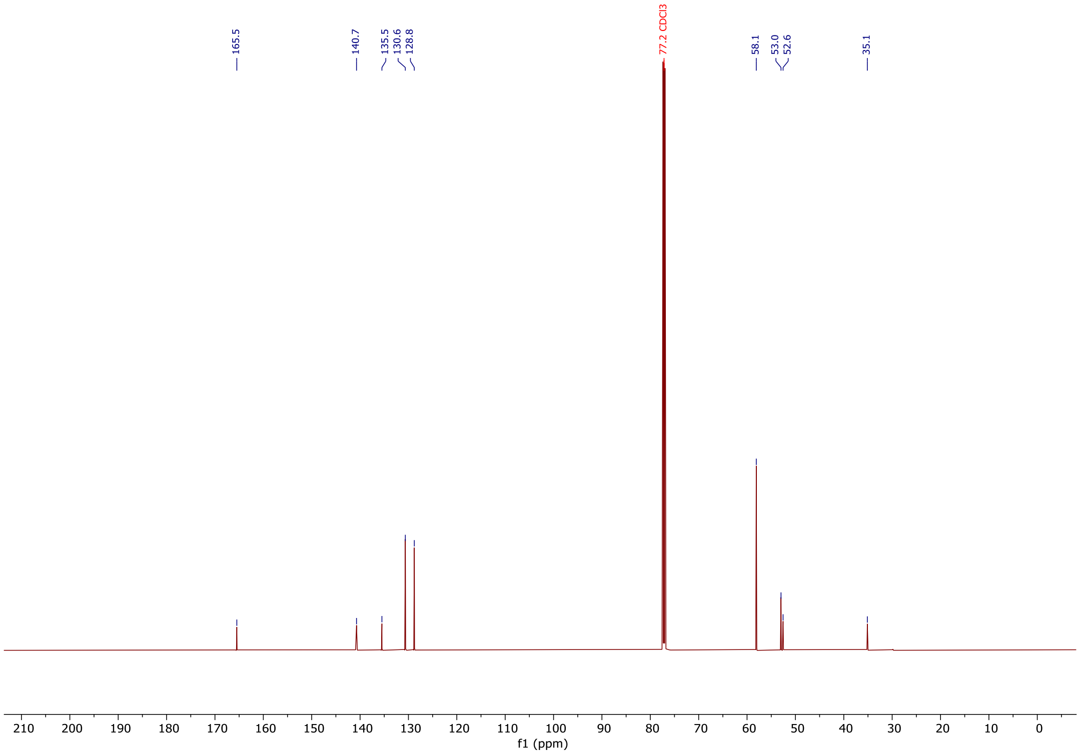

1-Iodo-3-(o-tolylsulfonyl)bicyclo[1.1.1]pentane, 3k-I

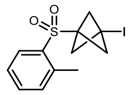

^{1}H NMR (400 MHz, $CDCl_3$)

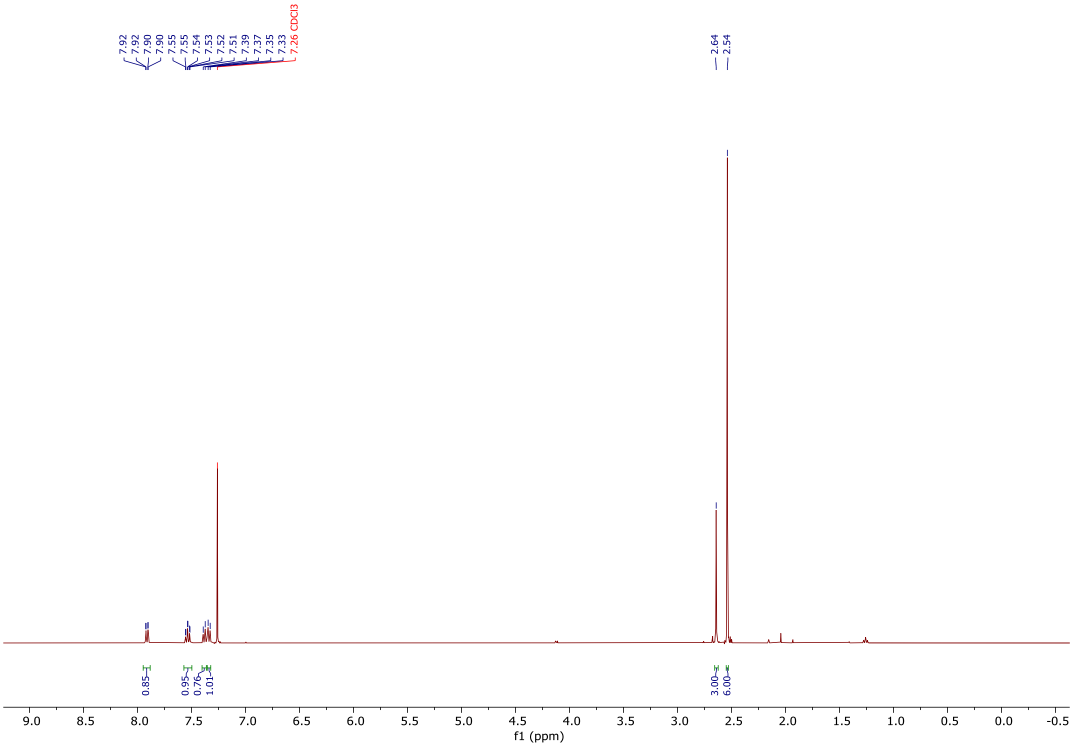

^{13}C NMR (101 MHz, $CDCl_3$)

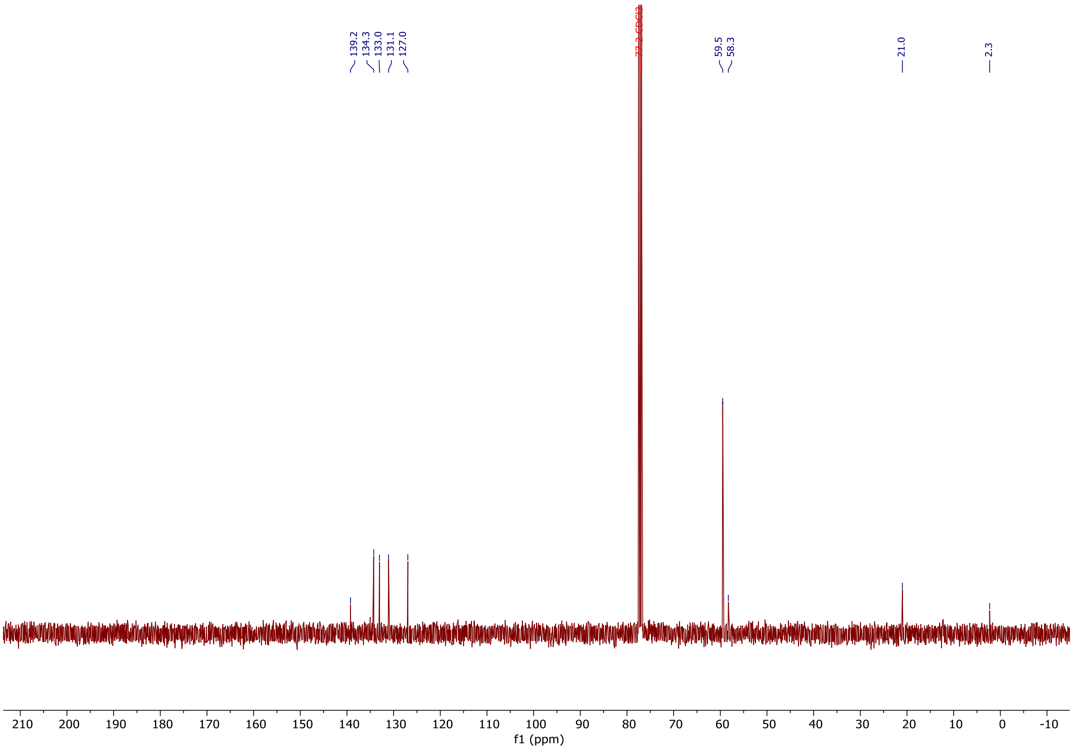

1-Bromo-3-(o-tolylsulfonyl)bicyclo[1.1.1]pentane, 3k-Br

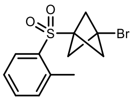

^{1}H NMR (400 MHz, $CDCl_3$)

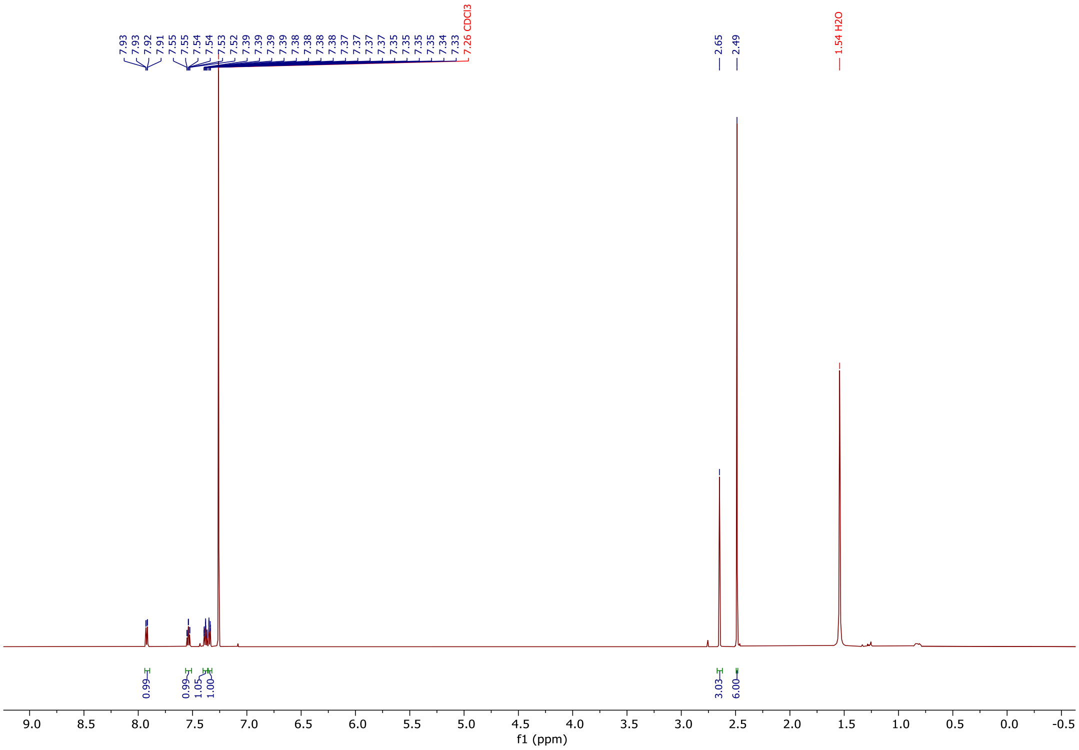

^{13}C NMR (151 MHz, $CDCl_3$)

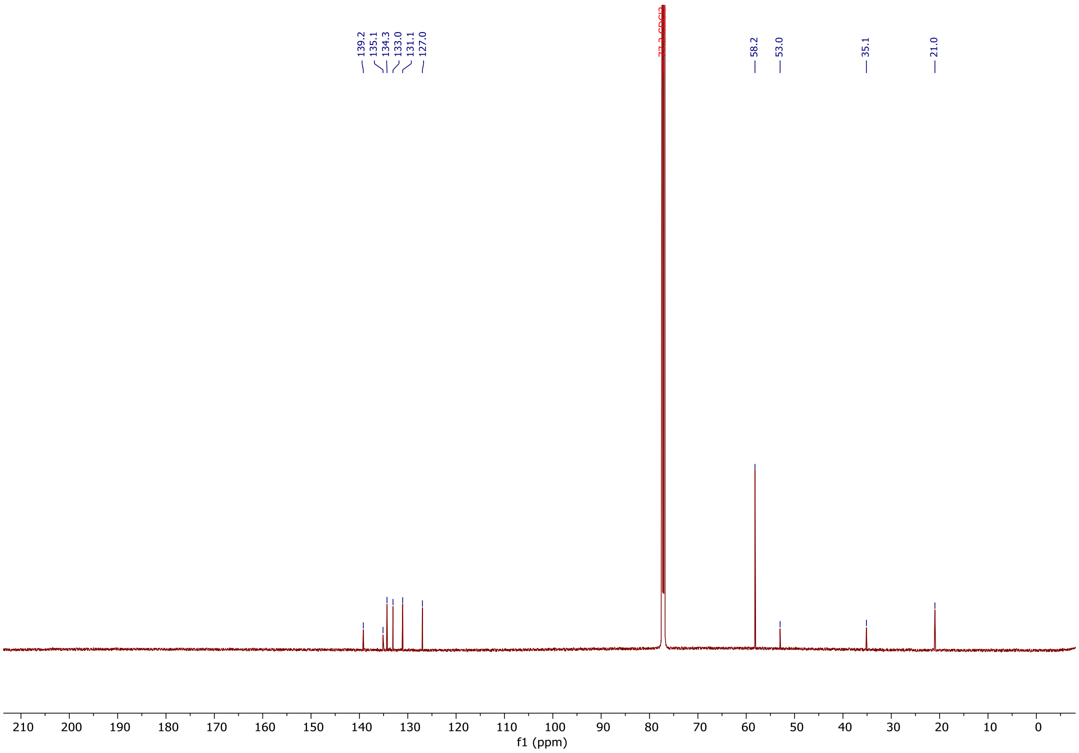

1-Iodo-3-(mesitylsulfonyl)bicyclo[1.1.1]pentane, 3l-I

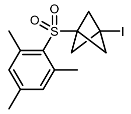

^{1}H NMR (600 MHz, CDCl$_3$)

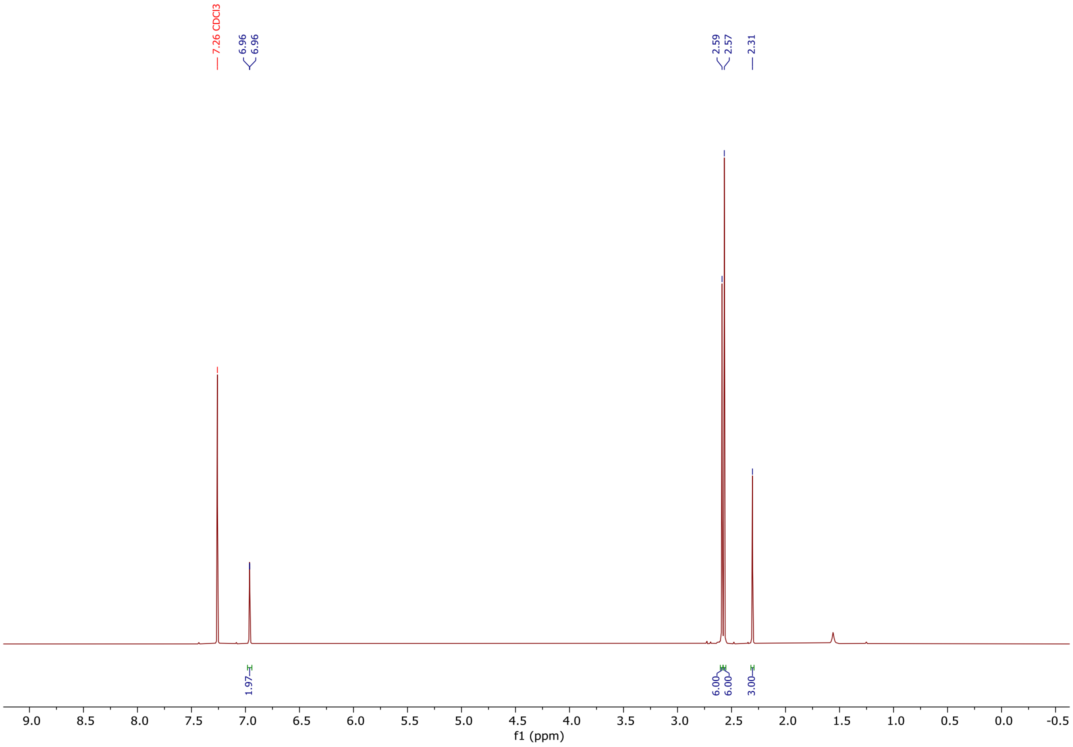

^{13}C NMR (151 MHz, CDCl$_3$)

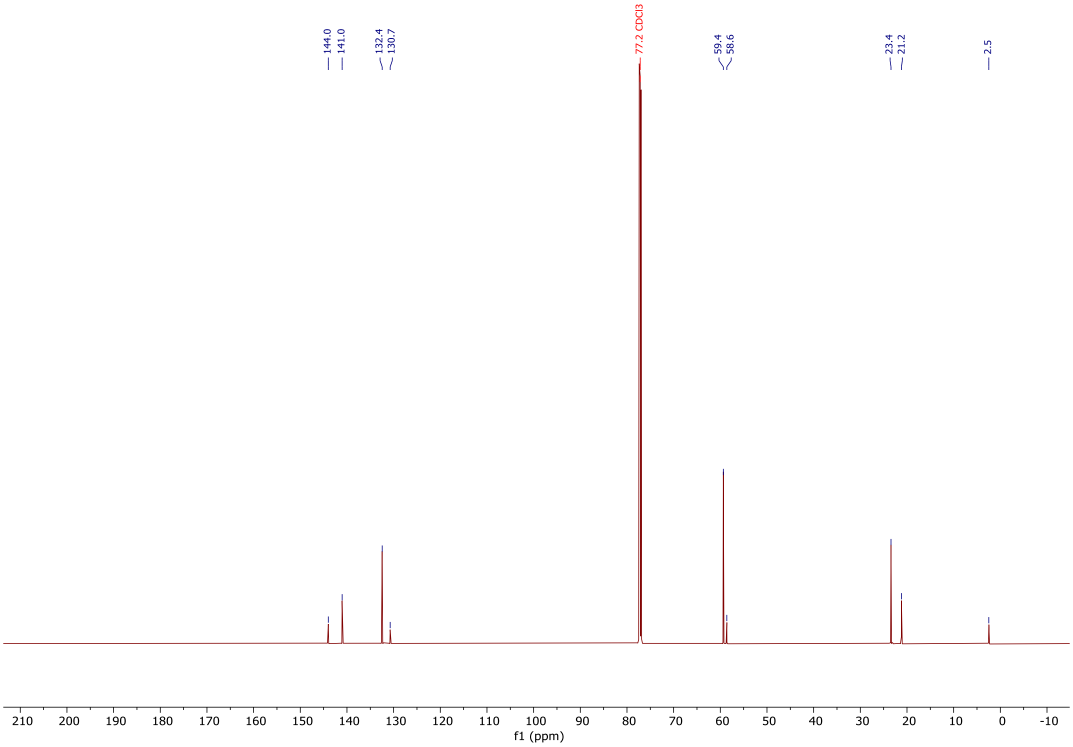

1-Bromo-3-(mesitylsulfonyl)bicyclo[1.1.1]pentane, 3l-Br

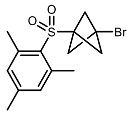

^{1}H NMR (400 MHz, $CDCl_3$)

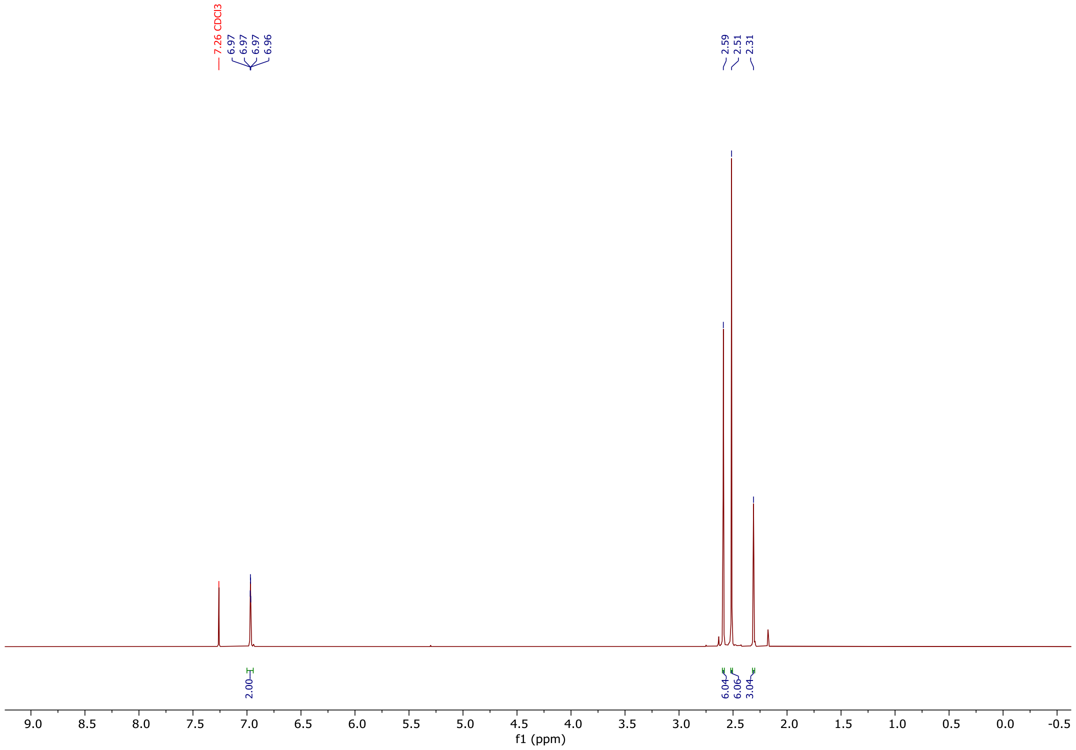

^{13}C NMR (101 MHz, $CDCl_3$)

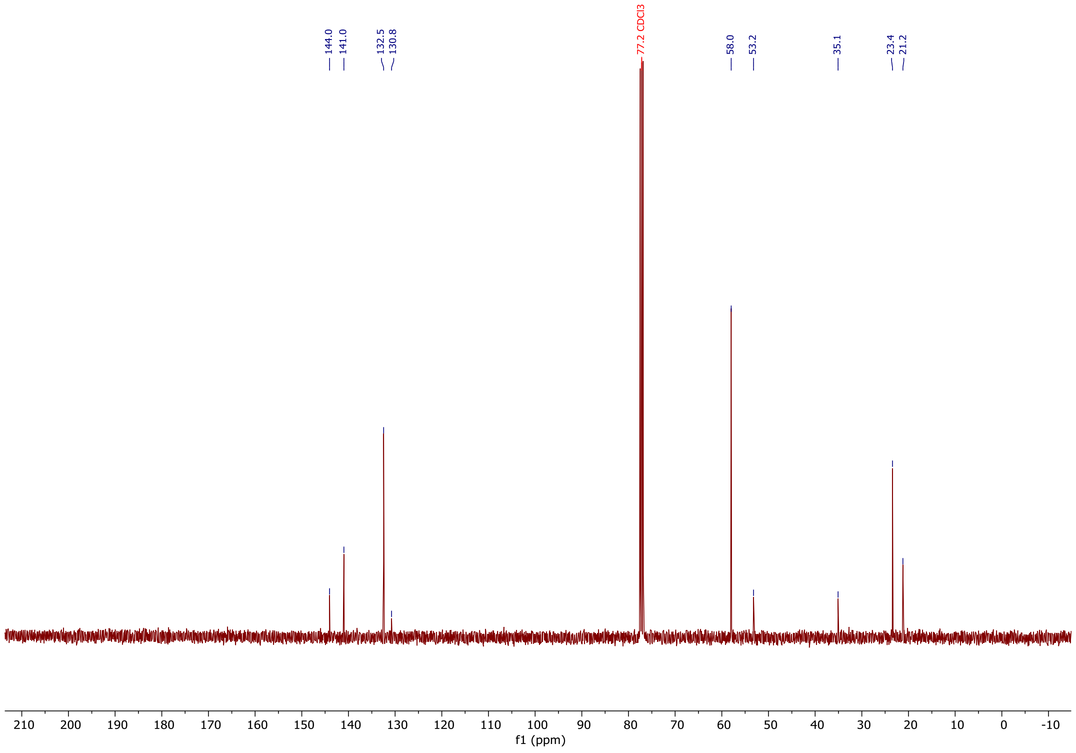

1-((3,5-Difluorophenyl)sulfonyl)-3-iodobicyclo[1.1.1]pentane, 3m-I

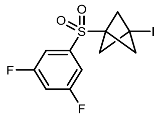

^{1}H **NMR** (400 MHz, $CDCl_3$)

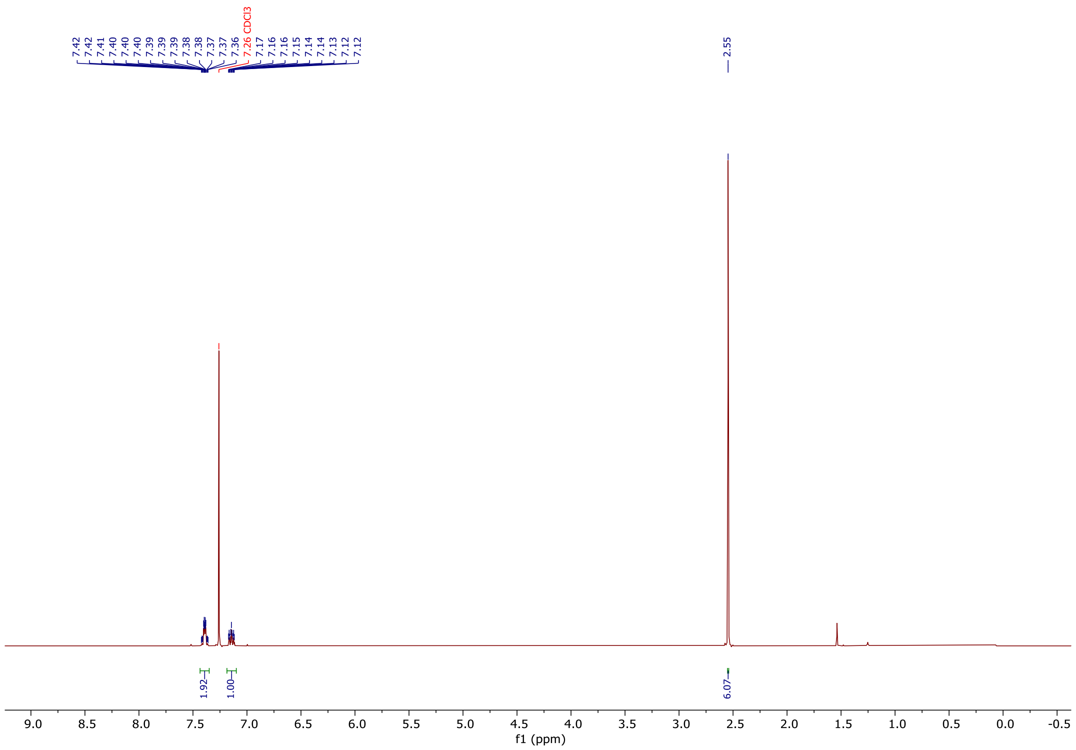

^{13}C **NMR** (151 MHz, $CDCl_3$)

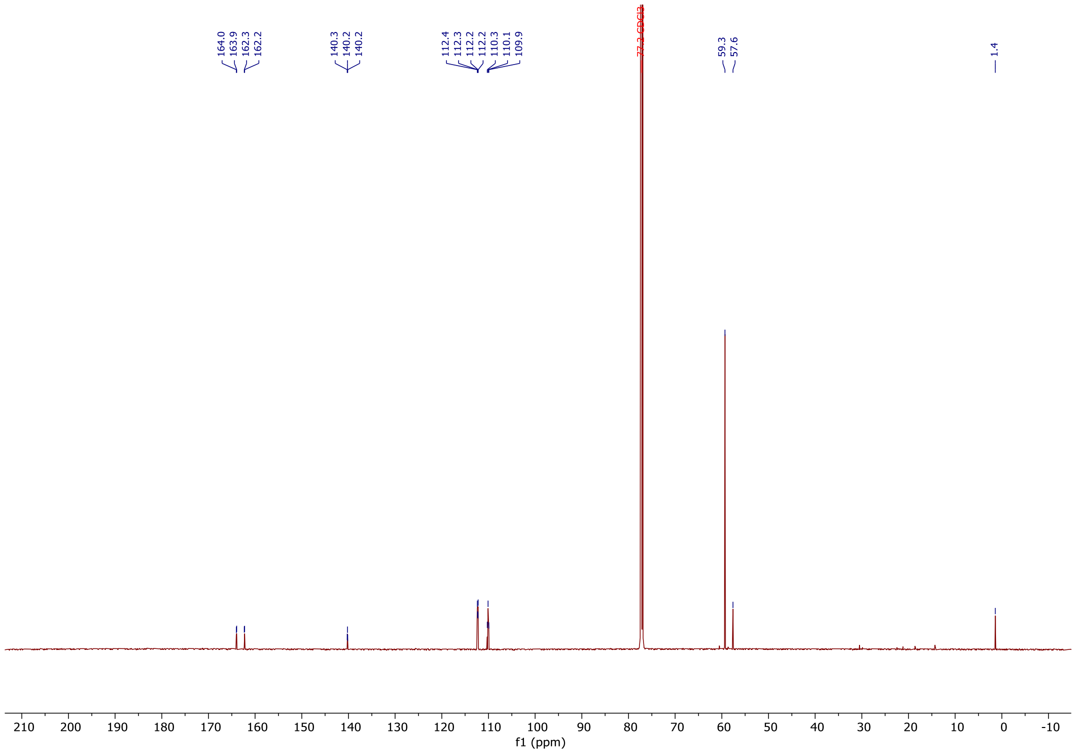

^{19}**F NMR** (377 MHz, $CDCl_3$)

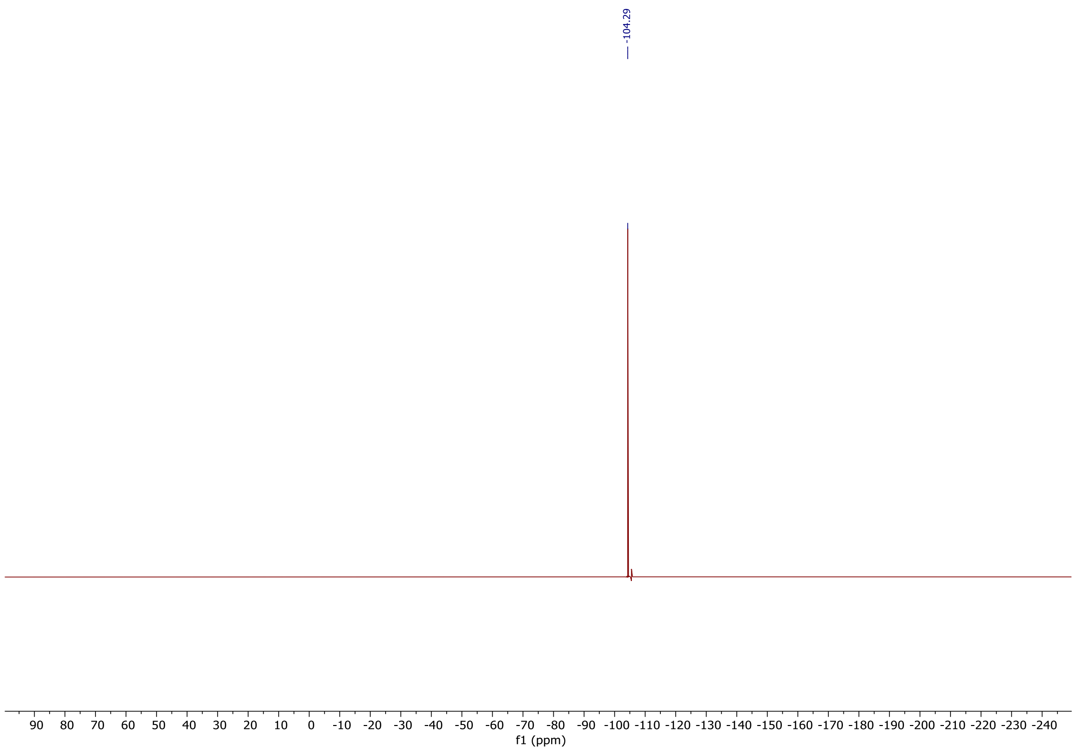

1-Bromo-3-((3,5-difluorophenyl)sulfonyl)bicyclo[1.1.1]pentane, 3m-Br

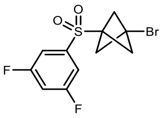

^{1}H NMR (600 MHz, CDCl$_3$)

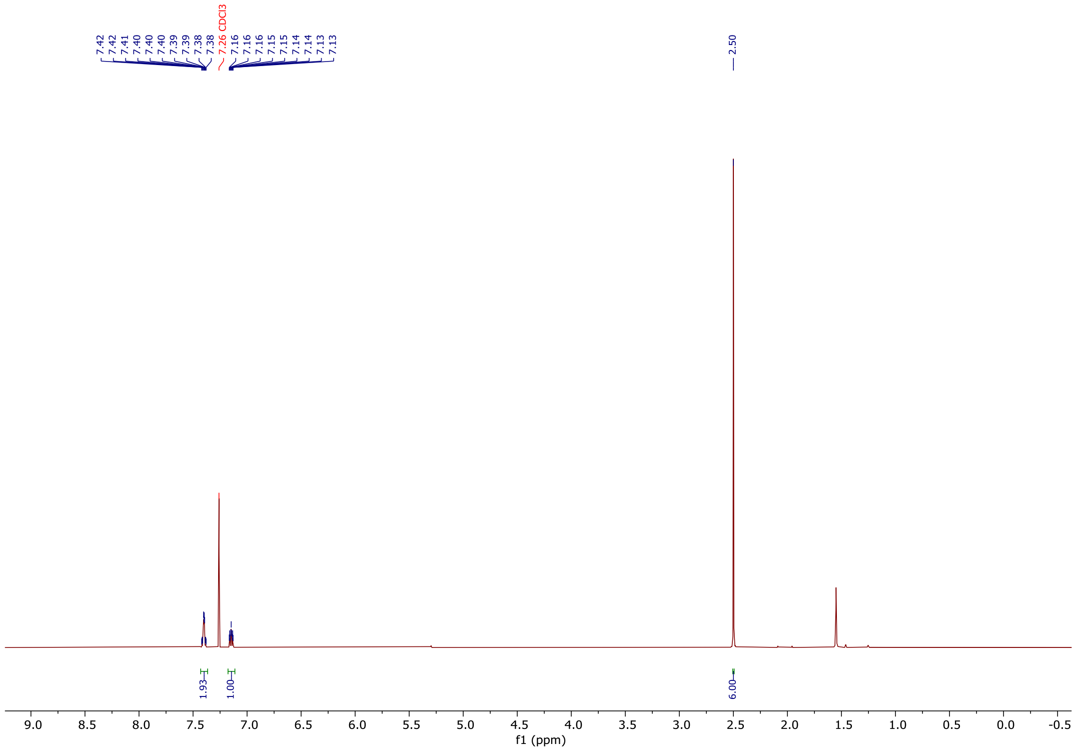

^{13}C NMR (151 MHz, CDCl$_3$)

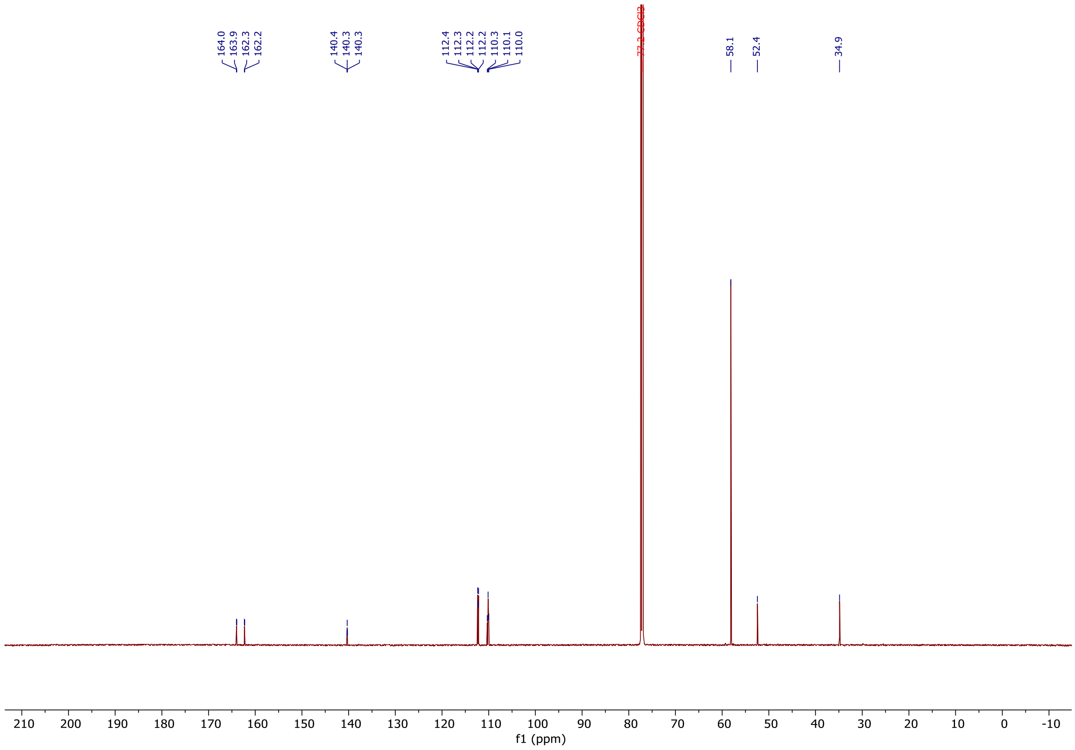

^{19}**F NMR** (377 MHz, $CDCl_3$)

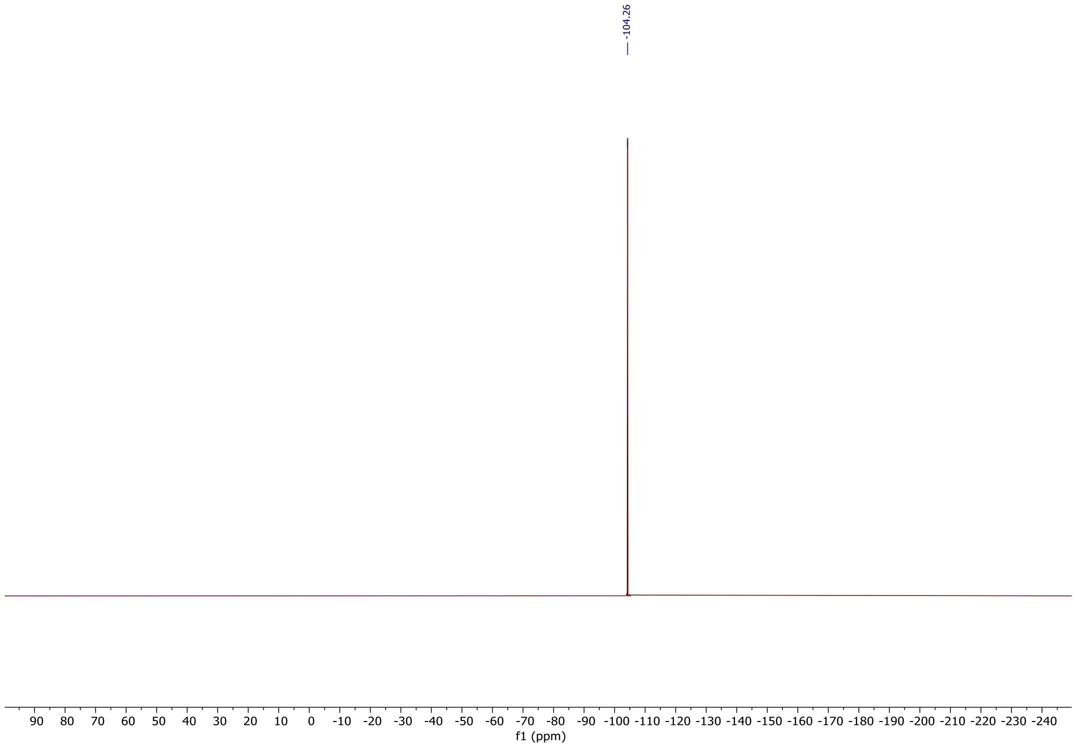

1-((3,5-Bis(trifluoromethyl)phenyl)sulfonyl)-3-iodobicyclo[1.1.1]pentane, 3n-I

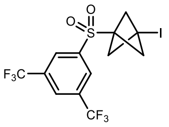

^{1}H NMR (600 MHz, $CDCl_3$)

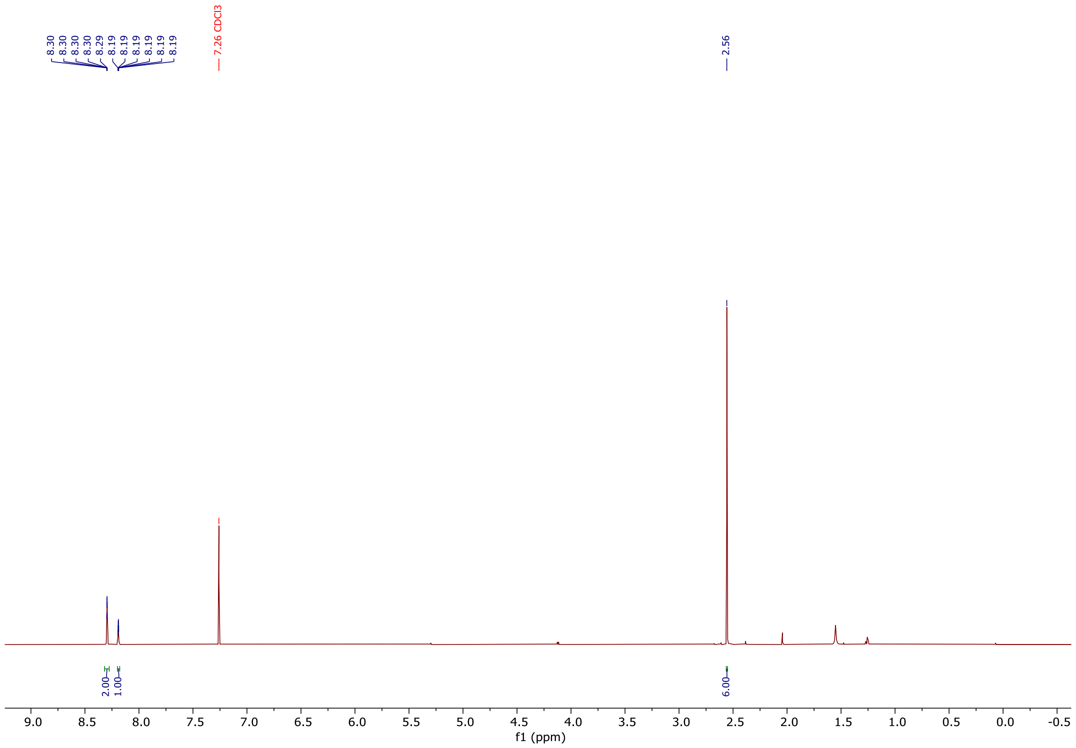

^{13}C NMR (151 MHz, $CDCl_3$)

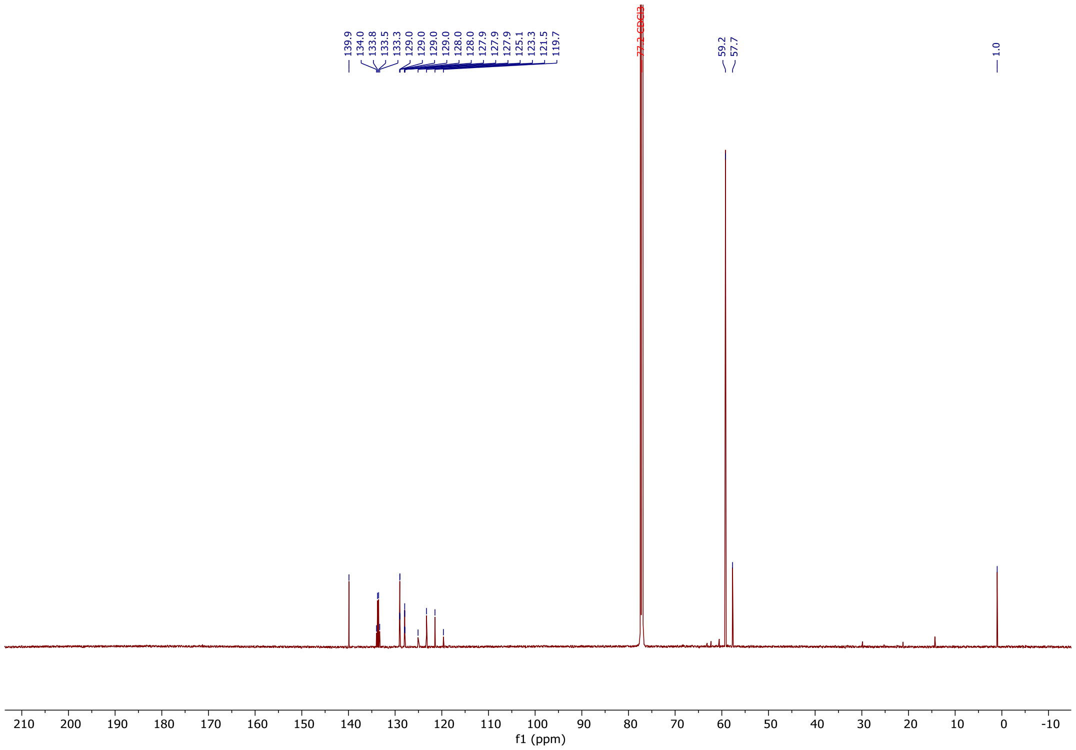

^{19}**F NMR** (377 MHz, $CDCl_3$)

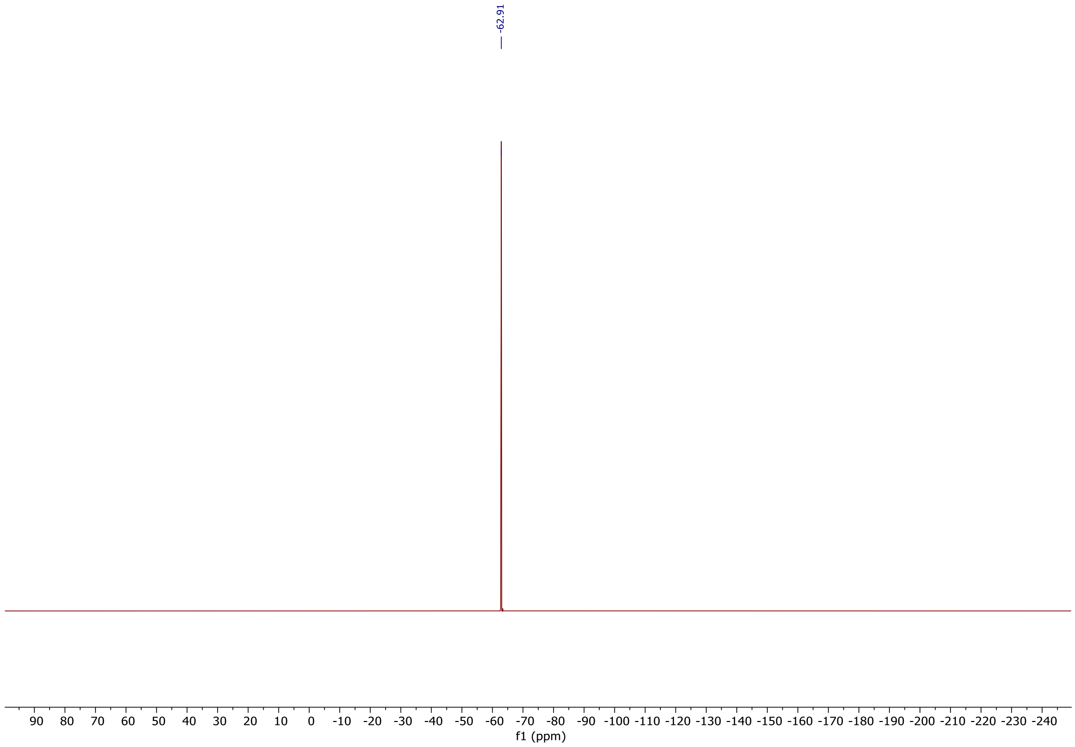

1-((3,5-Difluorophenyl)sulfonyl)-3-iodobicyclo[1.1.1]pentane, 3n-Br

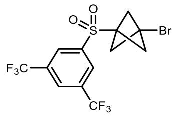

^{1}H NMR (400 MHz, $CDCl_3$)

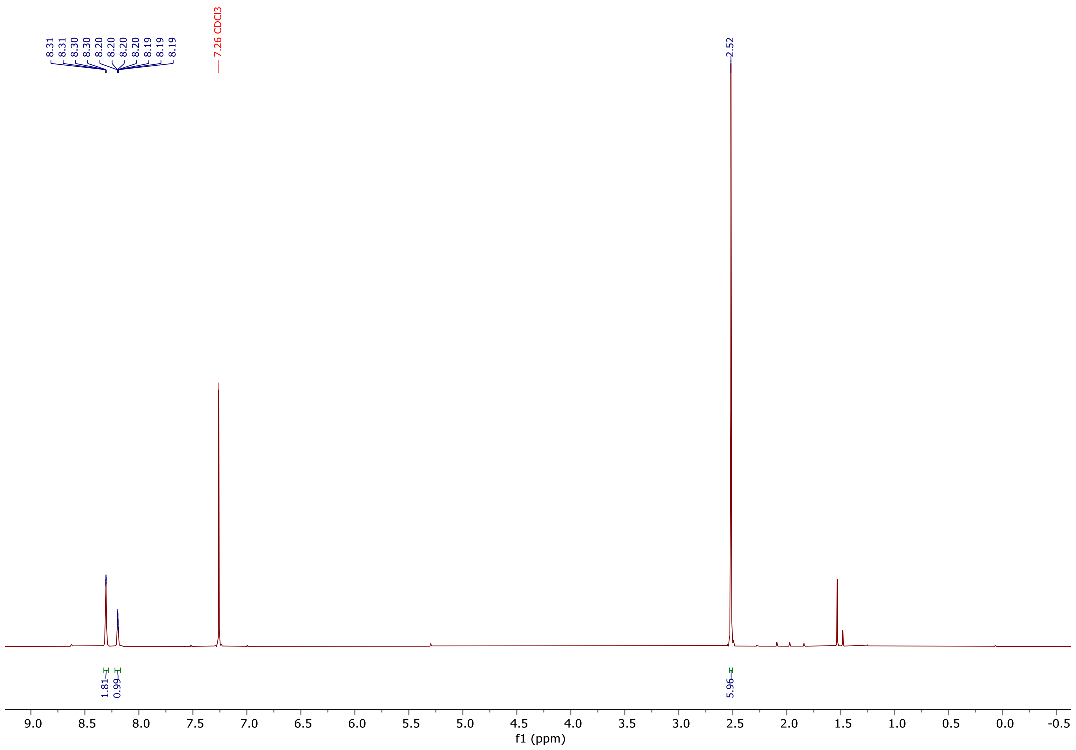

^{13}C NMR (151 MHz, $CDCl_3$)

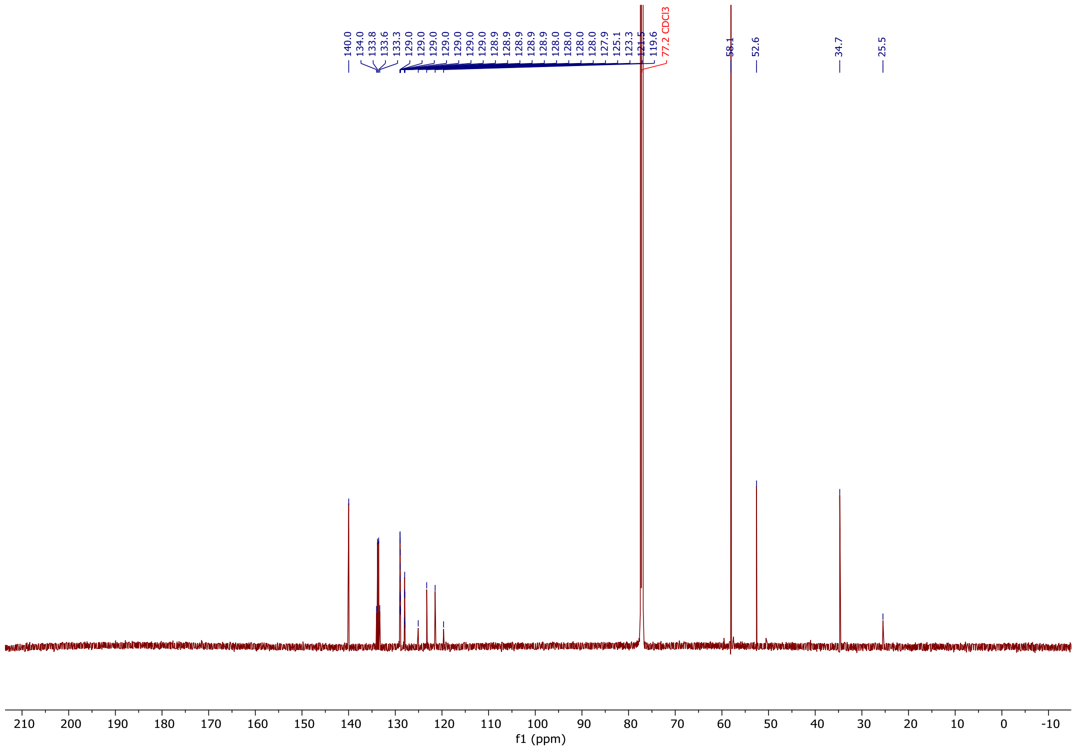

^{19}**F NMR** (377 MHz, $CDCl_3$)

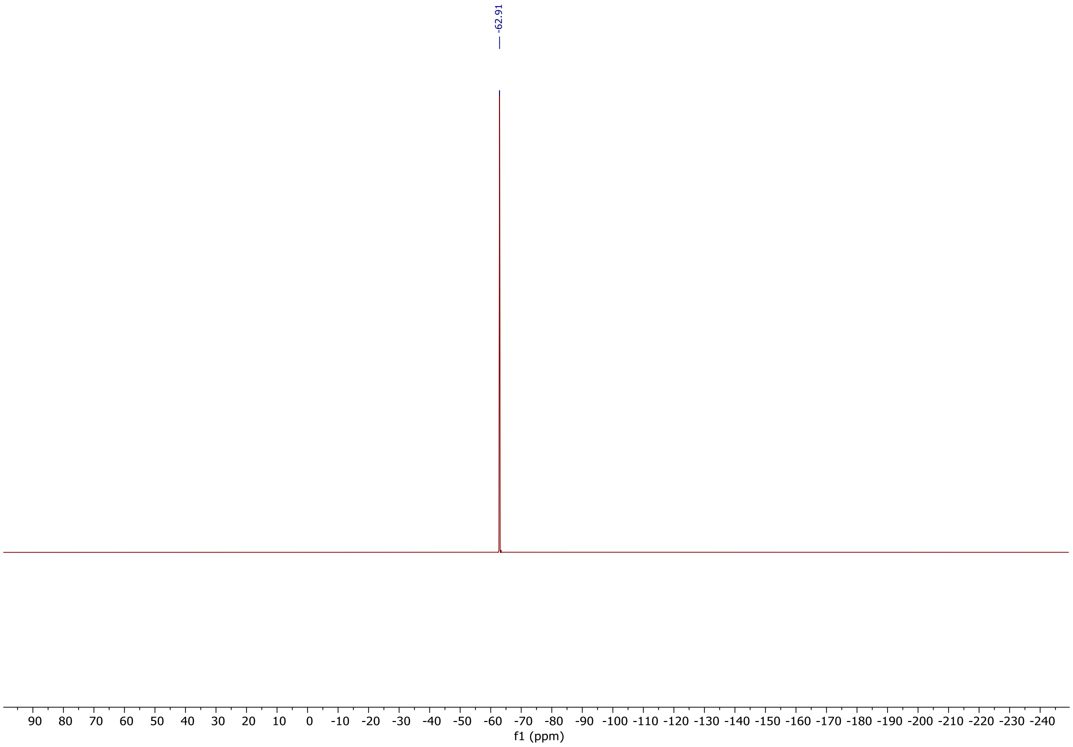

N-(4-((3-Iodobicyclo[1.1.1]pentan-1-yl)sulfonyl)phenyl)acetamide, 3o-Br

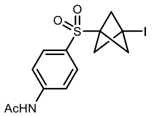

^{1}H NMR (600 MHz, $CDCl_3$)

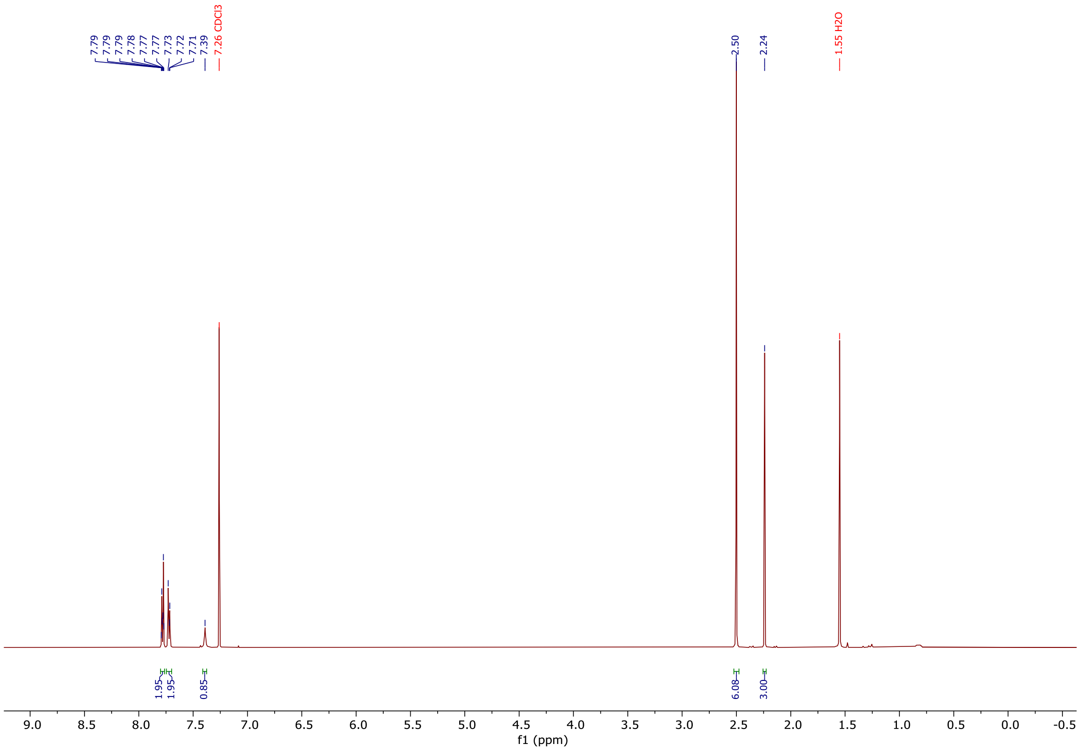

^{13}C NMR (151 MHz, $CDCl_3$)

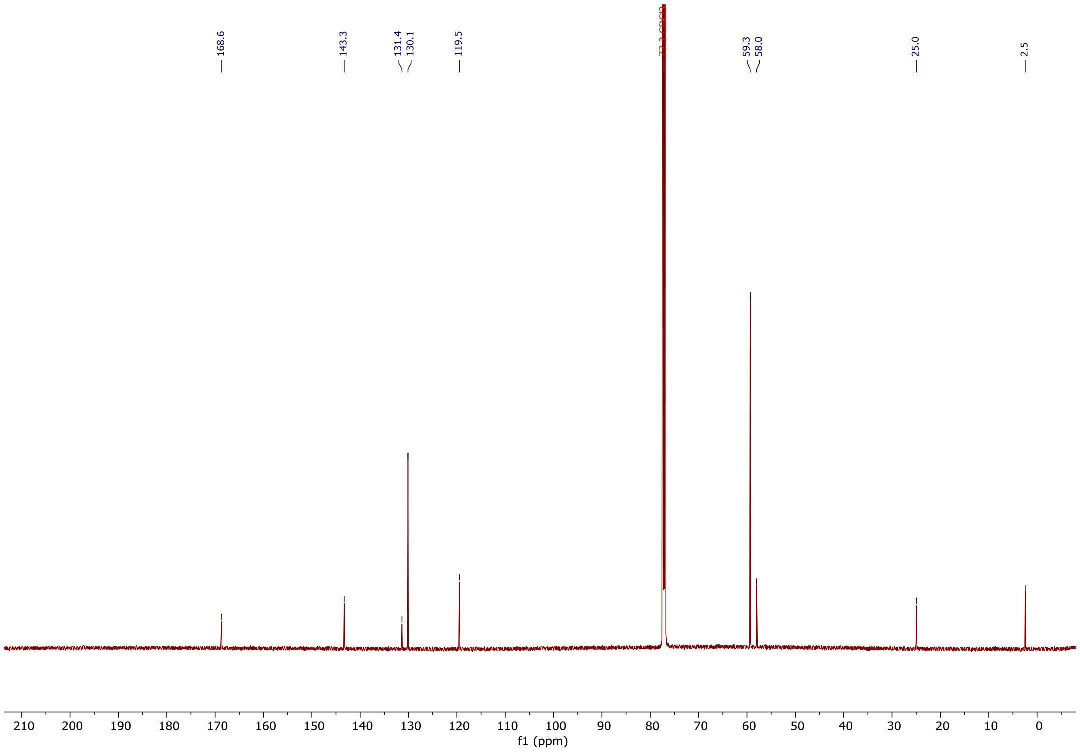

N-(4-((3-Bromobicyclo[1.1.1]pentan-1-yl)sulfonyl)phenyl)acetamide, 3o-Br

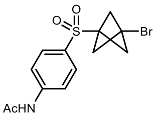

^{1}H NMR (400 MHz, $CDCl_3$)

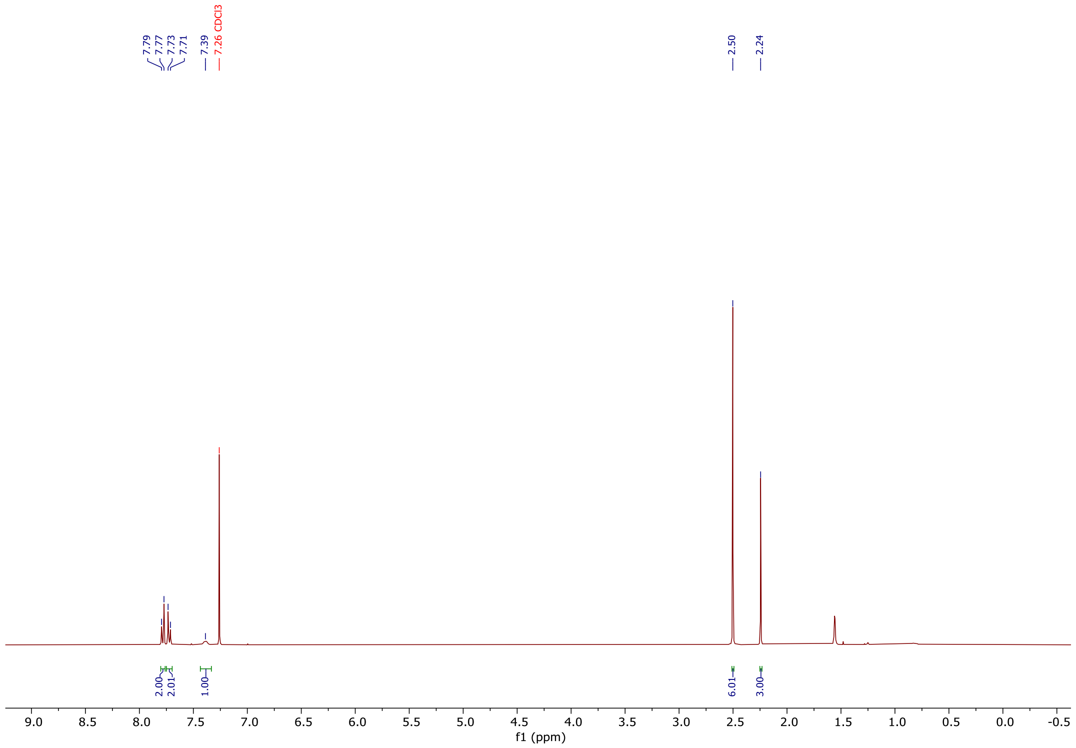

^{13}C NMR (151 MHz, $CDCl_3$)

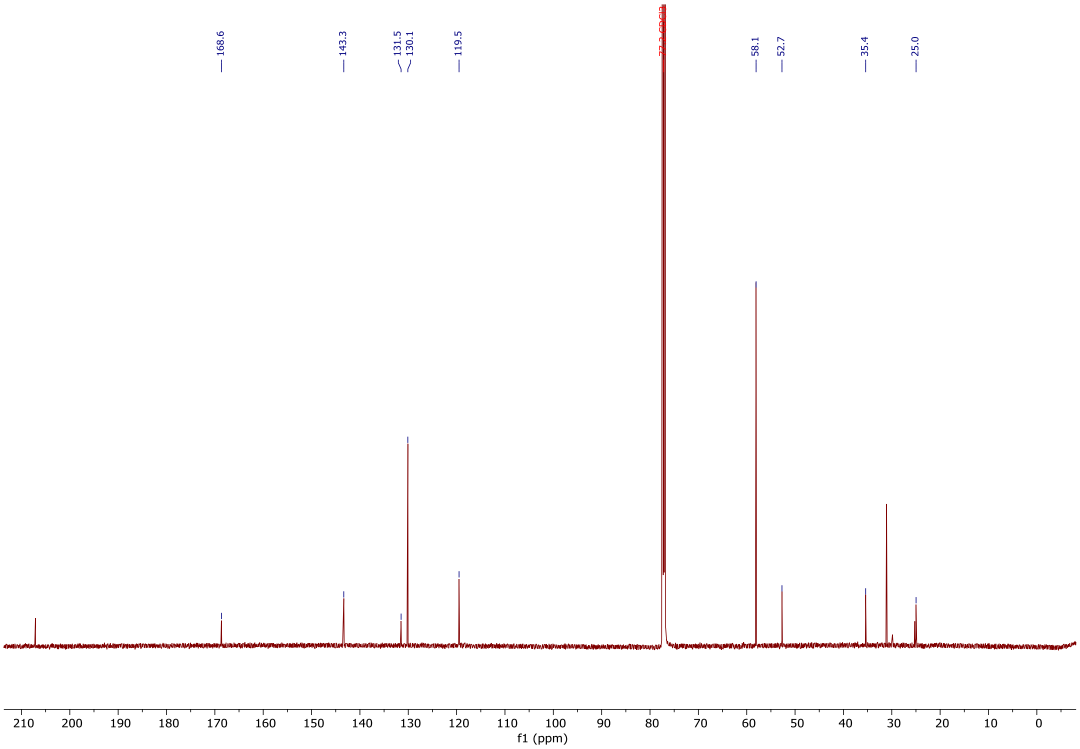

1,3-Bis((3-iodobicyclo[1.1.1]pentan-1-yl)sulfonyl)benzene, 3p-I

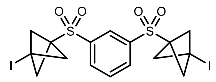

^{1}H NMR (600 MHz, $CDCl_3$)

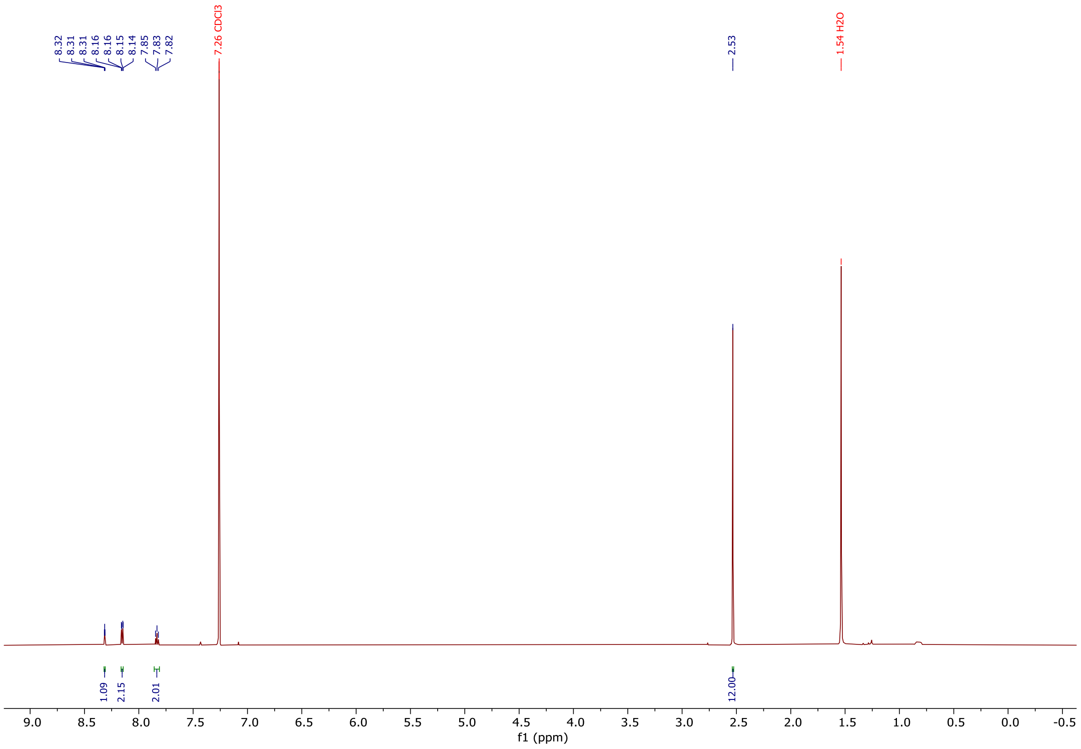

^{13}C NMR (151 MHz, $CDCl_3$)

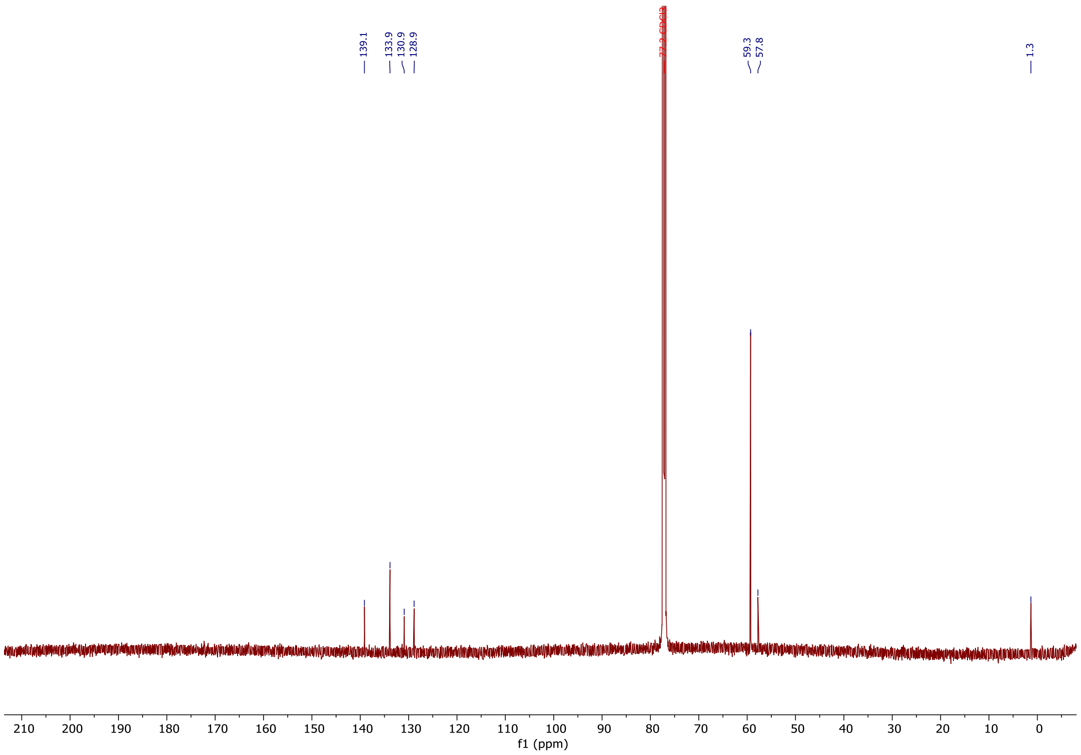

1,3-Bis((3-bromobicyclo[1.1.1]pentan-1-yl)sulfonyl)benzene, 3p-Br

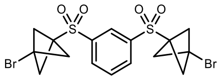

^{1}H NMR (400 MHz, $CDCl_3$)

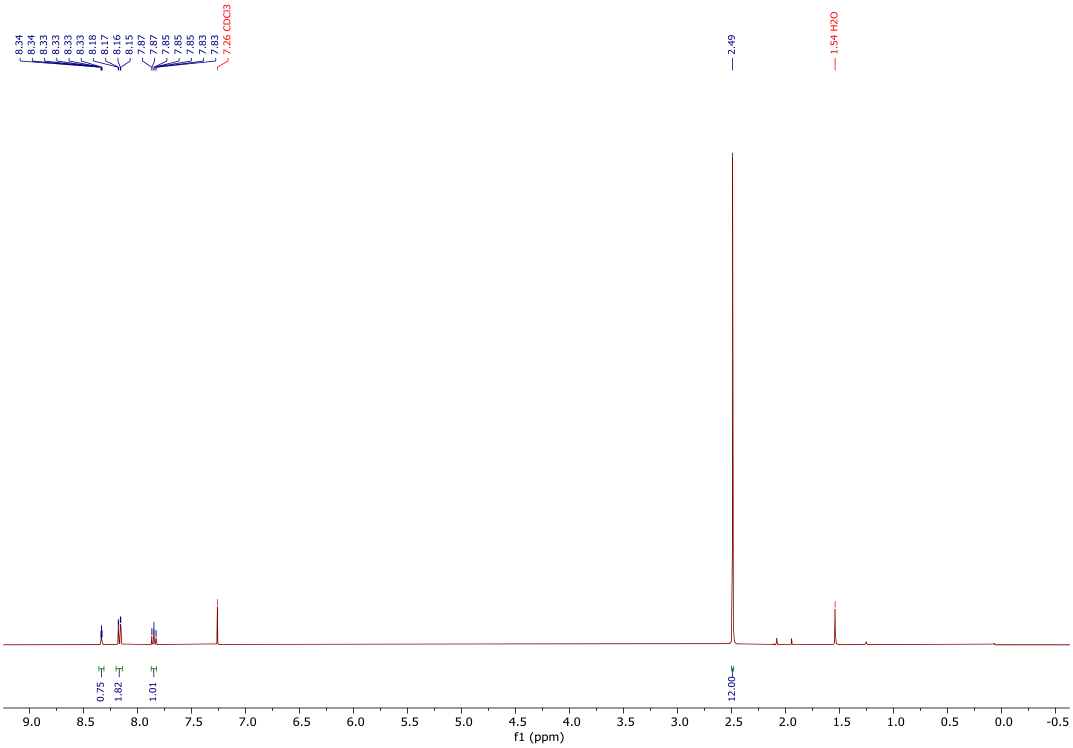

^{13}C NMR (101 MHz, $CDCl_3$)

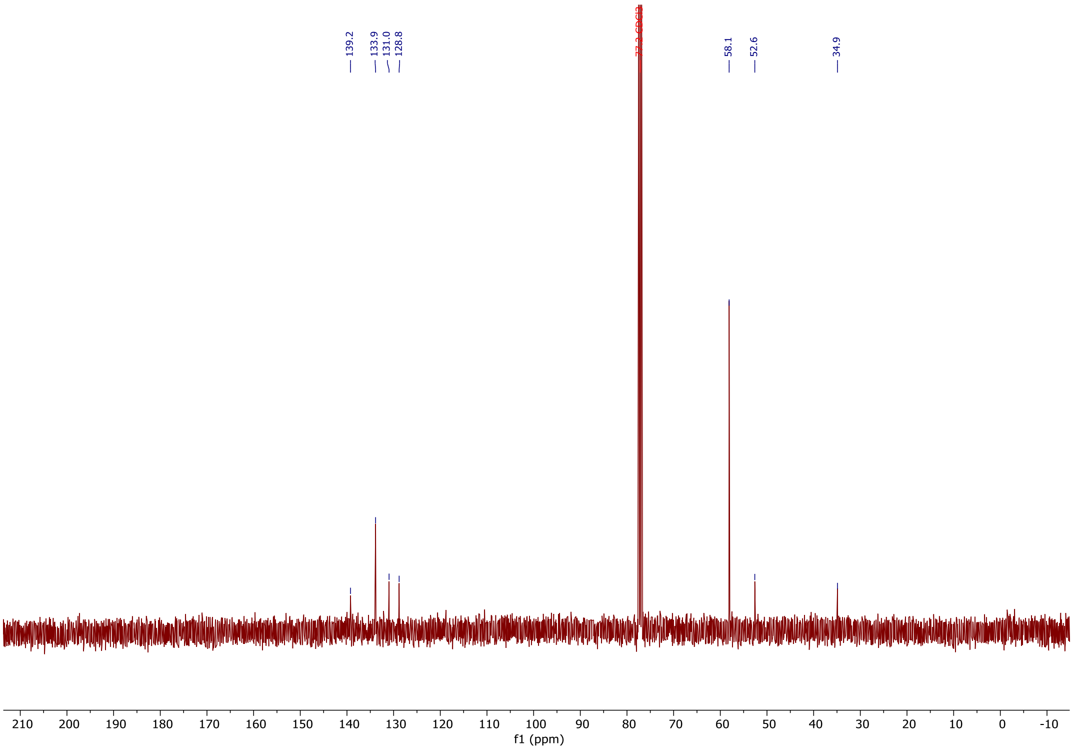

1-Iodo-5-tosylbicyclo[3.1.1]heptane, 5a-I

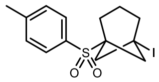

^{1}H NMR (400 MHz, $CDCl_3$)

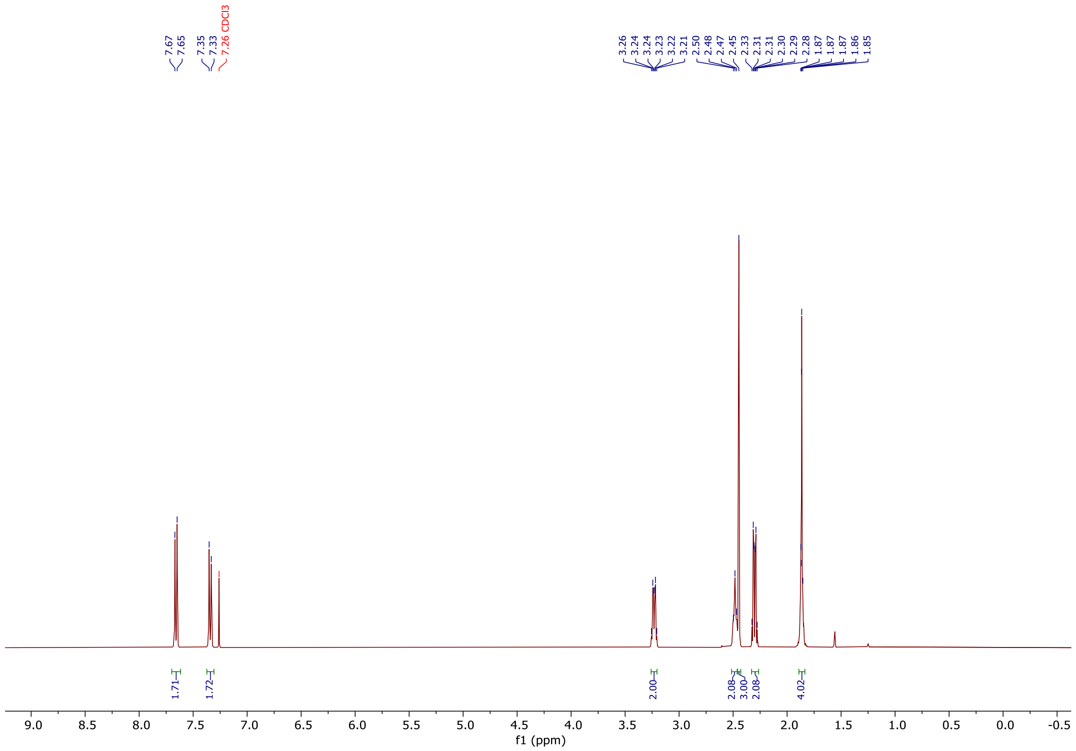

^{13}C NMR (101 MHz, $CDCl_3$)

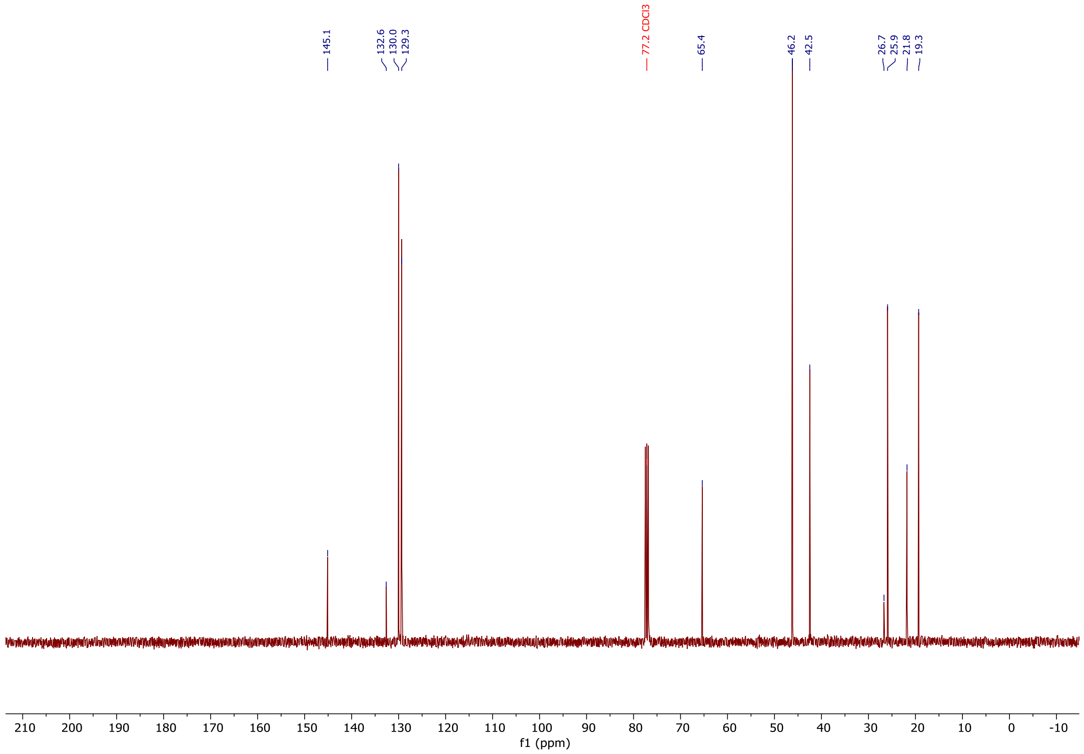

1-Bromo-5-tosylbicyclo[3.1.1]heptane, 5a-Br

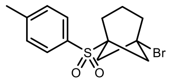

^{1}H NMR (400 MHz, CDCl$_3$)

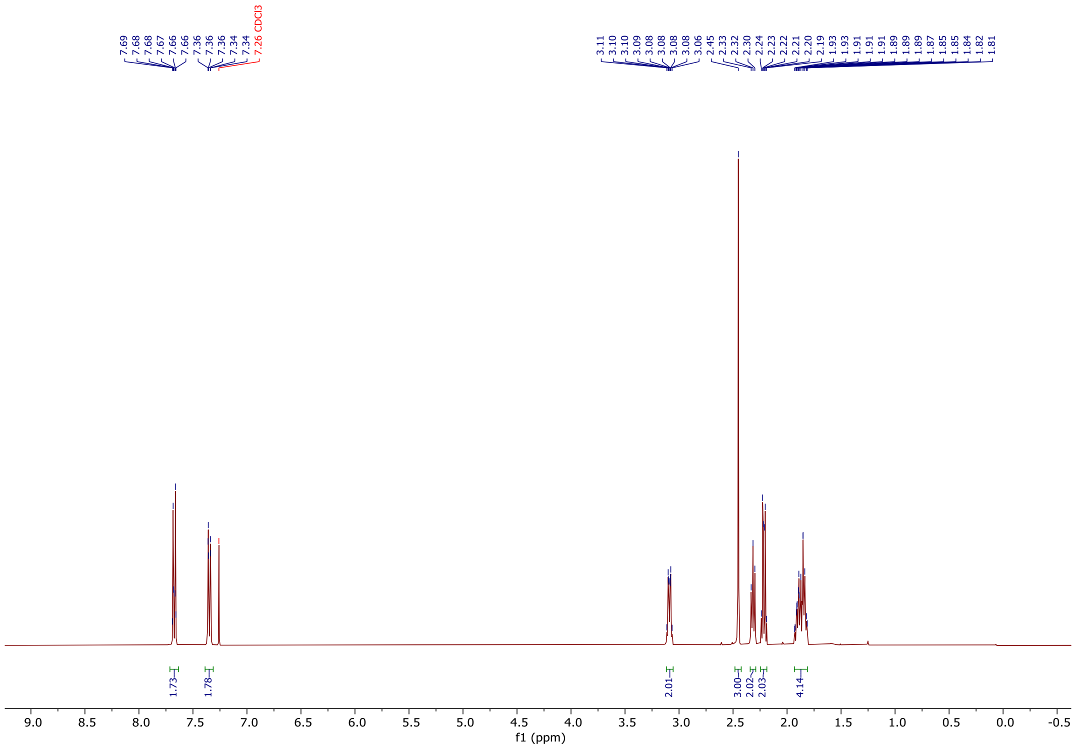

^{13}C NMR (101 MHz, CDCl$_3$)

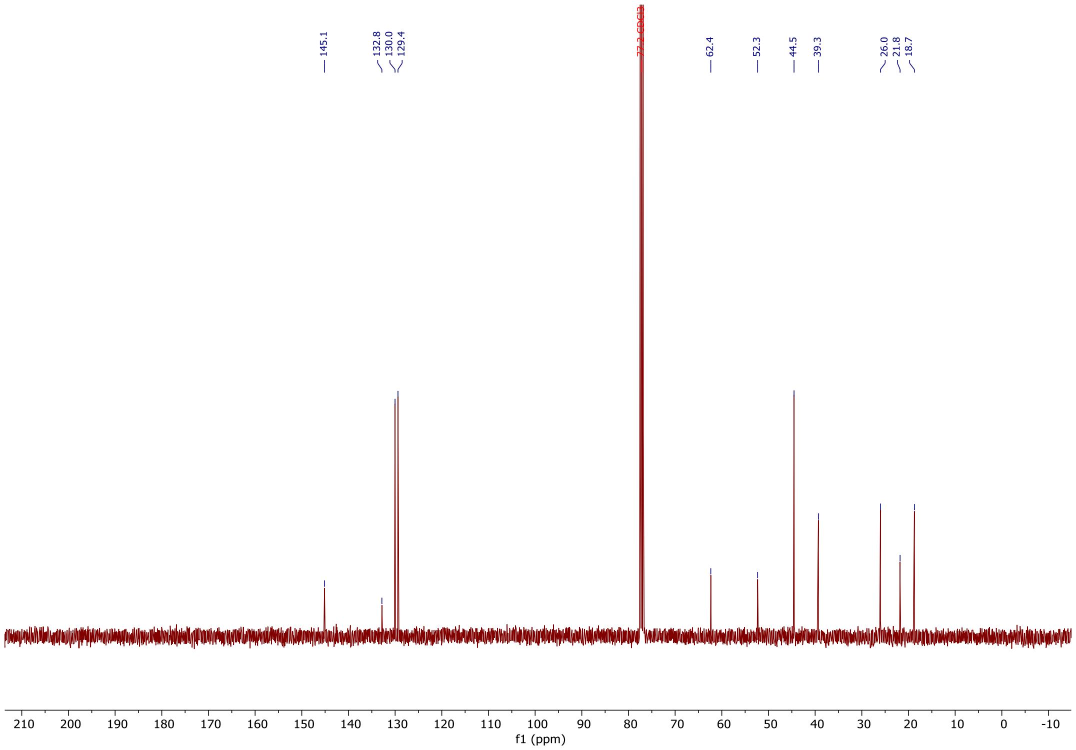

6.2 Heteroaryl BCP Iodides

2-((3-Iodobicyclo[1.1.1]pentan-1-yl)sulfonyl)thiophene, 3q-I

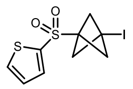

^{1}H NMR (400 MHz, $CDCl_3$)

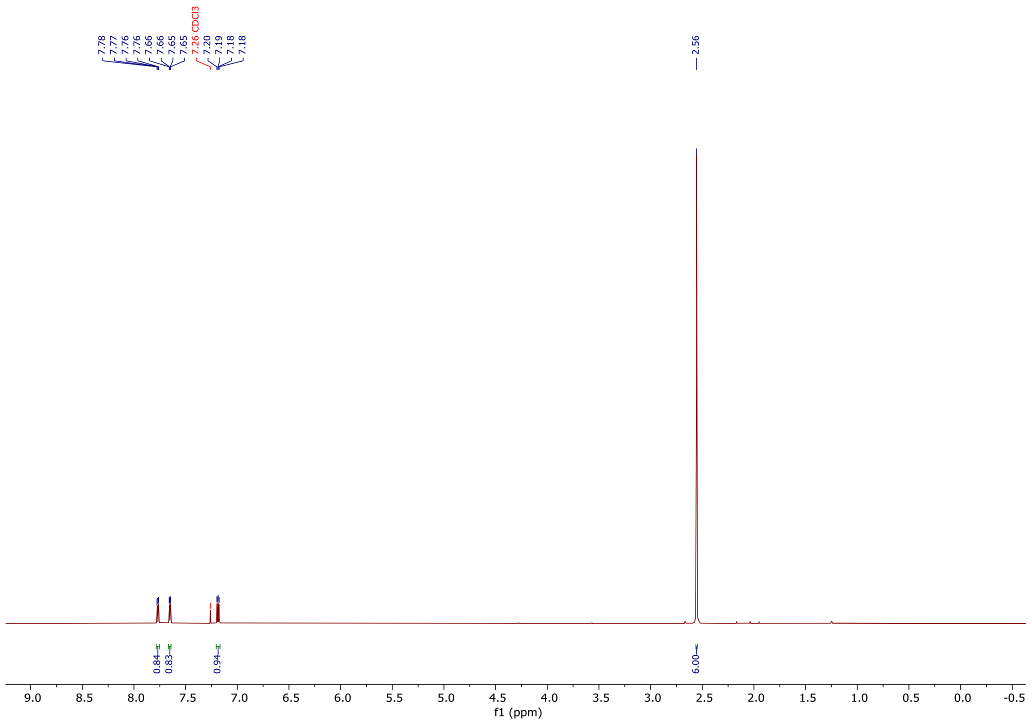

^{13}C NMR (101 MHz, $CDCl_3$)

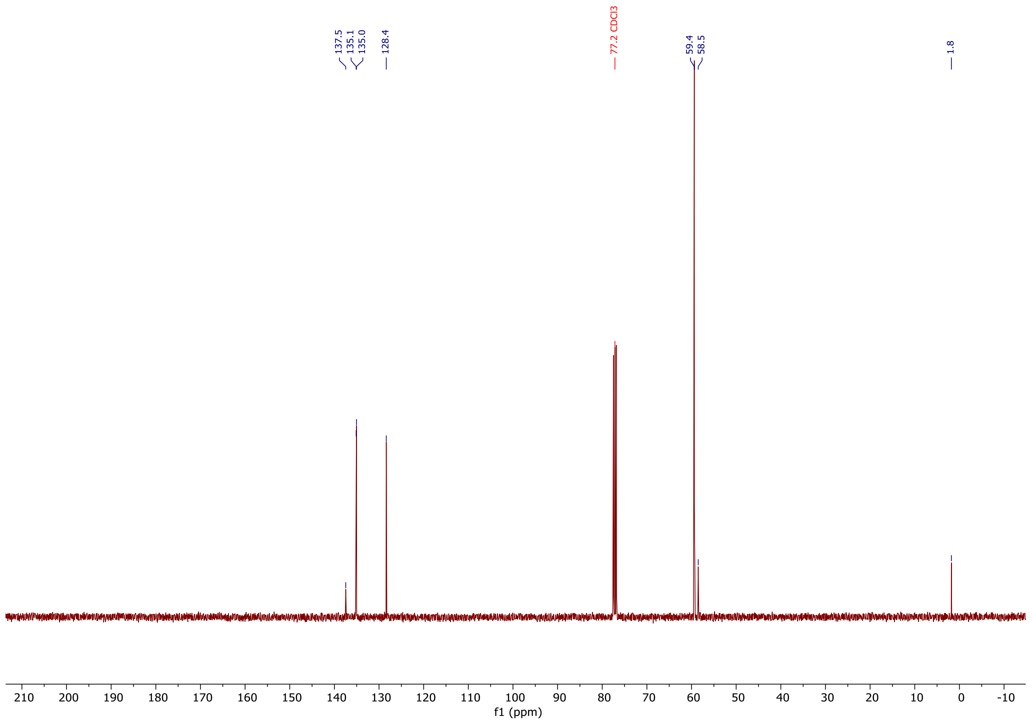

2-((3-Bromobicyclo[1.1.1]pentan-1-yl)sulfonyl)thiophene, 3q-Br

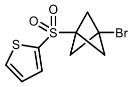

^{1}H NMR (400 MHz, $CDCl_3$)

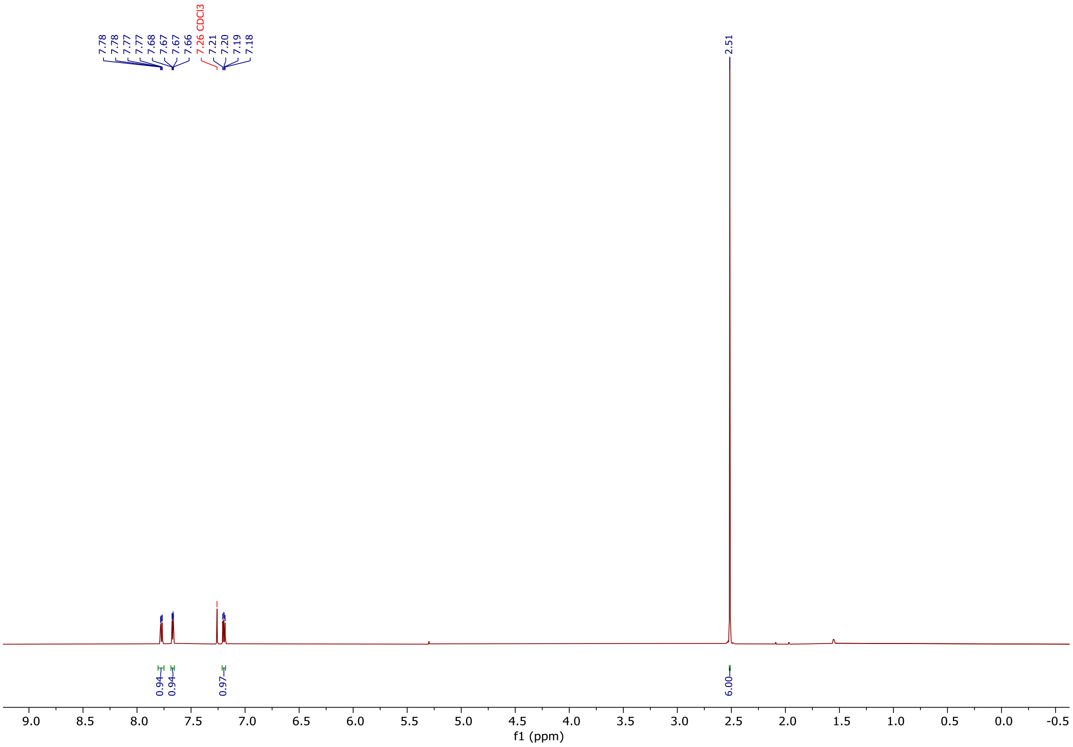

^{13}C NMR (151 MHz, $CDCl_3$)

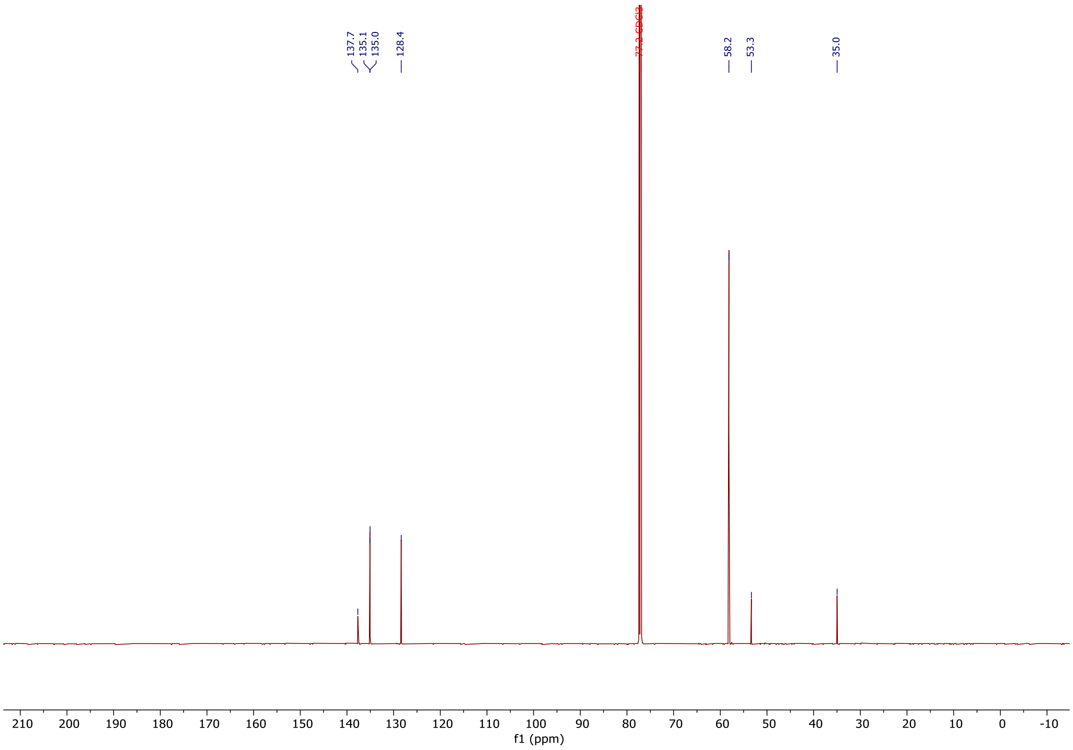

2-Bromo-5-((3-iodobicyclo[1.1.1]pentan-1-yl)sulfonyl)thiophene, 3r-I

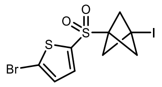

^{1}H NMR (600 MHz, $CDCl_3$)

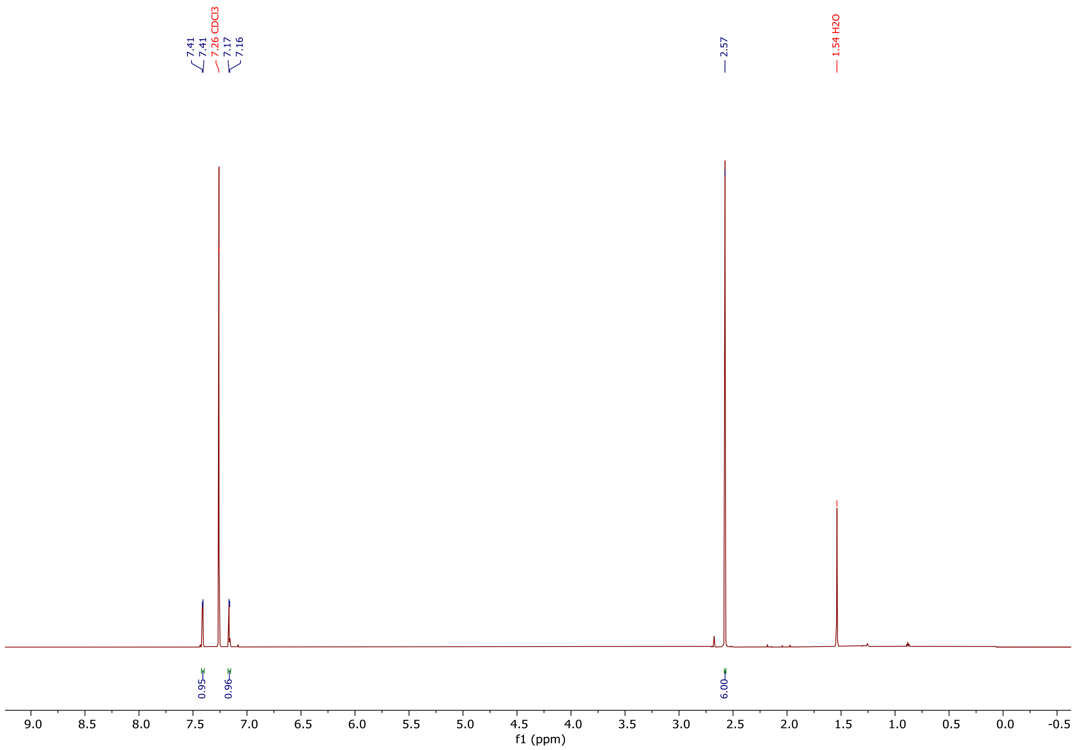

^{13}C NMR (151 MHz, $CDCl_3$)

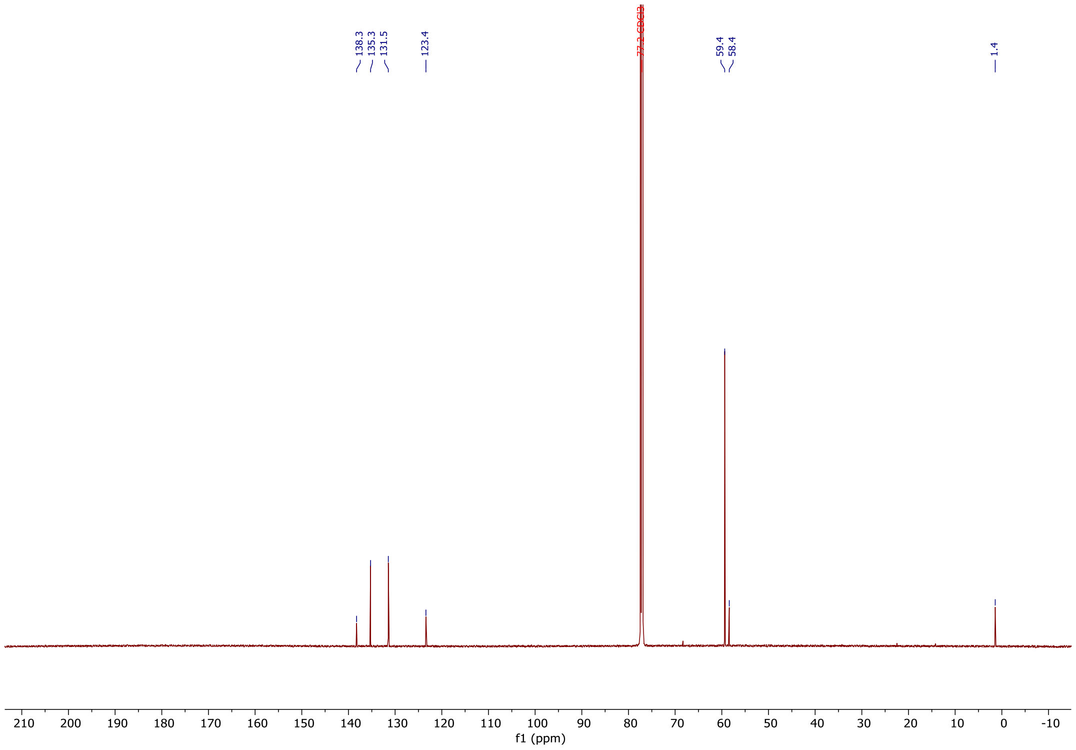

2-Bromo-5-((3-bromobicyclo[1.1.1]pentan-1-yl)sulfonyl)thiophene, 3r-Br

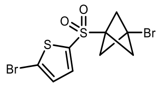

^{1}H NMR (600 MHz, $CDCl_3$)

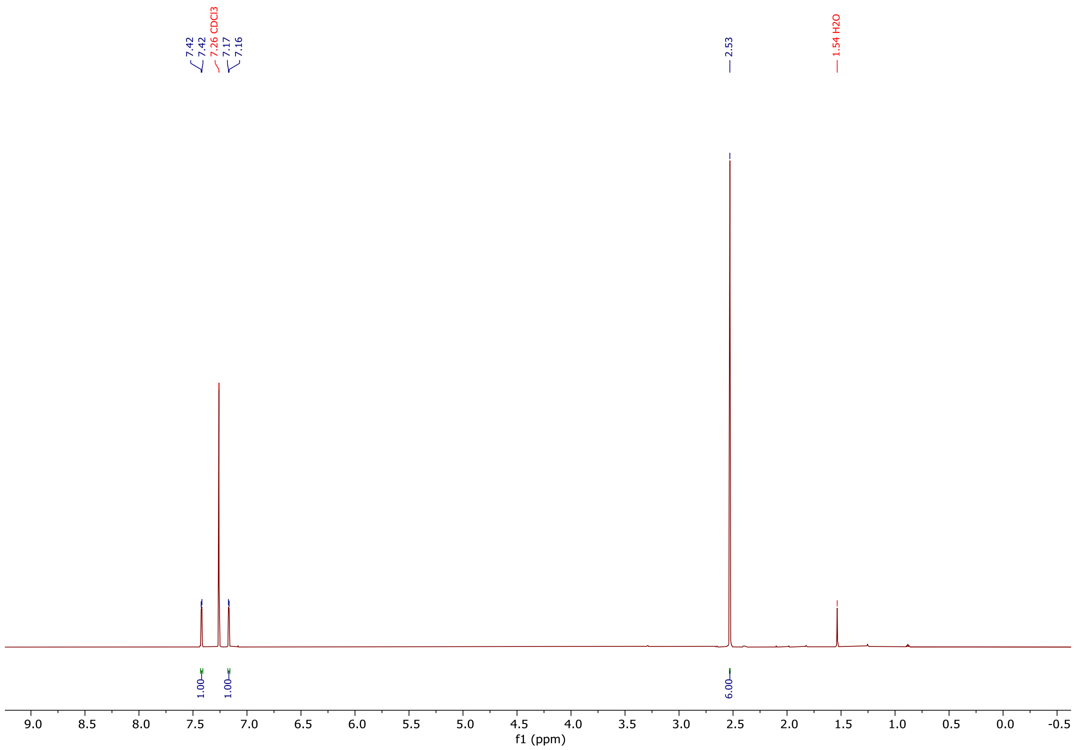

^{13}C NMR (151 MHz, $CDCl_3$)

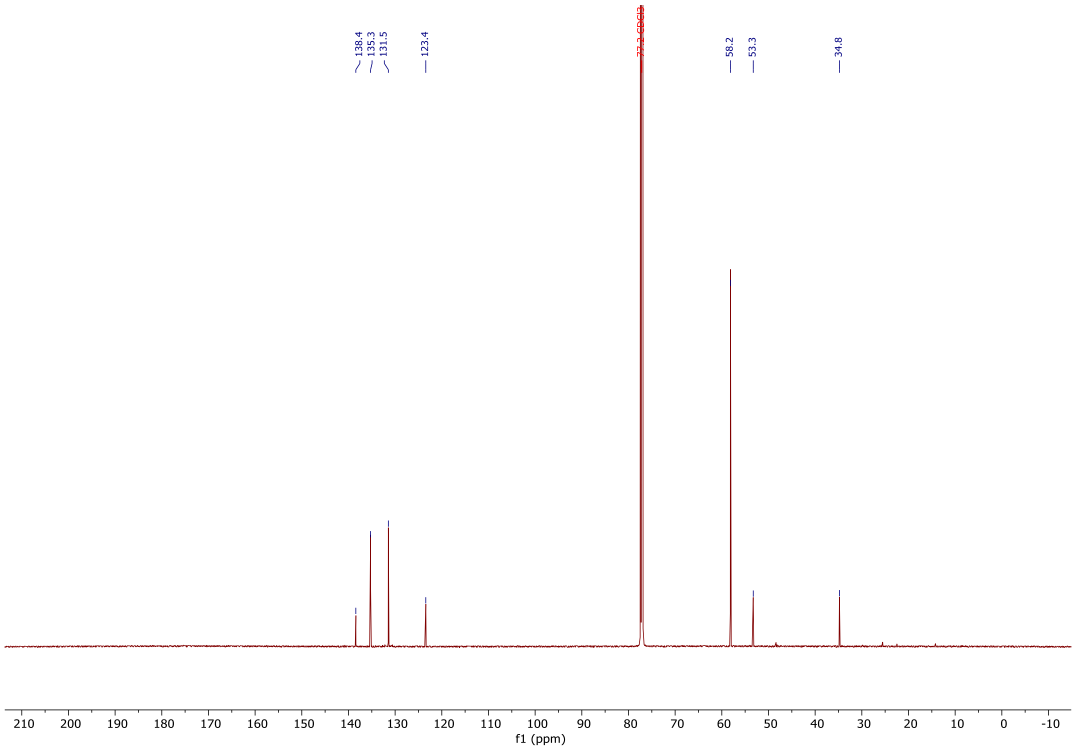

3-((3-Iodobicyclo[1.1.1]pentan-1-yl)sulfonyl)-1-methyl-1H-pyrazole, 3s-I

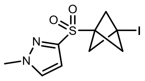

^{1}H NMR (600 MHz, $CDCl_3$)

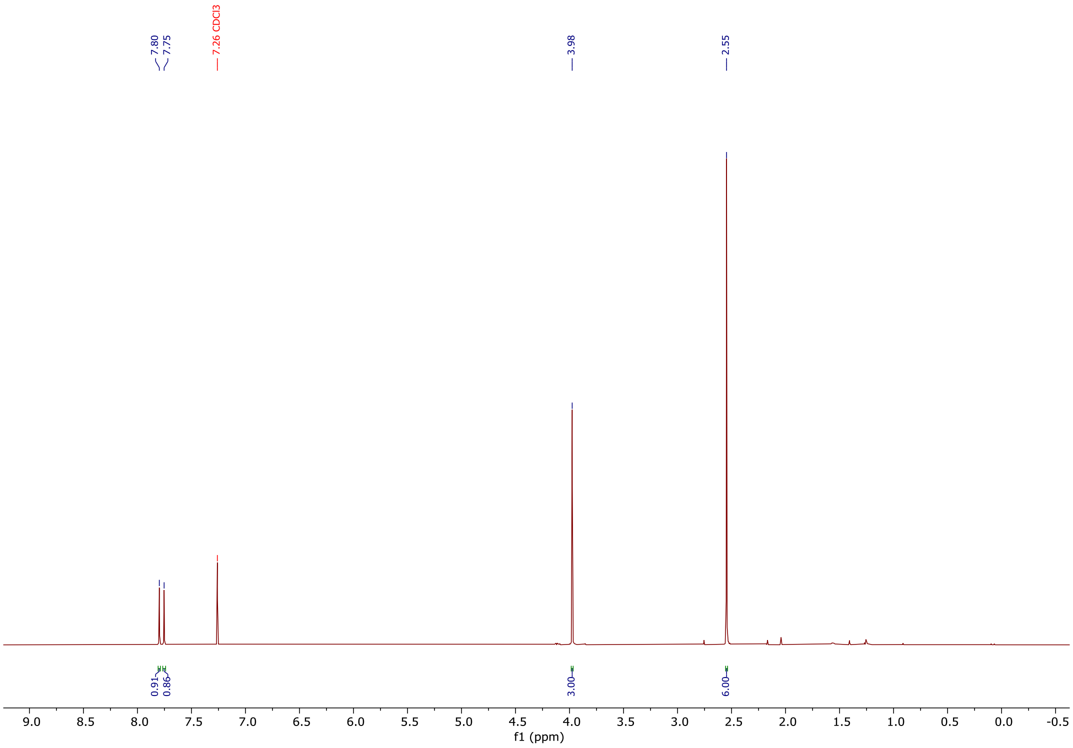

^{13}C NMR (151 MHz, $CDCl_3$)

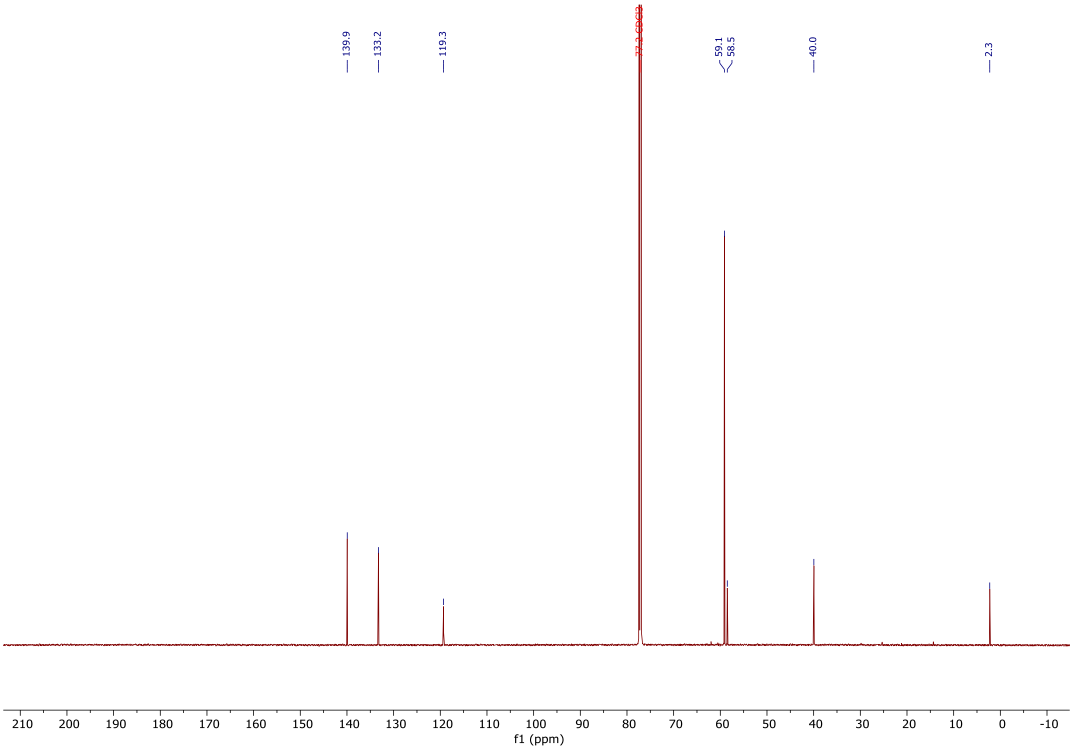

3-((3-Bromobicyclo[1.1.1]pentan-1-yl)sulfonyl)-1-methyl-1H-pyrazole, 3s-Br

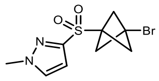

^{1}H NMR (600 MHz, $CDCl_3$)

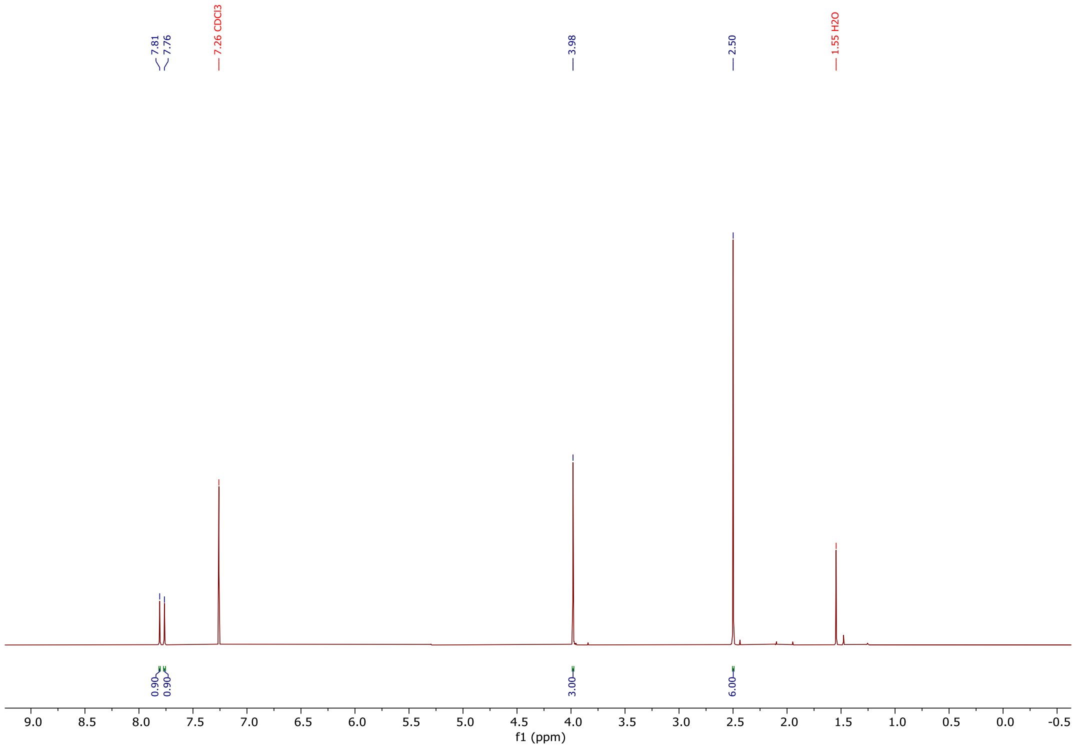

^{13}C NMR (151 MHz, $CDCl_3$)

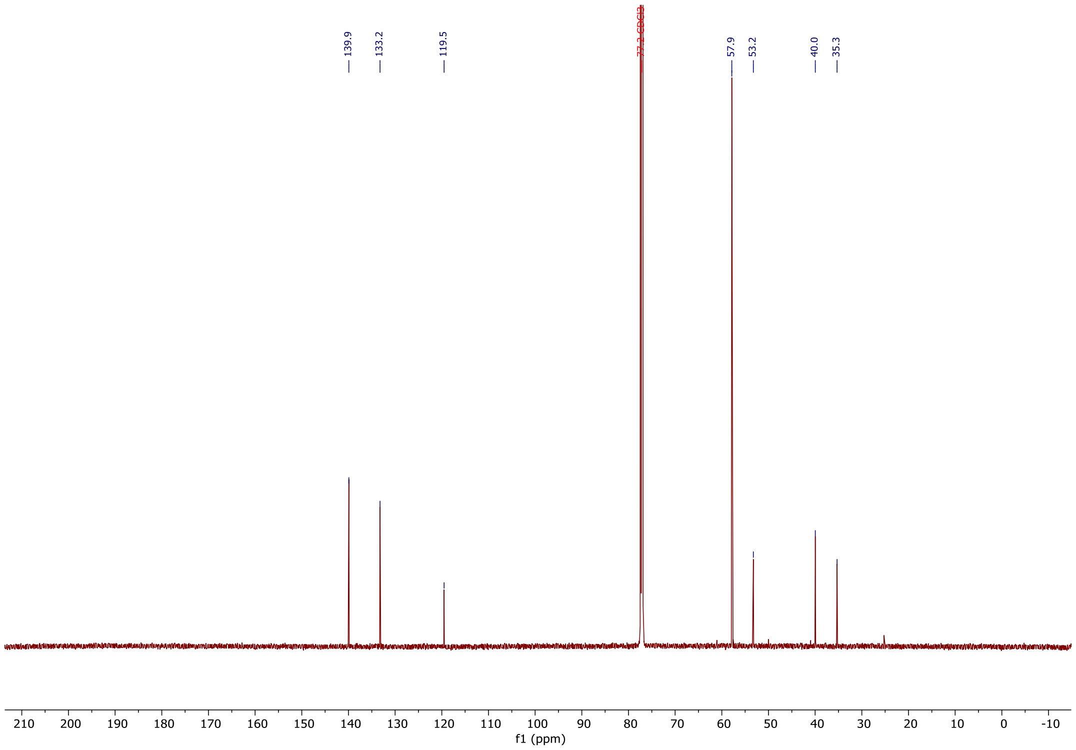

4-((3-Bromobicyclo[1.1.1]pentan-1-yl)sulfonyl)-1-methyl-1H-pyrazole, 3t-Br

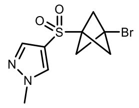

^{1}H NMR (500 MHz, $CDCl_3$)

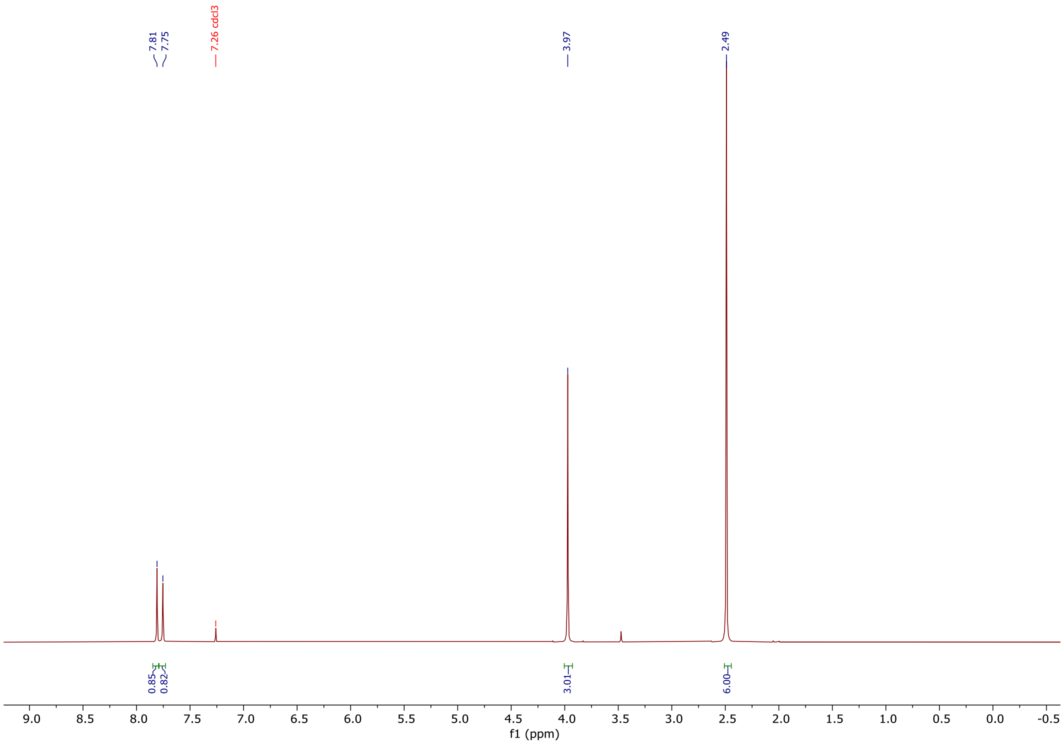

^{13}C NMR (151 MHz, $CDCl_3$)

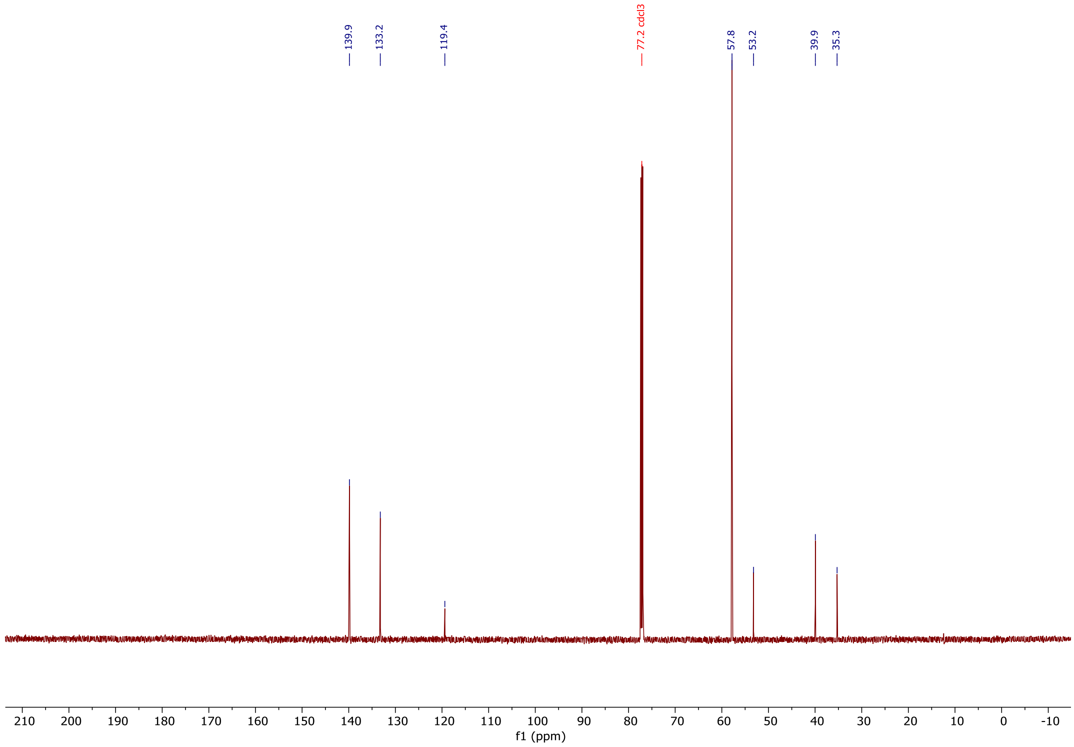

3-((3-Bromobicyclo[1.1.1]pentan-1-yl)sulfonyl)furan, 3u-Br

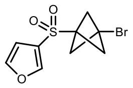

^{1}H NMR (500 MHz, $CDCl_3$)

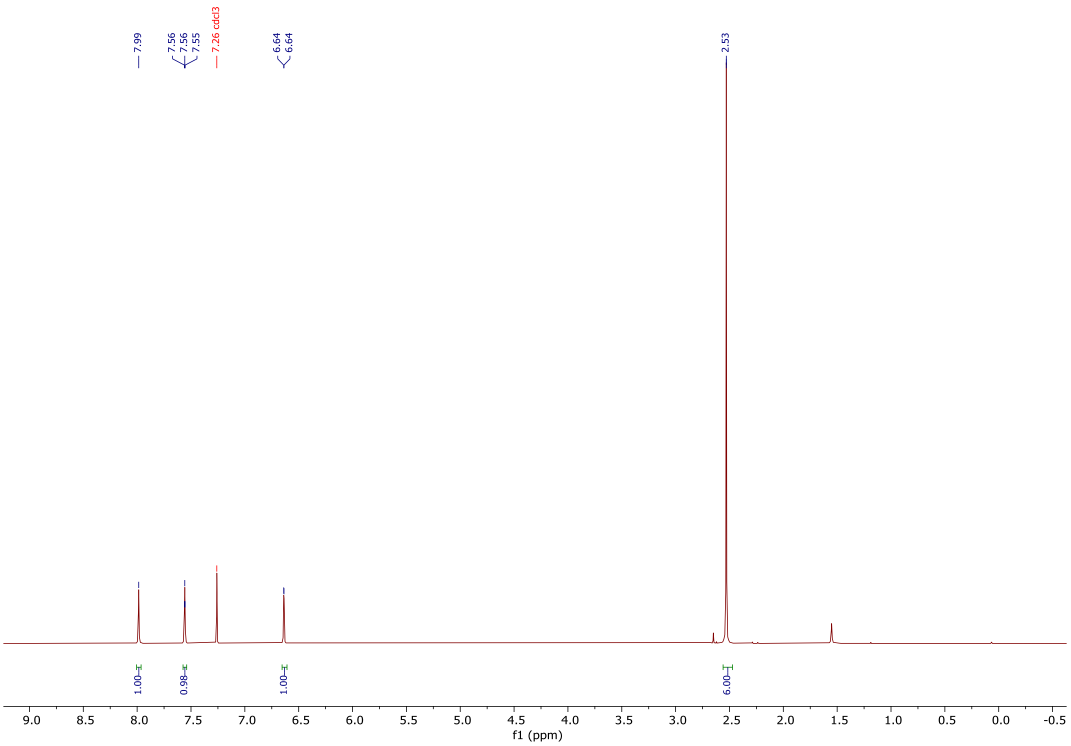

^{13}C NMR (126 MHz, $CDCl_3$)

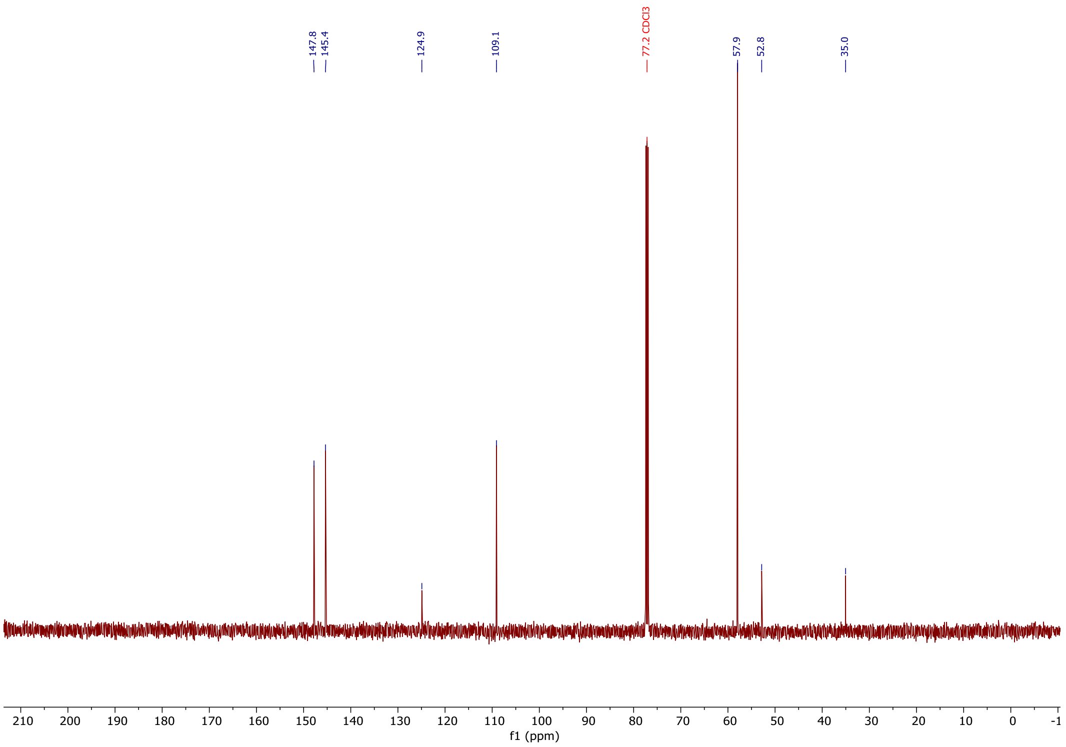

4-((3-Iodobicyclo[1.1.1]pentan-1-yl)sulfonyl)-3,5-dimethylisoxazole, 3v-I

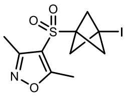

^{1}H NMR (600 MHz, $CDCl_3$)

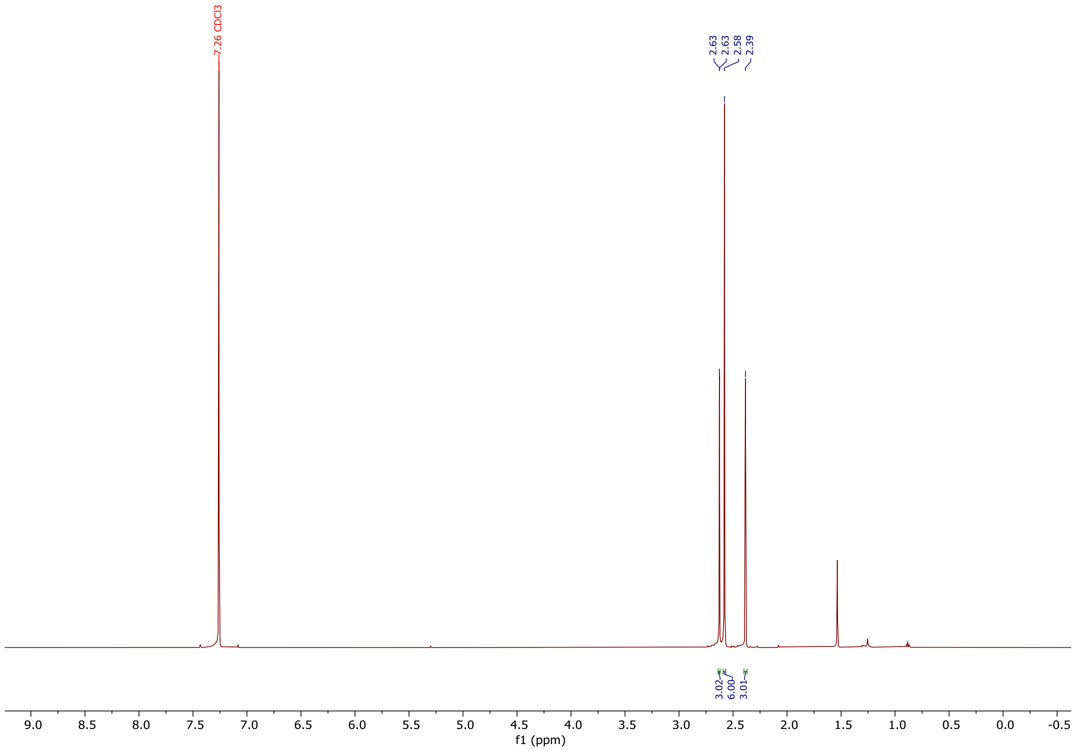

^{13}C NMR (151 MHz, $CDCl_3$)

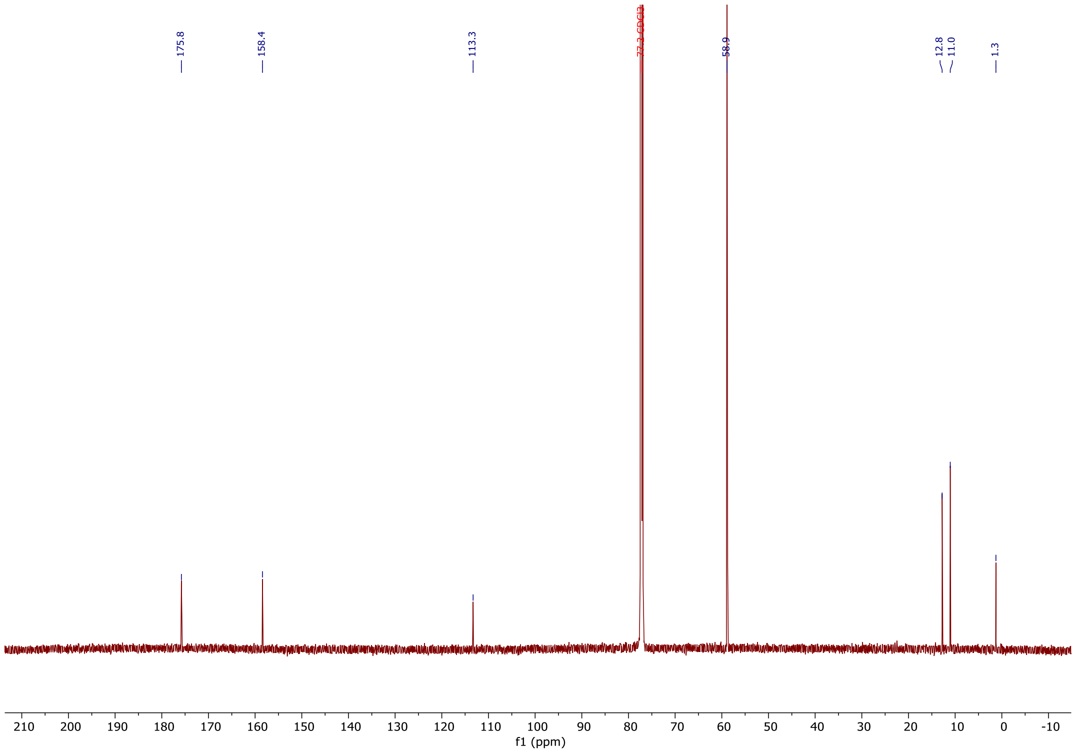

4-((3-Bromobicyclo[1.1.1]pentan-1-yl)sulfonyl)-3,5-dimethylisoxazole, 3v-Br

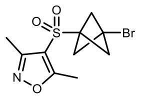

^{1}H NMR (400 MHz, $CDCl_3$)

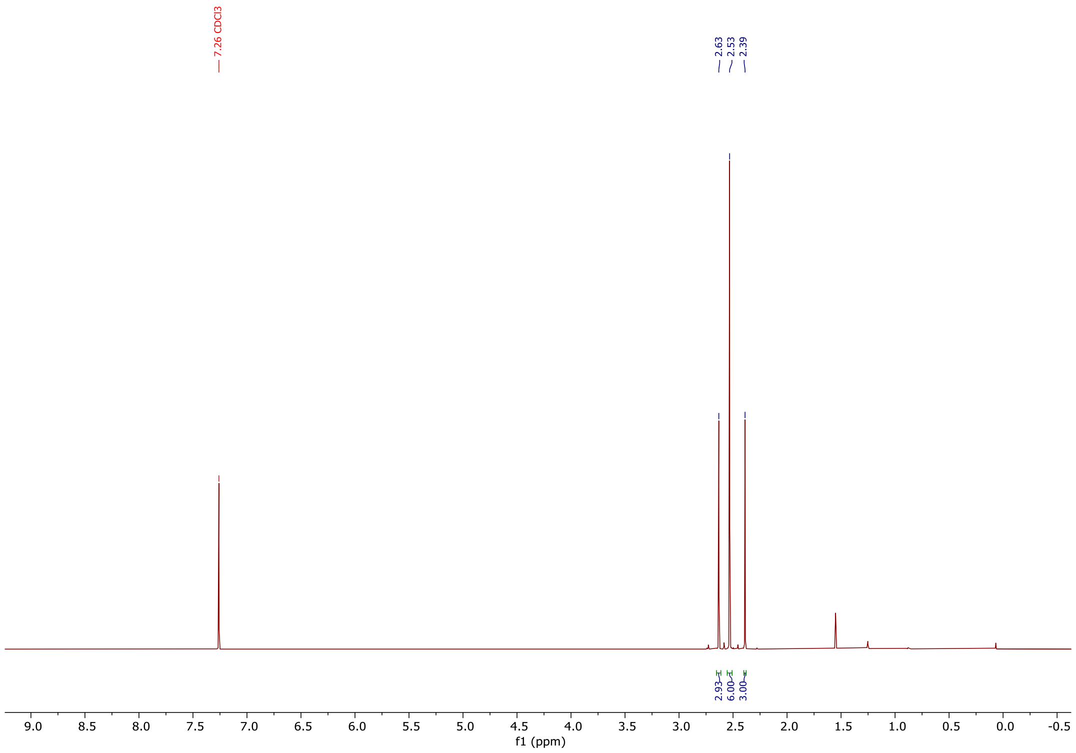

^{13}C NMR (151 MHz, $CDCl_3$)

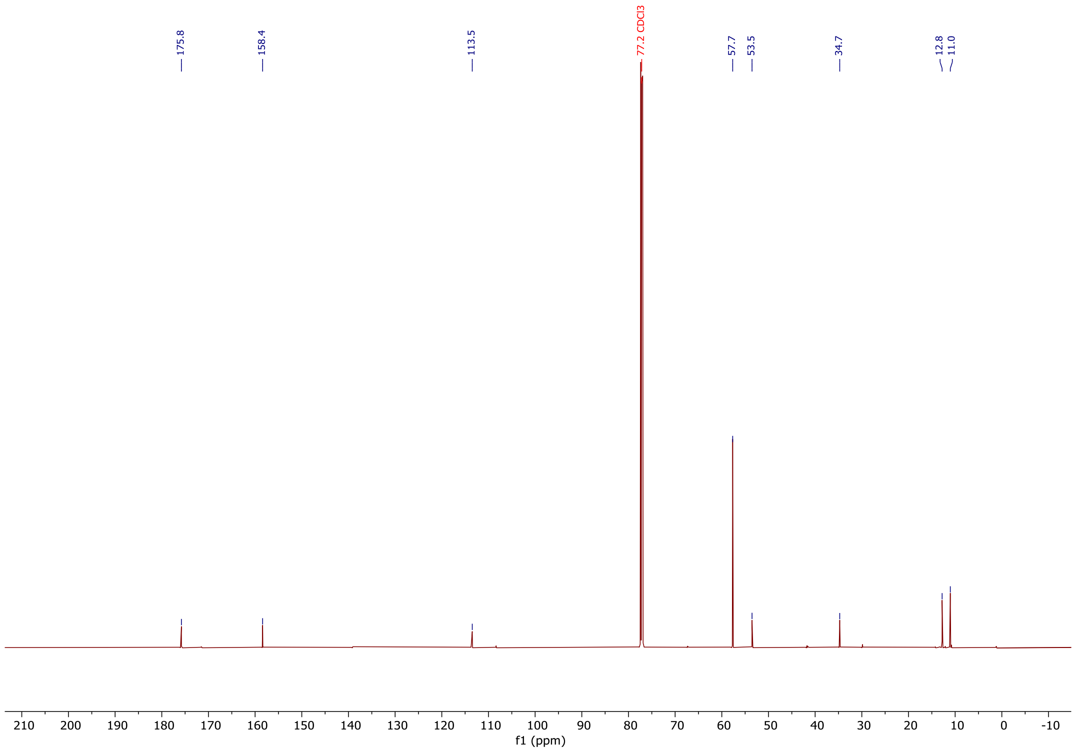

3-((3-Bromobicyclo[1.1.1]pentan-1-yl)sulfonyl)pyridine, 3w-Br

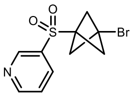

^1H NMR (600 MHz, $CDCl_3$)

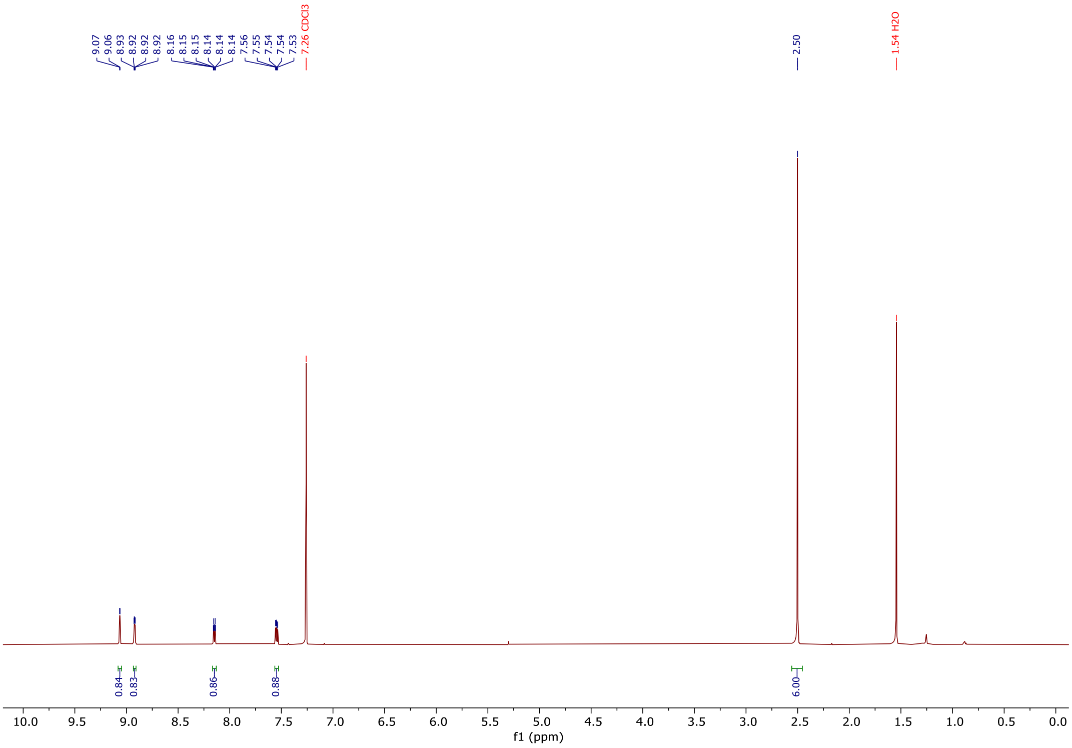

^{13}C NMR (151 MHz, $CDCl_3$)

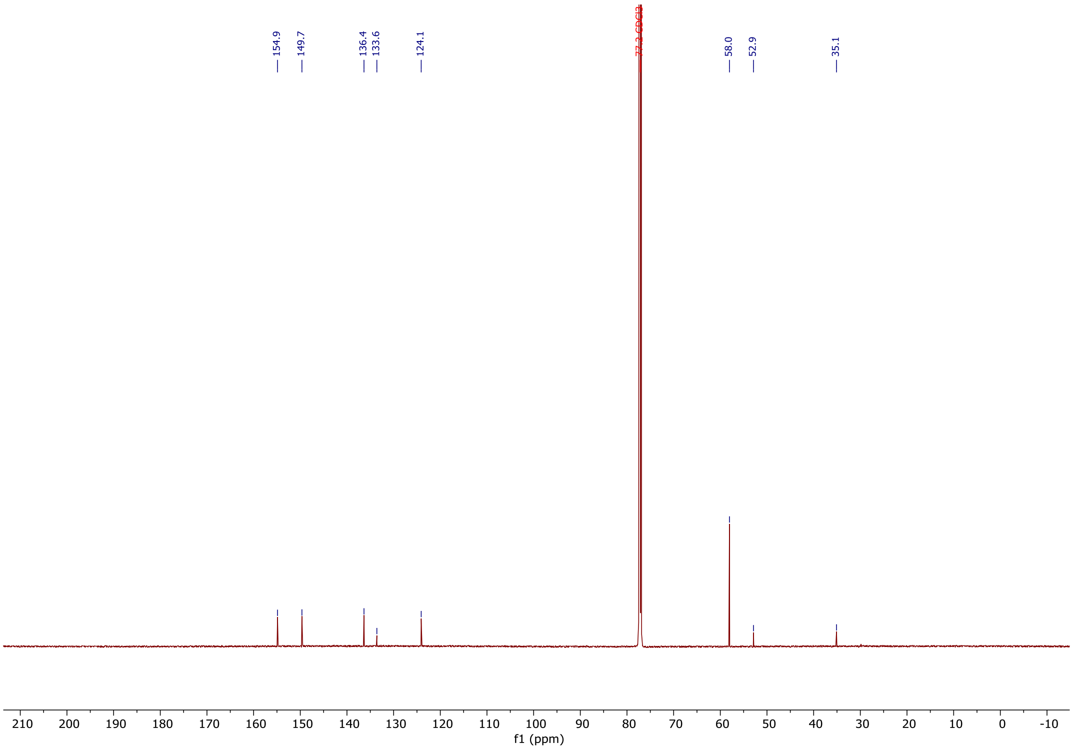

5-((3-Bromobicyclo[1.1.1]pentan-1-yl)sulfonyl)-2-methoxypyridine, 3x-Br

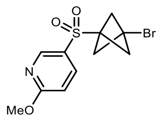

^{1}H NMR (500 MHz, DMSO-d$_6$)

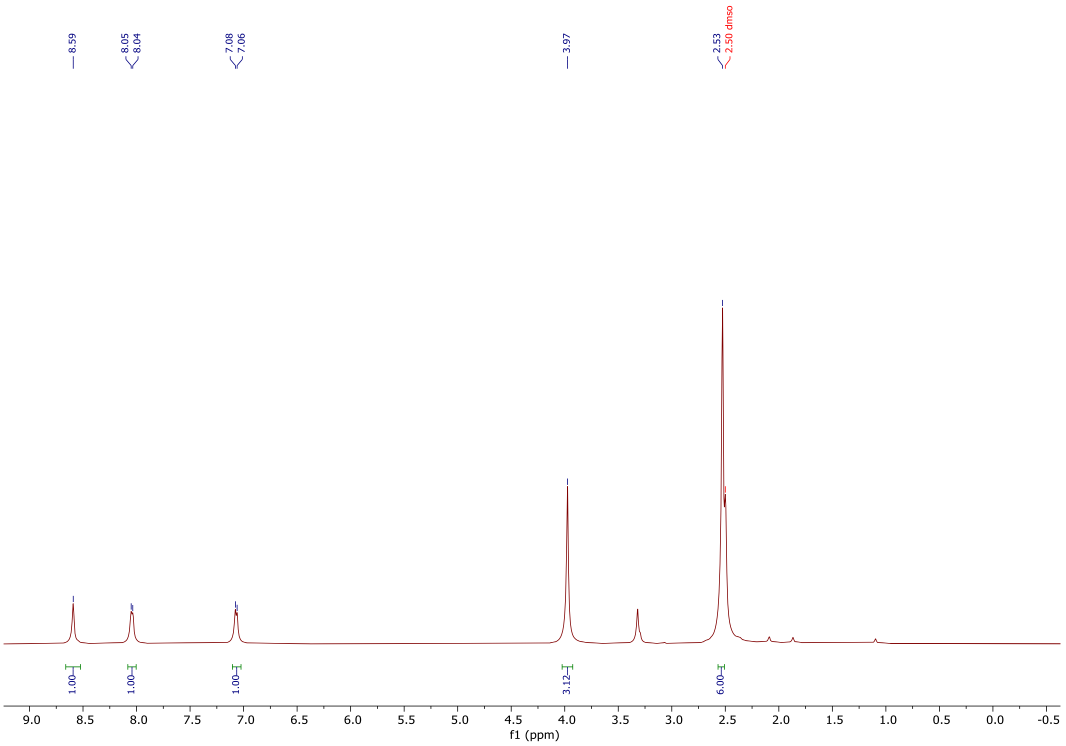

^{13}C NMR (126 MHz, DMSO-d$_6$)

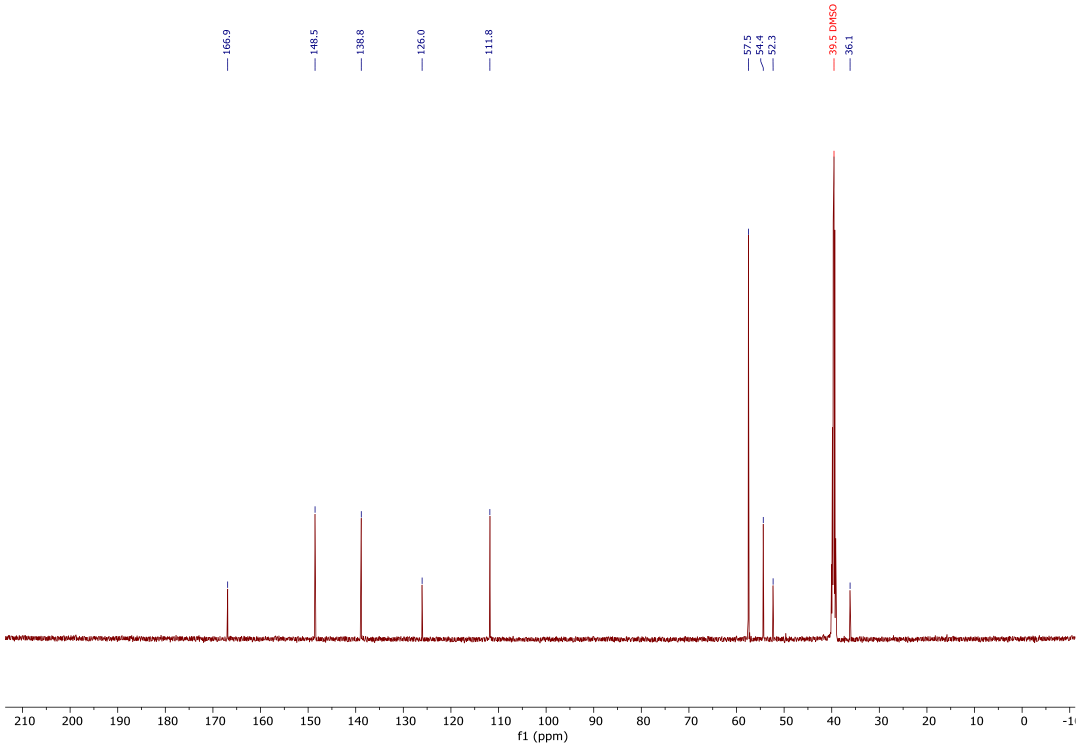

5-((3-Bromobicyclo[1.1.1]pentan-1-yl)sulfonyl)-2-chloropyridine, 3y-Br

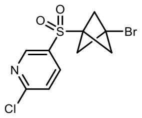

^{1}H NMR (500 MHz, $CDCl_3$)

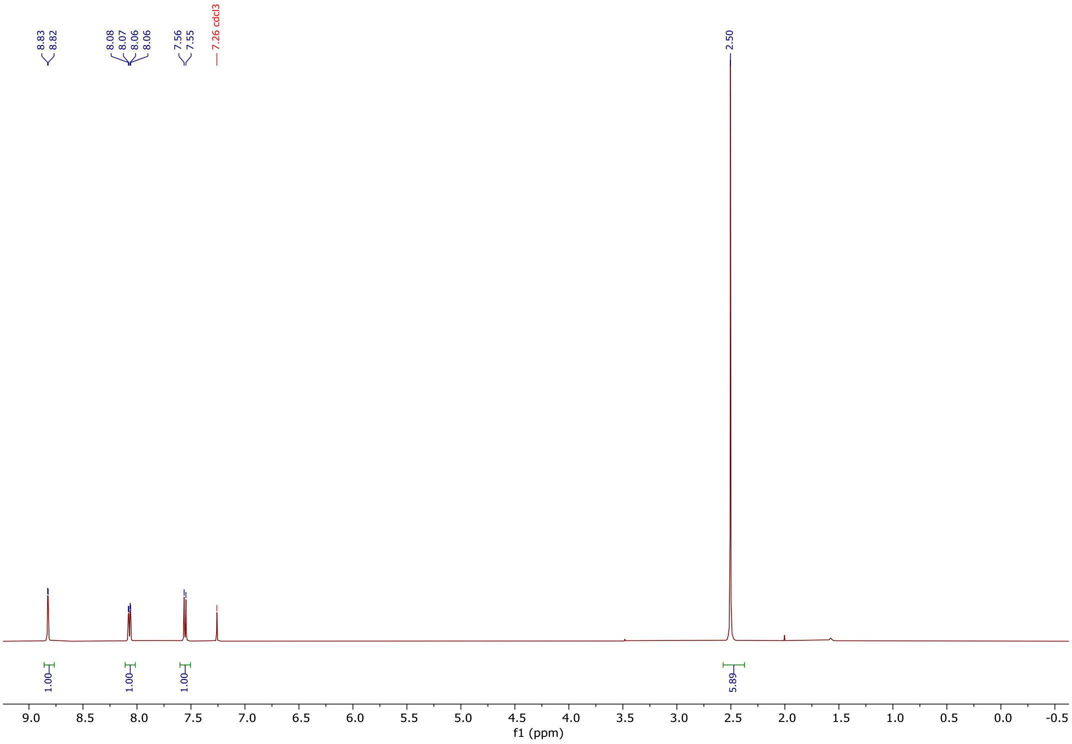

^{13}C NMR (151 MHz, $CDCl_3$)

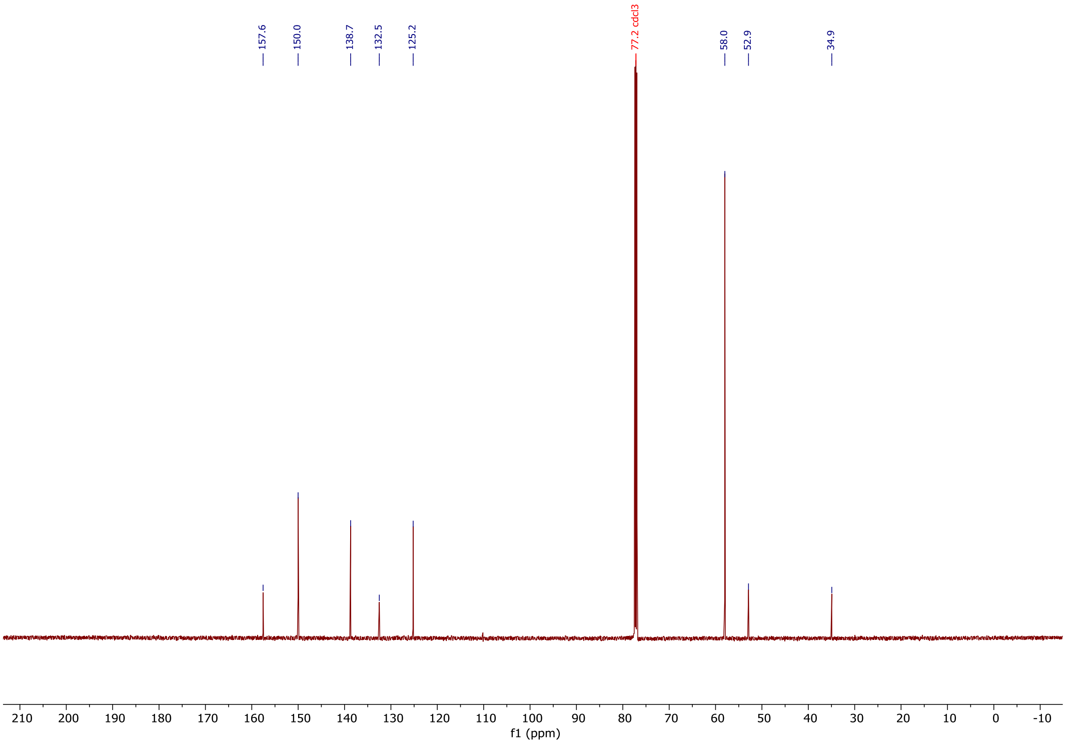

6.3 Pharmaceutical and Agrochemical BCP Halides

5-(2-Ethoxy-5-((3-iodobicyclo[1.1.1]pentan-1-yl)sulfonyl)phenyl)-1-methyl-3-propyl-1,6-dihydro–7H-pyrazolo[4,3-d]pyrimidin–7-one, 3z-I

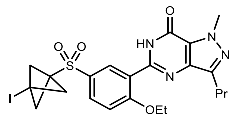

^{1}**H NMR** (600 MHz, $CDCl_3$)

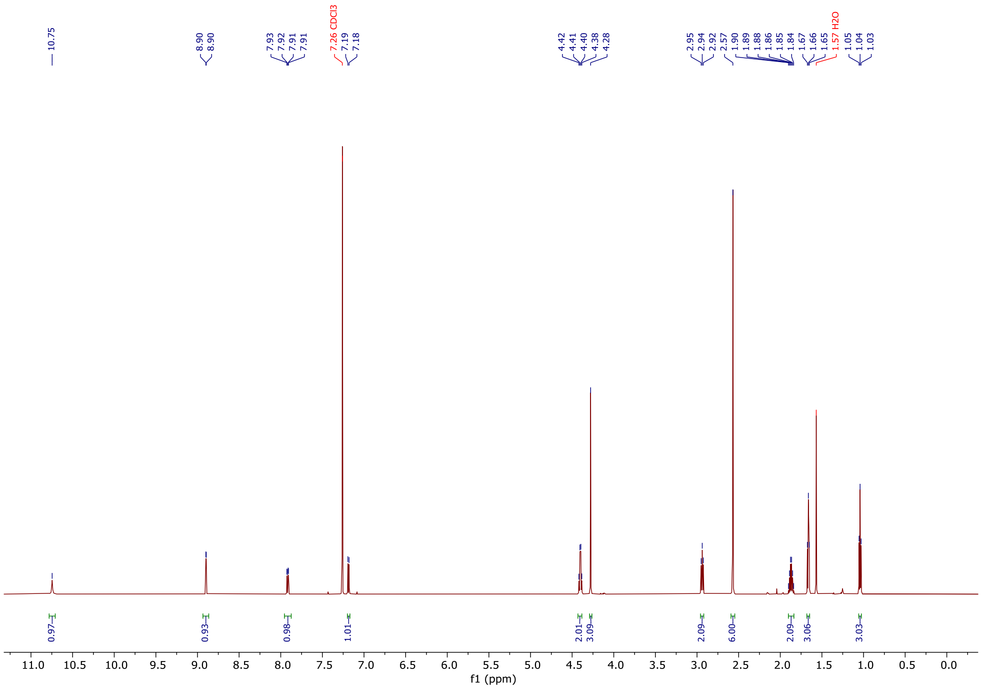

^{13}**C NMR** (151 MHz, $CDCl_3$)

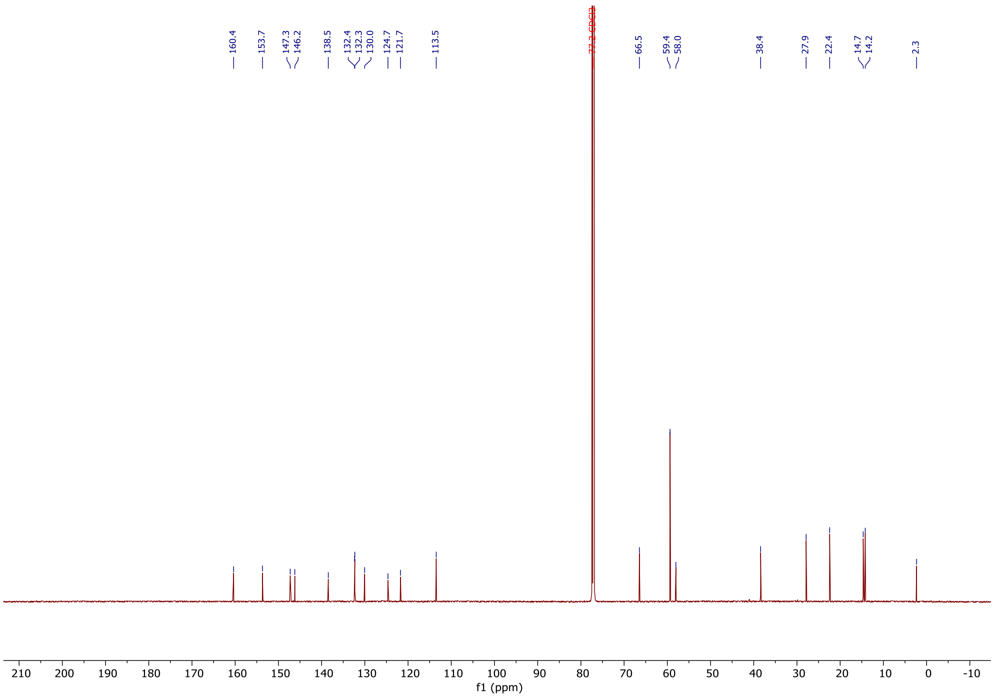

5-(2-Ethoxy-5-((3-bromoobicyclo[1.1.1]pentan-1-yl)sulfonyl)phenyl)-1-methyl-3-propyl-1,6-dihydro–7H-pyrazolo[4,3-d]pyrimidin–7-one, 3z-Br

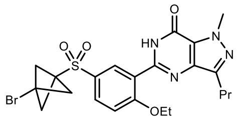

^{1}**H NMR** (400 MHz, $CDCl_3$)

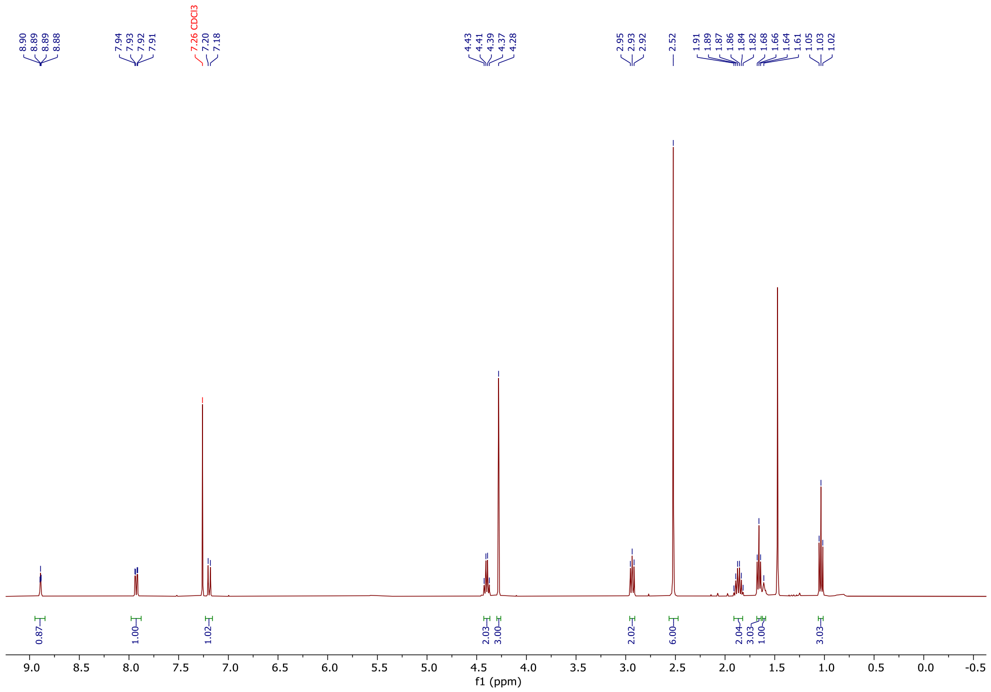

^{13}**C NMR** (151 MHz, $CDCl_3$)

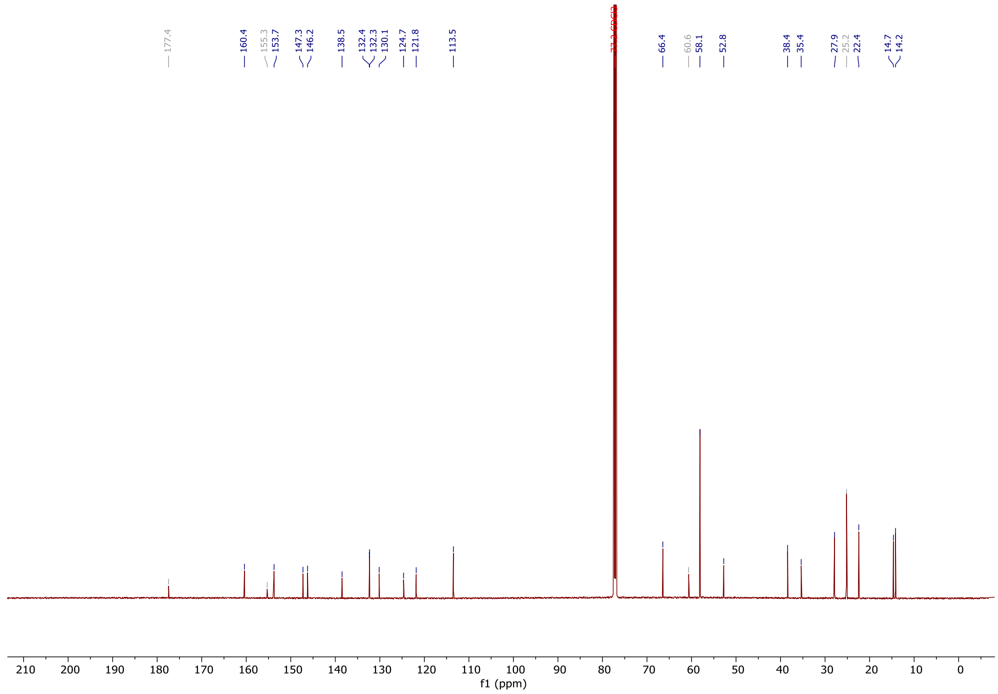

Note: ^{13}C NMR contains small amounts of dimethylhydantoin

3-((3-Iodobicyclo[1.1.1]pentan-1-yl)sulfonyl)-*N*,*N*-diethyl-4H-1,2,4-triazole-4-carboxamide, 3aa-I

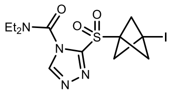

^{1}H NMR (600 MHz, CDCl$_3$)

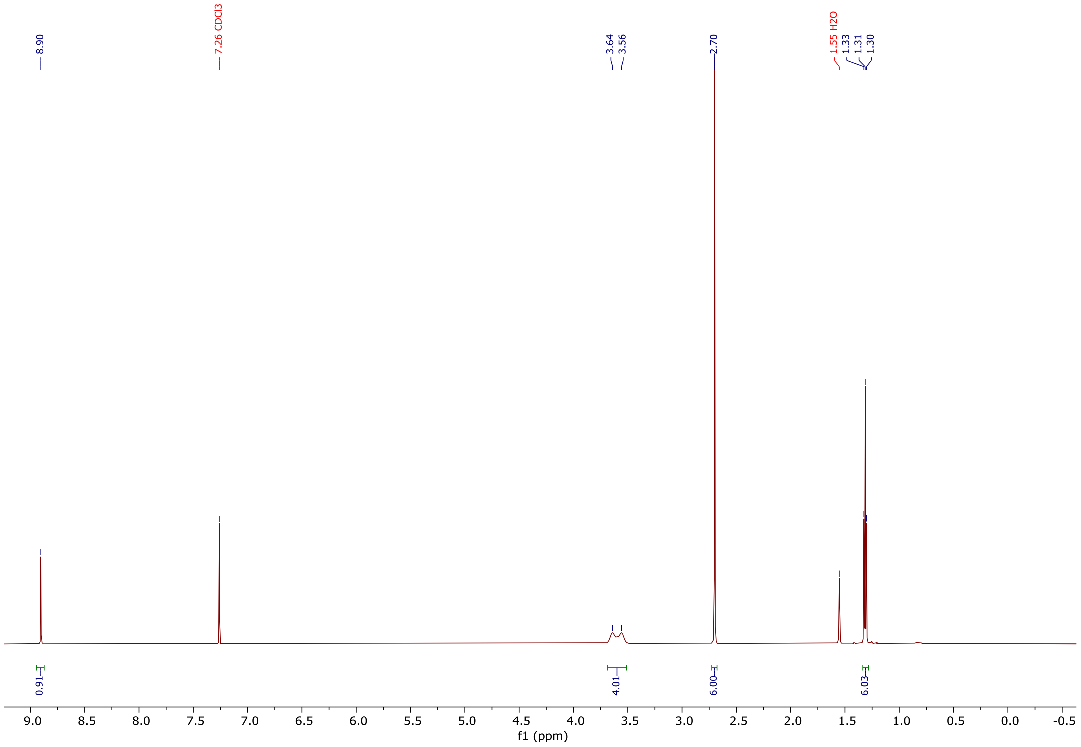

^{13}C NMR (151 MHz, CDCl$_3$)

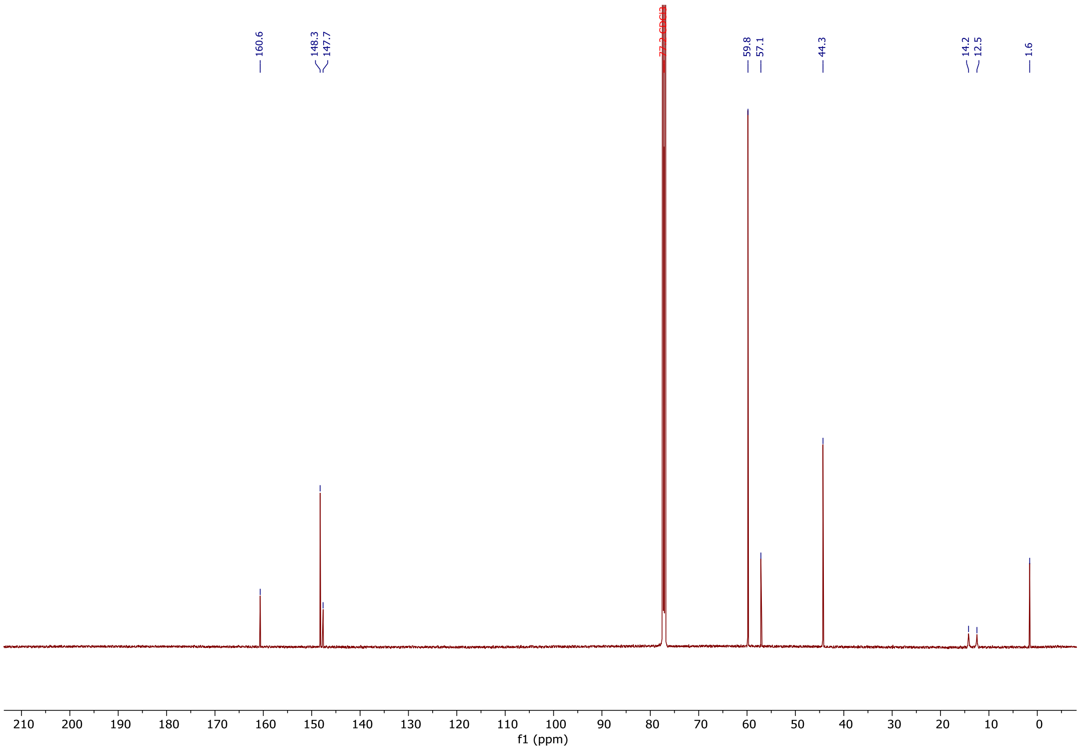

3-((3-Bromobicyclo[1.1.1]pentan-1-yl)sulfonyl)-*N*,*N*-diethyl-4H-1,2,4-triazole-4-carboxamide, 3aa-Br

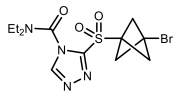

^{1}H NMR (600 MHz, $CDCl_3$)

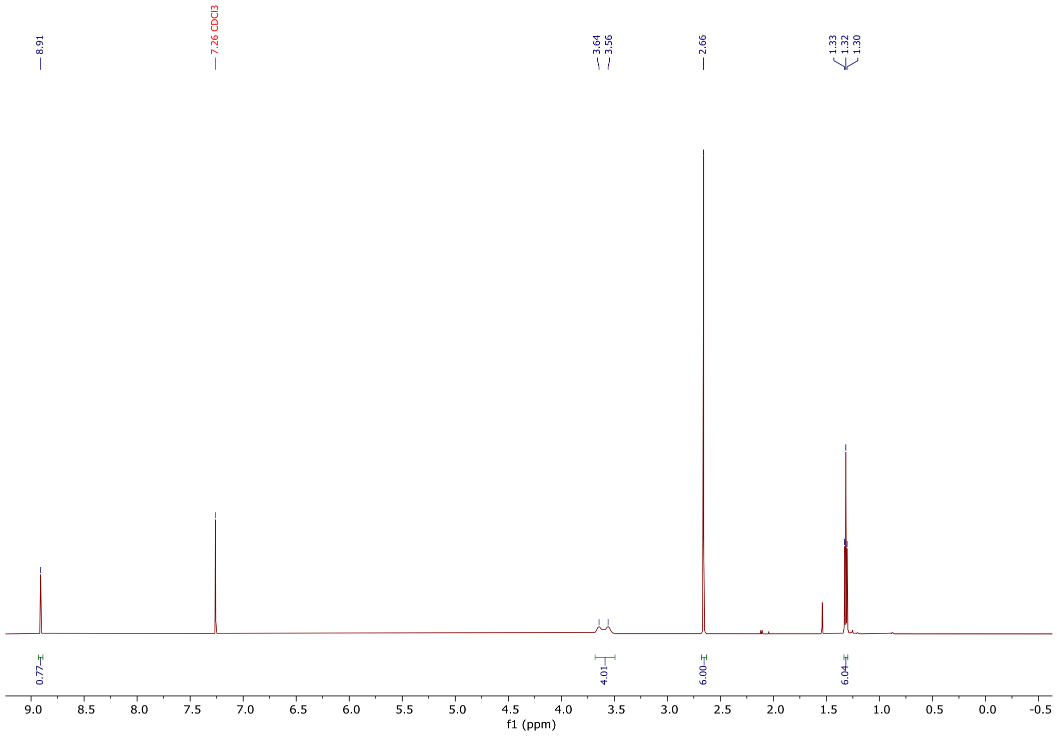

^{13}C NMR (151 MHz, $CDCl_3$)

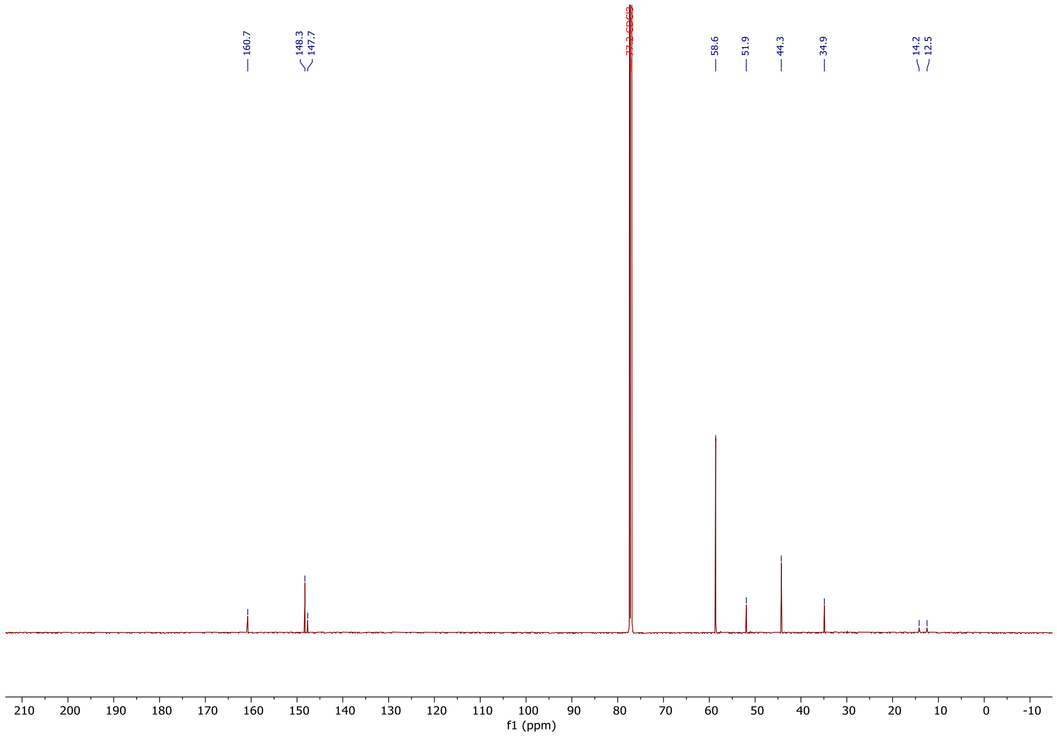

6.4 Alkyl Sulfonyl BCP Halides

1-Iodo-3-(methylsulfonyl)bicyclo[1.1.1]pentane, 7a-I

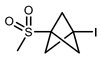

^{1}H NMR (400 MHz, $CDCl_3$)

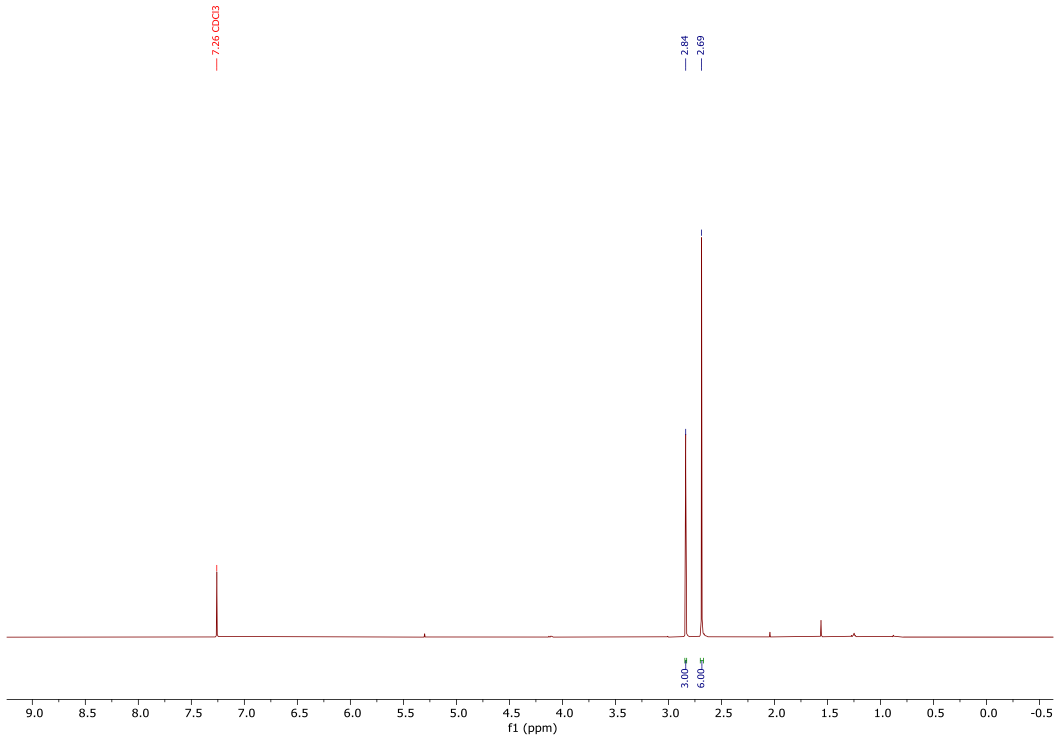

^{13}C NMR (101 MHz, $CDCl_3$)

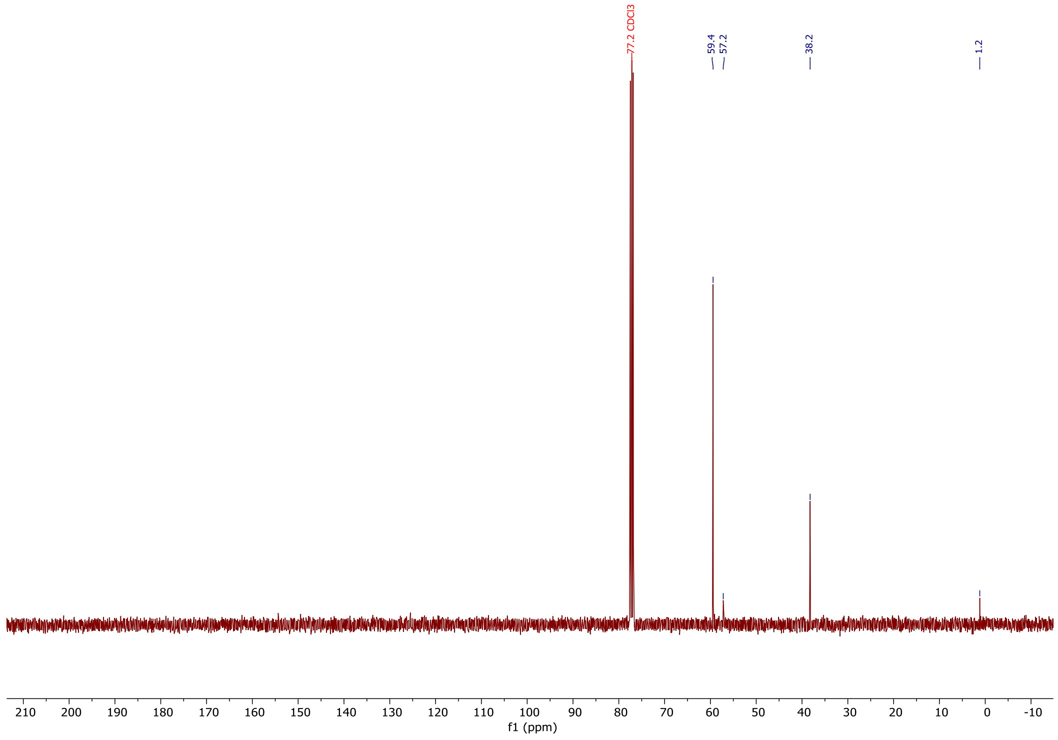

1-Bromo-3-(methylsulfonyl)bicyclo[1.1.1]pentane, 7a-Br

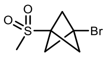

^{1}H NMR (400 MHz, $CDCl_3$)

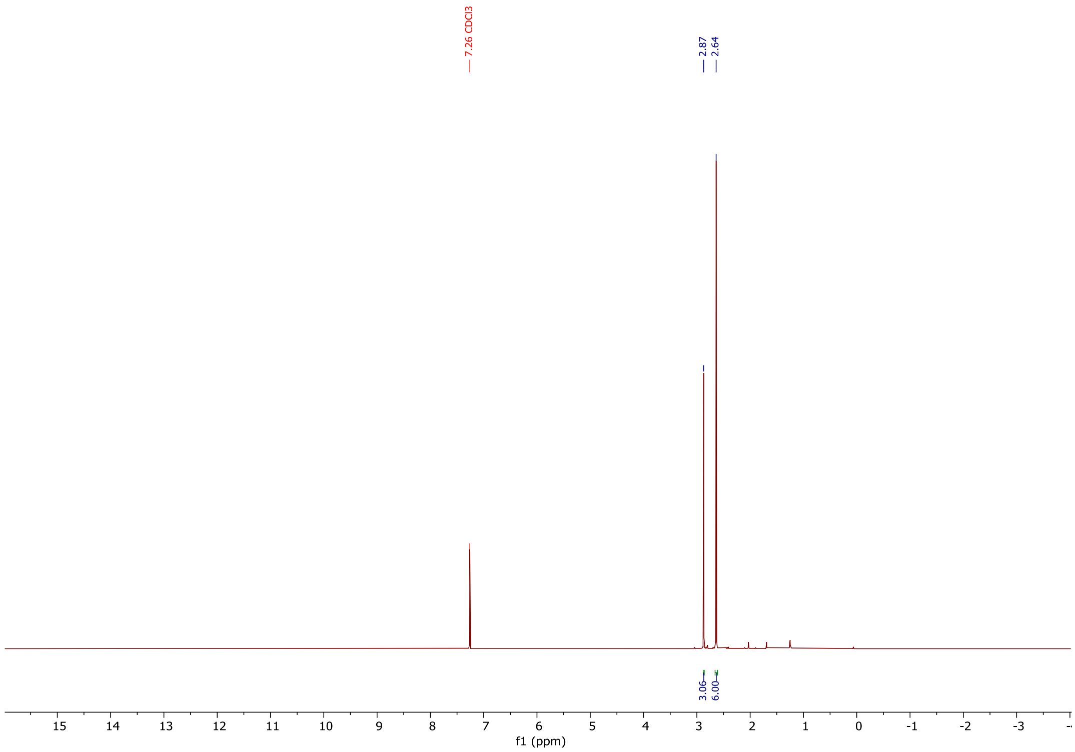

^{13}C NMR (101 MHz, $CDCl_3$)

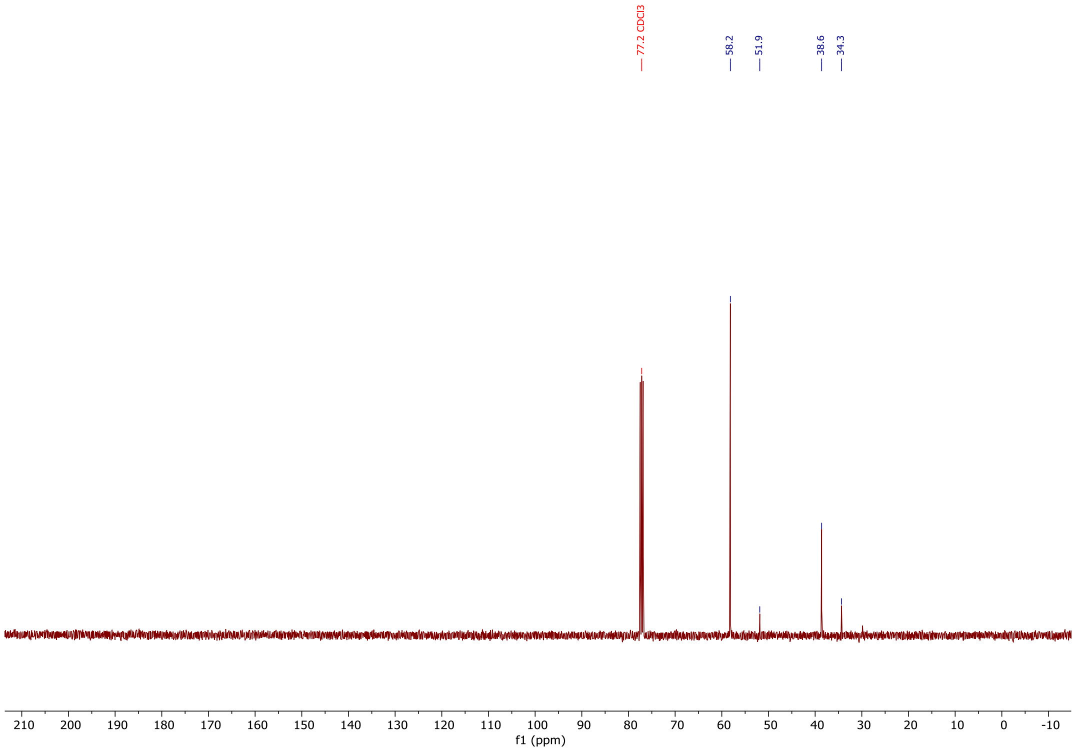

1-Bromo-3-(ethylsulfonyl)bicyclo[1.1.1]pentane, 7b-Br

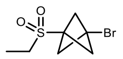

^{1}H NMR (500 MHz, CDCl$_3$)

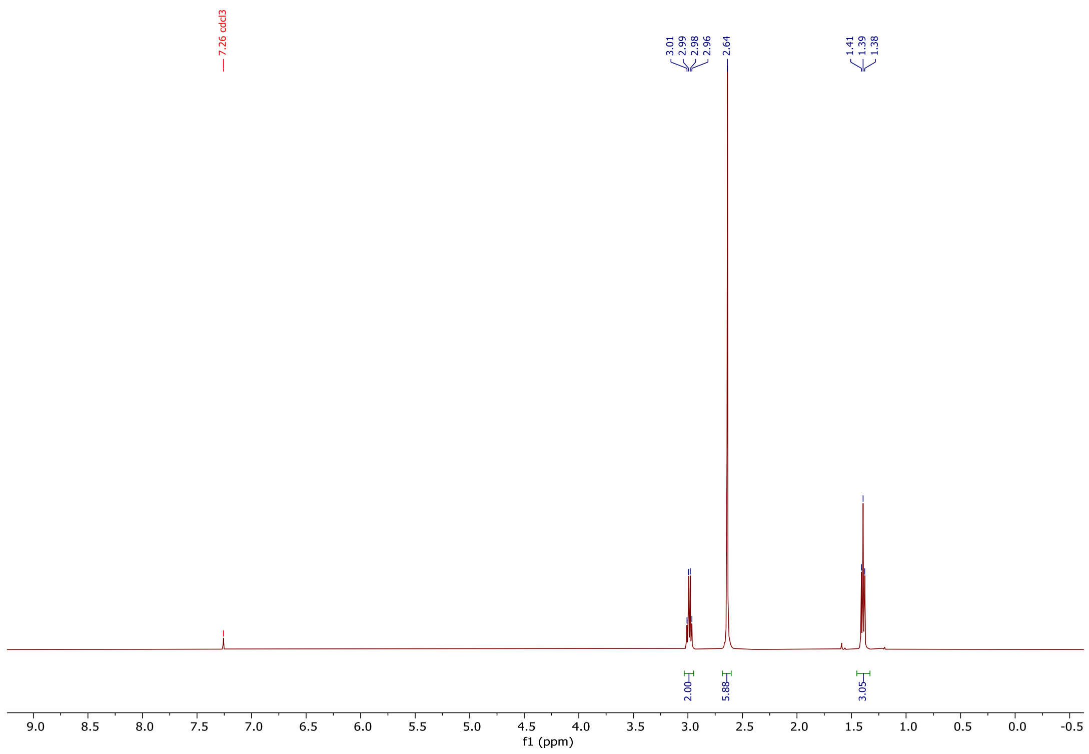

^{13}C NMR (126 MHz, CDCl$_3$)

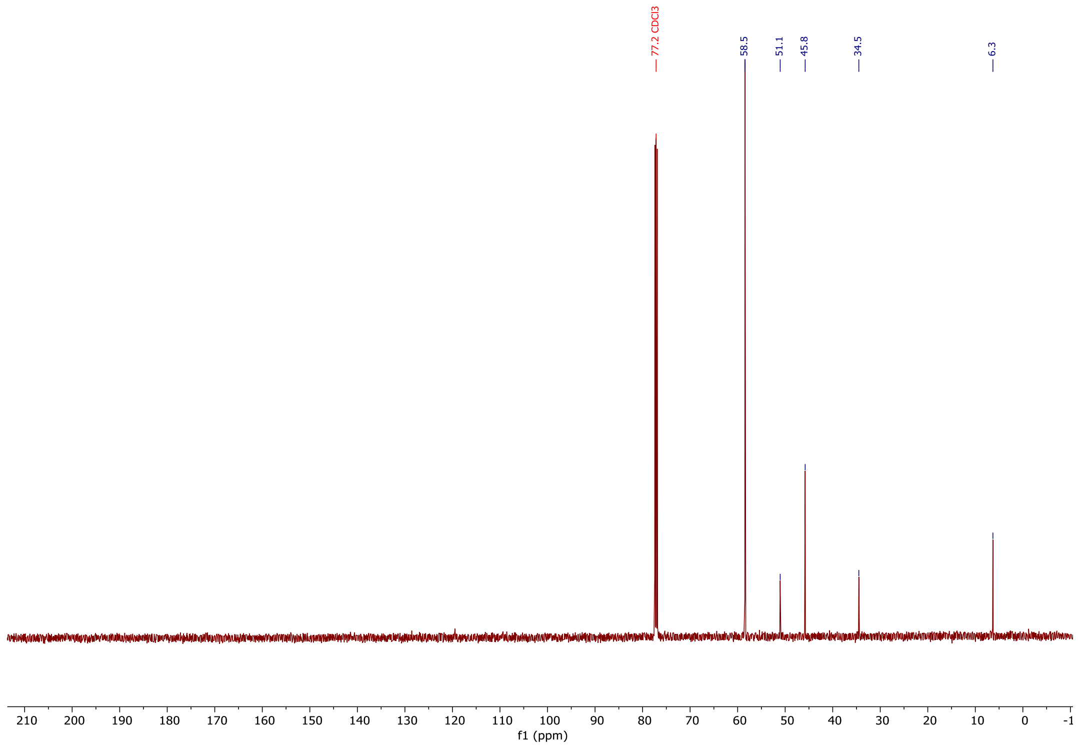

1-(Butylsulfonyl)-3-iodobicyclo[1.1.1]pentane, 7c-I

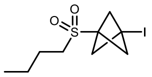

^{1}H NMR (400 MHz, $CDCl_3$)

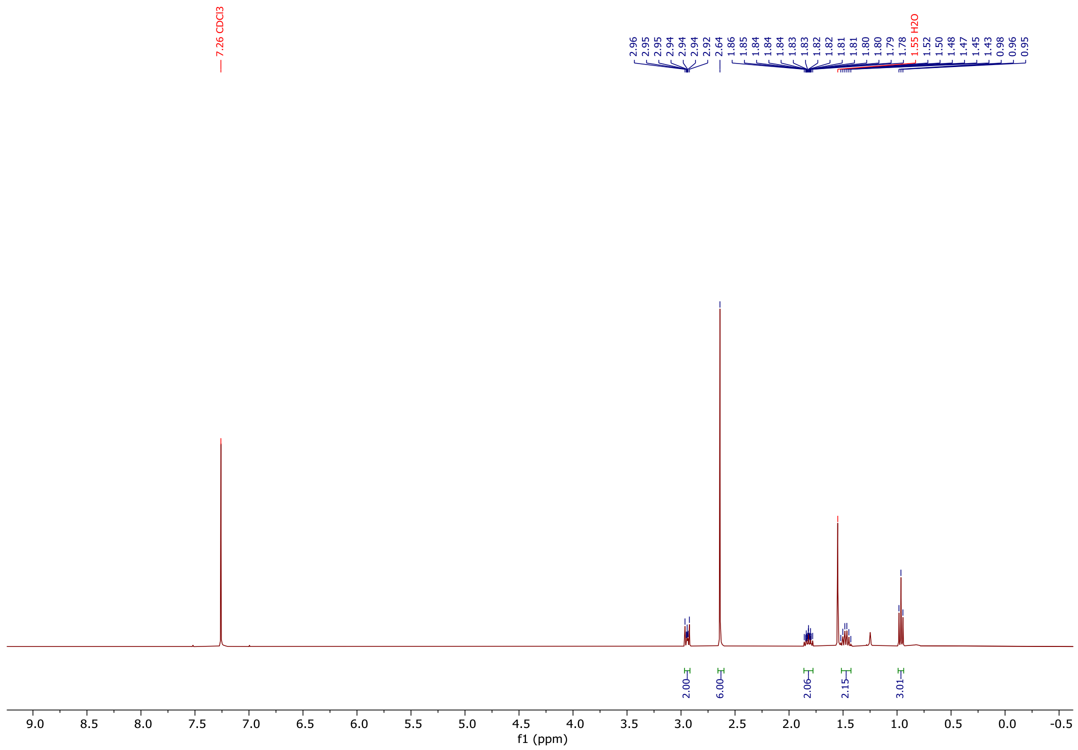

^{13}C NMR (151 MHz, $CDCl_3$)

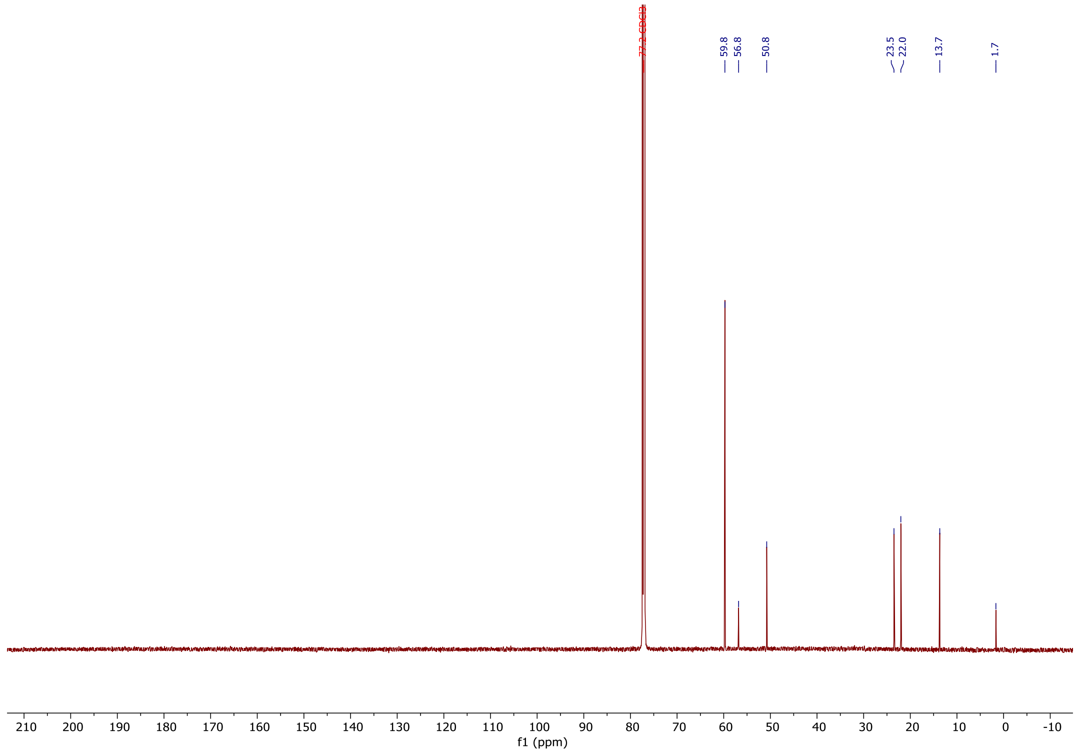

1-Bromo-3-(butylsulfonyl)bicyclo[1.1.1]pentane, 7c-Br

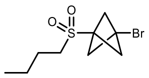

^{1}H NMR (600 MHz, $CDCl_3$)

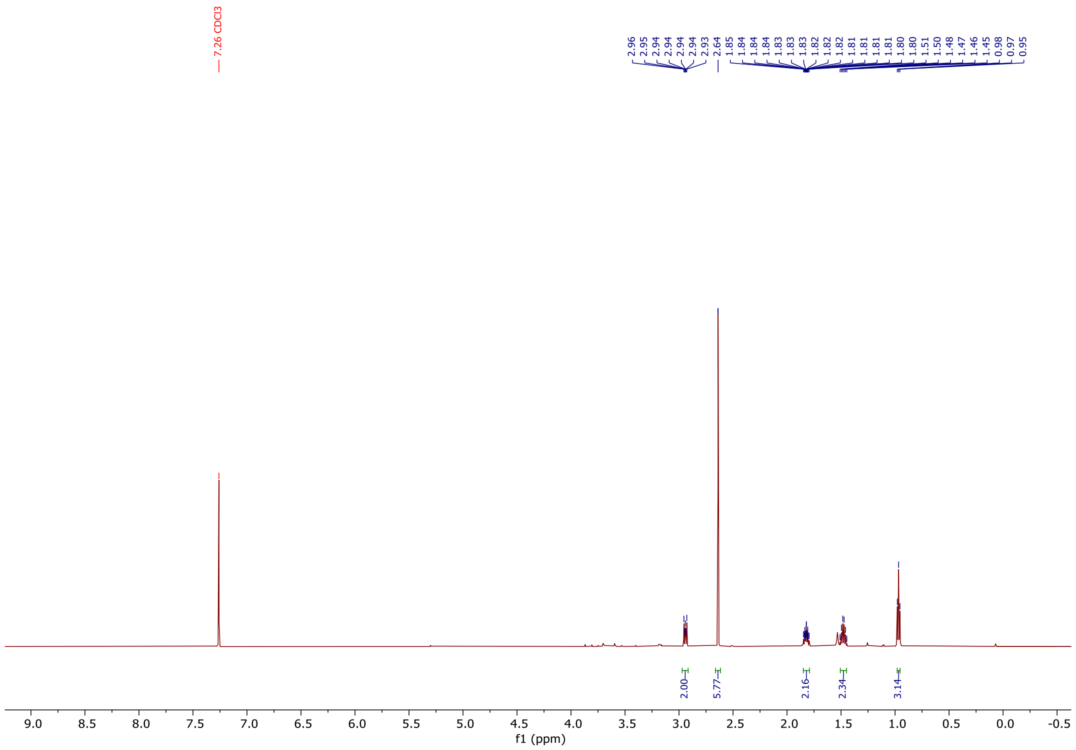

^{13}C NMR (151 MHz, $CDCl_3$)

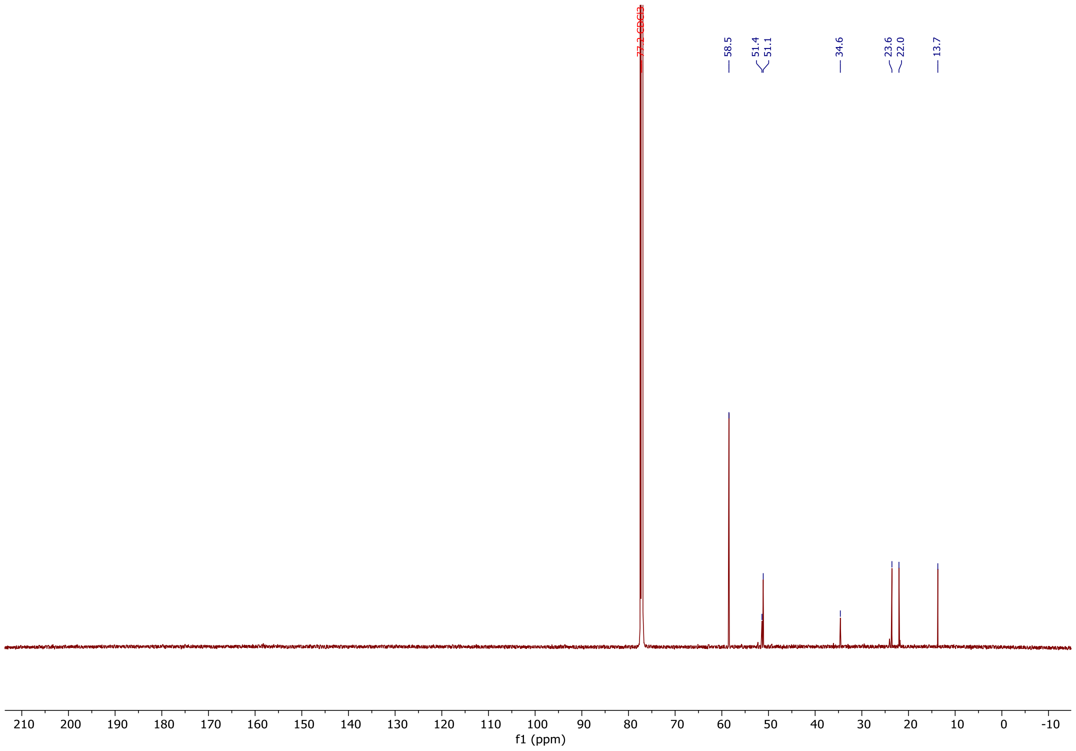

1-Iodo-3-(phenethylsulfonyl)bicyclo[1.1.1]pentane, 7d-I

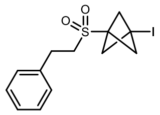

^{1}H NMR (600 MHz, $CDCl_3$)

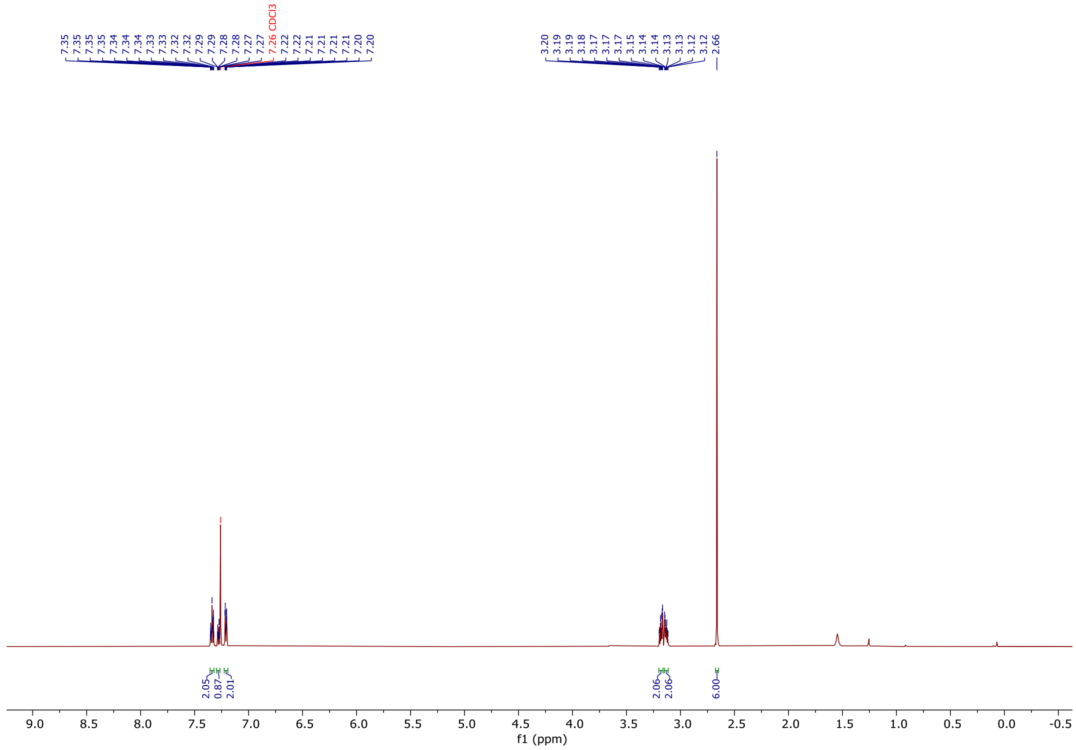

^{13}C NMR (151 MHz, $CDCl_3$)

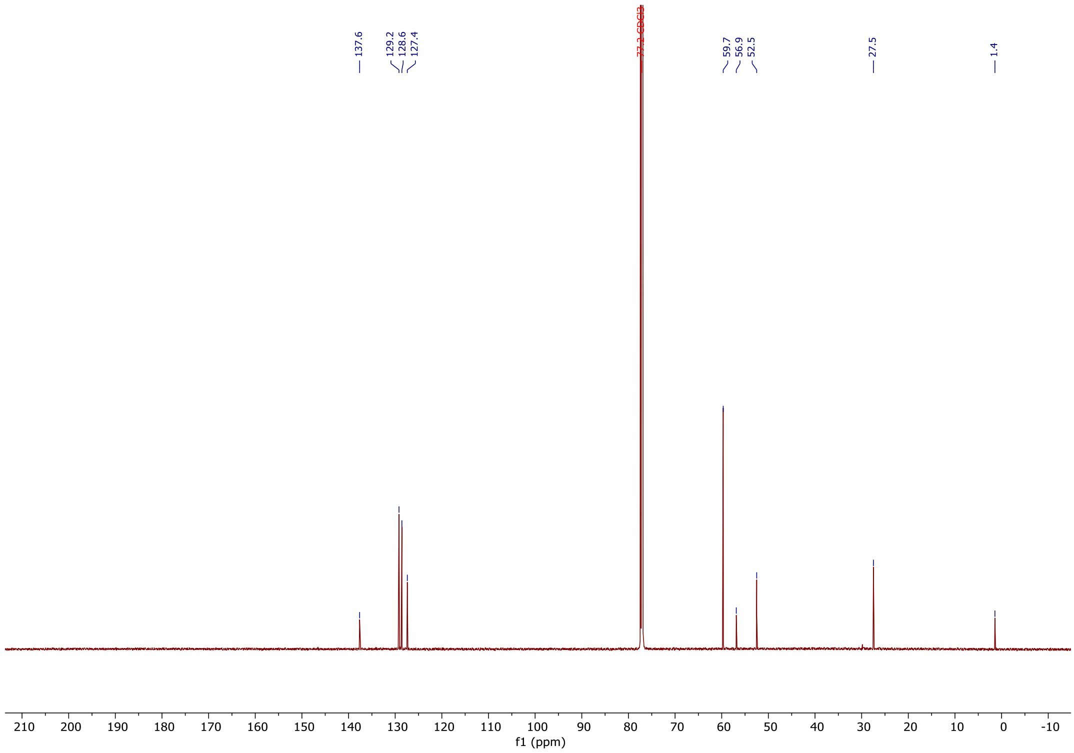

1-Iodo-3-(isopropylsulfonyl)bicyclo[1.1.1]pentane, 7e-I

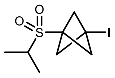

^{1}H NMR (600 MHz, $CDCl_3$)

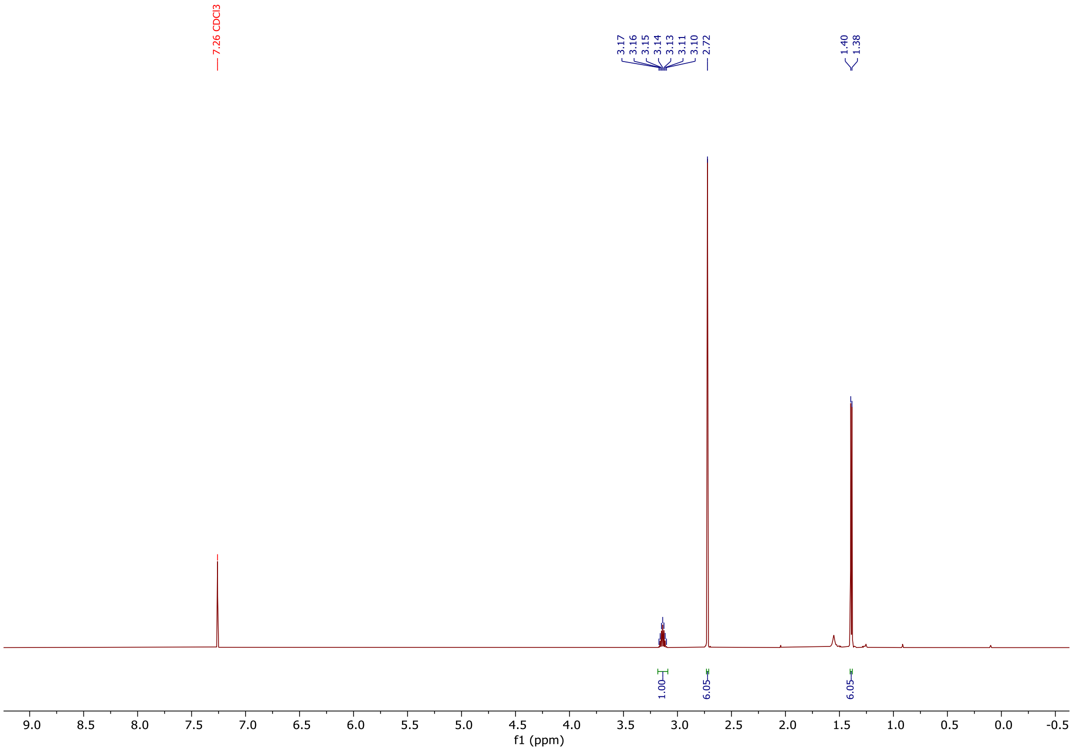

^{13}C NMR (151 MHz, $CDCl_3$)

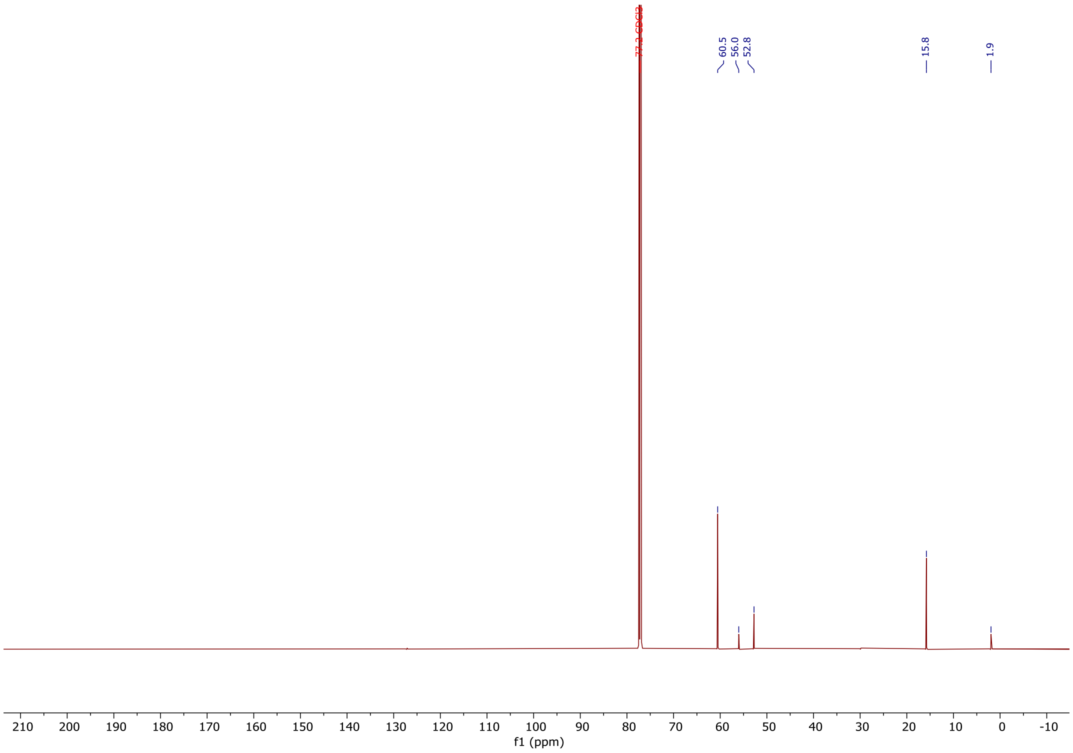

1-Bromo-3-(isopropylsulfonyl)bicyclo[1.1.1]pentane, 7e-Br

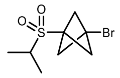

^{1}H NMR (600 MHz, CDCl$_3$)

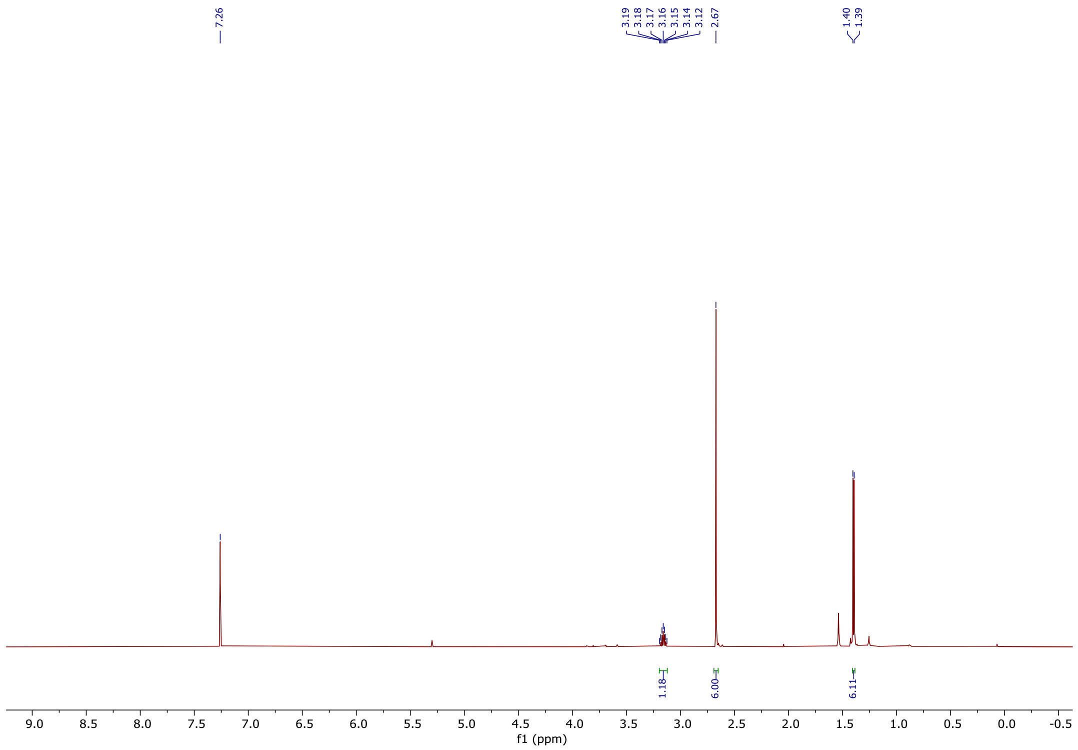

^{13}C NMR (151 MHz, CDCl$_3$)

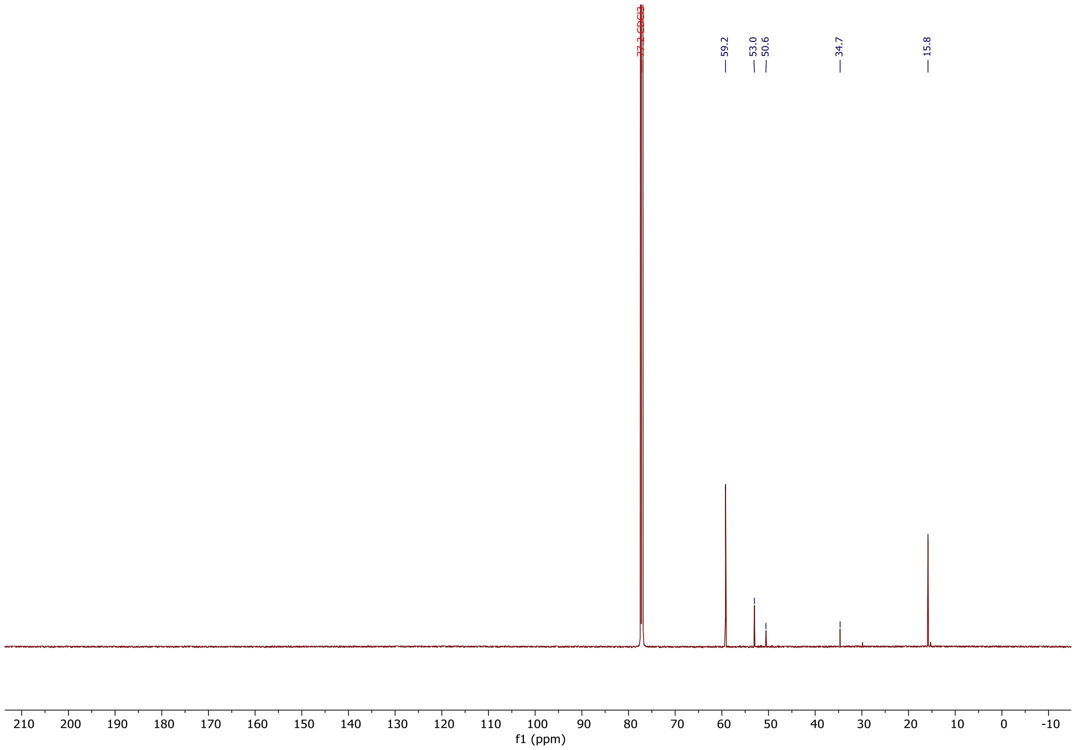

1-(Cyclopropylsulfonyl)-3-iodobicyclo[1.1.1]pentane, 7f-I

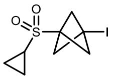

^{1}H NMR (600 MHz, $CDCl_3$)

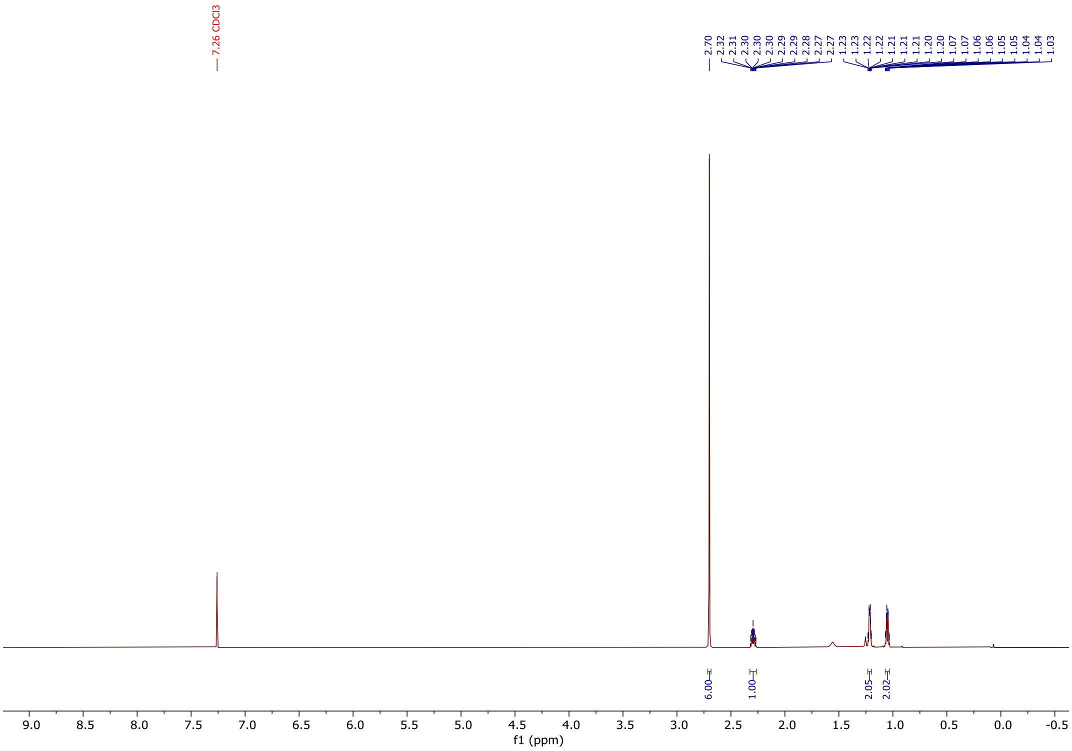

^{13}C NMR (151 MHz, $CDCl_3$)

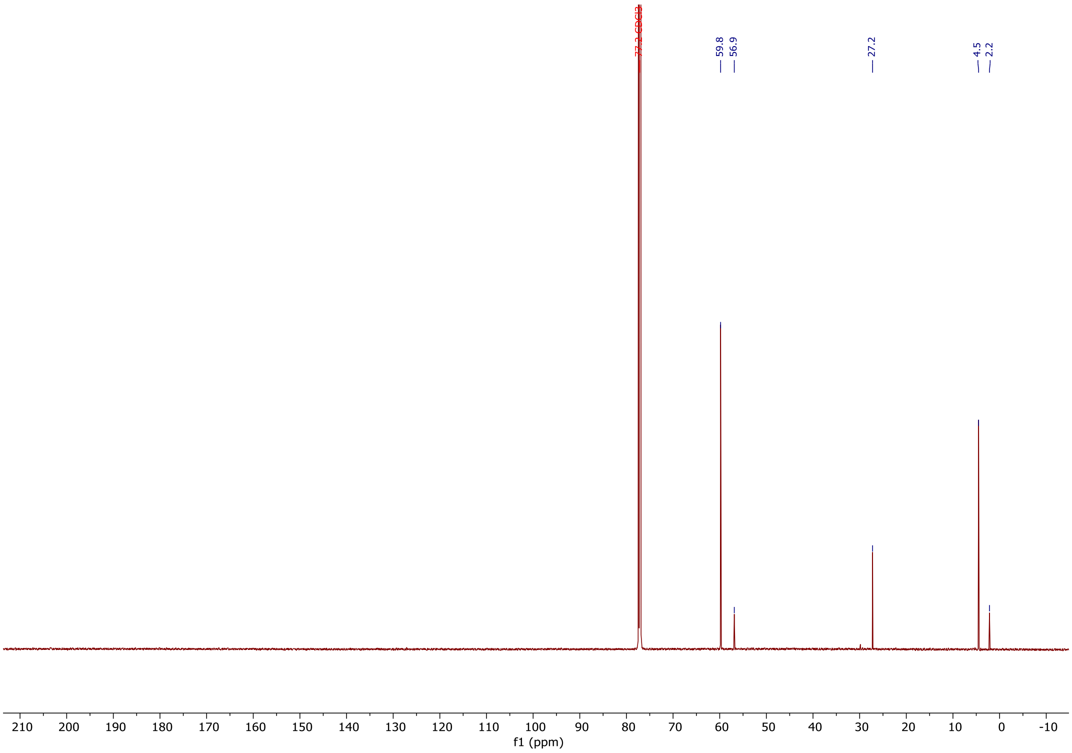

1-Bromo-3-(cyclopropylsulfonyl)bicyclo[1.1.1]pentane, 7f-Br

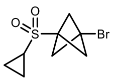

^{1}H **NMR** (600 MHz, $CDCl_3$)

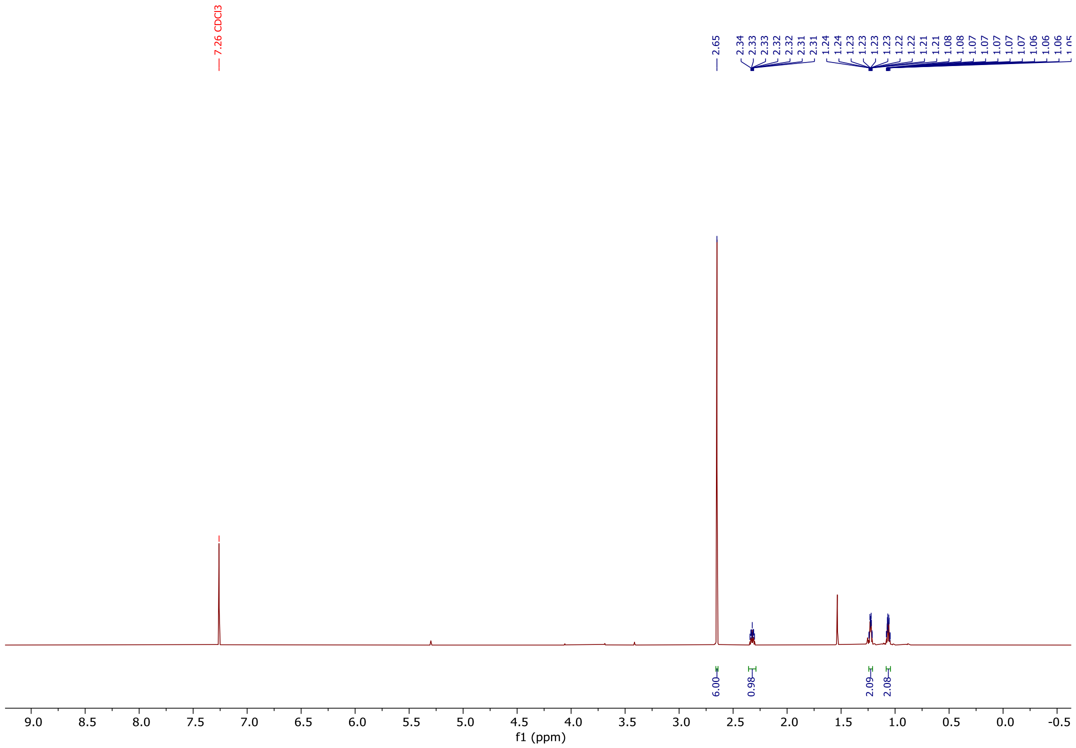

^{13}C **NMR** (151 MHz, $CDCl_3$)

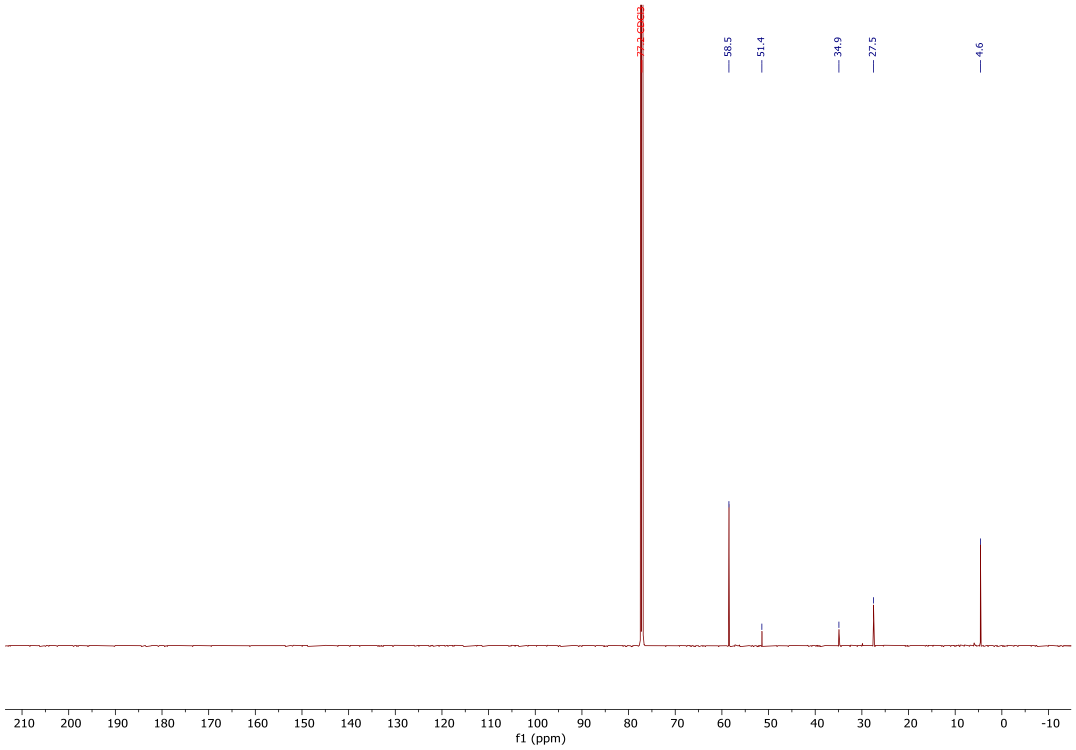

Methyl 3-((3-iodoobicyclo[1.1.1]pentan-1-yl)sulfonyl)propanoate, 7g-I

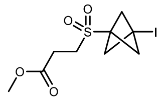

^{1}H NMR (400 MHz, $CDCl_3$)

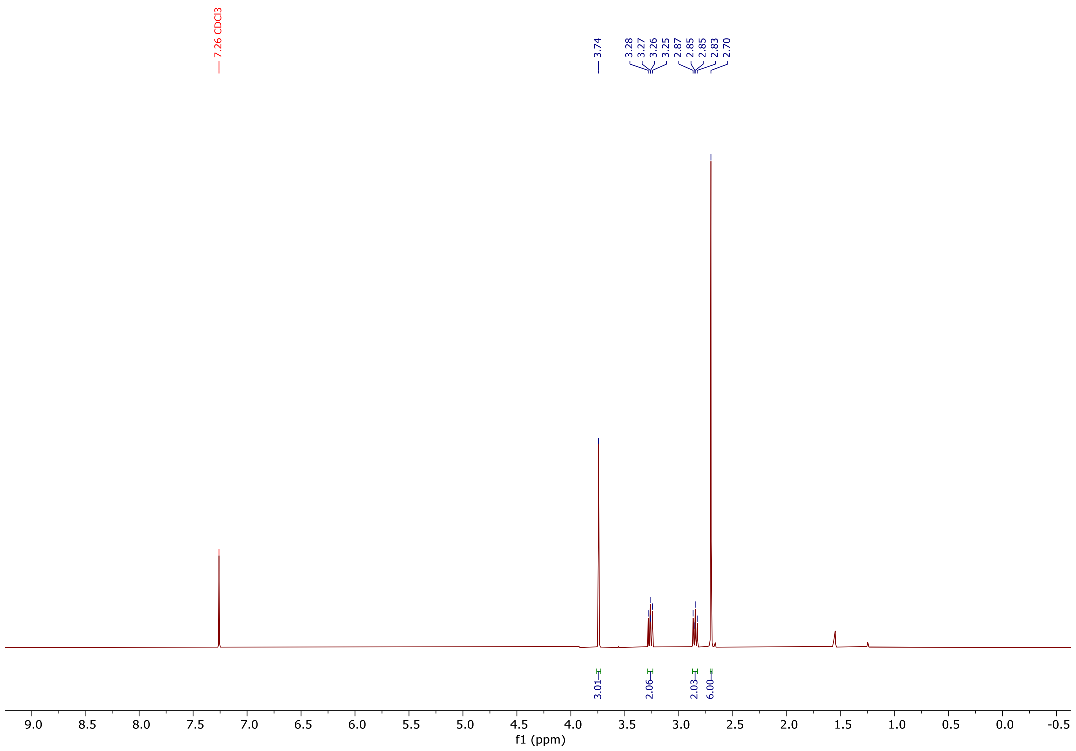

^{13}C NMR (101 MHz, $CDCl_3$)

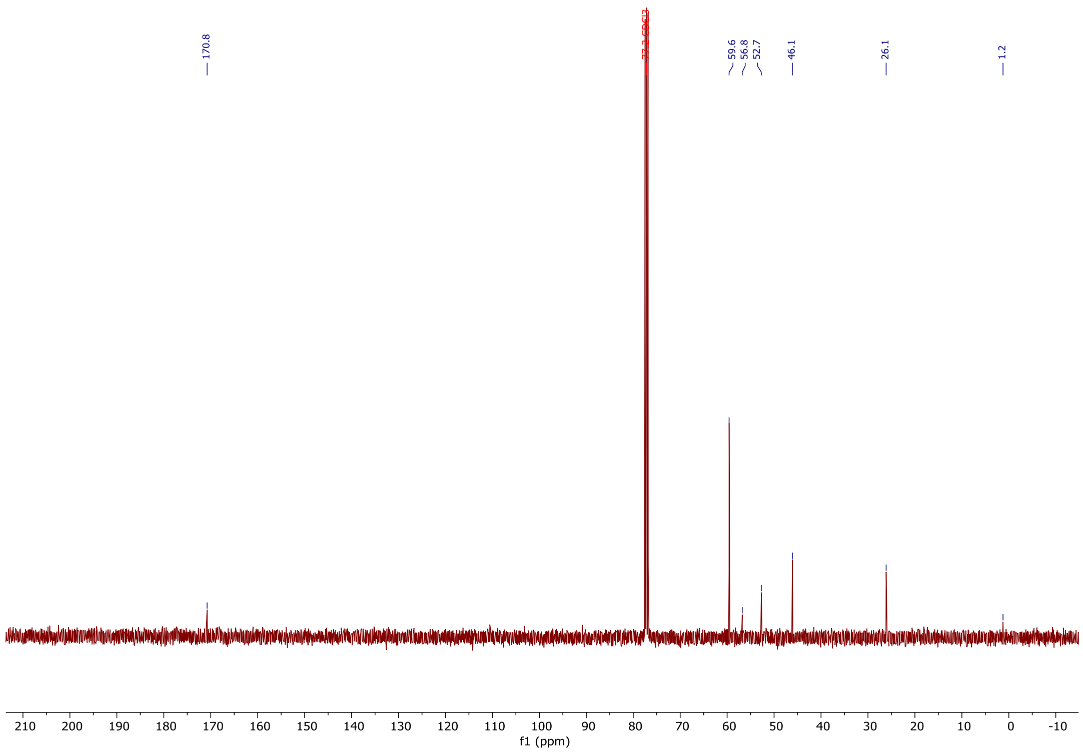

Methyl 3-((3-bromobicyclo[1.1.1]pentan-1-yl)sulfonyl)propanoate, 7g-Br

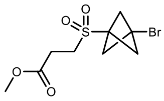

^{1}H NMR (600 MHz, $CDCl_3$)

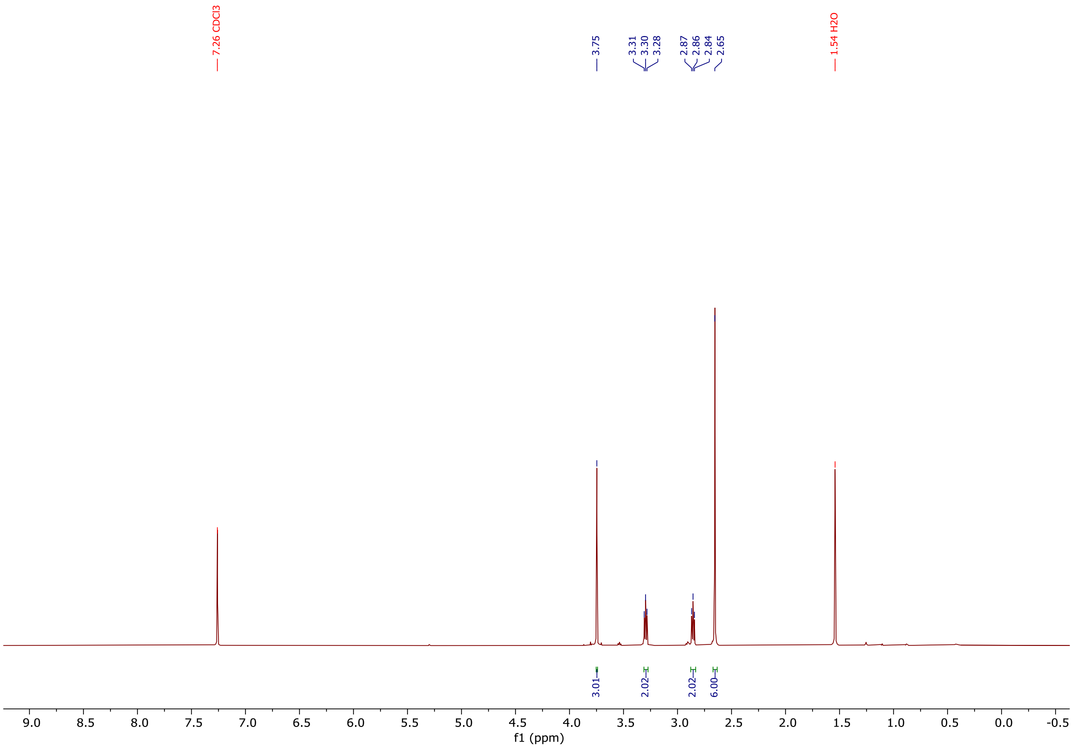

^{13}C NMR (151 MHz, $CDCl_3$)

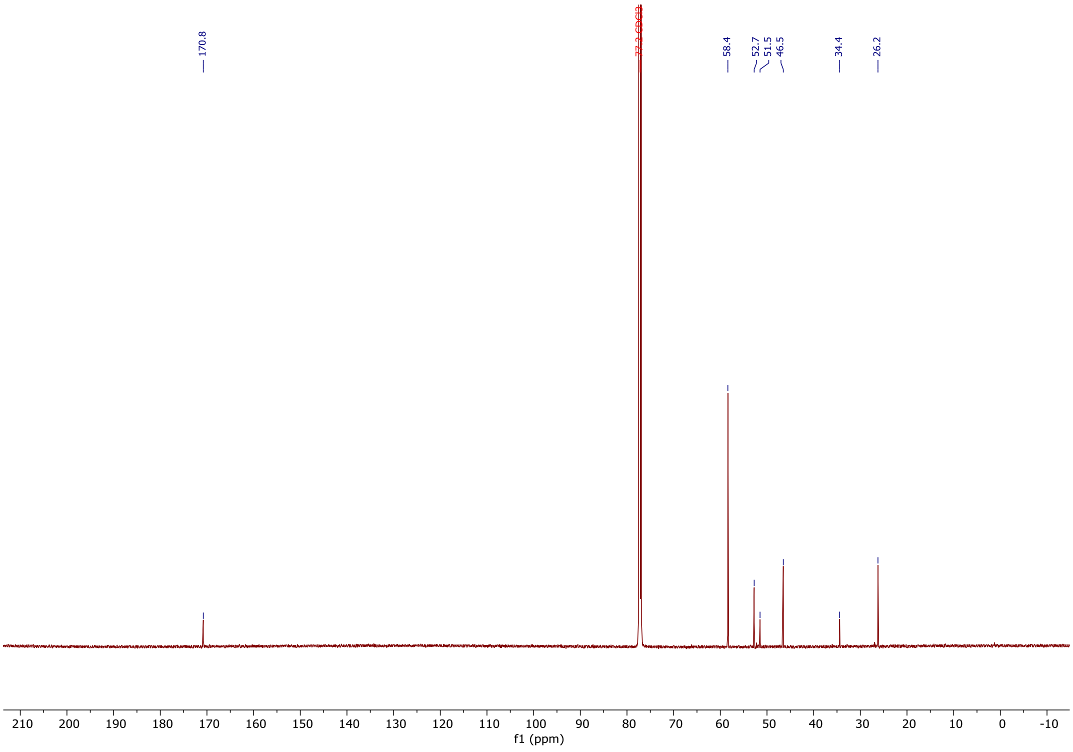

1-((4,4-Difluorocyclohexyl)sulfonyl)-3-iodobicyclo[1.1.1]pentane, 7h-I

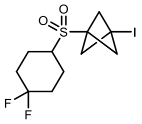

^{1}H NMR (600 MHz, $CDCl_3$)

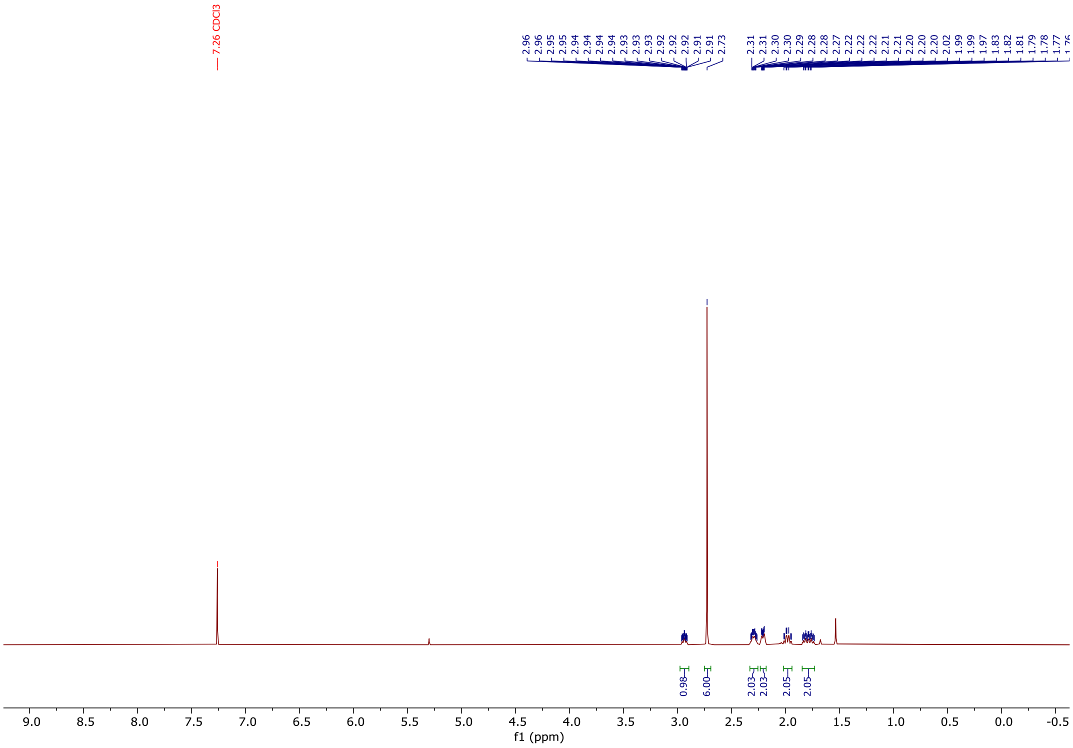

^{13}C NMR (151 MHz, $CDCl_3$)

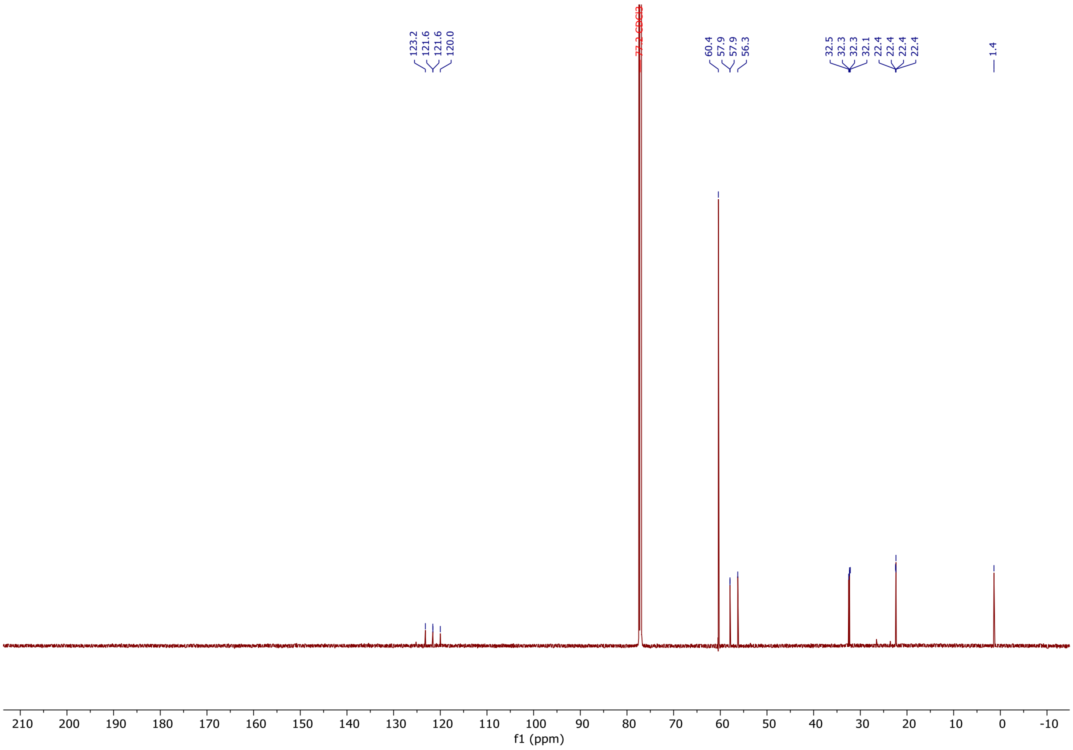

^{19}F NMR (377 MHz, $CDCl_3$)

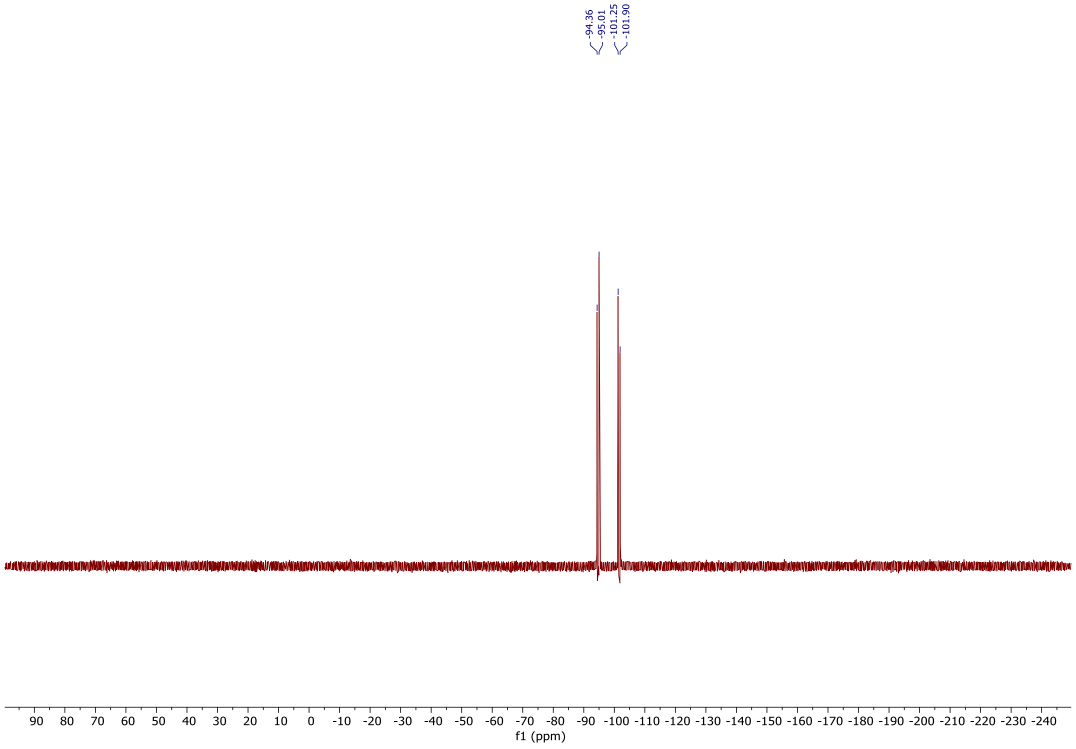

1-Bromo-3-((4,4-difluorocyclohexyl)sulfonyl)bicyclo[1.1.1]pentane, 7h-Br

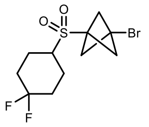

^{1}H NMR (600 MHz, $CDCl_3$)

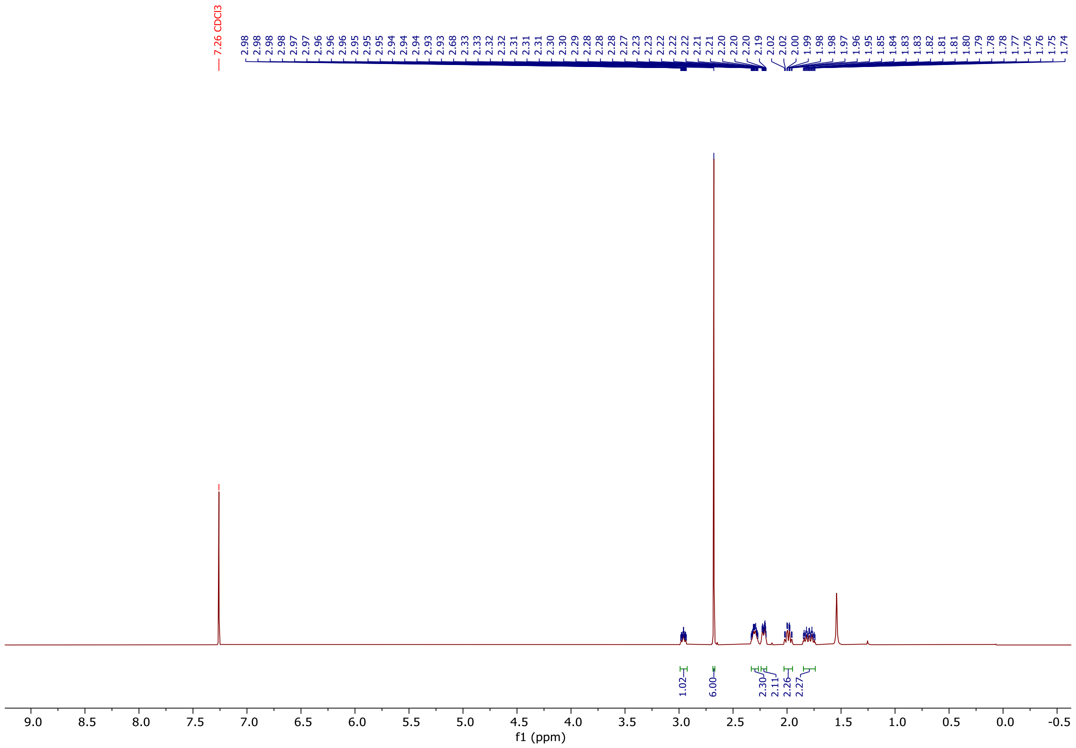

^{13}C NMR (151 MHz, $CDCl_3$)

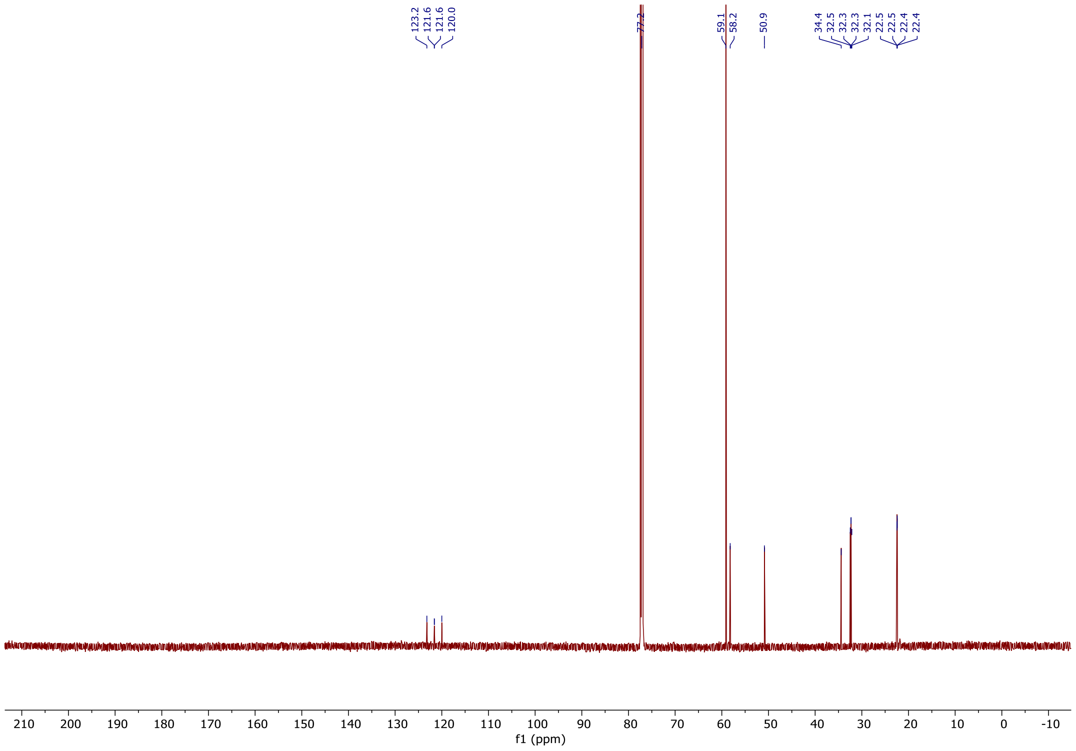

^{19}F NMR (377 MHz, $CDCl_3$)

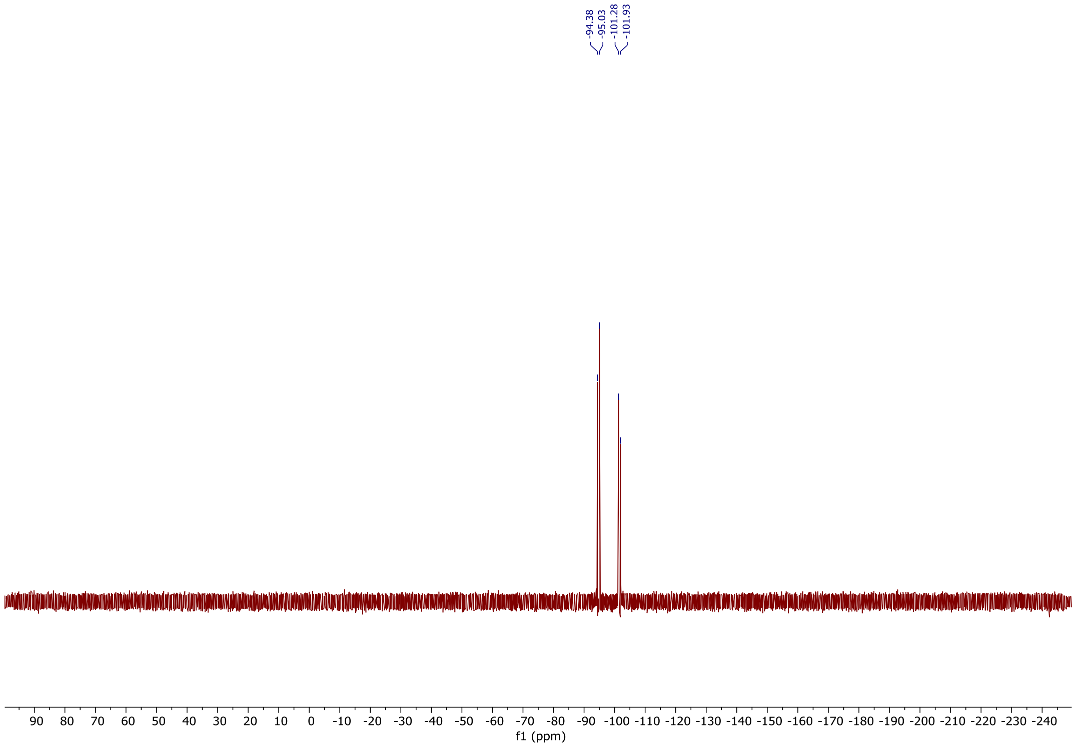

(1-(Bicyclo[1.1.1]pentan-1-ylsulfonyl)-3-bromobicyclo[1.1.1]pentane, 3i-I

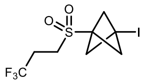

^{1}H NMR (400 MHz, $CDCl_3$)

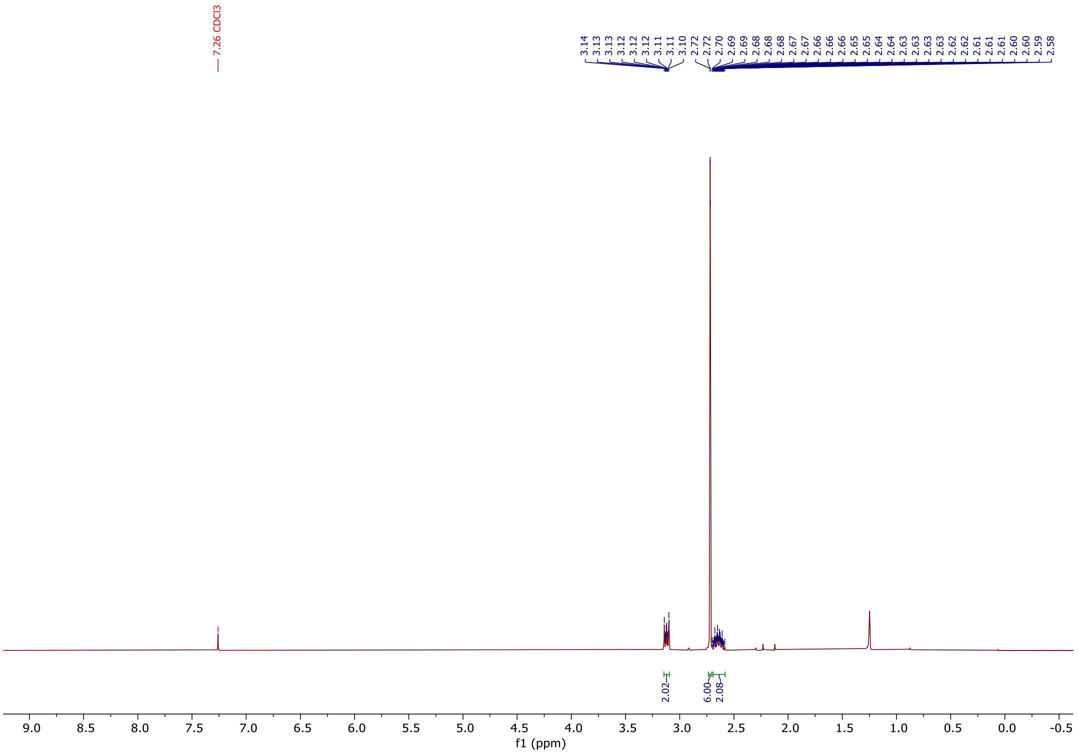

^{13}C NMR (101 MHz, $CDCl_3$)

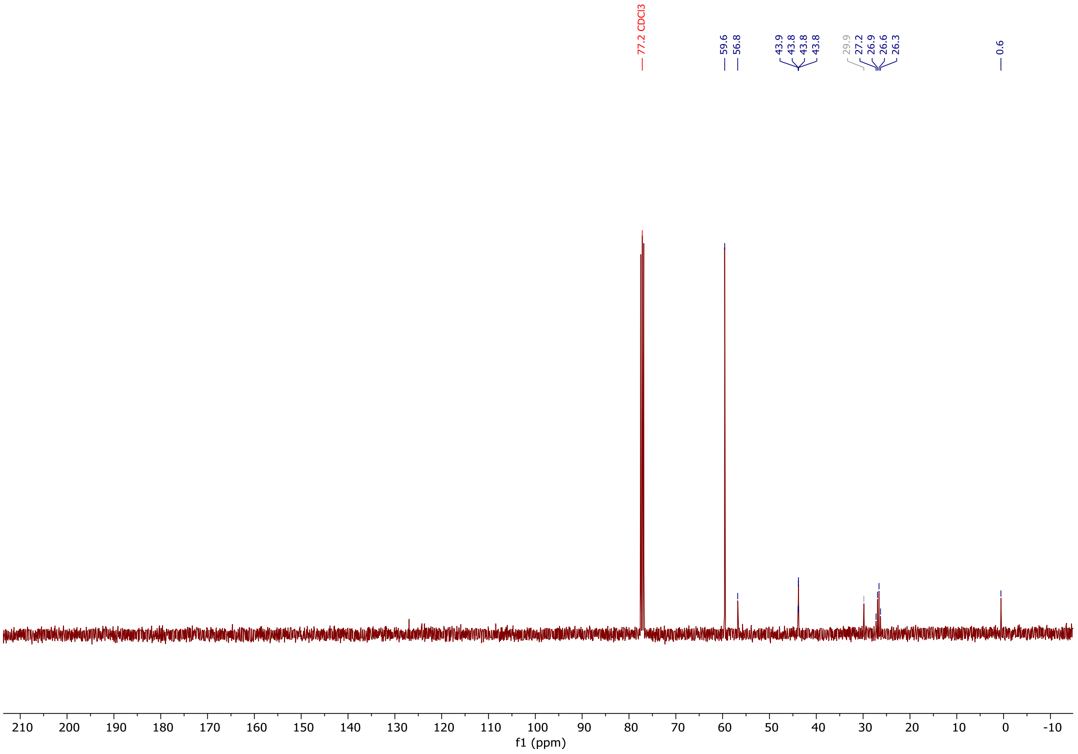

^{19}F NMR (377 MHz, $CDCl_3$)

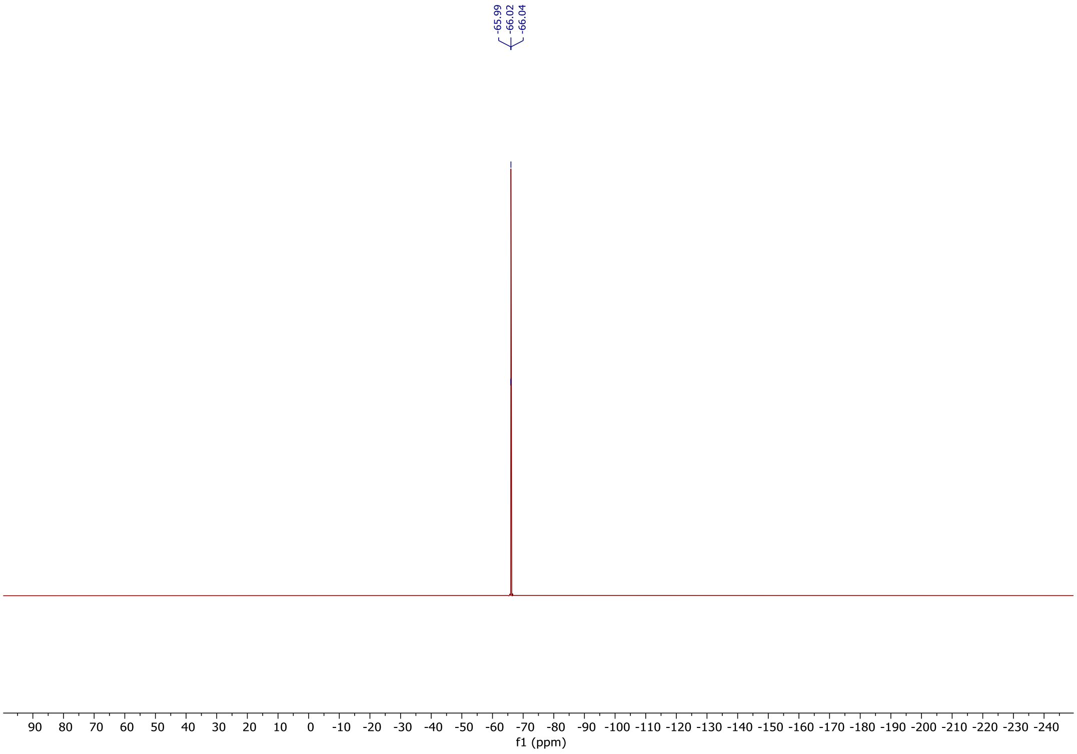

3-(((3-Iodobicyclo[1.1.1]pentan-1-yl)sulfonyl)methyl)-3-methyloxetane, 7j-I

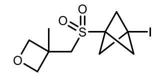

^{1}H NMR (600 MHz, $CDCl_3$)

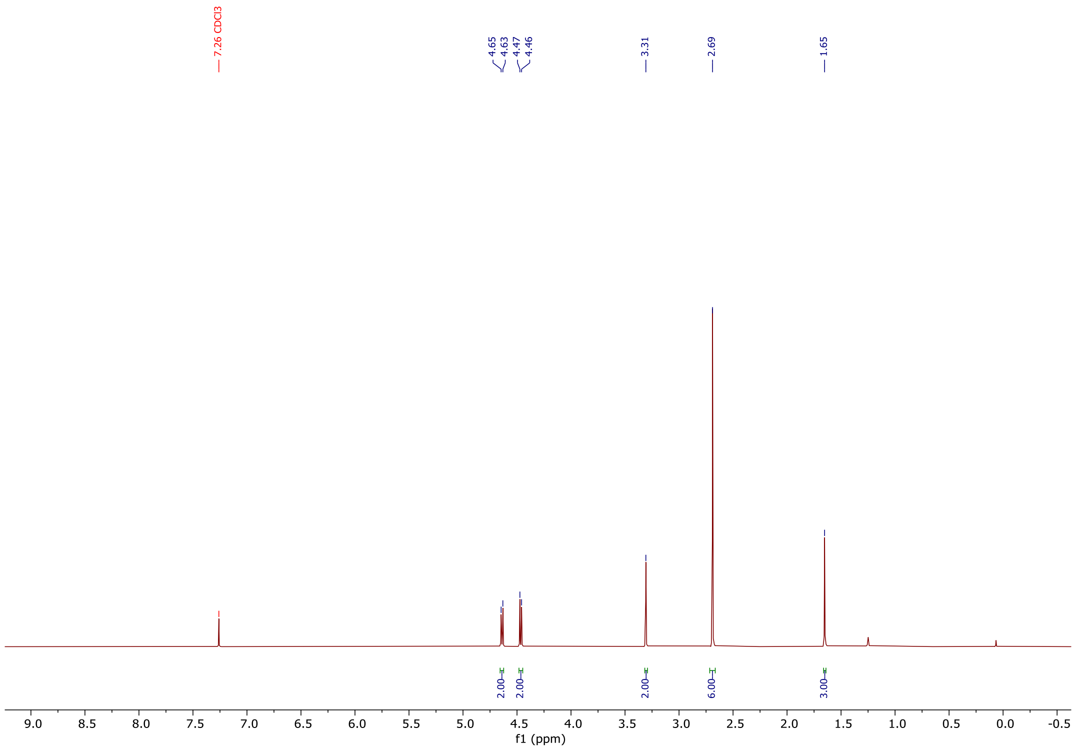

^{13}C NMR (151 MHz, $CDCl_3$)

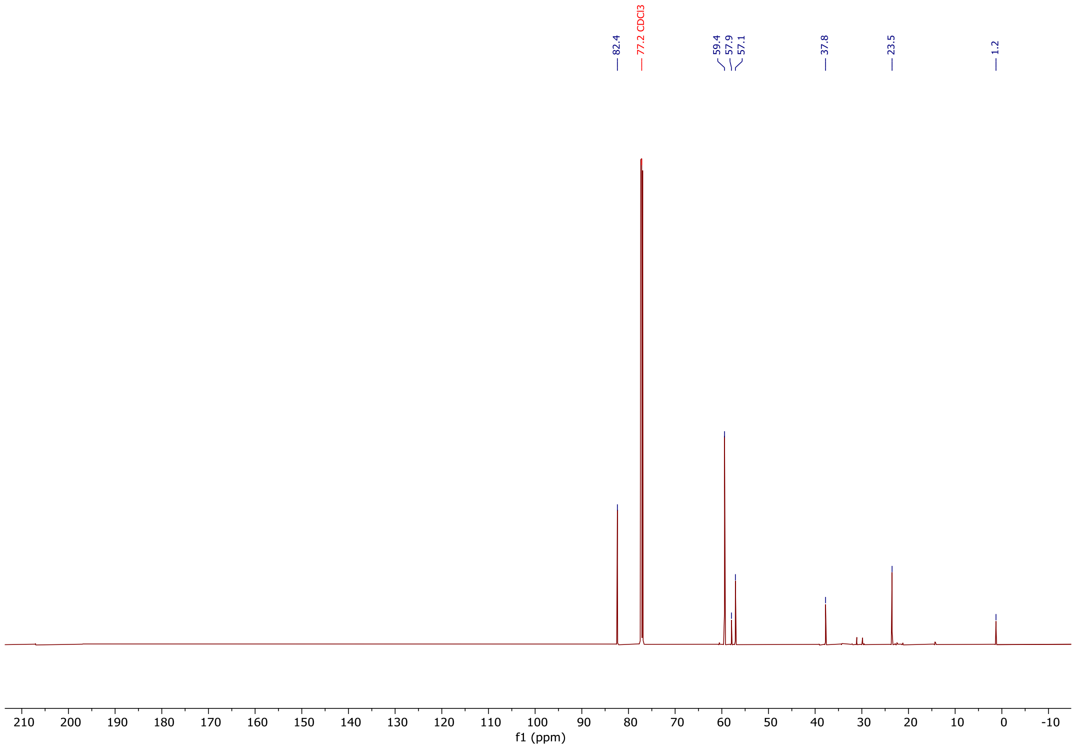

3-(((3-Bromobicyclo[1.1.1]pentan-1-yl)sulfonyl)methyl)-3-methyloxetane, 7j-Br

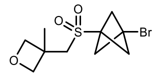

^{1}H NMR (600 MHz, $CDCl_3$)

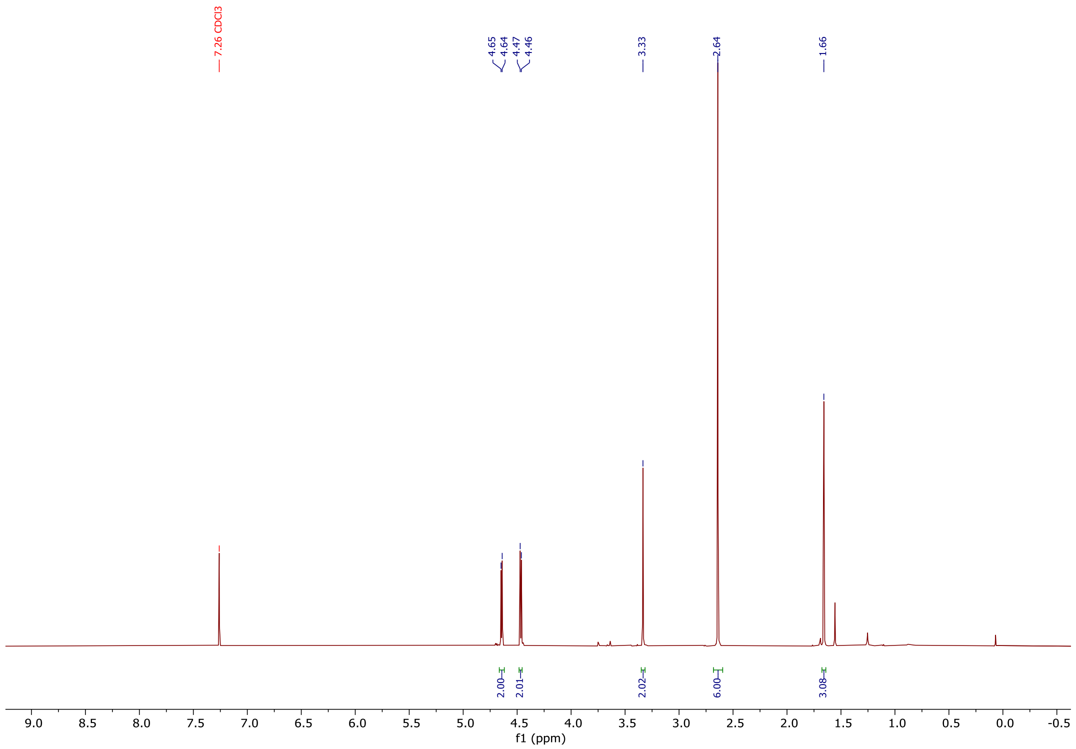

^{13}C NMR (151 MHz, $CDCl_3$)

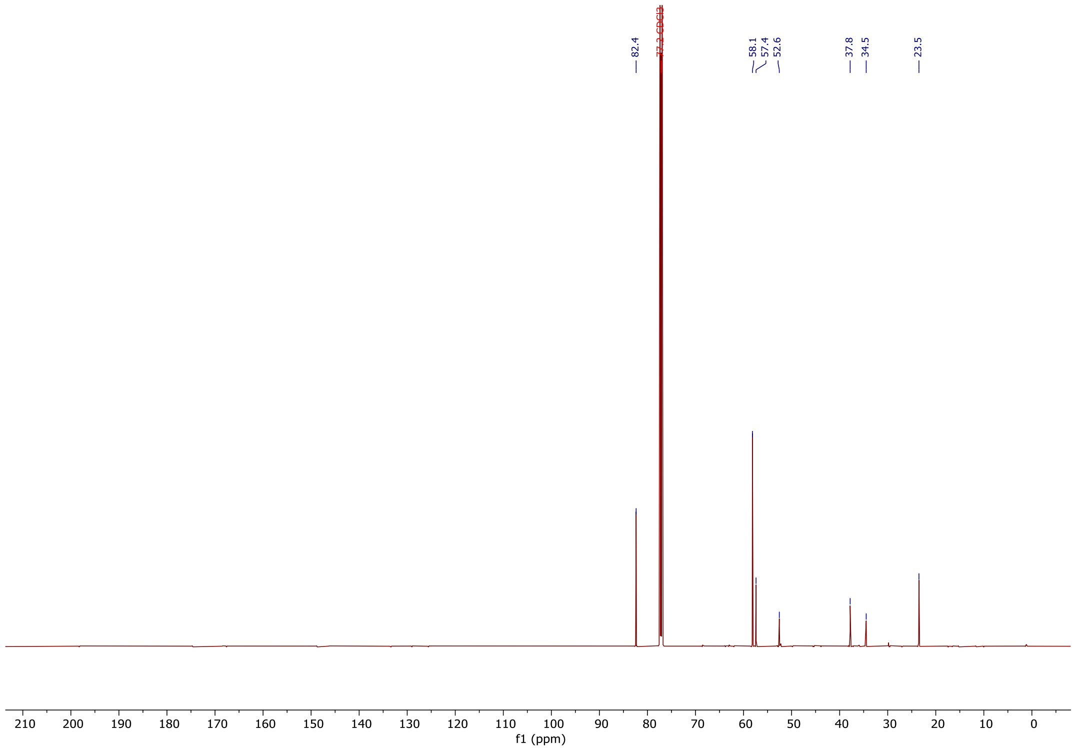

3-((3-Iodobicyclo[1.1.1]pentan-1-yl)sulfonyl)tetrahydrofuran, 7k-I

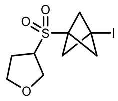

^{1}H NMR (600 MHz, $CDCl_3$)

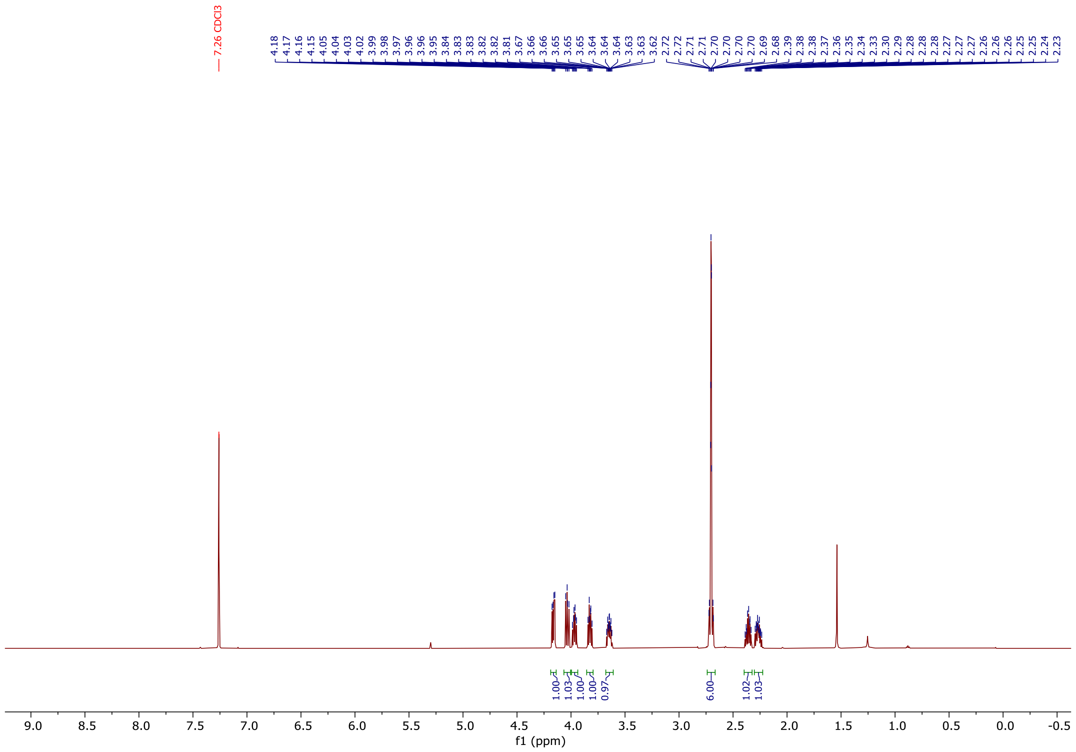

^{13}C NMR (151 MHz, $CDCl_3$)

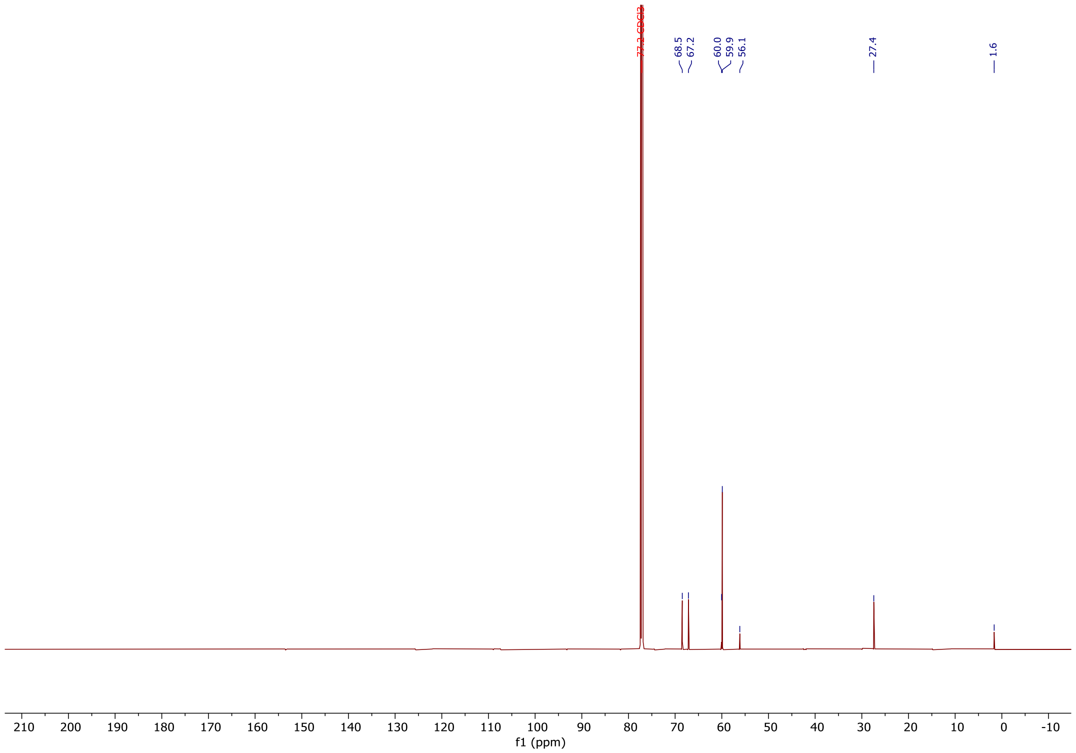

3-((3-Bromobicyclo[1.1.1]pentan-1-yl)sulfonyl)tetrahydrofuran, 7k-Br

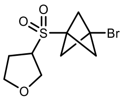

^{1}H NMR (600 MHz, $CDCl_3$)

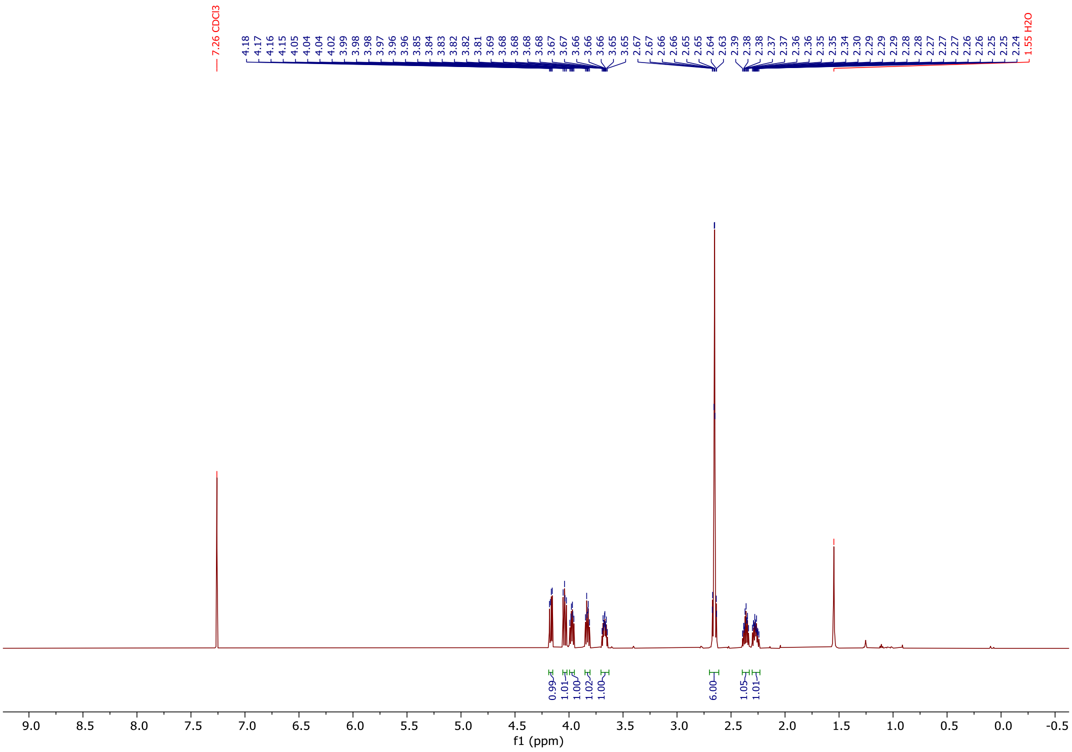

^{13}C NMR (151 MHz, $CDCl_3$)

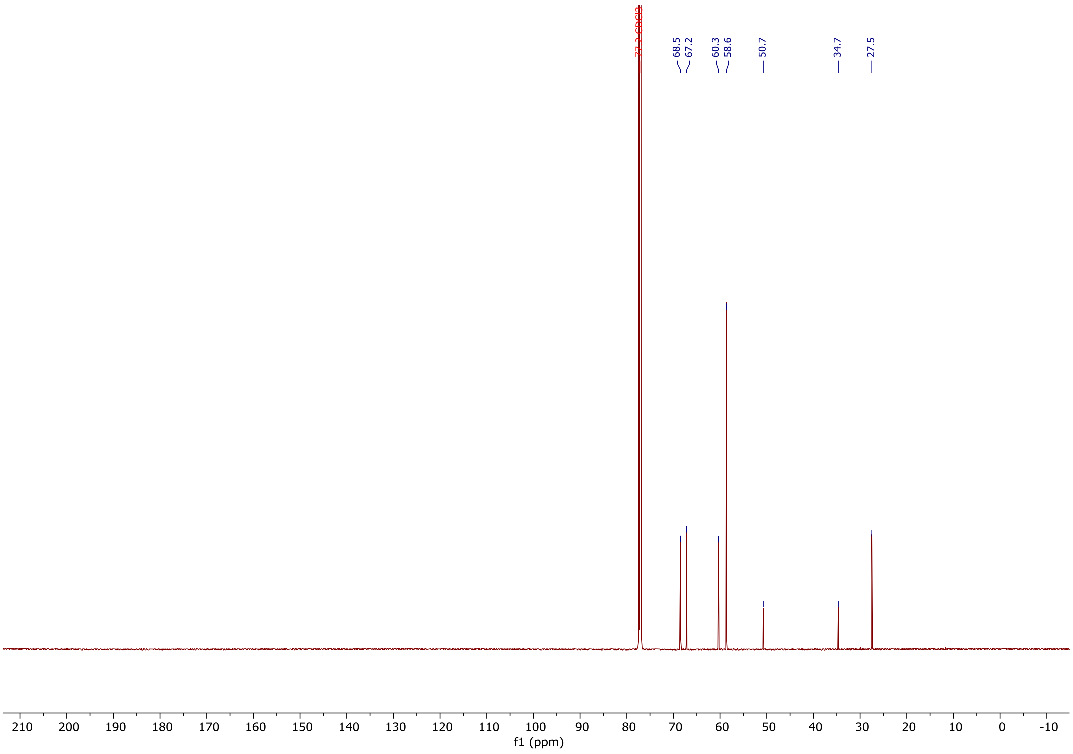

1-(Bicyclo[1.1.1]pentan-1-ylsulfonyl)-3-bromobicyclo[1.1.1]pentane, 7l-Br

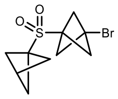

^{1}**H NMR** (500 MHz, $CDCl_3$)

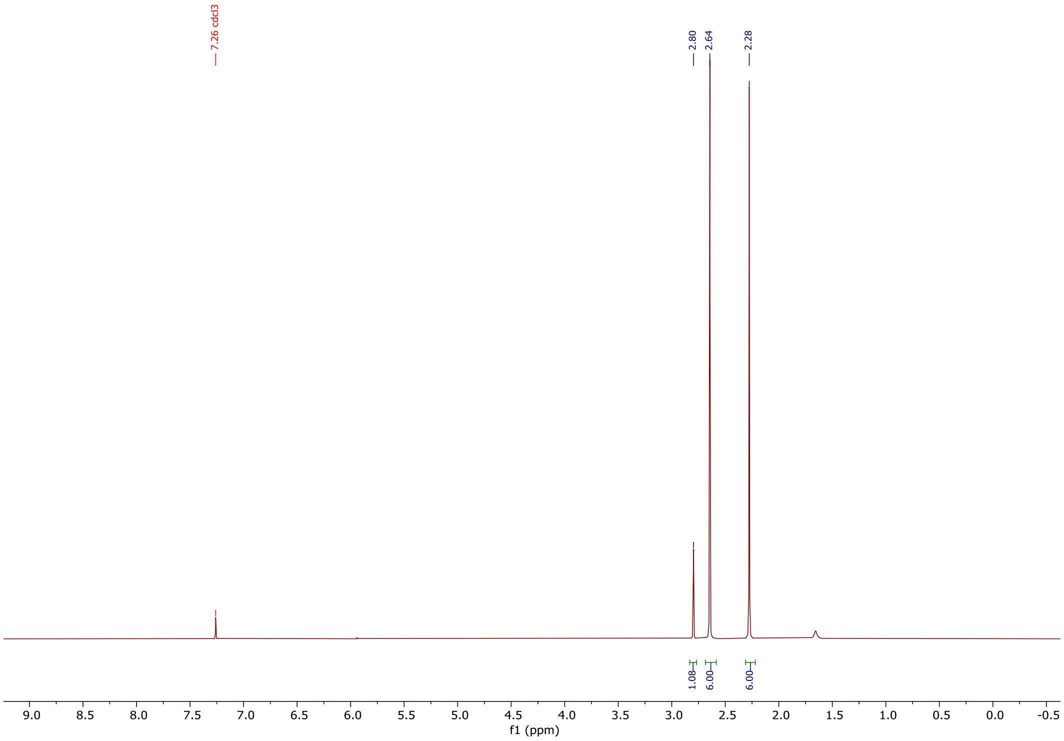

^{13}**C NMR** (126 MHz, $CDCl_3$)

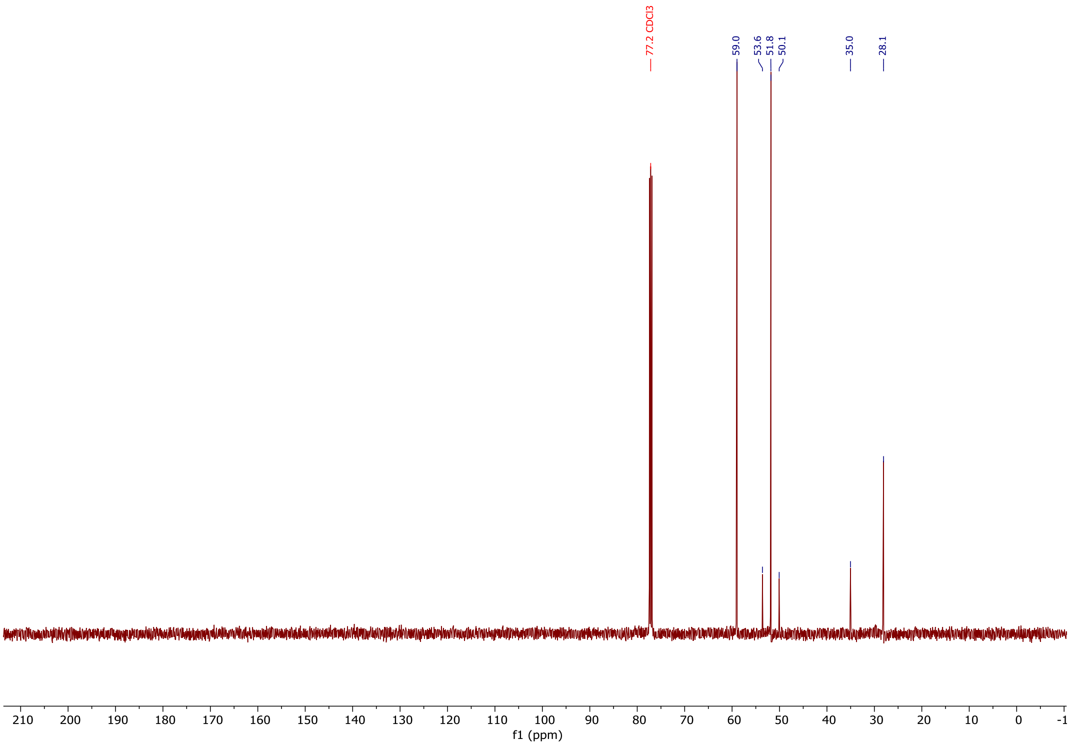

6.5 Sulfonyl Cyclobutyl Halides

1-Iodo-*N*,*N*-diisopropyl-3-tosylcyclobutane-1-carboxamide, 10a-I (major diasteromer)

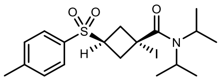

^{1}H NMR (600 MHz, $CDCl_3$)

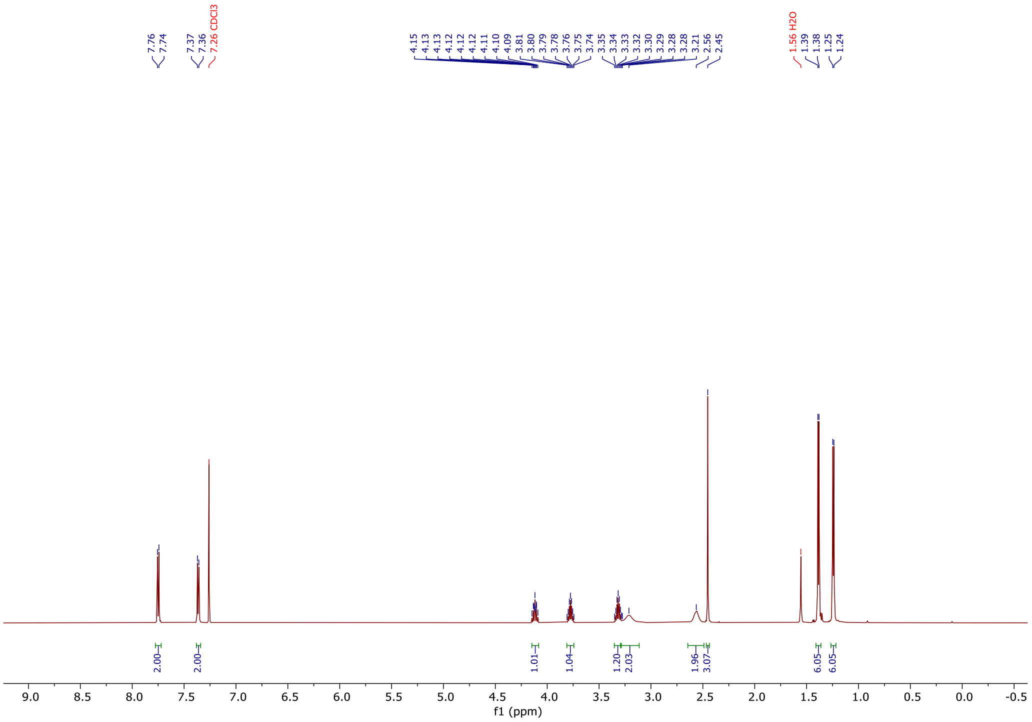

^{13}C NMR (151 MHz, $CDCl_3$)

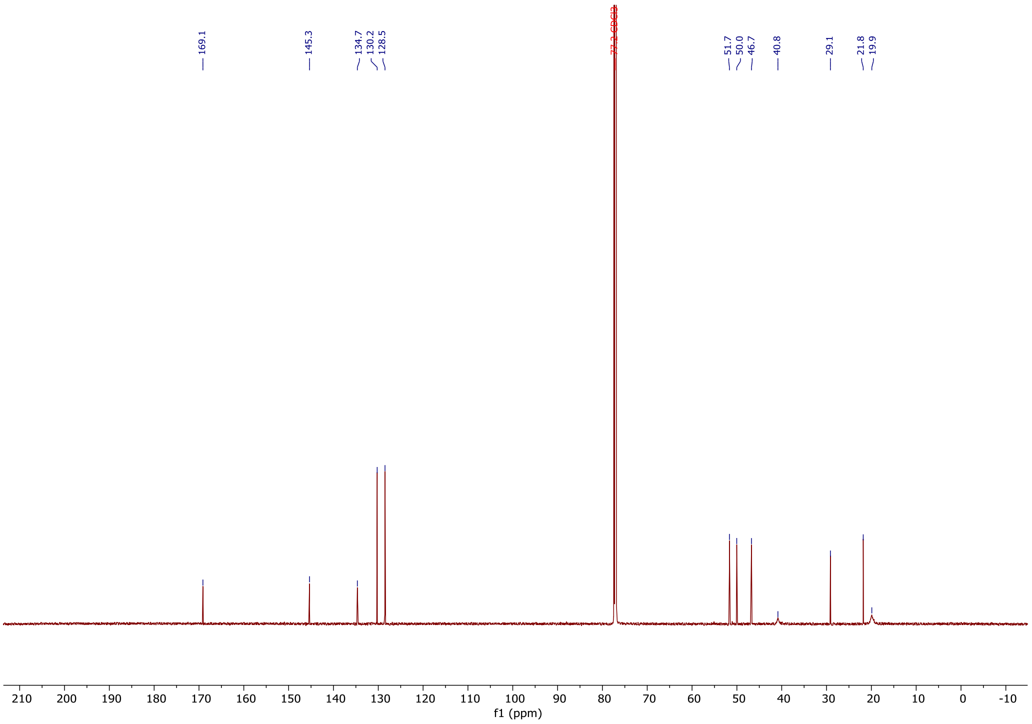

NOESY (600 MHz, $CDCl_3$)

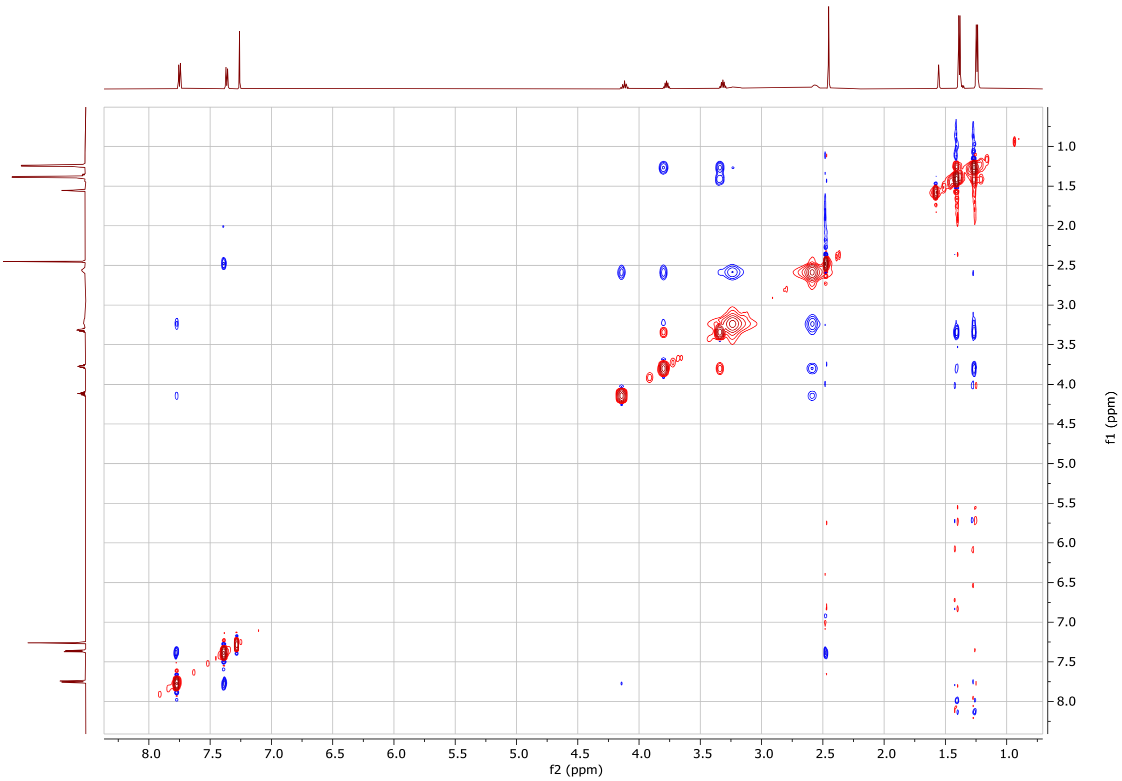

1-Bromo-*N*,*N*-diisopropyl-3-tosylcyclobutane-1-carboxamide, 10a-Br

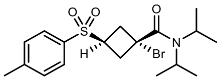

^{1}**H NMR** (600 MHz, $CDCl_3$)

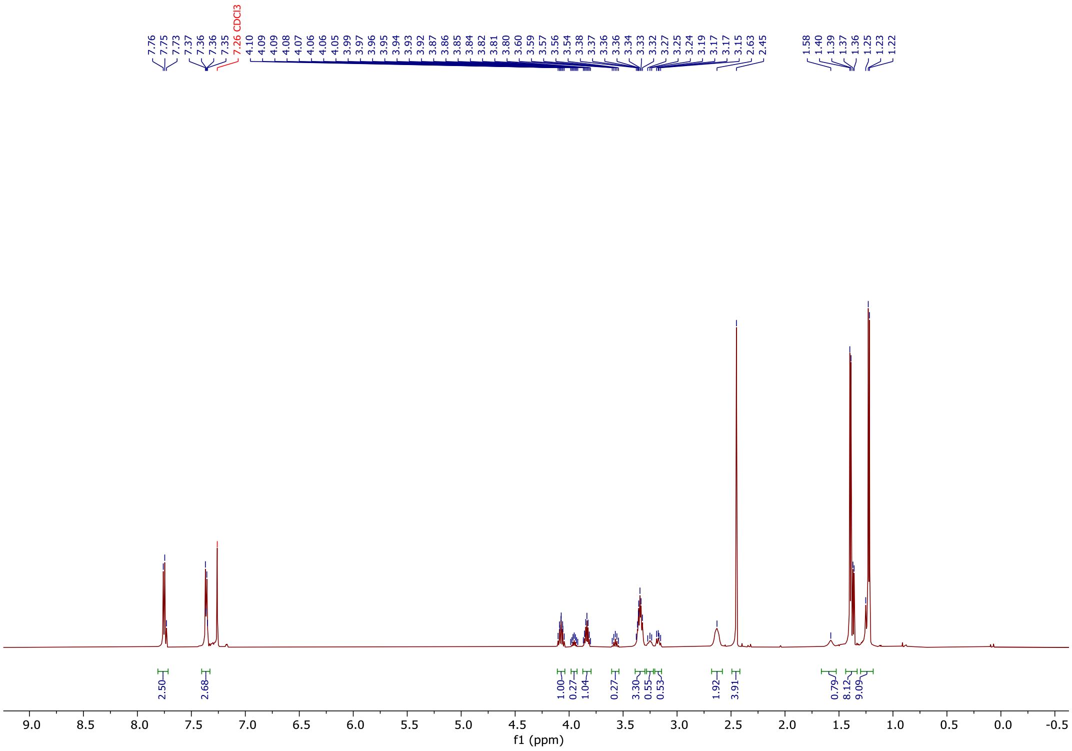

^{13}**C NMR** (151 MHz, $CDCl_3$)

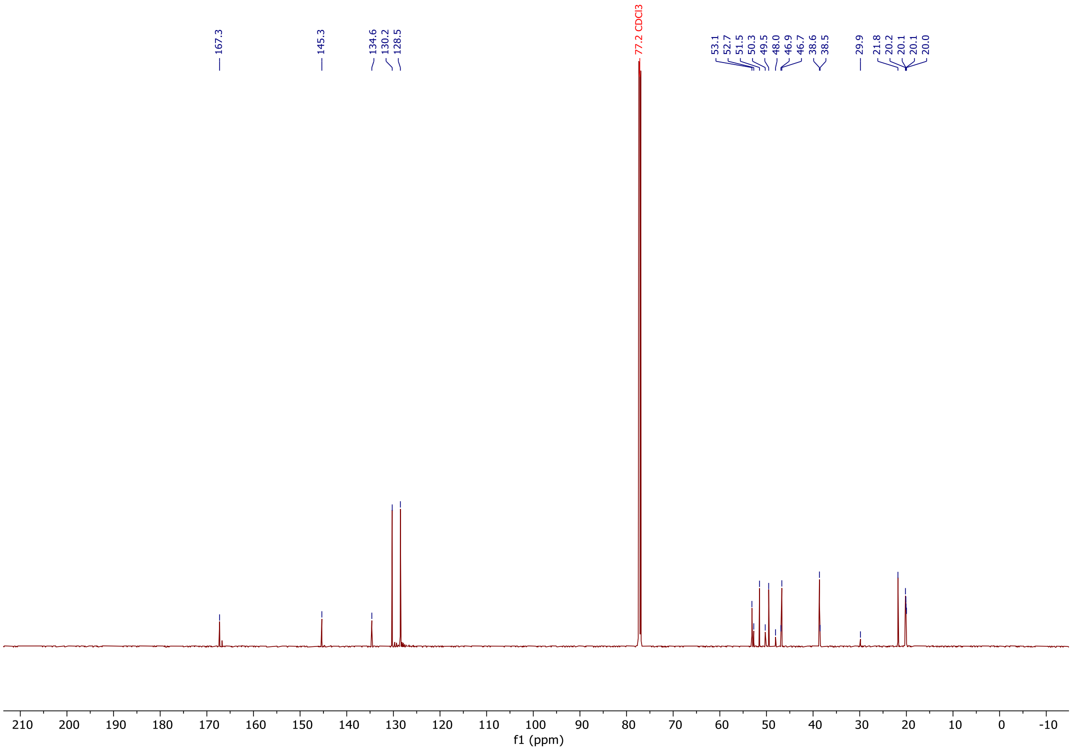

NOESY (600 MHz, $CDCl_3$)

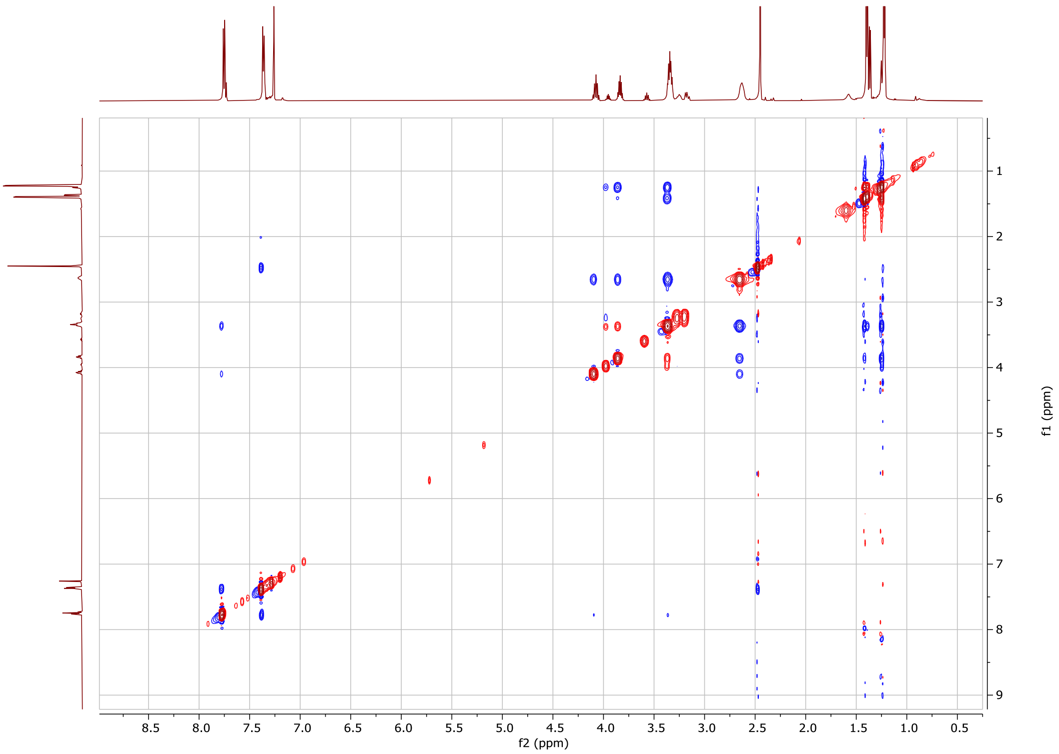

1-((3-Iodo-3-(phenylsulfonyl)cyclobutyl)sulfonyl)-4-methylbenzene, 10b-I

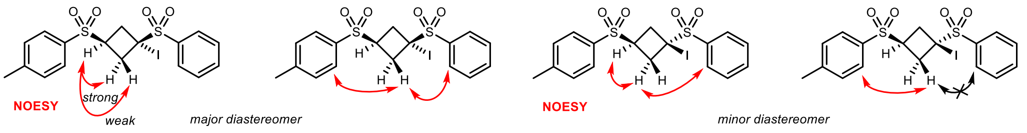

^{1}H NMR (600 MHz, $CDCl_3$)

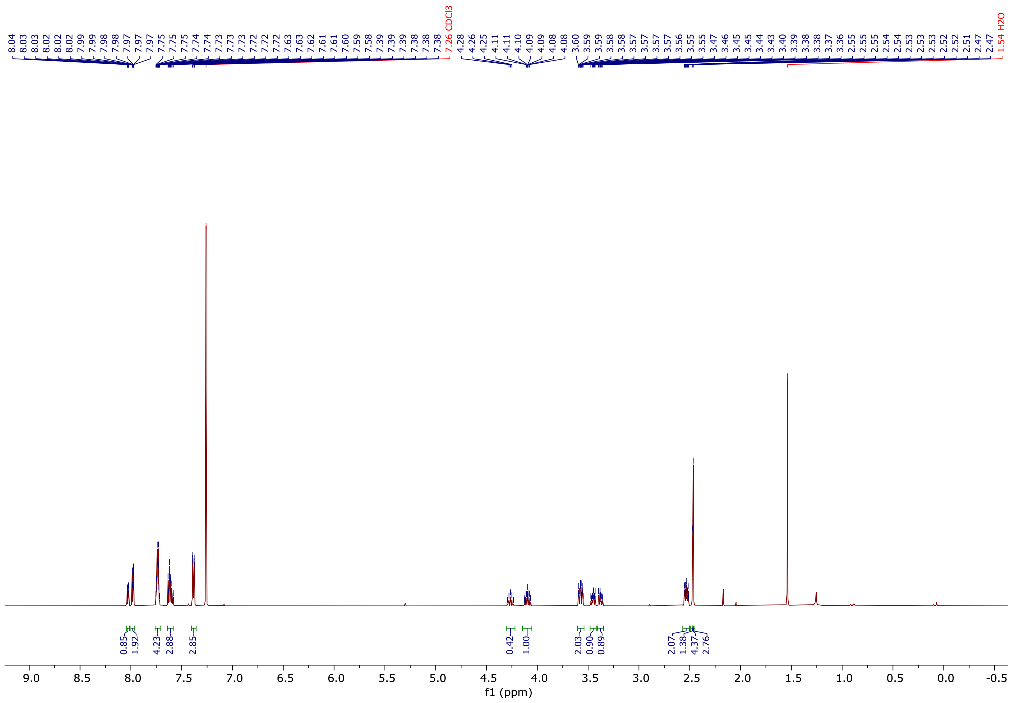

^{13}C NMR (151 MHz, $CDCl_3$)

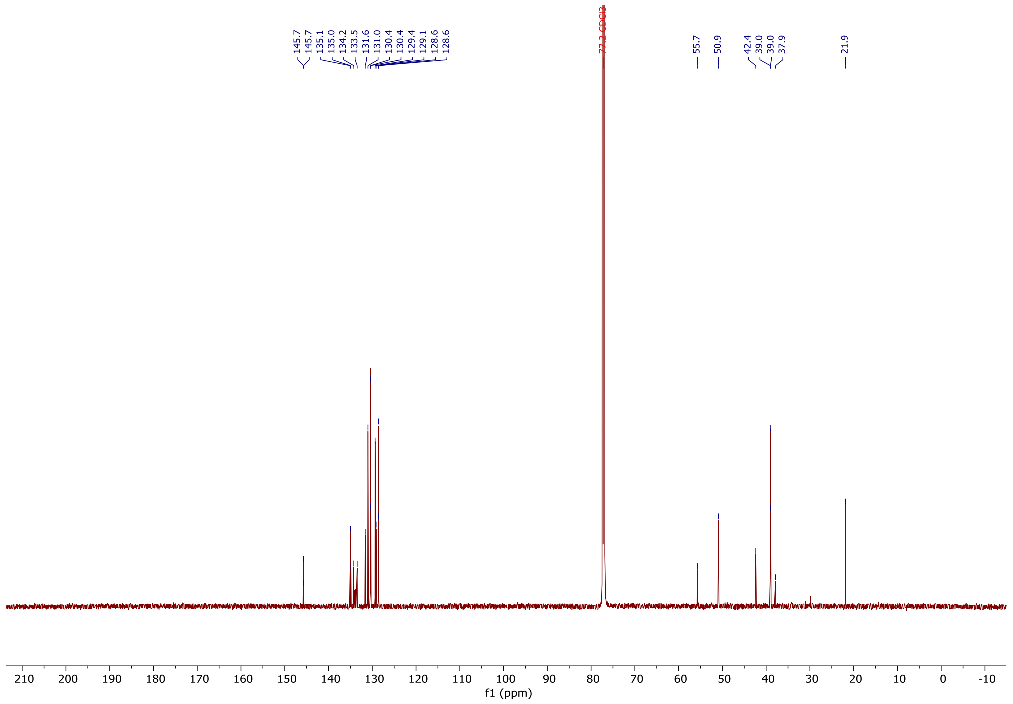

COSY (600 MHz, $CDCl_3$)

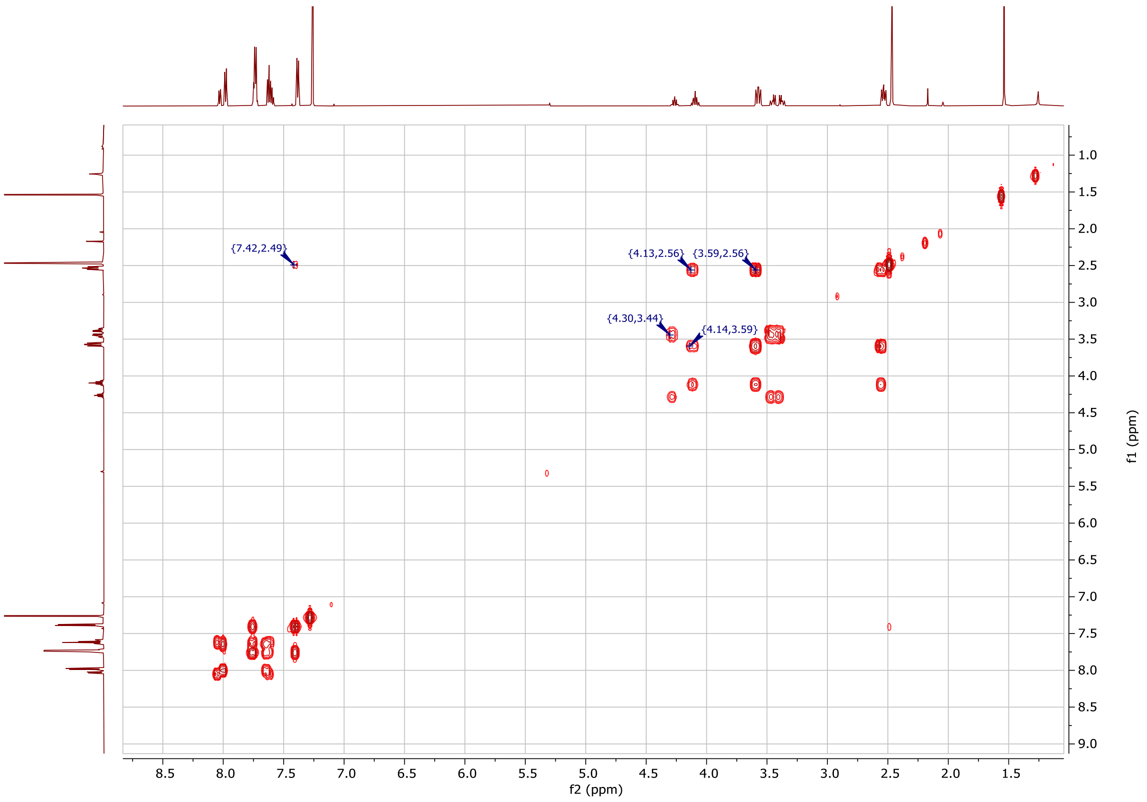

NOESY (600 MHz, $CDCl_3$)

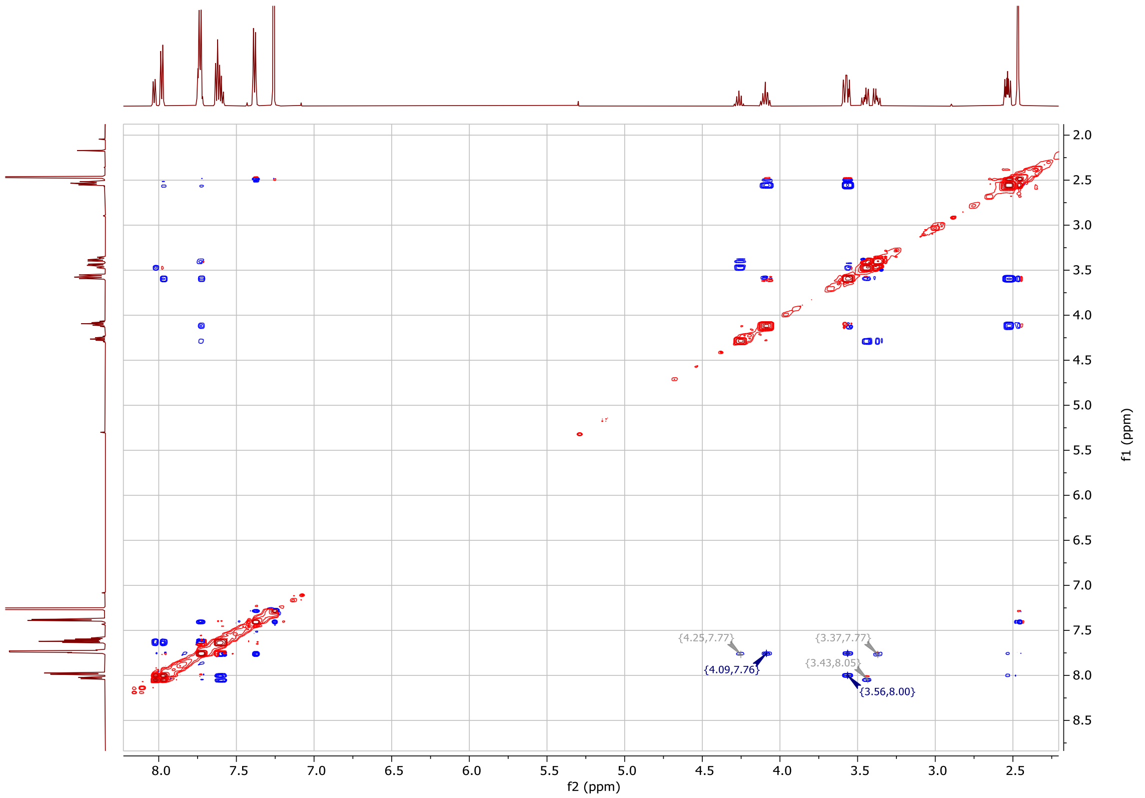

1-((3-(*tert*-Butylsulfonyl)-3-iodocyclobutyl)sulfonyl)-4-methylbenzene, 10c-I

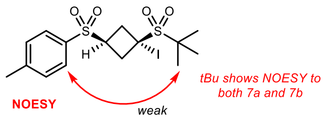

^{1}H NMR (600 MHz, $CDCl_3$)

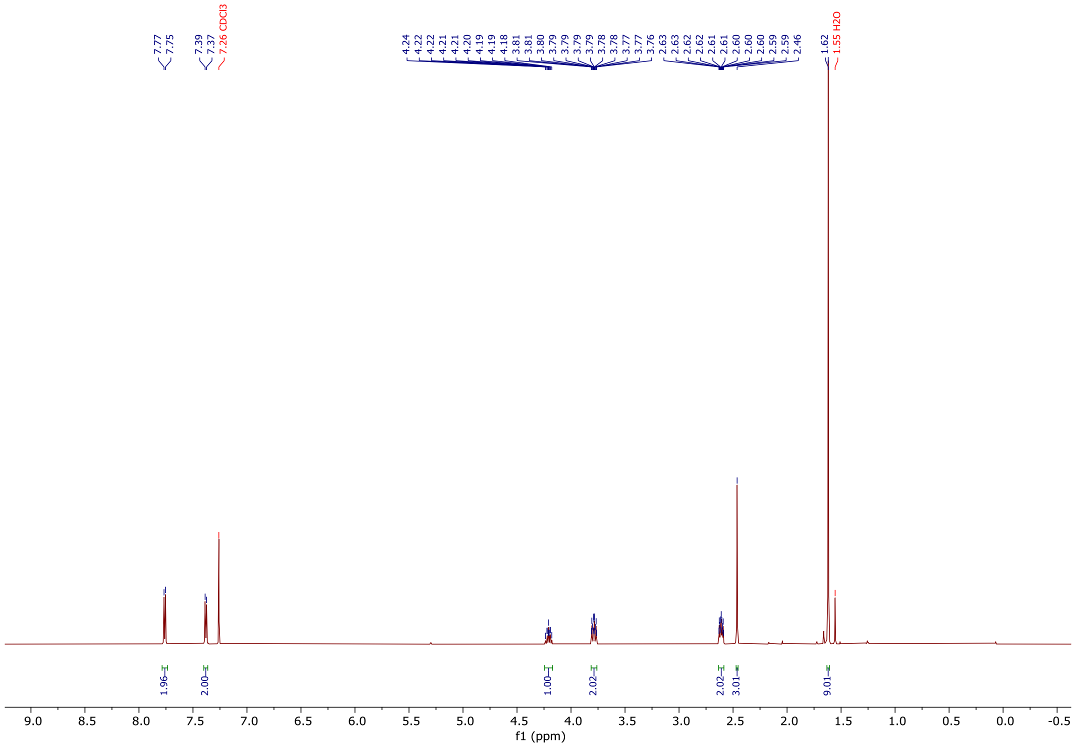

^{13}C NMR (151 MHz, $CDCl_3$)

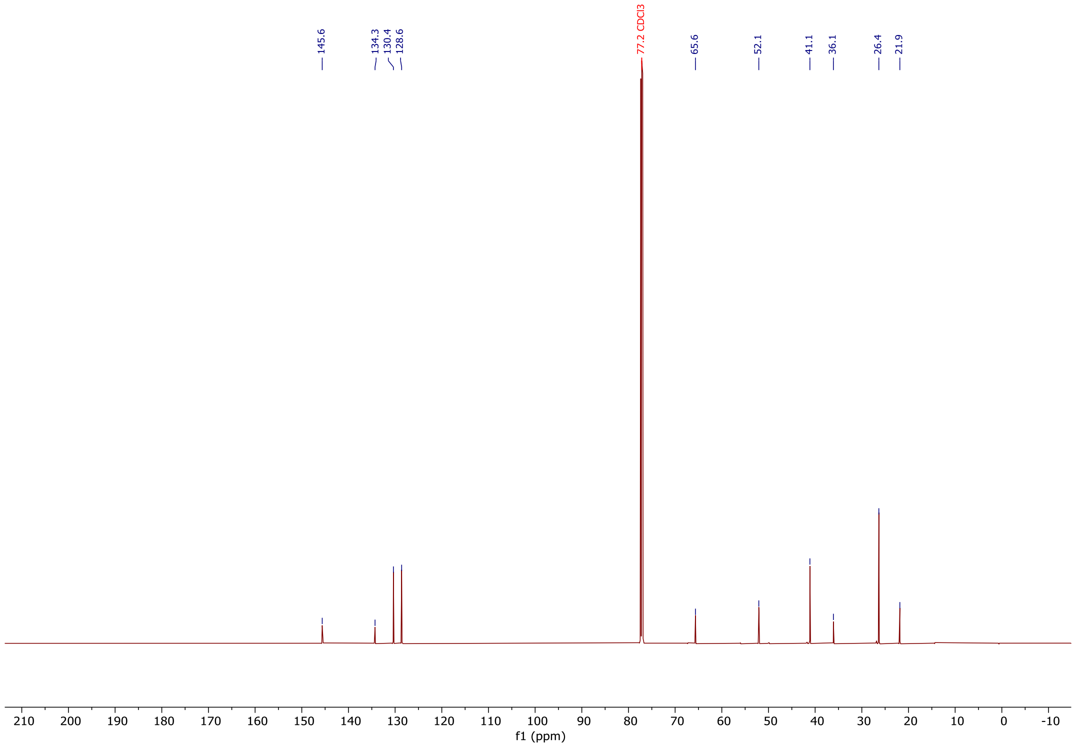

NOSEY (600 MHz, $CDCl_3$)

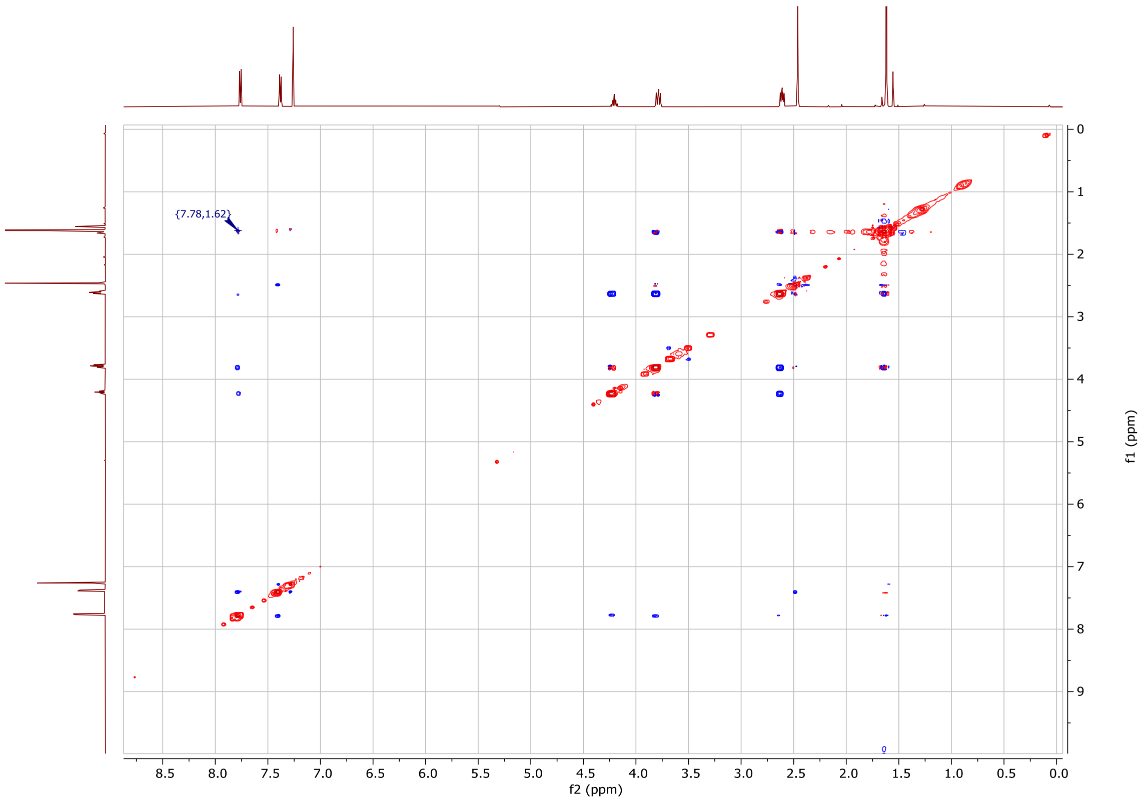

1-((3-Iodo-3-(phenylsulfonyl)cyclobutyl)sulfonyl)-4-methylbenzene, 10d-I

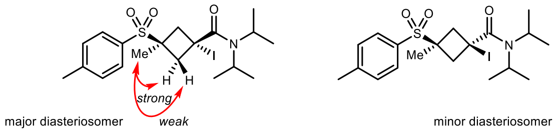

^{1}H NMR (600 MHz, CDCl$_3$)

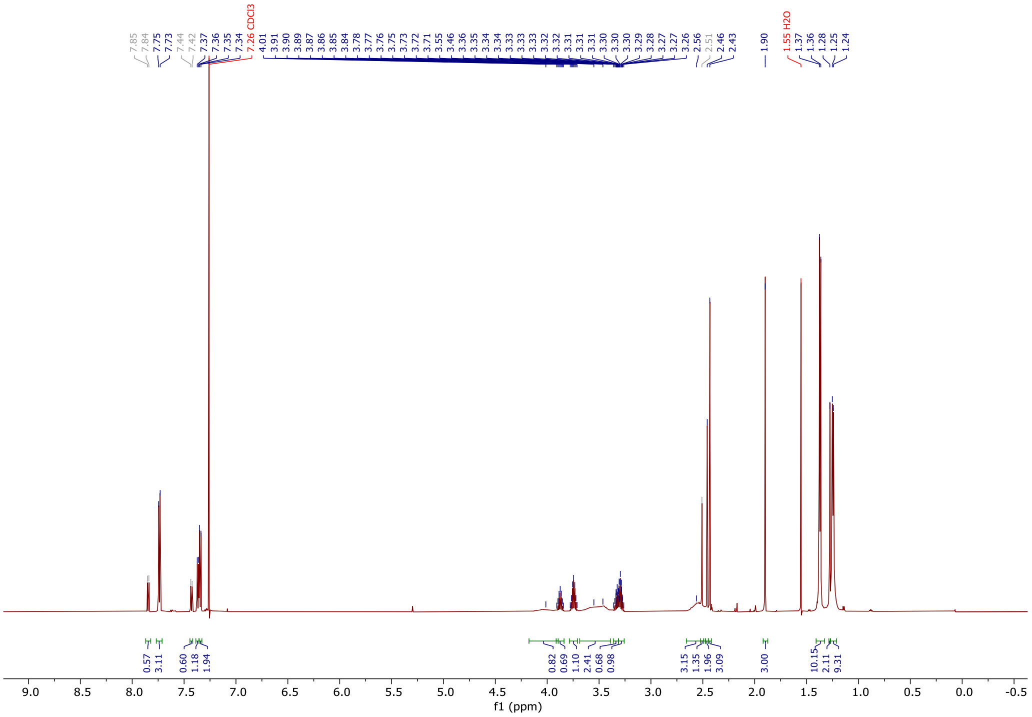

^{1}H NMR (600 MHz, CDCl$_3$)

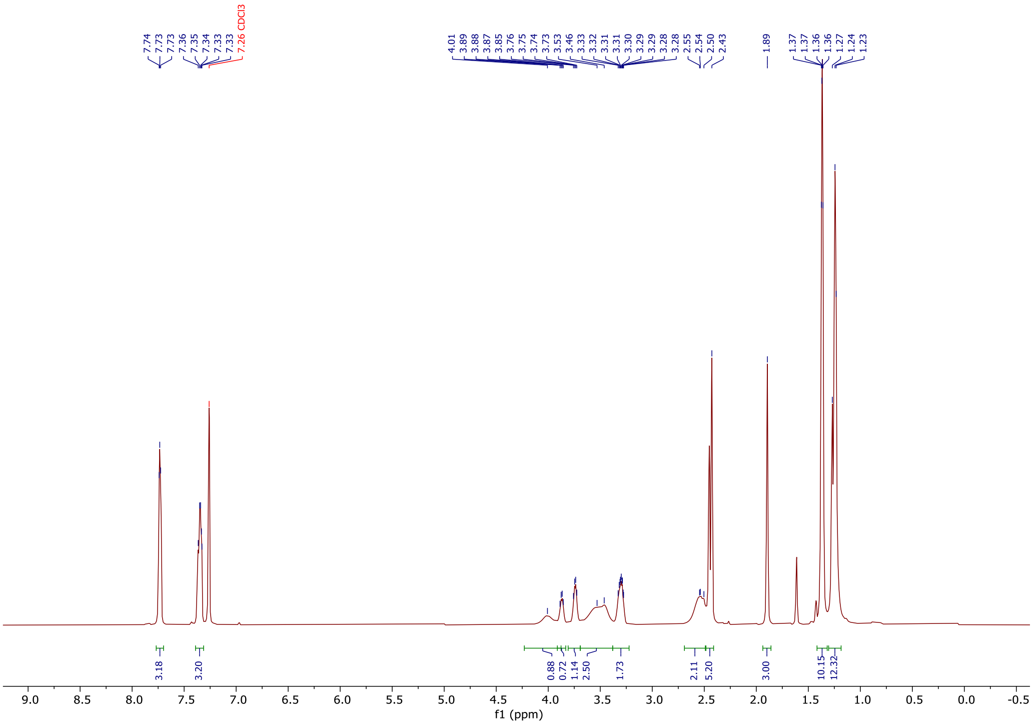

Note: Top spectra contains TsI, the bottom spectra is without TsI but is less well resolved.

^{13}C NMR (151 MHz, CDCl$_3$)

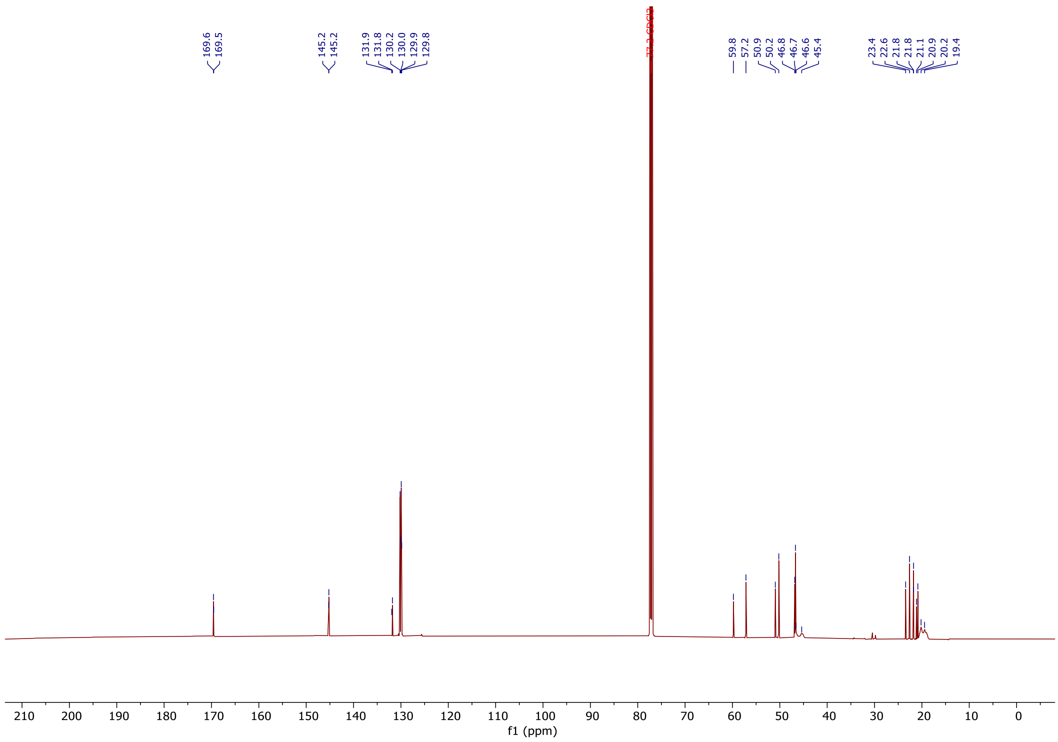

NOSEY (600 MHz, CDCl$_3$)

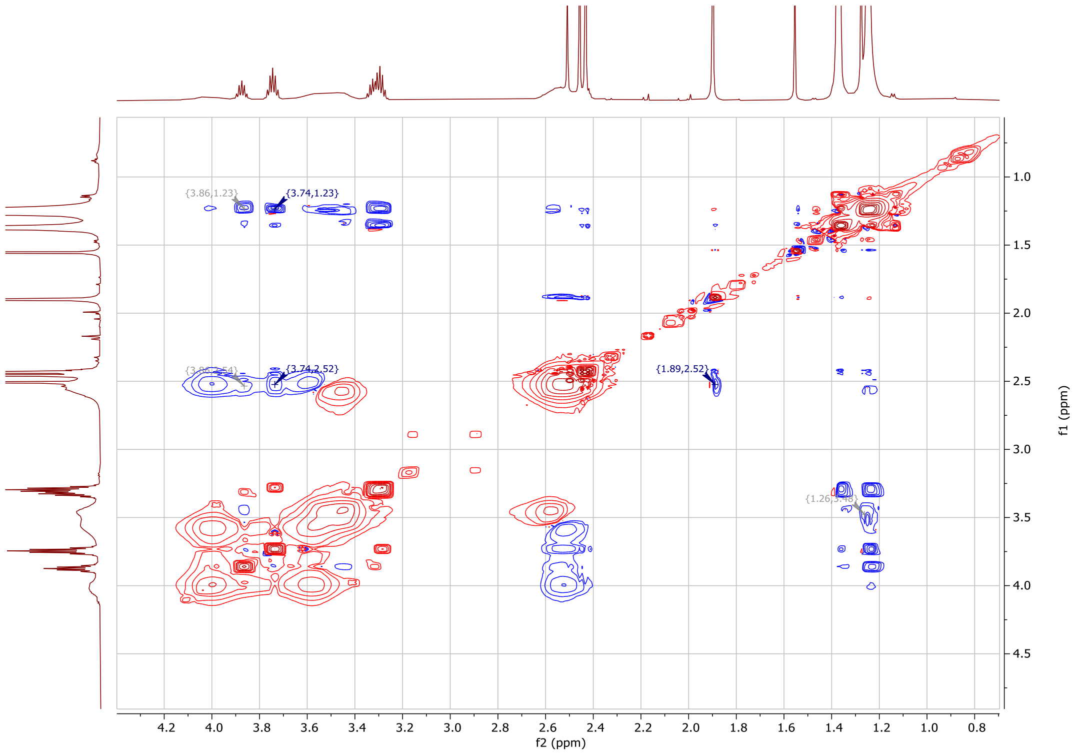

6.6 Sulfonyl Halides

4-Methylbenzenesulfonyl bromide

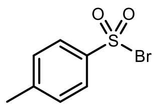

^{1}H NMR (500 MHz, $CDCl_3$)

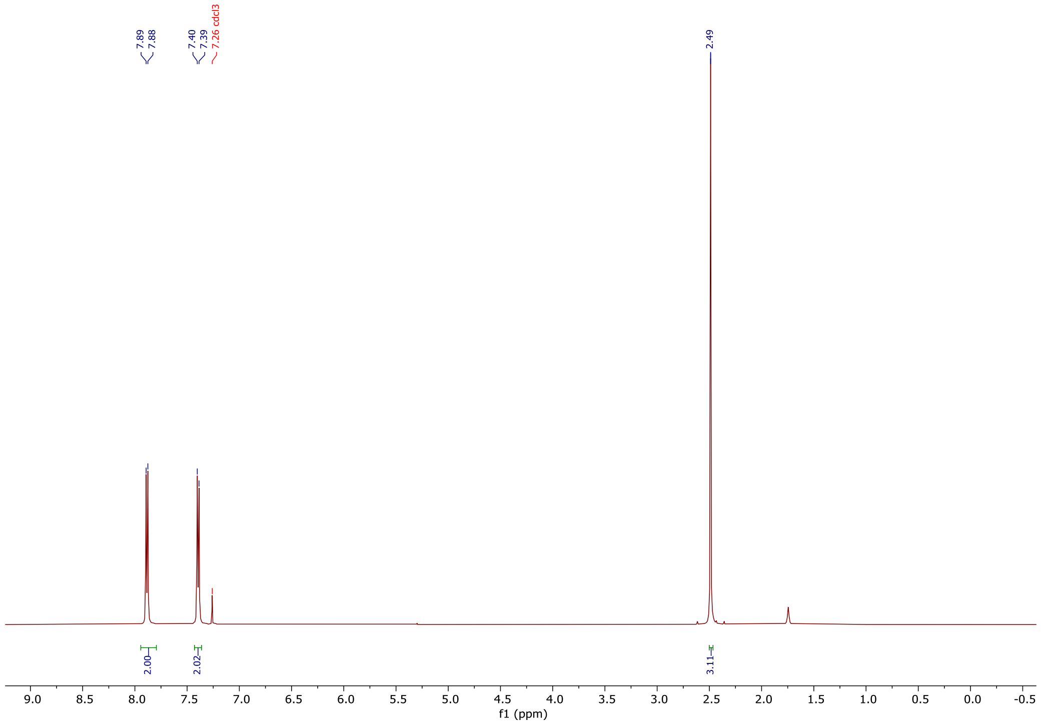

^{13}C NMR (126 MHz, $CDCl_3$)

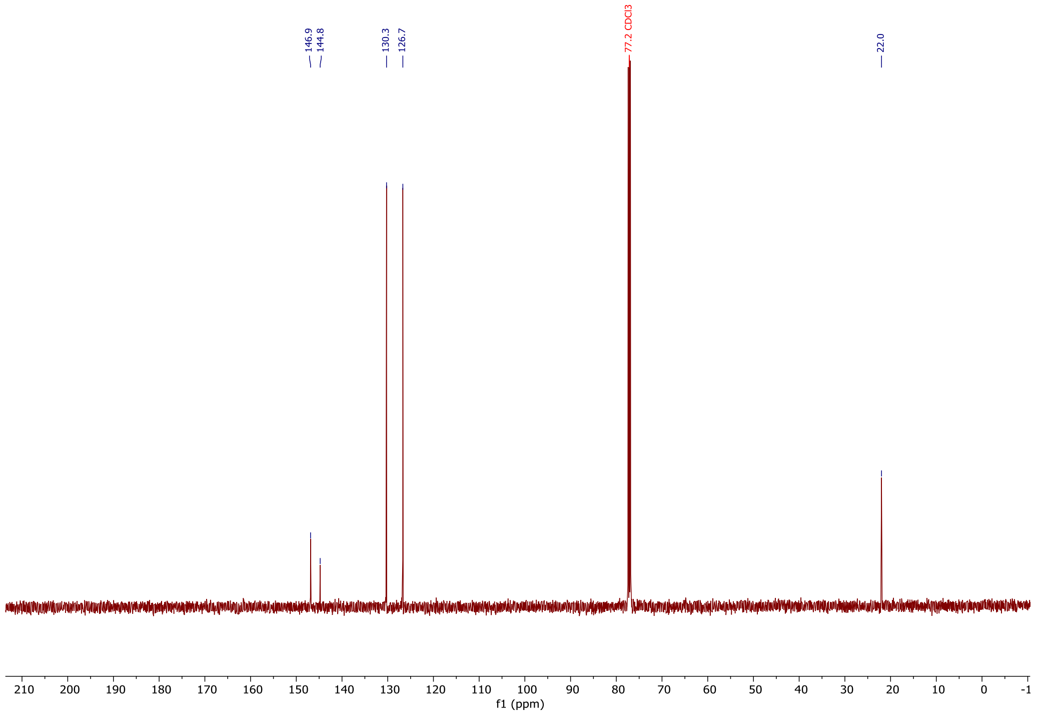

Benzenesulfonyl iodide

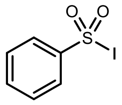

^{1}H NMR (500 MHz, $CDCl_3$)

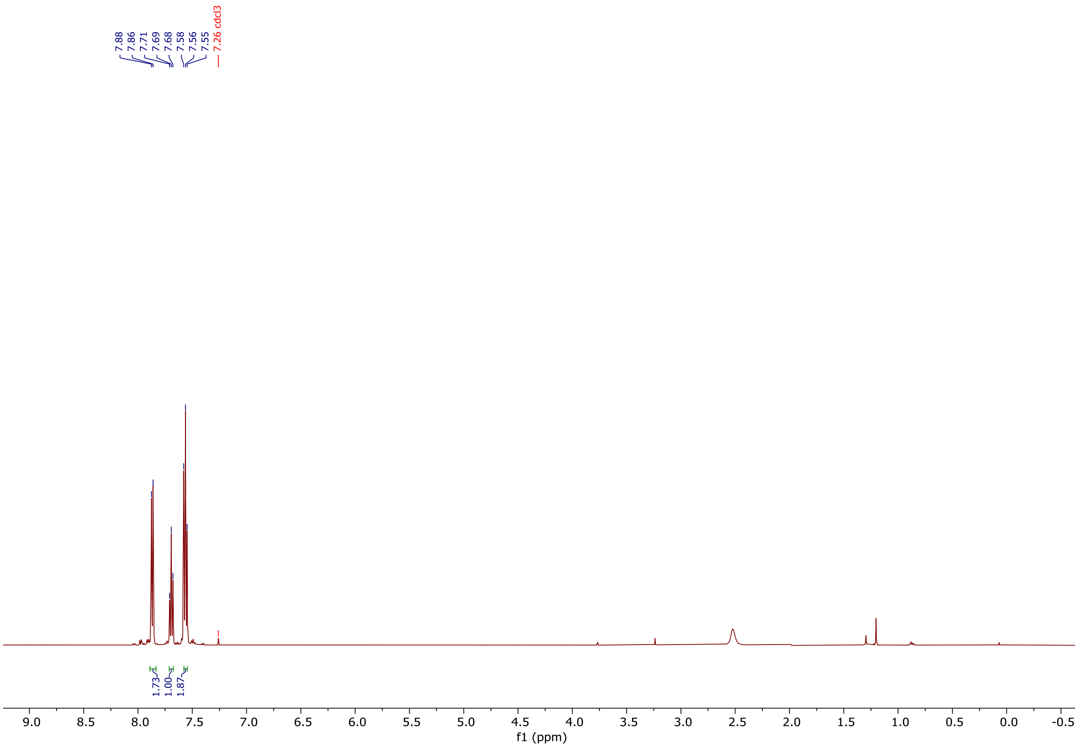

^{13}C NMR (126 MHz, $CDCl_3$)

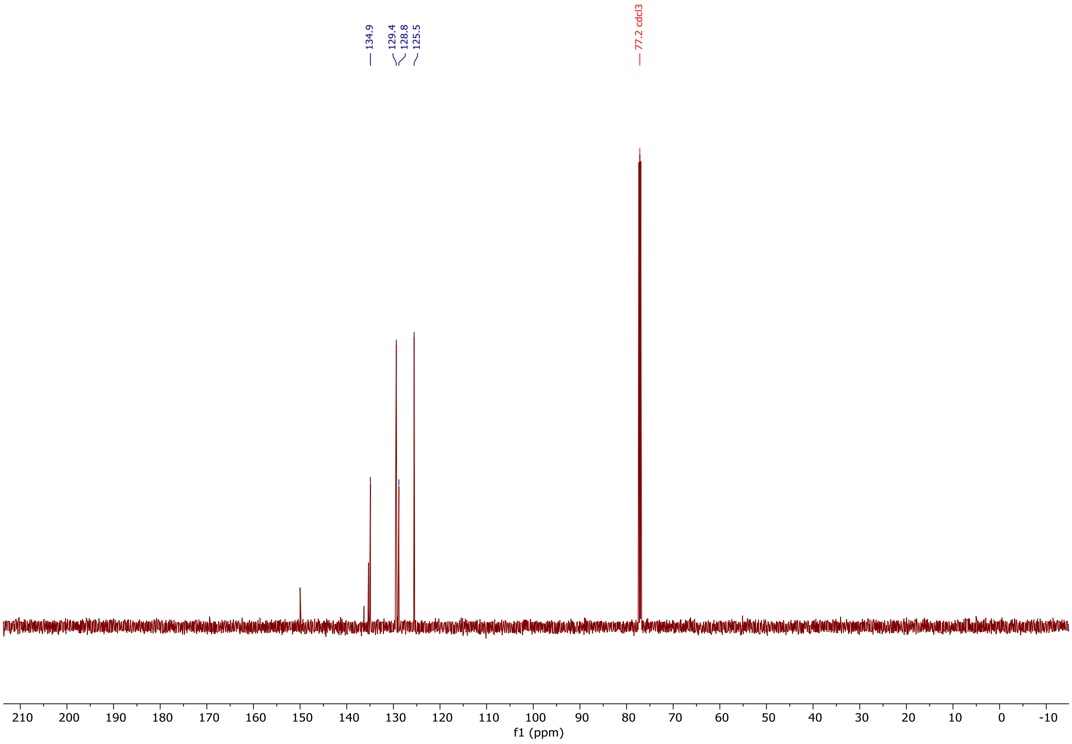

Benzenesulfonyl bromide

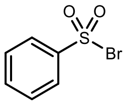

^{1}H NMR (500 MHz, $CDCl_3$)

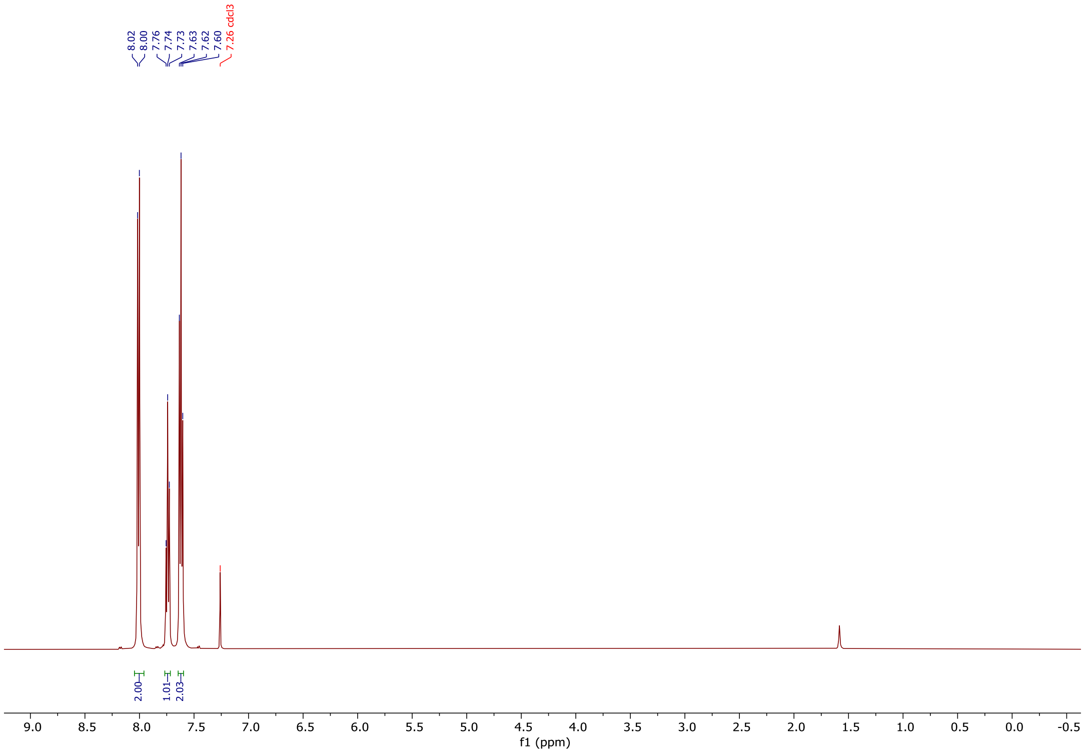

^{13}C NMR (126 MHz, $CDCl_3$)

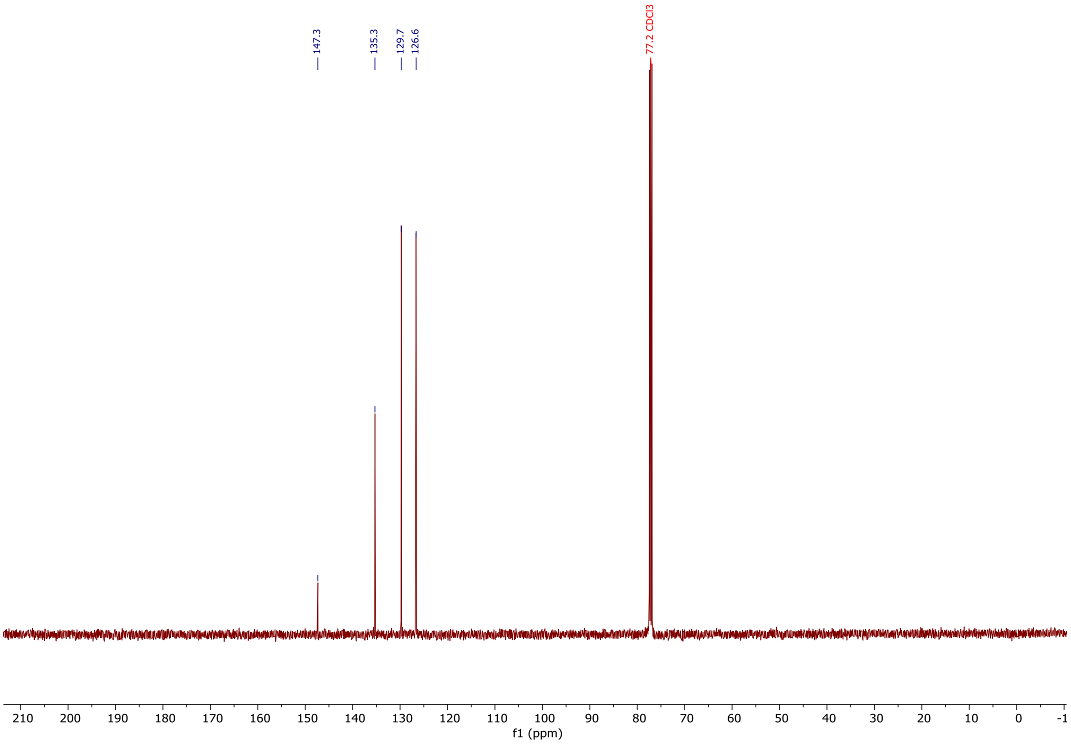

4-Methoxybenzenesulfonyl bromide

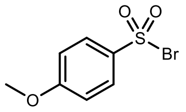

^{1}H NMR (500 MHz, $CDCl_3$)

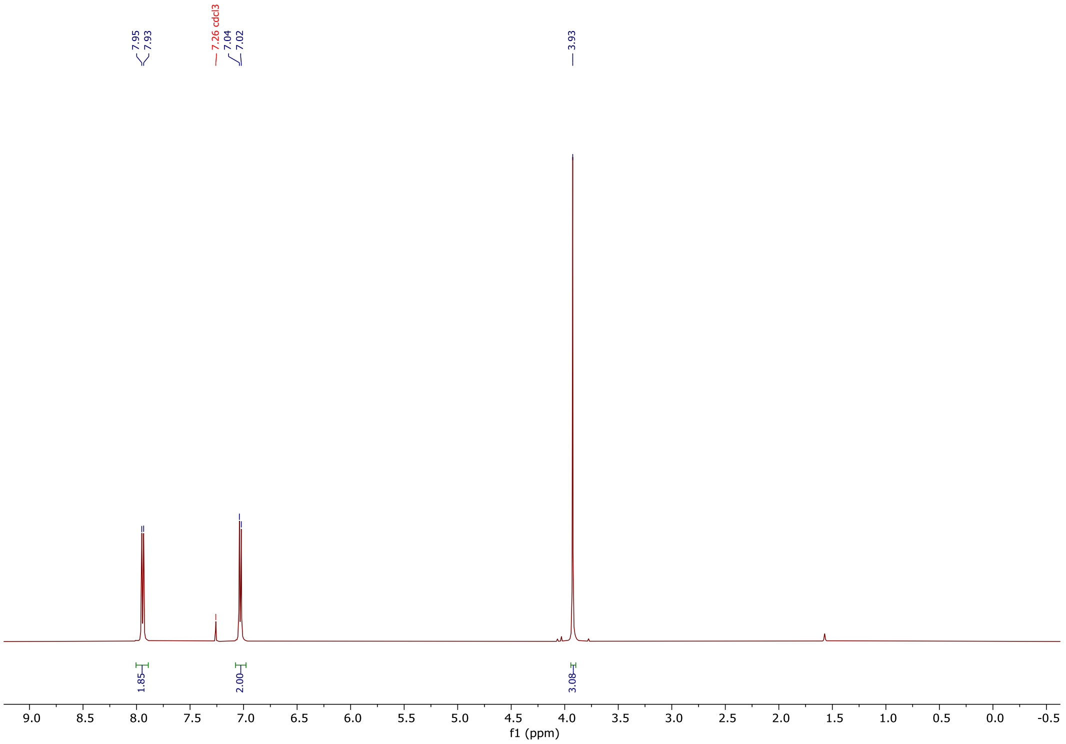

^{13}C NMR (126 MHz, $CDCl_3$)

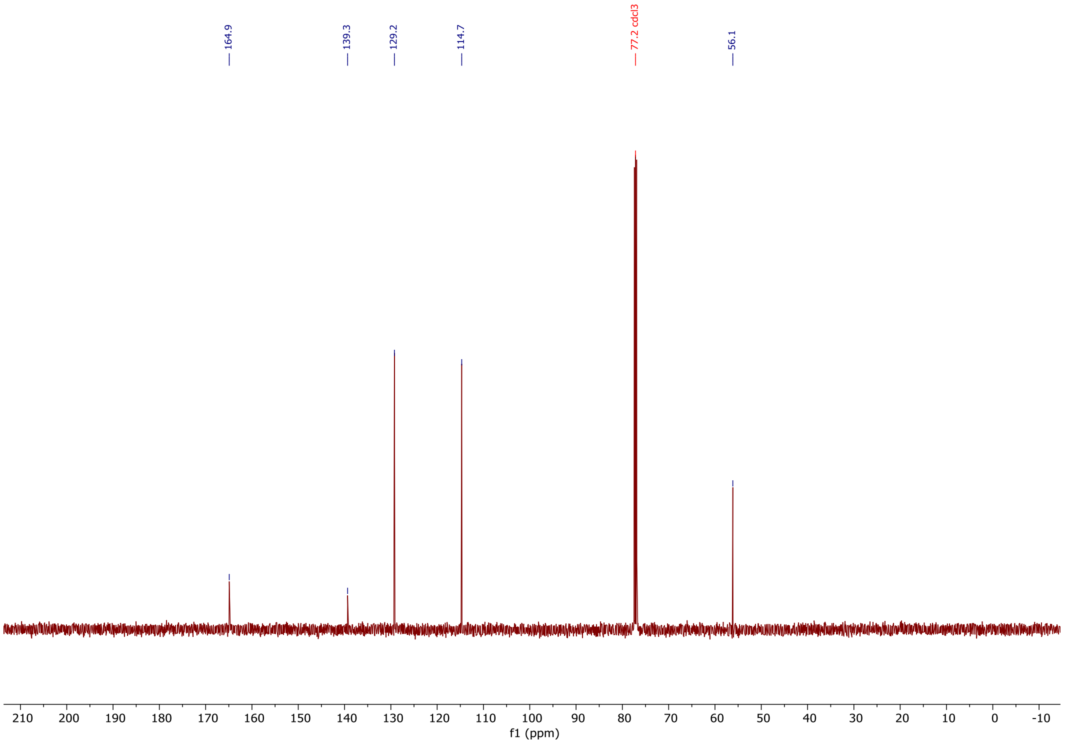

4-Fluorobenzenesulfonyl bromide

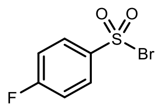

^{1}H NMR (500 MHz, $CDCl_3$)

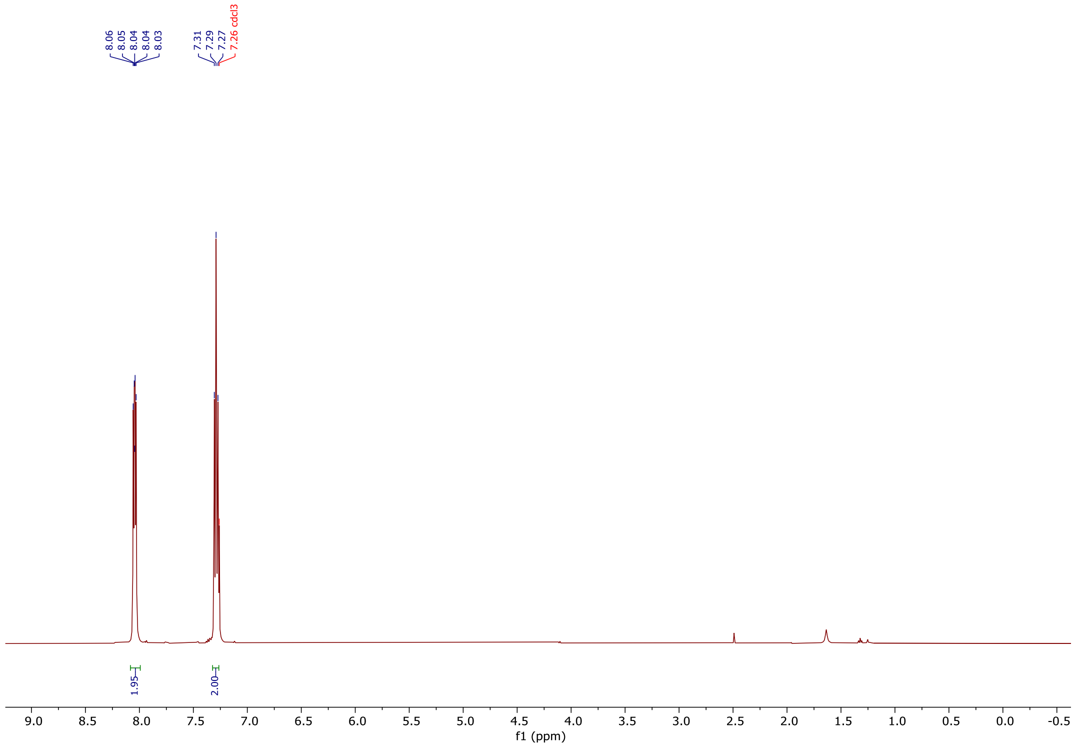

^{13}C NMR (126 MHz, $CDCl_3$)

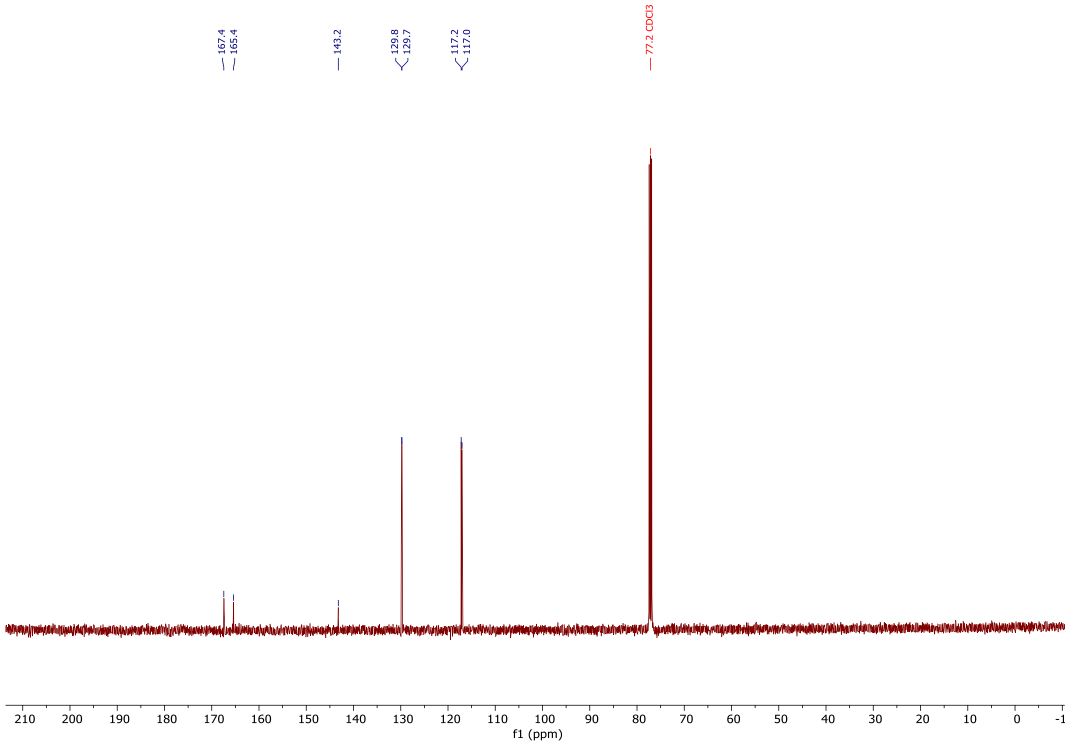

^{19}F {^{1}H} NMR (376 MHz, $CDCl_3$)

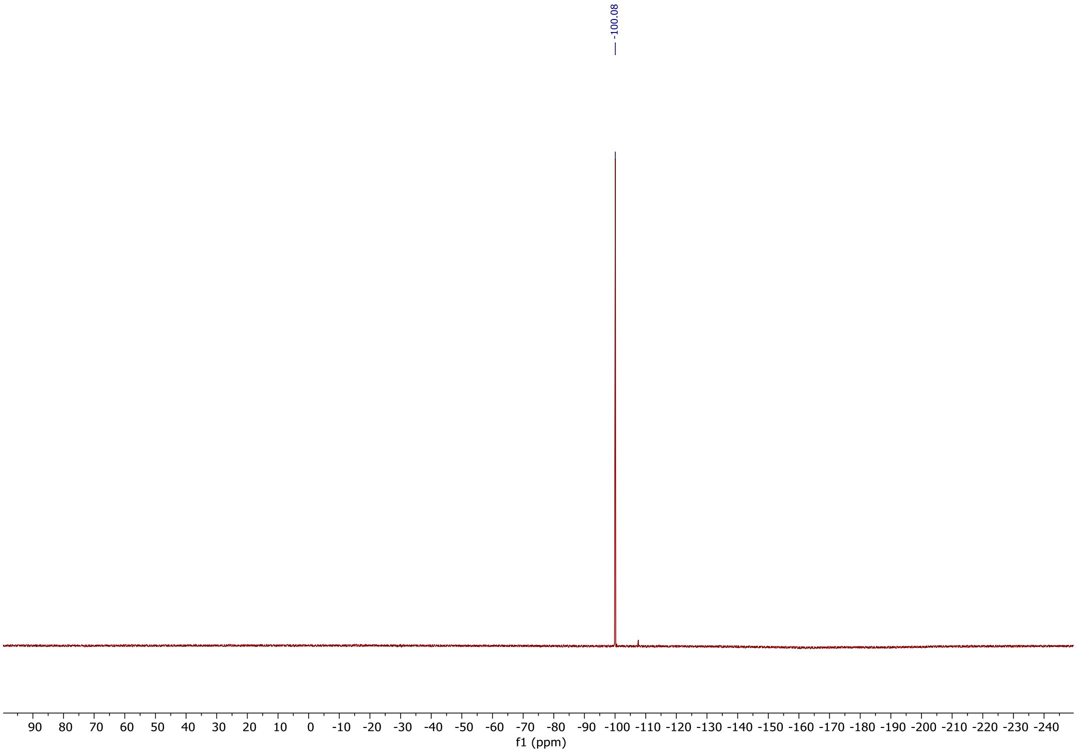

3-Fluorobenzenesulfonyl bromide

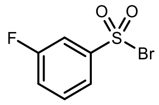

^{1}H NMR (500 MHz, $CDCl_3$)

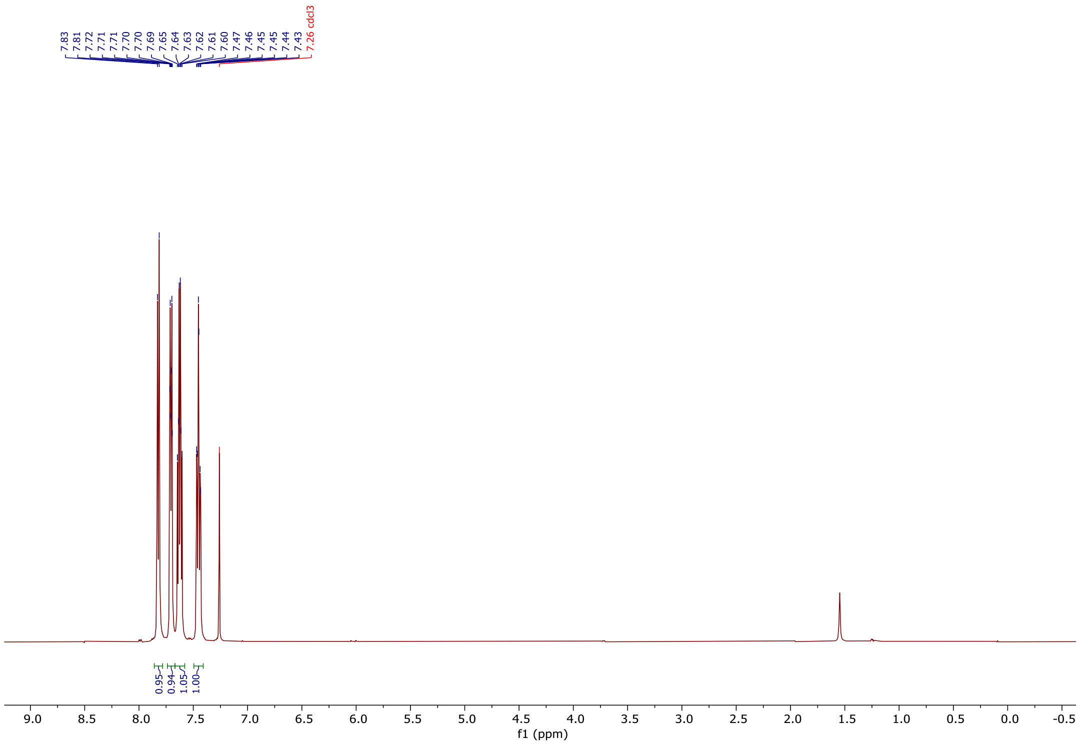

^{13}C NMR (126 MHz, $CDCl_3$)

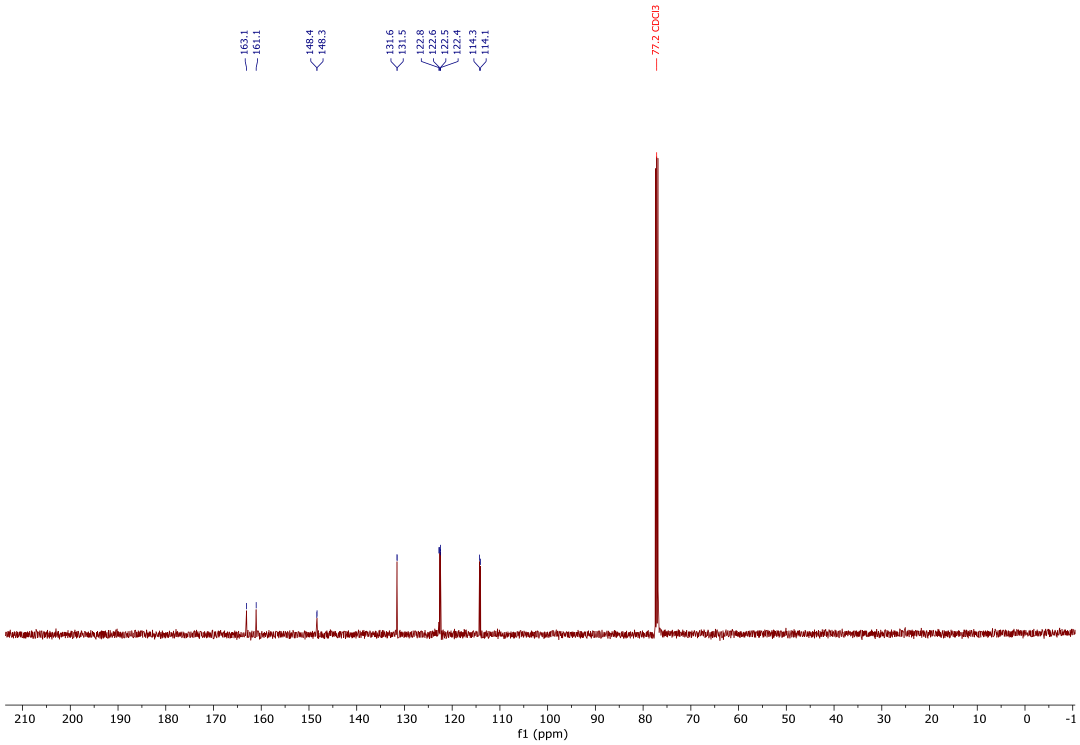

^{19}F {^{1}H} NMR (376 MHz, $CDCl_3$)

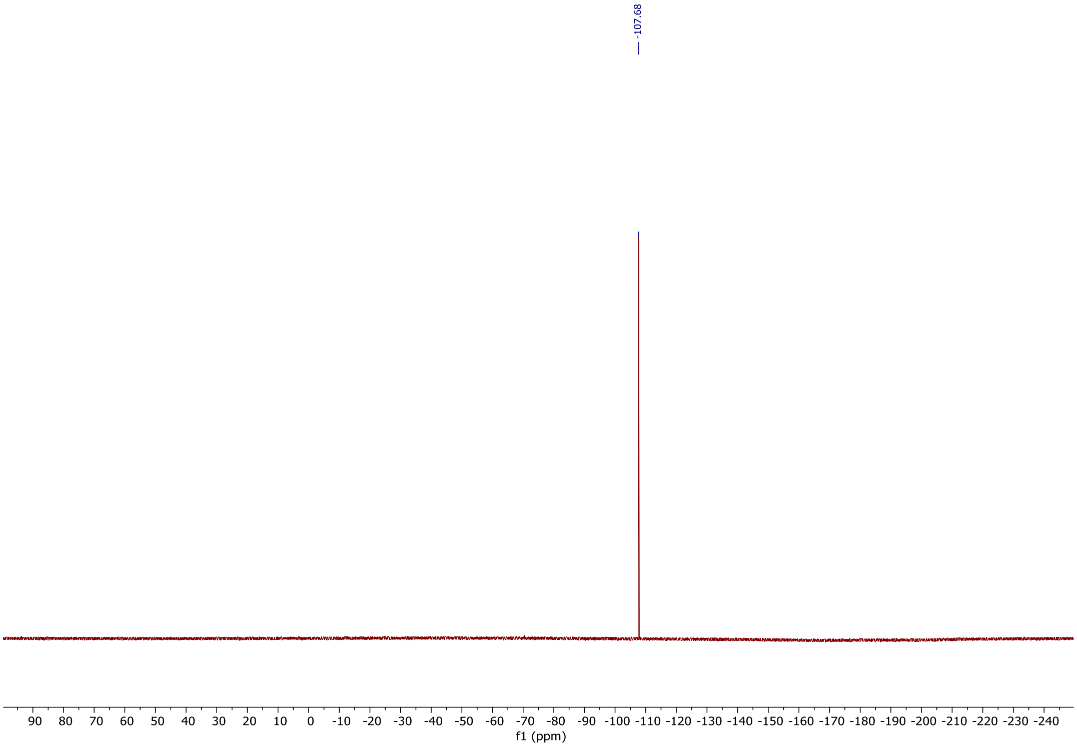

4-(Trifluoromethyl)benzenesulfonyl bromide

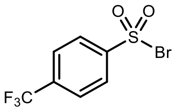

^{1}H NMR (500 MHz, $CDCl_3$)

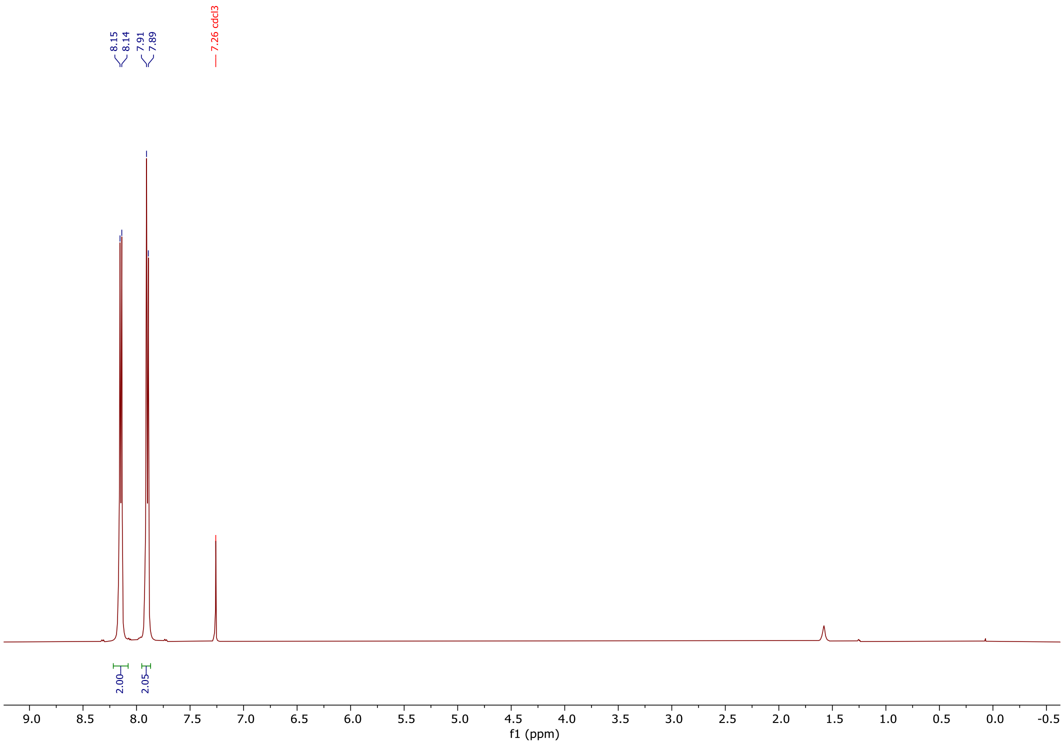

^{13}C NMR (151 MHz, $CDCl_3$)

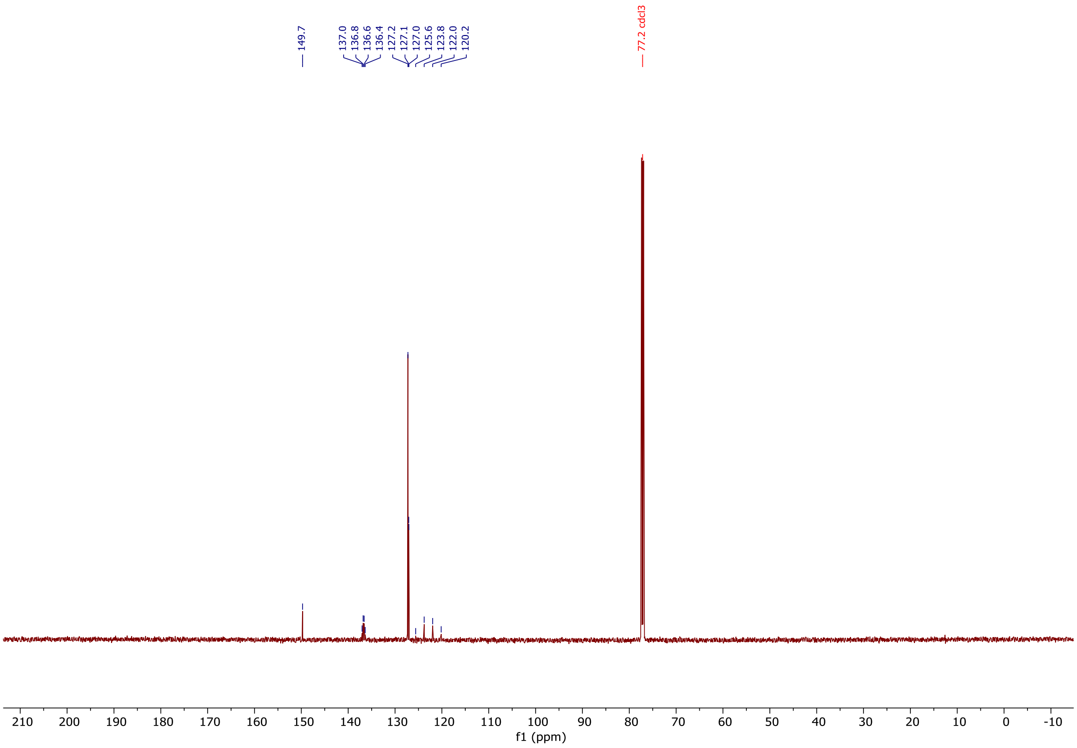

$^{19}F\ \{^{1}H\}$ NMR (376 MHz, $CDCl_3$)

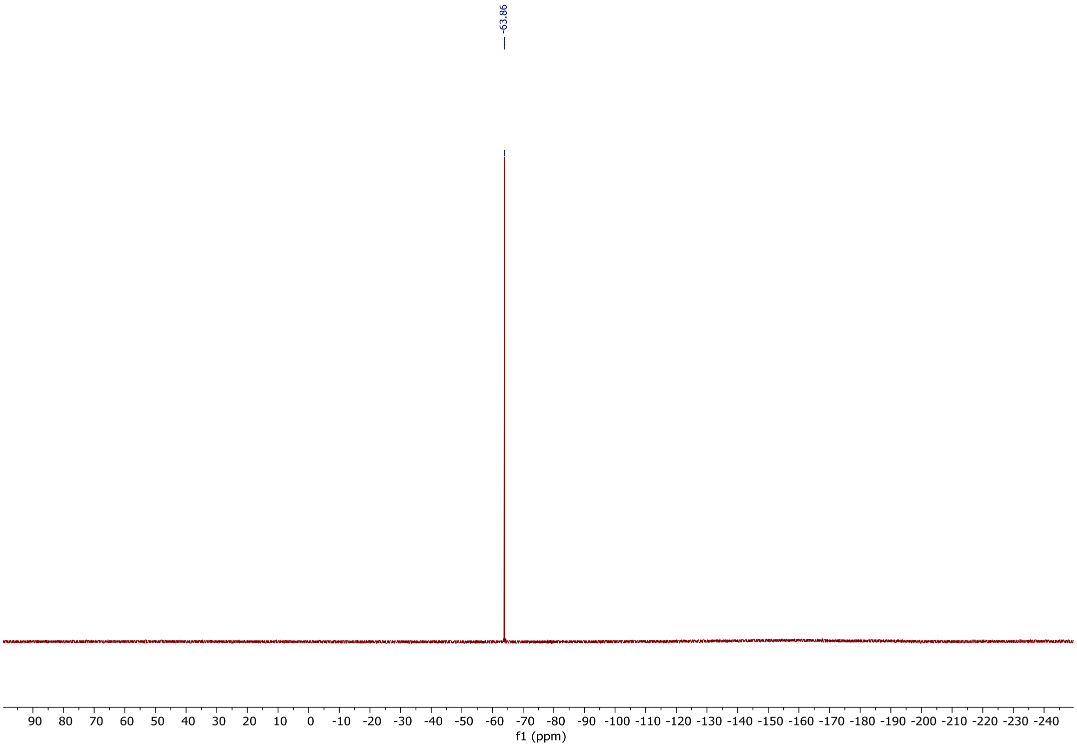

Thiophene-2-sulfonohydrazide

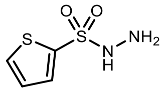

^{1}H NMR (500 MHz, $CDCl_3$)

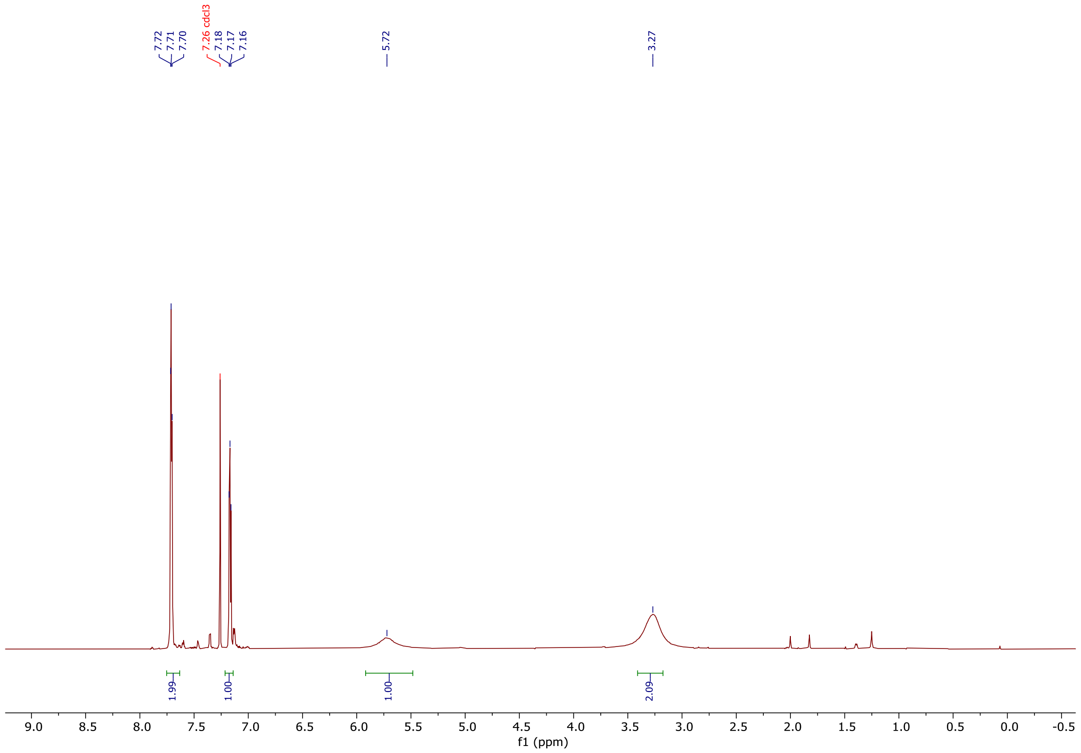

^{13}C NMR (126 MHz, $CDCl_3$)

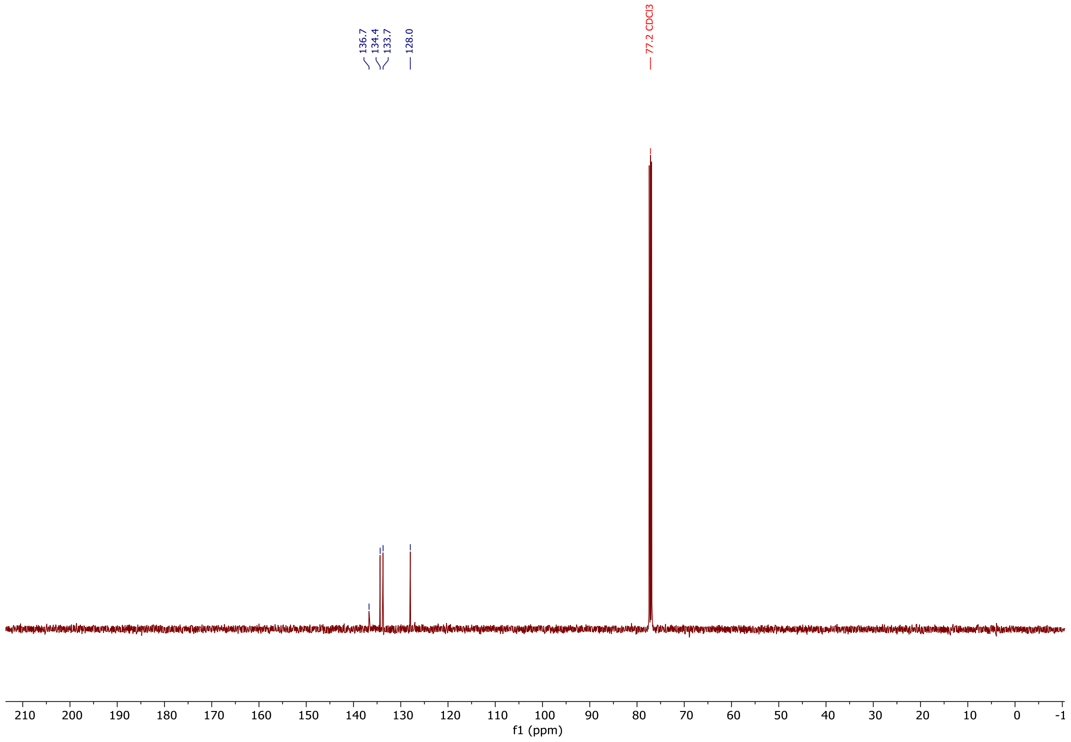

Thiophene-2-sulfonyl bromide

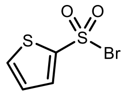

^{1}H NMR (500 MHz, CDCl$_3$)

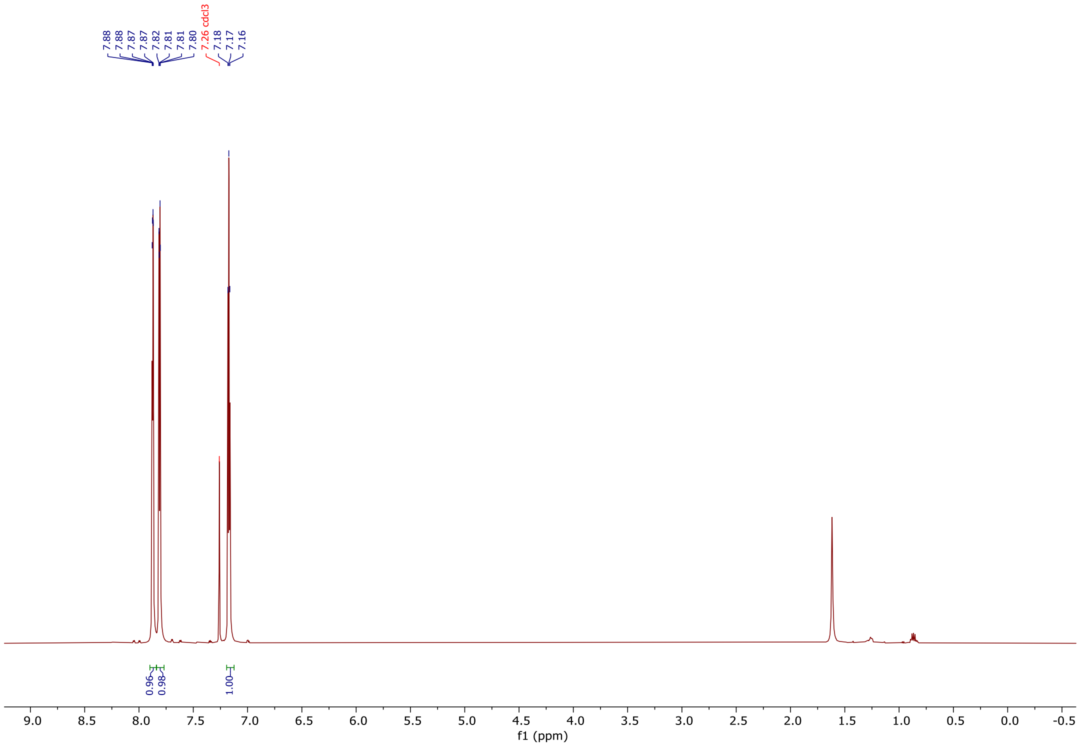

^{13}C NMR (126 MHz, CDCl$_3$)

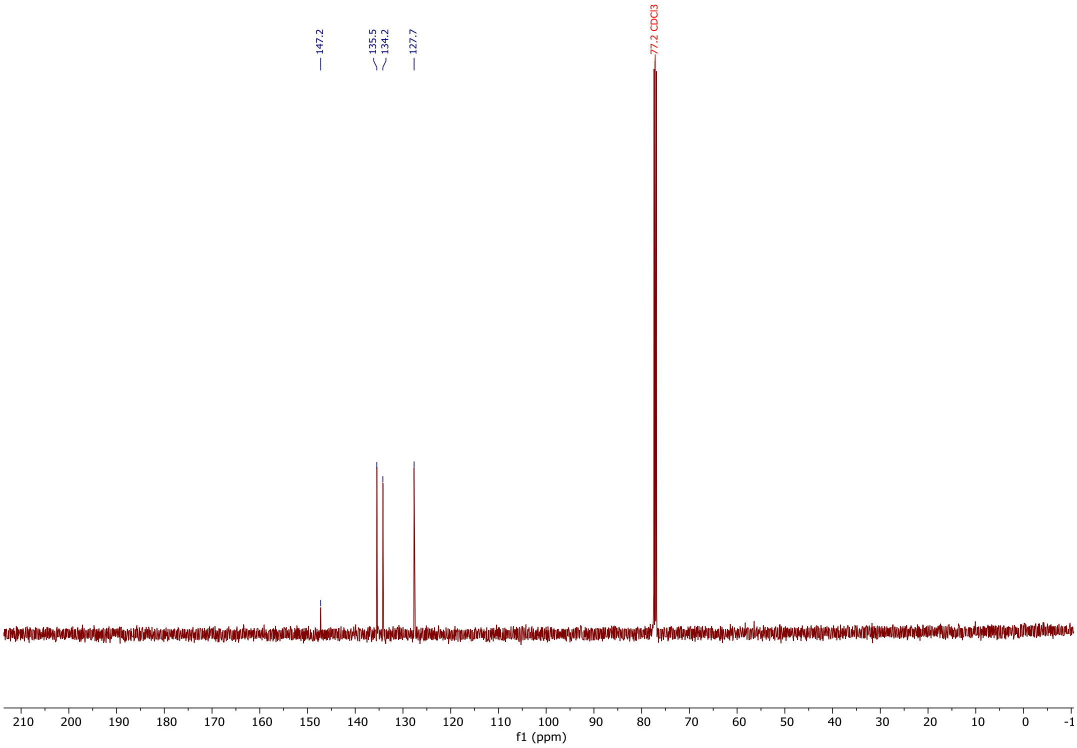

Methanesulfonyl bromide

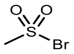

^{1}H NMR (500 MHz, CDCl$_3$)

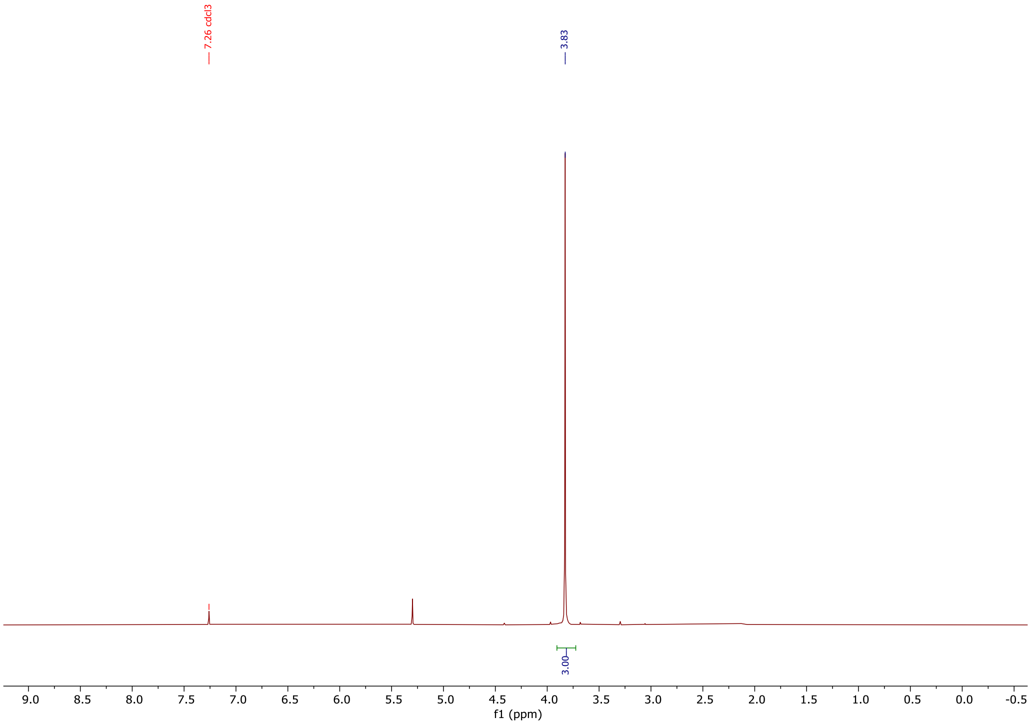

^{13}C NMR (126 MHz, CDCl$_3$)

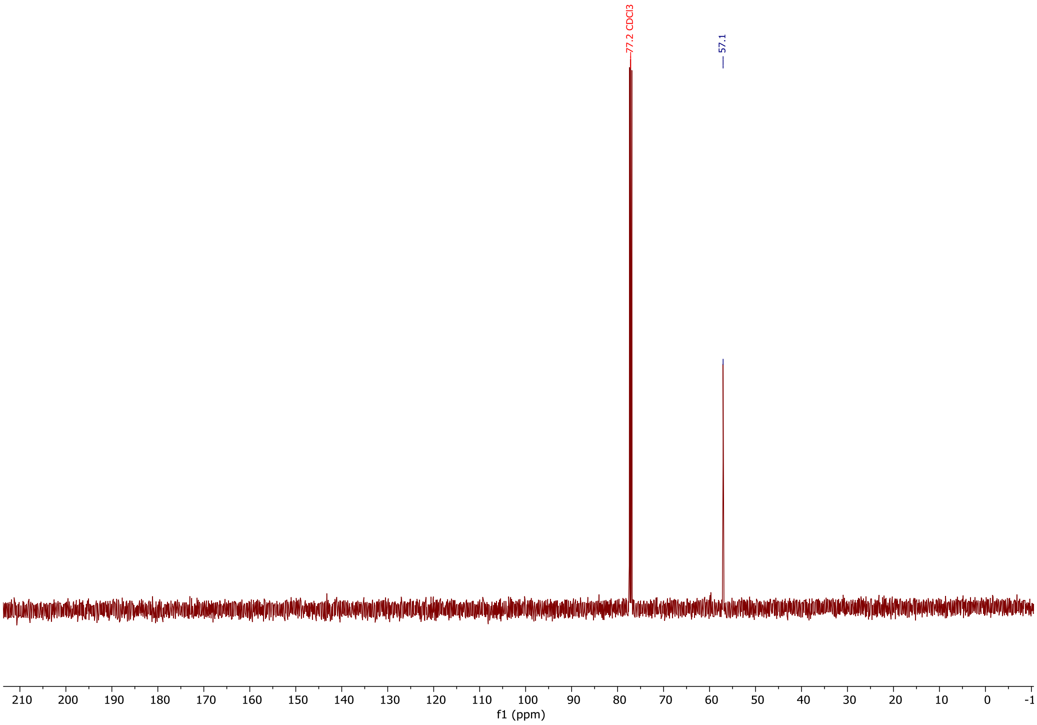

Ethanesulfonyl bromide

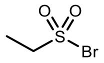

^{1}H NMR (500 MHz, $CDCl_3$)

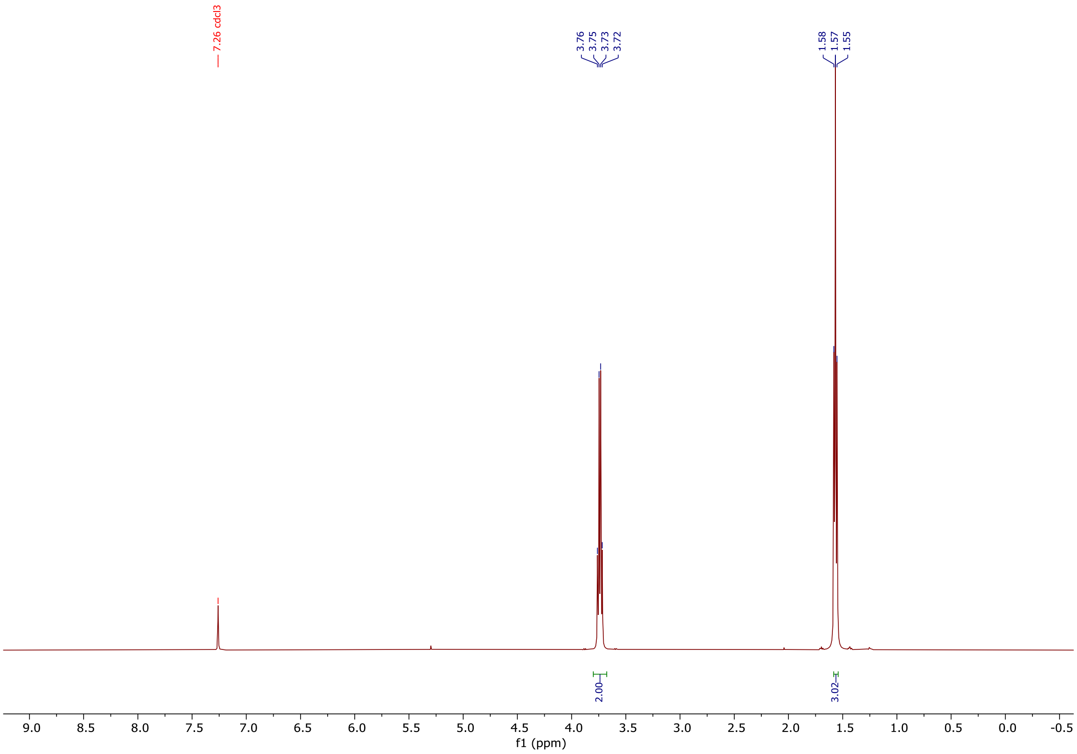

^{13}C NMR (151 MHz, $CDCl_3$)

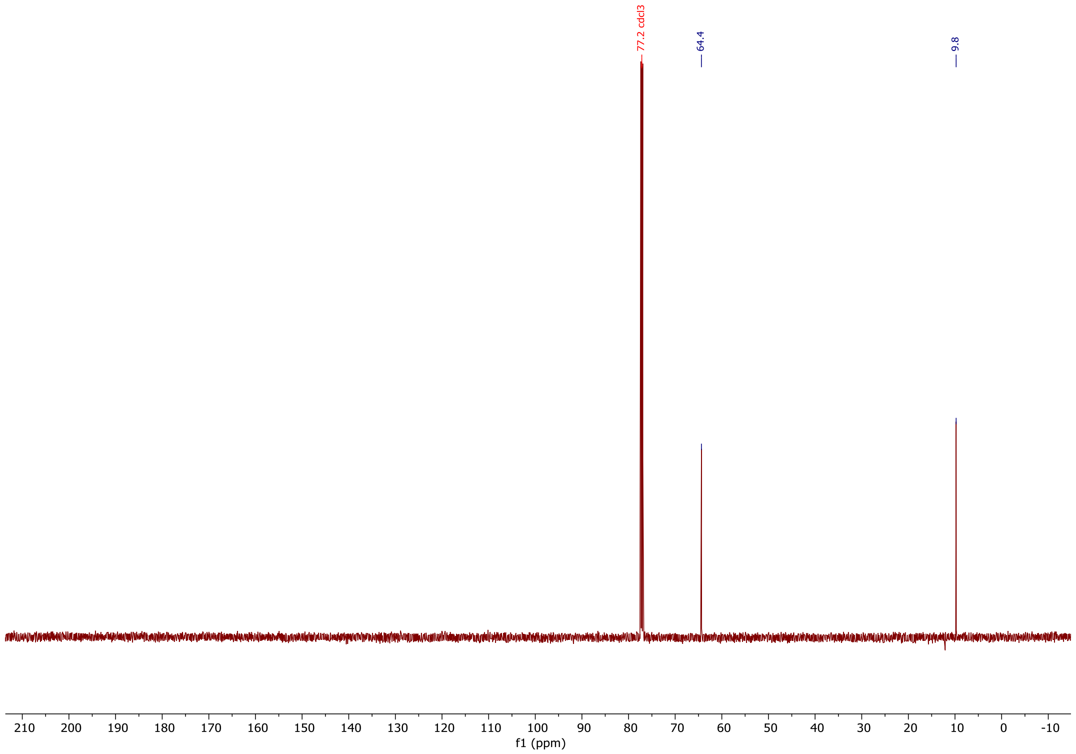

Propane-2-sulfonyl bromide

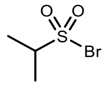

^{1}**H NMR** (500 MHz, $CDCl_3$)

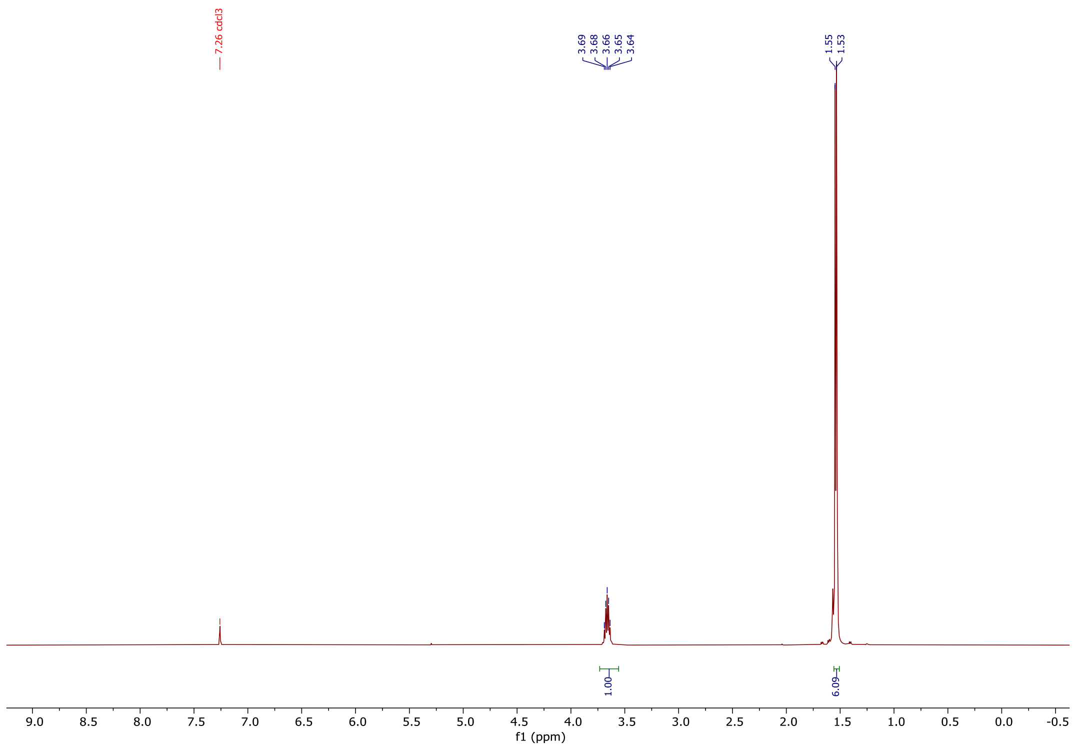

^{13}**C NMR** (126 MHz, $CDCl_3$)

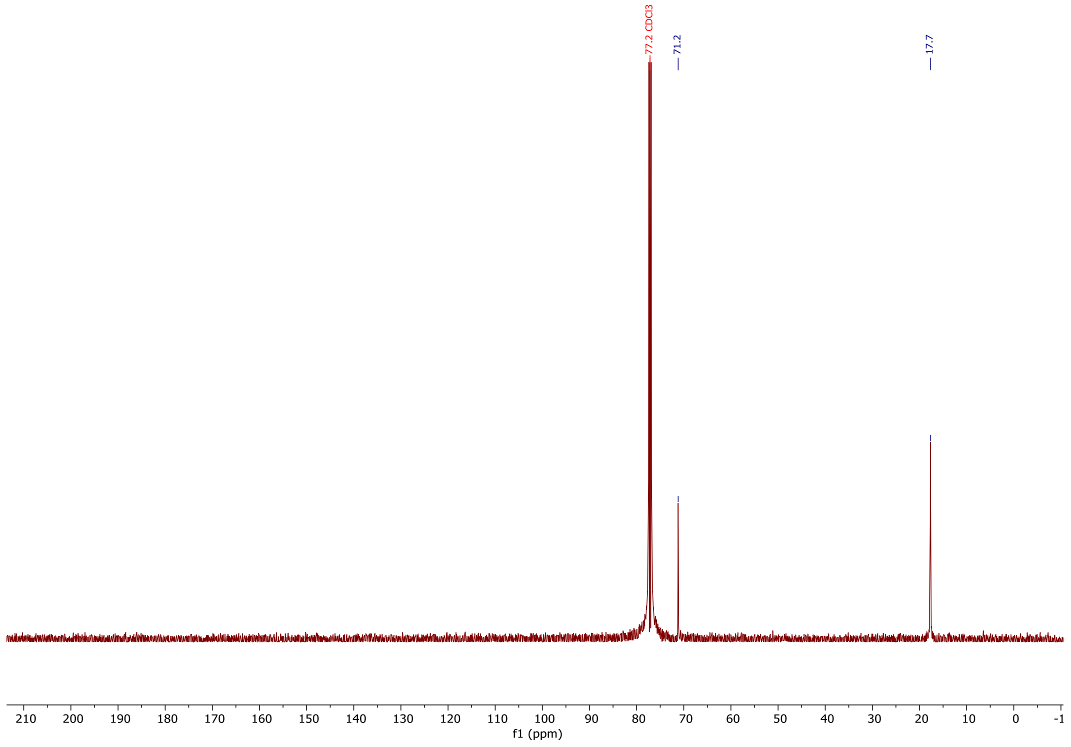

Cyclopropanesulfonyl bromide

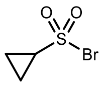

^{1}H NMR (500 MHz, CDCl$_3$)

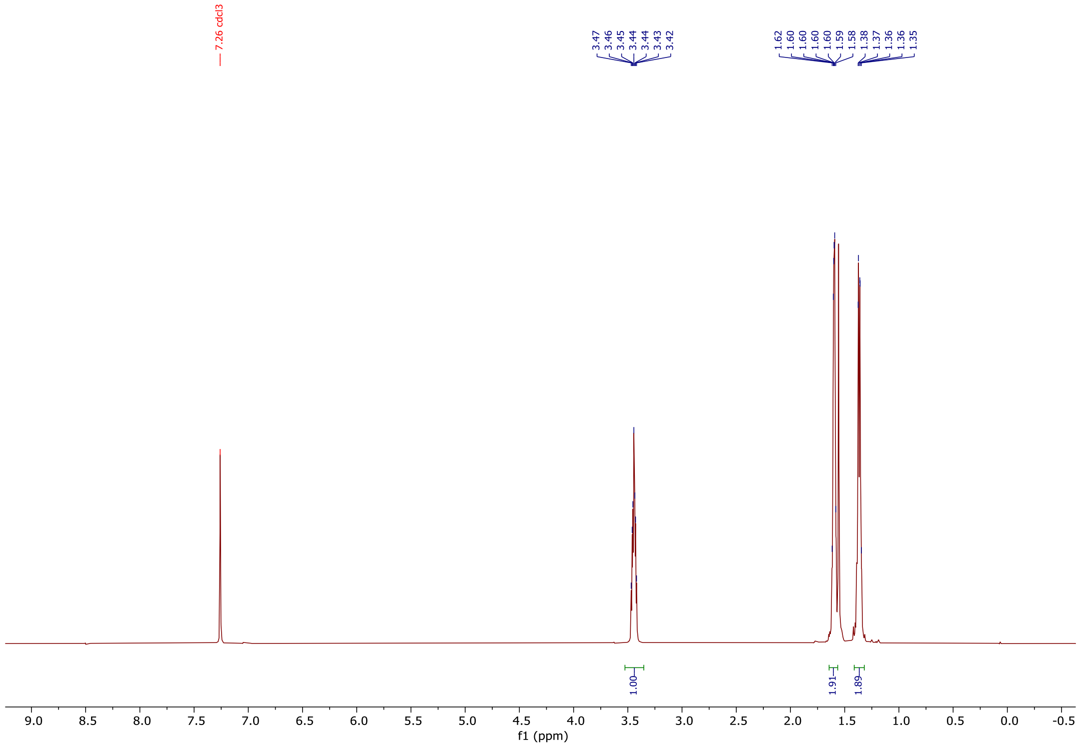

^{13}C NMR (126 MHz, CDCl$_3$)

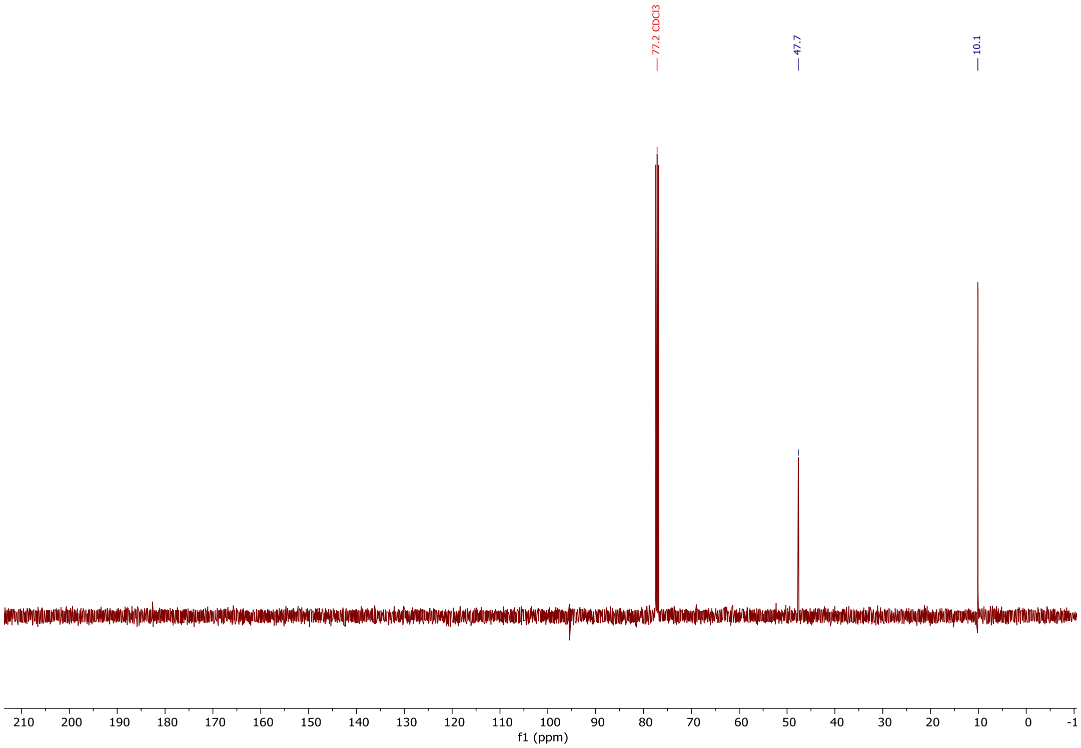

6.7 Sulfinate Salts

Sodium 4-methoxybenzenesulfinate, 2c

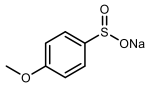

^{1}H NMR (400 MHz, MeOD)

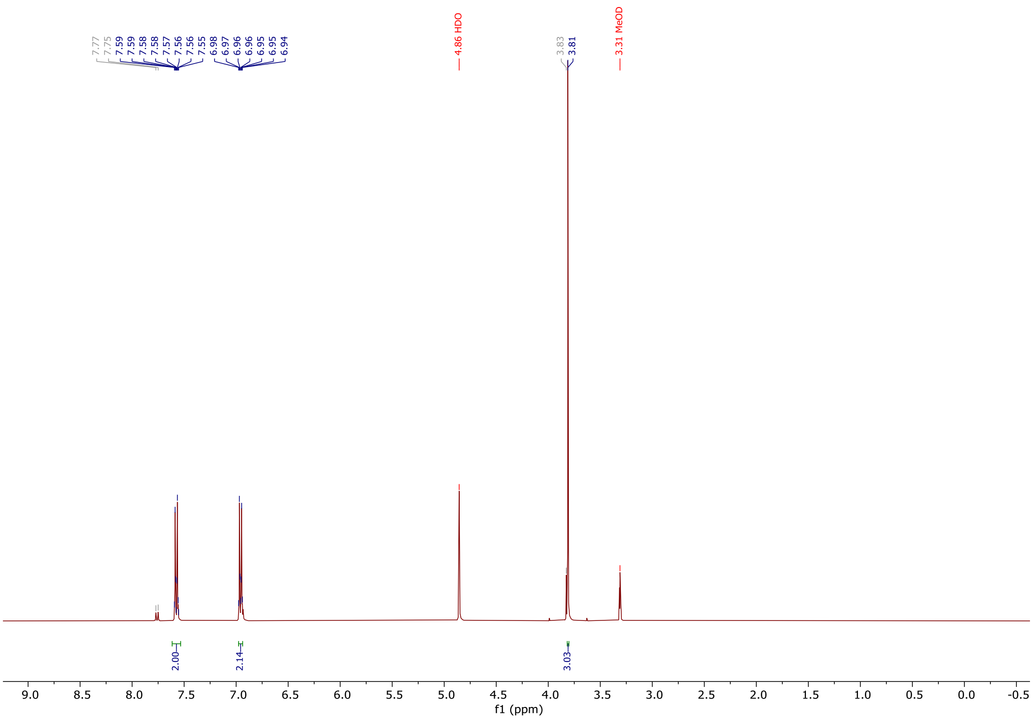

^{1}H NMR (400 MHz, D_2O)

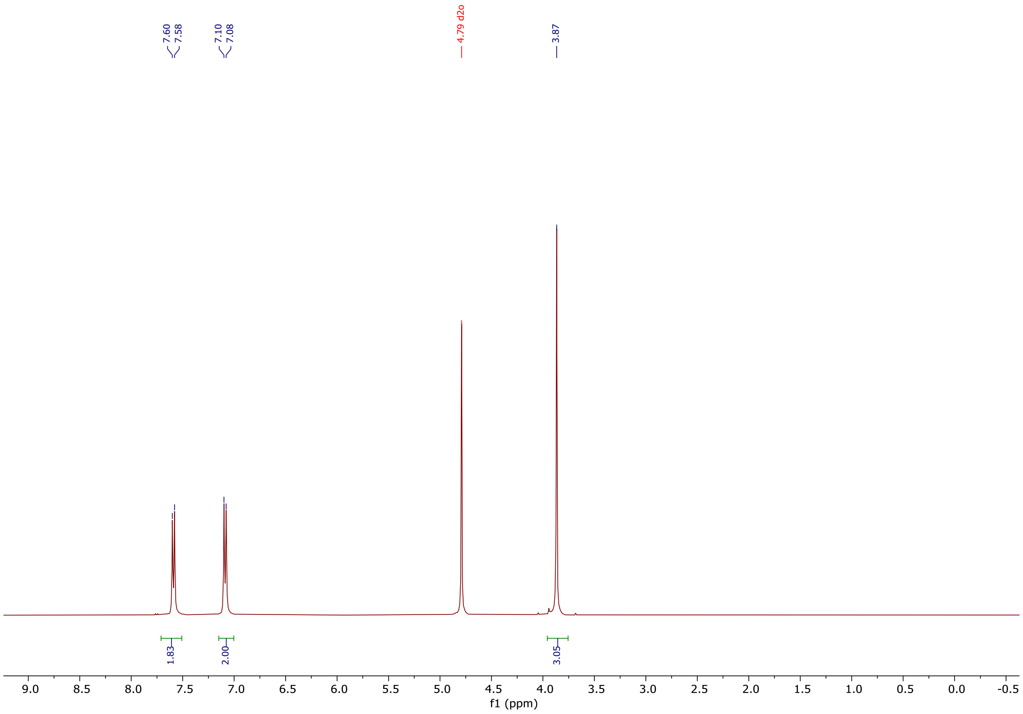

^{13}C NMR (101 MHz, MeOD)

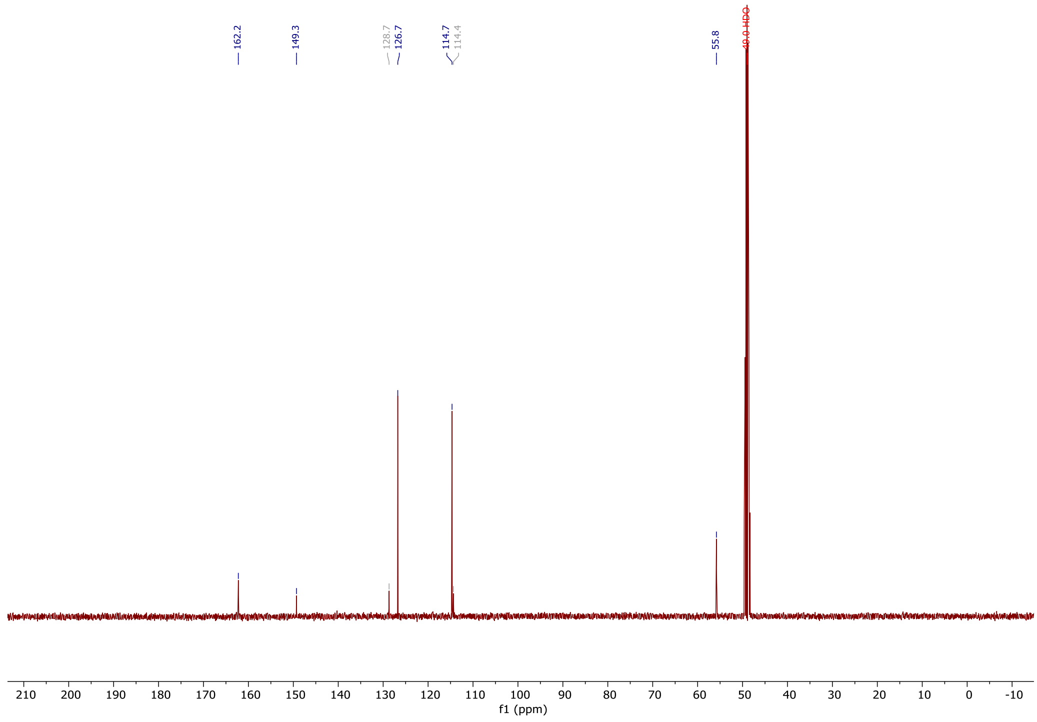

^{13}C NMR (126 MHz, D_2O)

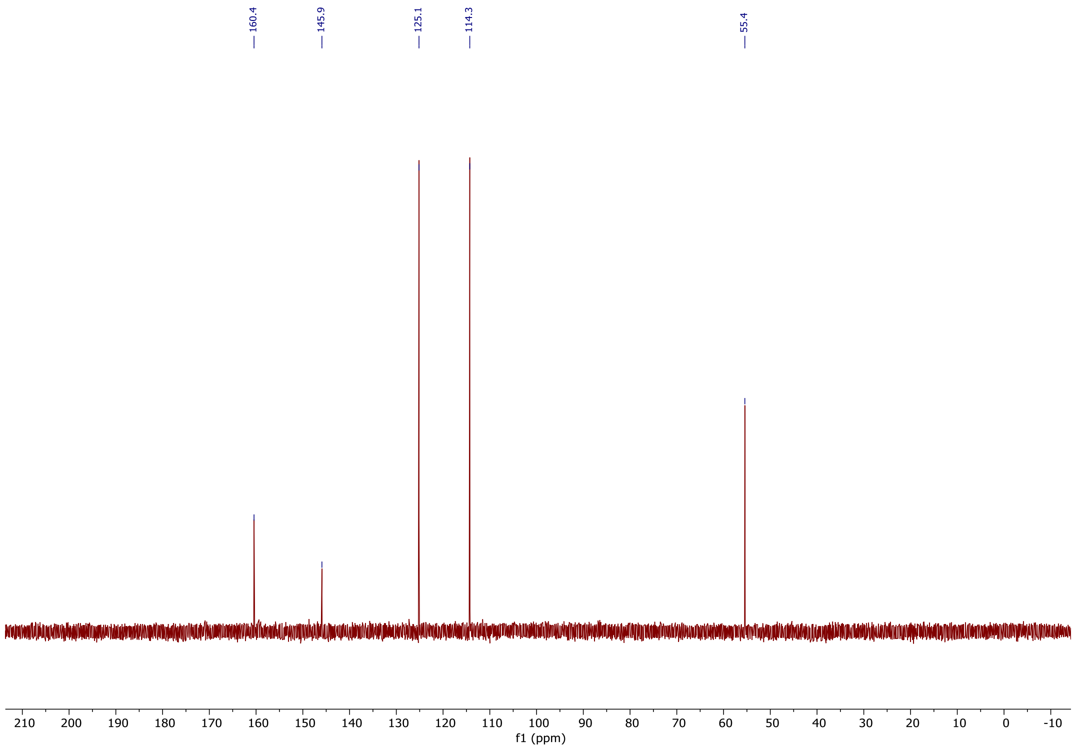

Sodium 4-bromobenzenesulfinate, 2d

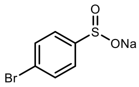

^{1}H NMR (400 MHz, MeOD)

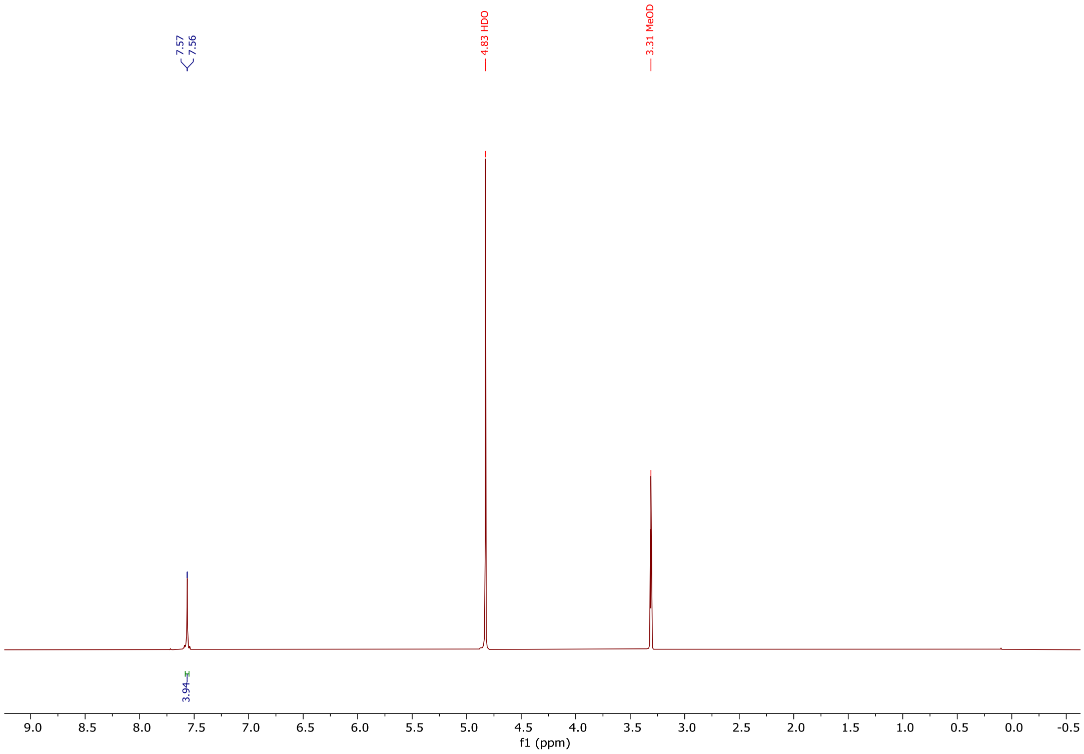

^{13}C NMR (101 MHz, MeOD)

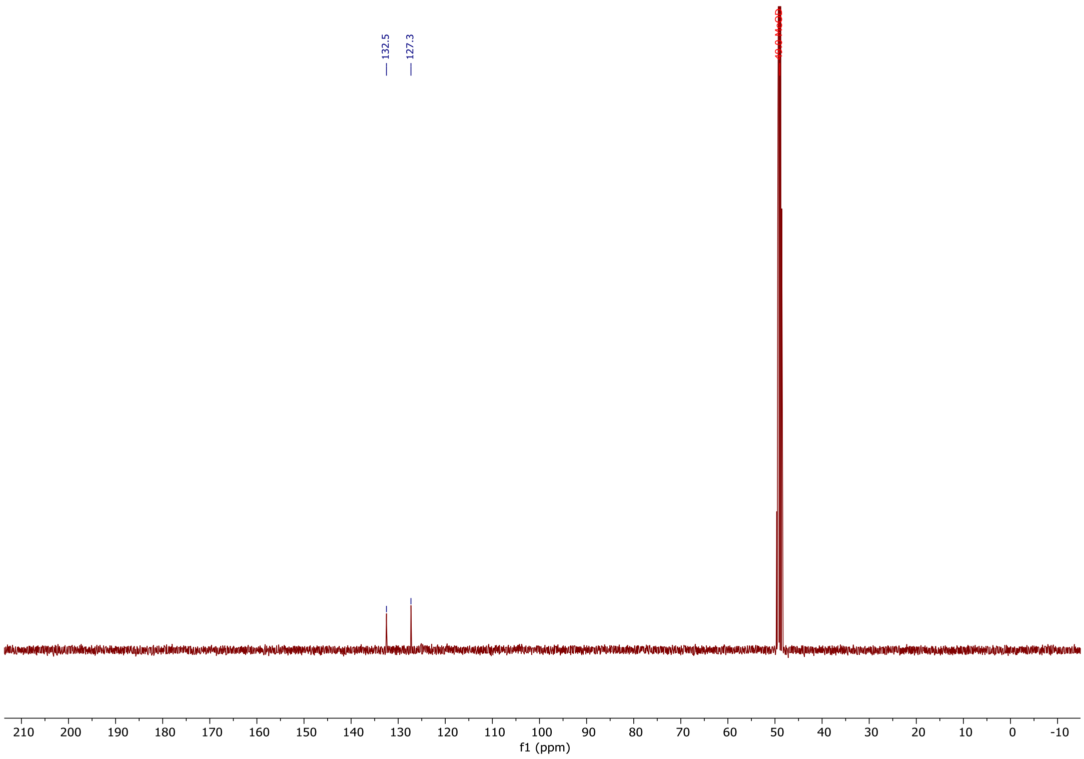

Sodium 4-fluorobenzenesulfinate, 2e

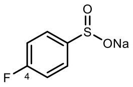

^{1}H NMR (400 MHz, DMSO-d$_6$)

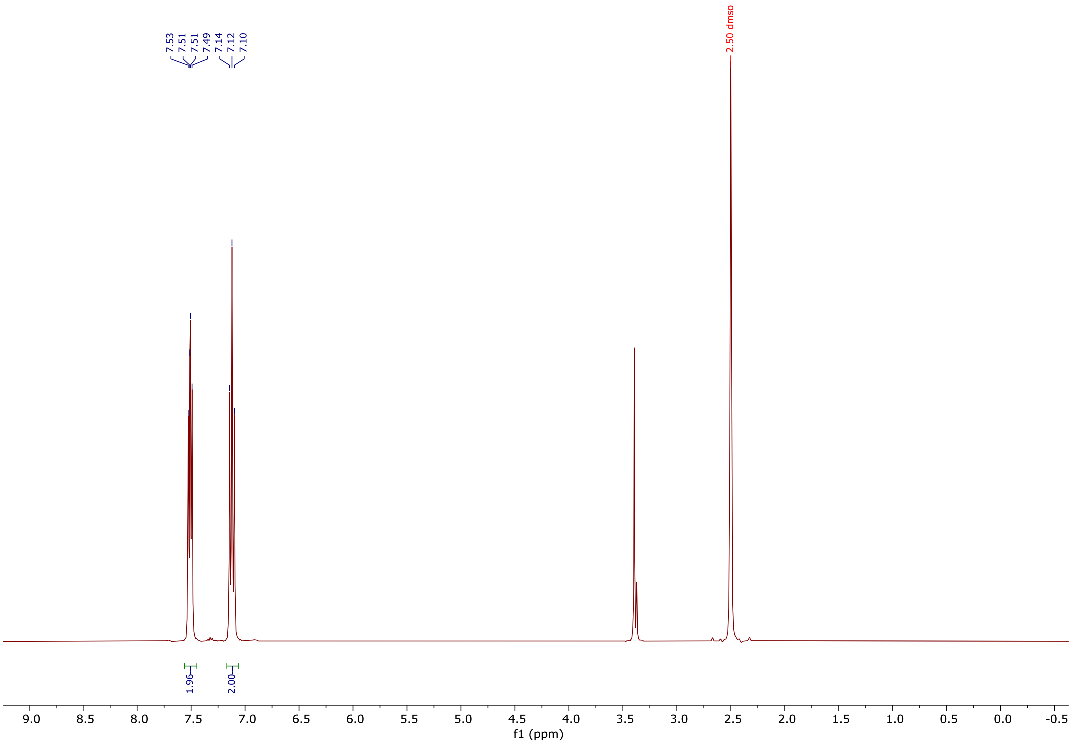

^{13}C NMR (151 MHz, DMSO-d$_6$)

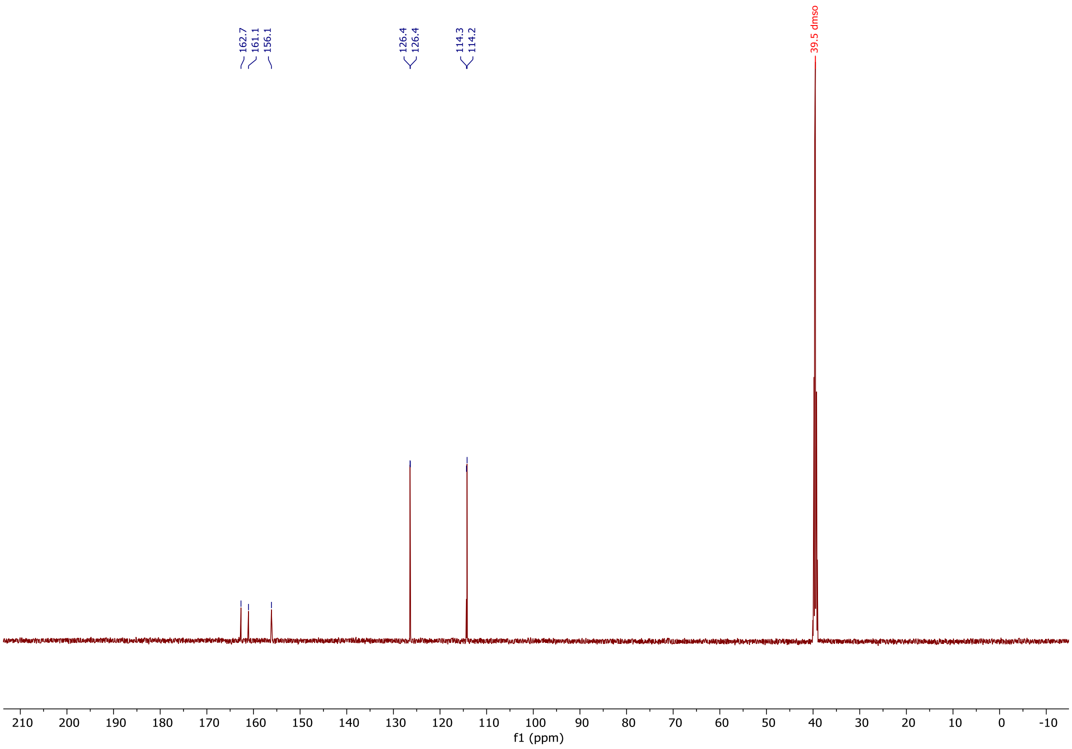

$^{19}F\{^1H\}$ **NMR** (376 MHz, DMSO-d$_6$)

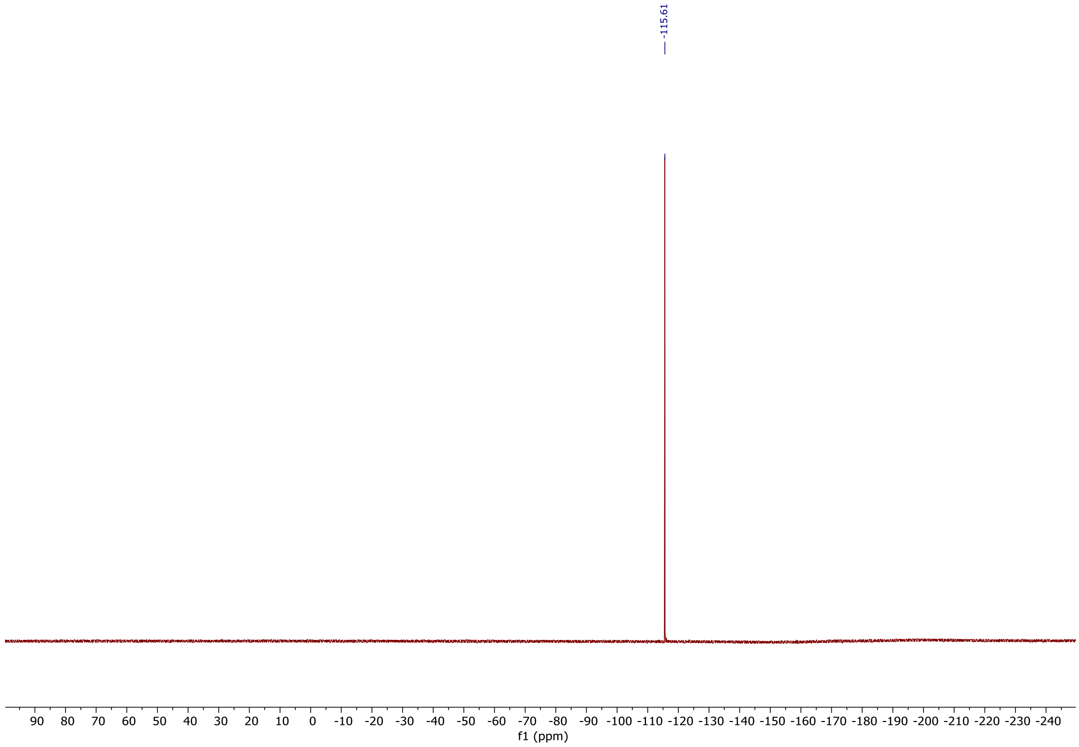

Sodium 3-fluorobenzenesulfinate, 2f

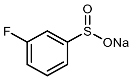

^{1}H NMR (400 MHz, D_2O)

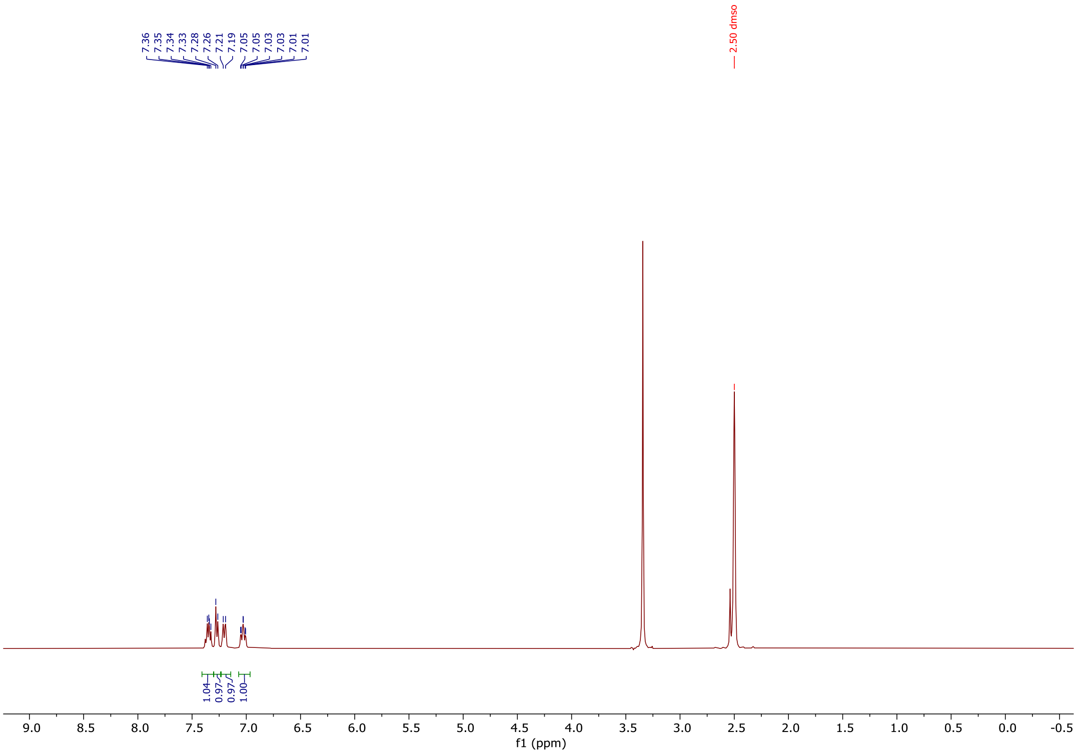

^{13}C NMR (151 MHz, DMSO-d_6)

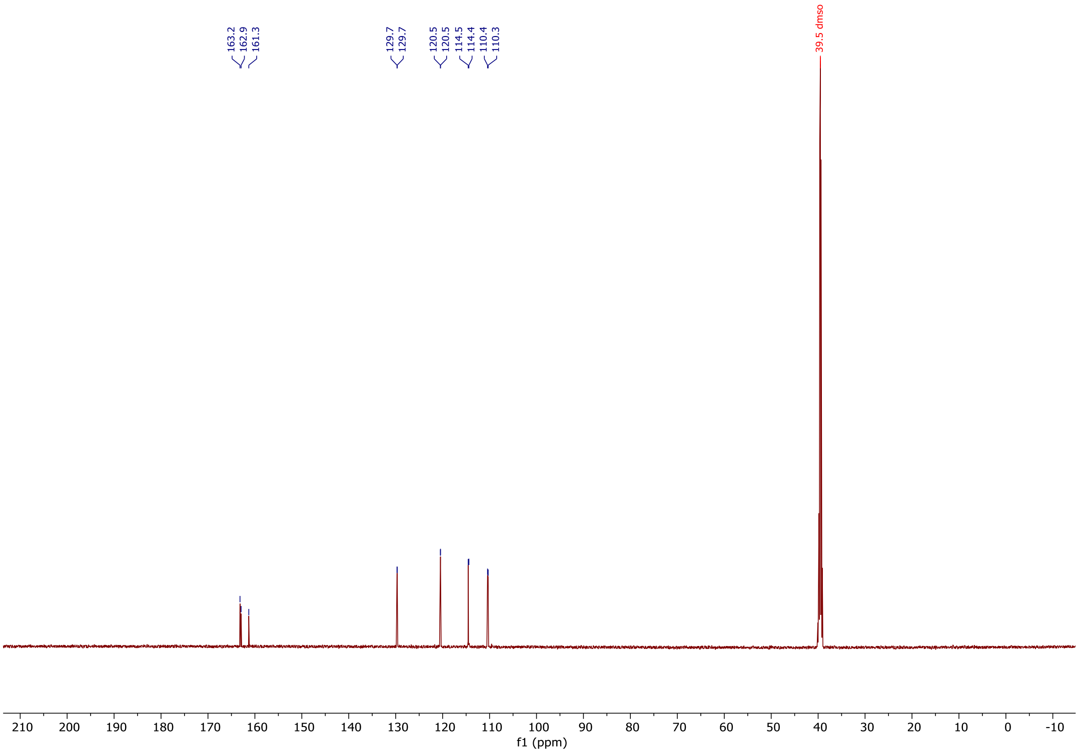

$^{19}F\{^{1}H\}$ **NMR** (376 MHz, DMSO-d_6)

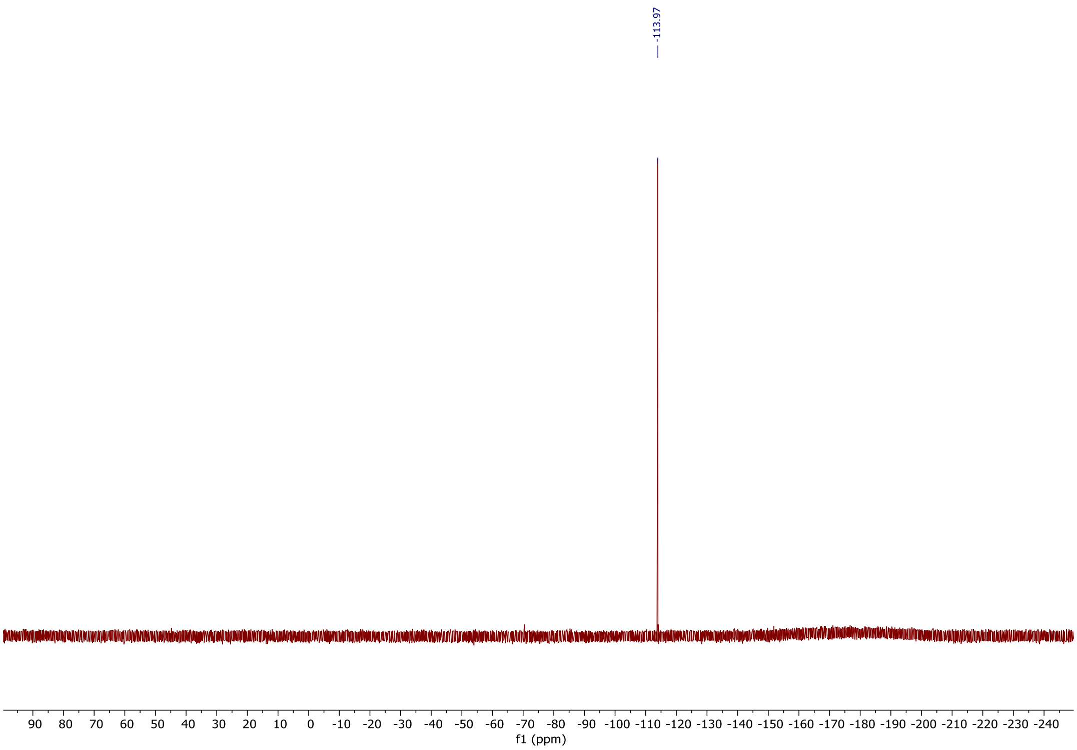

Sodium 4-nitrobenzenesulfinate, 2h

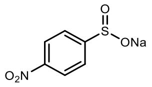

^{1}H NMR (400 MHz, MeOD)

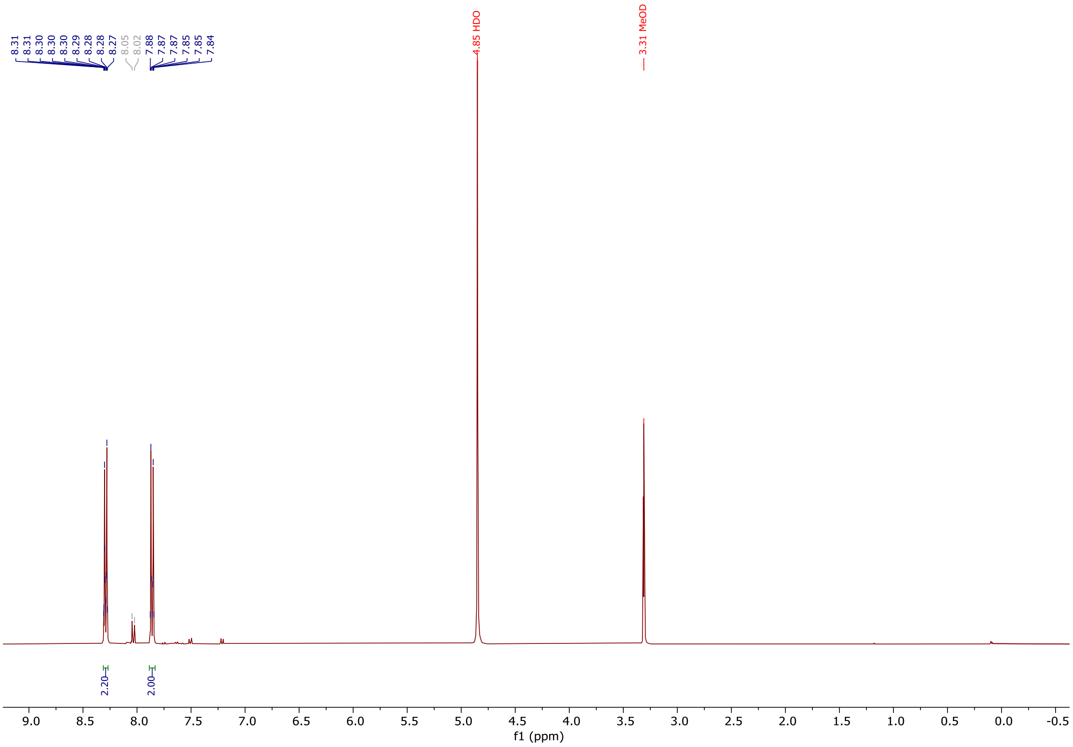

^{13}C NMR (101 MHz, MeOD)

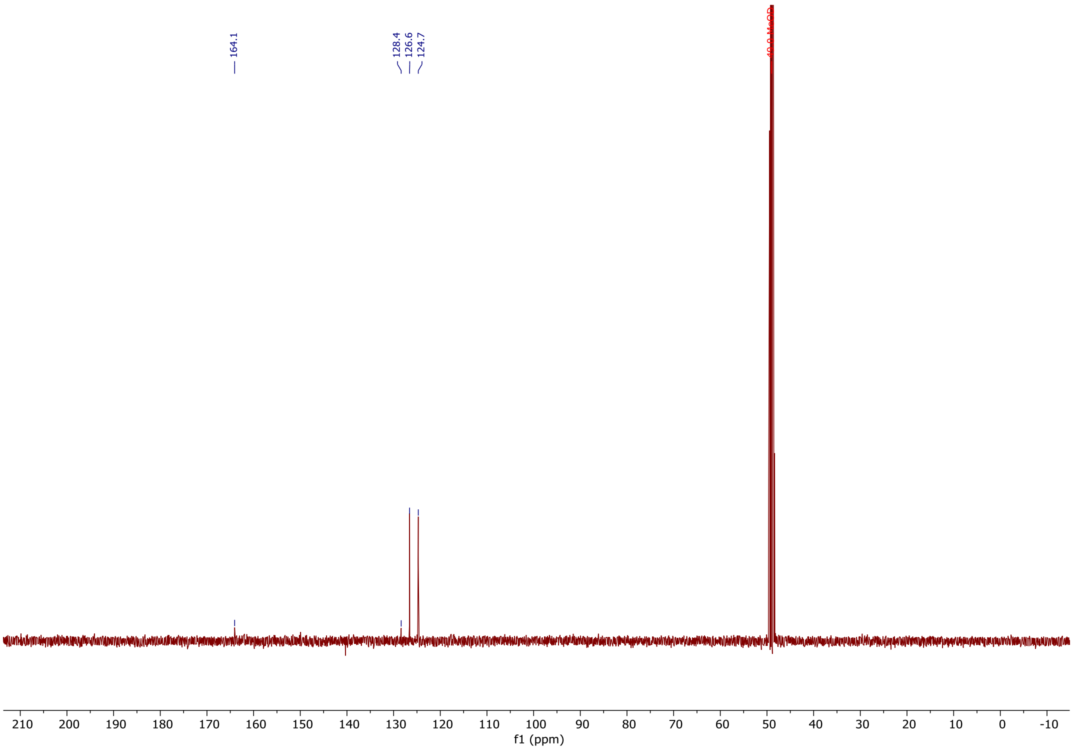

Sodium 4-(trifluoromethyl)benzenesulfinate, 2i

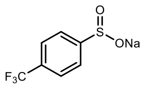

^{1}H NMR (500 MHz, D_2O)

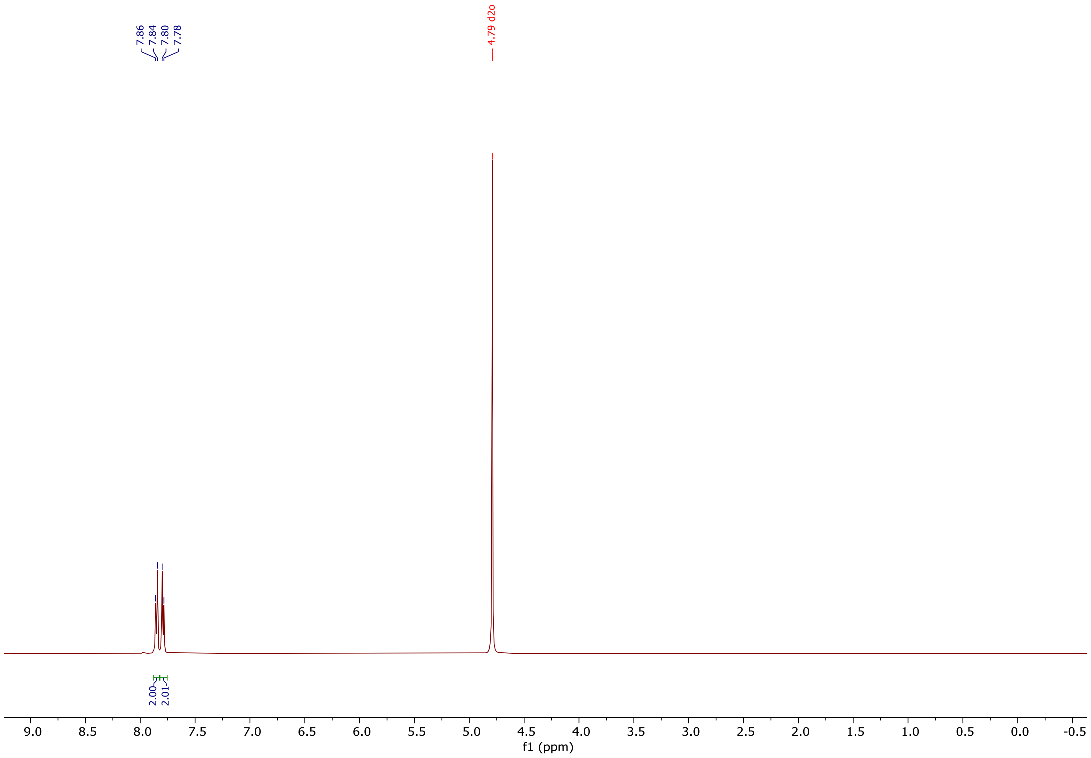

^{13}C NMR (126 MHz, D_2O)

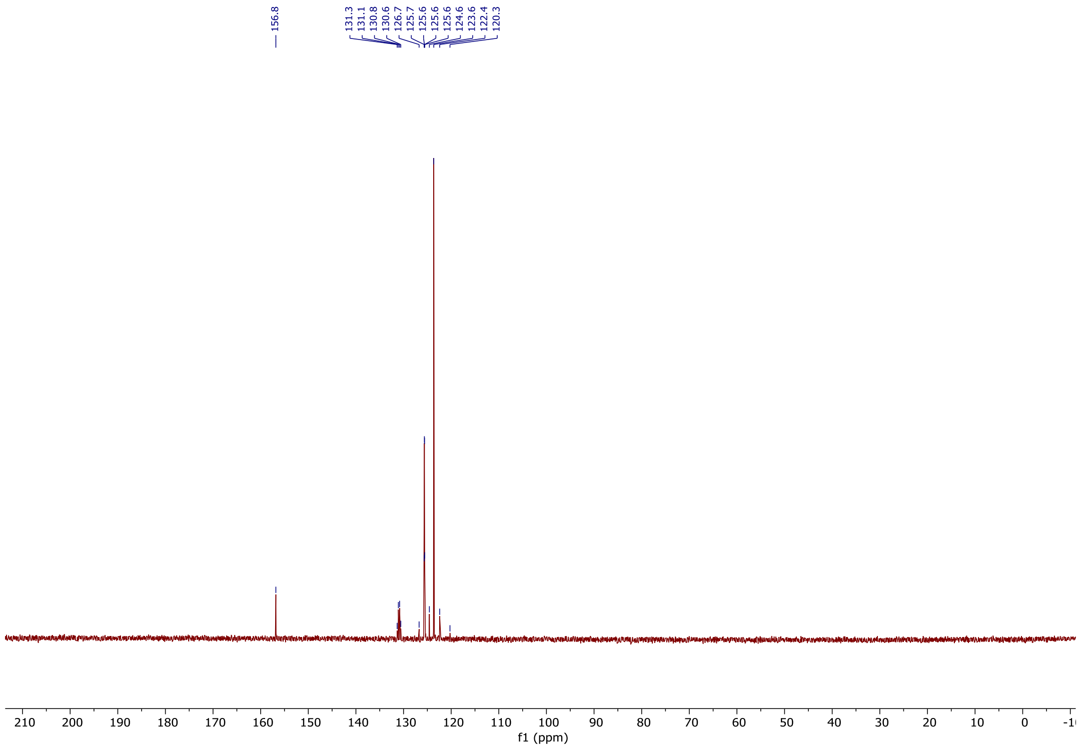

^{19}F {^{1}H} NMR (376 MHz, D_2O)

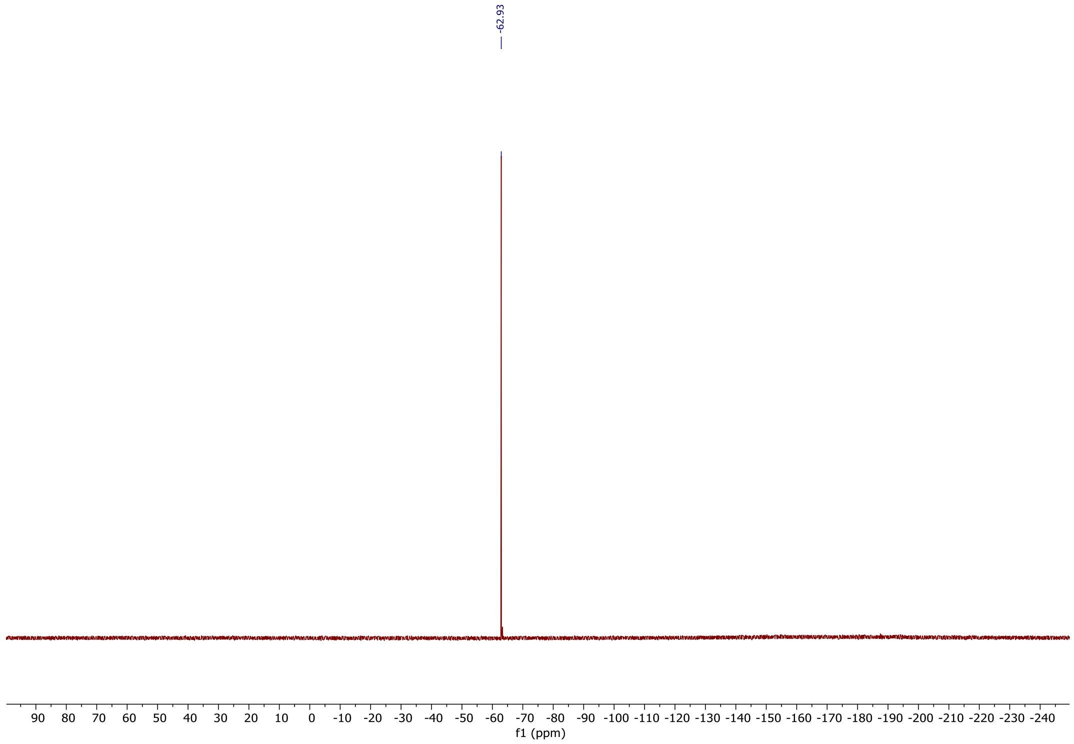

Sodium 3,5-difluorobenzenesulfinate, 2m

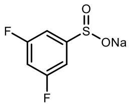

^{1}H NMR (400 MHz, MeOD)

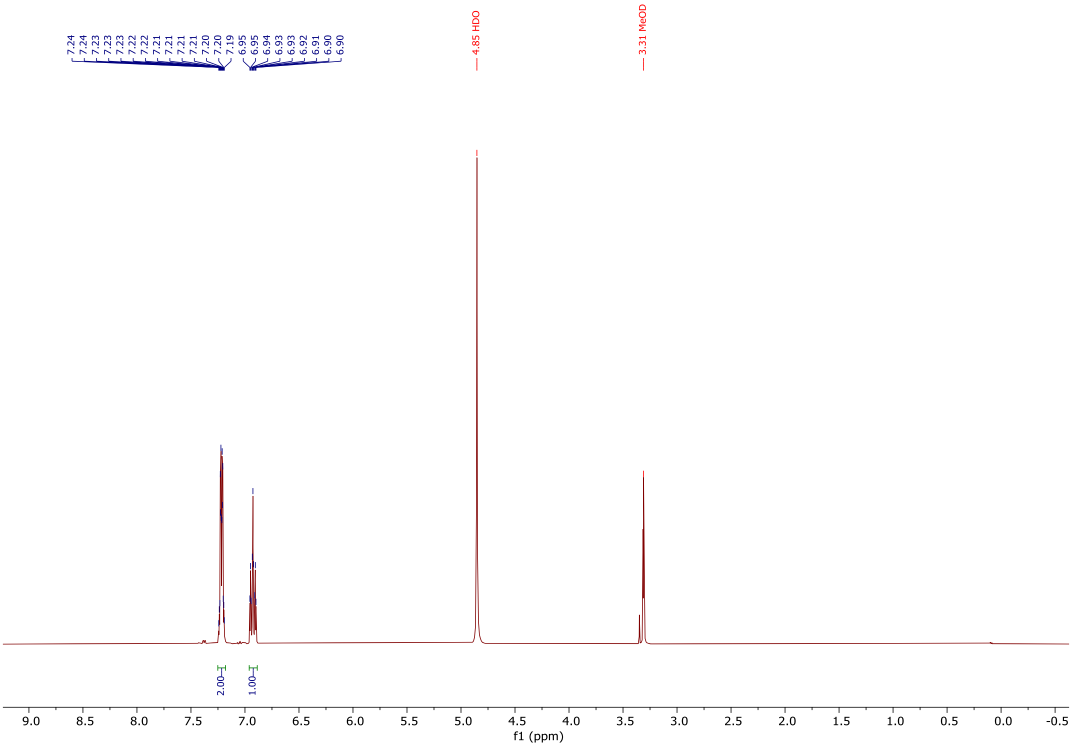

^{13}C NMR (101 MHz, MeOD)

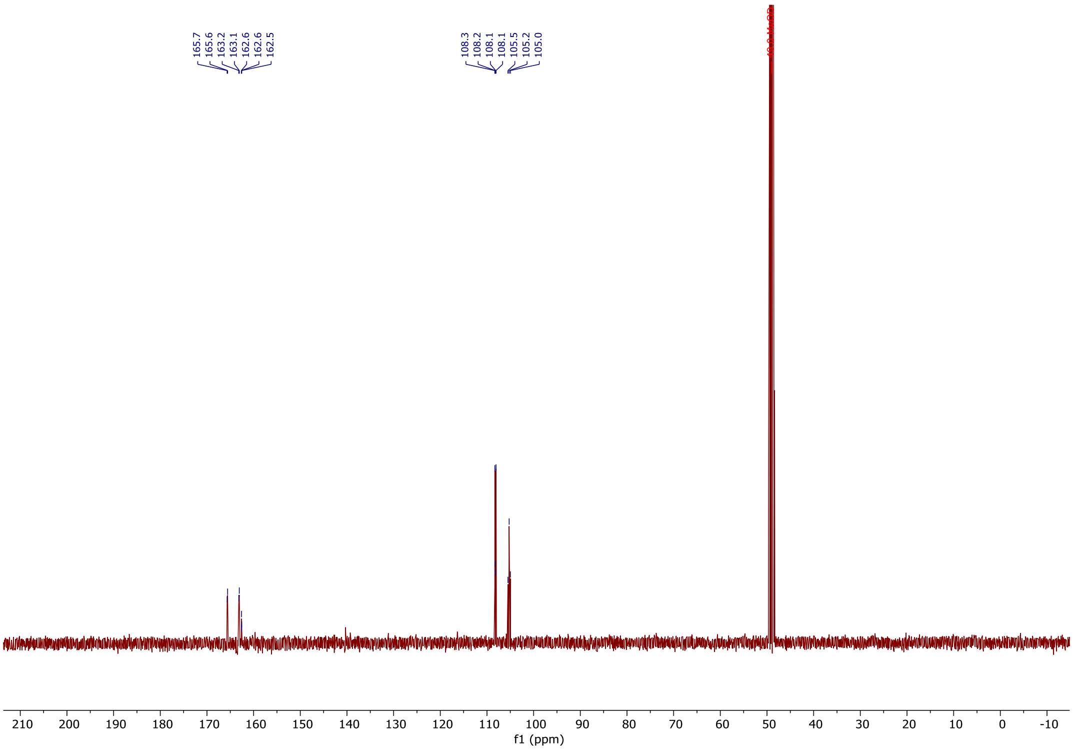

^{19}**F NMR** (377 MHz, MeOD)

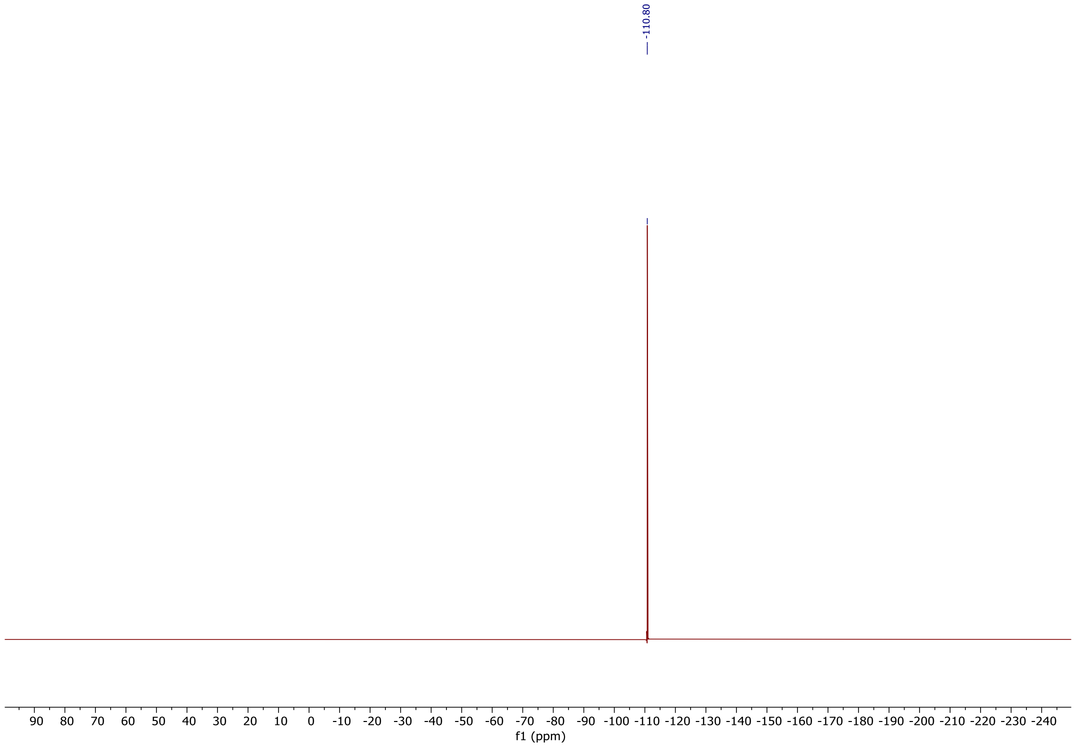

Sodium 3,5-bis(trifluoromethyl)benzenesulfinate, 2n

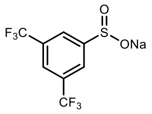

^{1}H NMR (600 MHz, MeOD)

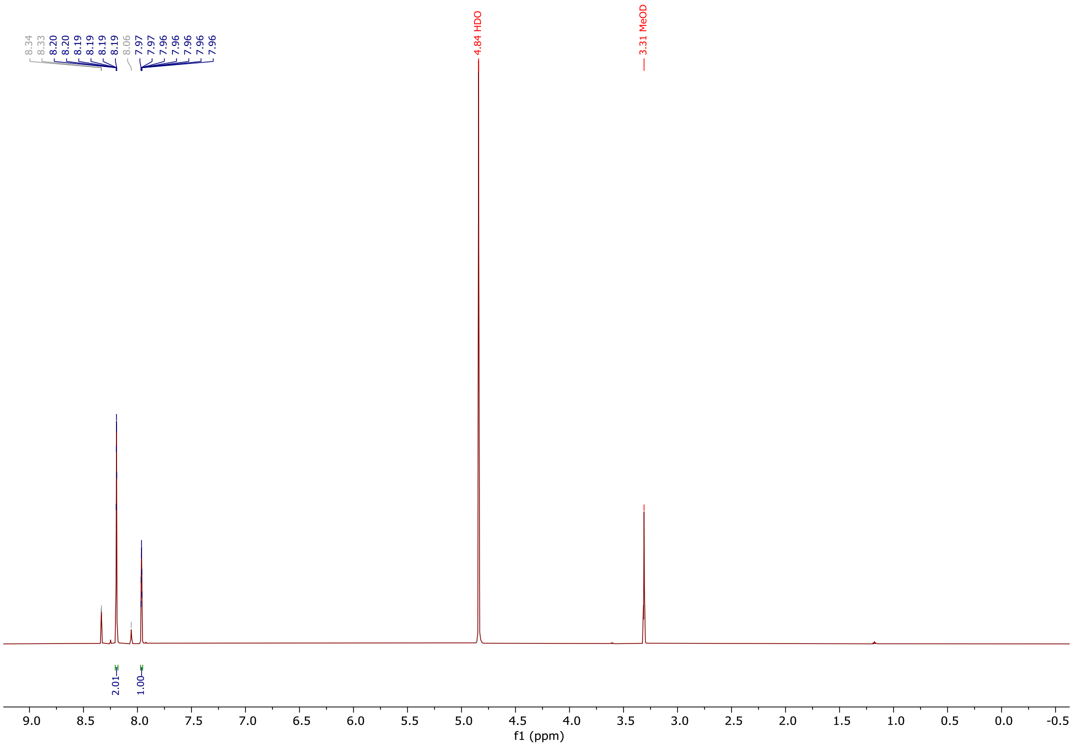

^{13}C NMR (151 MHz, MeOD)

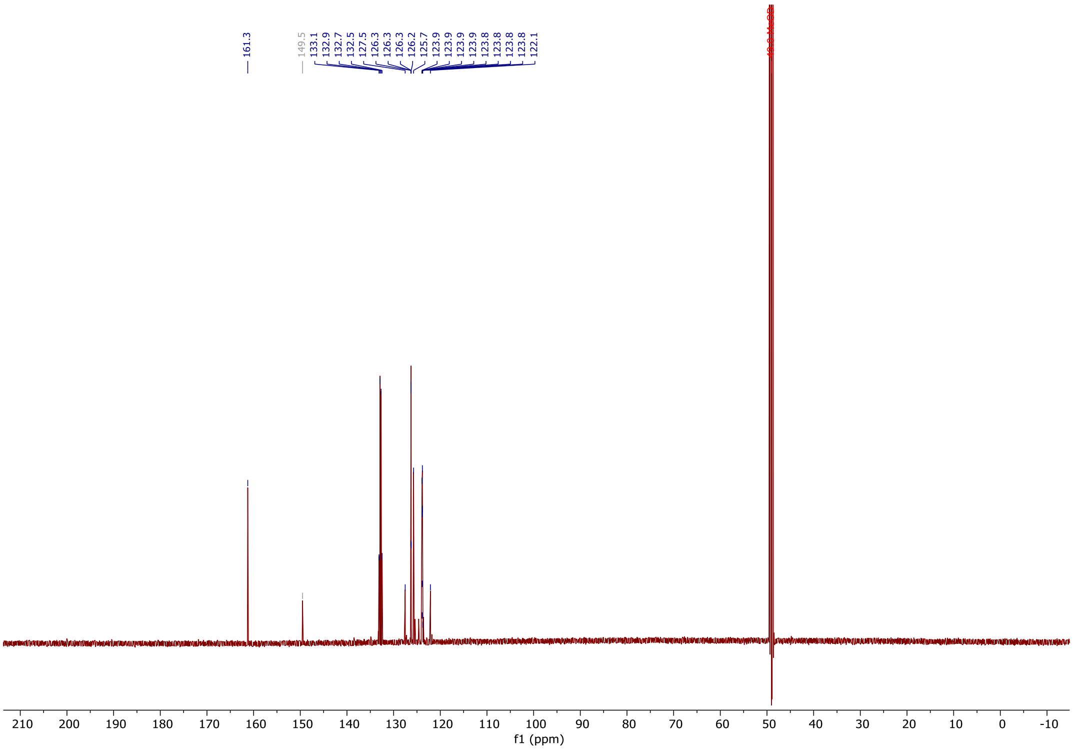

^{19}F NMR (377 MHz, MeOD)

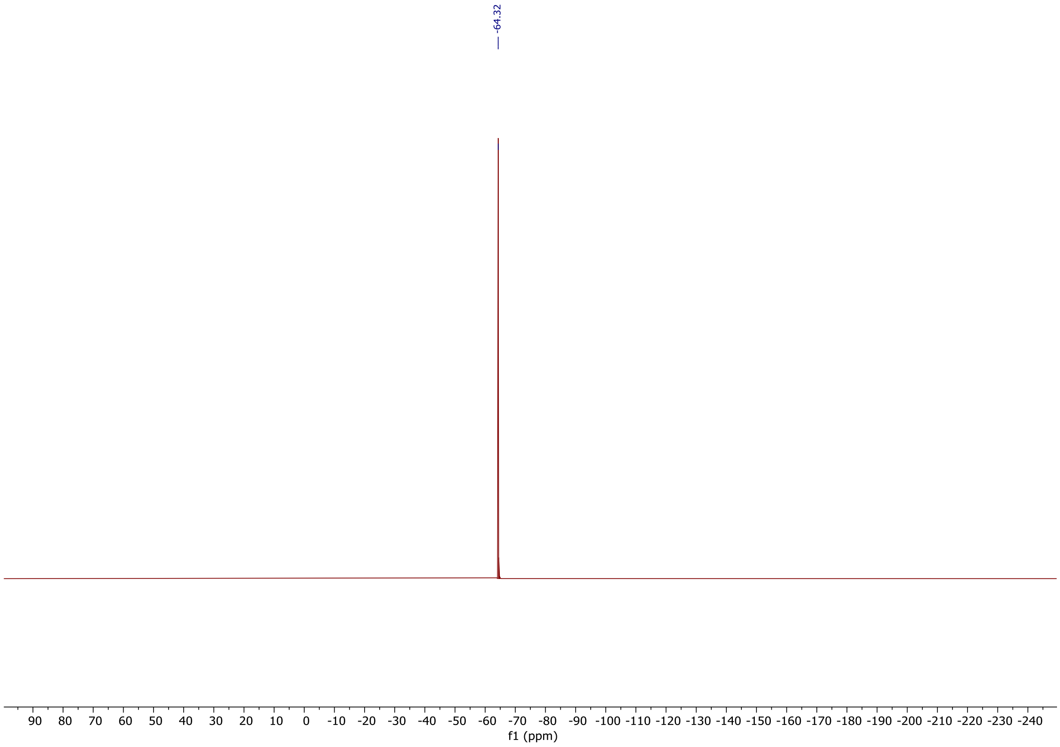

Sodium 4-acetamidobenzenesulfinate, 2o

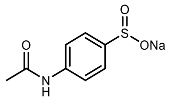

^{1}H NMR (101 MHz, MeOD)

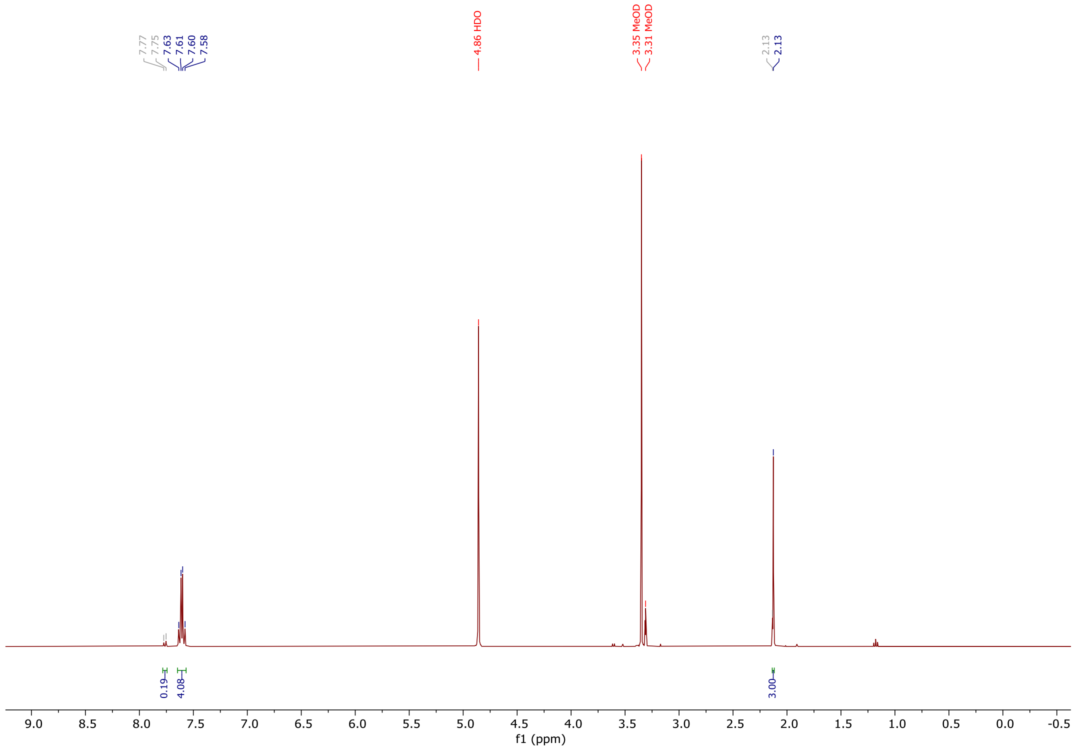

^{13}C NMR (101 MHz, MeOD)

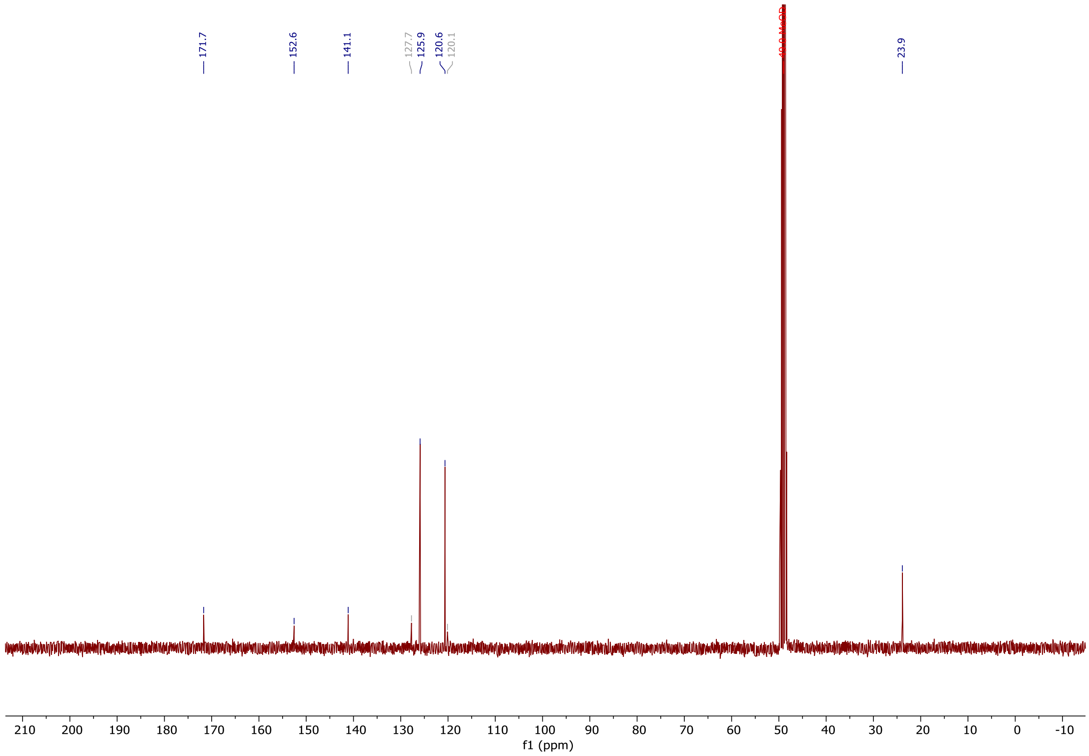

Sodium benzene-1,3-disulfinate, 2p

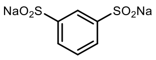

^{1}H NMR (400 MHz, MeOD)

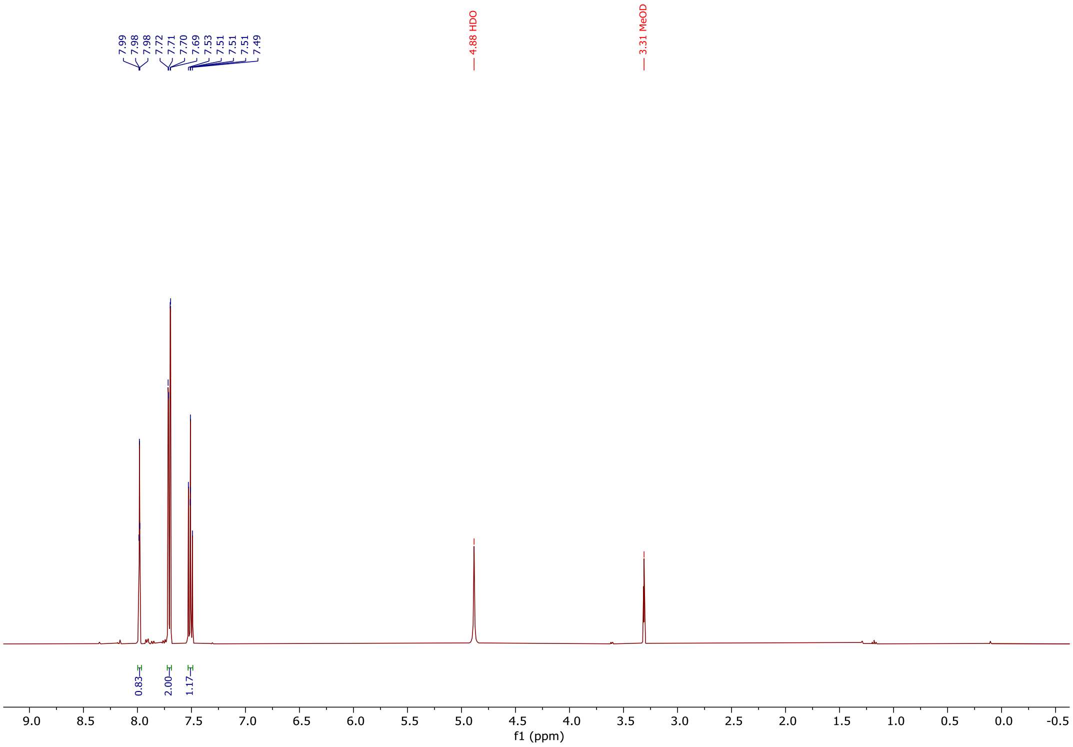

^{13}C NMR (101 MHz, MeOD)

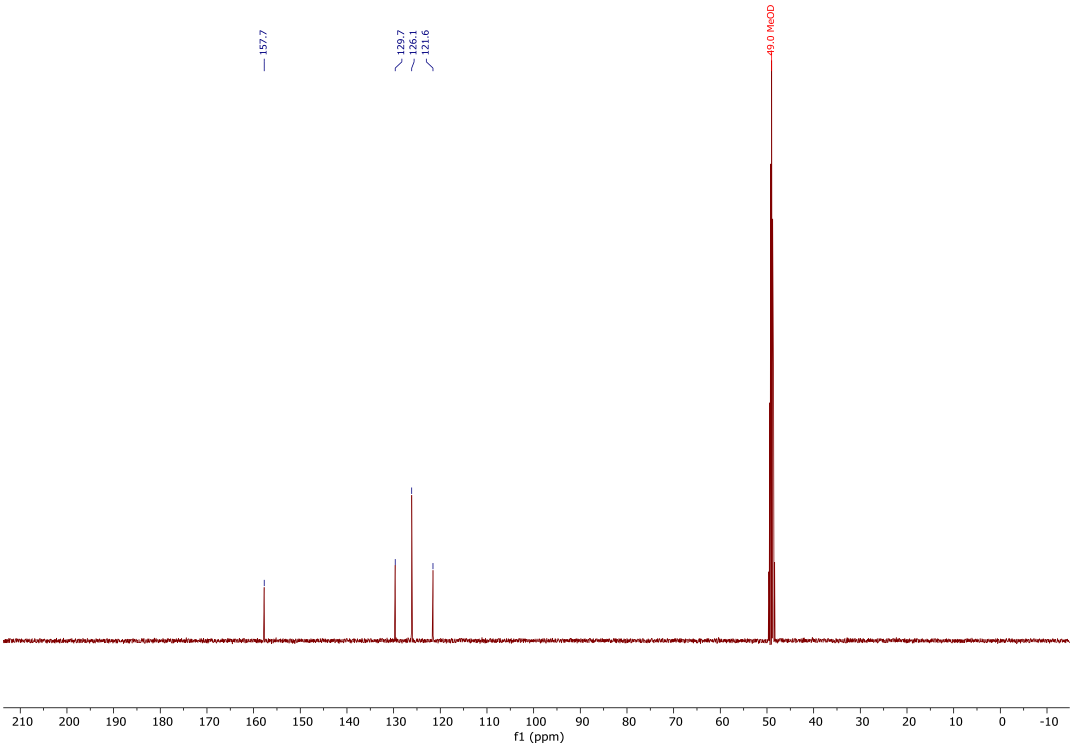

Sodium 5-bromothiophene-2-sulfinate, 2r

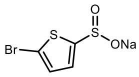

^{1}H NMR (400 MHz, MeOD)

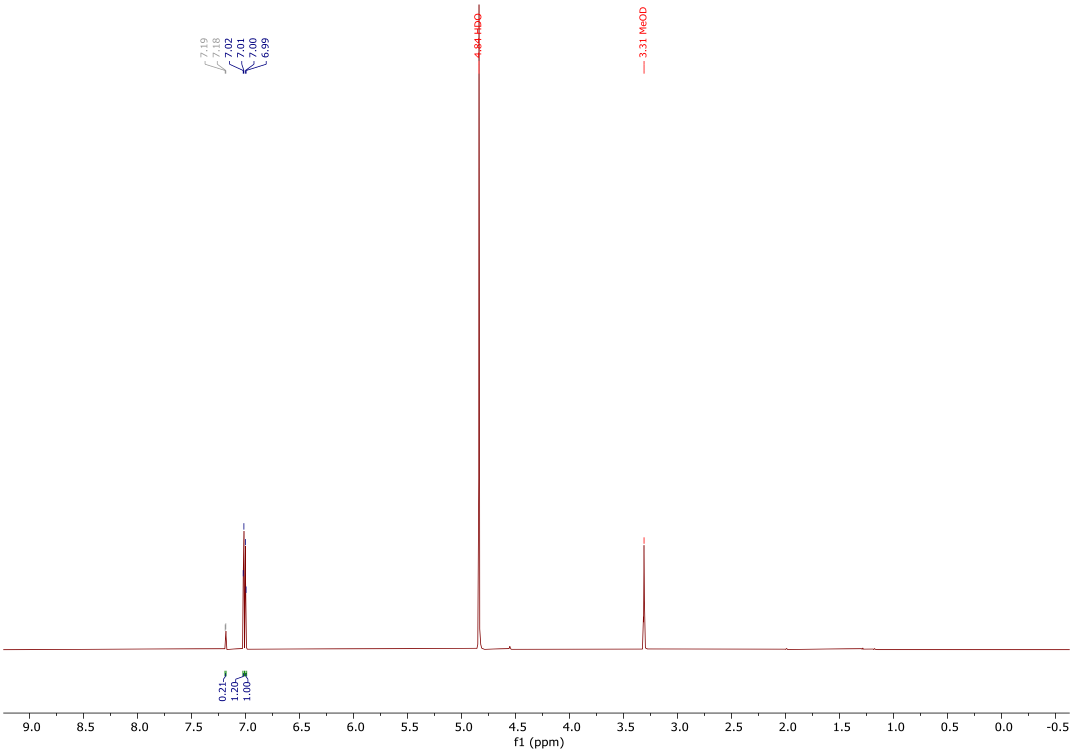

^{13}C NMR (101 MHz, MeOD)

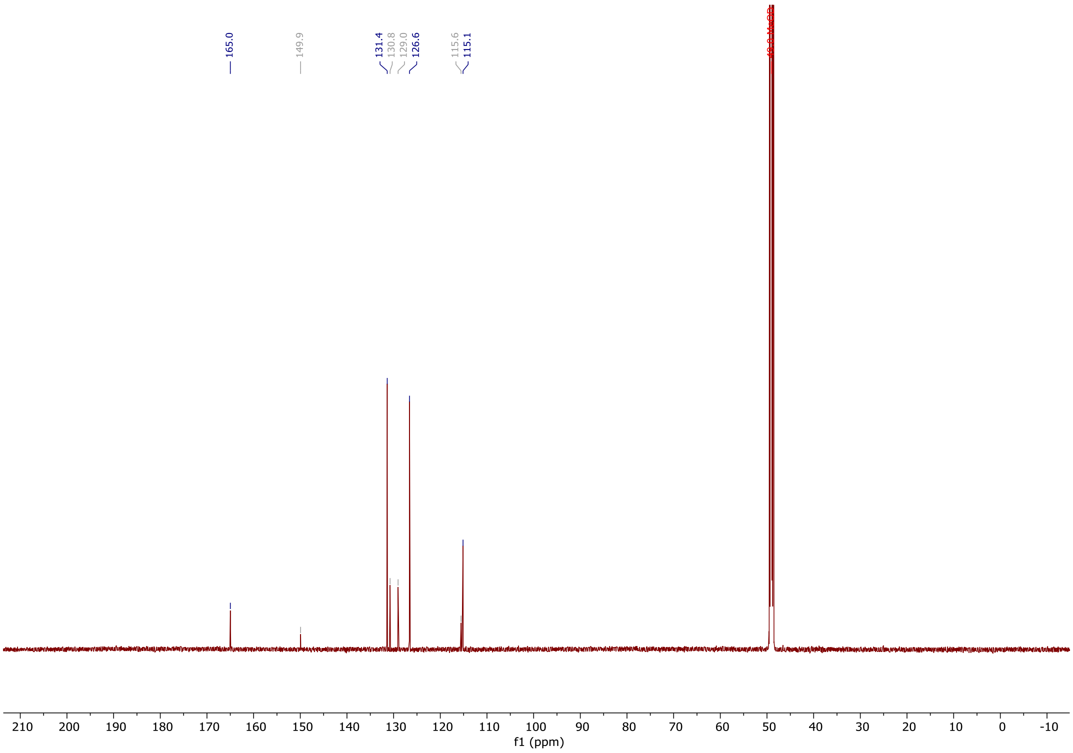

Sodium 1-methyl-1H-pyrazole-3-sulfinate, 2s

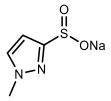

^{1}H NMR (400 MHz, MeOD)

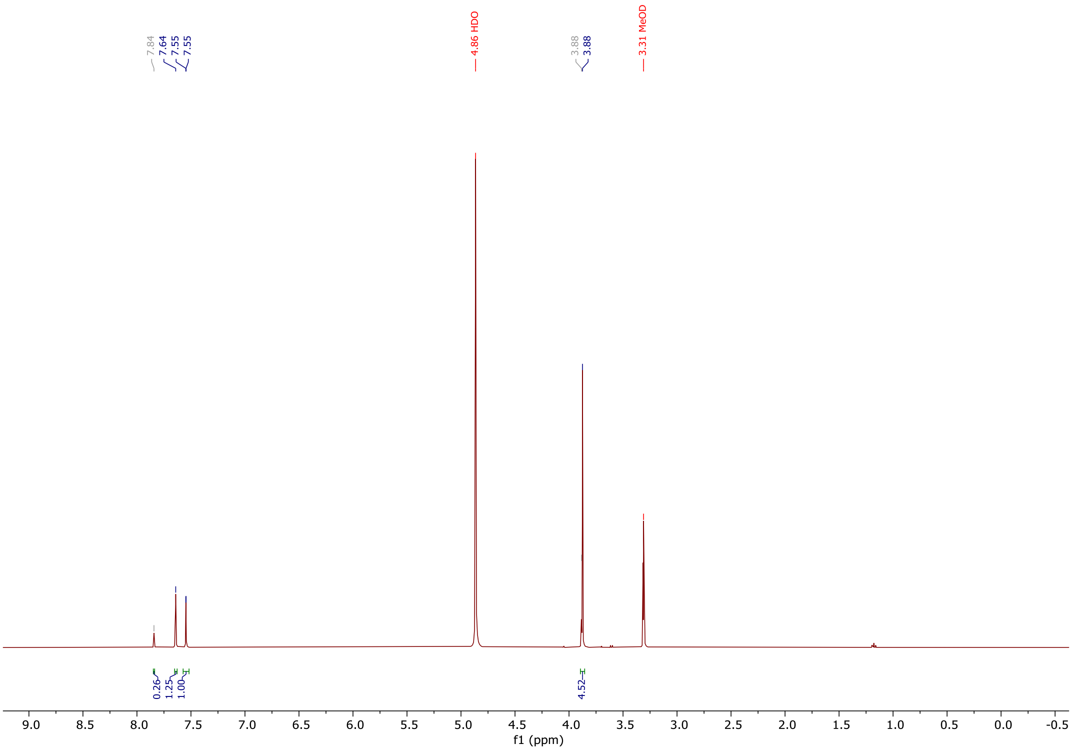

^{13}C NMR (101 MHz, MeOD)

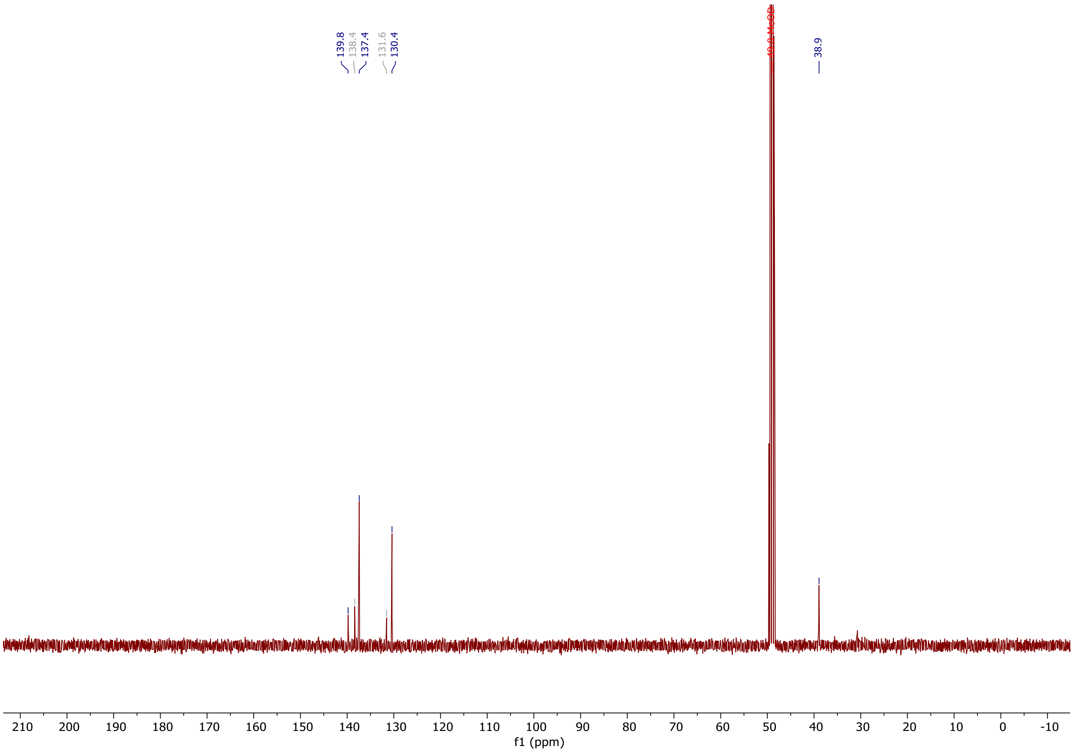

Sodium 1-methyl-1H-pyrazole-4-sulfinate, 2t

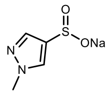

^{1}H NMR (500 MHz, D_2O)

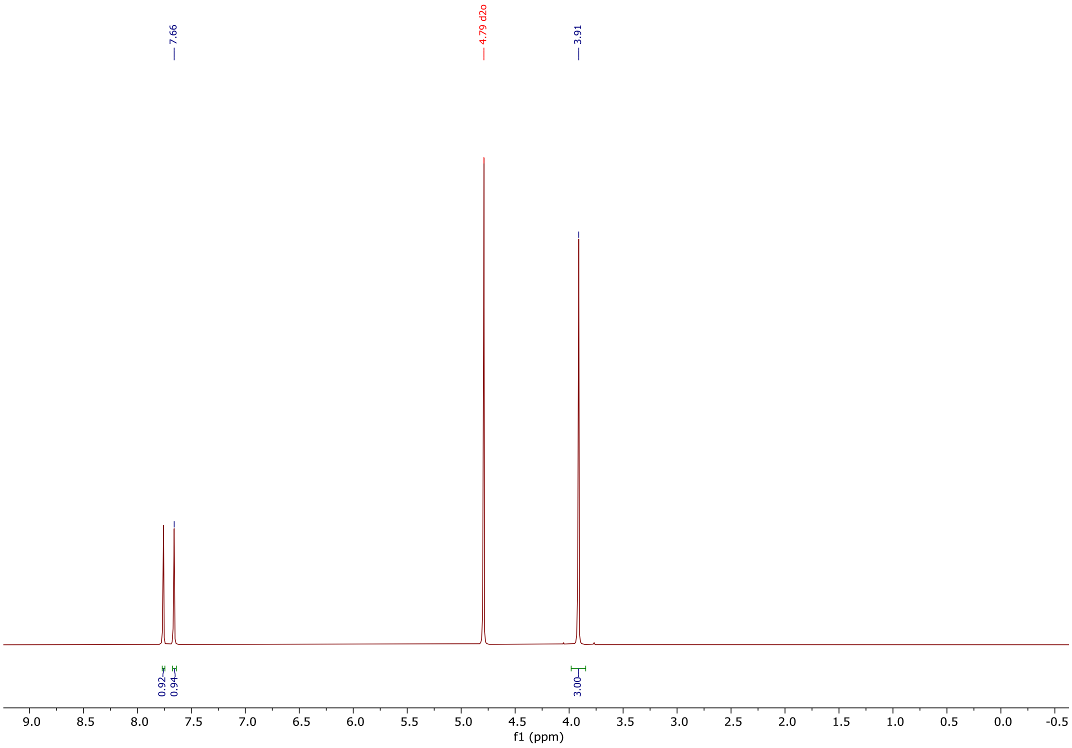

^{13}C NMR (151 MHz, D_2O)

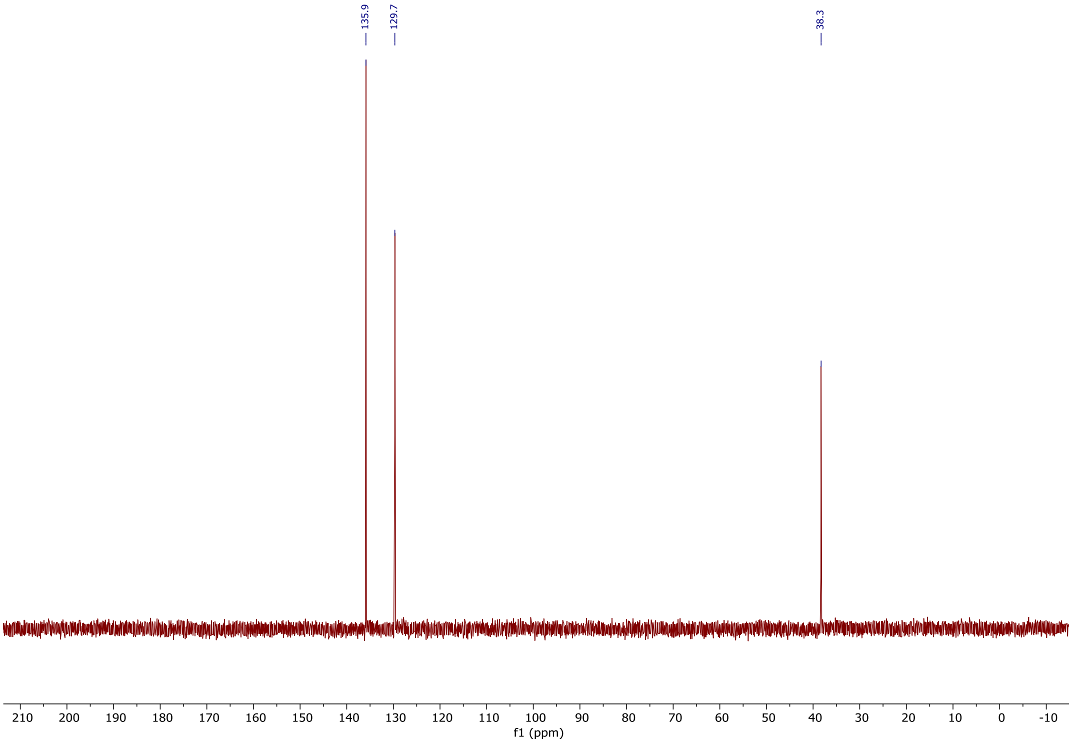

Lithium furan-3-sulfinate, 2u

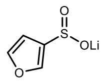

^{1}H NMR (500 MHz, D_2O)

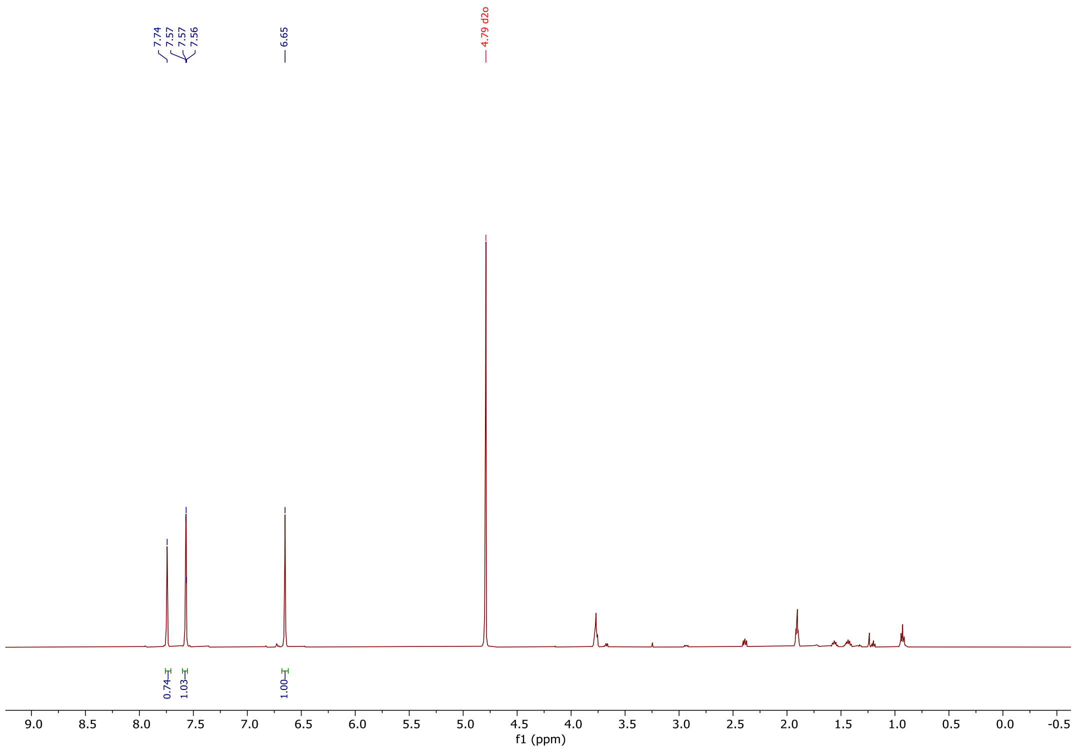

^{13}C NMR (126 MHz, D_2O)

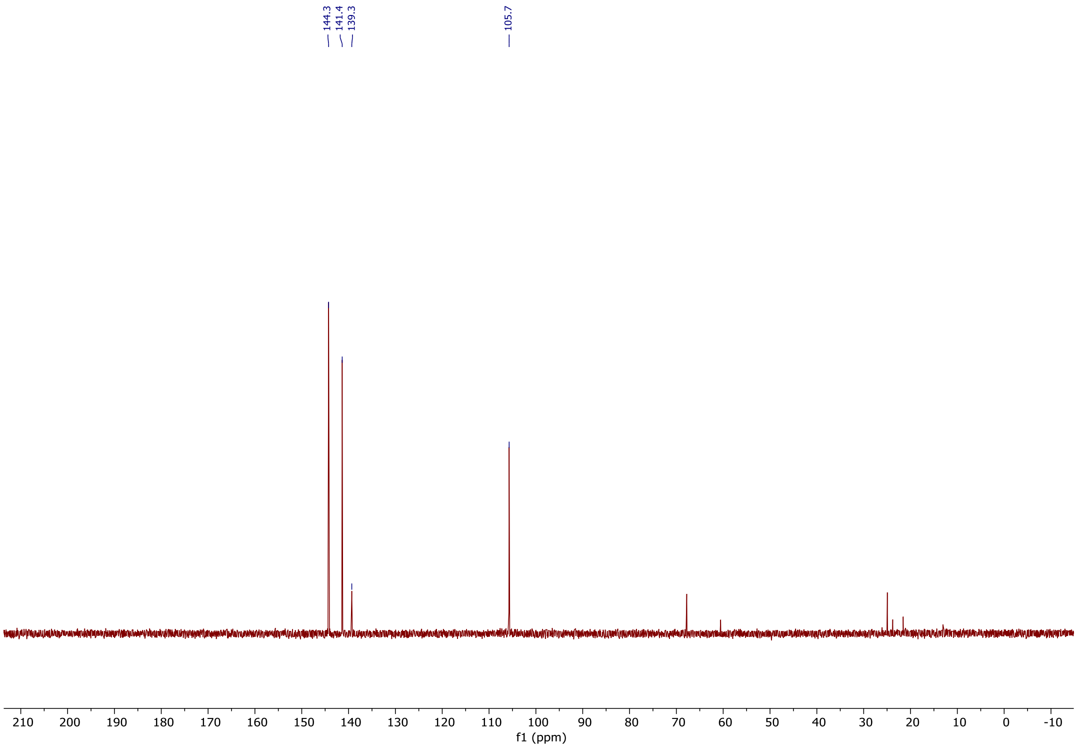

Sodium 3,5-dimethylisoxazole-4-sulfinate, 2v

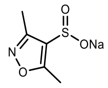

^{1}H NMR (400 MHz, MeOD)

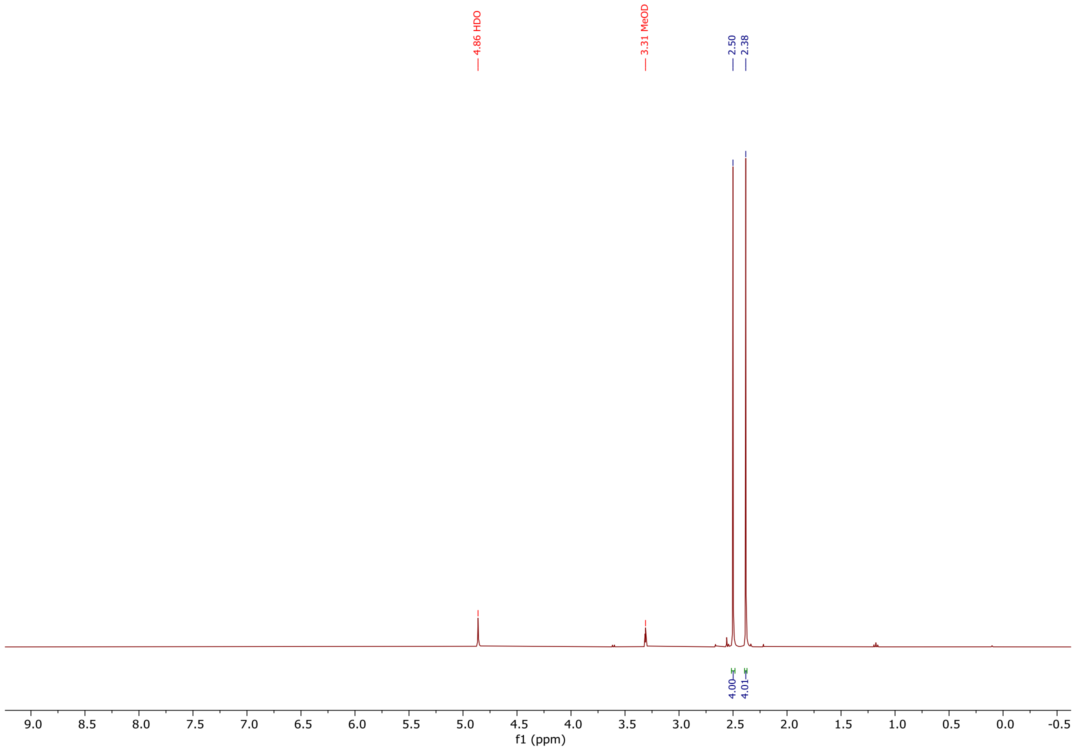

^{13}C NMR (101 MHz, MeOD)

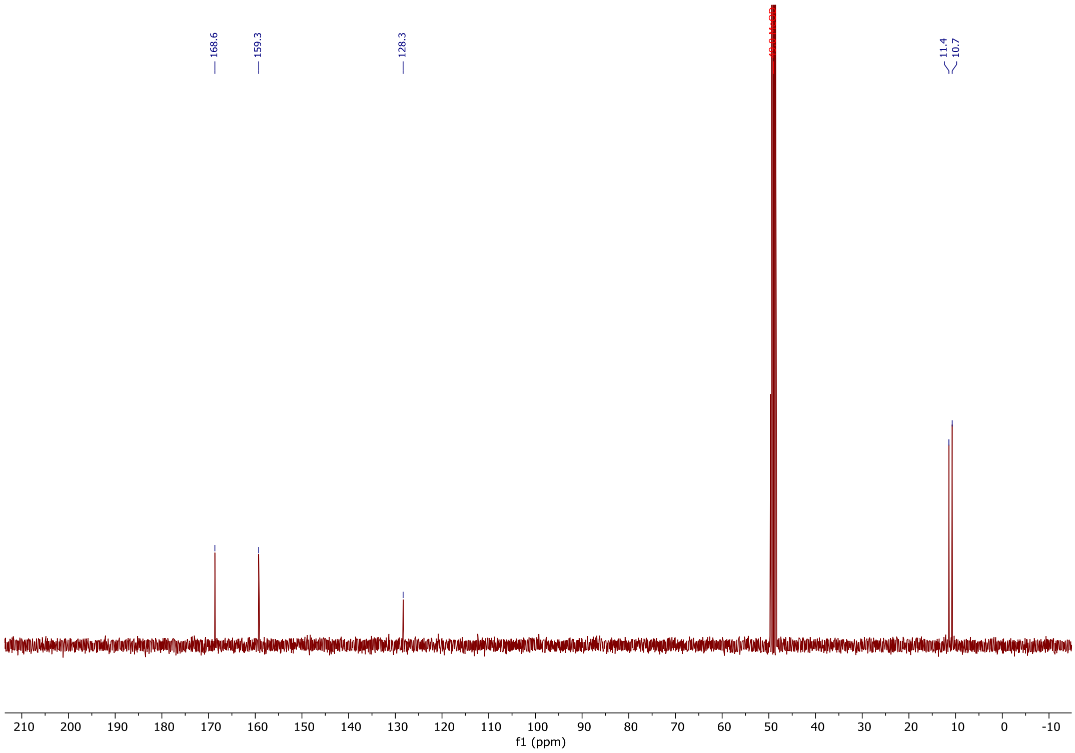

Sodium pyridine-3-sulfinate, 2w

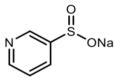

^{1}H NMR (400 MHz, MeOD)

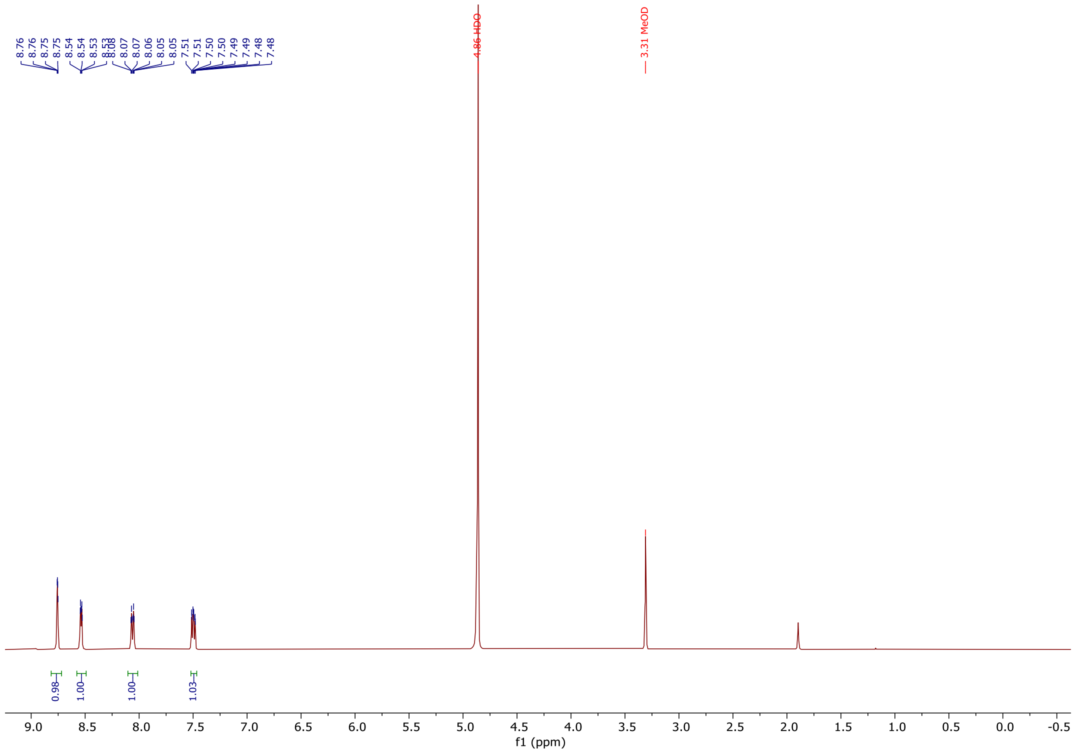

^{13}C NMR (101 MHz, MeOD)

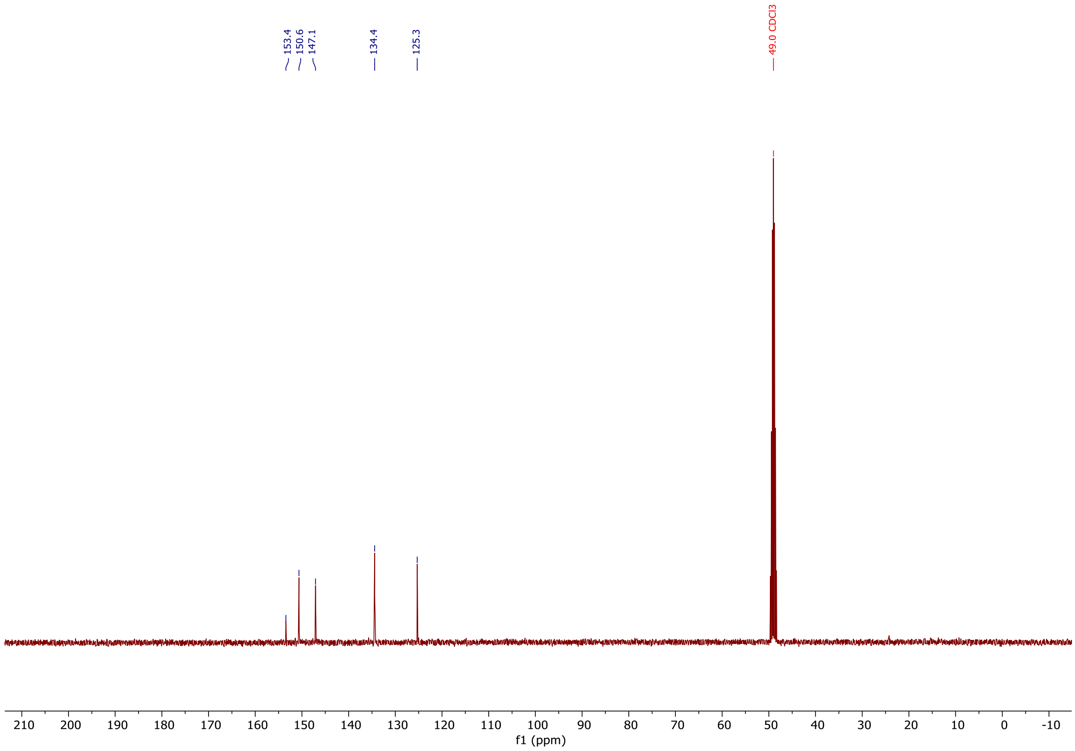

Lithium 6-methoxypyridine-3-sulfinate, 2x

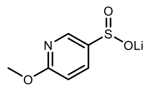

^{1}H NMR (400 MHz, D_2O)

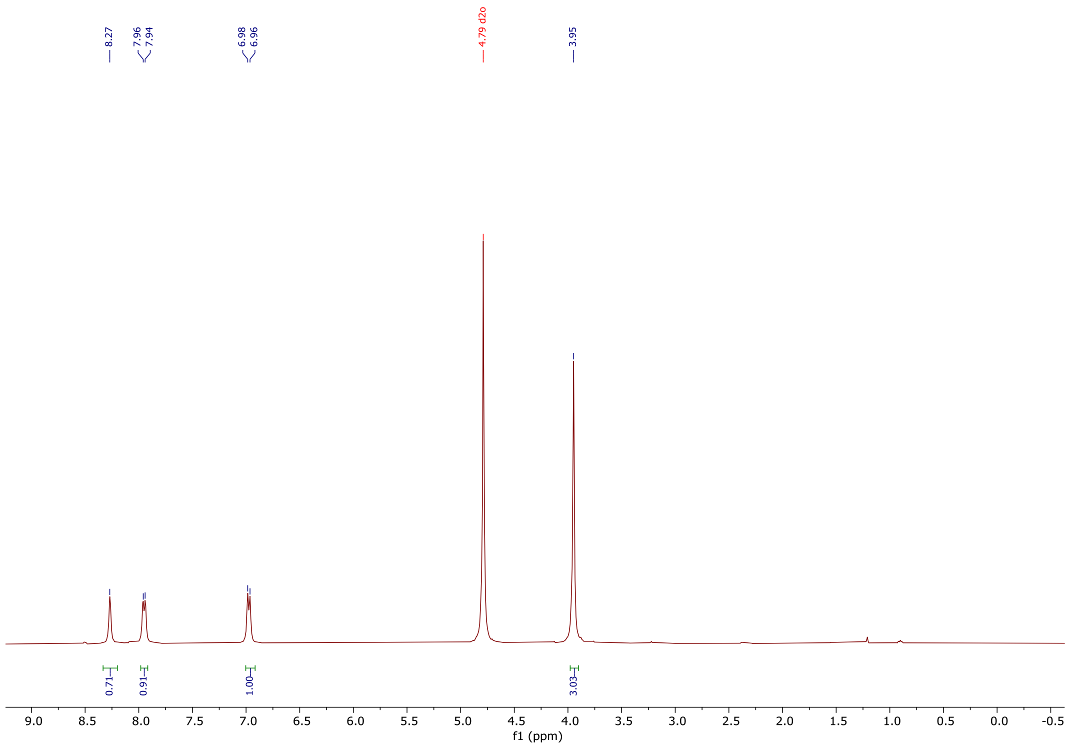

^{13}C NMR (126 MHz, D_2O)

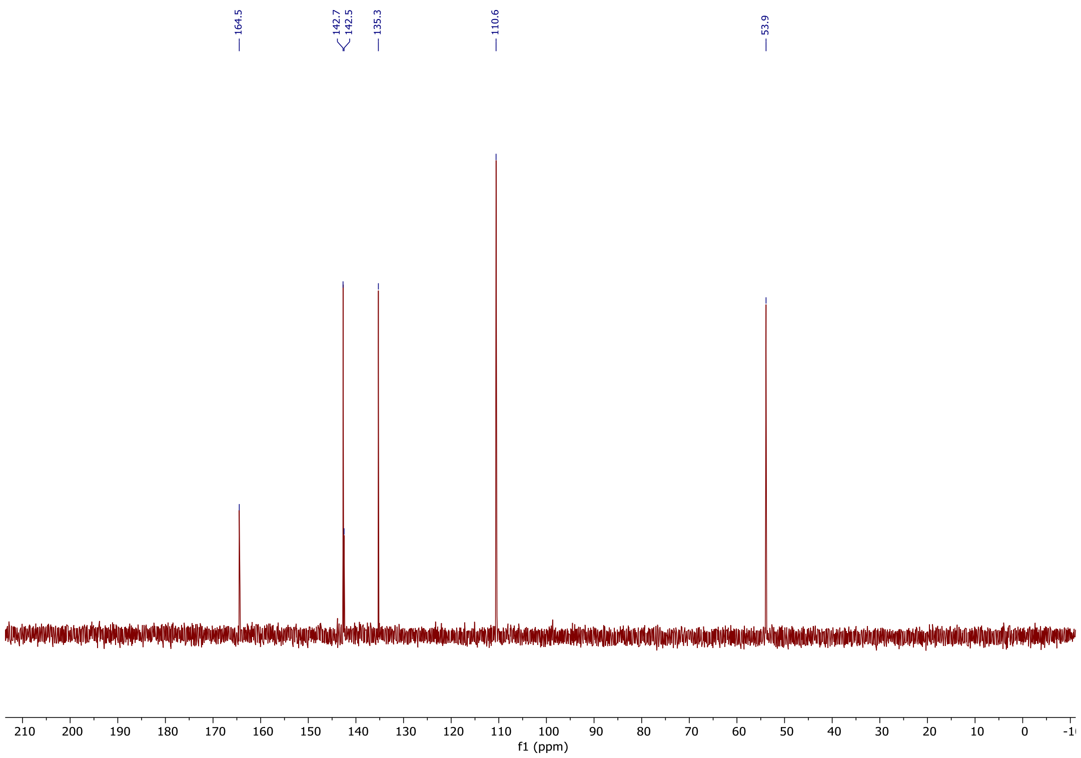

Lithium 6-chloropyridine-3-sulfinate, 2y

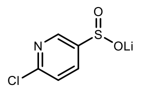

^{1}H NMR (500 MHz, DMSO-d_6)

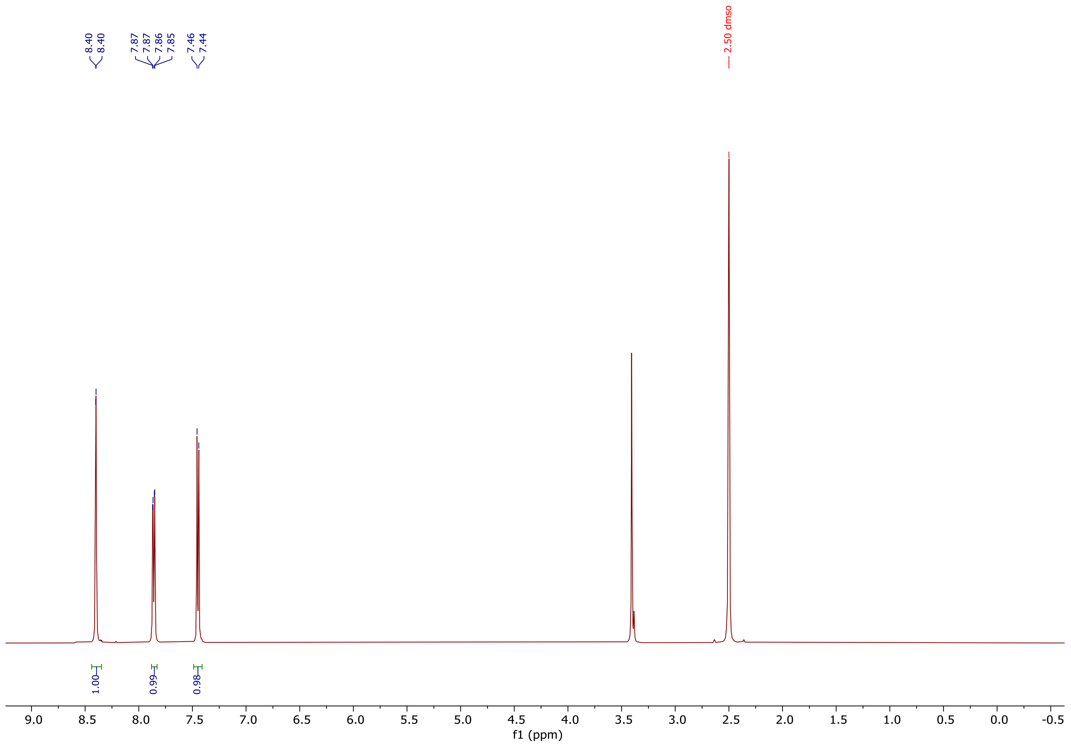

^{13}C NMR (126 MHz, DMSO-d_6)

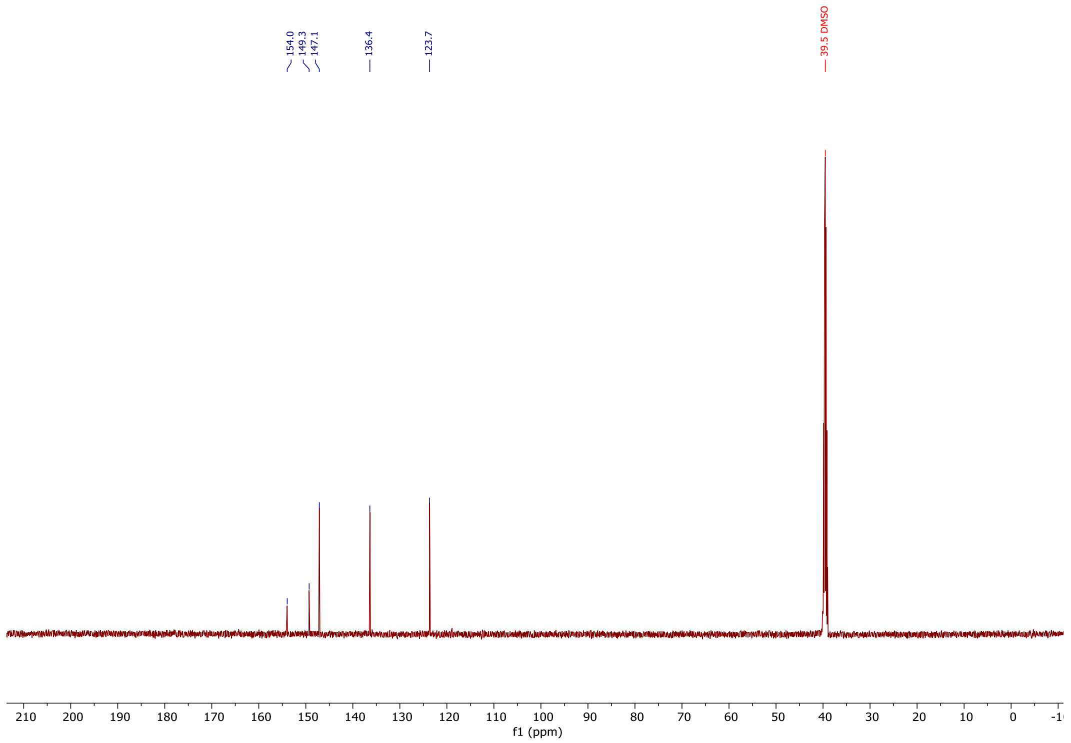

4-Ethoxy-3-(1-methyl–7-oxo-3-propyl-6,7-dihydro-1H-pyrazolo[4,3-d]pyrimidin-5-yl)benzenesulfonyl chloride, 2z2

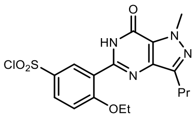

^{1}H NMR (600 MHz, $CDCl_3$)

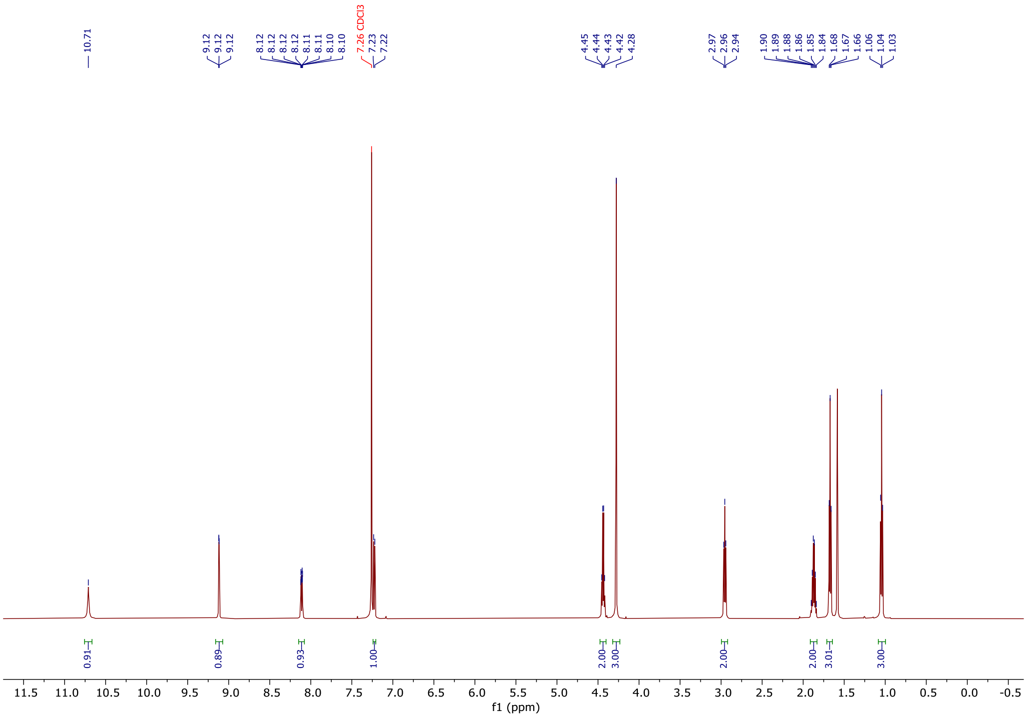

^{13}C NMR (151 MHz, $CDCl_3$)

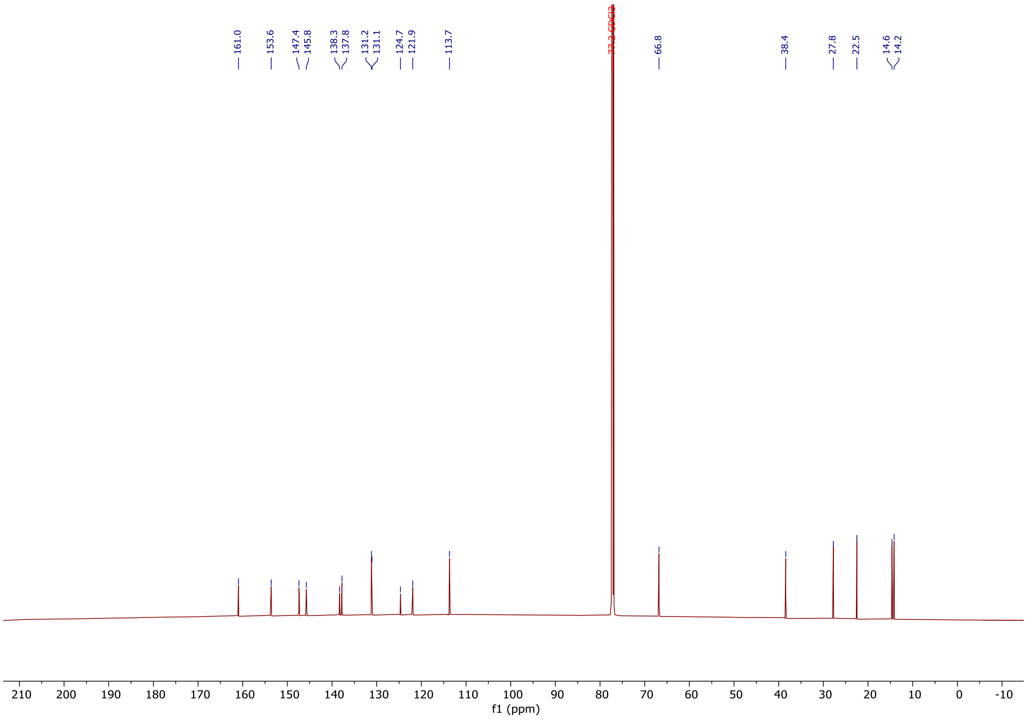

4-Ethoxy-3-(1-methyl–7-oxo-3-propyl-6,7-dihydro-1H-pyrazolo[4,3-d]pyrimidin-5-yl)benzenesulfonic acid, 2z3

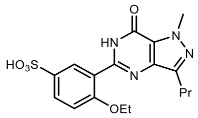

^{1}H NMR (400 MHz, CDCl$_3$)

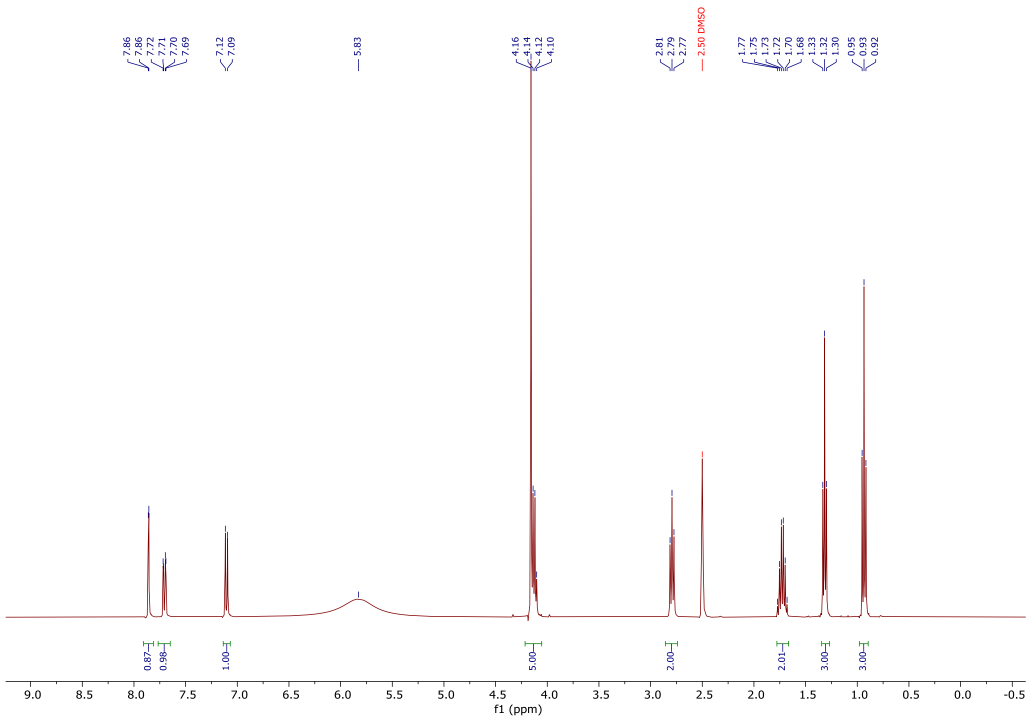

^{13}C NMR (101 MHz, CDCl$_3$)

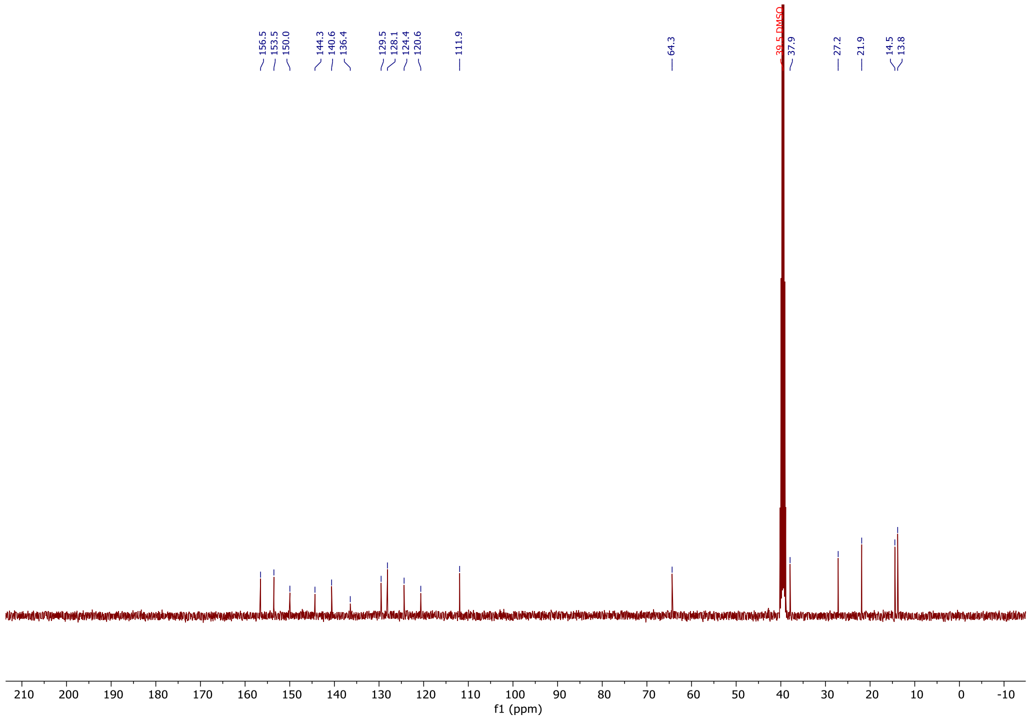

Sodium 4-ethoxy-3-(1-methyl–7-oxo-3-propyl-6,7-dihydro-1H-pyrazolo[4,3-d]pyrimidin-5-yl)benzenesulfinate, 2z

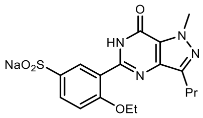

^{1}H NMR (600 MHz, MeOD)

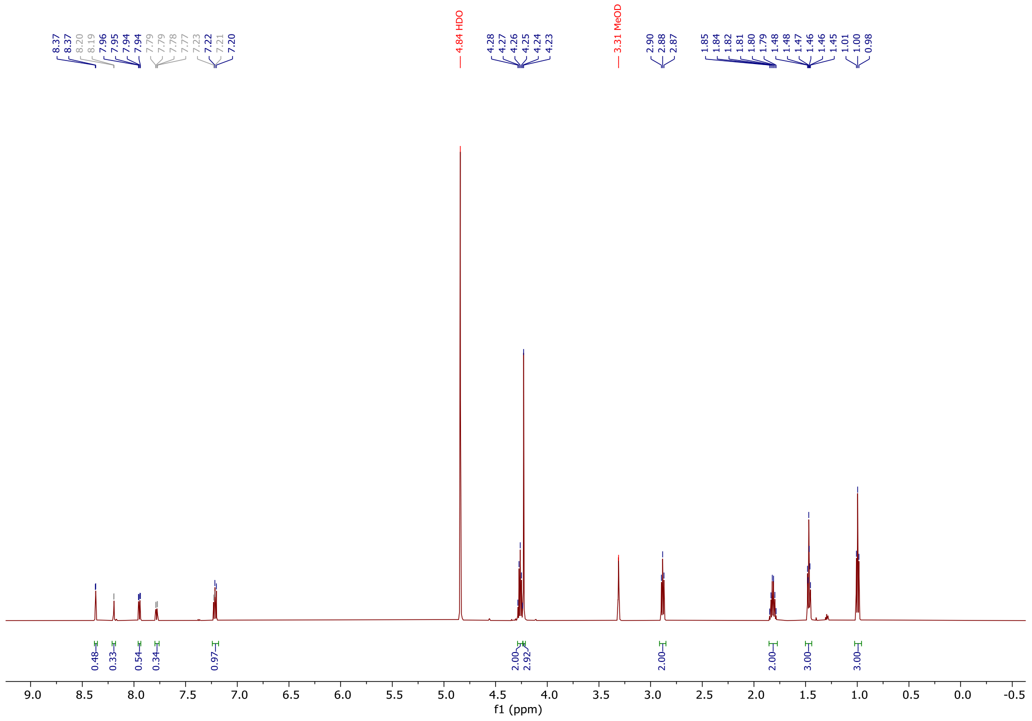

^{13}C NMR (151 MHz, MeOD)

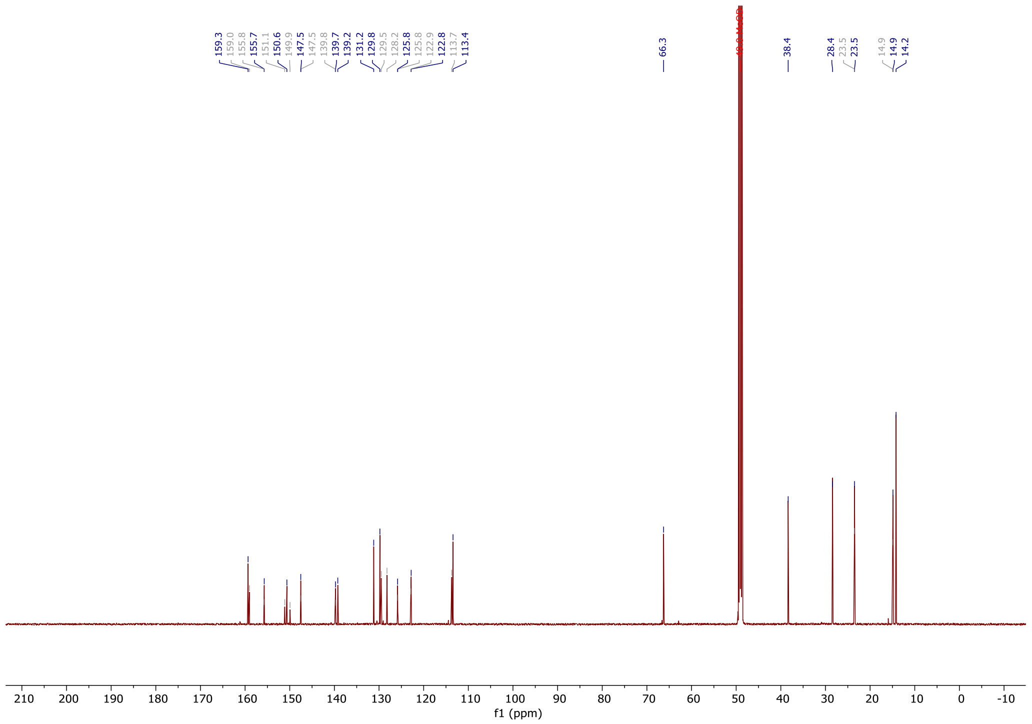

3-(Benzylthio)-*N*,*N*-diethyl-4H-1,2,4-triazole-4-carboxamide, 2aa1

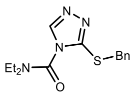

^{1}H NMR (400 MHz, $CDCl_3$)

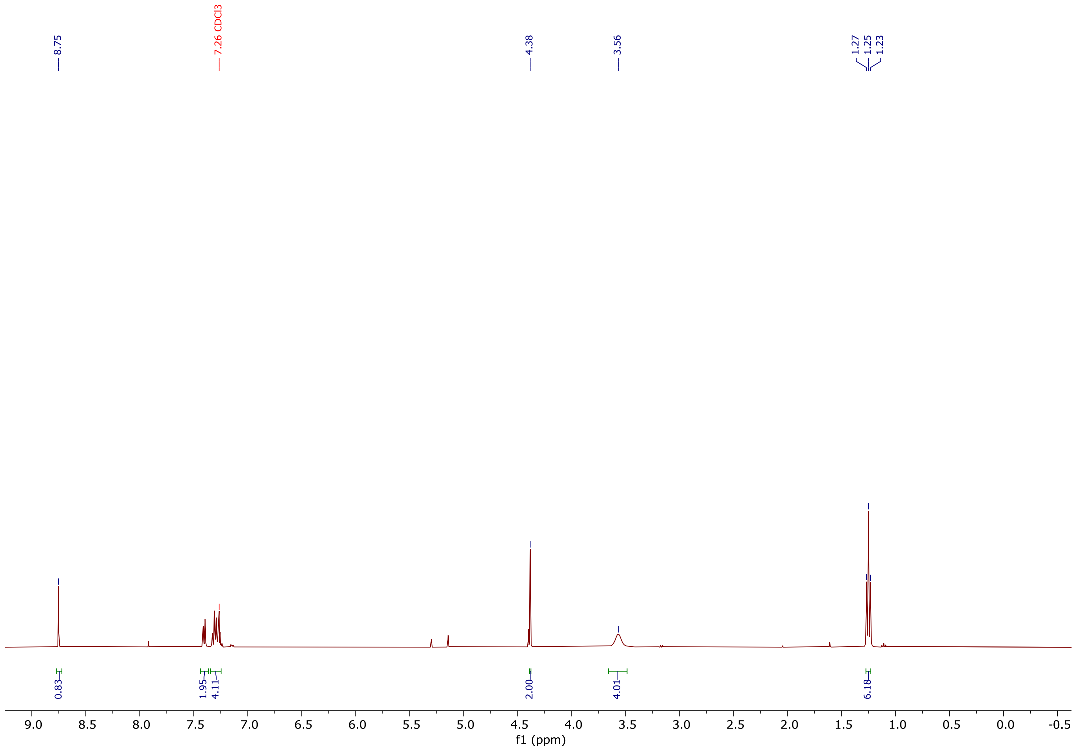

^{13}C NMR (101 MHz, $CDCl_3$)

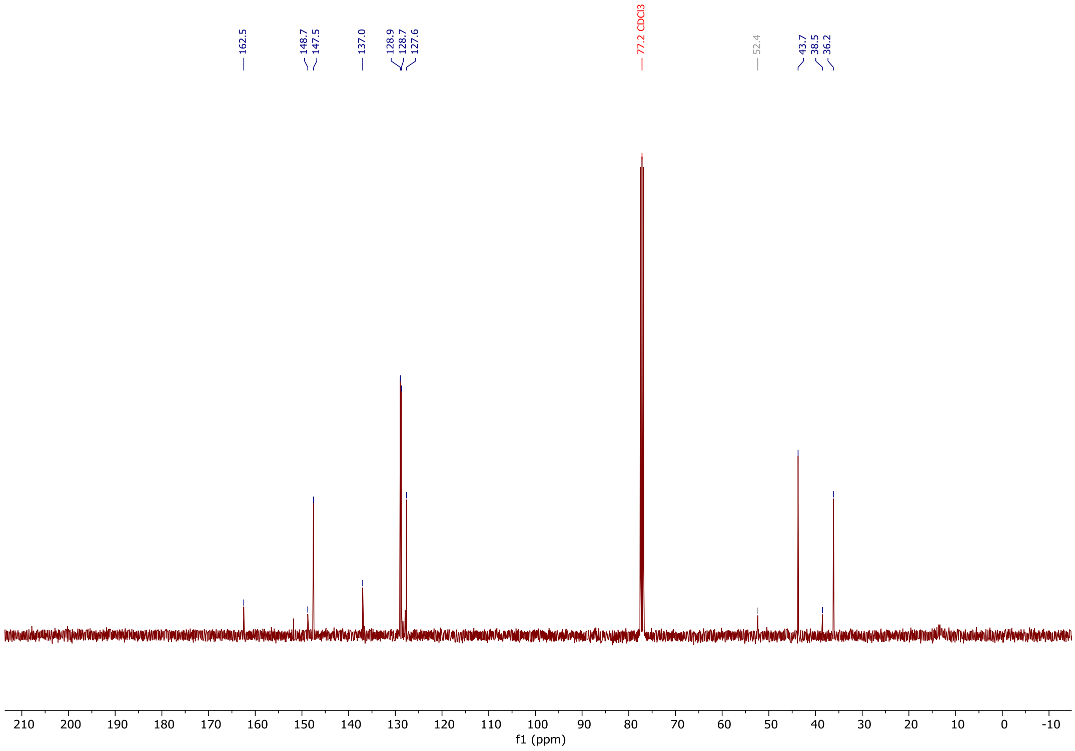

4-(Diethylcarbamoyl)-4H-1,2,4-triazole-3-sulfonyl chloride, 2aa2

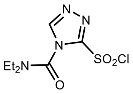

^{1}H NMR (400 MHz, $CDCl_3$)

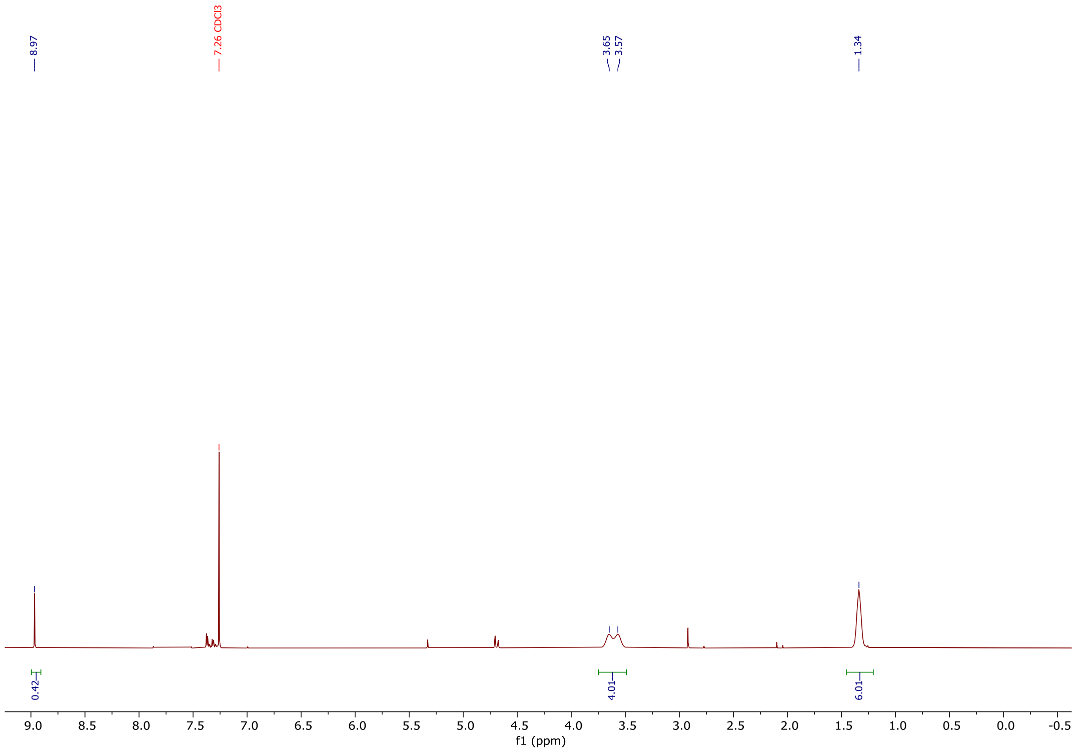

^{13}C NMR 101 MHz, $CDCl_3$)

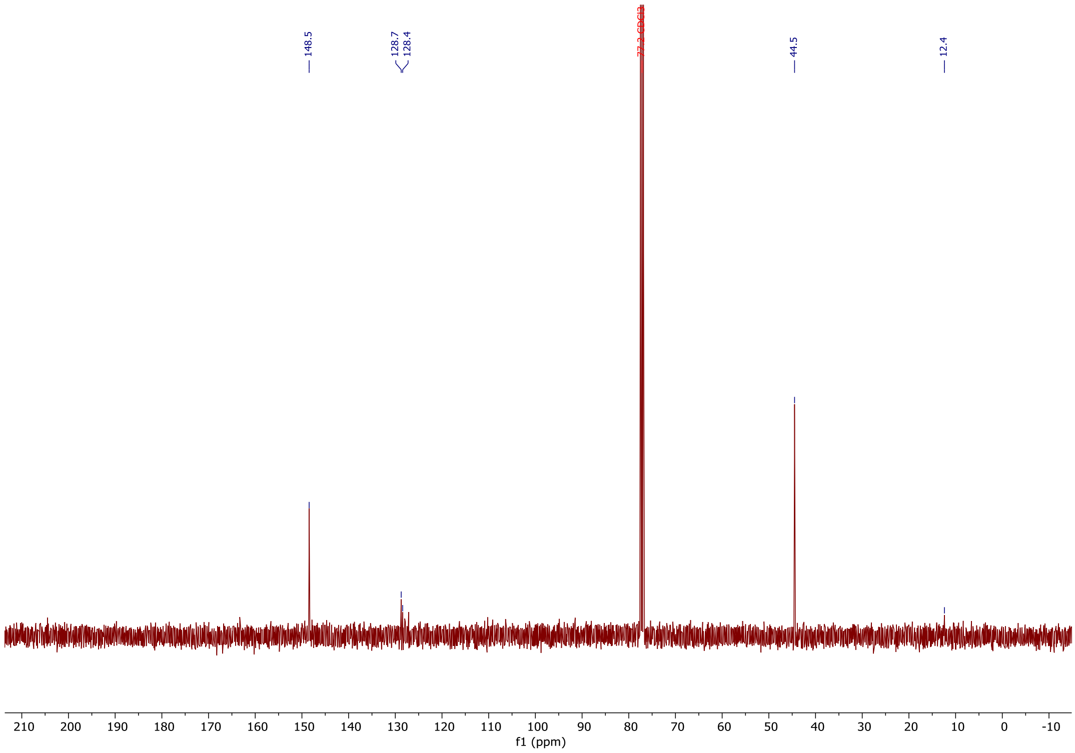

Sodium 4-(diethylcarbamoyl)-4H-1,2,4-triazole-3-sulfinate, 2aa

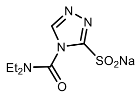

^{1}H NMR (600 MHz, MeOD)

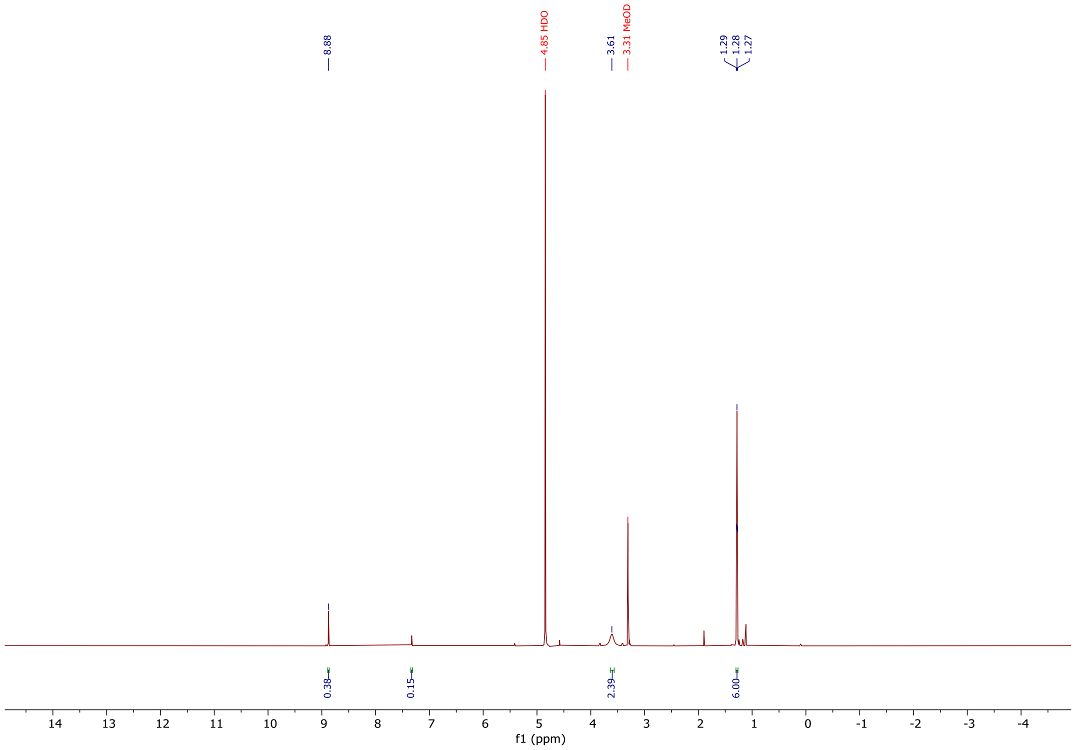

^{13}C NMR (151 MHz, MeOD)

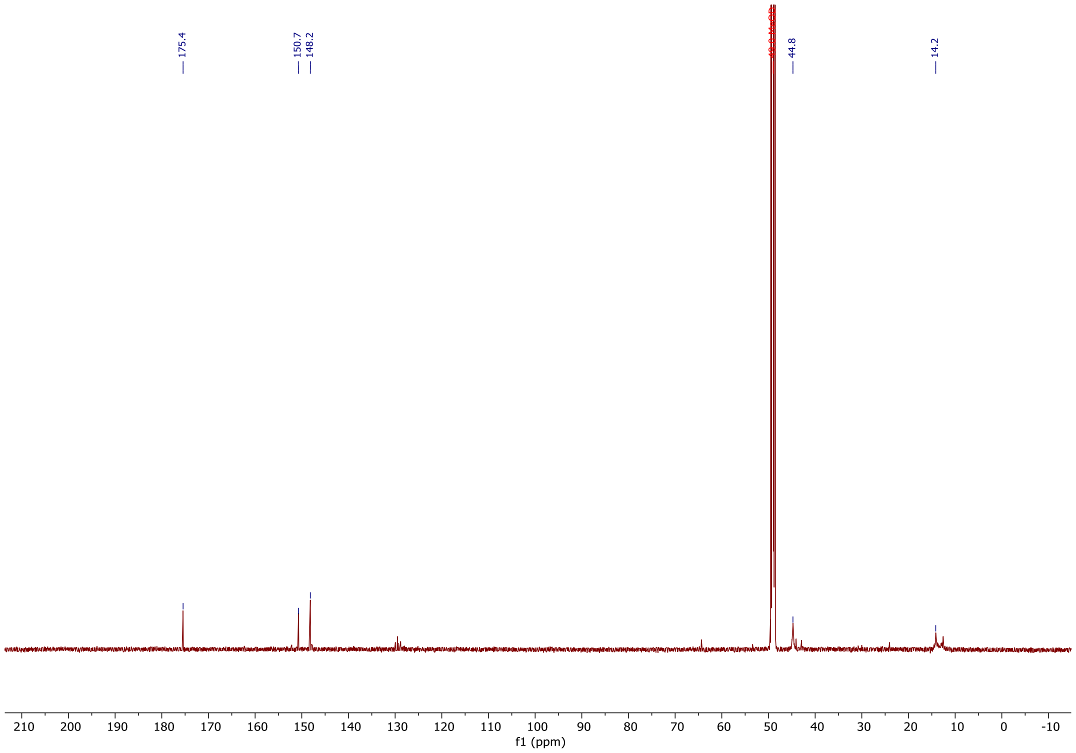

Sodium cyclopropanesulfinate, 6f

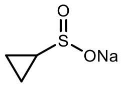

^{1}H NMR (400 MHz, DMSO-d$_6$)

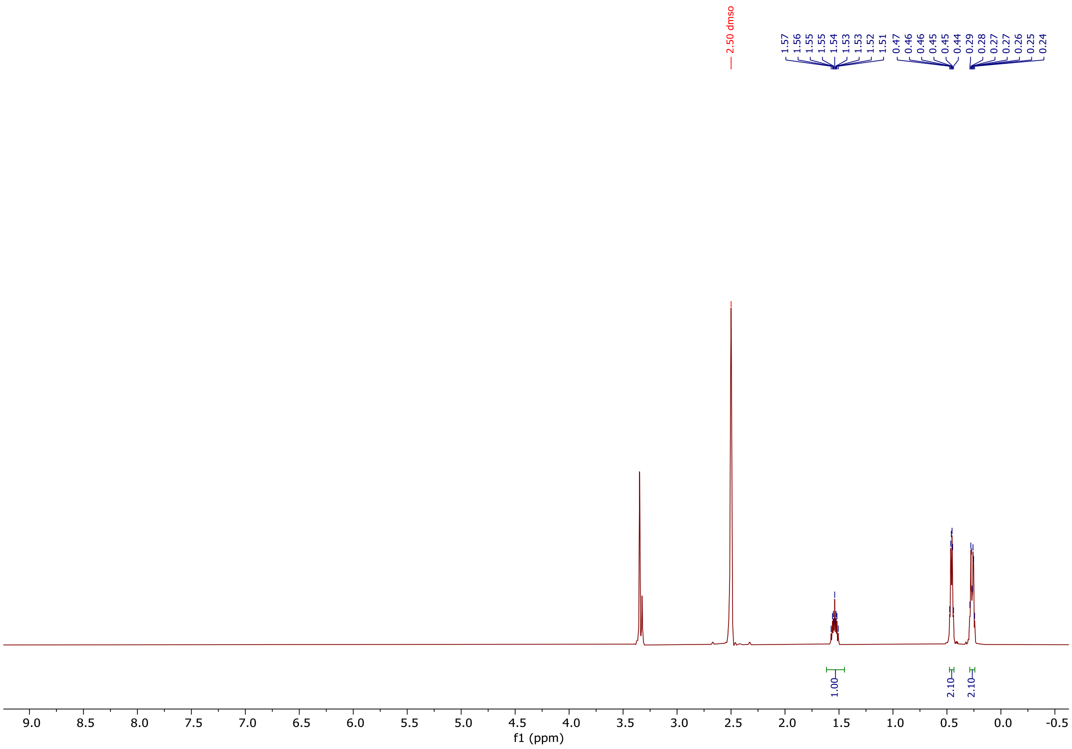

^{13}C NMR (126 MHz, DMSO-d$_6$)

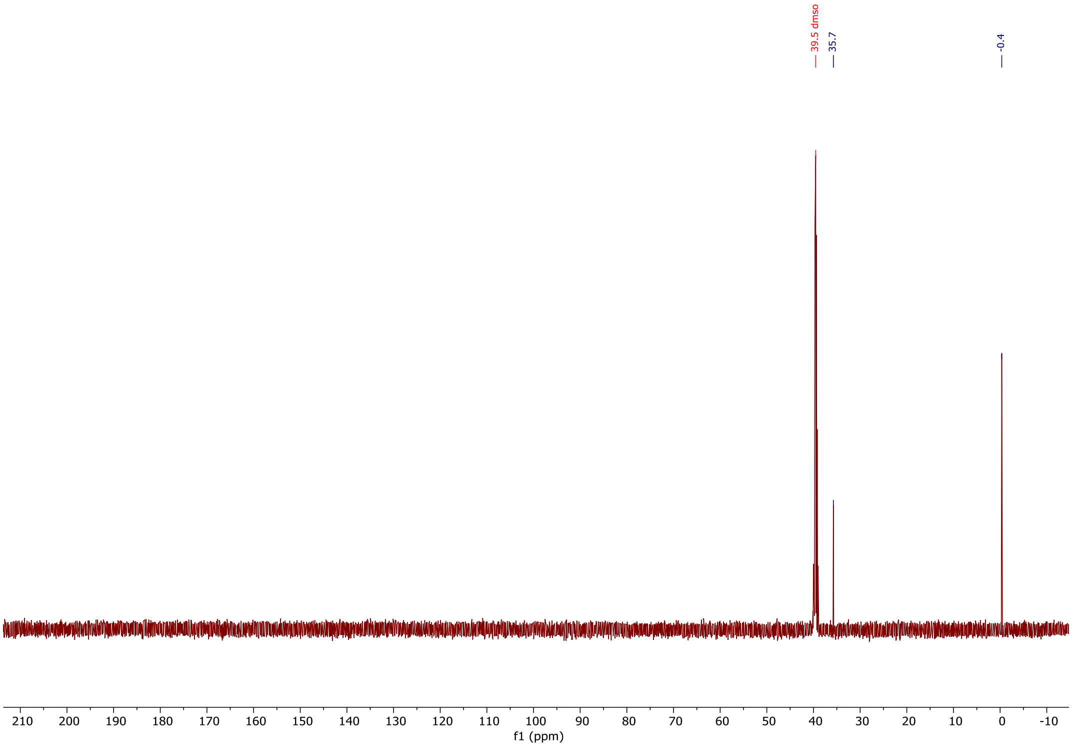

S-(Bicyclo[1.1.1]pentan-1-yl) ethanethioate, 6l1

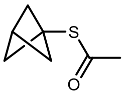

^{1}H NMR (500 MHz, $CDCl_3$)

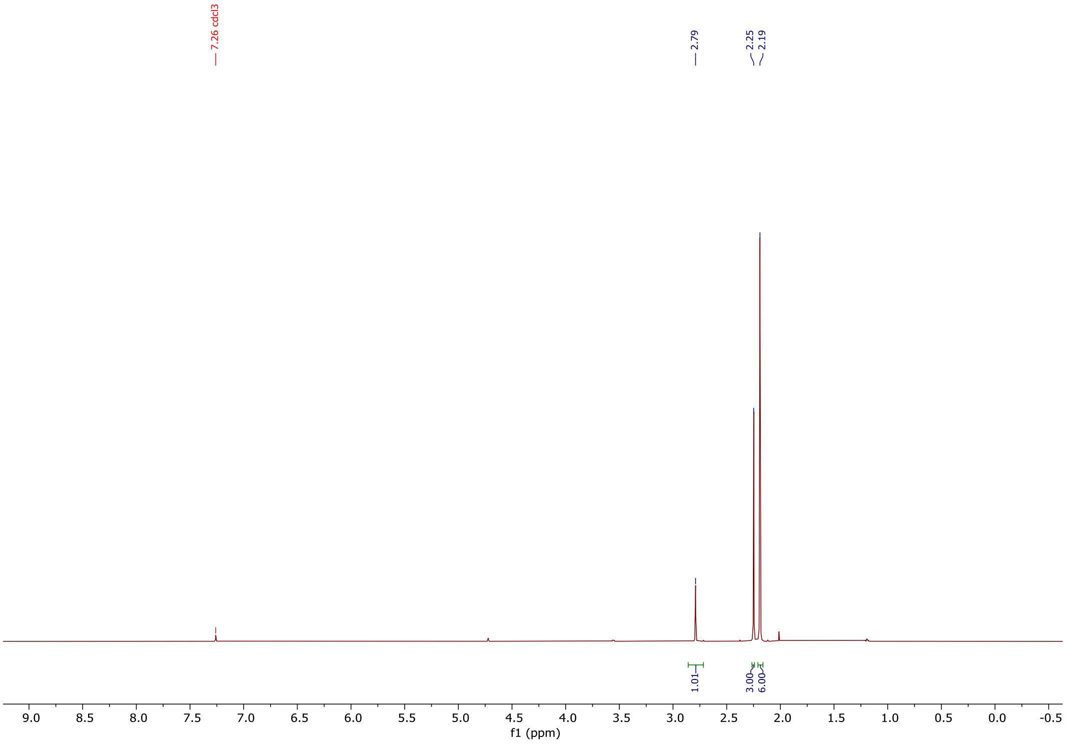

^{13}C NMR (126 MHz, $CDCl_3$)

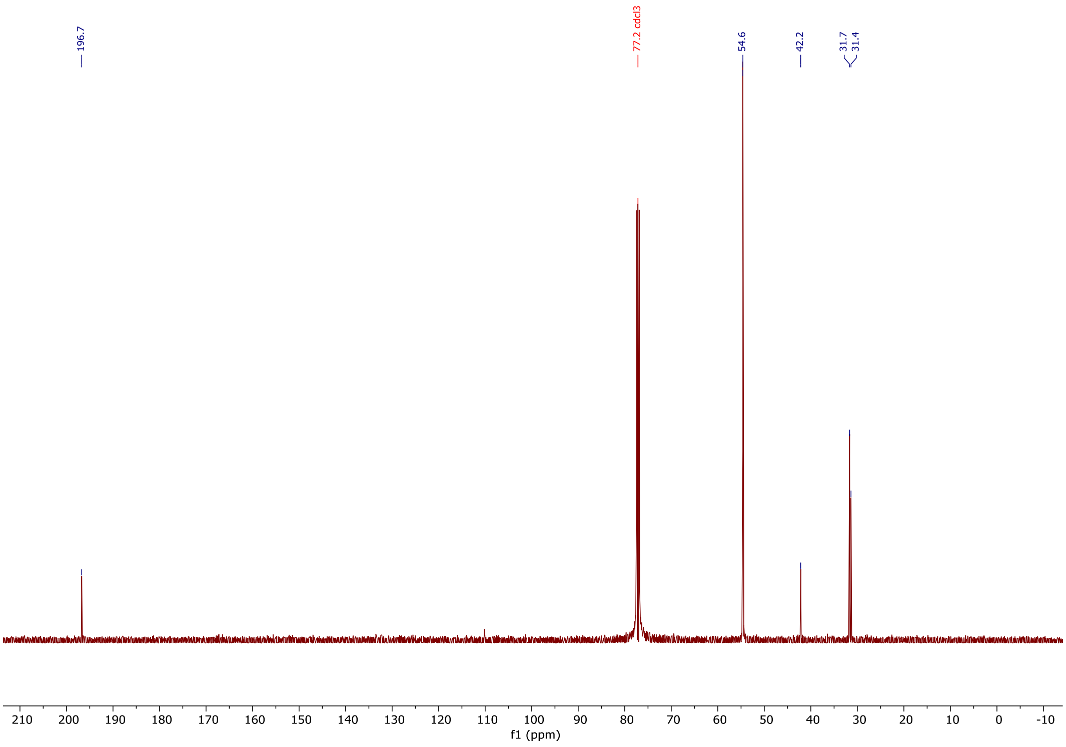

2-(Bicyclo[1.1.1]pentan-1-ylthio)pyrimidine, 6l2

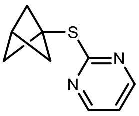

^{1}H NMR (500 MHz, DMSO-d_6)

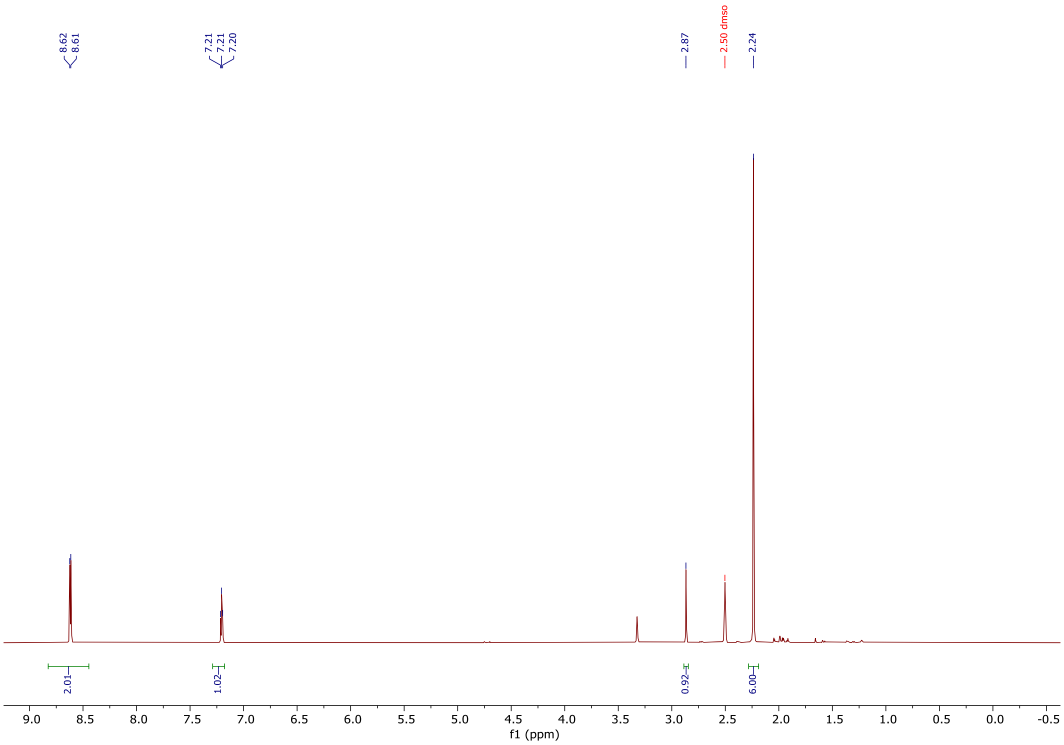

^{13}C NMR (151 MHz, DMSO-d_6)

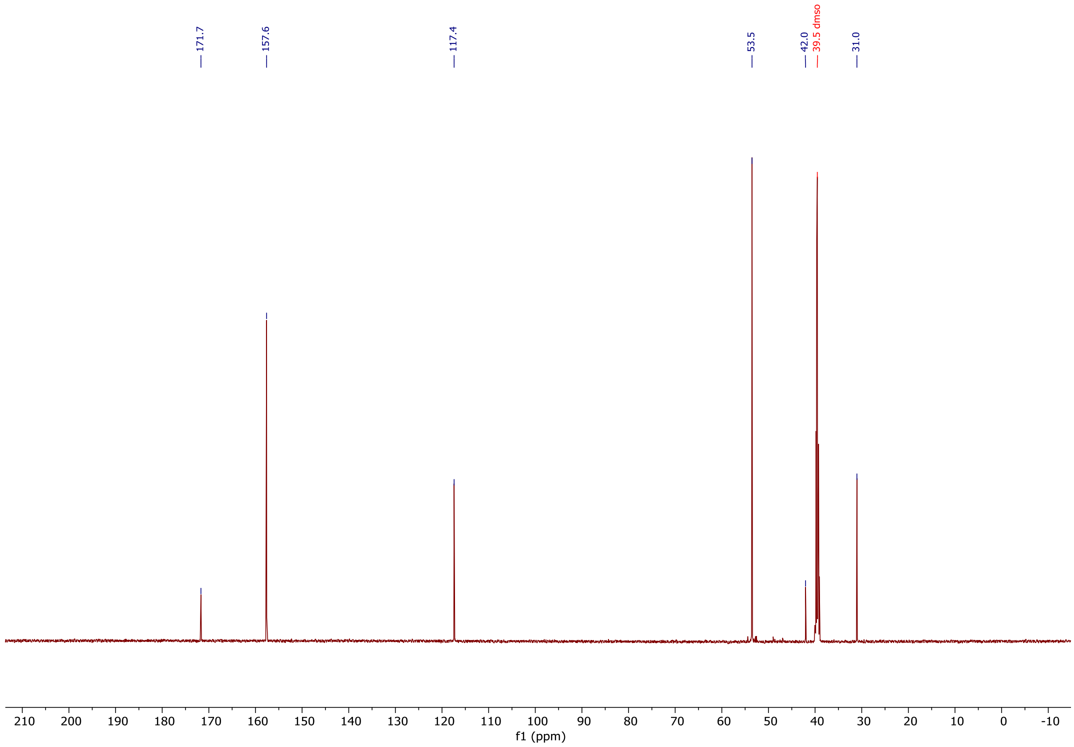

2-(Bicyclo[1.1.1]pentan-1-ylsulfonyl)pyrimidine, 6l3

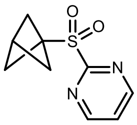

^{1}H NMR (500 MHz, DMSO-d_6)

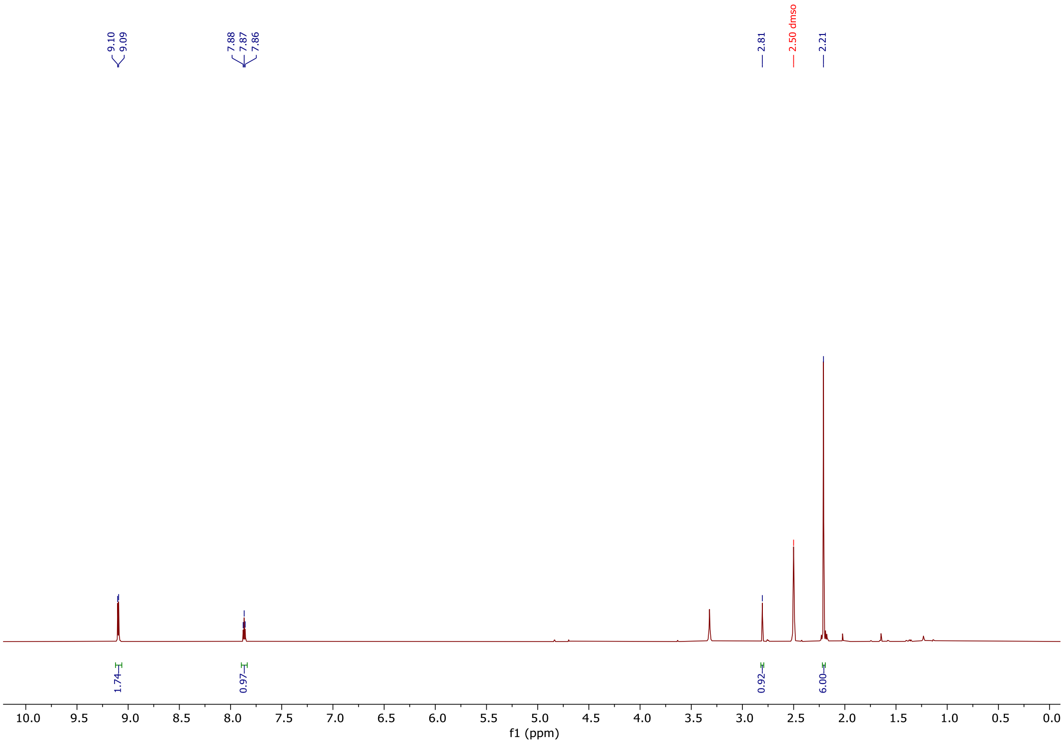

^{13}C NMR (151 MHz, DMSO-d_6)

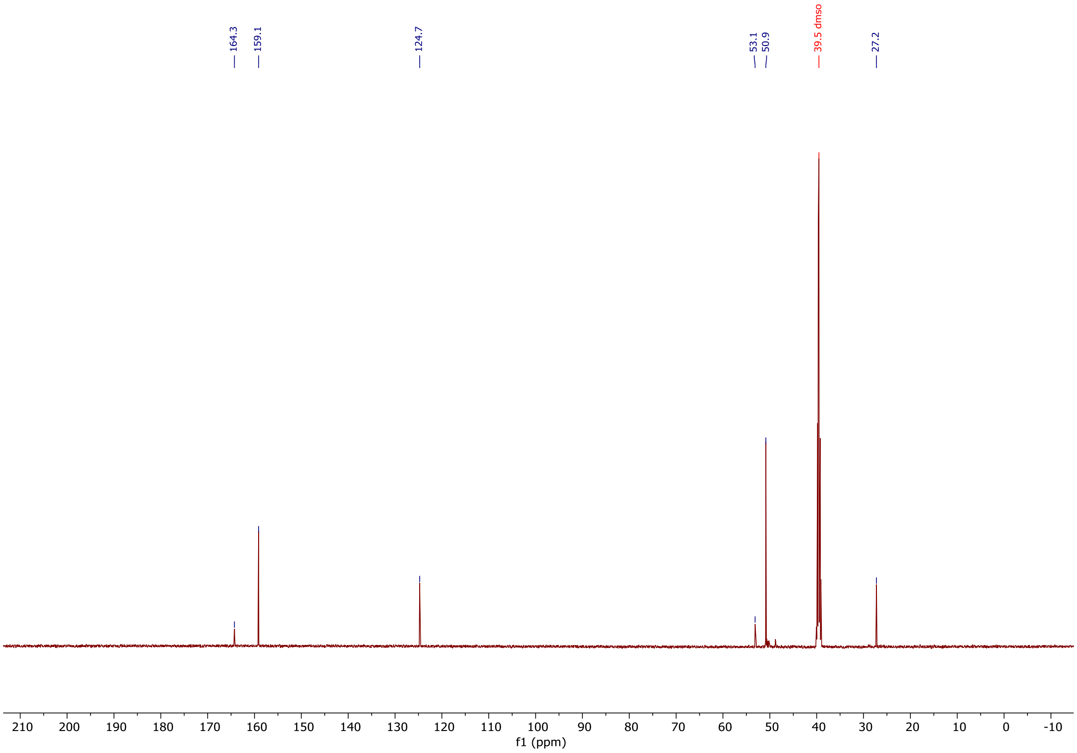

Sodium bicyclo[1.1.1]pentane-1-sulfinate, 6l

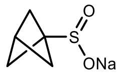

^{1}H NMR (500 MHz, DMSO-d6)

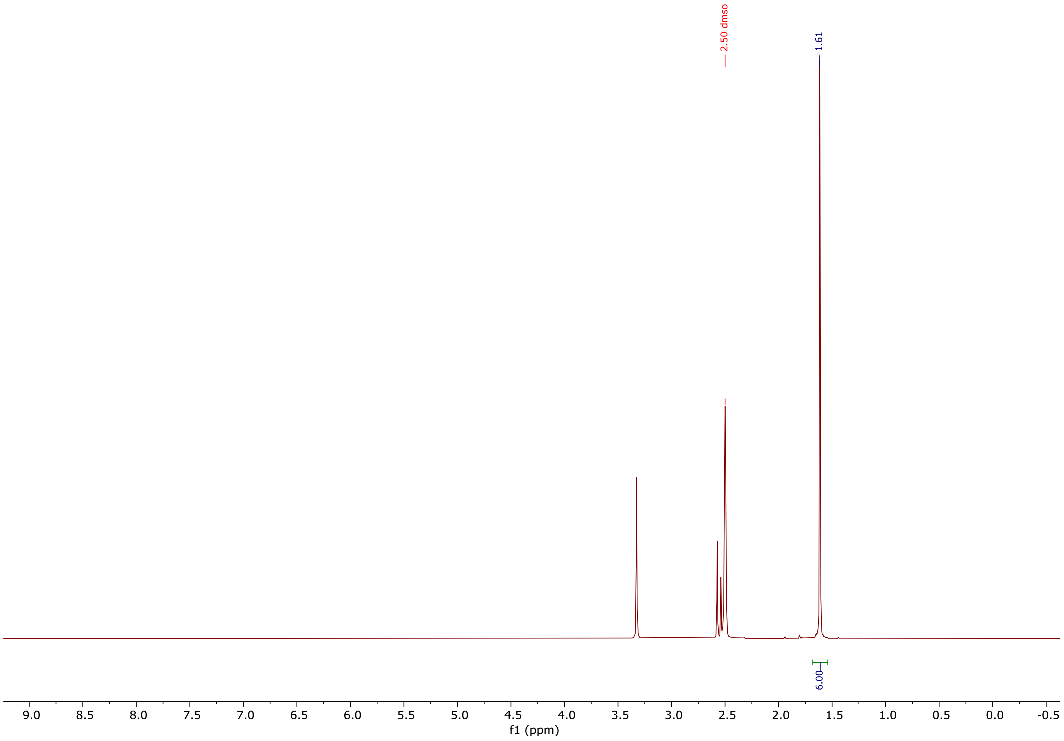

^{13}C NMR (151 MHz, DMSO-d6)

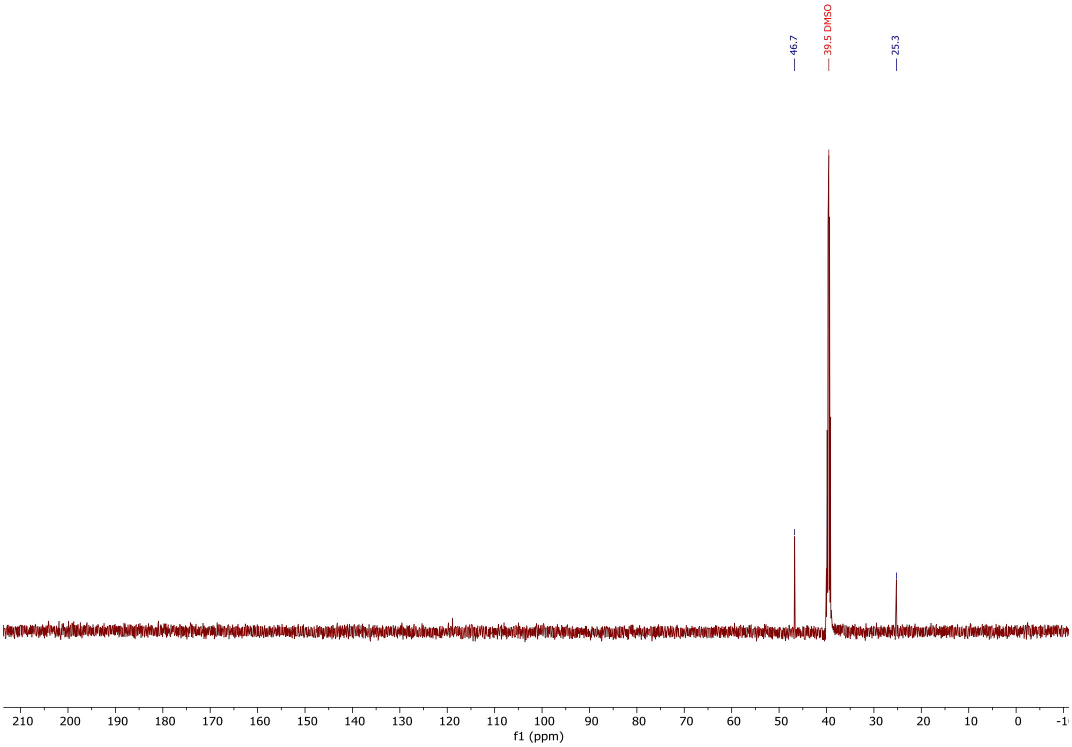

6.8 Unsuccessful Sulfinates

Sodium 2,4,6-triisopropylbenzenesulfinate

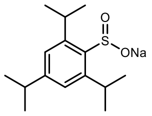

^{1}H NMR (600 MHz, MeOD)

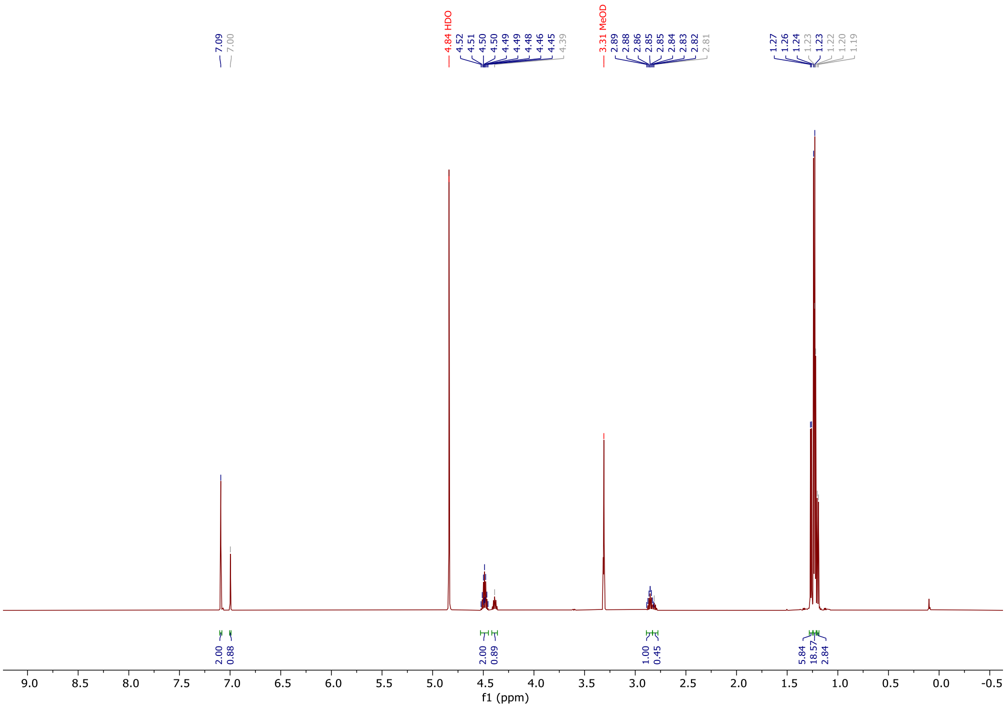

^{13}C NMR (151 MHz, MeOD)

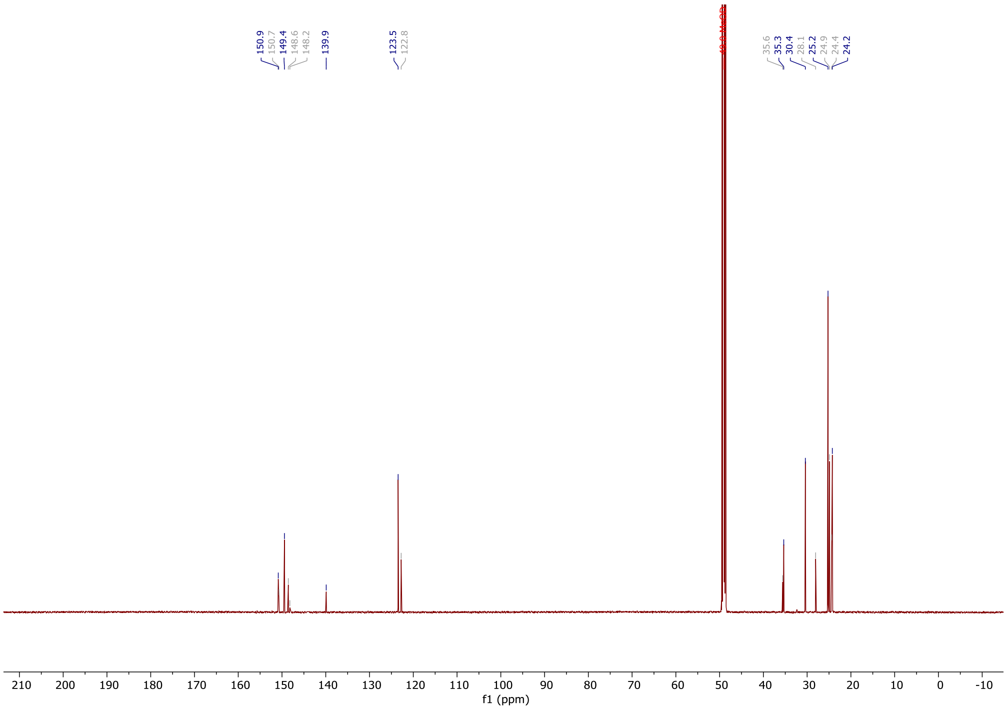

HMBC (600 MHz, MeOD)

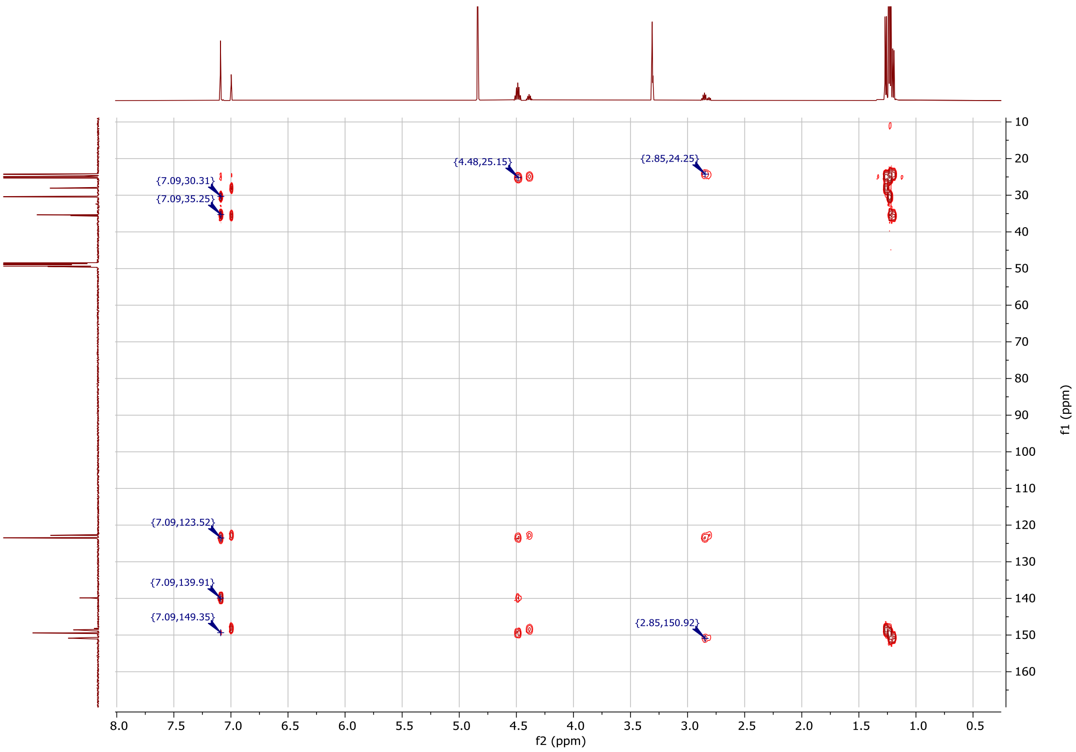

Sodium 2-nitrobenzenesulfinate

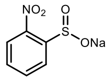

^{1}H NMR (101 MHz, MeOD)

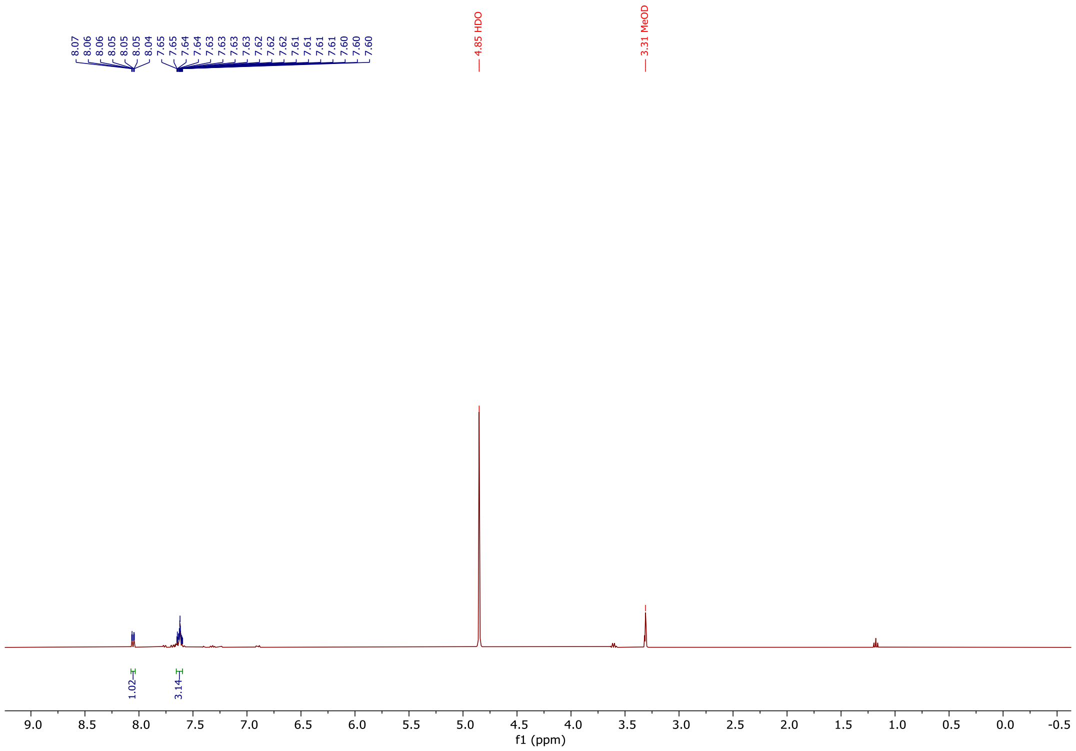

^{13}C NMR (101 MHz, MeOD)

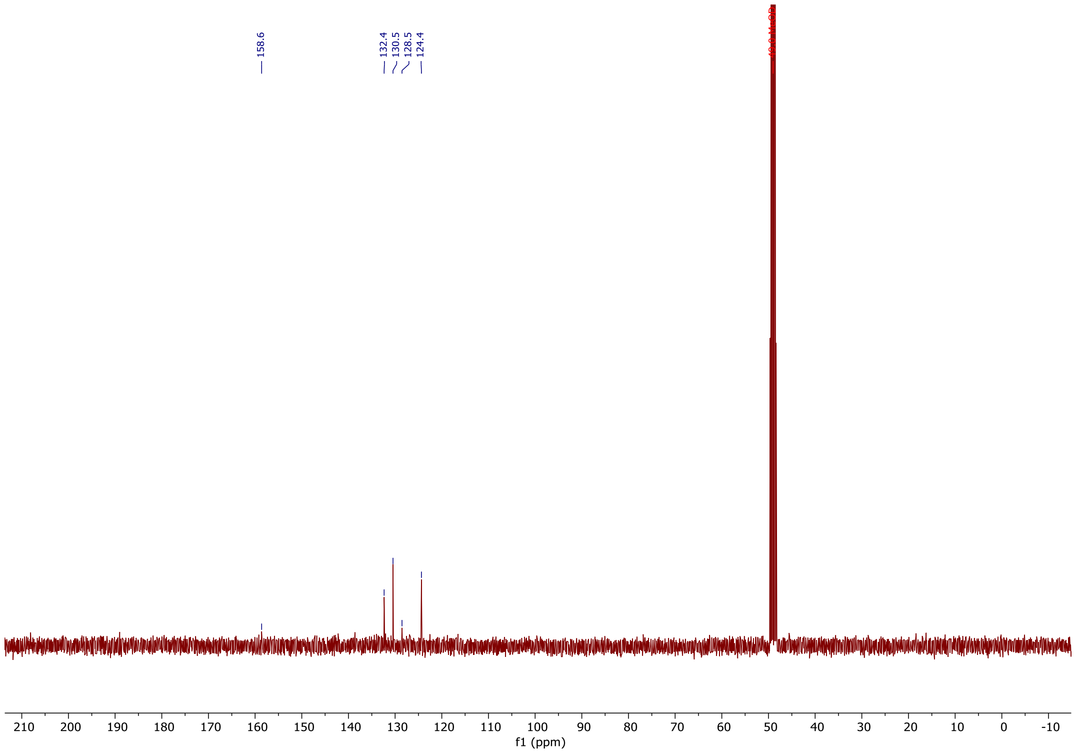

Sodium benzo[d]isothiazole-6-sulfinate

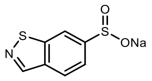

^{1}H NMR (600 MHz, MeOD)

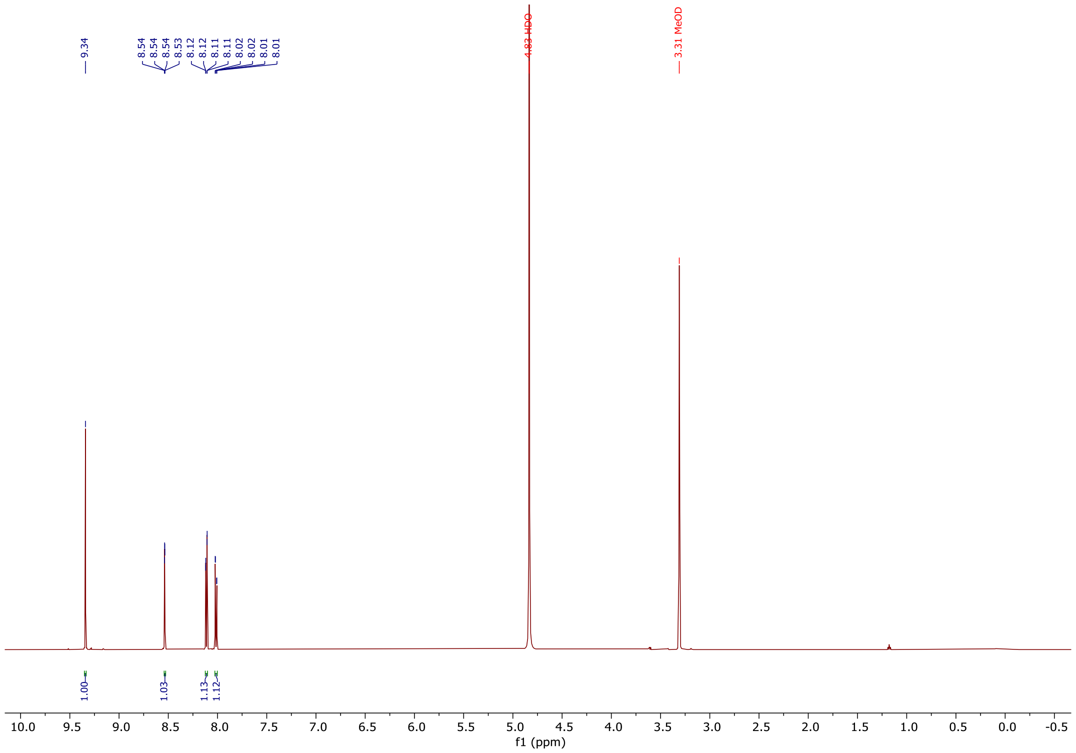

^{13}C NMR (151 MHz, MeOD)

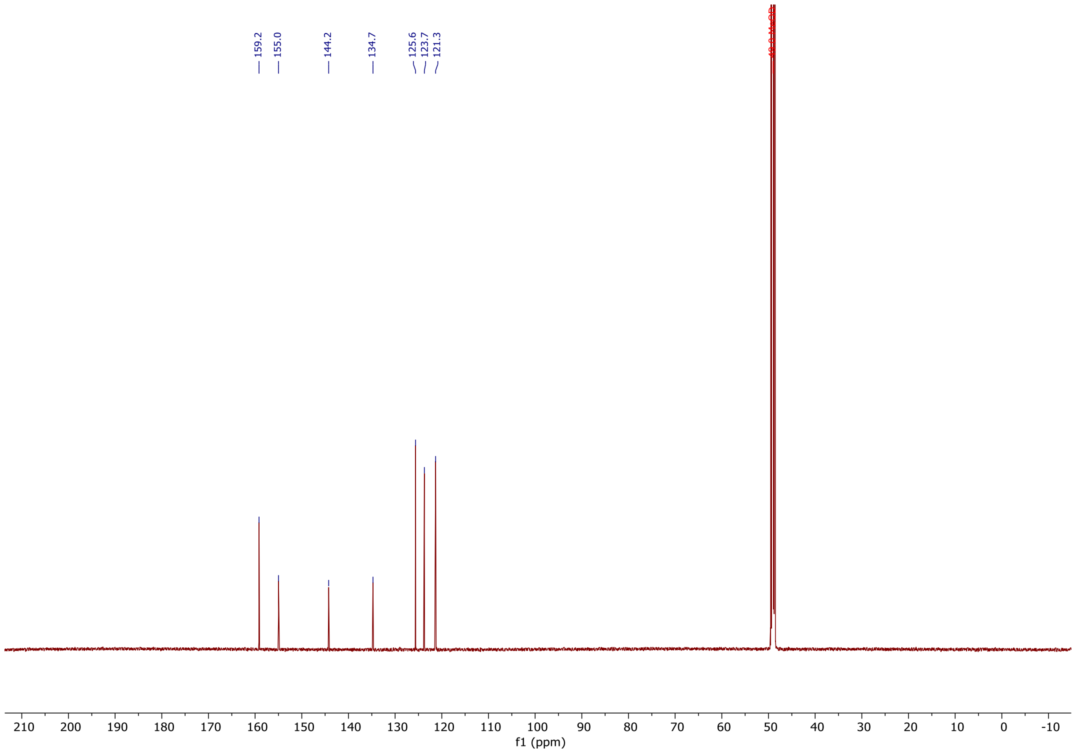

Sodium benzo[d]thiazole-2-sulfinate

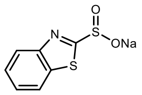

^{1}H NMR (400 MHz, MeOD)

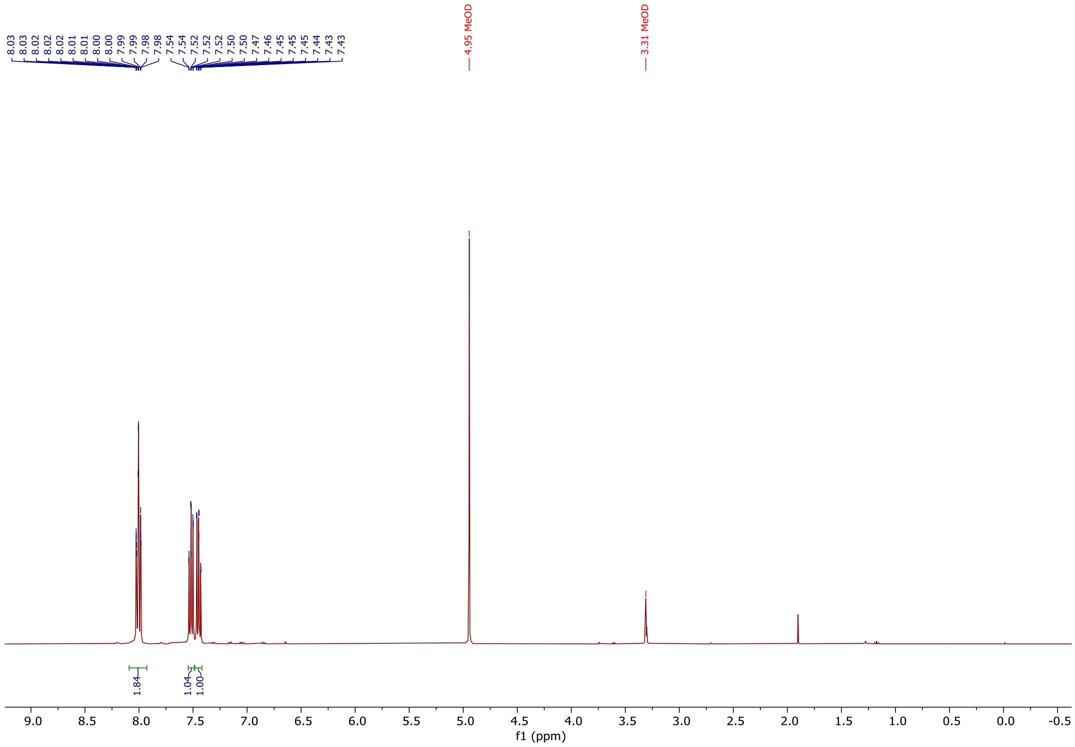

^{13}C NMR (101 MHz, MeOD)

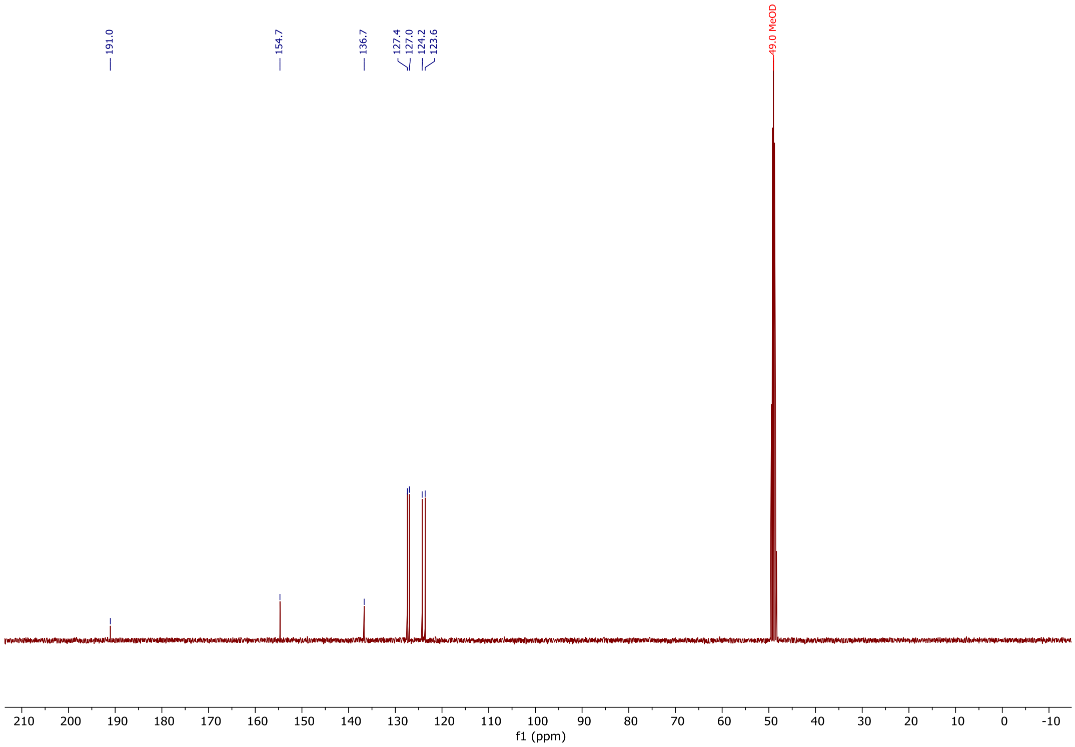

Sodium morpholine-4-sulfinate

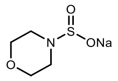

^{1}H NMR (400 MHz, MeOD)

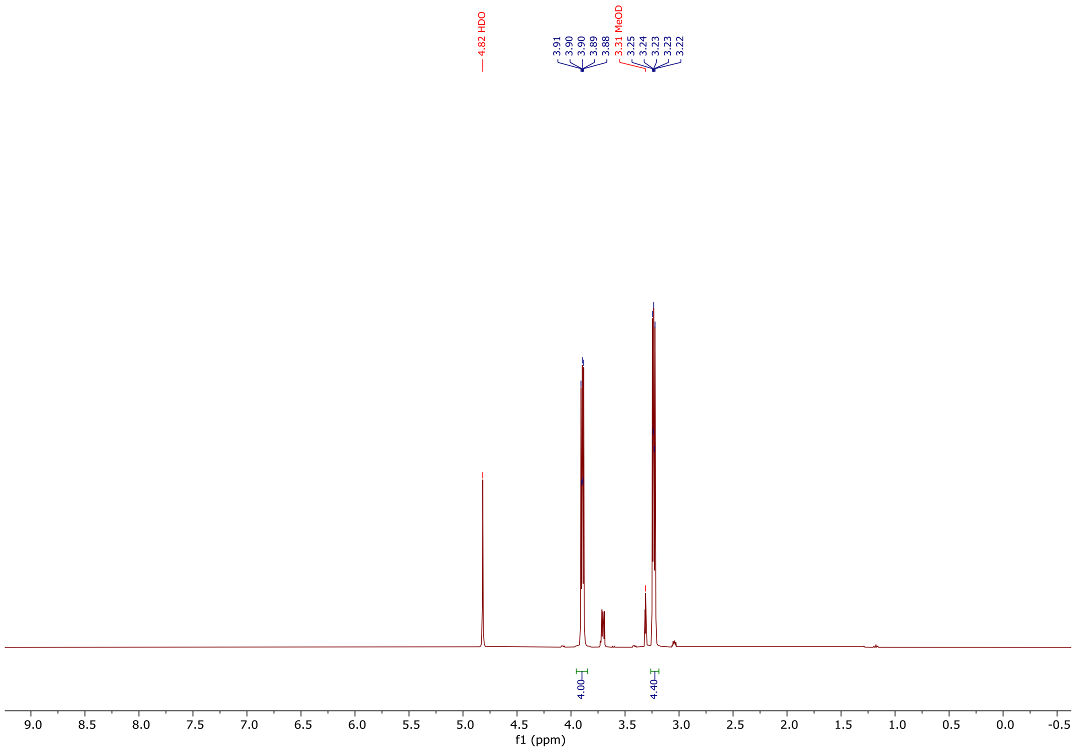

^{13}C NMR (101 MHz, MeOD)

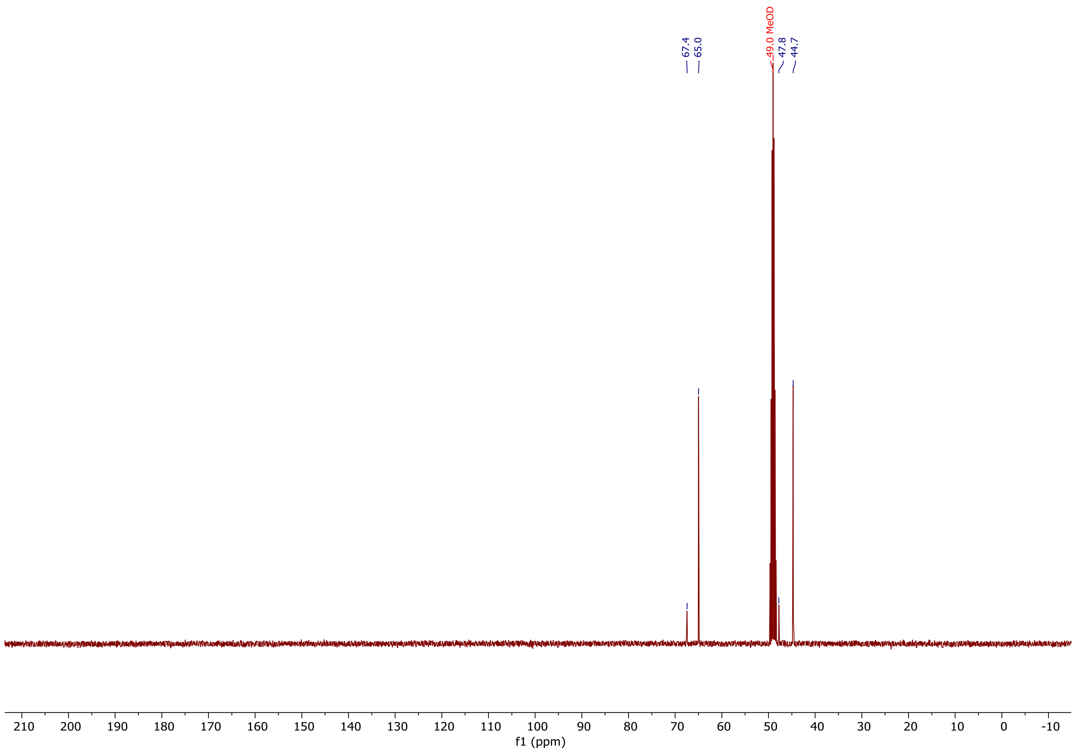

Sodium pyrrolidine-1-sulfinate

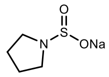

^{1}H NMR (400 MHz, MeOD)

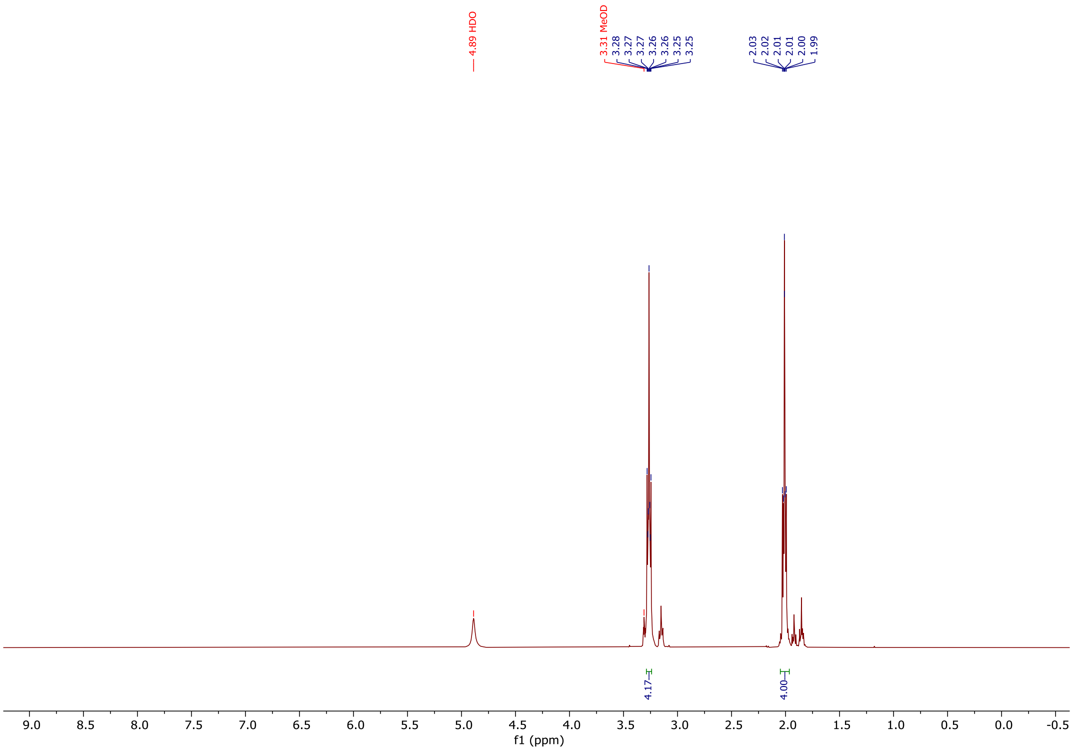

^{13}C NMR (101 MHz, MeOD)

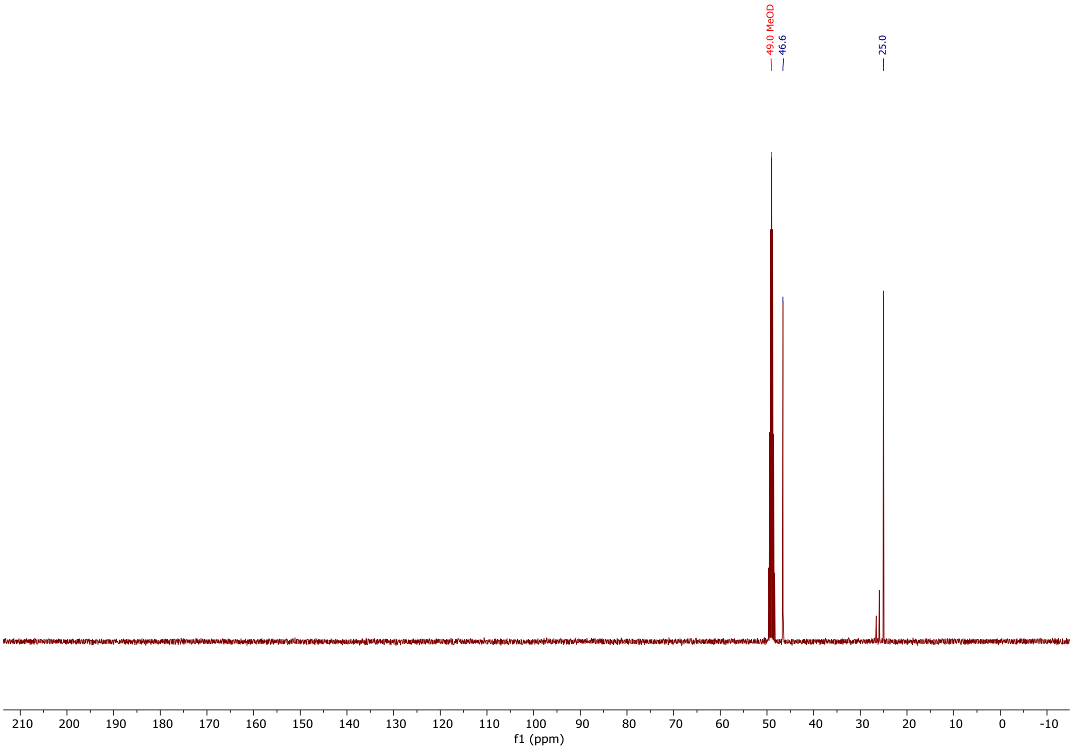

6.9 Functionalisation Products

2-((3-Bromobicyclo[1.1.1]pentan-1-yl)sulfonyl)acetic acid, 8

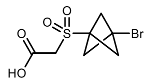

^{1}H NMR (500 MHz, $CDCl_3$)

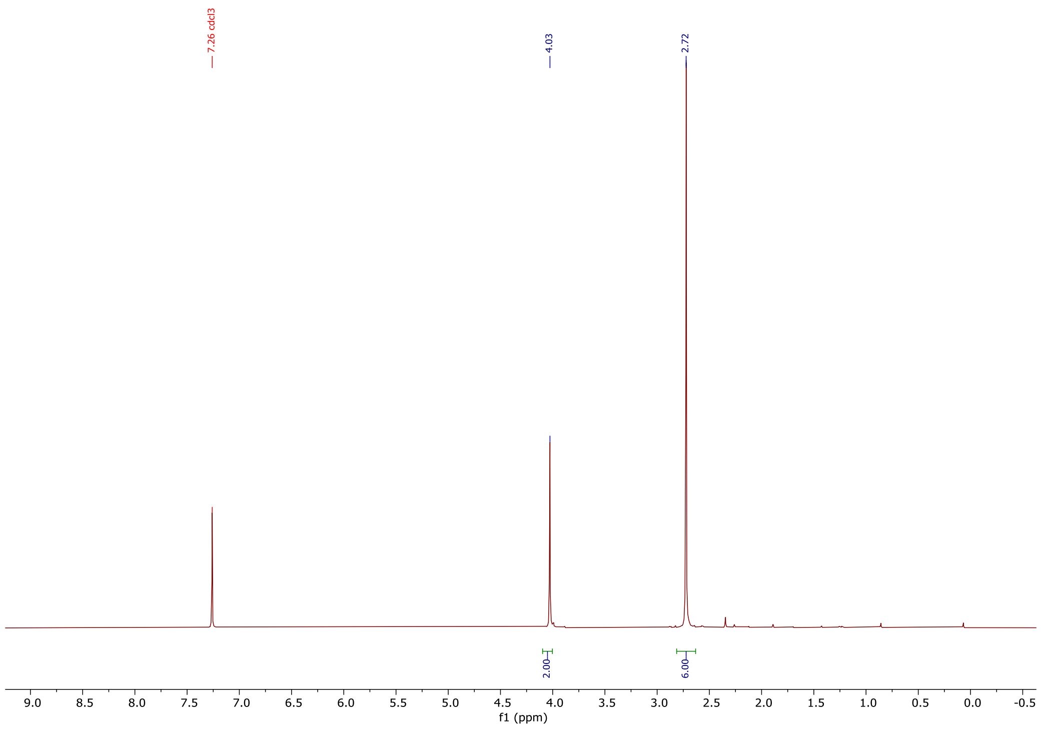

^{13}C NMR (126 MHz, $CDCl_3$)

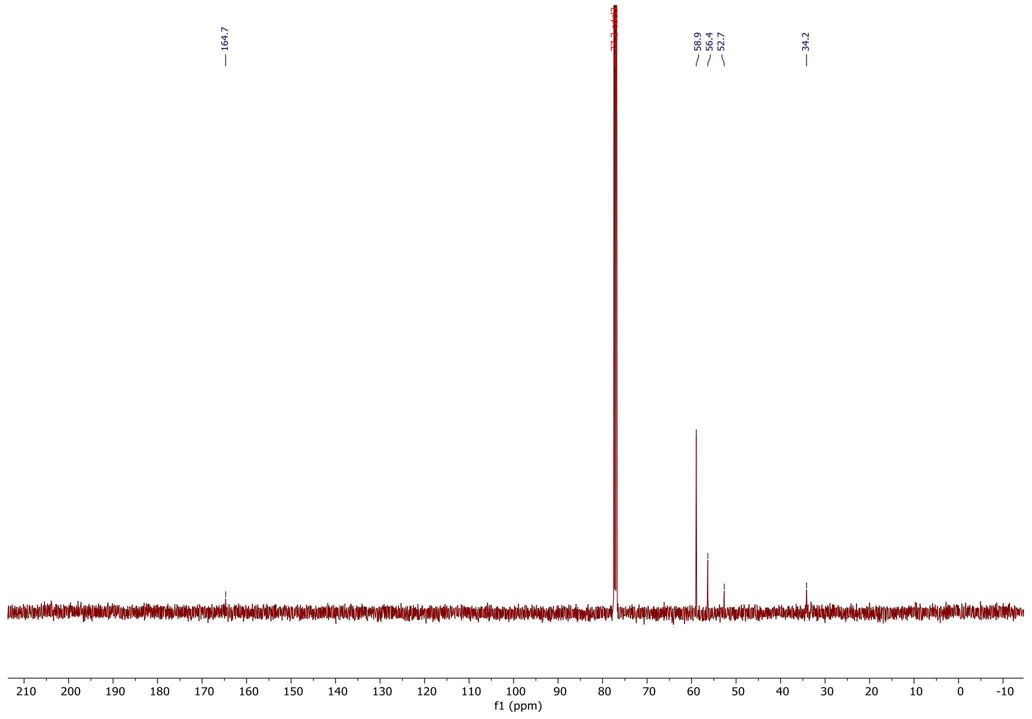

Methyl 2-(bis(tert-butoxycarbonyl)amino)-3-(3-tosylbicyclo[1.1.1]pentan-1-yl)propanoate, 11

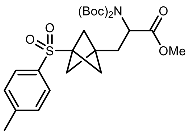

^{1}H NMR (600 MHz, $CDCl_3$)

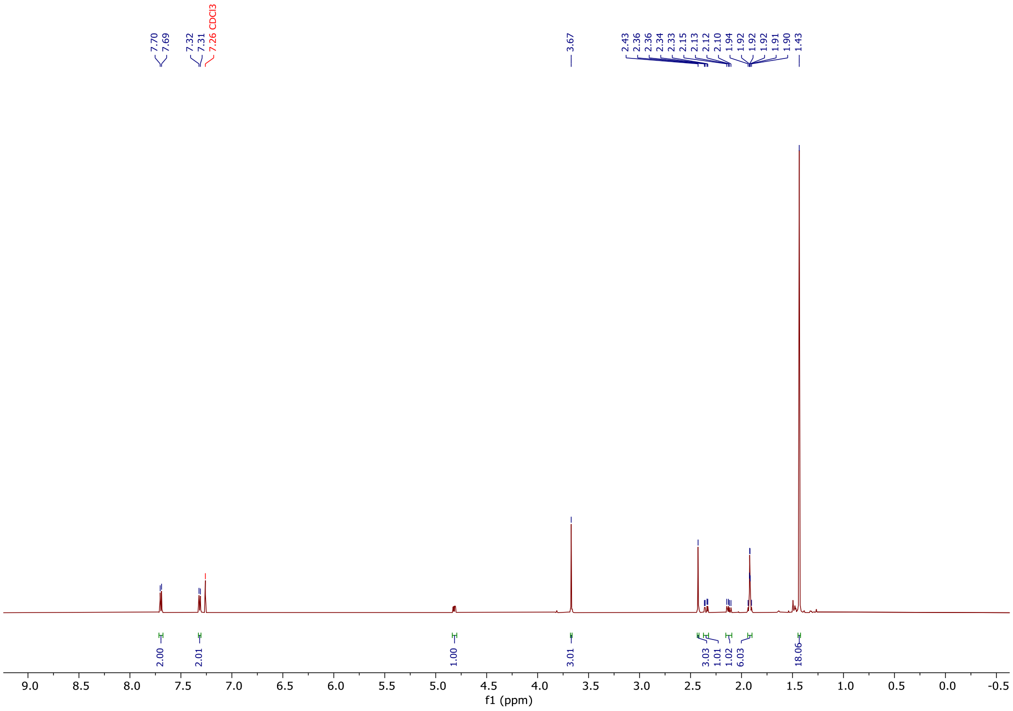

^{13}C NMR (151 MHz, $CDCl_3$)

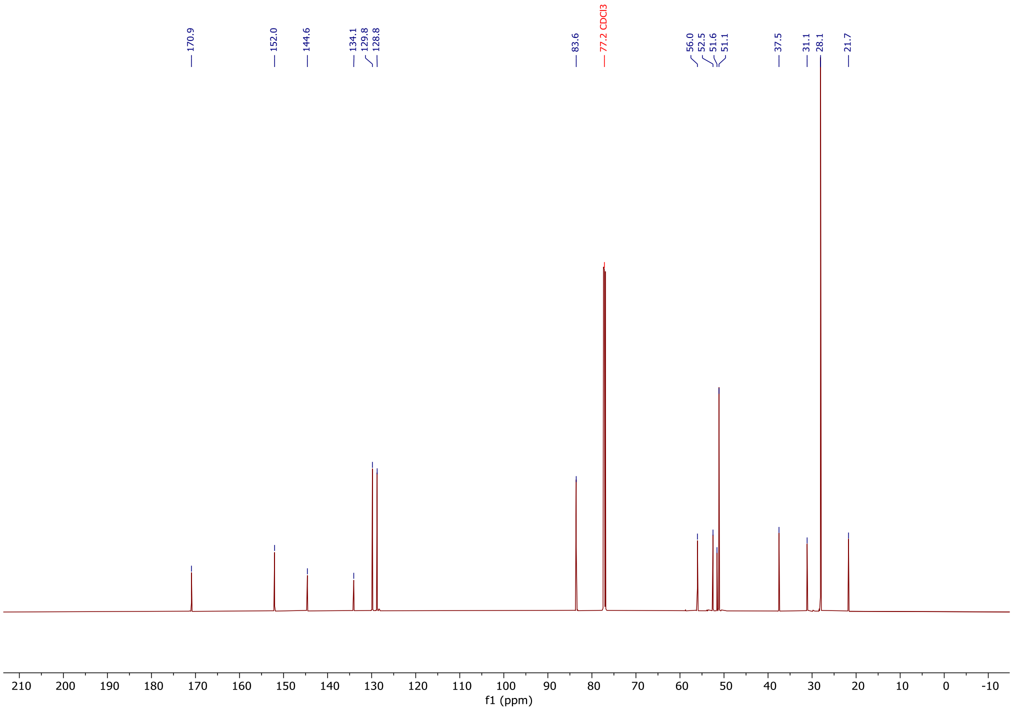

6.10 Miscellaneous Compounds

1-Phenylsulfonyl)bicyclo[1.1.1]pentane, S4

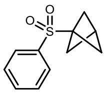

^{1}H **NMR** (500 MHz, $CDCl_3$)

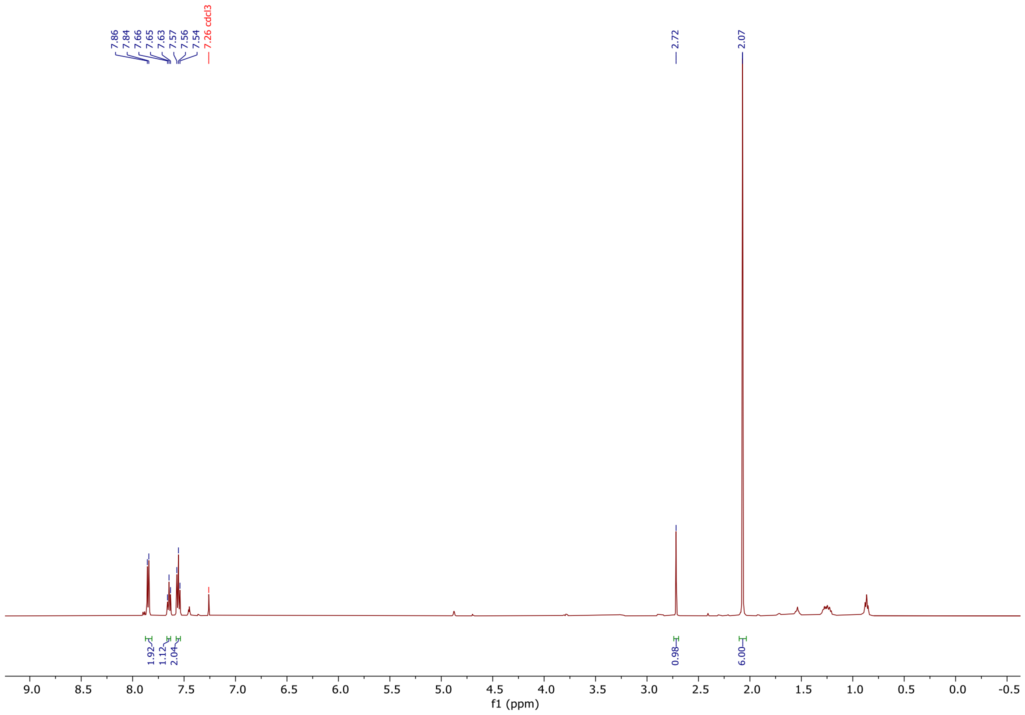

^{13}C **NMR** (126 MHz, $CDCl_3$)

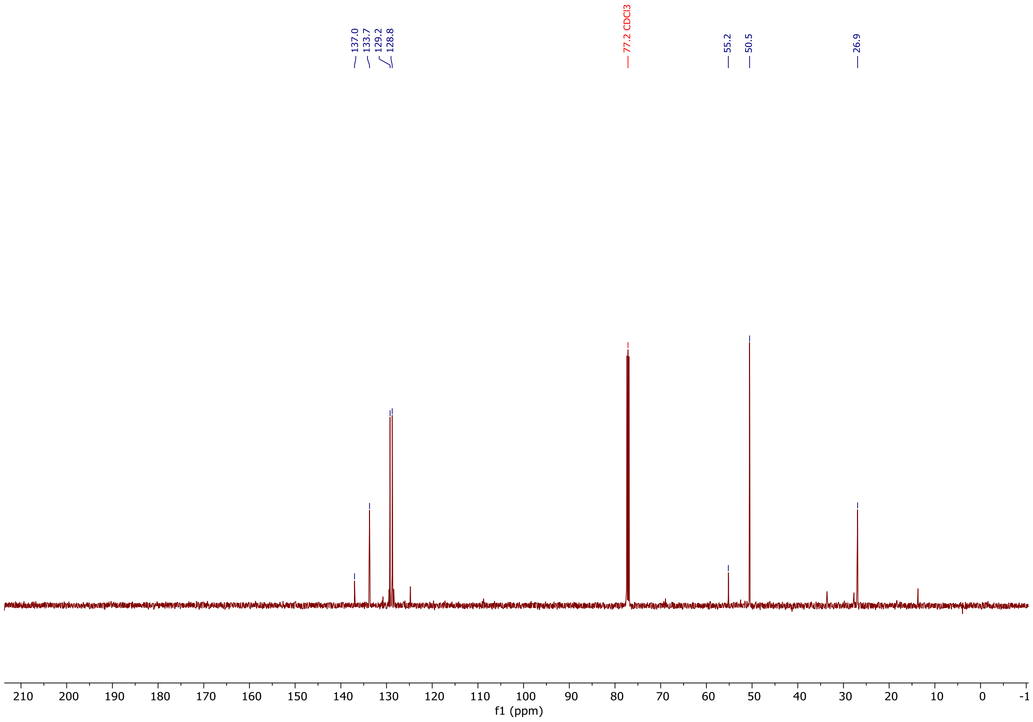

3-(*tert*-Butyl)bicyclo[1.1.1]pentane-1-carboxylic acid, S5 (crude)

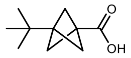

Crude: ^{1}H NMR (500 MHz, $CDCl_3$)

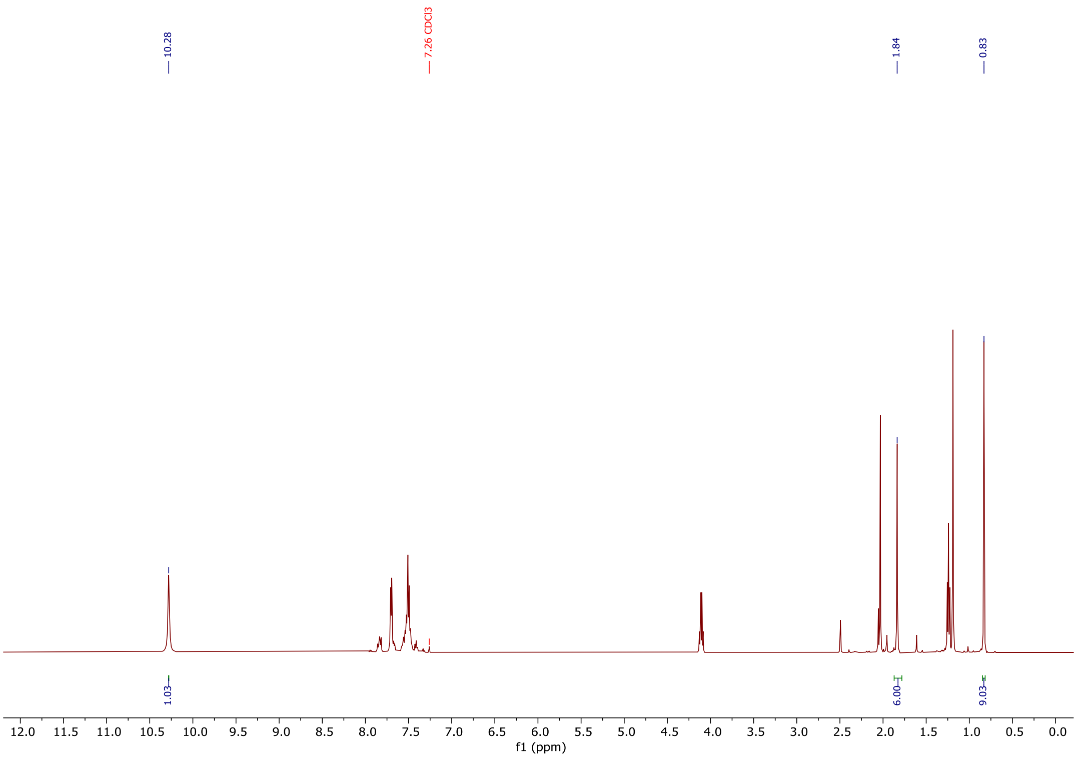

Crude: ^{13}C NMR (126 MHz, $CDCl_3$)

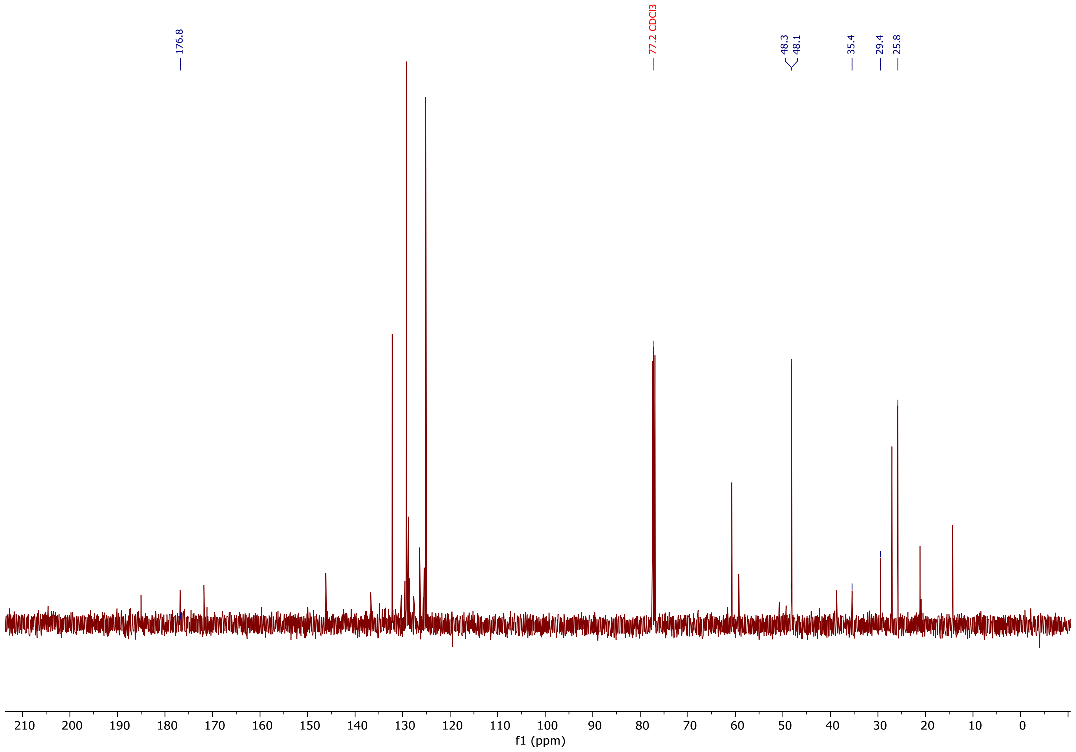

^{1}H NMR (500 MHz, CDCl$_3$)

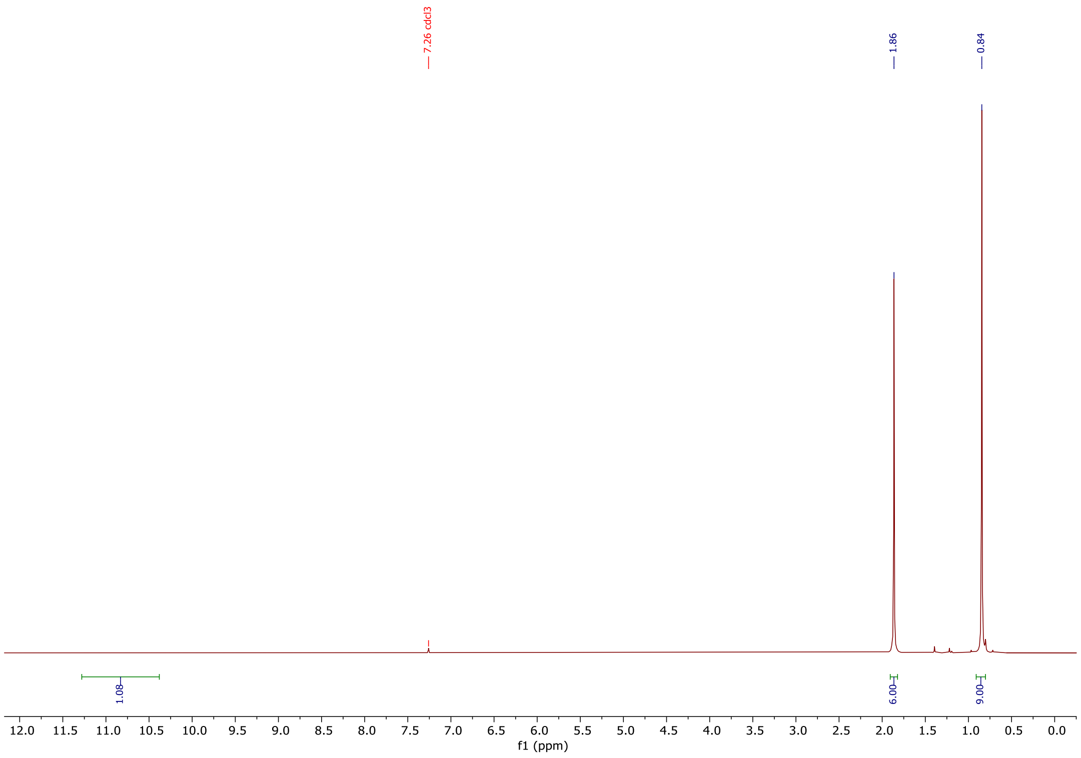

^{13}C NMR (126 MHz, CDCl$_3$)

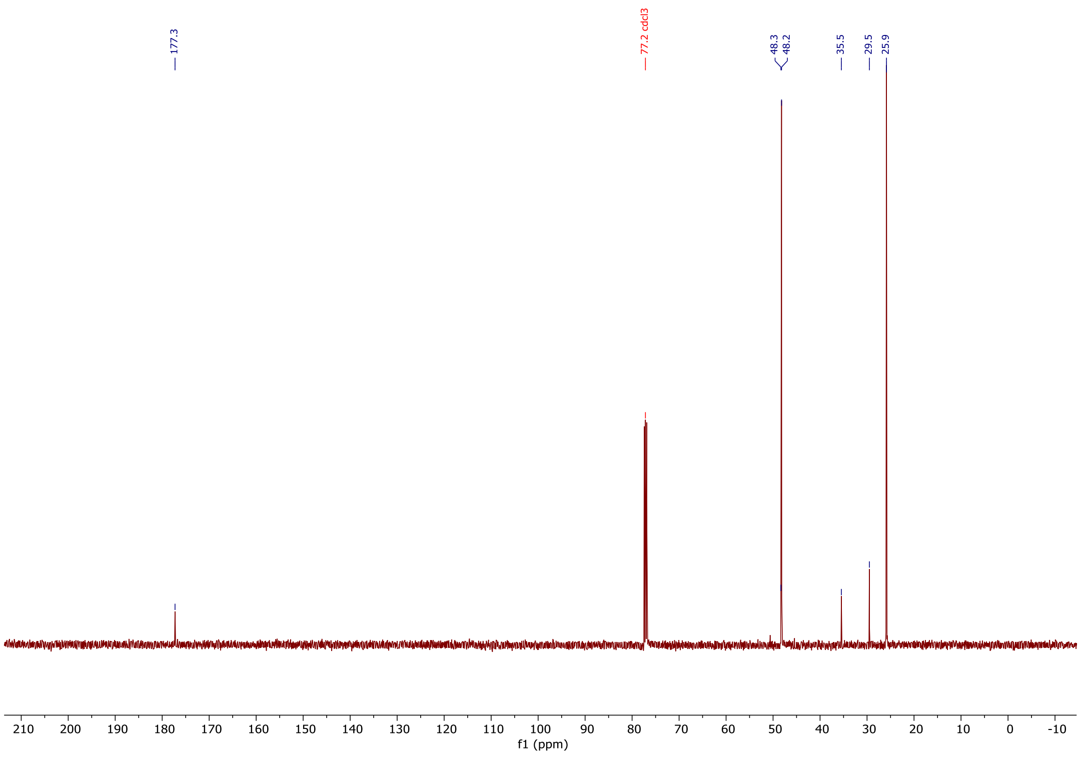

6.11 Phosphonate BCP Halides

Diethyl (3-bromobicyclo[1.1.1]pentan-1-yl)phosphonate, 12

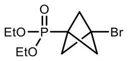

^{1}**H NMR** (500 MHz, $CDCl_3$)

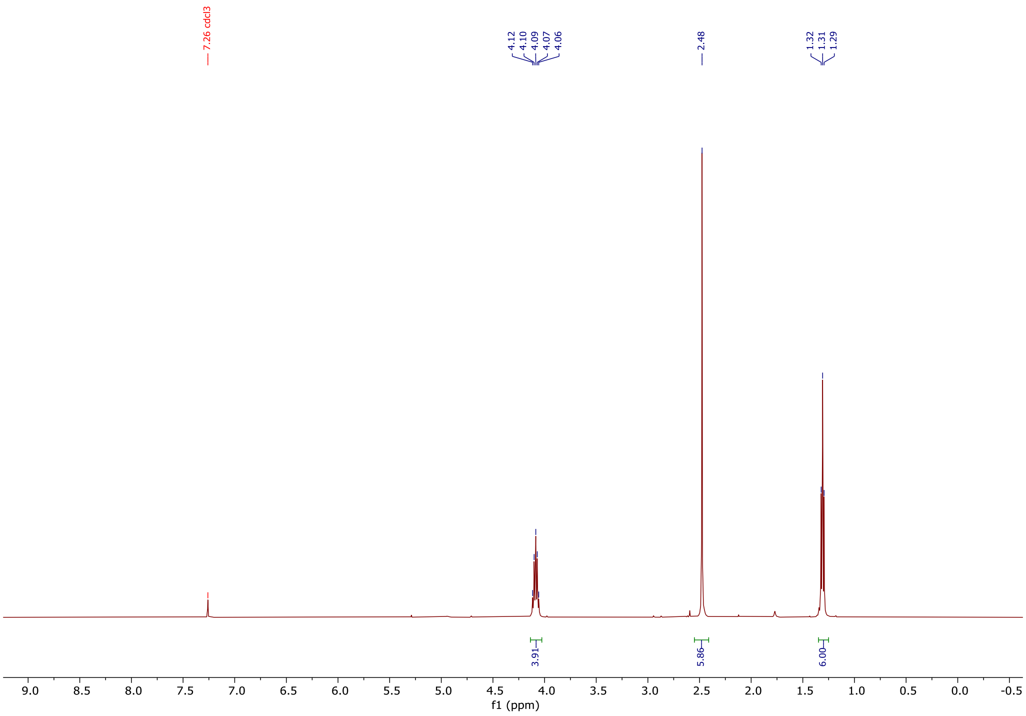

^{13}**C NMR** (126 MHz, $CDCl_3$)

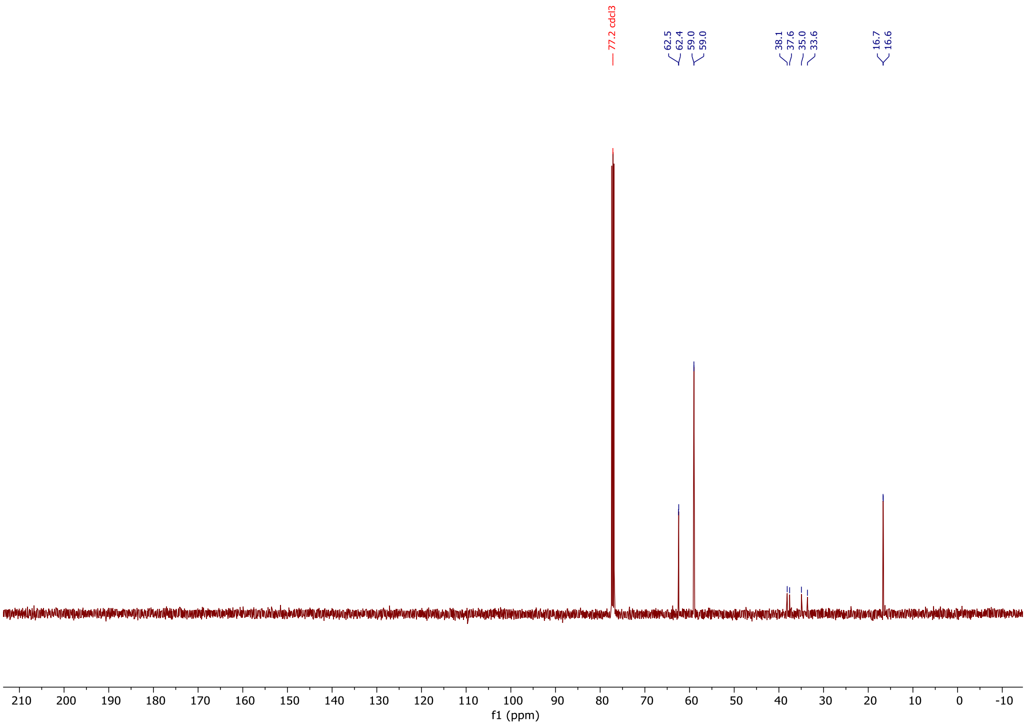

^{31}**P {^{1}H} NMR** (202 MHz, $CDCl_3$)

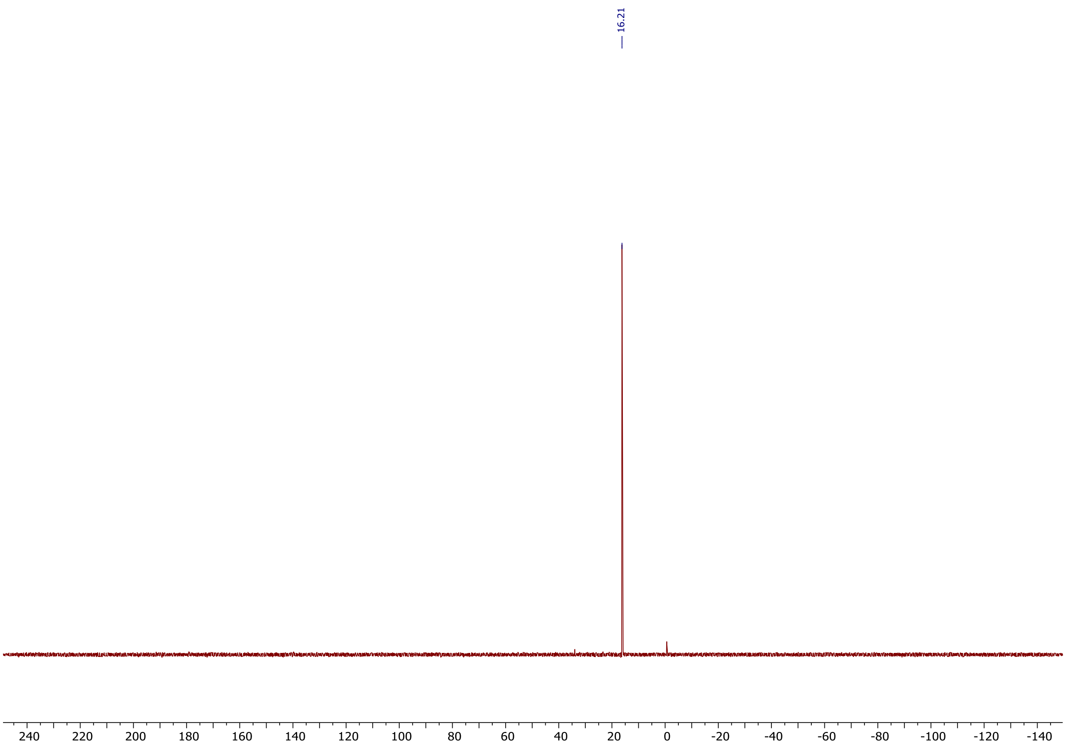

(3-Bromobicyclo[1.1.1]pentan-1-yl)dimethylphosphine oxide, 13

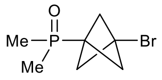

^{1}H NMR (500 MHz, DMSO-d$_6$)

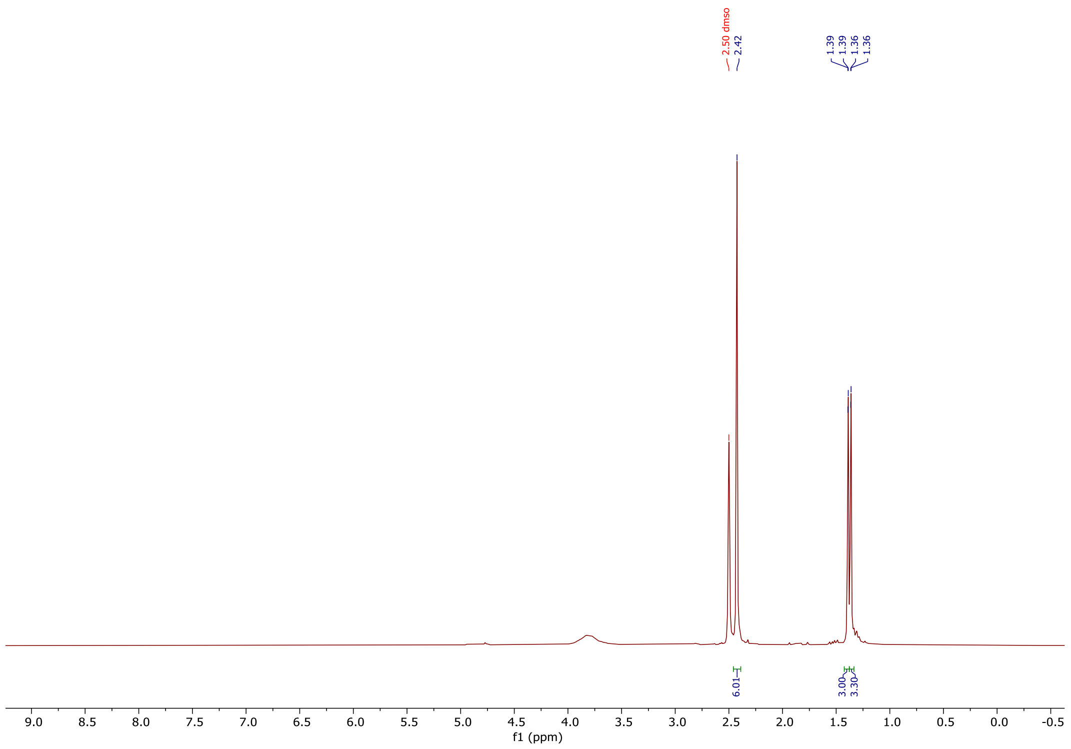

^{13}C NMR (126 MHz, DMSO-d$_6$)

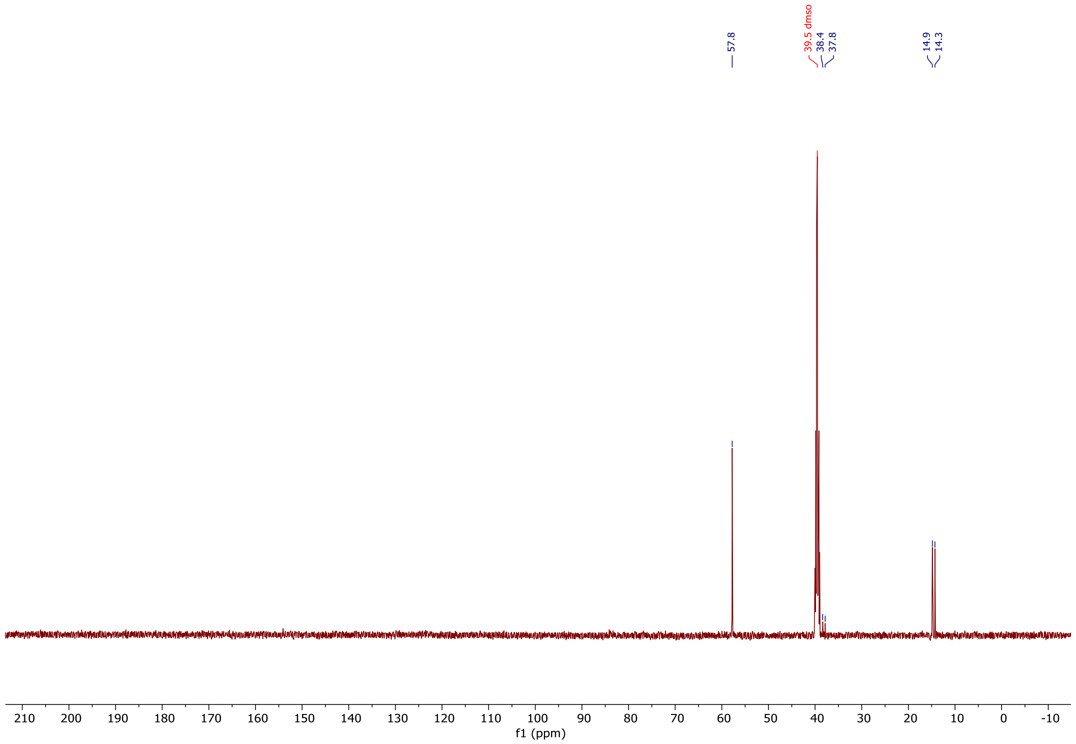

^{31}P {^{1}H} NMR (202 MHz, DMSO-d_6)

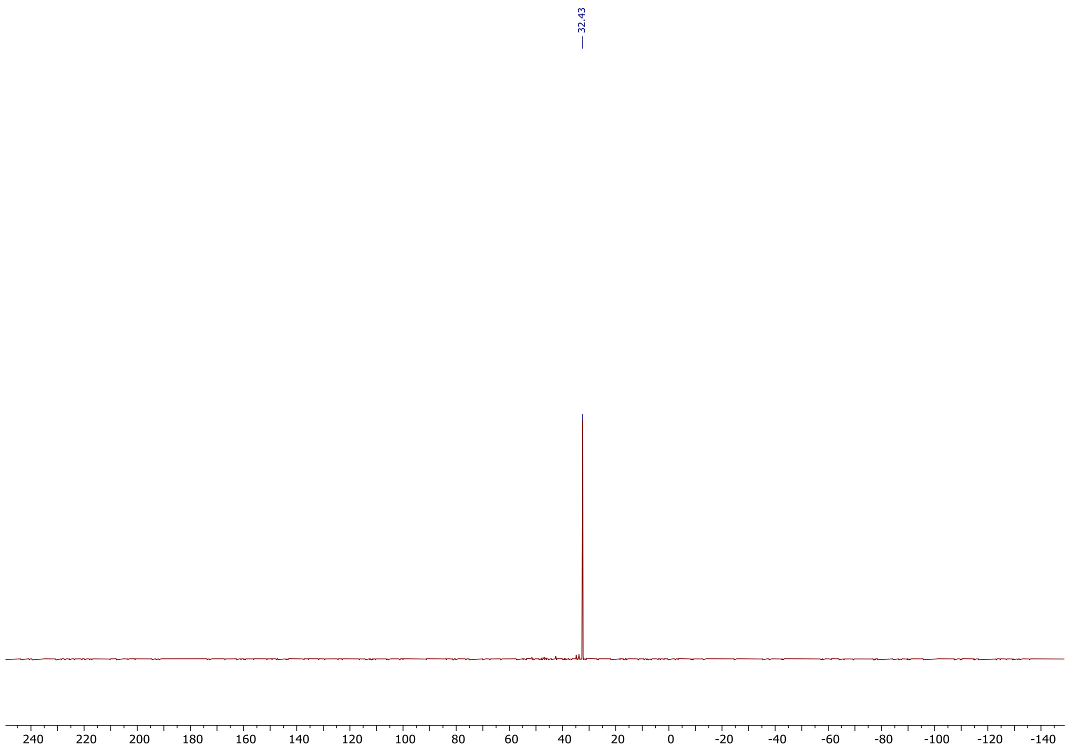

3-(Diethoxyphosphoryl)bicyclo[1.1.1]pentane-1-carboxylic acid, 14

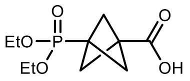

^{1}H NMR (500 MHz, $CDCl_3$)

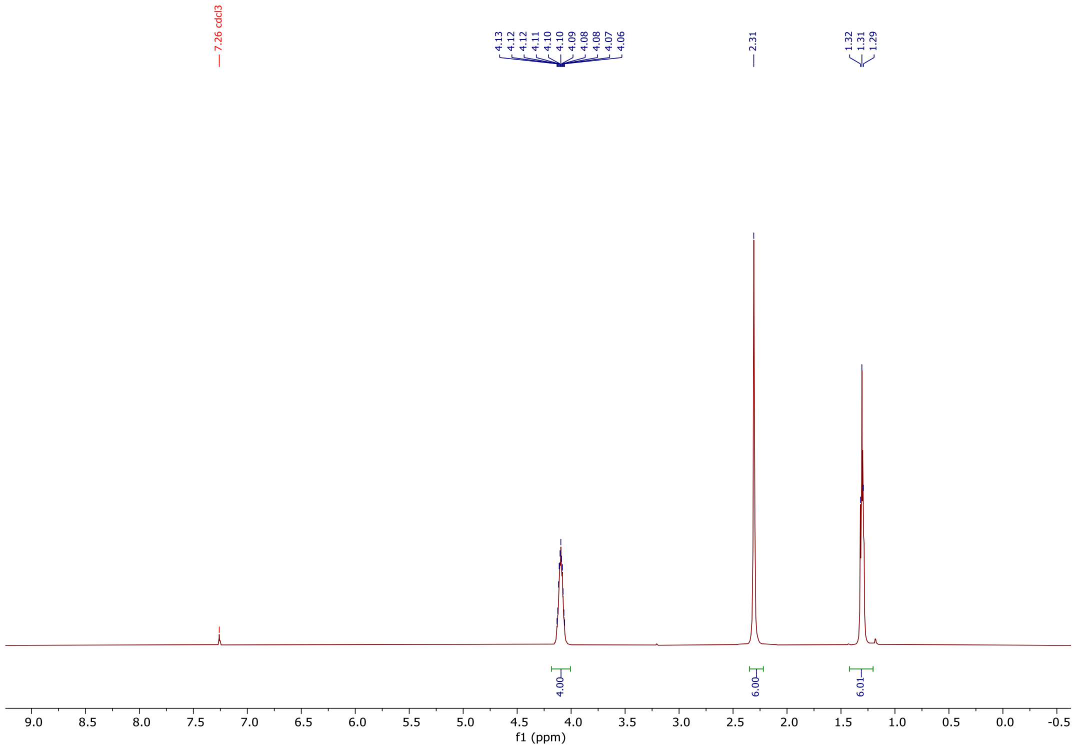

^{13}C NMR (126 MHz, $CDCl_3$)

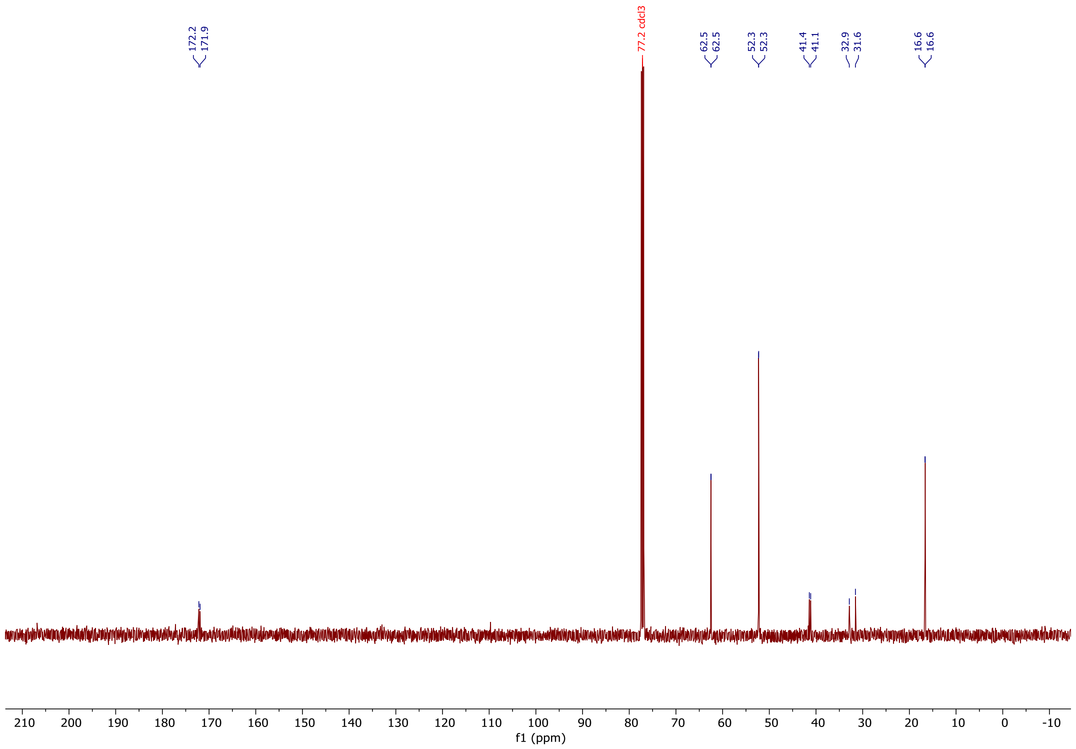

^{31}P {^{1}H} NMR (202 MHz, $CDCl_3$)

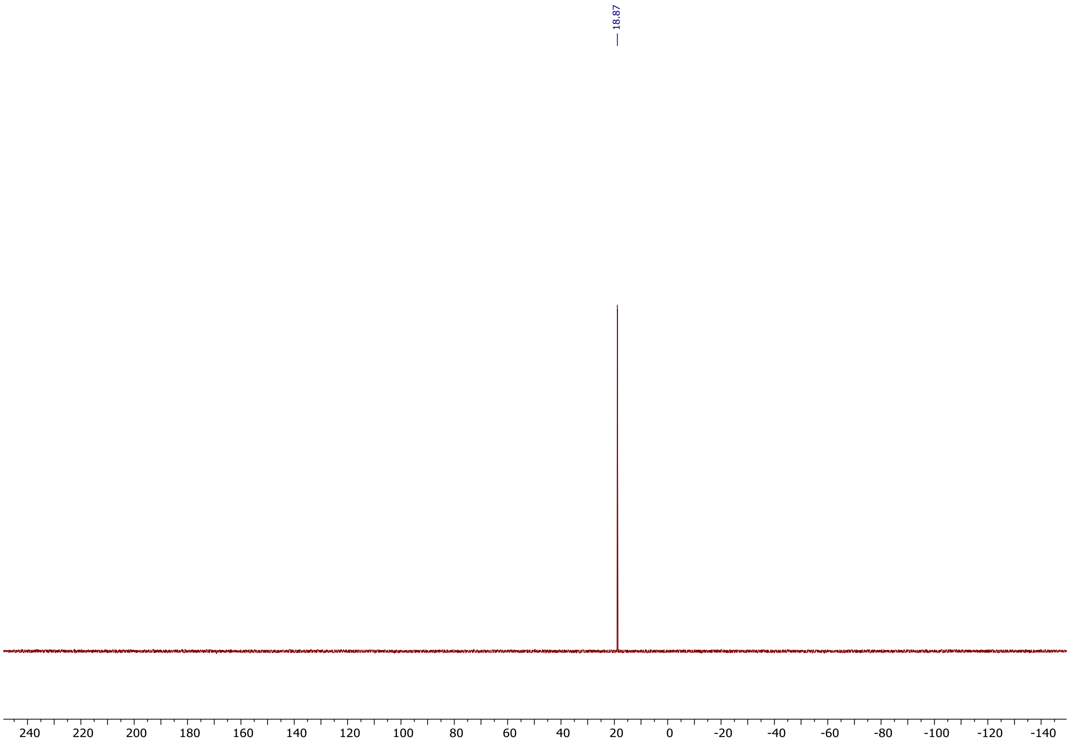

tert-Butyl (3-(diethoxyphosphoryl)bicyclo[1.1.1]pentan-1-yl)carbamate, 15

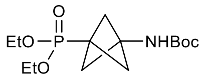

^{1}H NMR (500 MHz, $CDCl_3$)

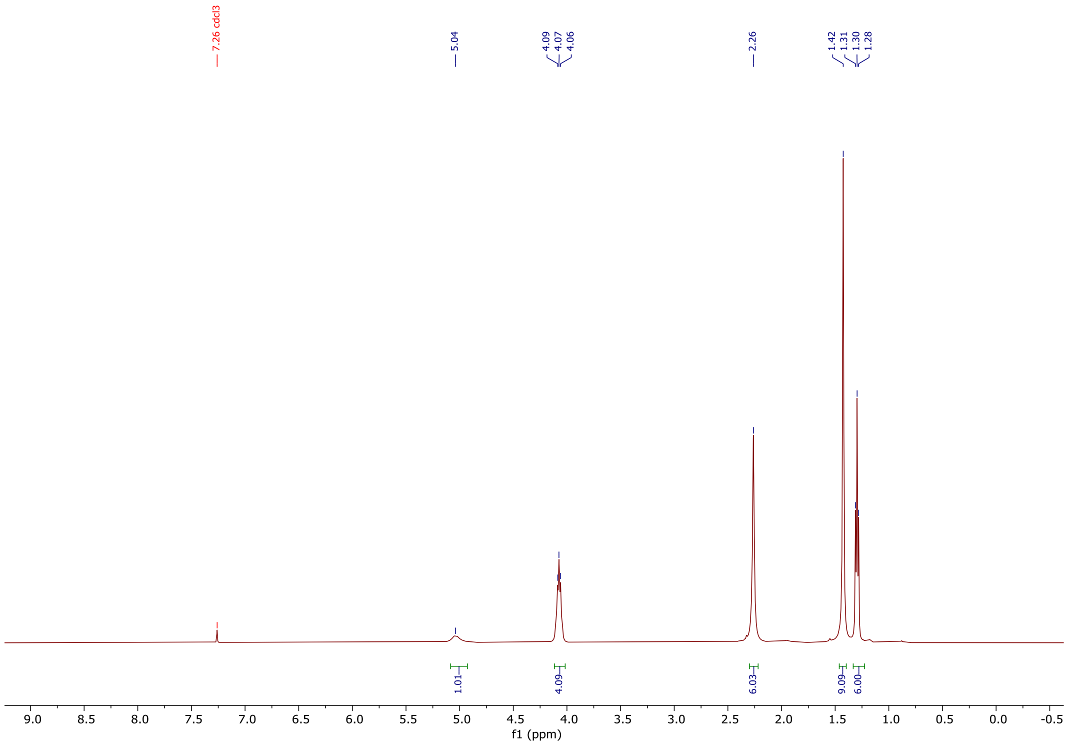

^{13}C NMR (151 MHz, $CDCl_3$)

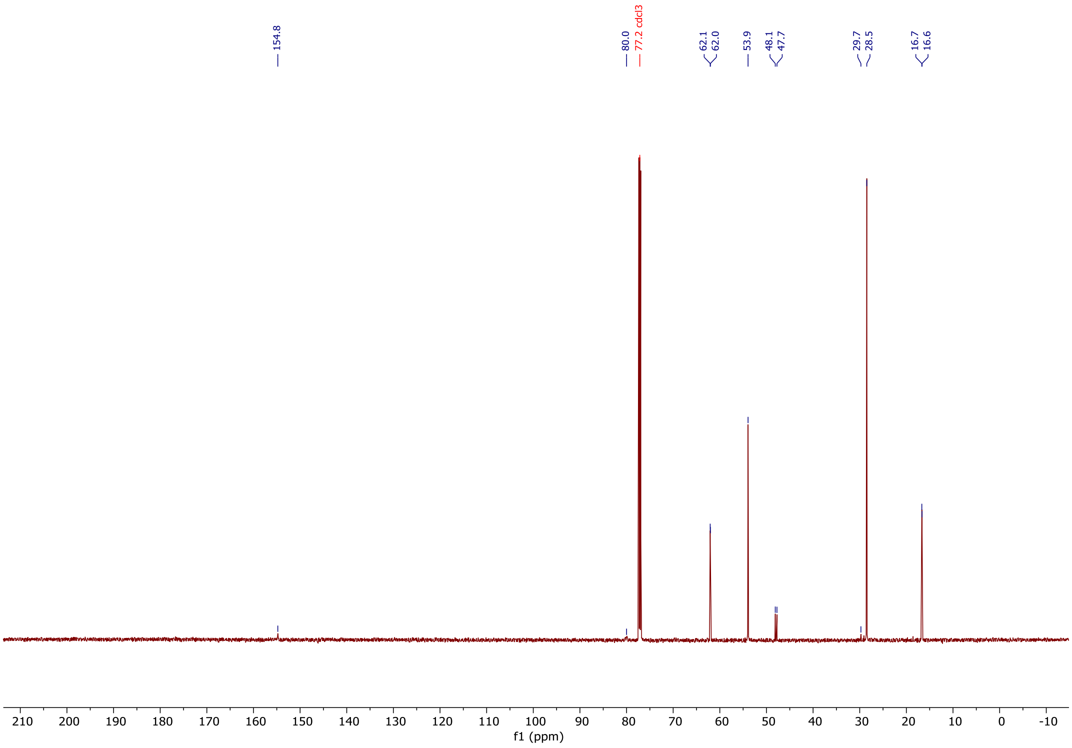

^{31}P {^{1}H} NMR (162 MHz, $CDCl_3$)

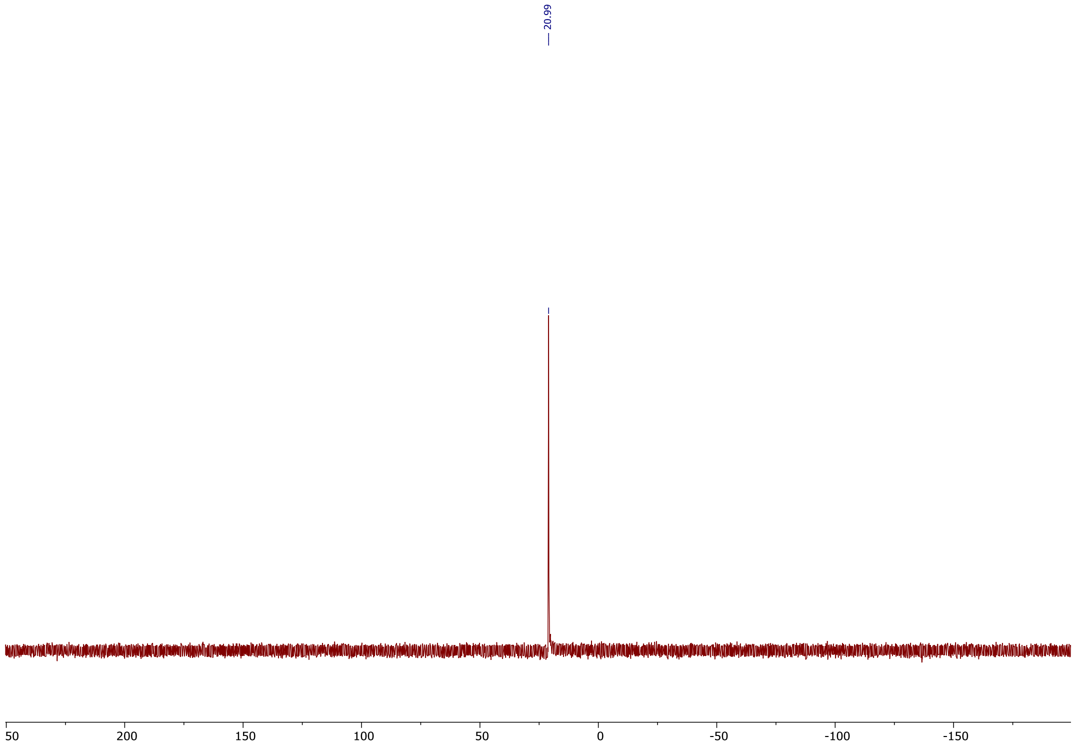

Diethyl (3-aminobicyclo[1.1.1]pentan-1-yl)phosphonate hydrochloride, 16

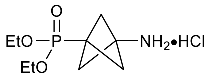

^{1}H NMR (400 MHz, DMSO-d$_6$)

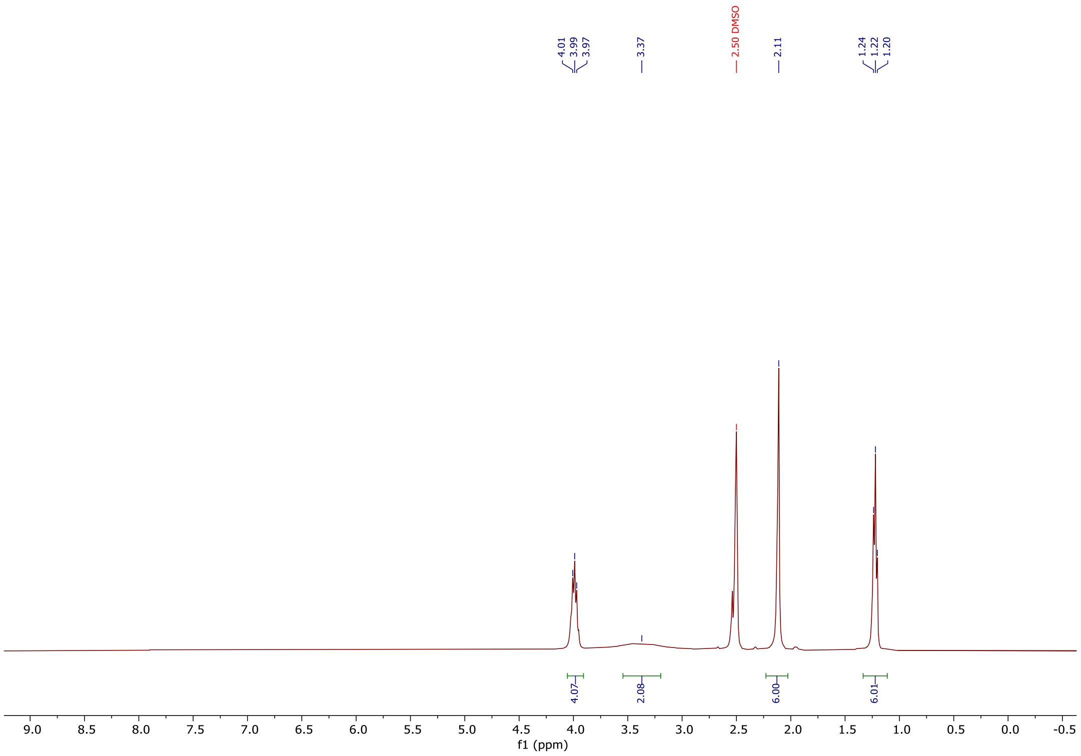

^{13}C NMR (126 MHz, DMSO-d$_6$)

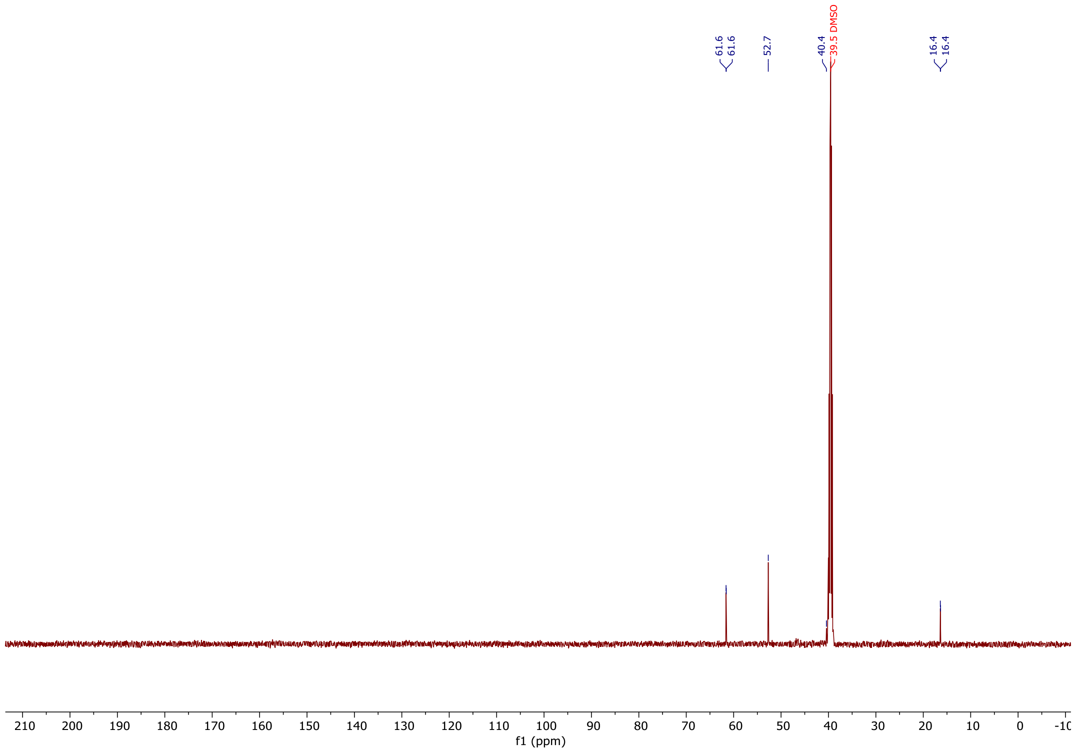

$^{31}P\{^1H\}$ NMR (162 MHz, DMSO-d_6)

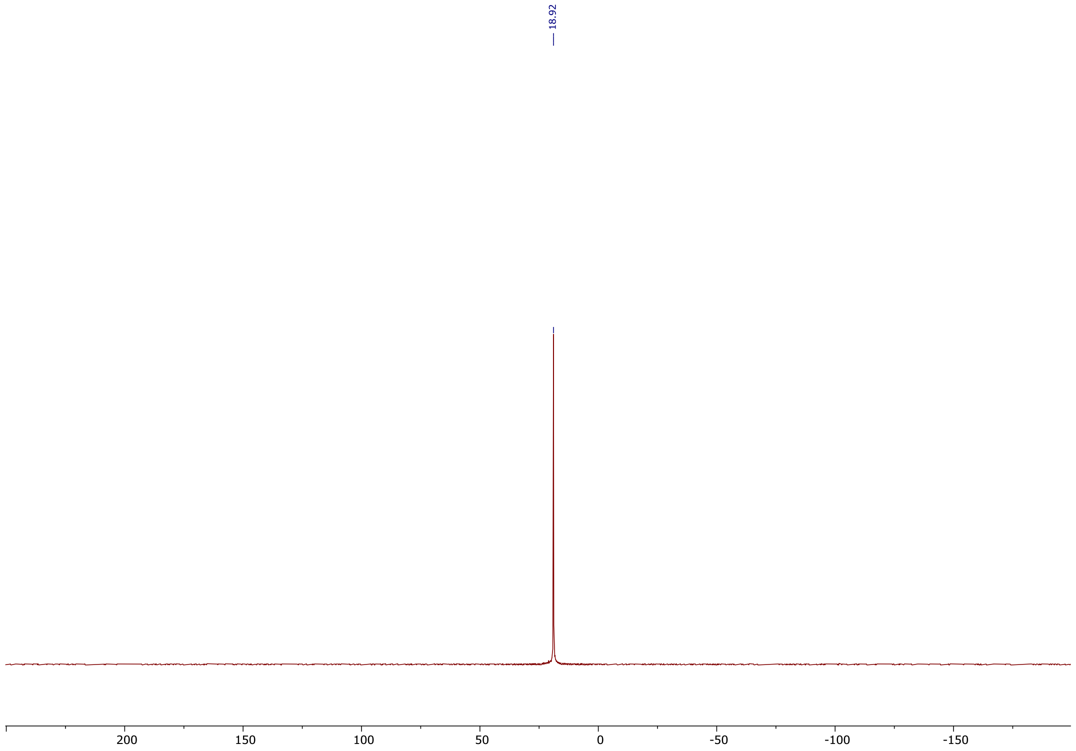

7. X-Ray Crystallography

7.1 X-Ray Crystal Structures

Low temperature single crystal X-ray diffraction data were collected using Oxford Diffraction (Rigaku) SuperNovae diffractometers at 150 K. These data were reduced using CrysAlisPro, solved using SuperFlip66 and the structures were refined using CRYSTALS. Further details about the refinements are documented in the CIF.

The crystallographic data for **3aa-Br**, **5a-I**, **10d-I** and **11** have been deposited with the CCDC as entries CCDC 2206272 – 2206275.

3-((3-Bromobicyclo[1.1.1]pentan-1-yl)sulfonyl)-N,N-diethyl-4H-1,2,4-triazole-4-carboxamide, 3aa-Br

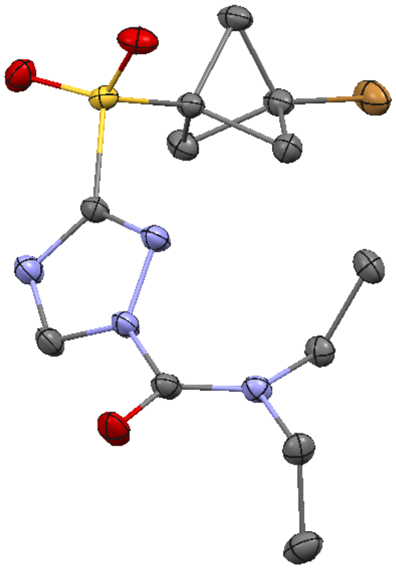

Structure of ***3aa-Br*** *from X-Ray diffraction studies. Displacement ellipsoid plots are drawn at 50% probability and Hydrogen atoms are omitted for clarity.*

CCDC Identification code	CCDC 2206272	
Empirical formula	C_{12} H_{17} B_1 N_4 O_3 S_1	
Formula Weight	377.26	
Temperature	150 K	
Wavelength	1.54184	
Crystal system	Triclinic	
Space Group	P -1	
Unit cell dimensions	a = 5.4252(1) Å	α = 84.376(2)°
	b = 8.3851(2) Å	β = 87.728(2)°
	c = 17.2856(4) Å	γ = 87.057(2)°
Volume	781.05(3) $Å^3$	
Z	2	
Density (calculated)	1.604 Mg/m^3	
Absorption coefficient	4.984 mm^{-1}	
F(000)	384.0	
Crystal size	0.04 x 0.19 x 0.21 mm^3	
Theta range for data collection	5.145 to 76.390°	
Index ranges	– 6<=h<=6, -10<=k<9, -21<=l<=21	
Reflections collected	9028	

Independent reflections	3003 [R(int) = 0.0256]
Completeness to theta = 76.39°	98.4%
Absorption correction	Multi Scan
Max. and min. transmission	0.490 and 0.820
Refinement method	Full-matrix least-squares on F^2
Data / restraints / parameters	3224 / 0 / 190
Goodness-of-fit on F2	1.0028
Final R indices [I>2sigma(I)]	R1 = 0.0256, wR2 = 0.0664
R indices (all data)	R1 = 0.0275, wR2 = 0.0683
Largest diff. peak and hole	0.42 and -0.39 e.$Å^{-3}$

1-Iodo-5-tosylbicyclo[3.1.1]heptane, 5a-I

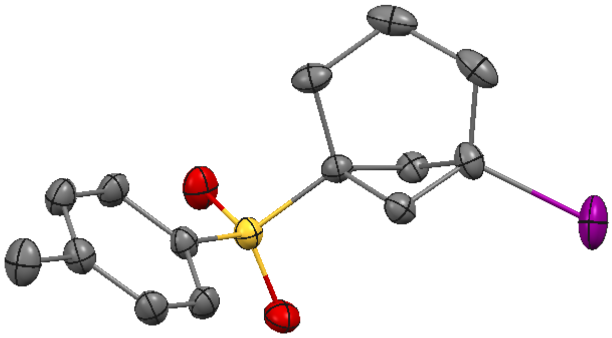

*Structure of **5a-I** from X-Ray diffraction studies. Displacement ellipsoid plots are drawn at 50% probability and Hydrogen atoms are omitted for clarity.*

CCDC Identification code	CCDC 2206273	
Empirical formula	$C_{14} H_{17} I_1 O_2 S_1$	
Formula Weight	376.24	
Temperature	150 K	
Wavelength	1.54184	
Crystal system	Orthorhombic	
Space Group	P b c a	
Unit cell dimensions	a = 10.2852(2) Å	α = 90°
	b = 11.9166(2) Å	β = 90°
	c = 23.6450(5) Å	γ = 90°
Volume	2896.83(10) Å^3	
Z	8	
Density (calculated)	1.725 Mg/m^3	
Absorption coefficient	18.654 mm^{-1}	
F(000)	1488.0	
Crystal size	0.08 x 0.15 x 0.20 mm^3	
Theta range for data collection	3.739 to 76.196°	
Index ranges	– 12<=h<=12, -13<=k<14, -28<=l<=29	
Reflections collected	8761	
Independent reflections	2664 [R(int) = 0.0402]	
Completeness to theta = 76.39°	98.4%	
Absorption correction	Multi Scan	
Max. and min. transmission	0.220 and 0.050	

Refinement method	Full-matrix least-squares on F^2
Data / restraints / parameters	3026 / 0 / 163
Goodness-of-fit on F2	0.9917
Final R indices [I>2sigma(I)]	R1 = 0.0402, wR2 = 0.1014
R indices (all data)	R1 = 0.0454, wR2 = 0.1066
Largest diff. peak and hole	1.36 and -1.83 e.$Å^{-3}$

1-((3-Iodo-3-(phenylsulfonyl)cyclobutyl)sulfonyl)-4-methylbenzene, 10d-I

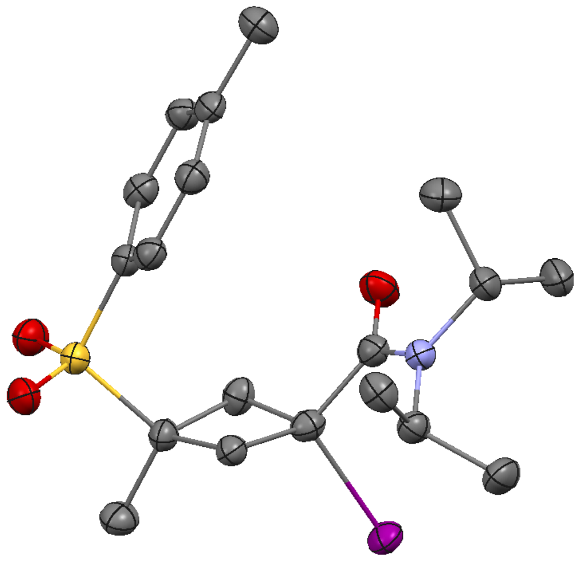

*Structure of **10d-I** from X-Ray diffraction studies. Displacement ellipsoid plots are drawn at 50% probability. Hydrogen atoms and disordered solvent are omitted for clarity.*

CCDC Identification code	CCDC 2206274	
Empirical formula	C_{19} H_{28} I_1 N_1 O_3 S_1	
Formula Weight	477.38	
Temperature	150 K	
Wavelength	1.54184	
Crystal system	Monoclinic	
Space Group	P 21/c	
Unit cell dimensions	a = 9.6739(2) Å	α = 90°
	b = 13.3506(4) Å	β = 92.361°
	c = 15.7257(4) Å	γ = 90°
Volume	2029.29(9) $Å^3$	
Z	4	
Density (calculated)	1.563 Mg/m^3	
Absorption coefficient	13.499 mm^{-1}	
F(000)	968.0	
Crystal size	0.03 x 0.13 x 0.30 mm^3	
Theta range for data collection	4.346 to 76.231°	
Index ranges	– 9<=h<=12, -16<=k<16, -19<=l<=18	
Reflections collected	8855	
Independent reflections	3683 [R(int) = 0.0399]	

Completeness to theta = 76.39°	99.3%
Absorption correction	Multi Scan
Max. and min. transmission	0.120 and 0.670
Refinement method	Full-matrix least-squares on F^2
Data / restraints / parameters	4203 / 0 / 226
Goodness-of-fit on F2	1.0286
Final R indices [I>2sigma(I)]	R1 = 0.0399, wR2 = 0.0992
R indices (all data)	R1 = 0.0474, wR2 = 0.1095
Largest diff. peak and hole	0.91 and -1.01 e.Å^{-3}

Methyl-2-(bis(tert-butoxycarbonyl)amino)-3-(3-tosylbicyclo[1.1.1]pentan-1-yl)propanoate, 11

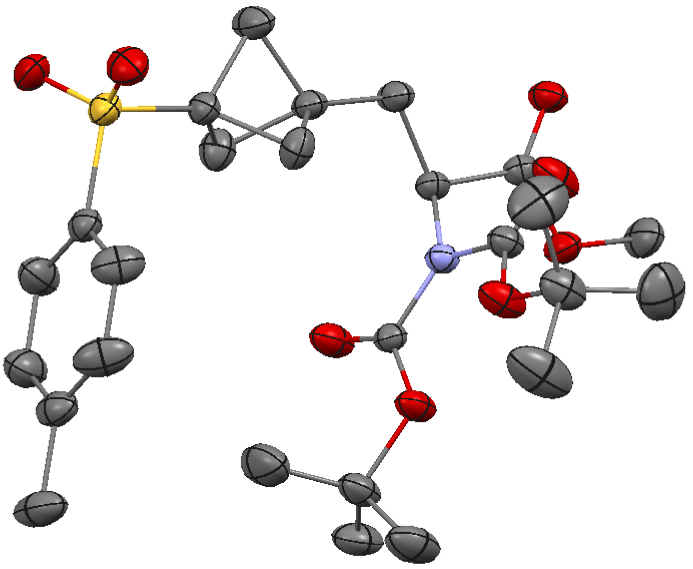

*Structure of **11** from X-Ray diffraction studies. Displacement ellipsoid plots are drawn at 50% probability and Hydrogen atoms are omitted for clarity.*

CCDC Identification code	CCDC 2206275	
Empirical formula	$C_{26} H_{37} N_1 O_8 S_1$	
Formula Weight	599.55	
Temperature	150 K	
Wavelength	1.54180	
Crystal system	Monoclinic	
Space Group	P 21/n	
Unit cell dimensions	a = 19.4393(4) Å	α = 90°
	b = 11.5541(2) Å	β = 104.915(2)°
	c = 28.8409(7) Å	γ = 90°
Volume	6259.5(2) $Å^3$	
Z	8	
Density (calculated)	1.272 Mg/m^3	
Absorption coefficient	2.706 mm^{-1}	
F(000)	2540.3	
Crystal size	0.08 x 0.12 x 0.20 mm^3	
Theta range for data collection	4.142 to 76.399°	
Index ranges	-24<=h<=24, -11<=k<14, -36<=l<=35	

Reflections collected	12983
Independent reflections	10038 [R(int) = 0.0526]
Completeness to theta = 76.39°	98.9%
Absorption correction	Multi Scan
Max. and min. transmission	0.680 and 0.810
Refinement method	Full-matrix least-squares on F^2
Data / restraints / parameters	12973 / 176 / 771
Goodness-of-fit on F2	1.0003
Final R indices [I>2sigma(I)]	R1 = 0.0526, wR2 = 0.1269
R indices (all data)	R1 = 0.0706, wR2 = 0.1445
Largest diff. peak and hole	0.72 and -0.61 e.$Å^{-3}$

8. References

1. Li, Y.; Li, X.; Li, X.; Shi, D., Highly E-Selective Synthesis of α-Fluoro-β-arylalkenyl Sulfones from gem-Difluoroalkenes with Sodium Sulfinates. *J. Org. Chem.,* **2021**, *86*, 6983-6993.

2. Sieber, G., Über die Spaltung von Tetrahydrofuran mit Methansulfobromid. *Justus Liebigs Ann. Chem.,* **1960**, *631*, 180-184.

3. Bär, R. M.; Gross, P. J.; Nieger, M.; Bräse, S., Sodium Bicyclo[1.1.1]pentanesulfinate: A Bench-Stable Precursor for Bicyclo[1.1.1]pentylsulfones and Bicyclo-[1.1.1]pentanesulfonamides. *Chem. Eur. J.,* **2020**, *26*, 4242-4245.

4. Pickford, H. D.; Nugent, J.; Owen, B.; Mousseau, J. J.; Smith, R. C.; Anderson, E. A., Twofold Radical-Based Synthesis of *N*,*C*-Difunctionalized Bicyclo[1.1.1]pentanes. *J. Am. Chem. Soc.,* **2021**, *143*, 9729-9736.

5. Wiberg, K. B.; Waddell, S. T., Reactions of [1.1.1]Propellane. *J. Am. Chem. Soc.,* **1990**, *112*, 2194-2216.

6. Nils Frank; Jeremy Nugent; Bethany Shire, H. P.; Patrick Rabe, A. S., Tryfon Zarganes-Tzitzikas,; Thomas Grimes*, et al.*, Synthesis of meta-substituted arene bioisosteres from [3.1.1]propellane. **2022**, *pre-print, DOI: 10.26434/chemrxiv-2022-6k9sl.*

7. Mcnamee, R. E.; Haugland, M. M.; Nugent, J.; Chan, R.; Christensen, K. E.; Anderson, E. A., Synthesis of 1,3-disubstituted bicyclo[1.1.0]butanes *via* directed bridgehead functionalization. *Chem. Sci.,* **2021**, *12*, 7480-7485.

8. Mondanaro, K. R.; Dailey, W. P., [1.1.1]Propellane, [Tricyclo[1.1.1.01,3]pentane]. *Org. Synth.,* **1998**, *75*, 98.

9. Johnson, T. C.; Elbert, L., B.; Farley, A. J. M.; Gorman, T. W.; Genicot, C.*, et al.*, Direct sulfonylation of anilines mediated by visible light. *Chem. Sci.,* **2018**, *9*, 629-633.

10. Hudwekar, A. D.; Reddy, G. L.; Verma, P. K.; Gupta, S.; Vishwakarma, R. A.; Sawant, S. D., Transition Metal-free Single Step Approach for Arylated Pyrazolopyrimidinones and Quinazolinones Using Benzylamines/Benzylalcohols/Benzaldehydes. *ChemistrySelect,* **2017**, *2*, 4963-4968.

11. Flores Toque, H. A.; Priviero, F. B. M.; Teixeira, C. E.; Perissutti, E.; Fiorino, F.*, et al.*, Synthesis and Pharmacological Evaluations of Sildenafil Analogues for Treatment of Erectile Dysfunction. *J. Med. Chem.,* **2008**, *51*, 2807-2815.

12. Mcconville, M.; Fernández, J.; Angulo-Barturen, Í.; Bahamontes-Rosa, N.; Ballell-Pages, L.*, et al.*, Carbamoyl Triazoles, Known Serine Protease Inhibitors, Are a Potent New Class of Antimalarials. *J. Med. Chem.,* **2015**, *58*, 6448-6455.

13. Eliazyan, K. A.; Shahbazyan, L. V.; Pivazyan, V. A.; Ghazaryan, E. A.; Yengoyan, A. P., Synthesis of novel 1,3-substituted 1*H*-[1,2,4]-triazole-3-thiol derivatives. *Heteroat. Chem.,* **2010**, *20*, 405-410.

14. Sigma-Aldrich, Diversinates™: Any-Stage Functionalization of (Hetero)aromatic Scaffolds, https://www.sigmaaldrich.com/GB/en/technical-documents/technical-article/chemistry-and-synthesis/ch-functionalization/baran-diversinates.

15. Gualandi, A.; Mazzarella, D.; Ortega-Martínez, A.; Mengozzi, L.; Calcinelli, F.*, et al.*, Photocatalytic Radical Alkylation of Electrophilic Olefins by Benzylic and Alkylic Zinc-Sulfinates. *ACS Catal.,* **2017**, *7*, 5357-5362.

16. Kim, D.-K.; Um, H.-S.; Park, H.; Kim, S.; Choi, J.; Lee, C., Silyloxymethanesulfinate as a sulfoxylate equivalent for the modular synthesis of sulfones and sulfonyl derivatives. *Chem. Sci.,* **2020**, *11*, 13071-13078.

17. Zálešák, F.; Kováč, O.; Lachetová, E.; Šťastná, N.; Pospíšil, J., Unified Approach to Benzo[*d*]thiazol-2-yl-Sulfonamides. *J. Am. Chem. Soc.,* **2021**, *86*, 11291-11309.

18. Song, L.; Zhang, L.; Luo, S.; Cheng, J.-P., Visible-Light Promoted Catalyst-Free Imidation of Arenes and Heteroarenes. *Chem. Eur. J.,* **2014**, *20*, 14231-14234.
